# Supplementary material for: Photoinduced copper-catalyzed C–N coupling with trifluoromethylated arenes
Source: Nat Commun. 2023 Dec 14;14:8292. doi: 10.1038/s41467-023-44097-y (PMC10719352; doi:10.1038/s41467-023-44097-y)
Supplement: Supplementary file 1 — Supplementary Information [file 41467_2023_44097_MOESM1_ESM.pdf]

Supplementary Information

Photoinduced Copper-Catalyzed C–N Coupling with Trifluoromethylated Arenes

Jun Huang,[1,‡] Qi Gao,[1,‡] Tao Zhong,[1] Shuai Chen,[1] Wei Lin,[1,2] Jie Han,*[1] Jin Xie*[1]

[1]State Key Laboratory of Coordination Chemistry, Jiangsu Key Laboratory of Advanced Organic Materials, Chemistry and Biomedicine Innovation Center (ChemBIC), School of Chemistry and Chemical Engineering, Nanjing University, Nanjing 210023, China

[2]School of Chemistry and Environmental Engineering, Jiangsu University of Technology, Changzhou 213001, China

‡These authors contributed equally

Email: jie.han@nju.edu.cn; xie@nju.edu.cn

Table of Contents

1. General Information....2
2. Optimization of the Reaction Conditions....3
3. Reagents and Starting Materials....12
4. General Procedures....17
5. Characterization of Products....21
6. Downstream Transformation of Products....58
7. Mechanistic Studies....65
8. DFT Studies....76
9. X-Ray Crystal Data....84
10. Copies of NMR Spectra....87
11. References....214

General Information

All the reactions were conducted in oven-dried 8 or 4 mL vials under nitrogen atmosphere unless otherwise noted. Reagents were purchased from Bide Chemical, TCI, J&K or other commercial suppliers and used as received unless otherwise noted. Flash column chromatographic purification of products is accomplished using forced-flow chromatography on Al_2O_3 (neutral, 200-300 mesh). **^{1}H NMR**, **^{13}C NMR** and **^{19}F NMR** spectra were recorded on a 500 MHz spectrometer in $CDCl_3$ (δ H = 7.26 ppm, δ C = 77.0 ppm as standard). Data for **^{1}H NMR** are reported as follows: chemical shift (ppm, scale), multiplicity, coupling constant (Hz), and integration. Data for **^{13}C NMR** are reported in terms of chemical shift (ppm, scale), multiplicity, and coupling constant (Hz). Abbreviations for signal couplings are: s, singlet; d, doublet; t, triplet; m, multiple. Gas chromatographic (GC) analyses are performed on a GC equipped with a flameionization detector and an Rtx@-65 (30 m × 0.32 mm ID × 0.25 μm df) column. GC-MS analyses are performed on a GC-MS with an EI mode. EI-HRMS and ESI-HRMS spectra were obtained on a Waters Premier GC-TOF MS and a Thermo Scientific Q Exactive HF Orbitrap-FTMS, respectively. FI-HRMS spectra were obtained on a JEOLAccuTOF-GCv4G-GCT MS. Melting points (m.p.) were determined with a digital electrothermal apparatus without further correction. IR spectra were recorded on a Thermo Scientific Nicolet 380 FT-IR spectrometer. The PR160L-390 nm LED lamps (40 W, λ = 360-420 nm, max = 390 nm) were purchased from Kessil.

2. Optimization of the Reaction Conditions

2.1 Reaction conditions of defluorinative C-N coupling of carbazoles

Supplementary Table 1. Screening of the base[a]

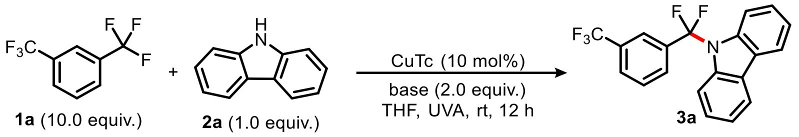

Entry	Base	Yield[b]
1	^{t}BuOLi	13%
2	^{t}BuONa	7%
3	^{t}BuOK	trace
4	Cs_2CO_3	N.D.
5	DBU	N.D.

[a] Reaction condition: trifluoromethyl-arenes (1.0 mmol, 10 equiv.), carbazole (0.1 mmol, 1 equiv.), CuTc (0.01 mmol, 10 mol%), base (0.2 mmol, 2 equiv.), THF (1 mL, 0.1 M), UVA, rt, 12 h. [b] GC yield with dodecane as internal standard.

Supplementary Table 2. Screening of the solvent[a]

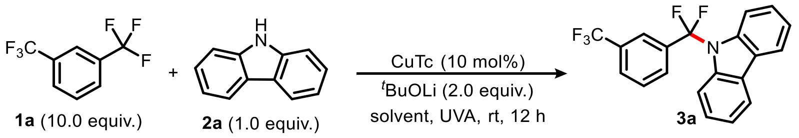

Entry	Solvent	Yield[b]
1	MeCN	trace
2	DMSO	N.D.
3	DMF	N.D.
4	Et_2O	21%
5	MTBE	21%
6	DME	trace

[a] Reaction condition: trifluoromethyl-arenes (1.0 mmol, 10 equiv.), carbazole (0.1 mmol, 1 equiv.), CuTc (0.01 mmol, 10 mol%), ^{t}BuOLi (0.2 mmol, 2 equiv.), solvent (1 mL, 0.1 M), UVA, rt, 12 h. [b] GC yield with dodecane as internal standard.

Supplementary Table 3. Screening of the concentrations[a]

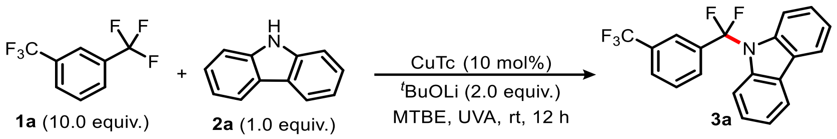

Entry	Volume	Concentration	Yield[b]
1	0.5 mL	0.2 M	13%
2	2.0 mL	0.05 M	24%
3	4.0 mL	0.025 M	30%
4	5.0 mL	0.02 M	30%

[a] Reaction condition: trifluoromethyl-arenes (1.0 mmol, 10 equiv.), carbazole (0.1 mmol, 1 equiv.), CuTc (0.01 mmol, 10 mol%), ^{t}BuOLi (0.2 mmol, 2 equiv.), MTBE, UVA, rt, 12 h. [b] GC yield with dodecane as internal standard.

Supplementary Table 4. Screening of the light source[a]

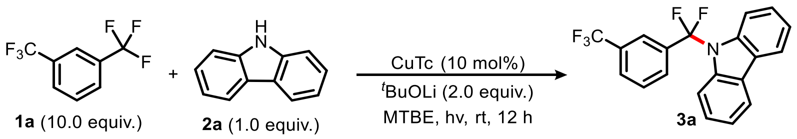

Entry	Lamp	Yield[b]
1	370 nm LED	55%
2	390 nm LED	57%
3	Blue LED	2%
4	White LED	6%
5	dark	N.D.

[a] Reaction condition: trifluoromethyl-arenes (1.0 mmol, 10 equiv.), carbazole (0.1 mmol, 1 equiv.), CuTc (0.01 mmol, 10 mol%), ^{t}BuOLi (0.2 mmol, 2 equiv.), MTBE (4 mL, 0.025 M), hv, rt, 12 h. [b] GC yield with dodecane as internal standard.

Supplementary Table 5. Screening of the ligand[a,b]

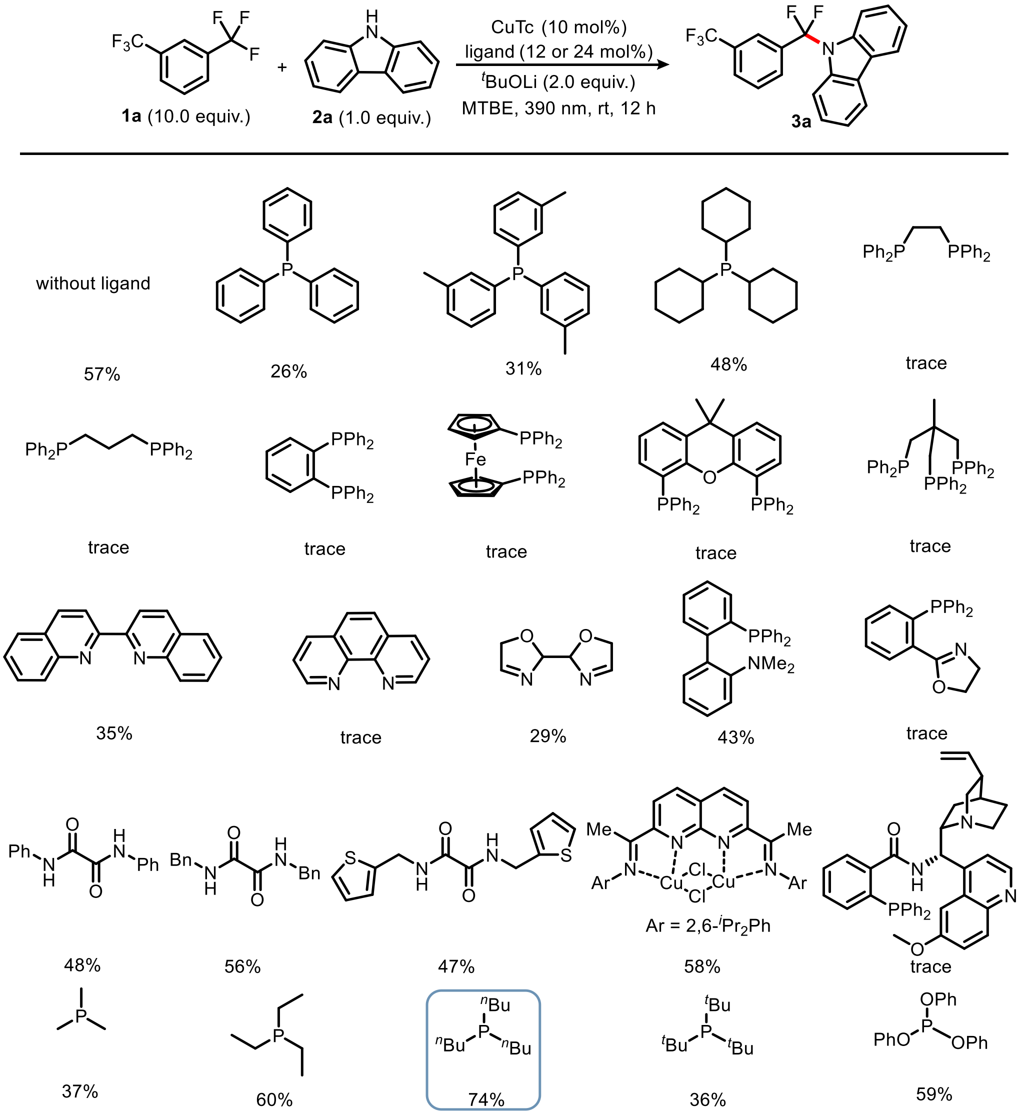

[a] Reaction condition: trifluoromethyl-arenes (1.0 mmol, 10 equiv.), carbazole (0.1 mmol, 1 equiv.), CuTc (0.01 mmol, 10 mol%), ligand (0.012 or 0.024 mmol, 12 or 24 mol%), ^{t}BuOLi (0.2 mmol, 2 equiv.), MTBE (4 mL, 0.025 M), 390 nm LED, rt, 12 h. [b] GC yield with dodecane as internal standard.

Discussion: Various ligands have been investigated (in order to ensure the reproducibility of the reaction, the equivalent of the ligand is appropriately increased-12 mol% for bidentate ligand and 24 mol% for monodentate ligand). The results have been shown here. Most of the ligands inhibit the reaction, but alkyl phosphine ligands can further improve the yield, especially $^{n}Bu_3P$ (subsequent control experiments have also shown that $^{n}Bu_3P$ can shorten the reaction time).

Supplementary Table 6. Screening of the Cu salts[a]

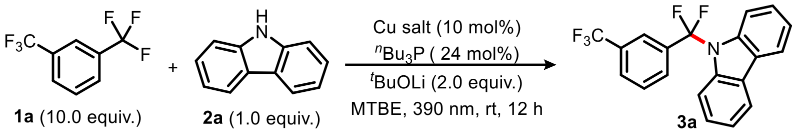

Entry	Cu salts	Yield[b]
1	CuCl	79%
2	CuBr	83%
3	CuOAc	34%
4	CuCN	81%
5	$Cu(MeCN)_4BF_4$	77%
6	$Cu(MeCN)_4PF_6$	76%
7	$CuCl_2$	73%

[a] Reaction condition: trifluoromethyl-arenes (1.0 mmol, 10 equiv.), carbazole (0.1 mmol, 1 equiv.), Cu salt (0.01 mmol, 10 mol%), ^{n}Bu$_3$P (0.024 mmol, 24 mol%), ^{t}BuOLi (0.2 mmol, 2 equiv.), MTBE (4 mL, 0.025 M), 390 nm LED, rt, 12 h. [b] GC yield with dodecane as internal standard.

Supplementary Table 7. Screening of the catalyst loading and the amount of 1a[a]

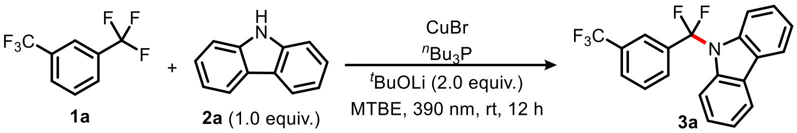

Entry	CuBr / ^{n}Bu$_3$P	**1a**	Yield[b]
1	5 mol% / 12 mol%	10 equiv.	84%
2	2 mol% / 4.8 mol%	10 equiv.	83%
3	2 mol% / 4.8 mol%	5 equiv.	73%
4	2 mol% / 4.8 mol%	3 equiv.	51%
5	2 mol% / 4.8 mol%	2 equiv.	37%
6	2 mol% / 4.8 mol%	1 equiv.	18%

[a] Reaction condition: trifluoromethyl-arenes, carbazole (0.1 mmol, 1 equiv.), CuBr, ^{n}Bu$_3$P, ^{t}BuOLi (0.2 mmol, 2 equiv.), MTBE (4 mL, 0.025 M), 390 nm LED, rt, 12 h. [b] GC yield with dodecane as internal standard.

Discussion: The catalyst loading could be decreased to 2 mol%. Considering the balance between the amount of **1a** used and the yield of **3a**, we decided to use 5.0 equiv. **1a**.

Supplementary Table 8. Screening of the reaction time[a]

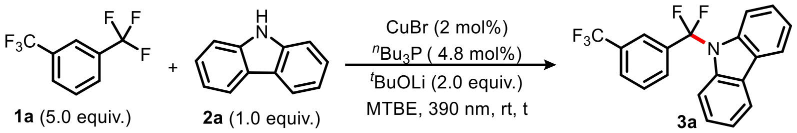

Entry	Reaction time	Yield[b]
1	0.5 h	69%
2	1 h	73% (72%[c])
3	2 h	72%
4	6 h	73%

[a] Reaction condition: trifluoromethyl-arenes (0.5 mmol, 5 equiv.), carbazole (0.1 mmol, 1 equiv.), CuBr (0.002 mmol, 2 mol%), $^{n}Bu_3P$ (0.0048 mmol, 4.8 mol%), ^{t}BuOLi (0.2 mmol, 2 equiv.), MTBE (4 mL, 0.025 M), 390 nm LED, rt, t. [b] GC yield with dodecane as internal standard. [c] Isolated yield.

Supplementary Table 9. Control experiments[a]

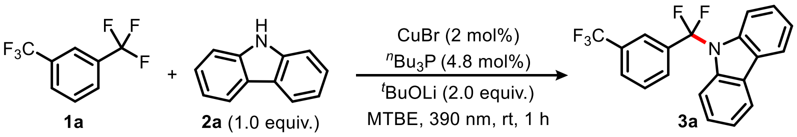

Entry	Reaction conditions	Yield[b]
1	**1a** (3.0 equiv.)	47%
2	**1a** (1.0 equiv.)	18%
3	KO^{t}Bu instead of LiO^{t}Bu	trace
4	MeCN instead of MTBE	N.D.
5	Et_2O instead of MTBE	71%
6	CuCl instead of CuBr	72%
7	CuI instead of CuBr	14%
8	H_2O (0.5 equiv)	20%
9	no CuBr	N.D.
10	no $^{n}Bu_3P$	13%
11	no light	N.D.

[a] Standard condition: trifluoromethyl-arenes (0.5 mmol, 5 equiv.), carbazole (0.1 mmol, 1 equiv.), CuBr (0.002 mmol, 2 mol%), $^{n}Bu_3P$ (0.0048 mmol, 4.8 mol%), ^{t}BuOLi (0.2 mmol, 2 equiv.), MTBE (4 mL, 0.025M), 390 nm, rt, 1 h. [b] GC yield with dodecane as internal standard.

Discussion: Although the dimmer of the corresponding difluorobenzylic radical could be detected as the major by-product, its yield typically remained below 5%.

2.2 Reaction conditions of the tandem C–N coupling and defluorination

Supplementary Table 10. Screening of additional Cu salts and ligands[a]

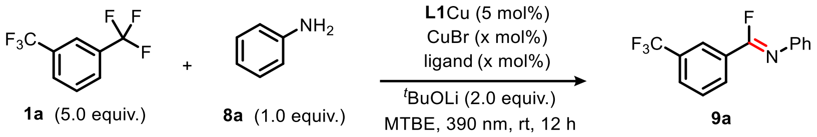

Entry	Cu salts	Ligand	Yield[b]
1	-	-	35%
2	CuBr (10 mol%)	-	7%
3	CuBr (10 mol%)	dtbpy (10 mol%)	4%
4	CuBr (10 mol%)	2, 2'-oxazoline (10 mol%)	7%
5	CuBr (10 mol%)	terpy (10 mol%)	9%

[a] Reaction conditions: trifluoromethyl-arenes (0.5 mmol, 5 equiv.), anilines (0.1 mmol, 1 equiv.), **L1**Cu (0.005 mmol, 5 mol%), CuBr, ligand, ^{t}BuOLi (0.2 mmol, 2 equiv.), MTBE (1 mL, 0.1 M), 390 nm, rt, 12 h. [b] GC yield with dodecane as internal standard.

Discussion: Although the corresponding α,α-difluoromethylamine can be detected by GC-MS, its yield has always been less than 5%. Thus we speculate that it is easily converted to imidoyl fluorides **9** under basic conditions.

Supplementary Table 11. Screening of the light source[a]

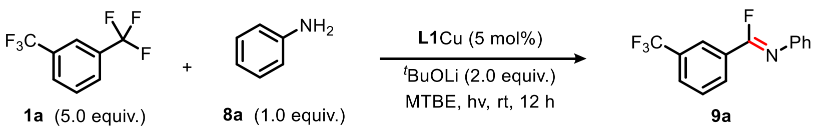

Entry	Lamp	Yield[b]
1	UVA	3%
2	370 nm	20%
3	390 nm	35%
4	Blue LED	13%
5	White LED	8%
6	50 °C without hv	NR

[a] Reaction conditions: trifluoromethyl-arenes (0.5 mmol, 5 equiv.), anilines (0.1 mmol, 1 equiv.), **L1**Cu (0.005 mmol, 5 mol%), ^{t}BuOLi (0.2 mmol, 2 equiv.), MTBE (1 mL, 0.1M), hv, rt, 12 h. [b] GC yield with dodecane as internal standard.

Supplementary Table 12. Screening of the catalyst loading and amount of **1a**[a]

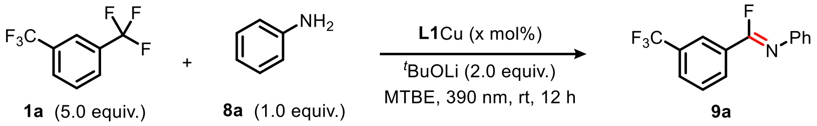

Entry	(Cbzdiphos^{Cy})Cu	**1a**:**8a**	Yield[b]
1	10 mol%	5:1	36%
2	5 mol%	5:1	35%
3	2 mol%	5:1	31%
4	5 mol%	3:1	35%
5	5 mol%	1:1	26%
6	5 mol%	1:2	28%

[a] Reaction conditions: trifluoromethyl-arenes, anilines, **L1**Cu, *t*BuOLi (0.2 mmol, 2 equiv.), MTBE (1 mL, 0.1M), 390 nm, rt, 12 h. [b] GC yield with dodecane as internal standard.

Supplementary Table 13. Screening of the solvent and concentration[a]

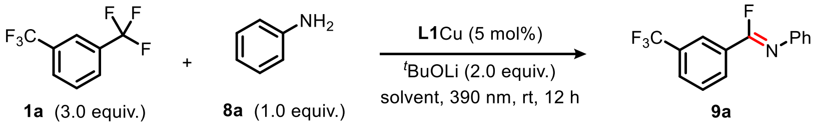

Entry	Solvent	Yield[b]
1	MTBE (1.0 mL)	35%
2	Et_2O (1.0 mL)	45%
3	EA (1.0 mL)	NR
4	THF (1.0 mL)	NR
5	toluene (1.0 mL)	32%
6	*n*hexane (1.0 mL)	26%
7	MeCN (1.0 mL)	8%
8	DMSO (1.0 mL)	NR
9	Et_2O (0.5 mL)	45%
10	Et_2O (2.0 mL)	45%

[a] Reaction conditions: trifluoromethyl-arenes (0.3 mmol, 3 equiv.), anilines (0.1 mmol, 1 equiv.), **L1**Cu (0.005 mmol, 5 mol%), *t*BuOLi (0.2 mmol, 2 equiv.), solvent, 390 nm, rt, 12 h. [b] GC yield with dodecane as internal standard.

Supplementary Table 14. Screening of the base and equiv.[a]

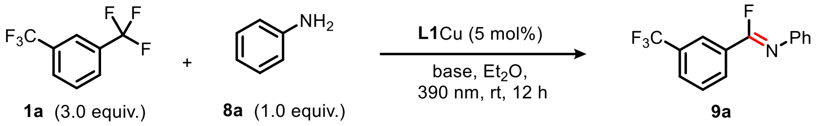

Entry	base	Yield[b]
1	KF (2 equiv.)	NR
2	K_2CO_3 (2 equiv.)	NR
3	K_3PO_4 (2 equiv.)	NR
4	NaOAc (2 equiv.)	NR
5	LiOMe (2 equiv.)	8%
6	Pyridine (2 equiv.)	NR
7	DBU (2 equiv.)	NR
8	DABCO (2 equiv.)	NR
9	^{t}BuOLi (1.5 equiv.)	29%
10	^{t}BuOLi (3 equiv.)	50%

[a] Reaction conditions: trifluoromethyl-arenes (0.3 mmol, 3 equiv.), anilines (0.1 mmol, 1 equiv.), **L1**Cu (0.005 mmol, 5 mol%), base, Et_2O (1 mL, 0.1 M), 390 nm, rt, 12 h. [b] GC yield with dodecane as internal standard.

Supplementary Table 15. Screening of the temperature and reaction time[a]

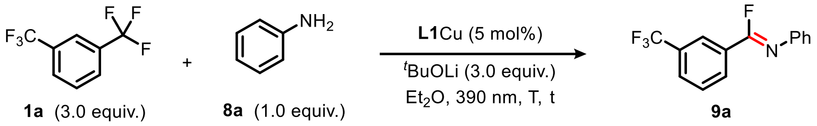

Entry	T	t	Yield[b]
1	rt (27 °C)	12 h	50%
2	0 °C	12 h	46%
3	45 °C	12 h	49%
4	rt (27 °C)	1 h	44%
5	rt (27 °C)	2 h	50% (48%[c])
6	rt (27 °C)	4 h	49%
7	rt (27 °C)	2 h	50% (48%[c, d])

[a] Reaction conditions: trifluoromethyl-arenes (0.3 mmol, 3 equiv.), anilines (0.1 mmol, 1 equiv.), **L1**Cu (0.005 mmol, 5 mol%), ^{t}BuOLi (0.3 mmol, 3 equiv.), Et_2O (1 mL, 0.1 M), 390 nm. [b] GC yield with dodecane as internal standard. [c] Isolated yield. [d] Catalyst is CuBr (0.005 mmol, 5 mol%) and **L1** (0.005 mmol, 5 mol%).

2.3 Reaction conditions of the 1,2-difluoroalkylamination reaction

Supplementary Table 16. Screening of the ratio of substrates[a]

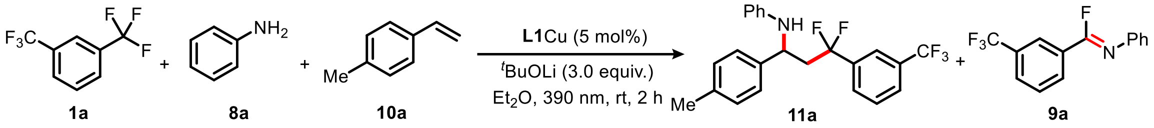

Entry	**1a**:**8a**:**10a**	Yield of **11a**[b]	Yield of **9a**[b]
1	3:1:3	71%(67%[c])	8%
2	3:1:2	60%	11%
3	3:1:1	45%	15%
4	2:1:3	53%	7%
5	1:2:3	30%	5%

[a] Reaction conditions: trifluoromethyl-arenes, anilines, alkenes, **L1**Cu (0.005 mmol, 5 mol%), ^{t}BuOLi (0.3 mmol, 3 equiv.), Et_2O (1 mL, 0.1M), 390 nm, rt, 2 h. [b] GC yield with dodecane as internal standard. [c] Isolated yield.

Supplementary Table 17. Screening of other alkenes

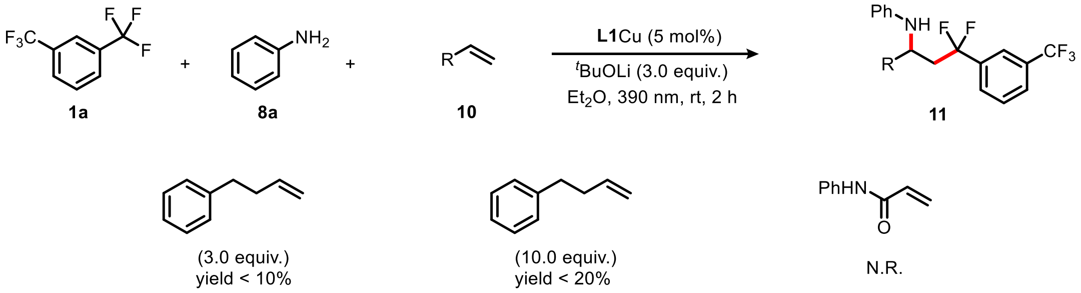

Discussion: Although the desired product can be detected on GC-MS when aliphatic olefins such as 4-Phenyl-1-butene is used, the major product in the reaction system is still the two-component product imidoyl fluoride **9**.

3. Reagents and Starting substrates

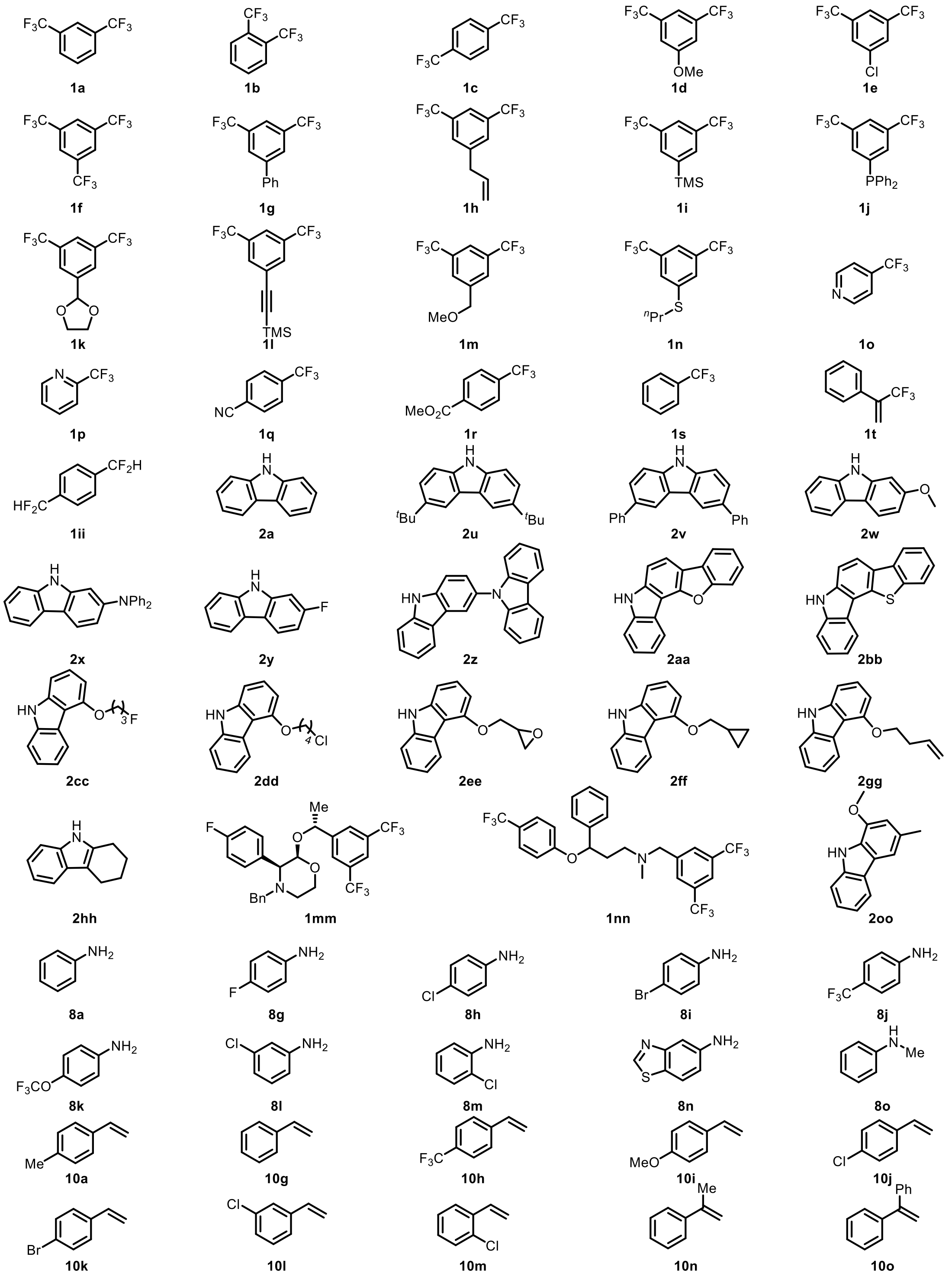

Supplementary Figure 1. Substrates used in the reaction

CuBr: purchased from Alfa Aesar Chemicals Inc. and use as received.

^{n}Bu$_3$P: purchased from TCI Chemicals Inc. and use as received.

P(O2,4^{t}Bu$_2$-Ph)$_3$: purchased from Sinocompound Chemicals Inc. and use as received.

LiO^{t}Bu, MTBE: purchased from J&K Chemicals Inc. and use as received.

1a-1f, **1o -1s**, **1ii**, **2a**, **2u-2bb**, **2oo**, **8a-8o, 10a-10o**: purchased from Bide Chemicals Inc. and use as received.

1g,[1] **1m**,[1] **1h**,[2] **1i**,[3] **1j**,[4] **1k**,[5] **1l**,[6] **1n**,[7] **1mm**,[9] **1nn**[9]are prepared according to previous reported procedures.

α-(trifluoromethyl) styrenes **1t** is prepared according to previous reported procedures.[8]

2cc, **2dd**, **2ff**, **2gg** are prepared according to the following procedures.

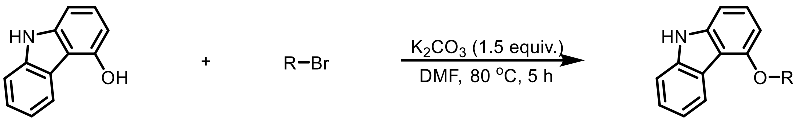

A 100 mL round-bottom flask equipped with a magnetic stir bar, are charged with 9H-carbazol-4-ol (1.83g, 10 mmol, 1.0 equiv.), alkyl bromide (11 mmol, 1.1 equiv.), K_2CO_3 (2.07g, 15 mmol, 1.5 equiv.), and DMF (50 mL). The reaction mixture is allowed to be stirred at 80 °C for 5 h. Then the mixture is poured into water (50 mL) and extracted with EA (30 mL* 3). The combined organic solution is washed with brine, dried over anhydrous sodium sulfate, filtered and concentrated. The product can be isolated by flash column chromatography.

Characterization of unknown substrates

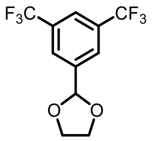

1k

2-[3,5-bis(trifluoromethyl)phenyl]-1,3-dioxolane (1k)

IR: ν = 2897, 1359, 1276, 1169, 1124, 947, 891, 842, 705, 680 cm^{-1}.

^{1}H NMR (500 MHz, Chloroform-*d*) δ 7.95 (s, 2H), 7.89 (s, 1H), 5.90 (s, 1H), 4.16 – 4.06 (m, 4H).

^{19}F NMR (471 MHz, Chloroform-*d*) δ -63.00 (s).

^{13}C NMR (126 MHz, Chloroform-*d*) δ 140.9, 131.8 (q, *J* = 33.5 Hz), 127.0 – 126.7 (m), 123.2 (q, *J* = 272.6 Hz), 123.2 – 122.8 (m), 101.9, 65.5.

HRMS (FI) calcd. for $C_{11}H_8F_6O_2$ $[M]^+$: 286.0423, found: 286.0417.

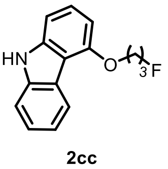

2cc

4-(3-fluoropropoxy)-9H-carbazole (2cc)

m.p.: 102.6-104.2 °C

IR: ν = 3394, 2937, 1508, 1452, 1439, 1264, 1082, 786, 751, 721 cm^{-1}.

^{1}H NMR (500 MHz, Chloroform-*d*) δ 8.28 (dd, *J* = 7.8, 1.0 Hz, 1H), 8.01 (s, 1H), 7.44 – 7.37 (m, 2H), 7.34 (t, *J* = 8.0 Hz, 1H), 7.29 – 7.23 (m, 1H), 7.04 (d, *J* = 8.1 Hz, 1H), 6.69 (d, *J* = 8.0 Hz, 1H), 4.87 (t, *J* = 5.9 Hz, 1H), 4.78 (t, *J* = 5.9 Hz, 1H), 4.38 (t, *J* = 6.0 Hz, 2H), 2.49 – 2.31 (m, 2H).

^{13}C NMR (126 MHz, Chloroform-*d*) δ 155.2, 140.9, 138.7, 126.7, 124.9, 122.8, 122.6, 119.6, 112.6, 110.0, 103.6, 101.0, 81.0 (d, *J* = 164.8 Hz), 63.6 (d, *J* = 5.5 Hz), 30.6 (d, *J* = 20.1 Hz).

HRMS (FI) calcd. for $C_{15}H_{14}ONF$ $[M]^+$: 243.1054, found: 243.1052.

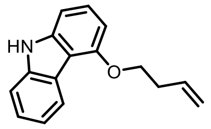

2gg

4-(but-3-en-1-yloxy)-9H-carbazole (2gg)

m.p.: 94.0-95.2 °C

IR: ν = 3404, 1585, 1506, 1453, 1439, 1259, 1094, 749, 715, 541 cm^{-1}.

^{1}H NMR (500 MHz, Chloroform-*d*) δ 8.35 (dd, *J* = 7.8, 1.0 Hz, 1H), 7.96 (s, 1H), 7.43 – 7.36 (m, 2H), 7.33 (t, *J* = 8.0 Hz, 1H), 7.28 – 7.24 (m, 1H), 7.01 (d, *J* = 8.1 Hz, 1H), 6.68 (d, *J* = 7.9 Hz, 1H), 6.08 (ddt, *J* = 17.0, 10.3, 6.8 Hz, 1H), 5.31 (dq, *J* = 17.1, 1.7 Hz, 1H), 5.19 (dq, *J* = 10.2, 1.3 Hz, 1H), 4.29 (t, *J* = 6.5 Hz, 2H), 2.81 – 2.72 (m, 2H).

^{13}C NMR (126 MHz, Chloroform-*d*) δ 155.5, 140.9, 138.7, 134.9, 126.6, 124.9, 123.1, 122.7, 119. 6, 117.0, 112.7, 109.9, 103.4, 101.0, 67.2, 34.0.

HRMS (FI) calcd. for $C_{16}H_{15}ON$ $[M]^+$: 237.1148, found: 237.1150.

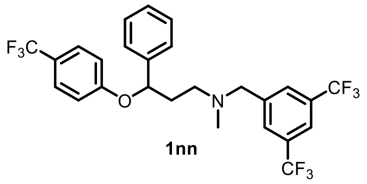

1nn

***N*-(3,5-bis(trifluoromethyl)benzyl)-N-methyl-3-phenyl-3-(4(trifluoromethyl)phenoxy)**

propan-1-amine (1nn)

IR: ν = 2958, 1326, 1276, 1250, 1165, 1107, 1067, 835, 701, 682 cm^{-1}.

^{1}H NMR (500 MHz, Chloroform-*d*) δ 7.73 (s, 2H), 7.70 (s, 1H), 7.41 (d, *J* = 8.7 Hz, 2H), 7.35 – 7.24 (m, 5H), 6.83 (d, *J* = 8.5 Hz, 2H), 5.25 (dd, *J* = 8.5, 4.5 Hz, 1H), 3.64 – 3.52 (m, 2H), 2.72 – 2.62 (m, 1H), 2.57 – 2.49 (m, 1H), 2.24 (s, 3H), 2.23 – 2.15 (m, 1H), 2.07 – 1.98 (m, 1H).

^{19}F NMR (471 MHz, Chloroform-*d*) δ -61.67 (s, 3F), -62.78 (s, 6F).

^{13}C NMR (126 MHz, Chloroform-*d*) δ 160.5, 142.1, 141.0, 131.5 (q, *J* = 33.1 Hz), 128. 8, 128.8 – 128.6 (m), 127.9, 126.8 (q, *J* = 3.8 Hz), 125.7, 123.3 (q, *J* = 272.2 Hz), 123.3 (q, *J* = 273.4 Hz), 122.8 (q, *J* = 32.7 Hz), 121.2 – 120.8 (m), 115.5, 78.1, 61.6, 53.5, 42.2, 36.7.

HRMS (FI) calcd. for $C_{26}H_{22}ONF_9$ $[M]^+$: 535.1552, found: 535.1548.

Synthesis and characterization of the ligand

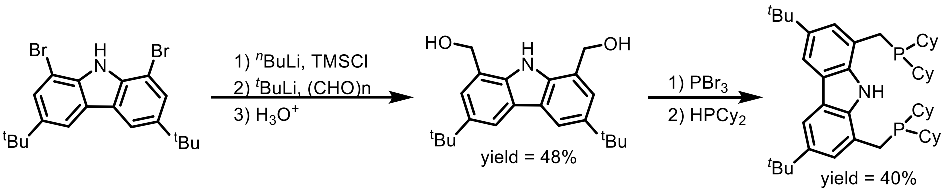

(3,6-Di-tert-butyl-9H-carbazole-1,8-diyl)dimethanol

It is prepared according to previous reported procedures.[10]

3,6-di-tert-butyl-1,8-bis((dicyclohexylphosphanyl)methyl)-9H-carbazole (L1)

PBr_3 (0.42 mL, 4.4 mmol) is added dropwise to a solution of (3,6-di-tert-butyl-9H-carbazole-1,8-diyl)dimethanol (678 mg, 2.0 mmol) in 10 mL of dichloromethane at 0 °C. After stirring for 1 h, hydrolysis is performed at 0 °C. The organic layer is washed with saturated $NaHCO_3$ and brine, dried over $MgSO_4$, filtered, and dried in vacuo to afford a white solid. The obtained 1,8-bis(bromomethyl)-3,6-di-tert-butyl-9Hcarbazole is transferred to a 50 ml round-bottom flask charged with a magnetic stir bar, then it is evacuated and backfilled with nitrogen (3 cycles). 10 mL dichloromethane and $HPCy_2$ (0.9 mL, 4.4 mmol) are added. The mixture is vigorously stirred overnight at room temperature. The solvent is removed in vacuo and the obtained solid is washed with PE (10 mL, 3 times). The white solid is then transferred to another 50 ml round-bottom flask charged with a magnetic stir bar, then it is evacuated and backfilled with nitrogen (3 cycles). NEt_3 (4.0 mL, 30 mmol) and 10 mL dichloromethane are added, the suspension is stirred for at rt 12 h. After removing solvents in vacuo, the residue is dissolved in 20 mL toluene and allowed to be stirred at rt for 4 h. After filtration, the solvent is removed, slow addition of PE to the mixture afford **L1** as a white solid (561 mg, 0.8 mmol, 40%).

IR: ν = 3437, 2919, 2846, 1493, 1444, 1302, 1246, 853, 828, 738 cm^{-1}.
^{1}H NMR (400 MHz, Benzene-d_6) δ 9.57 (t, J = 4.9 Hz, 1H), 8.17 (d, J = 1.7 Hz, 2H), 7.52 – 7.44 (m, 2H), 3.14 (s, 4H), 1.94 – 1.60 (m, 24H), 1.51 (s, 18H), 1.45 – 1.34 (m, 4H), 1.25 – 1.11 (m, 16H).
^{31}P NMR (162 MHz, Benzene-d_6) δ 2.64.
^{13}C NMR (101 MHz, Benzene-d_6) δ 142.4, 138.5, 125.0, 124.9, 122.3, 114.6, 34.8, 34.3, 34.1, 32.3, 30.3 (t, J = 7.2 Hz), 29.6 (t, J = 4.2 Hz), 27.8 (t, J = 5.4 Hz), 27.6 (t, J = 4.1 Hz), 27.0.
HRMS (ESI) calcd. for $C_{46}H_{72}NP_2$ $[M+H]^+$: 700.5135, found: 700.5131.

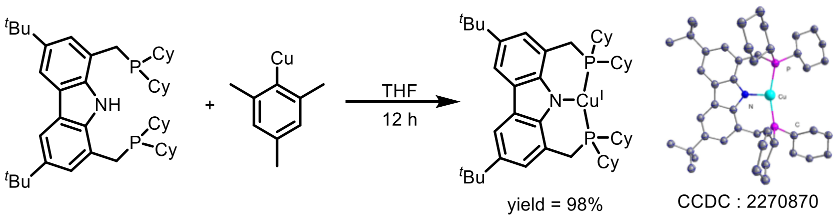

L1Cu

L1 (260 mg, 0.37 mmol) and mesitylcopper(I) (68 mg, 0.37 mmol) are dissolved in 5 mL THF under N_2 atmosphere, the mixture is stirred at rt for 12 h. The solvent is removed in vacuo to yield a light brown solid (275 mg, 0.36 mmol, 98%). Crystals suitable for single-crystal X-ray diffraction are obtained by recrystallization from EA and hexane at -20 °C.

^{1}H NMR (400 MHz, Benzene-d_6) δ 8.43 (d, J = 1.9 Hz, 2H), 7.41 (d, J = 1.9 Hz, 2H), 3.27 (t, J = 3.2 Hz, 4H), 1.66 (s, 18H), 1.63 – 1.39 (m, 28H), 1.14 – 1.01 (m, 16H).
^{31}P NMR (162 MHz, Benzene-d_6) δ 26.07.
^{13}C NMR (101 MHz, Benzene-d_6) δ 148.2, 137.3, 125.4, 123.3, 120.0, 115.7, 34.8, 33.0 (t, J = 7.3 Hz), 32.8, 30.4, 29.2, 27.3 (t, J = 6.1 Hz), 27.2 (t, J = 4.0 Hz), 26.7, 26.0 (t, J = 8.7 Hz).

4. General procedures

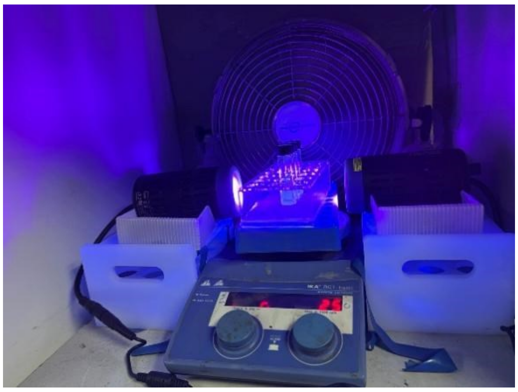

Supplementary Figure 2. Reaction set-up.
(The fan is used to keep the reaction temperature being around ambient temperature ~ 27 ^{o}C)

4.1 General procedures of defluorinative C-N coupling of carbazoles

General procedure A

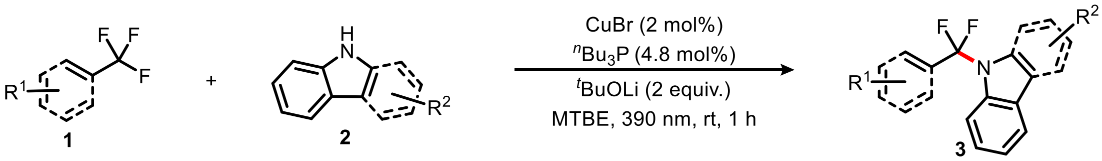

In a N_2-filled glove-box, an oven-dried vial (8 mL) equipped with a magnetic stir bar is charged with CuBr (0.29 mg, 0.002 mmol, 2 mol%), carbazole **2** (0.1 mmol, 1 equiv.), ^{t}BuOLi (16.0 mg, 0.2 mmol, 2 equiv.), MTBE (4 mL) and ^{n}Bu$_3$P (1.2 μL, 0.0048 mmol, 4.8 mol%). The reaction mixture is allowed to stir at rt for 30 min. Subsequently, the trifluoromethylated arene **1** (0.5 mmol, 5 equiv.) is added. Then the vial is sealed with a rubber cap, removed from the glove-box and is irradiated under 390 nm LED (40 W × 2) for 1 h at rt with vigorous stirring. When the reaction is completed, the mixture is then concentrated in vacuo. The crude product can be purified by flash column chromatography on neutral Al_2O_3 with petroleum ether, ethyl acetate and triethylamine as eluent to afford the desired coupling products.

General procedure B

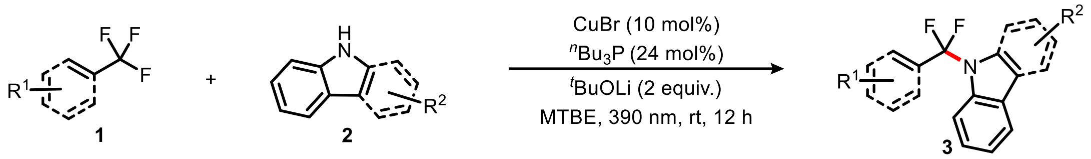

In a N_2-filled glove-box, an oven-dried vial (8 mL) equipped with a magnetic stir bar is charged with CuBr (1.43 mg, 0.01 mmol, 10 mol%), carbazole **2** (0.1 mmol, 1 equiv.), ^{t}BuOLi (16.0 mg, 0.2 mmol, 2 equiv.), MTBE (4 mL) and ^{n}Bu$_3$P (6.0 µL, 0.024 mmol, 24 mol%). The reaction mixture is allowed to stir at rt for 30 min. Subsequently, the trifluoromethylated arene **1** (0.5 mmol, 5 equiv.) is added. Then the vial is sealed with a rubber cap, removed from the glove-box and is irradiated under 390 nm LED (40 W × 2) for 12 h at rt with vigorous stirring. When the reaction is completed, the mixture is then concentrated in vacuo. The crude product can be purified by flash column chromatography on neutral Al_2O_3 with petroleum ether, ethyl acetate and triethylamine as eluent to afford the desired coupling products.

General procedure C

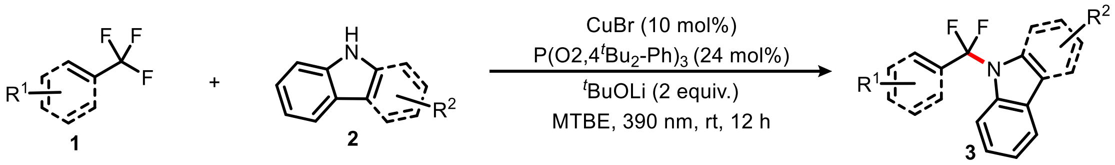

In a N_2-filled glove-box, an oven-dried vial (8 mL) equipped with a magnetic stir bar is charged with CuBr (1.43 mg, 0.01 mmol, 10 mol%), carbazole **2** (0.1 mmol, 1 equiv.), ^{t}BuOLi (16.0 mg, 0.2 mmol, 2 equiv.), tris(2,4-di-tert-butylphenyl)phosphite (15.5 mg, 0.024 mmol, 24 mol%) and MTBE (4 mL). Then the reaction mixture is allowed to stir at rt for 30 min. Subsequently, the trifluoromethylated arene **1** (0.5 mmol, 5 equiv.) is added. Then the vial is sealed with a rubber cap, removed from the glove-box and is irradiated under 390 nm LED (40 W × 2) for 12 h at rt with vigorous stirring. When the reaction is completed, the mixture is then concentrated in vacuo. The crude product can be purified by flash column chromatography on neutral Al_2O_3 with petroleum ether, ethyl acetate and triethylamine as eluent to afford the desired coupling products.

4.2 General procedures of the tandem C–N coupling and defluorination

General procedure D

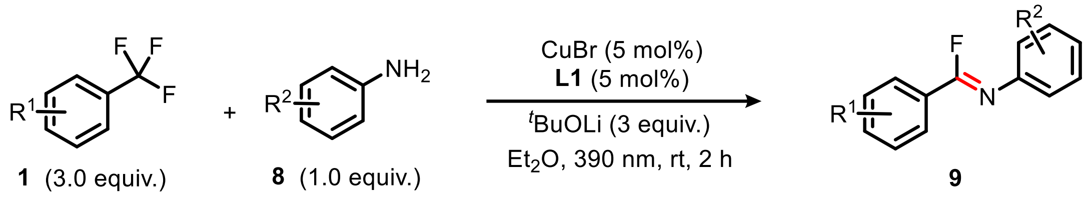

In a N_2-filled glove-box, an oven-dried vial (4 mL) equipped with a magnetic stir bar is charged with CuBr (1.43 mg, 0.01 mmol, 5 mol %), **L1** (7.0 mg, 0.01 mmol, 5 mol %), ^{t}BuOLi (48.0 mg, 0.6 mmol, 3 equiv.) and Et_2O (2 mL). Then the reaction mixture is allowed to stir at rt for 30 min. Subsequently, the trifluoromethylated arene **1** (0.6 mmol, 3 equiv.), aromatic amine **8** (0.2 mmol, 1 equiv.) are added. Then the vial is sealed with a rubber cap, removed from the glove-box and is irradiated under 390 nm LED (40 W × 2) for 2 h at rt with vigorous stirring. When the reaction is completed, the mixture is then concentrated in vacuo. The crude product can be purified by flash column chromatography on neutral Al_2O_3 with petroleum ether, and ethyl acetate as eluent to afford the desired coupling products.

4.3 General procedure for the 1,2-difluoroalkylamination reaction

General procedure E

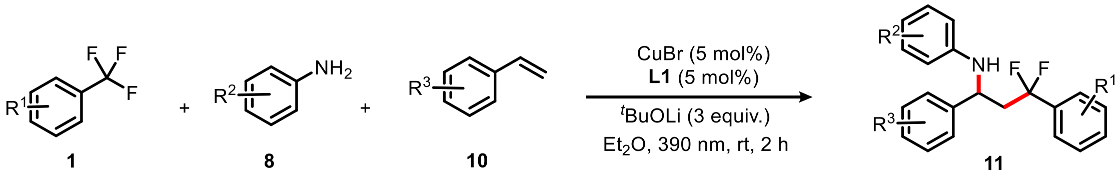

In a N_2-filled glove-box, an oven-dried vial (4 mL) equipped with a magnetic stir bar is charged with CuBr (1.43 mg, 0.01 mmol, 5 mol %), **L1** (7.0 mg, 0.01 mmol, 5 mol %), ^{t}BuOLi (48.0 mg, 0.6 mmol, 3 equiv.) and Et_2O (2 mL). Then the reaction mixture is allowed to stir at rt for 30 min. Subsequently, the trifluoromethylated arene **1** (0.6 mmol, 3 equiv.), styrene **10** (0.6 mmol, 3 equiv.), aromatic amine **8** (0.2 mmol, 1 equiv.) are added. Then the vial is sealed with a rubber cap, removed from the glove-box and is irradiated under 390 nm LED (40 W × 2) for 2 h at rt with vigorous stirring. When the reaction is completed, the mixture is then concentrated in vacuo. The crude

product can be purified by flash column chromatography on neutral Al_2O_3 with petroleum ether, and ethyl acetate as eluent to afford the desired coupling products.

5. Characterization of products

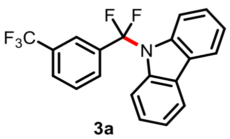

3a

9-(difluoro(3-(trifluoromethyl)phenyl)methyl)-9H-carbazole (3a)

According to the general procedure A, the crude product is purified by flash chromatography on Al_2O_3 (PE : NEt_3=100:1). (0.1 mmol scale, 26.0 mg, 72%). Rf= 0.7 (PE/EA = 20:1). Light yellow oil.

IR: ν = 3065, 2926, 2855, 1599, 1480, 1316, 1128, 747, 722, 698 cm^{-1}.
^{1}H NMR (500 MHz, Chloroform-*d*) δ 8.10 (d, *J* = 6.8 Hz, 2H), 8.00 (s, 1H), 7.79 (d, *J* = 7.7 Hz, 1H), 7.55 (d, *J* = 8.0 Hz, 1H), 7.50 (t, *J* = 7.8 Hz, 1H), 7.45 – 7.38 (m, 4H), 7.38 – 7.33 (m, 2H).
^{19}F NMR (471 MHz, Chloroform-*d*) δ -62.75 (s, 3F), -68.43 (s, 2F).
^{13}C NMR (126 MHz, Chloroform-d) δ 138.5, 135.6 (t, *J* = 33.8 Hz), 131.7 (q, *J* = 33.2 Hz), 129.7, 129.5 (t, *J* = 3.7 Hz), 128.3 – 128.1 (m), 126.8, 125.0, 123.5 (q, *J* = 272.2 Hz),123.2 (q, *J* = 3.9 Hz), 121.8, 120.2 (t, *J* = 252.0 Hz),120.2, 113.1 (t, *J* = 5.6 Hz).
HRMS (FI) calcd. for $C_{20}H_{12}NF_5$ $[M]^+$: 361.0884, found: 361.0889.

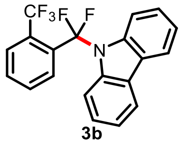

3b

9-(difluoro(2-(trifluoromethyl)phenyl)methyl)-9H-carbazole (3b)

According to the general procedure A, the crude product is purified by flash chromatography on Al_2O_3 (PE : NEt_3=100:1). (0.1 mmol scale, 27.9 mg, 77%). Rf= 0.7 (PE/EA = 20:1). Off-white solid.

m.p.: 78.0-80.5 °C.
IR: ν = 2923, 1447, 1305, 1280, 1157, 1137, 1038, 1023, 743, 670 cm^{-1}.
^{1}H NMR (500 MHz, Chloroform-*d*) δ 8.11 – 8.06 (m, 2H), 7.97 (d, *J* = 8.0 Hz, 1H), 7.66 (t, *J* = 7.8 Hz, 1H), 7.48 (t, *J* = 7.8 Hz, 1H), 7.38 – 7.30 (m, 3H), 7.29 – 7.25 (m, 2H), 7.17 (d, *J* = 8.0 Hz, 1H).
^{19}F NMR (471 MHz, Chloroform-*d*) δ -58.61 (t, *J* = 18.0 Hz, 3F), -64.11 (q, *J* = 18.0 Hz, 2F).
^{13}C NMR (126 MHz, Chloroform-*d*) δ 138.6, 132.4, 131.8, 129.2 (t, *J* = 6.1 Hz), 128.4 (q, *J* = 6.3 Hz), 126.7, 124.7, 123.2 (q, *J* = 274.7 Hz), 121.6, 120.1, 120.1 (t, *J* = 254.3 Hz), 113.1 (t, *J* = 5.3 Hz).
HRMS (FI) calcd. for $C_{20}H_{12}NF_5$ $[M]^+$: 361.0884, found: 361.0881.

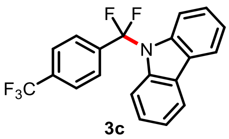

3c

9-(difluoro(4-(trifluoromethyl)phenyl)methyl)-9H-carbazole (3c)

According to the general procedure A, the crude product is purified by flash chromatography on Al_2O_3 (PE : NEt_3=100:1). (0.1 mmol scale, 31.4 mg, 87%). Rf= 0.7 (PE/EA = 20:1). Light yellow solid.

m.p.: 64.1-66.0 °C.
IR: ν = 3065, 1449, 1319, 1306, 1148, 1097, 1067, 832, 752, 635 cm^{-1}.
^{1}H NMR (500 MHz, Chloroform-*d*) δ 8.13 – 8.09 (m, 2H), 7.69 (d, *J* = 8.3 Hz, 2H), 7.63 (d, *J* = 8.3 Hz, 2H), 7.49 – 7.45 (m, 2H), 7.44 – 7.39 (m, 2H), 7.39 – 7.33 (m, 2H).
^{19}F NMR (471 MHz, Chloroform-*d*) δ -63.00 (s, 3F), -68.85 (s, 2F).
^{13}C NMR (126 MHz, Chloroform-*d*) δ 138.5, 138.0 (t, *J* = 33.8 Hz), 133.4 (q, *J* = 32.5 Hz), 126.8, 126.6 (t, *J* = 3.9 Hz), 126.0 (q, *J* = 3.9 Hz), 125.0, 123.4 (q, *J* = 273.4 Hz), 121.8, 120.3 (t, *J* = 251.7 Hz), 120.2, 113.1 (t, *J* = 5.7 Hz).
HRMS (FI) calcd. for $C_{20}H_{12}NF_5$ $[M]^+$: 361.0884, found: 361.0887.

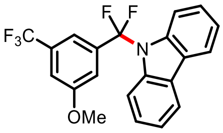

3d

9-(difluoro(3-methoxy-5-(trifluoromethyl)phenyl)methyl)-9H-carbazole (3d)

According to the general procedure A, the crude product is purified by flash chromatography on Al_2O_3 (PE : NEt_3=100:1). (0.1 mmol scale, 25.0 mg, 64%). Rf= 0.65 (PE/EA = 20:1). Off-white solid.

m.p.: 54.0-55.5 °C.
IR: ν = 2941, 2851, 1449, 1365, 1251, 1215, 1038, 753, 697, 408 cm^{-1}.
^{1}H NMR (500 MHz, Chloroform-*d*) δ 8.11 – 8.07 (m, 2H), 7.53 (s, 1H), 7.45 – 7.38 (m, 4H), 7.37 – 7.33 (m, 2H), 7.28 (s, 1H), 7.14 (s, 1H), 3.75 (s, 3H).
^{19}F NMR (471 MHz, Chloroform-*d*) δ -62.81 (s, 3F), -68.72 (s, 2F).
^{13}C NMR (126 MHz, Chloroform-*d*) δ 160.3, 138.5, 137.0 (t, *J* = 33.8 Hz), 132.9 (q, *J* = 33.2 Hz), 126.7, 125.0, 123.3 (q, *J* = 273.4 Hz), 121.8, 120.1, 120.1 (t, *J* = 251.7 Hz), 115.4 (t, *J* = 4.3 Hz), 115.2 – 114.9 (m), 113.7 – 113.4 (m), 113.1 (t, *J* = 5.5 Hz), 55.7.
HRMS (FI) calcd. for $C_{21}H_{14}ONF_5$ $[M]^+$: 391.0990, found: 391.0997.

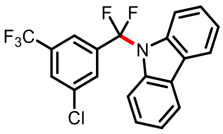

3e

9-((3-chloro-5-(trifluoromethyl)phenyl)difluoromethyl)-9H-carbazole (3e)

According to the general procedure A, the crude product is purified by flash chromatography on Al_2O_3 (PE : NEt_3=100:1). (0.1 mmol scale, 28.0 mg, 71%). Rf= 0.7 (PE/EA = 20:1). Off-white solid.

m.p.: 86.0-88.0 °C.
IR: ν = 3081, 1445, 1348, 1317, 1248, 1223, 1132, 905, 778, 420 cm^{-1}.
^{1}H NMR (500 MHz, Chloroform-*d*) δ 8.12 – 8.06 (m, 2H), 7.77 (s, 1H), 7.63 (s, 1H), 7.44 – 7.40 (m, 4H), 7.38 – 7.34 (m, 2H).
^{19}F NMR (471 MHz, Chloroform-*d*) δ -62.95 (s, 3F), -68.93 (s, 2F).
^{13}C NMR (126 MHz, Chloroform-*d*) δ 138.3, 137.5 (t, *J* = 34.8 Hz), 136.1, 133.2 (q, *J* = 33.8 Hz), 129.7 (t, *J* = 4.2 Hz), 128.6 (m), 126.9, 125.1, 122.6 (q, *J* = 273.2 Hz), 122.0, 121.4 – 121.2 (m), 120.3, 119.5 (t, *J* = 252.4 Hz), 113.0 (t, *J* = 5.7 Hz).
HRMS (FI) calcd. for $C_{20}H_{11}NClF_5$ $[M]^+$: 395.0495, found: 395.0501.

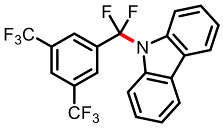

3f

9-((3,5-bis(trifluoromethyl)phenyl)difluoromethyl)-9H-carbazole (3f)

According to the general procedure A, the crude product is purified by flash chromatography on Al_2O_3 (PE : NEt_3=100:1). (0.1 mmol scale, 30.0 mg, 70%). Rf= 0.7 (PE/EA = 20:1). Light yellow solid.

m.p.: 84.0-85.8 °C.
IR: ν = 2926, 1447, 1279, 1170, 1128, 1099, 894, 748, 680, 417 cm^{-1}.
^{1}H NMR (500 MHz, Chloroform-*d*) δ 8.11 (d, *J* = 7.6 Hz, 2H), 8.07 (s, 1H), 8.01 (s, 2H), 7.45 – 7.35 (m, 6H).
^{19}F NMR (471 MHz, Chloroform-*d*) δ -63.06 (s, 6F), -68.98 (s, 2F).
^{13}C NMR (126 MHz, Chloroform-*d*) δ 138.3, 137.2 (t, *J* = 35.3 Hz), 132.9 (q, *J* = 34.2 Hz), 127.0, 126.6 – 126.3 (m), 125.5 – 125.3 (m), 125.3, 122.6 (q, *J* = 273.4 Hz), 122.2, 120.4, 119.5 (t, *J* = 252.0 Hz), 112.9 (t, *J* = 5.7 Hz).
HRMS (FI) calcd. for $C_{21}H_{11}NF_8$ $[M]^+$: 429.0758, found: 429.0763.

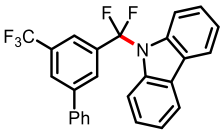

3g

9-(difluoro(5-(trifluoromethyl)-[1,1'-biphenyl]-3-yl)methyl)-9H-carbazole (3g)

According to the general procedure A, the crude product is purified by flash chromatography on Al_2O_3 (PE : NEt_3=100:1). (0.1 mmol scale, 27.6 mg, 63%). Rf= 0.7 (PE/EA = 20:1). Off-white solid.

m.p.: 78.5-80.0 °C.
IR: ν = 3059, 2926, 1494, 1454, 1317, 1228, 1162, 1127, 745, 420 cm^{-1}.
^{1}H NMR (500 MHz, Chloroform-*d*) δ 8.15 – 8.09 (m, 2H), 8.04 (s, 1H), 7.91 (s, 2H), 7.51 – 7.40 (m, 9H), 7.39 – 7.35 (m, 2H).
^{19}F NMR (471 MHz, Chloroform-*d*) δ -62.60 (s, 3F), -68.41 (s, 2F).
^{13}C NMR (126 MHz, Chloroform-*d*) δ 143.3, 138.6, 138.3, 136.2 (t, *J* = 33.5 Hz), 132.2 (q, *J* = 33.0 Hz), 129.1, 128.7, 128.0 (t, *J* = 4.3 Hz), 127.1, 126.8 (d, *J* = 3.7 Hz), 126.8, 125.1, 123.5 (q, *J* = 273.4 Hz), 121.8, 121.7 (m), 120.3 (t, *J* = 251.5 Hz), 120.2, 113.2(t, *J* = 5.5 Hz).
HRMS (FI) calcd. for $C_{26}H_{16}NF_5$ $[M]^+$: 437.1197, found: 437.1200.

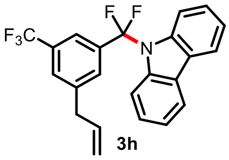

3h

9-((3-allyl-5-(trifluoromethyl)phenyl)difluoromethyl)-9H-carbazole (3h)

According to the general procedure A, the crude product is purified by flash chromatography on Al_2O_3 (PE : NEt_3=100:1). (0.1 mmol scale, 19.9 mg, 50%). Rf= 0.7 (PE/EA = 20:1). Light yellow oil.

IR: ν = 3066, 1447, 1317, 1237, 1162, 1126, 748, 722, 678, 418 cm^{-1}
^{1}H NMR (500 MHz, Chloroform-*d*) δ 8.11 – 8.06 (m, 2H), 7.76 (s, 1H), 7.63 (s, 1H), 7.50 (s, 1H), 7.39 – 7.32 (m, 6H), 5.86 (ddt, *J* = 17.0, 10.1, 6.6 Hz, 1H), 5.11 (dq, *J* = 10.1, 1.4 Hz, 1H), 5.04 (dq, *J* = 17.0, 1.4 Hz, 1H), 3.41 (d, *J* = 6.6 Hz, 2H).
^{19}F NMR (471 MHz, Chloroform-*d*) δ -62.65 (s, 3F), -68.46 (s, 2F).
^{13}C NMR (126 MHz, Chloroform-*d*) δ 142.5, 138.5, 135.7 (t, *J* = 33.3 Hz), 135.2, 131.8 (q, *J* = 32.9 Hz), 129.7 (t, *J* = 4.0 Hz), 128.6 – 128.2 (m), 126.7, 125.0, 123.5 (q, *J* = 273.4 Hz), 121.7, 121.2 – 120.9 (m), 120.2 (t, *J* = 251.4 Hz), 120.1, 117.6, 113.1 (t, *J* = 5.6 Hz), 39.5.
HRMS (FI) calcd. for $C_{23}H_{16}NF_5$ $[M]^+$: 401.1197, found: 401.1194.

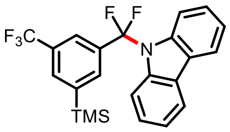

3i

9-(difluoro(3-(trifluoromethyl)-5-(trimethylsilyl)phenyl)methyl)-9H-carbazole (3i)

According to the general procedure A, the crude product is purified by flash chromatography on Al_2O_3 (PE : NEt_3=100:1). (0.1 mmol scale, 27.9 mg, 64%). Rf= 0.7 (PE/EA = 20:1). Light yellow oil.

IR: ν = 2957, 1447, 1317, 1299, 1233, 1127, 1105, 838, 747, 419 cm^{-1}.
^{1}H NMR (500 MHz, Chloroform-*d*) δ 8.11 – 8.08 (m, 2H), 7.91 (s, 1H), 7.84 (s, 1H), 7.78 (s, 1H), 7.42 – 7.33 (m, 6H), 0.24 (s, 9H).
^{19}F NMR (471 MHz, Chloroform-*d*) δ -62.59 (s, 3F), -68.26 (s, 2F).
^{13}C NMR (126 MHz, Chloroform-*d*) δ 143.9, 138.7, 134.6 (t, *J* = 33.2 Hz), 134.1 (t, *J* = 3.9 Hz), 132.8 – 132.4 (m), 130.8 (q, *J* = 32.5 Hz), 126.7, 125.1, 124.2 (q, *J* = 273.4 Hz), 123.6 – 123.2 (m), 121.8, 122.6 – 118.3 (m), 120.1, 113.3 (t, *J* = 5.5 Hz), -1.6.
HRMS (FI) calcd. for $C_{23}H_{20}NF_5Si$ $[M]^+$: 433.1280, found: 433.1282.

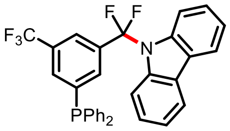

3j

9-((3-(diphenylphosphaneyl)-5-(trifluoromethyl)phenyl)difluoromethyl)-9H-carbazole (3j)

According to the general procedure A, the crude product is purified by flash chromatography on Al_2O_3 (PE : NEt_3=100:1). (0.1 mmol scale, 27.0 mg, 50%). Rf= 0.7 (PE/EA = 20:1). Off-white solid.

m.p.: 75.8-78.6 °C.
IR: ν = 3058, 1447, 1320, 1127, 1105, 1067, 740, 722, 695, 418 cm^{-1}.
^{1}H NMR (500 MHz, Chloroform-*d*) δ 8.09 – 8.04 (m, 2H), 7.97 (s, 1H), 7.77 (d, *J* = 7.1 Hz, 1H), 7.40 – 7.32 (m, 8H), 7.29 – 7.22 (m, 5H), 7.14 – 7.09 (m, 4H).
^{19}F NMR (471 MHz, Chloroform-*d*) δ -62.65 (s, 3F), -68.84 (s, 2F).
^{31}P NMR (202 MHz, Chloroform-*d*) δ -5.43.
^{13}C NMR (126 MHz, Chloroform-*d*) δ 141.8 (d, *J* = 18.2 Hz), 138.3, 135.5 (td, *J* = 34.1, 4.0 Hz), 134.8 (d, *J* = 10.4 Hz), 133.5 (d, *J* = 20.2 Hz), 133.0 – 132.6 (m), 131.7 (qd, *J* = 32.9, 7.5 Hz), 129.4, 128.8, 128.7, 126.7, 124.9, 123.3 (q, *J* = 273.4 Hz), 123.3 – 122.8 (m), 121.7, 120.2 (t, *J* = 252.0 Hz), 120.2, 113.0 (t, *J* = 5.6 Hz).
HRMS (FI) calcd. for $C_{32}H_{21}NF_5P$ $[M]^+$: 545.1326, found: 545.1330.

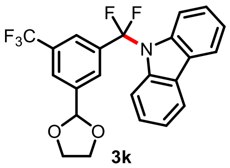

3k

9-((3-(1,3-dioxolan-2-yl)-5-(trifluoromethyl)phenyl)difluoromethyl)-9H-carbazole (3k)

According to the general procedure A, the crude product is purified by flash chromatography on Al_2O_3 (PE : NEt_3=100:1). (0.1 mmol scale, 25.7 mg, 59%). Rf= 0.4 (PE/EA = 20:1). Light yellow oil.

IR: ν = 2892, 1447, 1301, 1239, 1163, 1128, 748, 722, 677, 418 cm^{-1}.
^{1}H NMR (500 MHz, Chloroform-*d*) δ 8.11 – 8.07 (m, 2H), 7.96 (s, 1H), 7.88 (s, 1H), 7.82 (s, 1H), 7.43 – 7.37 (m, 4H), 7.36 – 7.32 (m, 2H), 5.81 (s, 1H), 4.05 – 3.96 (m, 4H).
^{19}F NMR (471 MHz, Chloroform-*d*) δ -62.68 (s, 3F), -68.46 (s, 2F).
^{13}C NMR (126 MHz, Chloroform-*d*) δ 140.9, 138.5, 135.8 (t, *J* = 33.9 Hz), 131.9 (q, *J* = 33.3 Hz), 127.9 – 127.6 (m), 126.7, 126.5 – 126.4 (m), 125.0, 123.9 – 123.6 (m), 123.3 (q, *J* = 273.4 Hz), 121.8, 120.2, 120.1 (t, *J* = 251.6 Hz), 113.1 (t, *J* = 5.6 Hz), 101.9, 65.3.
HRMS (FI) calcd. for $C_{23}H_{16}O_2NF_5$ $[M]^+$: 433.1096, found: 433.1102.

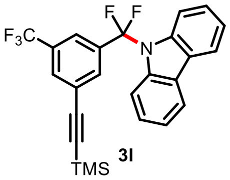

3l

9-(difluoro(3-(trifluoromethyl)-5-((trimethylsilyl)ethynyl)phenyl)methyl)-9H-carbazole (3l)

According to the general procedure A, the crude product is purified by flash chromatography on Al_2O_3 (PE : NEt_3=100:1). (0.1 mmol scale, 19.6 mg, 43%). Rf= 0.7 (PE/EA = 20:1). Light yellow oil.

IR: ν = 3066, 2961, 2168, 1447, 1240, 1133, 1109, 840, 746, 418 cm^{-1}.
^{1}H NMR (500 MHz, Chloroform-*d*) δ 8.12 – 8.07 (m, 2H), 7.90 (s, 1H), 7.80 (s, 2H), 7.42 – 7.33 (m, 6H), 0.26 (s, 9H).
^{19}F NMR (471 MHz, Chloroform-*d*) δ -63.00 (s, 3F), -68.99 (s, 2F).
^{13}C NMR (126 MHz, Chloroform-*d*) δ 138.4, 136.1 (t, *J* = 33.8 Hz), 132.5 (t, *J* = 4.0 Hz), 132.0 (q, *J* = 33.5 Hz), 131.7 – 131.6 (m), 126.8, 125.6, 125.1, 123.0 (q, *J* = 273.4 Hz), 122.8 – 122.5 (m), 121.8, 120.2, 119.8 (t, *J* = 252.0 Hz), 113.1 (t, *J* = 5.6 Hz), 101.8, 98.5, -0.4.
HRMS (FI) calcd. for $C_{25}H_{20}NF_5Si$ $[M]^+$: 457.1280, found: 457.1283.

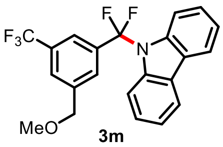

9-(difluoro(3-(methoxymethyl)-5-(trifluoromethyl)phenyl)methyl)-9H-carbazole (3m)

According to the general procedure A, the crude product is purified by flash chromatography on Al_2O_3 (PE : NEt_3=100:1). (0.1 mmol scale, 23.4 mg, 58%). Rf= 0.6 (PE/EA = 20:1). Light yellow solid.

m.p.: 72.0-73.4 °C.
IR: ν = 2929, 2861, 2827, 1447, 1148, 1121, 1099, 874, 746, 419 cm^{-1}.
^{1}H NMR (500 MHz, Chloroform-*d*) δ 8.11 – 8.06 (m, 2H), 7.84 (s, 1H), 7.81 (s, 1H), 7.62 (s, 1H), 7.41 – 7.32 (m, 6H), 4.45 (s, 2H), 3.35 (s, 3H).
^{19}F NMR (471 MHz, Chloroform-*d*) δ -62.69 (s, 3F), -68.51 (s, 2F).
^{13}C NMR (126 MHz, Chloroform-*d*) δ 141.1, 138.5, 135.7 (t, *J* = 33.5 Hz), 131.8 (q, *J* = 33.1 Hz), 128.0 (t, *J* = 4.1 Hz), 127.1 – 126.8 (m), 126.7, 125.0, 123.4 (q, *J* = 273.4 Hz), 122.3-122.1 (m), 121.8, 120.2 (t, *J* = 252.0 Hz), 120.1, 113.1 (t, *J* = 5.6 Hz), 73.0, 58.5.
HRMS (FI) calcd. for $C_{22}H_{16}ONF_5$ $[M]^+$: 405.1147, found: 405.1143.

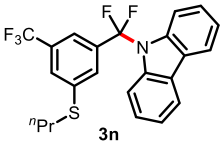

9-(difluoro(3-(propylthio)-5-(trifluoromethyl)phenyl)methyl)-9H-carbazole (3n)

According to the general procedure B, the crude product is purified by flash chromatography on Al_2O_3 (PE : NEt_3=100:1). (0.1 mmol scale, 32.9 mg, 76%). Rf= 0.7 (PE/EA = 20:1). Light yellow oil.

IR: ν = 2965, 1447, 1231, 1165, 1127, 1106, 747, 722, 697, 677 cm^{-1}.
^{1}H NMR (500 MHz, Chloroform-*d*) δ 8.09 (s, 1H), 8.08 (s, 1H), 7.67 (s, 1H), 7.63 (s, 1H), 7.45 – 7.37 (m, 4H), 7.37 – 7.32 (m, 3H), 2.77 – 2.69 (m, 2H), 1.49 (h, *J* = 7.3 Hz, 2H), 0.91 (t, *J* = 7.3 Hz, 3H).
^{19}F NMR (471 MHz, Chloroform-*d*) δ -62.91 (s, 3F), -68.69 (s, 2F).
^{13}C NMR (126 MHz, Chloroform-*d*) δ 141.1, 138.5, 136.1 (t, *J* = 33.9 Hz), 132.1 (q, *J* = 33.1 Hz), 127.9 – 127.4 (m), 127.2 – 126.9 (m), 126.8, 125.0, 123.2 (q, *J* = 273.4 Hz), 121.9, 120.2, 120.1 (t, *J* = 251.7 Hz), 119.7 – 119.4 (m), 113.1 (t, *J* = 5.7 Hz), 34.5, 21.9, 13.2.
HRMS (FI) calcd. for $C_{23}H_{18}NF_5S$ $[M]^+$: 435.1075, found: 435.1072.

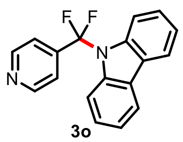

3o

9-(difluoro(pyridin-4-yl)methyl)-9H-carbazole (3o)

According to the general procedure B, the crude product is purified by flash chromatography on Al_2O_3 (PE : EA:NEt_3=100:5:1). (0.1 mmol scale, 18.5 mg, 63%). Rf= 0.35 (PE/EA = 5:1). Light yellow oil.

IR: ν = 3063, 1596, 1443, 1327, 1139, 1041, 816, 745, 661, 416 cm^{-1}.
^{1}H NMR (500 MHz, Chloroform-*d*) δ 8.70 (d, *J* = 6.1 Hz, 2H), 8.07 (d, *J* = 6.9 Hz, 2H), 7.49 – 7.44 (m, 2H), 7.42 – 7.31 (m, 6H).
^{19}F NMR (471 MHz, $CDCl_3$) δ -71.54 (s, 2F).
^{13}C NMR (126 MHz, Chloroform-*d*) δ 150.9, 142.3 (t, *J* = 35.3 Hz), 138.3, 126.8, 125.0, 121.9, 120.2, 120.0 (t, *J* = 3.7 Hz), 119.7 (t, *J* = 252.2 Hz), 113.0 (t, *J* = 5.8 Hz).
HRMS (FI) calcd. for $C_{18}H_{12}N_2F_2$ $[M]^+$: 294.0963, found: 294.0970.

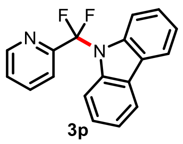

3p

9-(difluoro(pyridin-2-yl)methyl)-9H-carbazole (3p)

According to the general procedure B, the crude product is purified by flash chromatography on Al_2O_3 (PE : EA:NEt_3=100:5:1). (0.1 mmol scale, 10.4 mg, 35%). Rf= 0.35 (PE/EA = 5:1). Light yellow oil.

IR: ν = 3061, 2925, 1447, 1329, 1317, 1163, 1037, 744, 720, 418 cm^{-1}.
^{1}H NMR (500 MHz, Chloroform-*d*) δ 8.63 (d, *J* = 3.8 Hz, 1H), 8.05 (d, *J* = 8.3 Hz, 2H), 7.78 (td, *J* = 7.8, 1.7 Hz, 1H), 7.58 (dd, *J* = 7.9, 1.0 Hz, 1H), 7.47 (dt, *J* = 8.3, 1.0 Hz, 2H), 7.40 – 7.34 (m, 3H), 7.30 (td, *J* = 7.5, 1.0 Hz, 2H).
^{19}F NMR (471 MHz, $CDCl_3$) δ -75.13 (s, 2F).
^{13}C NMR (126 MHz, Chloroform-*d*) δ 152.0 (t, *J* = 35.3 Hz), 150.0, 138.7, 137.3, 126.5, 125.8 (t, *J* = 1.7 Hz), 124.9, 121.4, 121.0 (t, *J* = 2.9 Hz), 120.0, 118.0 (t, *J* = 252.7 Hz), 113.1 (t, *J* = 5.8 Hz).
HRMS (FI) calcd. for $C_{18}H_{12}N_2F_2$ $[M]^+$: 294.0963, found: 294.0967.

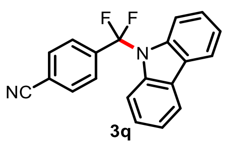

3q

4-((9H-carbazol-9-yl)difluoromethyl)benzonitrile (3q)

According to the general procedure C, the crude product is purified by flash chromatography on Al_2O_3 (PE : EA:NEt_3=100:5:1). (0.1 mmol scale, 16.5 mg, 52%). Rf= 0.4 (PE/EA = 10:1). Light yellow solid.

m.p.: 95.8-97.5 °C.
IR: ν = 3064, 2922, 2233, 1479, 1448, 1328, 1163, 1040, 749, 723 cm^{-1}.
^{1}H NMR (500 MHz, Chloroform-*d*) δ 8.12 – 8.04 (m, 2H), 7.69 (d, *J* = 8.3 Hz, 2H), 7.59 (d, *J* = 8.3 Hz, 2H), 7.46 – 7.31 (m, 6H).
^{19}F NMR (471 MHz, Chloroform-*d*) δ -69.48 (s, 2F).
^{13}C NMR (126 MHz, Chloroform-*d*) δ 138.7 (t, *J* = 34.3 Hz), 138.3, 132.8, 126.8, 126.8 (t, *J* = 3.9 Hz), 125.0, 121.9, 120.2, 120.0 (t, *J* = 252.0 Hz), 117.6, 115.4, 113.0 (t, *J* = 5.7 Hz).
HRMS (FI) calcd. for $C_{20}H_{12}N_2F_2$ [M]+: 318.0963, found: 318.0966.

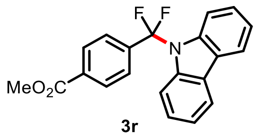

3r

methyl 4-((9H-carbazol-9-yl)difluoromethyl)benzoate (3r)

According to the general procedure C, the crude product is purified by flash chromatography on Al_2O_3 (PE : EA:NEt_3=100:5:1). (0.1 mmol scale, 14.0 mg, 40%). Rf= 0.45 (PE/EA = 10:1). Off-white solid.

m.p.: 74.8-77.5 °C.
IR: ν = 2954, 1721, 1287, 1224, 1164, 1010, 846, 773, 750, 418 cm^{-1}.
^{1}H NMR (500 MHz, Chloroform-*d*) δ 8.11 – 8.04 (m, 4H), 7.59 (d, *J* = 8.9 Hz, 2H), 7.41 – 7.38 (m, 2H), 7.37 – 7.34 (m, 2H), 7.32 (td, *J* = 7.3, 1.4 Hz, 2H), 3.93 (s, 3H).
^{19}F NMR (471 MHz, $CDCl_3$) δ -68.99 (s, 2F).
^{13}C NMR (126 MHz, Chloroform-*d*) δ 166.0, 138.5, 138.5 (t, *J* = 33.0 Hz), 132.9 (t, *J* = 2.1 Hz), 130.2, 126.7, 126.2 (t, *J* = 4.0 Hz), 124.9, 121.6, 120.5 (t, *J* = 251.4 Hz), 120.1, 52.4.
HRMS (FI) calcd. for $C_{21}H_{15}O_2NF_2$ $[M]^+$: 351.1065, found: 351.1060.

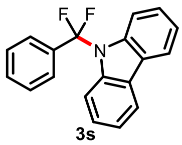

3s

9-(difluoro(phenyl)methyl)-9H-carbazole (3s)

According to the general procedure C, and $PhCF_3$(1.0 mmol, 10.0 equiv.) is added. the crude product is purified by flash chromatography on Al_2O_3 (PE : NEt_3=100:1). (0.2 mmol scale, 10.0 mg, 17%). Rf= 0.7 (PE/EA = 20:1). Light yellow oil.

IR: ν = 3064, 2925, 1598, 1491, 1446, 1327, 1221, 1018, 746, 418 cm^{-1}.
^{1}H NMR (500 MHz, Chloroform-*d*) δ 8.07 (d, *J* = 7.0 Hz, 2H), 7.55 – 7.49 (m, 3H), 7.43 (t, *J* = 7.7 Hz, 2H), 7.39 – 7.28 (m, 6H).
^{19}F NMR (471 MHz, $CDCl_3$) δ -68.05 (s, 2F).
^{13}C NMR (126 MHz, Chloroform-*d*) δ 138.8, 134.4 (t, *J* = 32.4 Hz), 131.4 (t, *J* = 2.1 Hz), 128.9, 126.5, 126.1 (t, *J* = 4.1 Hz), 124.8, 121.4, 121.0 (t, *J* = 250.6 Hz), 120.0, 113.3 (t, *J* = 5.5 Hz).
HRMS (FI) calcd. for $C_{19}H_{13}NF_2$ $[M]^+$: 293.1011, found: 293.1008.

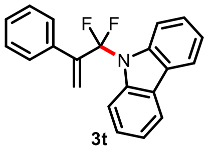

3t

9-(1,1-difluoro-2-phenylallyl)-9H-carbazole (3t)

According to the general procedure B, the crude product is purified by flash chromatography on Al_2O_3 (PE : NEt_3=100:1). (0.1 mmol scale, 10.5 mg, 33%). Rf= 0.7 (PE/EA = 20:1). Light yellow oil.

IR: ν = 2924, 1675, 1443, 1361, 1331, 1164, 749, 722, 697, 526 cm^{-1}.
^{1}H NMR (500 MHz, Chloroform-*d*) δ 8.04 (d, *J* = 7.8 Hz, 2H), 7.68 (d, *J* = 8.4 Hz, 2H), 7.44 – 7.37 (m, 4H), 7.33 – 7.26 (m, 5H), 5.66 (s, 1H), 5.44 (t, *J* = 2.3 Hz, 1H).
^{19}F NMR (471 MHz, $CDCl_3$) δ -71.48 (s, 2F)
^{13}C NMR (126 MHz, Chloroform-*d*) δ 142.3 (t, *J* = 31.8 Hz), 138.7, 134.9, 128.6, 128.3, 127.9, 126.5, 124.6, 121.4 (t, *J* = 6.4 Hz), 121.3, 120.8 (t, *J* = 252.5 Hz), 120.0, 113.2 (t, *J* = 5.9 Hz).
HRMS (FI) calcd. for $C_{21}H_{15}NF_2$ $[M]^+$: 319.1167, found: 319.1169.

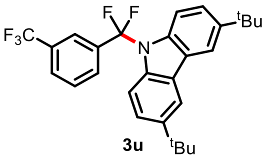

3u

3,6-di-tert-butyl-9-(difluoro(3-(trifluoromethyl)phenyl)methyl)-9H-carbazole (3u)

According to the general procedure A, the crude product is purified by flash chromatography on Al_2O_3 (PE : NEt_3=100:1). (0.1 mmol scale, 35.2 mg, 74%). Rf= 0.7 (PE/EA = 20:1). Light yellow solid.

m.p.: 113.9-116.4 °C.
IR: ν = 2962, 2869, 1618, 1471, 1316, 1231, 1172, 1126, 803, 699 cm^{-1}.
^{1}H NMR (500 MHz, Chloroform-*d*) δ 8.11 (d, *J* = 2.0 Hz, 2H), 8.04 (s, 1H), 7.81 (d, *J* = 7.8 Hz, 1H), 7.62 (d, *J* = 7.9 Hz, 1H), 7.54 (t, *J* = 7.8 Hz, 1H), 7.44 (d, *J* = 2.0 Hz, 1H), 7.43 (d, *J* = 2.1 Hz, 1H), 7.27 (d, *J* = 1.7 Hz, 1H), 7.26 (d, *J* = 1.7 Hz, 1H), 1.48 (s, 18H).
^{19}F NMR (471 MHz, $CDCl_3$) δ -62.69 (s, 3F), -68.63(s, 2F).
^{13}C NMR (126 MHz, Chloroform-*d*) δ 144.7, 137.0, 135.8 (t, *J* = 33.6 Hz), 131.6 (q, *J* = 33.2 Hz), 129.7 (t, *J* = 5.1Hz) 129.7, 128.2 – 128.1(m), 125.1, 124.4, 123.5 (q, *J* =273.4 Hz), 123.3 (q, *J* = 3.9 Hz), 120.2 (t, *J* = 250.6 Hz), 116.1, 112.6(t, *J* = 5.2 Hz), 34.7, 31.8.
HRMS (FI) calcd. for $C_{28}H_{28}NF_5$ $[M]^+$: 473.2136, found: 473.2143.

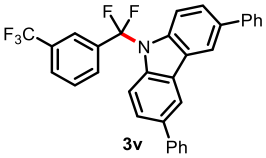

3v

9-(difluoro(3-(trifluoromethyl)phenyl)methyl)-3,6-diphenyl-9H-carbazole (3v)

According to the general procedure A, the crude product is purified by flash chromatography on Al_2O_3 (PE : NEt_3=100:1). (0.1 mmol scale, 32.5 mg, 63%). Rf= 0.7 (PE/EA = 20:1). Light yellow solid.

m.p.: 115.6-117.3 °C.
IR: ν = 3061, 3030, 1600, 1475, 1312, 1126, 1075, 809, 758, 694 cm^{-1}.
^{1}H NMR (500 MHz, Chloroform-*d*) δ 8.35 (d, *J* = 1.9 Hz, 2H), 8.05 (s, 1H), 7.82 (d, *J* = 7.7 Hz, 1H), 7.73 (d, *J* = 1.5 Hz, 2H), 7.72 – 7.71 (m, 2H), 7.65 (d, *J* = 1.9 Hz, 1H), 7.63 (d, *J* = 1.9 Hz, 1H), 7.60 (d, *J* = 8.0 Hz, 1H), 7.55 (d, *J* = 7.9 Hz, 1H), 7.52 – 7.48 (m, 4H), 7.45 (dt, *J* = 8.7, 1.8 Hz, 2H), 7.41 – 7.38 (m, 2H).
^{19}F NMR (471 MHz, $CDCl_3$) δ -62.68 (s, 3F), -68.53(s, 2F).
^{13}C NMR (126 MHz, Chloroform-*d*) δ 141.1, 138.3, 135.4 (t, *J* = 33.6 Hz), 135.3, 131.8 (q, *J* = 33.0 Hz), 129.8, 129.5 (t, *J* = 4.1 Hz), 128.8, 128.4 – 128.2 (m), 127.3, 127.0, 126.3, 125.6, 123.4 (q, *J* = 273.4 Hz), 123.2 (q, *J* = 3.8 Hz), 120.2 (t, *J* = 251.7 Hz), 118.6, 113.4 (t, *J* = 5.4 Hz).
HRMS (FI) calcd. for $C_{32}H_{20}NF_5$ $[M]^+$: 513.1510, found: 513.1515.

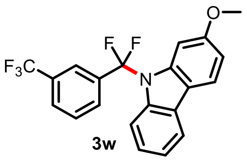

9-(difluoro(3-(trifluoromethyl)phenyl)methyl)-2-methoxy-9H-carbazole (3w)

According to the general procedure A, the crude product is purified by flash chromatography on Al_2O_3 (PE : NEt_3=100:1). (0.1 mmol scale, 21.4 mg, 55%). Rf= 0.6 (PE/EA = 20:1). Off-white solid.

m.p.: 110.0-111.2 °C.
IR: ν = 3077, 2835, 1670, 1368, 1271, 1163, 1122, 1066, 762, 718 cm^{-1}.
^{1}H NMR (500 MHz, Chloroform-*d*) δ 8.01 – 7.95 (m, 2H), 7.94 (d, *J* = 8.6 Hz, 1H), 7.81 – 7.76 (m, 1H), 7.57 – 7.49 (m, 2H), 7.39 – 7.35 (m, 1H), 7.33 – 7.28 (m, 2H), 6.94 (dd, *J* = 8.6, 2.2 Hz, 1H), 6.88 – 6.85 (m, 1H), 3.79 (s, 3H).
^{19}F NMR (471 MHz, Chloroform-*d*) δ -62.77 (s, 3F), -68.61 (s, 2F).
^{13}C NMR (126 MHz, Chloroform-*d*) δ 159.3, 139.7, 138.5, 135.5 (t, *J* = 33.8 Hz), 131.7 (q, *J* = 33.2 Hz), 129.7, 129.7 – 129.4 (m), 128.4 – 128.0 (m), 125.4, 125.1, 123.4 (q, *J* = 273.4 Hz), 123.5 – 123.0 (m), 121.9, 120.8, 120.2 (t, *J* = 251.2 Hz), 119.3, 118.6, 113.0 (t, *J* = 5.6 Hz), 110.0, 97.9 (t, *J* = 5.8 Hz), 55.5.
HRMS (FI) calcd. for $C_{21}H_{14}ONF_5$ $[M]^+$: 391.0990, found: 391.0987.

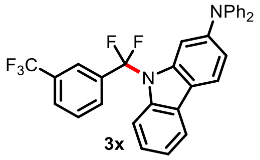

9-(difluoro(3-(trifluoromethyl)phenyl)methyl)-N,N-diphenyl-9H-carbazol-2-amine (3x)

According to the general procedure A, the crude product is purified by flash chromatography on Al_2O_3 (PE : NEt_3=100:1). (0.1 mmol scale, 28.7 mg, 54%). Rf= 0.6 (PE/EA = 20:1). Off-white solid.

m.p.: 98.9-100.4 °C.
IR: ν = 2923, 1591, 1487, 1462, 1167, 745, 694, 667, 516, 409 cm^{-1}.
^{1}H NMR (500 MHz, Chloroform-*d*) δ 8.02 – 7.97 (m, 1H), 7.90 (d, *J* = 8.4 Hz, 1H), 7.84 (s, 1H), 7.72 – 7.68 (m, 1H), 7.60 – 7.54 (m, 2H), 7.42 (t, *J* = 7.9 Hz, 1H), 7.38 (td, *J* = 7.9, 1.7 Hz, 1H), 7.34 (td, *J* = 7.4, 1.2 Hz, 1H), 7.26 – 7.21 (m, 4H), 7.10 (dd, *J* = 8.5, 1.9 Hz, 1H), 7.07 – 7.02 (m, 6H), 6.76 – 6.74 (m, 1H).
^{19}F NMR (471 MHz, Chloroform-*d*) δ -62.70 (s, 3F), -68.91 (s, 2F).
^{13}C NMR (126 MHz, Chloroform-*d*) δ 147.8, 147.1, 139.3, 139.0, 135.3 (t, *J* = 33.2 Hz), 131.5 (q, *J* = 33.1 Hz), 129.6, 129.4 – 129.2 (m), 129.2, 128.2 – 127.8 (m), 125.9, 125.0, 124.2, 123.4 (q, *J* = 273.4 Hz), 123.2 – 123.0 (m), 122.8, 122.0, 120.5, 120.3, 120.0 (t, *J* = 250.7 Hz), 119.5, 118.8, 113.3 (t, *J* = 6.6 Hz), 108.6 (t, *J* = 4.3 Hz).
HRMS (FI) calcd. for $C_{32}H_{21}N_2F_5$ $[M]^+$: 528.1619, found: 528.1624.

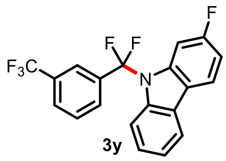

3y

9-(difluoro(3-(trifluoromethyl)phenyl)methyl)-2-fluoro-9H-carbazole (3y)

According to the general procedure A, the crude product is purified by flash chromatography on Al_2O_3 (PE : NEt_3=100:1). (0.1 mmol scale, 18.8 mg, 50%). Rf= 0.7 (PE/EA = 20:1). Light yellow solid.

m.p.: 88.5-90.2 ºC.
IR: ν = 3051, 2927, 1554, 1315, 1251, 1162, 1129, 744, 697, 422 cm^{-1}.
^{1}H NMR (500 MHz, Chloroform-*d*) δ 8.04 – 7.97 (m, 2H), 7.93 (s, 1H), 7.83 – 7.77 (m, 1H), 7.56 – 7.51 (m, 2H), 7.37 – 7.29 (m, 3H), 7.14 (dq, *J* = 10.4, 1.8 Hz, 1H), 7.08 (td, *J* = 8.8, 2.3 Hz, 1H).
^{19}F NMR (471 MHz, Chloroform-*d*) δ -62.83 (s, 3F), -68.85 (s, 2F), -113.22 (s, 1F).
^{13}C NMR (126 MHz, Chloroform-*d*) δ 163.0, 161.1, 139.0 (d, *J* = 12.6 Hz), 138.8 (d, *J* = 2.3 Hz), 135. 1 (t, *J* = 33.5 Hz), 131.8 (q, *J* = 33.3 Hz), 129.8, 129.4 (t, *J* = 4.3 Hz), 128.5-128.3 (m), 126.4, 124.5, 123.2 (q, *J* = 273.4 Hz), 123.2-123.0 (m), 122.1, 121.0 (d, *J* = 10.4 Hz), 120. 1 (t, *J* =252.0 Hz), 119.8, 113.0 (t, *J* = 5.3 Hz), 109.9 (d, *J* = 24.0 Hz), 100.7 (dt, *J* = 28.9, 6.2 Hz).
HRMS (FI) calcd. for $C_{20}H_{11}NF_6$ $[M]^+$: 379.0790, found: 379.0788.

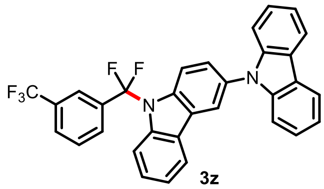

3z

9-(difluoro(3-(trifluoromethyl)phenyl)methyl)-9H-3,9'-bicarbazole (3z)

According to the general procedure A, the crude product is purified by flash chromatography on Al_2O_3 (PE : NEt_3=100:1). (0.1 mmol scale, 28.3 mg, 54%). Rf= 0.6 (PE/EA = 20:1). Light yellow solid.

m.p.: 91.7-92.5 ºC.
IR: ν = 3050, 2925, 1494, 1453, 1316, 1230, 1090, 745, 723, 422 cm^{-1}.
^{1}H NMR (500 MHz, Chloroform-*d*) δ 8.28 (d, *J* = 2.1 Hz, 1H), 8.23 (d, *J* = 7.8 Hz, 2H), 8.11 – 8.05 (m, 2H), 7.88 (d, *J* = 7.8 Hz, 1H), 7.70 (d, *J* = 7.9 Hz, 1H), 7.66 (d, *J* = 8.8 Hz, 1H), 7.62 (t, *J* = 7.7 Hz, 1H), 7.59 (dd, *J* = 8.8, 2.1 Hz, 1H), 7.48 – 7.43 (m, 6H), 7.39 – 7.33 (m, 3H).
^{19}F NMR (471 MHz, Chloroform-*d*) δ -62.67 (s, 3F), -68.42 (s, 2F).
^{13}C NMR (126 MHz, Chloroform-*d*) δ 141.5, 139.1, 137.4, 135.3 (t, *J* = 33.5 Hz), 131.8 (q, *J* = 33.2 Hz), 131.8, 129.9, 129.6 – 129.4 (m), 128.6 – 128.2 (m), 127.5, 126.3, 126.1, 125.9, 124.5, 123.4 (q, *J* = 273.4 Hz), 123.2, 123.4 – 123.1 (m), 122.1, 120.5, 120.3, 120.2 (t, *J* = 252.0 Hz), 119.8, 119.1, 114.3 (t, *J* = 5.9 Hz), 113.3 (t, *J* = 5.4 Hz), 109.7.
HRMS (FI) calcd. for $C_{32}H_{19}N_2F_5$ $[M]^+$: 526,1463, found: 526,1464.

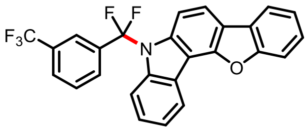

3aa

5-(difluoro(3-(trifluoromethyl)phenyl)methyl)-5H-benzofuro[3,2-c]carbazole (3aa)

According to the general procedure A, the crude product is purified by flash chromatography on Al_2O_3 (PE : NEt_3=100:1). (0.1 mmol scale, 27.2 mg, 60%). Rf= 0.7 (PE/EA = 10:1). Light yellow solid.

m.p.: 123.8-124.4 °C.
IR: ν = 2973, 1315, 1280, 1228, 1167, 1051, 749, 740, 727, 641 cm^{-1}.
^{1}H NMR (500 MHz, Chloroform-*d*) δ 8.59 – 8.53 (m, 1H), 8.01 – 7.95 (m, 2H), 7.90 (d, *J* = 8.7 Hz, 1H), 7.78 (dt, *J* = 6.8, 1.7 Hz, 1H), 7.74 (d, *J* = 8.2 Hz, 1H), 7.52 – 7.37 (m, 8H). **^{19}F NMR** (471 MHz, Chloroform-*d*) δ -62.77 (s, 3F), -68.18 (s, 2F).
^{13}C NMR (126 MHz, Chloroform-*d*) δ 156.4 , 150.7 , 138.5 , 138.3 , 135.5 (t, *J* = 33.8 Hz), 131.7 (q, *J* = 33.1 Hz), 129.7 , 129.4 (t, *J* = 4.2 Hz), 128.3 (dt, *J* = 3.8, 2.1 Hz), 126.6 , 126.0 , 124.4 , 123.4 (q, *J* = 273.4 Hz), 123.3 – 123.1 (m), 123.1 , 122.9 , 122.6 , 122.4 , 120.3 (t, *J* = 252.1 Hz), 120.0 , 118.6 , 118.2 , 113.0 (t, *J* = 6.0 Hz), 111.8 , 110.6 , 108.4 (t, *J* = 6.0 Hz).
HRMS (FI) calcd. for $C_{26}H_{14}ONF_5$ $[M]^+$: 451.0990, found: 451.0994.

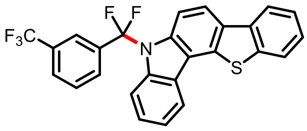

3bb

5-(difluoro(3-(trifluoromethyl)phenyl)methyl)-5H-benzo[4,5]thieno[3,2-c]carbazole (3bb)

According to the general procedure A, the crude product is purified by flash chromatography on Al_2O_3 (PE : NEt_3=100:1). (0.1 mmol scale, 26.7 mg, 57%). Rf= 0.7 (PE/EA = 10:1). Light yellow solid.

m.p.: 107.9-109.9 °C.
IR: ν = 2926, 1426, 1310, 1231, 1162, 1115, 912, 752, 730, 429 cm^{-1}.
^{1}H NMR (500 MHz, Chloroform-*d*) δ 8.29 – 8.25 (m, 1H), 8.19 – 8.16 (m, 1H), 8.13 (d, *J* = 8.8 Hz, 1H), 8.01 (s, 1H), 8.00 – 7.96 (m, 1H), 7.81 – 7.75 (m, 1H), 7.55 (dt, *J* = 8.8, 1.7 Hz, 1H), 7.54 – 7.45 (m, 7H).
^{19}F NMR (471 MHz, Chloroform-*d*) δ -62.75 (s, 3F), -68.06 (s, 2F).
^{13}C NMR (126 MHz, Chloroform-*d*) δ 138.9, 138.4, 137.4, 135.4 (t, *J* = 33.8 Hz), 135.3, 132.4, 131.7 (q, *J* = 33.1 Hz), 130.5, 129.7, 129.5 – 129.2 (m), 128.5 – 128.0 (m), 126.6, 125.9, 124.7, 124.1, 123.4 (q, *J* = 273.4 Hz), 123.2-123.0 (m), 122.9, 122.3, 121.8, 121.2, 120.3 (t, *J* = 252.0 Hz) 119.9, 118.9, 113.1 (t, *J* = 5.9 Hz), 110.3 (t, *J* = 6.0 Hz).
HRMS (FI) calcd. for $C_{26}H_{14}SNF_5$ $[M]^+$: 467.0762, found: 462.0763.

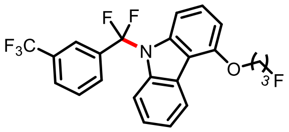

3cc

9-(difluoro(3-(trifluoromethyl)phenyl)methyl)-4-(3-fluoropropoxy)-9H-carbazole (3cc)

According to the general procedure A, the crude product is purified by flash chromatography on Al_2O_3 (PE : NEt_3=100:1). (0.1 mmol scale, 22.8 mg, 52%). Rf= 0.7 (PE/EA = 20:1). Light yellow oil.

IR: ν = 2970, 1451, 1314, 1269, 1163, 1045, 785, 748, 697, 432 cm^{-1}.
^{1}H NMR (500 MHz, Chloroform-*d*) δ 8.40 – 8.32 (m, 1H), 7.97 (s, 1H), 7.80 – 7.74 (m, 1H), 7.52 – 7.46 (m, 2H), 7.45 – 7.41 (m, 1H), 7.39 – 7.32 (m, 2H), 7.30 (t, *J* = 8.2 Hz, 1H), 7.04 (dt, *J* = 8.4, 1.9 Hz, 1H), 6.80 (d, *J* = 8.0 Hz, 1H), 4.83 (dt, *J* = 47.0, 5.8 Hz, 2H), 4.39 (t, *J* = 6.0 Hz, 2H), 2.48 – 2.35 (m, 2H). **^{19}F NMR** (471 MHz, Chloroform-*d*) δ -62.76 (s, 3F), -68.47 (s, 2F), -221.58 (s, 1F).
^{13}C NMR (126 MHz, Chloroform-*d*) δ 155.0, 139.8, 137.8, 135.6 (t, *J* = 33.8 Hz), 131.6 (q, *J* = 33.1 Hz), 129.6, 129.4 (t, *J* = 4.1 Hz), 128.3 – 128.1 (m), 127.5, 125.8, 124.3, 123.4 (q, *J* = 273.4 Hz), 123.2, 123.1 – 123.0 (m), 121.9, 120.2 (t, *J* = 251.8 Hz), 114.1, 112.5 (t, *J* = 5.9 Hz), 105.9 (t, *J* = 5.9 Hz), 103.5, 80.9 (d, *J* = 165.1 Hz), 63.9 (d, *J* = 5.2 Hz), 30.6 (d, *J* = 20.1 Hz).
HRMS (FI) calcd. for $C_{23}H_{17}ONF_6$ [M]+: 437.1209, found: 437.1213.

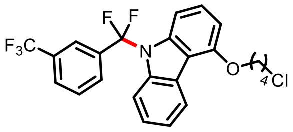

3dd

4-(4-chlorobutoxy)-9-(difluoro(3-(trifluoromethyl)phenyl)methyl)-9H-carbazole (3dd)

According to the general procedure A, the crude product is purified by flash chromatography on Al_2O_3 (PE : NEt_3=100:1). (0.1 mmol scale, 22.7 mg, 49%). Rf= 0.7 (PE/EA = 20:1). Light yellow oil.

IR: ν = 2925, 1450, 1270, 1230, 1162, 1043, 786, 749, 695, 432 cm^{-1}.
^{1}H NMR (500 MHz, Chloroform-*d*) δ 8.40 – 8.28 (m, 1H), 7.90 (s, 1H), 7.78 – 7.68 (m, 1H), 7.48 – 7.42 (m, 2H), 7.40 – 7.35 (m, 1H), 7.34 – 7.28 (m, 2H), 7.24 (d, *J* = 8.6 Hz, 1H), 6.97 (dt, *J* = 8.3, 1.9 Hz, 1H), 6.73 (d, *J* = 8.0 Hz, 1H), 4.25 (t, *J* = 5.7 Hz, 2H), 3.68 (t, *J* = 6.0 Hz, 2H), 2.21 – 2.07 (m, 4H).
^{19}F NMR (471 MHz, Chloroform-*d*) δ -62.78 (s, 3F), -68.49 (s, 2F).
^{13}C NMR (126 MHz, Chloroform-*d*) δ 155.2, 139.8, 137.8, 135.6 (t, *J* = 33.8 Hz), 131.6 (q, *J* = 33.2 Hz), 129.7, 129.5 – 129.3 (m), 128.3 – 128.0 (m), 127.5, 125.8, 124.3, 123.5 (q, *J* = 273.4 Hz), 123.3, 123.3 – 123.0 (m), 121.9, 120.2 (t, *J* = 251.8 Hz), 114.2, 112.5 (t, *J* = 5.9 Hz), 105.8(t, *J* = 5.8 Hz), 103.4, 67.2, 44.8, 29.5, 26.8.
HRMS (FI) calcd. for $C_{24}H_{19}ONF_5Cl$ [M]+: 467.1070, found: 467.1072.

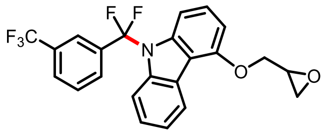

3ee

9-(difluoro(3-(trifluoromethyl)phenyl)methyl)-4-(oxiran-2-ylmethoxy)-9H-carbazole (3ee)

According to the general procedure A, the crude product is purified by flash chromatography on Al_2O_3 (PE : NEt_3=100:1). (0.1 mmol scale, 23.4 mg, 54%). Rf= 0.45 (PE/EA = 5:1). Light yellow oil.

IR: ν = 2928, 1271, 1231, 1163, 1122, 1075, 784, 748, 697, 431 cm^{-1}.
^{1}H NMR (500 MHz, Chloroform-*d*) δ 8.46 – 8.40 (m, 1H), 7.94 (s, 1H), 7.80 – 7.73 (m, 1H), 7.50 – 7.45 (m, 2H), 7.43 – 7.39 (m, 1H), 7.39 – 7.33 (m, 2H), 7.28 (t, *J* = 8.4 Hz, 1H), 7.05 (dt, *J* = 8.4, 1.9 Hz, 1H), 6.76 (d, *J* = 8.0 Hz, 1H), 4.50 (dd, *J* = 10.9, 3.1 Hz, 1H), 4.22 (dd, *J* = 10.9, 5.7 Hz, 1H), 3.56 (ddt, *J* = 5.7, 4.1, 3.1 Hz, 1H), 3.01 (dd, *J* = 4.9, 4.1 Hz, 1H), 2.88 (dd, *J* = 4.9, 2.7 Hz, 1H).
^{19}F NMR (471 MHz, Chloroform-*d*) δ -62.76 (s, 3F), -68.45 (s, 2F).
^{13}C NMR (126 MHz, Chloroform-*d*) δ 154.7, 139.8, 137.8, 135.5 (t, *J* = 33.8 Hz), 131.6 (q, *J* = 33.1 Hz), 129.6, 129.5 – 129.3 (m), 128.3 – 128.1 (m), 127.4, 125.9, 124.1, 123.5, 123.4 (q, *J* = 273.4 Hz), 123.2 – 122.9 (m), 122.0, 120.2 (t, *J* = 251.7 Hz), 114.4, 112.4 (t, *J* = 5.9 Hz), 106.3 (t, *J* = 5.9 Hz), 103.7, 69.0, 50.2, 44.7.
HRMS (FI) calcd. for $C_{23}H_{16}O_2NF_5$ $[M]^+$: 433.1096, found: 433.1098.

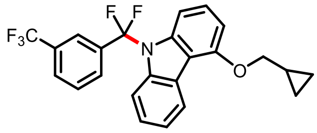

3ff

4-(cyclopropylmethoxy)-9-(difluoro(3-(trifluoromethyl)phenyl)methyl)-9H-carbazole (3ff)

According to the general procedure A, the crude product is purified by flash chromatography on Al_2O_3 (PE : NEt_3=100:1). (0.1 mmol scale, 28.9 mg, 67%). Rf= 0.6 (PE/EA = 10:1). Light yellow oil.

IR: ν = 2920, 1451, 1270, 1230, 1163, 1128, 1039, 748, 697, 430 cm^{-1}.
^{1}H NMR (500 MHz, Chloroform-*d*) δ 8.54 – 8.46 (m, 1H), 7.96 (s, 1H), 7.81 – 7.73 (m, 1H), 7.51 – 7.46 (m, 2H), 7.44 – 7.40 (m, 1H), 7.39 – 7.34 (m, 2H), 7.27 (t, *J* = 8.2 Hz, 1H), 7.01 (dt, *J* = 8.4, 1.9 Hz, 1H), 6.74 (d, *J* = 8.0 Hz, 1H), 4.10 (d, *J* = 6.8 Hz, 2H), 1.54 – 1.45 (m, 1H), 0.79 – 0.73 (m, 2H), 0.54 – 0.48 (m, 2H).
^{19}F NMR (471 MHz, Chloroform-*d*) δ -62.76 (s, 3F), -68.47 (s, 2F).
^{13}C NMR (126 MHz, Chloroform-*d*) δ 155.4, 139.8, 137.8, 135.6 (t, *J* = 33.9 Hz), 131.6 (q, *J* = 33.1 Hz), 129.6, 129.5 – 129.4 (m), 128.2 – 128.0 (m), 127.4, 125.7, 124.5, 123.5, 123.5 (q, *J* = 273.4 Hz), 123.3 – 123.0 (m), 121.9, 120.2 (t, *J* = 251.6 Hz), 114.3, 112.4 (t, *J* = 5.8 Hz), 105.6 (t, *J* = 5.8 Hz), 103.6, 72.8, 10.4, 3.2.
HRMS (FI) calcd. for $C_{24}H_{18}ONF_5$ $[M]^+$: 431.1303, found: 431.1306.

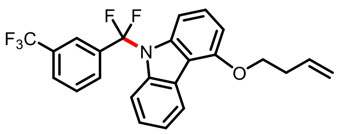

3gg

4-(but-3-en-1-yloxy)-9-(difluoro(3-(trifluoromethyl)phenyl)methyl)-9H-carbazole (3gg)

According to the general procedure A, the crude product is purified by flash chromatography on Al_2O_3 (PE : NEt_3=100:1). (0.1 mmol scale, 18.5 mg, 43%). Rf= 0.5 (PE/EA = 20:1). Light yellow oil.

IR: ν = 2933, 1314, 1270, 1231, 1163, 1127, 784, 747, 697, 431 cm^{-1}.
^{1}H NMR (500 MHz, Chloroform-*d*) δ 8.45 – 8.39 (m, 1H), 7.95 (s, 1H), 7.79 – 7.73 (m, 1H), 7.51 – 7.46 (m, 2H), 7.43 – 7.39 (m, 1H), 7.38 – 7.31 (m, 2H), 7.28 (t, *J* = 8.2 Hz, 1H), 7.01 (dt, *J* = 8.4, 1.9 Hz, 1H), 6.77 (d, *J* = 8.0 Hz, 1H), 6.07 (ddt, *J* = 17.2, 10.2, 6.8 Hz, 1H), 5.33 (dq, *J* = 17.2, 1.7 Hz, 1H), 5.21 (dq, *J* = 10.2, 1.4 Hz, 1H), 4.30 (t, *J* = 6.4 Hz, 2H), 2.78 (qt, *J* = 6.4, 1.4 Hz, 2H).
^{19}F NMR (471 MHz, Chloroform-*d*) δ -62.76 (s, 3F), -68.48 (s, 2F).
^{13}C NMR (126 MHz, Chloroform-*d*) δ 155.3, 139.8, 137.8, 135.6 (t, *J* = 34.0 Hz), 134.7, 131.6 (q, *J* = 33.0 Hz), 129.6, 129.5 – 129.3 (m), 128.3 – 128.0 (m), 127.4, 125.8, 124.4, 123.5, 123.4 (q, *J* = 273.4 Hz), 123.2 – 122.9 (m), 121.8, 120.2 (t, *J* = 251.7 Hz), 117.2, 114.2, 112.4 (t, *J* = 5.9 Hz), 105.7 (t, *J* = 5.8 Hz), 103.4, 67.4, 33.9.
HRMS (FI) calcd. for $C_{24}H_{18}ONF_5$ $[M]^+$: 431.1303, found: 431.1310.

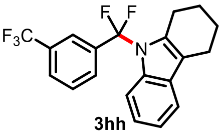

3hh

9-(difluoro(3-(trifluoromethyl)phenyl)methyl)-2,3,4,9-tetrahydro-1H-carbazole (3hh)

According to the general procedure B, the crude product is purified by flash chromatography on Al_2O_3 (PE : NEt_3=100:1). (0.2 mmol scale, 11.0 mg, 15%). Rf= 0.7 (PE/EA = 20:1). Light yellow oil.

IR: ν = 2936, 1336, 1308, 1230, 1168, 1128, 1077, 906, 740, 697 cm^{-1}.
^{1}H NMR (500 MHz, Chloroform-*d*) δ 7.82 (s, 1H), 7.77 (d, *J* = 7.8 Hz, 1H), 7.53 (t, *J* = 7.9 Hz, 1H), 7.47 (d, *J* = 7.7 Hz, 1H), 7.41 (d, *J* = 7.9 Hz, 1H), 7.20 – 7.13 (m, 2H), 7.12 – 7.07 (m, 1H), 2.75 – 2.70 (m, 2H), 2.66 – 2.61 (m, 2H), 1.89 – 1.82 (m, 4H).
^{19}F NMR (471 MHz, Chloroform-*d*) δ -62.78 (s, 3F), -66.12 (s, 2F).
^{13}C NMR (126 MHz, Chloroform-*d*) δ 136.4 (t, *J* = 34.1 Hz), 135.8, 134.9, 131.5 (q, *J* = 32.9 Hz), 129.6, 129.5 – 129.3 (m), 129.2, 128.1 – 127.9 (m), 123.4 – 122.9 (m), 122.7, 121.1, 119.8 (t, *J* = 250.8 Hz), 118.0, 115.2, 112.7 (t, *J* = 5.3 Hz).
HRMS (FI) calcd. for $C_{20}H_{16}NF_5$ $[M]^+$: 365.1197, found: 365.1202.

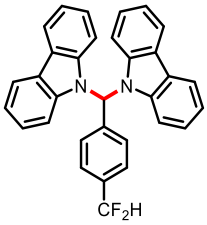

3ii

9,9'-((4-(difluoromethyl)phenyl)methylene)bis(9H-carbazole) (3ii)

According to the general procedure A, the crude product is purified by flash chromatography on Al_2O_3 (PE : NEt_3=100: 1). (0.05 mmol scale, 17.2 mg, 73%). Rf= 0.7 (PE/EA = 10:1). Light yellow oil.

IR: ν = 3051, 2945, 1444, 1423, 1287, 1264, 1221, 746, 721, 629 cm^{-1};
^{1}H NMR (500 MHz, Chloroform-*d*) δ 8.42 (s, 1H), 8.15 – 8.08 (m, 4H), 7.49 (d, *J* = 8.0 Hz, 2H), 7.27 – 7.19 (m, 10H), 6.94 – 6.87 (m, 4H), 6.66 (t, *J* = 56.2 Hz, 1H).
^{19}F NMR (471 MHz, Chloroform-*d*) δ -111.27 (s, 2F).
^{13}C NMR (126 MHz, Chloroform-*d*) δ 139.9, 138.0 (t, *J* = 2.0 Hz), 135.5 (t, *J* = 22.6 Hz), 128.2, 126.6 (t, *J* = 6.0 Hz), 126.3, 123.9, 120.5, 120.3, 114.1 (t, *J* = 239.5 Hz), 110.5, 68.9.
HRMS (FI) calcd. for $C_{32}H_{22}N_2F_2$ $[M]^+$: 472.1746, found: 472.1741.

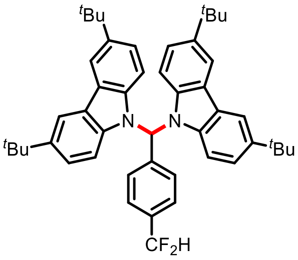

3jj

9,9'-((4-(difluoromethyl)phenyl)methylene)bis(3,6-di-tert-butyl-9H-carbazole) (3jj)

According to the general procedure A, the crude product is purified by flash chromatography on Al_2O_3 (PE : NEt_3=100: 1). (0.05 mmol scale, 23.0 mg, 66%). Rf= 0.7 (PE/EA = 10:1). Yellow wax.

IR: ν = 2956, 2904, 1490, 1466, 1364, 1293, 905, 808, 730, 612 cm^{-1};
^{1}H NMR (500 MHz, Chloroform-*d*) δ 8.34 (s, 1H), 8.12 (d, *J* = 2.0 Hz, 4H), 7.48 (d, *J* = 8.1 Hz, 2H), 7.27 (dd, *J* = 8.7, 2.0 Hz, 4H), 7.19 (d, *J* = 8.0 Hz, 2H), 6.84 (d, *J* = 8.7 Hz, 4H), 6.67 (t, *J* = 56.3 Hz, 1H), 1.42 (s, 36H).
^{19}F NMR (471 MHz, Chloroform-*d*) δ -111.14 (s, 2F).
^{13}C NMR (126 MHz, Chloroform-*d*) δ 142.8, 138.5 (t, *J* = 2.1 Hz), 138.4, 135.1 (t, *J* = 22.6 Hz), 128.3, 126.4 (t, *J* = 6.0 Hz), 123.9, 123.7, 116.3, 114.2 (t, *J* = 239.3 Hz), 109.9, 68.6, 34.6, 31.9.
HRMS (ESI) calcd. for $C_{48}H_{54}F_2N_2Na$ $[M+Na]^+$: 719.4147, found:719.4144.

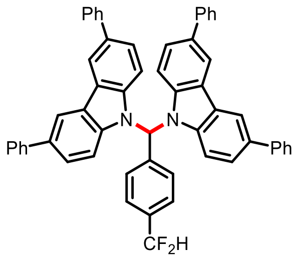

3kk

9,9'-((4-(difluoromethyl)phenyl)methylene)bis(3,6-diphenyl-9H-carbazole) (3kk)

According to the general procedure A, the crude product is purified by flash chromatography on Al_2O_3 (PE : NEt_3=100: 1). (0.05 mmol scale, 23.5 mg, 60%). Rf= 0.6 (PE/EA = 10:1). Light yellow wax.

IR: ν = 2956, 2834, 1601, 1462, 1205, 1171, 1036, 819, 743, 723 cm^{-1};

^{1}H NMR (500 MHz, Chloroform-*d*) δ 8.53 (s, 1H), 8.43 (d, *J* = 1.8 Hz, 4H), 7.74 – 7.68 (m, 8H), 7.61 (d, *J* = 8.1 Hz, 2H), 7.56 (dd, *J* = 8.6, 1.9 Hz, 4H), 7.48 (t, *J* = 7.7 Hz, 8H), 7.40 – 7.34 (m, 6H), 7.05 (d, *J* = 8.6 Hz, 4H), 6.75 (t, *J* = 56.2 Hz, 1H).

^{19}F NMR (471 MHz, Chloroform-*d*) δ -111.27 (s, 2F).

^{13}C NMR (126 MHz, Chloroform-*d*) δ 141.4, 139.8, 137.8 (t, *J* = 2.3 Hz), 135.7 (t, *J* = 22.7 Hz), 134.0, 128.8, 128.3, 127.3,127.2, 126.8, 126.1, 124.6, 119.0, 114.1 (t, *J* = 239.6 Hz), 110.8, 69.1.

HRMS (ESI) calcd. for $C_{56}H_{39}F_2N_2$ $[M+H]^+$: 777.3076, found: 777.3060.

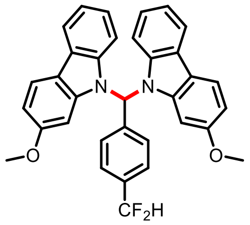

3ll

9,9'-((4-(difluoromethyl)phenyl)methylene)bis(2-methoxy-9H-carbazole) (3ll)

According to the general procedure A, the crude product is purified by flash chromatography on Al_2O_3 (PE : NEt_3=100: 1). (0.05 mmol scale, 17.4 mg, 65%). Rf= 0.6 (PE/EA = 10:1). White foam.

IR: ν = 3029, 2926, 1601, 1476, 1456, 1268, 1228, 812, 760, 697 cm^{-1};

^{1}H NMR (500 MHz, Chloroform-*d*) δ 8.37 (s, 1H), 8.05 – 7.99 (m, 2H), 7.94 (d, *J* = 8.6 Hz, 2H), 7.56 (d, *J* = 8.1 Hz, 2H), 7.38 – 7.33 (m, 2H), 7.26 – 7.21 (m, 4H), 7.03 – 6.97 (m, 2H), 6.80 (dd, *J* = 8.6, 2.2 Hz, 2H), 6.69 (t, *J* = 56.0 Hz, 1H), 6.27 (d, *J* = 2.2 Hz, 2H), 3.33 (s, 6H).

^{19}F NMR (471 MHz, Chloroform-*d*) δ -111.04 (s, 2F).

^{13}C NMR (126 MHz, Chloroform-d) δ 159.0, 141.0, 140.1, 138.0, 135.6 (t, J = 22.6 Hz), 128.4, 126.7 (t, J = 5.9 Hz), 124.9, 124.0, 121.1, 120.5, 119.6, 117.4, 114.1 (t, J = 239.3 Hz), 109.7, 109.4, 94.9, 68.7, 55.0.

HRMS (ESI) calcd. for $C_{34}H_{27}N_2O_2F_2$ $[M+H]^+$: 533.2035, found: 533.2033.

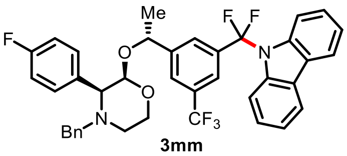

(2R,3S)-2-((R)-1-(3-((9H-carbazol-9-yl)difluoromethyl)-5-(trifluoromethyl)phenyl)ethoxy)-4-benzyl-3-(4-fluorophenyl)morpholine (3mm)

According to the general procedure B, the crude product is purified by flash chromatography on Al_2O_3 (PE : EA :NEt_3=100:2:1). (0.1 mmol scale, 32.8 mg, 49%). Rf= 0.5 (PE/EA = 10:1). Light yellow oil.

IR: ν = 2976, 1509, 1447, 1222, 1171, 1130, 1057, 879, 732, 724 cm^{-1}
^{1}H NMR (500 MHz, Chloroform-*d*) δ 8.09 – 7.99 (m, 2H), 7.58 (s, 1H), 7.38 – 7.16 (m, 12H), 7.15 – 7.06 (m, 3H), 6.85 (t, *J* = 8.6 Hz, 2H), 4.78 (q, *J* = 6.6 Hz, 1H), 4.33 (d, *J* = 3.0 Hz, 1H), 4.17 (td, *J* = 11.6, 2.5 Hz, 1H), 3.74 (d, *J* = 13.4 Hz, 1H), 3.52 (ddd, *J* = 11.2, 3.5, 1.7 Hz, 1H), 3.34 (d, *J* = 3.0 Hz, 1H), 2.84 – 2.73 (m, 2H), 2.29 (td, *J* = 11.9, 3.5 Hz, 1H), 1.38 (d, *J* = 6.6 Hz, 3H).
^{19}F NMR (471 MHz, Chloroform-*d*) δ -62.70 (s, 3F), -68.42 (s, 2F), -115.50 (s, 1F).
^{13}C NMR (126 MHz, Chloroform-*d*) δ 162.3 (d, *J* = 246.4 Hz), 145.9, 138.4, 137.8, 135.4 (t, *J* = 32.8 Hz), 133.4 (d, *J* = 3.0 Hz), 131.8 (q, *J* = 33.0 Hz), 130.7 (d, *J* = 7.9 Hz), 128.9, 128.2, 127.6 – 127.3 (m), 127.0, 126.6, 125.9 – 125.6 (m), 125.0, 123.1 (q, *J* = 273.4 Hz) 122.2 (q, *J* = 4.1 Hz), 121.8, 120.1, 119.8 (t, *J* = 252.0 Hz), 114.9 (d, *J* = 21.3 Hz), 113.1 (t, *J* = 5.1 Hz), 95.5, 72.1, 69.2, 59.7, 59.5, 51.6, 24.6.
HRMS (FI) calcd. for $C_{39}H_{32}O_2N_2F_6$ $[M]^+$: 674.2362, found: 674.2360.

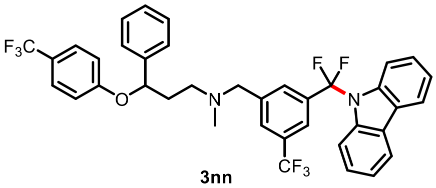

N-(3-((9H-carbazol-9-yl)difluoromethyl)-5-(trifluoromethyl)benzyl)-N-methyl-3-phenyl-3-(4-(trifluoromethyl)phenoxy)propan-1-amine (3nn)

According to the general procedure B, the crude product is purified by flash chromatography on Al_2O_3 (PE : EA :NEt_3=100:2:1). (0.1 mmol scale, 29.8 mg, 44%). Rf= 0.7 (PE/EA = 10:1). Light yellow oil.

IR: ν = 2954, 1615, 1423, 1223, 1161, 1108, 1077, 749, 724, 700 cm^{-1}
^{1}H NMR (500 MHz, Chloroform-*d*) δ 8.10 – 8.05 (m, 2H), 7.81 (s, 1H), 7.74 (s, 1H), 7.59 (s, 1H), 7.41 (d, *J* = 8.6 Hz, 2H), 7.38 – 7.30 (m, 8H), 7.29 – 7.23 (m, 3H), 6.82 (d, *J* = 8.5 Hz, 2H), 5.18 (dd, *J* = 8.3, 4.7 Hz, 1H), 3.49 (s, 2H), 2.60 – 2.50 (m, 1H), 2.49 – 2.39 (m, 1H), 2.20 – 2.12 (m, 1H), 2.10 (s, 3H), 1.98 – 1.89 (m, 1H).
^{19}F NMR (471 MHz, Chloroform-*d*) δ -61.52 (s, 3F), -62.57 (s, 3F), -68.43 (s, 2F).
^{13}C NMR (126 MHz, Chloroform-*d*) δ 160.5, 142.1, 141.0, 138.5, 135.5 (t, *J* = 33.2 Hz), 131.7 (q, *J* = 33.0 Hz), 129.7 (t, *J* = 4.5 Hz), 128.7, 128.4 (d, *J* = 4.0 Hz), 127.8, 126.7 (t, *J* = 3.7 Hz), 126.7, 125.8, 125.0, 124.4 (q, *J* = 270.9 Hz), 123.5 (q, *J* = 273.4 Hz), 122.7 (q, *J* = 32.8 Hz) 121.9 – 121.7 (m), 121.8, 120.2 (t, *J* = 252.0 Hz), 120.2, 115.6, 113.2 (t, *J* = 5.4 Hz), 78.1, 61.4, 53.5, 41.8, 36.5.
HRMS (FI) calcd. for $C_{38}H_{30}ON_2F_8$ $[M]^+$: 682.2225, found: 682.2221.

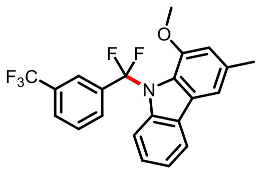

3oo

9-(difluoro(3-(trifluoromethyl)phenyl)methyl)-1-methoxy-3-methyl-9H-carbazole (3oo)

According to the general procedure B, the crude product is purified by flash chromatography on Al_2O_3 (PE : NEt_3=100:1). (0.1 mmol scale, 22.8 mg, 56%). Rf= 0.7 (PE/EA = 10:1). Light yellow oil.

IR: ν = 2965, 1668, 1588, 1299, 1277, 1231, 1143, 751, 665, 582 cm^{-1}.
^{1}H NMR (500 MHz, Chloroform-*d*) δ 8.08 – 8.04 (m, 1H), 7.97 (s, 1H), 7.92 – 7.86 (m, 1H), 7.74 – 7.68 (m, 1H), 7.51 – 7.50 (m, 1H), 7.49 – 7.46 (m, 1H), 7.45 – 7.41 (m, 2H), 7.38 – 7.34 (m, 1H), 6.63 (s, 1H), 3.42 (s, 3H), 2.49 (s, 3H).
^{19}F NMR (471 MHz, Chloroform-*d*) δ -62.58 (s, 3F), -64.37 (s, 2F).
^{13}C NMR (126 MHz, Chloroform-*d*) δ 145.9, 140.1, 138.7 (t, *J* = 33.4 Hz), 132.7, 130.8 (q, *J* = 32.8 Hz), 128.9, 128.7 – 128.4 (m), 127.4, 126.9 – 126.9 (m), 126.9 – 126.8 (m), 125.8, 124.6, 123.8 (q, *J* = 273.4 Hz), 122.4-122.3 (m), 121.4, 120.6 (t, *J* = 253.9 Hz), 120.0, 114.6 (t, *J* = 11.6 Hz), 112.4, 110.8, 54.9, 21.4.
HRMS (FI) calcd. for $C_{22}H_{16}ONF_5$ $[M]^+$: 405.1147, found: 405.1153.

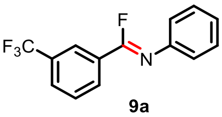

9a

***N*-phenyl-3-(trifluoromethyl)benzimidoyl fluoride (9a)**

According to the general procedure D, the crude product is purified by flash chromatography on Al_2O_3 (PE). (0.2 mmol scale, 25.7 mg, 48%). Rf= 0.9 (PE/EA = 10:1). Colorless oil.

IR: ν = 3065, 1650, 1538, 1435, 1317, 1167, 1122, 1072, 753, 692 cm^{-1}.
^{1}H NMR (500 MHz, Chloroform-*d*) δ 8.33 (s, 1H), 8.23 (d, *J* = 7.9 Hz, 1H), 7.82 (d, *J* = 7.9 Hz, 1H), 7.62 (t, *J* = 7.8 Hz, 1H), 7.44 – 7.38 (m, 2H), 7.29 (d, *J* = 7.3 Hz, 2H), 7.26 – 7.21 (m, 1H).
^{19}F NMR (471 MHz, Chloroform-*d*) δ -46.63 (s, 1F), -62.85 (s, 3F).
^{13}C NMR (126 MHz, Chloroform-*d*) δ 146.8 (d, *J* = 341.6 Hz), 142.3 (d, *J* = 11.9 Hz), 131.6 (d, *J* = 4.0 Hz), 131.3 (q, *J* = 32.8, 32.3 Hz), 130.2 (d, *J* = 45.1 Hz), 129.2 , 128.9, 128.7 (q, *J* = 3.6 Hz), 126.0 , 125.6 – 125.4 (m), 123.6 (q, *J* = 273.4 Hz), 123.6 (d, *J* = 2.4 Hz).
HRMS (ESI) calcd. for $C_{14}H_{10}F_4N$ $[M+H]^+$: 268.0744, found: 268.0733.

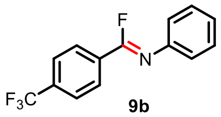

9b

***N*-phenyl-4-(trifluoromethyl)benzimidoyl fluoride (9b)**

According to the general procedure D, the crude product is purified by flash chromatography on Al_2O_3 (PE). (0.2 mmol scale, 32.2 mg, 60%). Rf= 0.9 (PE/EA = 10:1). White solid.

m.p. 71.2-72.5 °C
IR: ν = 2925, 1651, 1533, 1328, 1171, 1116, 1069, 754, 693, 507 cm^{-1}.
^{1}H NMR (500 MHz, Chloroform-*d*) δ 8.18 (d, *J* = 8.1 Hz, 2H), 7.75 (d, *J* = 8.2 Hz, 2H), 7.46 – 7.38 (m, 2H), 7.30 (d, *J* = 7.3 Hz, 2H), 7.27 – 7.22 (m, 1H).
^{19}F NMR (471 MHz, Chloroform-*d*) δ -46.26 (s, 1F), -63.05 (s, 3F).
^{13}C NMR (126 MHz, Chloroform-*d*) δ 146.9 (d, *J* = 342.2 Hz), 142.3 (d, *J* = 11.9 Hz), 133.7 (q, *J* = 32.9 Hz), 132.6 (d, *J* = 45.0 Hz), 128.9 , 128.9 (d, *J* = 4.5 Hz), 126.0, 125.6 (q, *J* = 3.8 Hz), 123.7 (q, *J* = 273.4 Hz), 123.7 (d, *J* = 2.7 Hz).
HRMS (ESI) calcd. for $C_{14}H_{10}F_4N$ $[M+H]^+$: 268.0744, found: 268.0734.

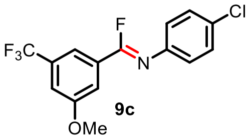

9c

***N*-(4-chlorophenyl)-3-methoxy-5-(trifluoromethyl)benzimidoyl fluoride (9c)**

According to the general procedure D, the crude product is purified by flash chromatography on Al_2O_3 (PE). (0.2 mmol scale, 28.6 mg, 43%). Rf= 0.9 (PE/EA = 10:1). Pale yellow oil.

IR: ν = 2941, 2850, 1699, 1463, 1360, 1253, 1125, 1036, 839, 693 cm^{-1}.
^{1}H NMR (500 MHz, Chloroform-*d*) δ 7.87 (s, 1H), 7.71 (s, 1H), 7.38 – 7.34 (m, 2H), 7.32 (s, 1H), 7.25 – 7.21 (m, 2H), 3.92 (s, 3H).
^{19}F NMR (471 MHz, Chloroform-*d*) δ -45.61 (s, 1F), -62.94 (s, 3F).
^{13}C NMR (126 MHz, Chloroform-*d*) δ 160.0, 147.3 (d, *J* = 342.5 Hz), 140.7 (d, *J* = 12.2 Hz), 132.5 (q, *J* = 33.5 Hz), 131.6, 131.2 (d, *J* = 44.8 Hz), 129.1, 125.2 (d, *J* = 2.6 Hz), 123.4 (q, *J* = 273.4 Hz), 118.1 – 117.3 (m), 116.6 (d, *J* = 4.5 Hz), 115.1 (q, *J* = 3.6 Hz), 55.9.
HRMS (ESI) calcd. for $C_{15}H_{11}ClF_4NO$ $[M+H]^+$: 332.0460, found: 332.0452.

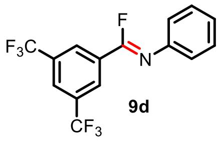

***N*-phenyl-3,5-bis(trifluoromethyl)benzimidoyl fluoride (9d)**

According to the general procedure D, the crude product is purified by flash chromatography on Al_2O_3 (PE). (0.2 mmol scale, 28.3 mg, 42%). Rf= 0.9 (PE/EA = 10:1). Pale yellow oil.

IR: ν = 3091, 2924, 1704, 1385, 1275, 1175, 1129, 882, 697, 692 cm^{-1}.
^{1}H NMR (500 MHz, Chloroform-*d*) δ 8.50 (s, 2H), 8.06 (s, 1H), 7.45 – 7.40 (m, 2H), 7.33 (d, *J* = 7.4 Hz, 2H), 7.30 – 7.25 (m, 1H).
^{19}F NMR (471 MHz, Chloroform-*d*) δ -46.52 (s, 1F), -63.03 (s, 6F).
^{13}C NMR (126 MHz, Chloroform-*d*) δ 145.2 (d, *J* = 343.5 Hz), 141.6 (d, *J* = 11.0 Hz), 132.4 (q, *J* = 34.2 Hz), 131.7 (d, *J* = 47.4 Hz), 129.1, 128.5 (t, *J* = 3.9 Hz), 126.7, 125.4 (p, *J* = 3.8 Hz), 124.0 (d, *J* = 2.8 Hz), 122.9 (q, *J* = 272.9 Hz).
HRMS (ESI) calcd. for $C_{15}H_9F_7N$ $[M+H]^+$: 336.0618, found: 336.0609.

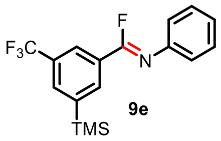

***N*-phenyl-3-(trifluoromethyl)-5-(trimethylsilyl)benzimidoyl fluoride (9e)**

According to the general procedure D, the crude product is purified by flash chromatography on Al_2O_3 (PE). (0.2 mmol scale, 38.0 mg, 56%). Rf= 0.9 (PE/EA = 10:1). Colorless oil.

IR: ν = 2958, 1703, 1345, 1251, 1173, 1130, 1104, 840, 758, 690 cm^{-1}.
^{1}H NMR (500 MHz, Chloroform-*d*) δ 8.36 (s, 1H), 8.29 (s, 1H), 7.92 (s, 1H), 7.44 – 7.37 (m, 2H), 7.29 (d, *J* = 7.3 Hz, 2H), 7.25 – 7.21 (m, 1H), 0.38 (s, 9H).
^{19}F NMR (471 MHz, Chloroform-*d*) δ -46.47 (s, 1F), -62.66 (s, 3F).
^{13}C NMR (126 MHz, Chloroform-*d*) δ 147.3 (d, *J* = 342.2 Hz), 143.1, 142.5 (d, *J* = 12.2 Hz), 136.3, 133.1 (q, *J* = 3.6 Hz), 130.6 (q, *J* = 32.3 Hz), 129.3 (d, *J* = 44.1 Hz), 128.9, 125.8, 125.8 – 125.6 (m), 123.9 (q, *J* = 272.8 Hz), 123.5 (d, *J* = 2.2 Hz), -1.4.
HRMS (ESI) calcd. for $C_{17}H_{18}F_4NSi$ $[M+H]^+$: 340.1139, found: 340.1130.

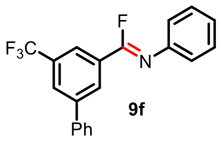

9f

***N*-phenyl-5-(trifluoromethyl)-[1,1'-biphenyl]-3-carbimidoyl fluoride (9f)**

According to the general procedure D, the crude product is purified by flash chromatography on Al_2O_3 (PE). (0.2 mmol scale, 30.9 mg, 45%). Rf= 0.9 (PE/EA = 10:1). Colorless oil.

IR: ν = 2928, 1704, 1345, 1256, 1176, 1130, 1123, 1074, 755, 692 cm^{-1}.
^{1}H NMR (500 MHz, Chloroform-*d*) δ 8.45 (s, 1H), 8.28 (s, 1H), 8.03 – 7.99 (m, 1H), 7.70 – 7.64 (m, 2H), 7.55 – 7.49 (m, 2H), 7.47 – 7.40 (m, 3H), 7.33 – 7.29 (m, 2H), 7.26 – 7.22 (m, 1H).
^{19}F NMR (471 MHz, Chloroform-*d*) δ -46.30 (s, 1F), -62.70 (s, 3F).
^{13}C NMR (126 MHz, Chloroform-*d*) δ 146.9 (d, *J* = 342.3 Hz), 142.8, 142.3 (d, *J* = 11.9 Hz), 138.7, 131.9 (q, *J* = 33.2 Hz), 130.8 (d, *J* = 44.9 Hz), 130.2 (d, *J* = 3.5 Hz), 129.2 , 128.9, 128.6, 127.4 – 127.1 (m), 127.2, 126.0, 124.1 – 123.9 (m), 123.7 (q, *J* = 273.4 Hz), 123.7 (d, *J* = 2.4 Hz).
HRMS (ESI) calcd. for $C_{20}H_{14}F_4N$ $[M+H]^+$: 344.1057, found: 344.1049.

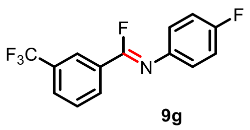

9g

***N*-(4-fluorophenyl)-3-(trifluoromethyl)benzimidoyl fluoride (9g)**

According to the general procedure D, the crude product is purified by flash chromatography on Al_2O_3 (PE). (0.2 mmol scale, 28.1 mg, 49%). Rf= 0.9 (PE/EA = 10:1). colorless oil.

IR: ν = 2928, 2856, 1700, 1504, 1337, 1251, 1129, 844, 694, 460 cm^{-1}.
^{1}H NMR (500 MHz, Chloroform-*d*) δ 8.31 (s, 1H), 8.21 (d, *J* = 7.9 Hz, 1H), 7.85 – 7.78 (m, 1H), 7.62 (t, *J* = 7.9 Hz, 1H), 7.35 – 7.29 (m, 2H), 7.12 – 7.04 (m, 2H).
^{19}F NMR (471 MHz, Chloroform-*d*) δ -46.84 (s, 1F), -62.88 (s, 3F), -115.84 (s, 1F).
^{13}C NMR (126 MHz, Chloroform-*d*) δ 160.8 (d, *J* = 246.0 Hz), 146.8 (d, *J* = 342.2 Hz), 139.0 – 137.3 (m), 131.5 (d, *J* = 4.0 Hz), 131.2, 130.1 (d, *J* = 45.2 Hz), 129.3, 128.7 (q, *J* = 3.8 Hz), 125.9 (dd, *J* = 8.4, 3.2 Hz), 125.6 – 125.2 (m), 123.6 (q, *J* = 273.4 Hz), 115.7 (d, *J* = 22.5 Hz).
HRMS (ESI) calcd. for $C_{14}H_9F_5N$ $[M+H]^+$: 286.0650, found: 286.0642.

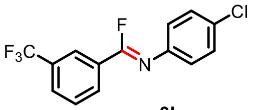

9h

***N*-(4-chlorophenyl)-3-(trifluoromethyl)benzimidoyl fluoride (9h)**

According to the general procedure D, the crude product is purified by flash chromatography on Al_2O_3 gel (PE). (0.2 mmol scale, 37.8 mg, 63%). Rf= 0.9 (PE/EA = 10:1). colorless oil.

IR: ν = 2927, 1699, 1488, 1336, 1168, 1127, 1095, 840, 805, 692 cm^{-1}.
^{1}H NMR (500 MHz, Chloroform-*d*) δ 8.31 (s, 1H), 8.21 (d, J = 7.9 Hz, 1H), 7.85 – 7.80 (m, 1H), 7.62 (t, J = 7.8 Hz, 1H), 7.39 – 7.33 (m, 2H), 7.24 (d, J = 8.7 Hz, 2H).
^{19}F NMR (471 MHz, Chloroform-*d*) δ -46.06 (s, 1F), -62.88 (s, 3F).
^{13}C NMR (126 MHz, Chloroform-*d*) δ 147.3 (d, J = 342.4 Hz), 140.7 (d, J = 12.2 Hz), 131.6 (d, J = 4.7 Hz), 131.3, 129.9 (d, J = 45.0 Hz), 129.3, 129.1, 128.8 (q, J = 3.7 Hz), 125.6 – 125.4 (m), 125.3, 125.2, 123.6 (d, J = 272.6 Hz).
HRMS (ESI) calcd. for $C_{14}H_9ClF_4N$ $[M+H]^+$: 302.0354, found: 302.0346.

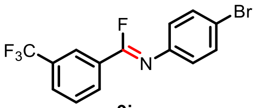

9i

***N*-(4-bromophenyl)-3-(trifluoromethyl)benzimidoyl fluoride (9i)**

According to the general procedure D, the crude product is purified by flash chromatography on Al_2O_3 gel (PE). (0.2 mmol scale, 38.4 mg, 56%). Rf= 0.9 (PE/EA = 10:1). colorless oil.

IR: ν = 2928, 2856, 1699, 1485, 1336, 1252, 1169, 1129, 1073, 693 cm^{-1}.
^{1}H NMR (500 MHz, Chloroform-*d*) δ 8.31 (s, 1H), 8.21 (d, J = 7.8 Hz, 1H), 7.85 – 7.78 (m, 1H), 7.63 (t, J = 7.8 Hz, 1H), 7.54 – 7.46 (m, 2H), 7.17 (d, J = 8.6 Hz, 2H).
^{19}F NMR (471 MHz, Chloroform-*d*) δ -45.94 (s, 1F), -62.88 (s, 3F).
^{13}C NMR (126 MHz, Chloroform-*d*) δ 147.4 (d, J = 342.4 Hz), 141.2 (d, J = 12.4 Hz), 132.0, 131.6 (d, J = 4.5 Hz), 131.4 (d, J = 33.3 Hz), 129.9 (d, J = 44.7 Hz), 129.3, 128.9 (q, J = 3.7 Hz), 125.7 – 125.4 (m), 125.5, 123.6 (q, J = 272.6 Hz), 119.4.
HRMS (ESI) calcd. for $C_{14}H_9BrF_4N$ $[M]^+$: 345.9849, found: 345.9846.

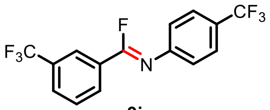

9j

3-(trifluoromethyl)-*N*-(4-(trifluoromethyl)phenyl)benzimidoyl fluoride (9j)

According to the general procedure D, the crude product is purified by flash chromatography on Al_2O_3 gel (PE). (0.2 mmol scale, 40.1 mg, 60%). Rf= 0.9 (PE/EA = 10:1). colorless oil.

IR: ν = 2928, 1660, 1606, 1534, 1318, 1165, 1119, 1067, 815, 696 cm^{-1}.
^{1}H NMR (500 MHz, Chloroform-*d*) δ 8.33 (s, 1H), 8.23 (d, *J* = 7.9 Hz, 1H), 7.85 (d, *J* = 7.8 Hz, 1H), 7.71 – 7.62 (m, 3H), 7.31 (d, *J* = 8.2 Hz, 2H).
^{19}F NMR (471 MHz, Chloroform-*d*) δ -45.18 (s, 1F), -62.27 (s, 3F), -62.92 (s, 3F).
^{13}C NMR (126 MHz, Chloroform-*d*) δ 148.1 (d, *J* = 341.8 Hz), 145.6 (d, *J* = 12.9 Hz), 131.8 (d, *J* = 4.3 Hz), 131.5 (q, *J* = 34.0 Hz), 129.5 (d, *J* = 43.9 Hz), 129.4 , 129.2 (q, *J* = 3.7 Hz), 127.7 (q, *J* = 32.5 Hz), 126.2 (q, *J* = 3.7 Hz), 125.8 – 125.6 (m), 124.2 (q, *J* = 272.2 Hz), 123.5 (q, *J* = 272.7 Hz), 123.3.
HRMS (ESI) calcd. for $C_{15}H_9F_7N$ $[M+H]^+$: 336.0618, found: 361.0596.

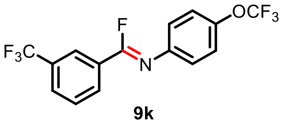

9k

***N*-(4-(trifluoromethoxy)phenyl)-3-(trifluoromethyl)benzimidoyl fluoride (9k)**

0According to the general procedure D, the crude product is purified by flash chromatography on Al_2O_3 gel (PE). (0.2 mmol scale, 45.1 mg, 64%). Rf= 0.9 (PE/EA = 10:1). colorless oil.

IR: ν = 2928, 1702, 1504, 1338, 1254, 1222, 1196, 1166, 1131, 693 cm^{-1}.
^{1}H NMR (500 MHz, Chloroform-*d*) δ 8.32 (s, 1H), 8.22 (d, *J* = 7.9 Hz, 1H), 7.86 – 7.81 (m, 1H), 7.63 (t, *J* = 7.8 Hz, 1H), 7.35 – 7.29 (m, 2H), 7.26 – 7.21 (m, 2H).
^{19}F NMR (471 MHz, Chloroform-*d*) δ -46.17 (s, 1F), -58.01 (s, 3F), -62.93 (s, 3F).
^{13}C NMR (126 MHz, Chloroform-*d*) δ 147.5 (d, *J* = 342.5 Hz), 146.9, 140.8 (d, *J* = 12.2 Hz), 131.6 (d, *J* = 4.1 Hz), 131.4 (q, *J* = 33.2 Hz), 129.8 (d, *J* = 44.8 Hz), 129.3, 128.9 (q, *J* = 3.6 Hz), 125.9 – 125.4 (m), 125.2 (d, *J* = 2.3 Hz), 123.6 (q, *J* = 273.4 Hz), 120.5 (q, *J* = 257.0 Hz), 121.5.
HRMS (ESI) calcd. for $C_{15}H_9F_7NO$ $[M+H]^+$: 352.0567, found: 352.0560.

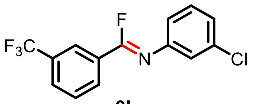

9l

***N*-(3-chlorophenyl)-3-(trifluoromethyl)benzimidoyl fluoride (9l)**

According to the general procedure D, the crude product is purified by flash chromatography on Al_2O_3 gel (PE). (0.2 mmol scale, 34.4 mg, 57%). Rf= 0.9 (PE/EA = 10:1). colorless oil.

IR: ν = 2929, 2856, 1706, 1337, 1250, 1212, 1170, 1128, 746, 693 cm^{-1}.
^{1}H NMR (500 MHz, Chloroform-*d*) δ 8.31 (s, 1H), 8.21 (d, *J* = 7.9 Hz, 1H), 7.83 (d, *J* = 6.8 Hz, 1H), 7.63 (t, *J* = 7.9 Hz, 1H), 7.32 (t, *J* = 8.0 Hz, 1H), 7.28 (t, *J* = 2.0 Hz, 1H), 7.23 – 7.19 (m, 1H), 7.18 – 7.13 (m, 1H).
^{19}F NMR (471 MHz, Chloroform-*d*) δ -45.37 (s, 1F), -62.89 (s, 3F).
^{13}C NMR (126 MHz, Chloroform-*d*) δ 147.7 (d, *J* = 342.4 Hz), 143.5 (d, *J* = 12.7 Hz), 134.5 , 131.7 (d, *J* = 4.3 Hz), 131.4 (q, *J* = 34.2, 33.8 Hz), 129.9 , 129.5, 129.3, 129.0 (q, *J* = 3.7 Hz), 126.0, 125.7 – 125.4 (m), 123.7 (d, *J* = 2.3 Hz), 123.6 (q, *J* = 272.3 Hz), 121.8 (d, *J* = 2.3 Hz).
HRMS (ESI) calcd. for $C_{14}H_9ClF_4N$ $[M+H]^+$: 302.0354, found: 302.0346.

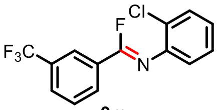

9m

***N*-(2-chlorophenyl)-3-(trifluoromethyl)benzimidoyl fluoride (9m)**

According to the general procedure D, the crude product is purified by flash chromatography on Al_2O_3 gel (PE). (0.2 mmol scale, 24.5 mg, 41%). Rf= 0.9 (PE/EA = 10:1). colorless oil.

IR: ν = 2928, 2856, 1699, 1615, 1336, 1248, 1126, 1073, 693, 680cm^{-1}.
^{1}H NMR (500 MHz, Chloroform-*d*) δ 8.35 (s, 1H), 8.28 (d, *J* = 7.9 Hz, 1H), 7.85 (d, *J* = 7.9 Hz, 1H), 7.65 (t, *J* = 7.9 Hz, 1H), 7.46 (dd, *J* = 8.1, 1.4 Hz, 1H), 7.28 (td, *J* = 7.7, 1.4 Hz, 1H), 7.19 – 7.12 (m, 2H).
^{19}F NMR (471 MHz, Chloroform-*d*) δ -41.51 (s, 1F), -62.87 (s, 3F).
^{13}C NMR (126 MHz, Chloroform-*d*) δ 148.3 (d, *J* = 341.0 Hz), 140.5 (d, *J* = 12.1 Hz), 132.0 (d, *J* = 4.3 Hz), 131.5 (q, *J* = 33.2 Hz), 129.9, 129.6, 129.4, 129.2 (q, *J* = 3.7 Hz), 127.1, 126.6, 126.2, 125.9 – 125.5 (m), 123.6 (q, *J* = 272.4 Hz), 123.0 (d, *J* = 1.7 Hz).
HRMS (ESI) calcd. for $C_{14}H_9ClF_4N$ $[M+H]^+$: 302.0354, found: 302.0347.

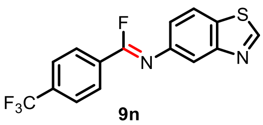

9n

N-(benzo[d]thiazol-5-yl)-4-(trifluoromethyl)benzimidoyl fluoride (9n)

According to the general procedure D, the crude product is purified by flash chromatography on Al_2O_3 gel (PE/EA = 40:1). (0.2 mmol scale, 28.9 mg, 45%). Rf= 0.5 (PE/EA = 10:1). Pale yellow solid.

m.p. 92.3-93.7 °C
IR: ν = 3041, 2927, 1694, 1323, 1174, 1127, 1067, 1006, 849, 635 cm^{-1}.
^{1}H NMR (500 MHz, Chloroform-*d*) δ 9.04 (s, 1H), 8.20 (d, *J* = 8.1 Hz, 2H), 8.08 (s, 1H), 7.95 (d, *J* = 8.5 Hz, 1H), 7.76 (d, *J* = 8.2 Hz, 2H), 7.42 (d, *J* = 8.5 Hz, 1H).
^{19}F NMR (471 MHz, Chloroform-*d*) δ -45.78 (s, 1F), -63.05 (s, 3F).
^{13}C NMR (126 MHz, Chloroform-*d*) δ 155.1, 153.9, 147.5 (d, *J* = 343.1 Hz), 140.9 (d, *J* = 12.2 Hz), 133.9 (q, *J* = 32.8 Hz), 132.4 (d, *J* = 44.6 Hz), 131.2, 129.0 (d, *J* = 4.3 Hz), 125.6 (q, *J* = 3.9 Hz), 123.6 (q, *J* = 272.6 Hz), 122.4, 121.9, 118.3 (d, *J* = 2.9 Hz).
HRMS (ESI) calcd. for $C_{15}H_9F_4N_2S$ $[M+H]^+$: 325.0417, found: 325.0409.

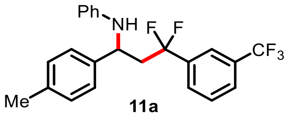

11a

N-(3,3-difluoro-1-(p-tolyl)-3-(3-(trifluoromethyl)phenyl)propyl)aniline (11a)

According to the general procedure E, the crude product is purified by flash chromatography on silica gel (PE/EA = 80:1). (0.2 mmol scale, 54.3 mg, 67%). Rf= 0.6 (PE/EA = 10:1). Pale yellow oil.

IR: ν = 3439, 2926, 1602, 1504, 1336, 1167, 1125, 1075, 701, 691 cm^{-1}.
^{1}H NMR (500 MHz, Chloroform-*d*) δ 7.74 – 7.68 (m, 2H), 7.66 (d, *J* = 7.9 Hz, 1H), 7.53 (t, *J* = 7.8 Hz, 1H), 7.23 – 7.18 (m, 2H), 7.15 – 7.05 (m, 4H), 6.74 – 6.65 (m, 1H), 6.49 – 6.42 (m, 2H), 4.53 (dd, *J* = 8.7, 4.5 Hz, 1H), 4.18 (s, 1H), 2.80 – 2.52 (m, 2H), 2.32 (s, 3H).
^{19}F NMR (471 MHz, Chloroform-*d*) δ -62.80 (s, 3F), -91.49 (d, *J* = 250.8 Hz, 1F), -94.45 (d, *J* = 250.9 Hz, 1F).
^{13}C NMR (126 MHz, Chloroform-*d*) δ 146.5, 139.6, 137.7 (t, *J* = 27.1 Hz), 137.2, 131.1 (q, *J* = 32.9 Hz), 129.5, 129.2, 129.1, 128.4 (t, *J* = 6.2 Hz), 126.8 – 126.6 (m), 126.0, 123.6 (q, *J* = 273.4 Hz), 122.2 – 121.8 (m), 121.6 (t, *J* = 244.5 Hz), 117.8, 113.4, 53.5 (t, *J* = 3.6 Hz), 47.1 (t, *J* = 26.2 Hz), 21.0.
HRMS (ESI) calcd. for $C_{23}H_{21}F_5N$ $[M+H]^+$: 406.1589, found: 406.1581.

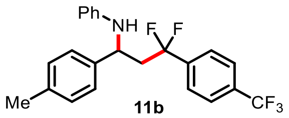

***N*-(3,3-difluoro-1-(p-tolyl)-3-(4-(trifluoromethyl)phenyl)propyl)aniline (11b)**

According to the general procedure E, the crude product is purified by flash chromatography on silica gel (PE/EA = 80:1). (0.2 mmol scale, 50.3 mg, 62%). Rf= 0.6 (PE/EA = 10:1). Pale yellow oil.

IR: ν = 3453, 2927, 1603, 1505, 1323, 1168, 1128, 1068, 819, 750 cm^{-1}.
^{1}H NMR (500 MHz, Chloroform-*d*) δ 7.65 (d, *J* = 8.2 Hz, 2H), 7.57 (d, *J* = 8.2 Hz, 2H), 7.21 – 7.16 (m, 2H), 7.13 – 7.05 (m, 4H), 6.71 – 6.64 (m, 1H), 6.45 – 6.38 (m, 2H), 4.49 (dd, *J* = 9.0, 4.4 Hz, 1H), 4.15 (s, 1H), 2.77 – 2.52 (m, 2H), 2.31 (s, 3H).
^{19}F NMR (471 MHz, Chloroform-*d*) δ -62.91 (s, 3F), -91.58 (d, *J* = 250.9 Hz, 1F), -94.78 (d, *J* = 251.0 Hz, 1F).
^{13}C NMR (126 MHz, Chloroform-*d*) δ 146.5, 140.2 (t, *J* = 26.8 Hz), 139.6, 137.2, 132.1 (q, *J* = 34.0, 33.0 Hz), 129.5, 129.1, 126.0, 125.7 – 125.3 (m), 123.6 (q, *J* = 273.4 Hz), 121.7 (t, *J* = 245.7 Hz), 117.8, 113.4, 53.5 (t, *J* = 3.8 Hz), 47.2 (t, *J* = 26.1 Hz), 21.0.
HRMS (ESI) calcd. for $C_{23}H_{21}F_5N$ $[M+H]^+$: 406.1589, found: 406.1580.

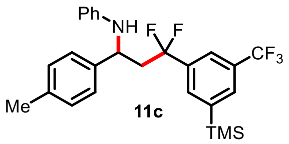

***N*-(3,3-difluoro-1-(p-tolyl)-3-(3-(trifluoromethyl)-5-(trimethylsilyl)phenyl)propyl)aniline (11c)**

According to the general procedure E, the crude product is purified by flash chromatography on silica gel (PE/EA = 100:1). (0.2 mmol scale, 63.0 mg, 66%). Rf= 0.7 (PE/EA = 10:1). Pale yellow oil.

IR: ν = 3440, 2927, 1675, 1605, 1321, 1168, 1127, 1065, 813, 750 cm^{-1}.
^{1}H NMR (500 MHz, Chloroform-*d*) δ 7.78 (s, 1H), 7.73 (s, 1H), 7.69 – 7.65 (m, 1H), 7.21 – 7.16 (m, 2H), 7.13 – 7.04 (m, 4H), 6.69 – 6.64 (m, 1H), 6.44 – 6.38 (m, 2H), 4.44 (dd, *J* = 8.9, 4.5 Hz, 1H), 4.19 (s, 1H), 2.77 – 2.52 (m, 2H), 2.31 (s, 3H), 0.24 (s, 9H).
^{19}F NMR (471 MHz, Chloroform-*d*) δ -62.62 (s, 3F), -90.87 (d, *J* = 250.5 Hz, 1F), -93.92 (d, *J* = 250.8 Hz, 1F).
^{13}C NMR (126 MHz, Chloroform-*d*) δ 146.5, 143.2, 139.6, 137.1, 136.6 (t, *J* = 26.6 Hz), 132.8 (t, *J* = 5.2 Hz), 131.3 – 131.1 (m), 130.3 (q, *J* = 32.2 Hz), 129.5, 129.1 , 126.1, 123.9 (q, *J* = 273.4 Hz), 122.4 – 122.1 (m), 122.0 (t, *J* = 245.7 Hz), 117.8, 113.3, 53.6 (t, *J* = 3.7 Hz), 47.2 (t, *J* = 26.3 Hz), 21.0, -1.5.
HRMS (ESI) calcd. for $C_{26}H_{29}F_5NSi$ $[M+H]^+$: 478.1984, found: 478.1982.

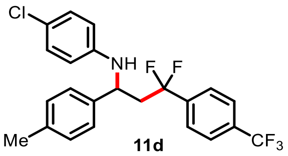

11d

4-chloro-*N*-(3,3-difluoro-1-(p-tolyl)-3-(4-(trifluoromethyl)phenyl)propyl)aniline (11d)

According to the general procedure E, the crude product is purified by flash chromatography on silica gel (PE/EA = 80:1). (0.2 mmol scale, 51.1 mg, 58%). Rf= 0.6 (PE/EA = 10:1). Pale yellow oil.

IR: ν = 3441, 2927, 1677, 1600, 1321, 1167, 1127, 1067, 843, 813 cm^{-1}.
^{1}H NMR (500 MHz, Chloroform-*d*) δ 7.66 (d, *J* = 8.2 Hz, 2H), 7.57 (d, *J* = 8.2 Hz, 2H), 7.16 (d, *J* = 8.1 Hz, 2H), 7.11 (d, *J* = 8.0 Hz, 2H), 7.05 – 6.98 (m, 2H), 6.37 – 6.29 (m, 2H), 4.43 (dd, *J* = 9.1, 4.2 Hz, 1H), 4.19 (s, 1H), 2.75 – 2.49 (m, 2H), 2.31 (s, 3H).
^{19}F NMR (471 MHz, Chloroform-*d*) δ -62.91 (s, 3F), -91.49 (d, *J* = 251.2 Hz, 1F), -95.04 (d, *J* = 251.1 Hz, 1F).
^{13}C NMR (126 MHz, Chloroform-*d*) δ 145.1, 140.1 (t, *J* = 26.8 Hz), 139.1, 137.4, 132.2 (q, *J* = 32.6 Hz), 129.6, 128.9, 125.9, 125.6 (q, *J* = 3.9 Hz), 125.5 (t, *J* = 6.1 Hz), 123.6 (q, *J* = 273.4 Hz), 122.5, 121.6 (t, *J* = 245.7 Hz), 114.5, 53.6 (dd, *J* = 4.4, 2.7 Hz), 47.2 (t, *J* = 26.2 Hz), 21.0.
HRMS (ESI) calcd. for $C_{23}H_{20}ClF_5N$ $[M+H]^+$: 440.1199, found: 440.1193.

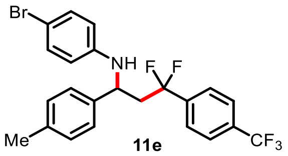

11e

4-bromo-*N*-(3,3-difluoro-1-(p-tolyl)-3-(4-(trifluoromethyl)phenyl)propyl)aniline (11e)

According to the general procedure E, the crude product is purified by flash chromatography on silica gel (PE/EA = 80:1). (0.2 mmol scale, 50.4 mg, 52%). Rf= 0.6 (PE/EA = 10:1). Pale yellow oil.

IR: ν = 3438, 2926, 1594, 1496, 1322, 1168, 1128, 1068, 843, 812 cm^{-1}.
^{1}H NMR (500 MHz, Chloroform-*d*) δ 7.66 (d, *J* = 8.2 Hz, 2H), 7.56 (d, *J* = 8.2 Hz, 2H), 7.18 – 7.13 (m, 4H), 7.10 (d, *J* = 7.9 Hz, 2H), 6.33 – 6.25 (m, 2H), 4.43 (dd, *J* = 9.1, 4.2 Hz, 1H), 4.21 (s, 1H), 2.73 – 2.51 (m, 2H), 2.31 (s, 3H).
^{19}F NMR (471 MHz, Chloroform-*d*) δ -62.91 (s, 3F), -91.48 (d, *J* = 251.2 Hz, 1F), -95.05 (d, *J* = 251.4 Hz, 1F).
^{13}C NMR (126 MHz, Chloroform-*d*) δ 145.5, 140.1 (t, *J* = 26.7 Hz), 139.0, 137.4, 132.2 (q, *J* = 32.8 Hz), 131.8, 129.6, 125.9, 125.6 (q, *J* = 3.9 Hz), 125.5 (t, *J* = 6.2 Hz), 123.6 (q, *J* = 273.4 Hz), 121.6 (t, *J* = 245.7 Hz), 115.0, 109.6, 53.5 (dd, *J* = 4.6, 2.8 Hz), 47.2, 21.0.
HRMS (ESI) calcd. for $C_{23}H_{20}BrF_5N$ $[M]^+$: 484.0694, found: 484.0687.

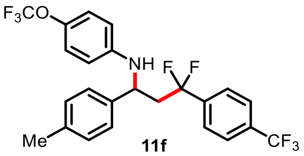

***N*-(3,3-difluoro-1-(p-tolyl)-3-(4-(trifluoromethyl)phenyl)propyl)-4-(trifluoromethoxy)aniline (11f)**

According to the general procedure E, the crude product is purified by flash chromatography on silica gel (PE/EA = 80:1). (0.2 mmol scale, 63.6 mg, 65%). Rf= 0.6 (PE/EA = 10:1). Pale yellow oil.

IR: ν = 3447, 2927, 1616, 1530, 1318, 1163, 1109, 1065, 844, 824 cm^{-1}.
^{1}H NMR (500 MHz, Chloroform-*d*) δ 7.66 (d, *J* = 8.2 Hz, 2H), 7.56 (d, *J* = 8.2 Hz, 2H), 7.30 (d, *J* = 8.4 Hz,2H), 7.15 (d, *J* = 8.2 Hz, 2H), 7.11 (d, *J* = 8.0 Hz, 2H), 6.42 (d, *J* = 8.5 Hz, 2H), 4.58 – 4.41 (m, 2H), 2.73 – 2.52 (m, 2H), 2.31 (s, 3H).
^{19}F NMR (471 MHz, Chloroform-*d*) δ -61.19 (s, 3F), -62.96 (s, 3F), -91.57 (d, *J* = 251.6 Hz, 1F), -95.32 (d, *J* = 251.6 Hz, 1F).
^{13}C NMR (126 MHz, Chloroform-*d*) δ 148.9, 140.0 (t, *J* = 25.2 Hz), 138.7, 137.6, 132.3 (q, *J* = 33.1, 31.6 Hz), 129.7, 128.4 (q, *J* = 273.4 Hz), 126.5 (q, *J* = 3.7 Hz), 125.9, 125.7 (q, *J* = 3.9 Hz), 125.6 (q, *J* = 244.4 Hz), 125.5 (t, *J* = 6.3 Hz), 121.6 (t, *J* = 245.7 Hz), 119. 5 (q, *J* = 32.8 Hz), 112.6, 53.2 (t, *J* = 3.6 Hz), 47.2 (t, *J* = 26.4 Hz), 21.0.
HRMS (ESI) calcd. for $C_{24}H_{20}F_8NO$ $[M]^+$: 490.1412, found: 490.1404.

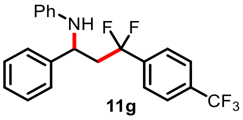

***N*-(3,3-difluoro-1-phenyl-3-(4-(trifluoromethyl)phenyl)propyl)aniline (11g)**

According to the general procedure E, the crude product is purified by flash chromatography on silica gel (PE/EA = 80:1). (0.2 mmol scale, 48.6 mg, 62%). Rf= 0.6 (PE/EA = 10:1). Pale yellow oil.

IR: ν = 3438, 2929, 1602, 1505, 1322, 1167, 1126, 1112, 1067, 749 cm^{-1}.
^{1}H NMR (500 MHz, Chloroform-*d*) δ 7.67 (d, *J* = 8.2 Hz, 2H), 7.59 (d, *J* = 8.2 Hz, 2H), 7.36 – 7.28 (m, 4H), 7.27 – 7.22 (m, 1H), 7.13 – 7.06 (m, 2H), 6.71 – 6.65 (m, 1H), 6.42 (d, *J* = 7.6 Hz, 2H), 4.53 (dd, *J* = 9.1, 4.2 Hz, 1H), 4.19 (s, 1H), 2.76 – 2.52 (m, 2H).
^{19}F NMR (471 MHz, Chloroform-*d*) δ -62.92 (s, 3F), -91.80 (d, *J* = 250.9 Hz, 1F), -94.77 (d, *J* = 250.9 Hz, 1F).
^{13}C NMR (126 MHz, Chloroform-*d*) δ 146.5, 142.7, 140.2 (t, *J* = 26.8 Hz), 132.2 (q, *J* = 32.9 Hz), 129.1, 128.9, 127.5, 126.1, 125.6 (q, *J* = 3.7 Hz), 125.5 (t, *J* = 6.2 Hz), 123.6 (q, *J* = 273.4 Hz), 121.7 (t, *J* = 245.7 Hz), 117.9, 113.4, 53. 8 (t, *J* = 3.7 Hz), 47.3 (t, *J* = 26.3 Hz).
HRMS (ESI) calcd. for $C_{22}H_{19}F_5N$ $[M+H]^+$: 392.1432, found: 392.1427.

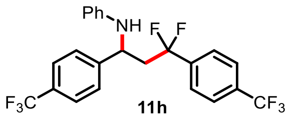

11h

***N*-(3,3-difluoro-1,3-bis(4-(trifluoromethyl)phenyl)propyl)aniline (11h)**

According to the general procedure E, the crude product is purified by flash chromatography on silica gel (PE/EA = 80:1). (0.2 mmol scale, 43.1 mg, 47%). Rf= 0.6 (PE/EA = 10:1). Pale yellow oil.

IR: ν = 3446, 2930, 1603, 1506, 1323, 1165, 1123, 1067, 844, 720 cm^{-1}.
^{1}H NMR (500 MHz, Chloroform-*d*) δ 7.67 (d, *J* = 8.2 Hz, 2H), 7.61 – 7.54 (m, 4H), 7.45 (d, *J* = 8.1 Hz, 2H), 7.13 – 7.06 (m, 2H), 6.74 – 6.67 (m, 1H), 6.42 – 6.34 (m, 2H), 4.60 (dd, *J* = 9.4, 3.9 Hz, 1H), 4.34 – 4.11 (m, 1H), 2.75 – 2.49 (m, 2H).
^{19}F NMR (471 MHz, Chloroform-*d*) δ -62.53 (s, 3F), -62.99 (s, 3F), -92.09 (d, *J* = 251.5 Hz, 1F), -94.78 (d, *J* = 251.6 Hz, 1F).
^{13}C NMR (126 MHz, Chloroform-*d*) δ 146.9, 146.0, 139.9 (t, *J* = 26.9 Hz), 132.4 (q, *J* = 32.8 Hz), 129.9 (q, *J* = 32.5 Hz), 129.2, 126.5, 125.9 (q, *J* = 3.6 Hz), 125.8 (q, *J* = 3.7 Hz), 125.5 (t, *J* = 6.2 Hz), 124.0 (q, *J* = 272.2 Hz), 123.6 (q, *J* = 272.2 Hz), 121.5 (t, *J* = 245.7 Hz), 118.4, 113.5, 53.6 (t, *J* = 3.6 Hz), 47.2 (t, *J* = 26.5 Hz).
HRMS (ESI) calcd. for $C_{23}H_{18}F_8N$ $[M+H]^+$: 460.1306, found: 460.1300.

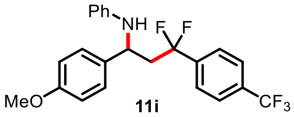

11i

***N*-(3,3-difluoro-1-(4-methoxyphenyl)-3-(4-(trifluoromethyl)phenyl)propyl)aniline (11i)**

According to the general procedure E, the crude product is purified by flash chromatography on silica gel (PE/EA = 80:1). (0.2 mmol scale, 38.1 mg, 45%). Rf= 0.6 (PE/EA = 10:1). Pale yellow oil.

IR: ν = 3447, 2927, 1603, 1511, 1325, 1248, 1171, 1129, 1069, 751 cm^{-1}.
^{1}H NMR (500 MHz, Chloroform-*d*) δ 7.65 (d, *J* = 8.2 Hz, 2H), 7.57 (d, *J* = 8.2 Hz, 2H), 7.24 – 7.16 (m, 2H), 7.12 – 7.03 (m, 2H), 6.87 – 6.79 (m, 2H), 6.70 – 6.64 (m, 1H), 6.45 – 6.37 (m, 2H), 4.47 (dd, *J* = 8.9, 4.4 Hz, 1H), 4.14 (s, 1H), 3.77 (s, 3H), 2.78 – 2.47 (m, 2H).
^{19}F NMR (471 MHz, Chloroform-*d*) δ -62.92 (s, 3F), -91.66 (d, *J* = 250.9 Hz, 1F), -94.85 (d, *J* = 251.4 Hz, 1F).
^{13}C NMR (126 MHz, Chloroform-*d*) δ 158. 9, 146.5, 140.3 (t, *J* = 26.5 Hz), 134.6, 132.1 (q, *J* = 32.8, 32.4 Hz), 129.1, 127.2, 125.6, 125.7 – 125.4 (m), 123.6 (q, *J* = 273.4 Hz), 121.7 (t, *J* = 244.4 Hz), 117.8, 114.2, 113.4, 55.2, 53.2 (t, *J* = 3.8 Hz), 47.2 (t, *J* = 26.1 Hz).
HRMS (ESI) calcd. for $C_{23}H_{21}F_5NO$ $[M]^+$: 422.1538, found: 422.1536.

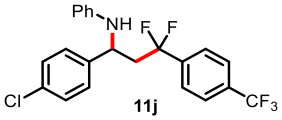

***N*-(1-(4-chlorophenyl)-3,3-difluoro-3-(4-(trifluoromethyl)phenyl)propyl)aniline (11j)**

According to the general procedure E, the crude product is purified by flash chromatography on silica gel (PE/EA = 80:1). (0.2 mmol scale, 34.3 mg, 40%). Rf= 0.6 (PE/EA = 10:1). Pale yellow oil.

IR: ν = 3435, 2928, 1602, 1323, 1168, 1128, 1068, 844, 751, 692 cm^{-1}.
^{1}H NMR (500 MHz, Chloroform-*d*) δ 7.66 (d, *J* = 8.1 Hz, 2H), 7.57 (d, *J* = 8.2 Hz, 2H), 7.29 – 7.22 (m, 4H), 7.11 – 7.04 (m, 2H), 6.72 – 6.65 (m, 1H), 6.40 – 6.33 (m, 2H), 4.49 (dd, *J* = 9.3, 4.0 Hz, 1H), 4.17 (s, 1H), 2.72 – 2.45 (m, 2H).
^{19}F NMR (471 MHz, Chloroform-*d*) δ -62.93 (s, 3F), -92.08 (d, *J* = 251.2 Hz, 1F), -94.73 (d, *J* = 251.2 Hz, 1F).
^{13}C NMR (126 MHz, Chloroform-*d*) δ 146.2, 141.3, 140.0 (t, *J* = 26.8 Hz), 133.2, 132.3 (q, *J* = 33.1 Hz), 129.2, 129.1, 127.5, 125.7 (q, *J* = 3.9 Hz), 125.5 (t, *J* = 6.2 Hz), 123.6 (q, *J* = 273.4 Hz), 121.5 (t, *J* = 245.7 Hz), 118.2, 113.5, 53.3 (t, *J* = 3.7 Hz), 47.3 (t, *J* = 26.4 Hz).
HRMS (ESI) calcd. for $C_{22}H_{18}ClF_5N$ $[M]^+$: 426.1042, found: 426.1037.

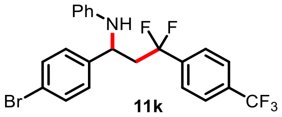

***N*-(1-(4-bromophenyl)-3,3-difluoro-3-(4-(trifluoromethyl)phenyl)propyl)aniline (11k)**

According to the general procedure E, the crude product is purified by flash chromatography on silica gel (PE/EA = 80:1). (0.2 mmol scale, 44.7 mg, 48%). Rf= 0.6 (PE/EA = 10:1). Pale yellow oil.

IR: ν = 3436, 2928, 1602, 1505, 1322, 1127, 1068, 1010, 844, 750 cm^{-1}.
^{1}H NMR (500 MHz, Chloroform-*d*) δ 7.67 (d, *J* = 8.2 Hz, 2H), 7.57 (d, *J* = 8.2 Hz, 2H), 7.47 – 7.39 (m, 2H), 7.23 – 7.17 (m, 2H), 7.12 – 7.05 (m, 2H), 6.74 – 6.66 (m, 1H), 6.40 – 6.34 (m, 2H), 4.48 (dd, *J* = 9.3, 4.0 Hz, 1H), 4.18 (s, 1H), 2.74 – 2.45 (m, 2H).
^{19}F NMR (471 MHz, Chloroform-*d*) δ -62.94 (s, 3F), -92.09 (d, *J* = 251.1 Hz, 1F), -94.73 (d, *J* = 251.4 Hz, 1F).
^{13}C NMR (126 MHz, Chloroform-*d*) δ 146.2, 141.8, 140.0 (t, *J* = 27.3 Hz), 132.3 (q, *J* = 32.8 Hz), 132.0, 129.2, 127.9, 125.8 (q, *J* = 3.6 Hz), 125.5 (t, *J* = 6.3 Hz), 123.6 (q, *J* = 273.4 Hz), 121.5 (t, *J* = 244.4 Hz), 121.3, 118.2, 113.5, 53.4 (t, *J* = 3.6 Hz), 47.2 (t, *J* = 26.4 Hz).
HRMS (ESI) calcd. for $C_{22}H_{18}BrF_5N$ $[M]^+$: 470.0537, found: 470.0532.

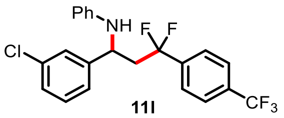

11l

***N*-(1-(3-chlorophenyl)-3,3-difluoro-3-(4-(trifluoromethyl)phenyl)propyl)aniline (11l)**

According to the general procedure E, the crude product is purified by flash chromatography on silica gel (PE/EA = 80:1). (0.2 mmol scale, 42.1 mg, 50%). Rf= 0.6 (PE/EA = 10:1). Pale yellow oil.

IR: ν = 3439, 2930, 1602, 1505, 1323, 1126, 1067, 844, 750, 692 cm^{-1}.
^{1}H NMR (500 MHz, Chloroform-*d*) δ 7.67 (d, *J* = 8.2 Hz, 2H), 7.58 (d, *J* = 8.2 Hz, 2H), 7.31 (s, 1H), 7.26 – 7.17 (m, 3H), 7.14 – 7.07 (m, 2H), 6.74 – 6.68 (m, 1H), 6.43 – 6.36 (m, 2H), 4.49 (dd, *J* = 9.4, 3.9 Hz, 1H), 4.19 (s, 1H), 2.73 – 2.49 (m, 2H).
^{19}F NMR (471 MHz, Chloroform-*d*) δ -62.95 (s, 3F), -91.95 (d, *J* = 251.3 Hz, 1F), -94.87 (d, *J* = 251.5 Hz, 1F).
^{13}C NMR (126 MHz, Chloroform-*d*) δ 146.2, 145.0, 140.0 (t, *J* = 26.7 Hz), 134.8, 132.3 (q, *J* = 33.0 Hz), 130.2, 129.2, 127.8, 126.3, 125.7 (q, *J* = 3.9 Hz), 125.5 (t, *J* = 6.2 Hz), 124.3, 123.6 (q, *J* = 273.4 Hz), 121.5 (t, *J* = 244.4 Hz), 118.2, 113.4, 53.6 (t, *J* = 3.7 Hz), 47.2 (t, *J* = 26.5 Hz).
HRMS (ESI) calcd. for $C_{22}H_{18}ClF_5N$ $[M]^+$: 426.1042, found: 426.1039.

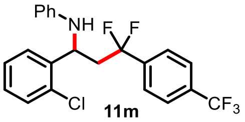

11m

***N*-(1-(2-chlorophenyl)-3,3-difluoro-3-(4-(trifluoromethyl)phenyl)propyl)aniline (11m)**

According to the general procedure E, the crude product is purified by flash chromatography on silica gel (PE/EA = 80:1). (0.2 mmol scale, 51.0 mg, 60%). Rf= 0.6 (PE/EA = 10:1). Pale yellow oil.

IR: ν = 3443, 2930, 1602, 1505, 1168, 1127, 1067, 845, 749, 691 cm^{-1}.
^{1}H NMR (500 MHz, Chloroform-*d*) δ 7.66 (d, *J* = 8.3 Hz, 2H), 7.62 (d, *J* = 8.3 Hz, 2H), 7.50 – 7.44 (m, 1H), 7.37 – 7.32 (m, 1H), 7.21 – 7.15 (m, 2H), 7.12 – 7.04 (m, 2H), 6.72 – 6.66 (m, 1H), 6.38 – 6.31 (m, 2H), 4.85 (dd, *J* = 10.2, 3.0 Hz, 1H), 4.37 (s, 1H), 2.76 – 2.63 (m, 1H), 2.62 – 2.46 (m, 1H).
^{19}F NMR (471 MHz, Chloroform-*d*) δ -62.90 (s, 3F), -90.01 (d, *J* = 251.5 Hz, 1F), -95.15 (d, *J* = 251.0 Hz, 1F).
^{13}C NMR (126 MHz, Chloroform-*d*) δ 146.0, 139.8 (t, *J* = 26.4 Hz), 139.3, 132.2 (q, *J* = 32.8 Hz), 132.1, 129.9, 129.2, 128.7, 127.6, 127.5, 125.7 – 125.4 (m), 125.6, 123.6 (q, *J* = 273.4 Hz), 121.9 (t, *J* = 245.7 Hz), 118.1, 113.2, 50.9 (dd, *J* = 5.3, 2.3 Hz), 44.8 (t, *J* = 26.6 Hz).
HRMS (ESI) calcd. for $C_{22}H_{18}ClF_5N$ $[M]^+$: 426.1042, found: 426.1035.

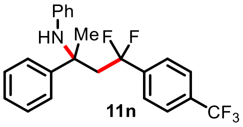

11n

N-(4,4-difluoro-2-phenyl-4-(4-(trifluoromethyl)phenyl)butan-2-yl)aniline (11n)

According to the general procedure E, the crude product is purified by flash chromatography on silica gel (PE/EA = 80:1). (0.2 mmol scale, 55.0 mg, 68%). Rf= 0.6 (PE/EA = 10:1). Pale yellow oil.

IR: ν = 3435, 2933, 1601, 1497, 1321, 1127, 1067, 842, 750, 698 cm^{-1}.
^{1}H NMR (500 MHz, Chloroform-*d*) δ 7.56 (d, *J* = 8.2 Hz, 2H), 7.49 – 7.39 (m, 4H), 7.33 – 7.27 (m, 2H), 7.25 – 7.20 (m, 1H), 7.02 – 6.93 (m, 2H), 6.68 – 6.61 (m, 1H), 6.22 – 6.14 (m, 2H), 4.33 (s, 1H), 3.04 – 2.89 (m, 1H), 2.82 – 2.65 (m, 1H), 1.84 (s, 3H).
^{19}F NMR (471 MHz, Chloroform-*d*) δ -62.97 (s, 3F), -86.81 (d, *J* = 249.7 Hz, 1F), -95.40 (d, *J* = 249.8 Hz, 1F).
^{13}C NMR (126 MHz, Chloroform-*d*) δ 146.2, 145.2, 141.2 (t, *J* = 26.6 Hz), 131.8 (q, *J* = 32.9 Hz), 128.7, 128.6, 126.8, 125.6, 125.4 (q, *J* = 4.1 Hz), 125.3 (t, *J* = 6.6 Hz), 123.6 (q, *J* = 273.4 Hz), 122. 5 (t, *J* = 245.3 Hz), 117.6, 115.7, 57.7 (d, *J* = 2.6 Hz), 48.3 (t, *J* = 25.0 Hz), 27.6 (t, *J* = 2.4 Hz).
HRMS (ESI) calcd. for $C_{23}H_{21}F_5N$ $[M+H]^+$: 406.1589, found: 406.1588.

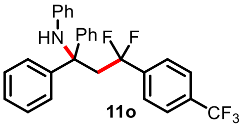

11o

N-(3,3-difluoro-1,1-diphenyl-3-(4-(trifluoromethyl)phenyl)propyl)aniline (11o)

According to the general procedure E, the crude product is purified by flash chromatography on silica gel (PE/EA = 80:1). (0.2 mmol scale, 60.6 mg, 65%). Rf= 0.8 (PE/EA = 10:1). Pale yellow oil.

IR: ν = 3435, 3058, 1601, 1498, 1324, 1169, 1129, 844, 749, 723 cm^{-1}.
^{1}H NMR (500 MHz, Chloroform-*d*) δ 7.47 – 7.42 (m, 6H), 7.28 – 7.22 (m, 6H), 7.20 – 7.15 (m, 2H), 6.97 – 6.91 (m, 2H), 6.67 – 6.61 (m, 1H), 6.26 – 6.20 (m, 2H), 4.97 (s, 1H), 3.53 (t, *J* = 15.6 Hz, 2H).
^{19}F NMR (471 MHz, Chloroform-*d*) δ -63.00 (s, 3F), -88.61 (s, 2F).
^{13}C NMR (126 MHz, Chloroform-*d*) δ 145.2, 143.9, 141.0 (t, *J* = 26.6 Hz), 131.4 (q, *J* = 32.8 Hz), 128. 6, 128.4, 126.9, 126.7, 125.2, 125.3 – 125.1 (m), 123.6 (q, *J* = 273.4 Hz), 122.1 (t, *J* = 246.0 Hz), 117.9, 115.9, 63.3, 46.3 (t, *J* = 25.3 Hz).
HRMS (ESI) calcd. for $C_{28}H_{23}F_5N$ $[M+H]^+$: 468.1745, found: 468.1737.

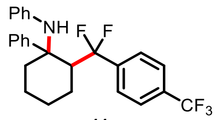

11p

***N*-(2-(difluoro(4-(trifluoromethyl)phenyl)methyl)-1-phenylcyclohexyl)aniline (11p)**

According to the general procedure E, the crude product is purified by flash chromatography on silica gel (PE/EA = 40:1). (0.2 mmol scale, 28.3 mg, 32%, d.r.>20:1). Rf= 0.6 (PE/EA = 10:1). Pale yellow oil.

IR: ν = 3428, 2939, 2870, 1601, 1498, 1325, 1169, 1069, 845, 698 cm^{-1}.
^{1}H NMR (500 MHz, Chloroform-*d*) δ 7.49 – 7.43 (m, 2H), 7.43 (d, *J* = 8.1 Hz, 2H), 7.18 (d, *J* = 8.1 Hz, 2H), 7.14 – 7.05 (m, 3H), 6.99 – 6.89 (m, 2H), 6.60 (t, *J* = 7.3 Hz, 1H), 6.21 (d, *J* = 8.0 Hz, 2H), 4.30 (s, 1H), 2.86 – 2.63 (m, 2H), 2.55 – 2.44 (m, 1H), 2.10 – 2.02 (m, 1H), 2.00 – 1.87 (m, 1H), 1.77 – 1.70 (m, 1H), 1.67 – 1.52 (m, 3H).
^{19}F NMR (471 MHz, Chloroform-*d*) δ -62.96 (s, 3F), -85.76 (d, *J* = 254.7 Hz), -93.00 (d, *J* = 254.8 Hz).
^{13}C NMR (101 MHz, Chloroform-*d*) δ 144.6, 144.3, 142.0 (t, *J* = 27.2 Hz), 130.7 (q, *J* = 32.3 Hz), 128.7, 128.1, 127.6, 127.0, 125.1 (t, *J* = 6.5 Hz), 124.9 (q, *J* = 3.9 Hz), 123.7 (q, *J* = 273.7 Hz), 123.0 (t, *J* = 248.5 Hz), 117. 8, 115.7, 59.1, 53.6 – 52.4 (m), 26.6, 23.3 (d, *J* = 6.5 Hz), 21.1, 21.0.
HRMS (ESI) calcd. for $C_{26}H_{25}F_5N$ $[M+H]^+$: 446.1902, found: 446.1902.

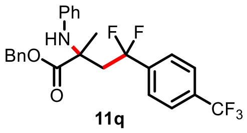

11q

benzyl 4,4-difluoro-2-methyl-2-(phenylamino)-4-(4-(trifluoromethyl)phenyl)butanoate (11q)

According to the general procedure E, the crude product is purified by flash chromatography on silica gel (PE/EA = 40:1). (0.2 mmol scale, 44.9 mg, 48%). Rf= 0.5 (PE/EA = 10:1). Pale yellow oil.

IR: ν = 3410, 2987, 2952, 1732, 1603, 1322, 1127, 1067, 747, 696 cm^{-1}.
^{1}H NMR (400 MHz, Chloroform-*d*) δ 7.38 (d, *J* = 8.2 Hz, 2H), 7.27 (d, *J* = 8.2 Hz, 2H), 7.27 – 7.12 (m, 5H), 6.97 – 6.87 (m, 2H), 6.66 – 6.56 (m, 1H), 6.31 – 6.23 (m, 2H), 5.03 (d, *J* = 1.5 Hz, 2H), 4.13 (s, 1H), 2.99 – 2.76 (m, 2H), 1.58 (s, 3H).
^{19}F NMR (376 MHz, Chloroform-*d*) δ -62.96 (s, 3F), -92.44 (s, 1F), -92.47 (s, 1F).
^{13}C NMR (101 MHz, Chloroform-*d*) δ 174.4, 144.2, 140. 6 (t, *J* = 26.7 Hz), 135.1, 131.7 (q, *J* = 32.6 Hz), 128.9, 128.6, 128.5, 128.4, 125.4 (t, *J* = 6.5 Hz), 125.1 (q, *J* = 3.8 Hz), 123.6 (q, *J* = 273.7 Hz), 121.5 (t, *J* = 245.4 Hz), 118.6, 115.5, 67.8, 58.5, 43.5 (t, *J* = 26.1 Hz), 24.8.
HRMS (ESI) calcd. for $C_{25}H_{23}F_5NO_2$ $[M+H]^+$: 464.1643, found: 464.1638.

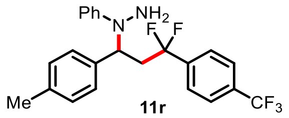

11r

1-(3,3-difluoro-1-(p-tolyl)-3-(4-(trifluoromethyl)phenyl)propyl)-1-phenylhydrazine (11r)

According to the general procedure E, the crude product is purified by flash chromatography on silica gel (PE/EA = 30:1). (0.2 mmol scale, 35.4 mg, 42%). Rf= 0.6 (PE/EA = 5:1). Pale yellow oil.

IR: ν = 3344, 3022, 2927, 1596, 1497, 1323, 1128, 1068, 845, 749 cm^{-1}.

^{1}H NMR (400 MHz, Chloroform-*d*) δ 7.71 – 7.41 (m, 4H), 7.20 (t, *J* = 7.8 Hz, 2H), 7.12 (d, *J* = 7.7 Hz, 2H), 7.06 (d, *J* = 7.7 Hz, 2H), 6.90 – 6.60 (m, 3H), 5.21 – 5.02 (m, 1H), 3.53 – 3.30 (m, 1H), 3.25 – 2.48 (m, 3H), 2.29 (s, 3H).

^{19}F NMR (376 MHz, Chloroform-*d*) δ -62.86 (s, 3F), -91.46 (d, *J* = 249.7 Hz), -96.41 (d, *J* = 249.6 Hz).

^{13}C NMR (101 MHz, Chloroform-*d*) δ 150.7, 140.8 (t, *J* = 26.9 Hz), 137.3, 135.4, 131.6 (q, *J* = 33.3 Hz), 129.1, 129.0, 127.4, 125.5 (t, *J* = 6.3 Hz), 125.3 – 124.9 (m), 123.7 (q, *J* = 273.7 Hz), 122.1 (t, *J* = 244.4 Hz), 118.5, 112.9, 58.2 (t, *J* = 4.7 Hz), 40.9 (t, *J* = 26.8 Hz), 21.0.

HRMS (ESI) calcd. for $C_{23}H_{22}F_5N_2$ $[M+H]^+$: 421.1698, found: 421.1690.

6. Downstream Transformation of Products

6.1 Gram-scale reaction

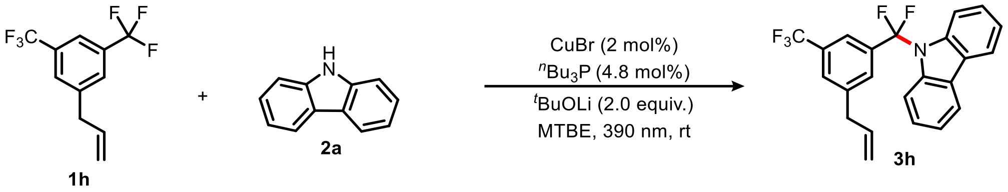

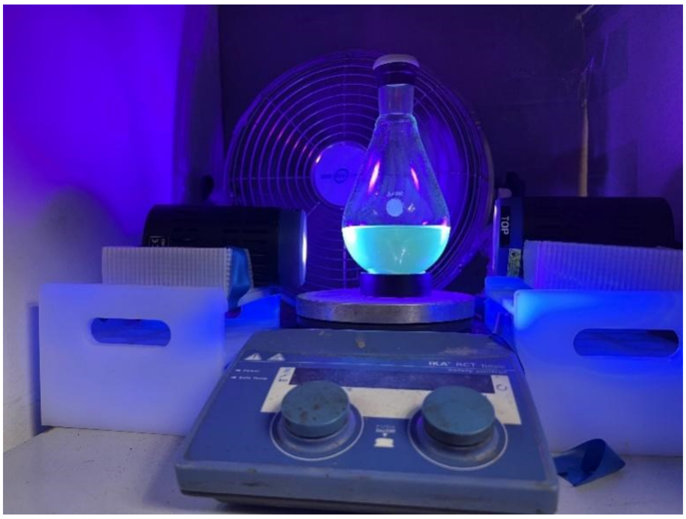

Supplementary Figure 3. Gram-scale reaction

In a N_2-filled glove-box, an oven-dried 250 mL flask equipped with a magnetic stir bar is charged with CuBr (14.3 mg, 0.1 mmol, 2.0 mol%), carbazole **2a** (835.0 mg, 5.0 mmol, 1.0 equiv.), ^{t}BuOLi (800.0 mg, 10 mmol, 2.0 equiv.), MTBE (100.0 mL) and ^{n}Bu$_3$P (60.0 µL, 0.24 mmol, 4.8 mol%). Then the reaction mixture is allowed to stir at rt for 30 min. Subsequently, the trifluoromethylated arene **1h** (6.35g, 25.0 mmol, 5.0 equiv.) is added. Then the flask is sealed, removed from the glove-box and is irradiated under 390 nm LED (40 W × 2) for 12 h at 27 °C with vigorous stirring. When TLC shows that **2a** is disappeared (~12 h), the mixture is then concentrated in vacuo. The crude product is purified by flash column chromatography on neutral Al_2O_3 with petroleum ether, ethyl acetate and triethylamine as eluent to afford **3h** (52%, 1.04 g).

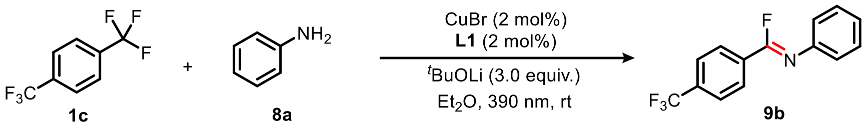

In a N_2-filled glove-box, an oven-dried 250 mL flask equipped with a magnetic stir bar is charged with CuBr (28.7 mg, 0.2 mmol, 2.0 mol %), **L1** (140.0 mg, 0.2 mmol, 2.0 mol %), ^{t}BuOLi (2.4 g, 30.0 mmol, 3.0 equiv.) and Et_2O (100.0 mL). Then the reaction mixture is allowed to stir at rt for 30 min. **1c** (4.65 mL, 30.0 mmol, 3.0 equiv.) and **8a** (0.91 mL, 10.0 mmol, 1.0 equiv.) are added sequentially. Then the flask is sealed, removed from the glove-box and is irradiated under 390 nm LED (40 W × 2) for 2 h at 27 °C with vigorous stirring. When TLC showed that **8a** is disappeared (~20 h), the mixture is then concentrated in vacuo. The crude product is purified by flash column chromatography on neutral Al_2O_3 with petroleum ether as eluent to afford **9b** (57%, 1.51 g).

6.2 Downstream transformations

Remote migratory hydro-arylation of 3h

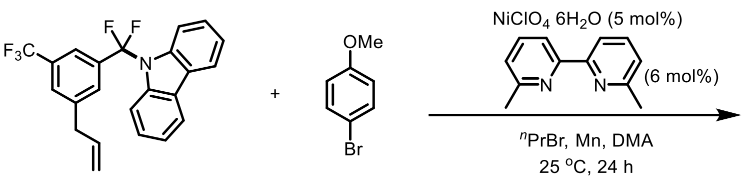

3h **12**, 68%, rr > 20:1

Following literature procedure[11], in a N_2-filled glove-box, an oven-dried vial (8 mL) equipped with a magnetic stir bar is charged with $Ni(ClO_4)_2$ $6H_2O$ (2.7 mg, 5.0 mol%) and 6,6'-dimethyl-2,2'-bipyridine (1.7 mg, 6.0 mol%). Anhydrous DMA (1.0 mL) were added. The mixture is stirred for 10 min before aryl bromide (37.5 μL, 0.30 mmol, 2.0 equiv.), **3h** (60 mg, 0.15 mmol, 1.0 equiv.), ^{n}PrBr (20.0 μL, 0.225 mmol, 1.5 equiv.) and Mn (20.6 mg, 0.375 mmol, 2.5 equiv.) were added to the resulting mixture in this order. The vial is sealed, removed from the glove box and stirred at rt (25 °C) for 4 h before the ^{n}PrBr (14.0 μL, 0.15 mmol, 1.0 equiv.) is added by syringe. Then the reaction mixture is stirred at this temperature for up to 24 h. The crude product is purified by flash column chromatography on neutral Al_2O_3 with petroleum ether, ethyl acetate and triethylamine as eluent to afford **12** (52.1 mg, 68%).

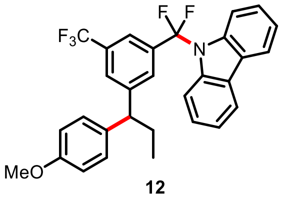

12

9-(difluoro(3-(1-(4-methoxyphenyl)propyl)-5-(trifluoromethyl)phenyl)methyl)-9H-carbazole (12)

The crude product is purified by flash chromatography on Al_2O_3 (PE : NEt_3=100: 1). (0.15 mmol scale, 52.1 mg, 68%). Rf= 0.6 (PE/EA = 10:1). Light yellow oil.

IR: ν = 2963, 2933, 1492, 1446, 1300, 1236, 1161, 1127, 1111, 748 cm^{-1}.

^{1}H NMR (500 MHz, Chloroform-*d*) δ 8.10 – 8.04 (m, 2H), 7.72 (s, 1H), 7.60 (s, 1H), 7.38 (s, 1H), 7.36 – 7.27 (m, 6H), 6.95 – 6.88 (m, 2H), 6.78 – 6.71 (m, 2H), 3.78 (s, 3H), 3.74 (t, *J* = 7.8 Hz, 1H), 1.90 (qt, *J* = 13.6, 7.4 Hz, 2H), 0.78 (t, *J* = 7.3 Hz, 3H).

^{19}F NMR (471 MHz, Chloroform-*d*) δ 62.55 (s, 3F), -68.32 (s, 2F).

^{13}C NMR (126 MHz, Chloroform-*d*) δ 158.2, 148.1, 138.6, 135.4 (t, *J* = 33.2 Hz), 135.0, 131.7 (q, *J* = 32.7 Hz), 128.8, 128.5, 127.8 – 127.3 (m), 126.7, 125.0, 123.5 (q, *J* = 273.4 Hz), 121.7, 120.9 (q, *J* = 3.9 Hz), 120.3 (t, *J* = 250.9 Hz), 120.1, 114.0, 113.3 (t, *J* = 5.4 Hz), 55.2, 51.9, 28.4, 12.4.

HRMS (FI) calcd. for $C_{30}H_{24}ONF_5$ $[M]^+$: 509.1773, found: 509.1771.

Epoxidation of 3h

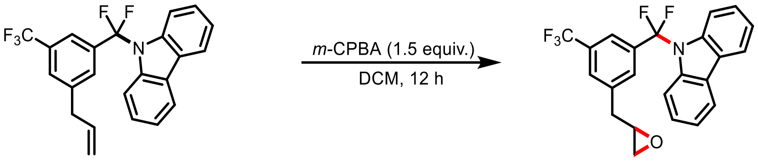

3h **13**, 88%

In a 25 mL flask equipped with a magnetic stir bar is charged with **3h** (80.0 mg, 0.2 mmol, 1.0 equiv.) and *m*-CPBA (61.0 mg, 0.3 mmol, 1.5 equiv.). Anhydrous DCM (2.0 mL) were added. The mixture is stirred at rt (25 °C) for 12 h. The crude product is purified by flash column chromatography on neutral Al_2O_3 with petroleum ether, ethyl acetate and triethylamine as eluent to afford **13** (71.0 mg, 88%).

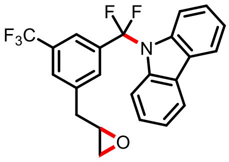

13

9-(difluoro(3-(oxiran-2-ylmethyl)-5-(trifluoromethyl)phenyl)methyl)-9H-carbazole (13)

The crude product is purified by flash chromatography on Al_2O_3 (PE : EA : NEt_3=100: 10 : 1). (0.2 mmol scale, 71.0 mg, 88%). Rf= 0.7 (PE/EA = 5:1). Light yellow oil.

IR: ν = 3053, 1447, 1238, 1162, 1126, 797, 749, 723, 703, 677 cm^{-1}.
^{1}H NMR (500 MHz, Chloroform-*d*) δ 8.08 (dt, *J* = 7.6, 1.2 Hz, 2H), 7.81 (s, 1H), 7.71 (s, 1H), 7.50 (s, 1H), 7.42 – 7.37 (m, 4H), 7.37 – 7.32 (m, 2H), 3.08 – 3.03 (m, 1H), 2.94 (dd, *J* = 14.9, 4.4 Hz, 1H), 2.82 (dd, *J* = 14.8, 6.2 Hz, 1H), 2.74 – 2.69 (m, 1H), 2.38 (dd, *J* = 4.8, 2.6 Hz, 1H).
^{19}F NMR (471 MHz, Chloroform-*d*) δ -62.64 (s, 3F), -68.47 (s, 1F), -68.48 (s, 1F).
^{13}C NMR (126 MHz, Chloroform-*d*) δ 139.6, 138.5, 135.8 (t, *J* = 33.5 Hz), 131.9 (q, *J* = 33.0 Hz), 130.0 (t, *J* = 4.3 Hz), 128.8 (dq, *J* = 3.6, 1.9 Hz), 126.7, 125.0, 123.4 (q, *J* = 273.4 Hz), 121.8, 121.7 – 121.4 (m), 120.2, 120.1 (t, *J* = 252.0 Hz), 113.1 (t, *J* = 5.6 Hz), 51.3, 46.3, 38.0.
HRMS (FI) calcd. for $C_{23}H_{16}ONF_5$ $[M]^+$: 417.1147, found: 417.1143.

Nucleophilic substitution of 9b

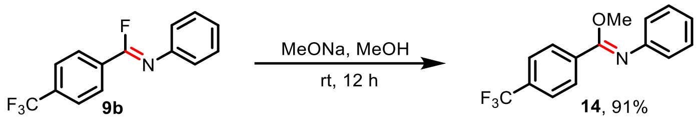

Following literature procedure[12], in a 25 mL flask equipped with a magnetic stir bar is charged with **9b** (26.7 mg, 0.1 mmol, 1.0 equiv.) and it is dissolved in anhydrous MeOH (2.0 mL). MeONa solution (5.4 M in MeOH) is added (56 µL, 0.3 mmol, 3.0 equiv.). The mixture is stirred at rt (25 °C) for 12 h. The crude product is purified by flash column chromatography on neutral Al_2O_3 with petroleum ether as eluent to afford **14** (25.5 mg, 91%).

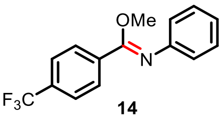

14

methyl-*N*-phenyl-4-(trifluoromethyl)benzimidate (14)

The crude product is purified by flash chromatography on Al_2O_3 (PE). (0.1 mmol scale, 25.5 mg, 91%). Rf= 0.7 (PE). Colorless oil.

IR: ν = 2945, 2855, 1662, 1324, 1169, 1111, 1068, 1018, 849, 720 cm^{-1}.
^{1}H NMR (500 MHz, Chloroform-*d*) δ 7.51 – 7.46 (m, 2H), 7.42 (d, *J* = 8.2 Hz, 2H), 7.23 – 7.15 (m, 2H), 7.02 – 6.95 (m, 1H), 6.75 – 6.68 (m, 2H), 3.99 (s, 3H).
^{19}F NMR (471 MHz, Chloroform-*d*) δ -63.02 (s, 3F).
^{13}C NMR (126 MHz, Chloroform-*d*) δ 157.6, 147.7, 134.7, 131. 6 (q, *J* = 32.8 Hz), 129.7, 129.1, 124.9 (q, *J* = 3.7 Hz), 123.6 (q, *J* = 273.4 Hz), 123.0, 121.5, 54.2.
HRMS (ESI) calcd. for $C_{15}H_{13}F_3NO$ $[M+H]^+$: 280.0944, found: 280.0935.

Azidation of 9b

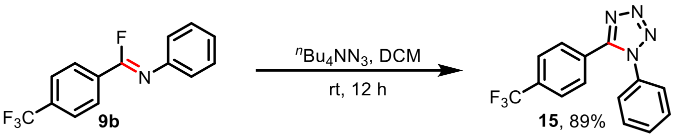

Following literature procedure[12], in a 25 mL flask equipped with a magnetic stir bar is charged with **9b** (53.4 mg, 0.2 mmol, 1.0 equiv.) and it is dissolved in anhydrous DCM (2.0 mL). $^{n}Bu_4NN_3$ (113.8 mg, 0.4 mmol, 2.0 equiv.) is added. The mixture is stirred at rt (25 °C) for 12 h. The crude product is purified by flash column chromatography on neutral Al_2O_3 with petroleum ether and ethyl acetate as eluent to afford **15** (51.4 mg, 89%).

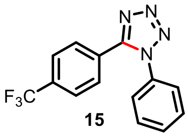

15

1-phenyl-5-(4-(trifluoromethyl)phenyl)-1H-tetrazole (15)

The crude product is purified by flash chromatography on Al_2O_3 (PE/EA = 20 :1). (0.2 mmol scale, 51.4 mg, 89%). Rf= 0.3 (PE/EA = 5:1). Colorless crystal.

IR: ν = 3066, 2928, 1479, 1322, 1169, 1125, 1067, 848, 764,691 cm^{-1}.
^{1}H NMR (500 MHz, Chloroform-*d*) δ 7.73 – 7.64 (m, 4H), 7.62 – 7.53 (m, 3H), 7.42 – 7.37 (m, 2H).
^{19}F NMR (471 MHz, Chloroform-*d*) δ -63.20 (s, 3F).
^{13}C NMR (126 MHz, Chloroform-*d*) δ 152.4, 134.1, 133.0 (q, *J* = 33.1 Hz), 130.8, 130.1, 129.3, 127.1, 125.9 (q, *J* = 3.9 Hz), 125.3, 123.4 (q, *J* = 273.4 Hz),.
HRMS (ESI) calcd. for $C_{15}H_{13}F_3NO$ $[M+H]^+$: 291.0852, found: 291.0843.

6.3 Synthesis of Carvedilol derivative

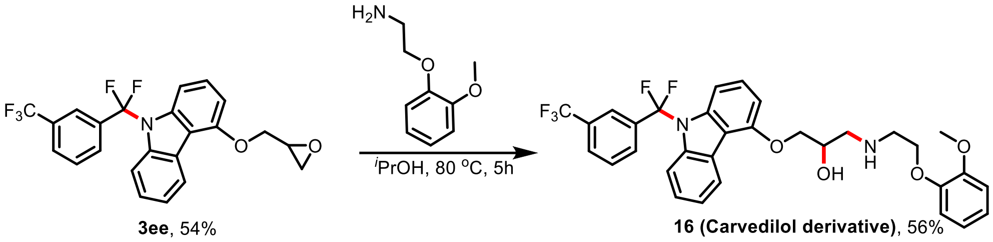

In a 25 mL flask equipped with a magnetic stir bar is charged with **3ee** (43.3 mg, 0.1 mmol, 1.0 equiv.) and amine (22.6 μL, 0.15 mmol, 1.5 equiv.). Anhydrous *i*PrOH (2.0 mL) were added. The mixture is stirred at 80 °C for 5 h. The crude product is purified by flash column chromatography on neutral Al_2O_3 with DCM, MeOH and NEt_3 as eluent to afford **16** (33.6 mg, 56%).

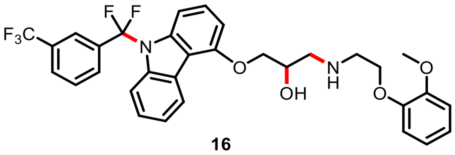

16

1-((9-(difluoro(3-(trifluoromethyl)phenyl)methyl)-9H-carbazol-4-yl)oxy)-3-((2-(2-methoxyphenoxy)ethyl)amino)propan-2-ol (16)

The crude product is purified by flash chromatography on Al_2O_3 (DCM : MeOH : NEt_3=100: 10 : 1). (0.1 mmol scale, 33.6 mg, 56%). Rf= 0.2 (DCM : MeOH = 10 : 1). Light yellow oil.

IR: ν = 3544, 3311, 2921 1450, 1271, 1253, 1153, 1120, 1044, 741 cm^{-1}.
^{1}H NMR (500 MHz, Chloroform-*d*) δ 8.38 – 8.30 (m, 1H), 7.91 (s, 1H), 7.78 – 7.69 (m, 1H), 7.49 – 7.40 (m, 2H), 7.41 – 7.35 (m, 1H), 7.35 – 7.23 (m, 3H), 7.03 – 6.97 (m, 1H), 6.96 – 6.82 (m, 4H), 6.75 (d, *J* = 8.1 Hz, 1H), 4.32 – 4.26 (m, 2H), 4.25 – 4.19 (m, 1H), 4.14 (t, *J* = 5.1 Hz, 2H), 3.80 (s, 3H), 3.14 – 3.07 (m, 3H), 2.98 (dd, *J* = 12.3, 7.5 Hz, 1H).
^{19}F NMR (471 MHz, Chloroform-*d*) δ -62.75 (s, 3F), -68.45 (s, 2F).
^{13}C NMR (126 MHz, Chloroform-*d*) δ 155.0, 149.7, 148.1, 139.8, 137.7, 135.5 (t, *J* = 34.0 Hz), 131.6 (q, *J* = 33.1 Hz), 129.6, 129.4 – 129.2 (m), 128.3 – 127.9 (m), 127.4, 125.8, 124.2, 123.4 (q, *J* = 273.4 Hz), 123.3, 123.1 – 122.9 (m), 121.9, 121.6, 120.8, 120.2 (t, *J* = 251.7 Hz), 114.2, 114.1, 112.4 (t, *J* = 5.8 Hz), 111.8, 106.0 (t, *J* = 5.8 Hz), 103.6, 70.5, 68.8, 68.3, 55.7, 51.9, 48.7.
HRMS (DART) calcd. for $C_{32}H_{30}F_5N_2O_4$ $[M]^+$: 601.2120, found: 601.2112.

7. Mechanistic Studies

7.1 Radical trapping experiments

Radical trapping experiment with TEMPO

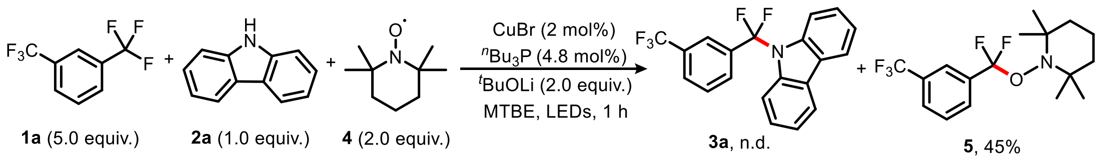

In a N_2-filled glove-box, an oven-dried vial (8 mL) equipped with a magnetic stir bar was charged with CuBr (0.29 mg, 0.002 mmol, 2.0 mol%), carbazole **2a** (16.7 mg, 0.1 mmol, 1.0 equiv.), ^{t}BuOLi (16.0 mg, 0.2 mmol, 2.0 equiv.), TEMPO (31.2 mg, 0.2 mmol, 2.0 equiv.), MTBE (4.0 mL) and ^{n}Bu$_3$P (1.2 μL, 0.0048 mmol, 4.8 mol%). The reaction mixture was allowed to stir at rt for 30 min. Subsequently, **1a** (85 μL, 0.5 mmol, 5.0 equiv.) was added. Then the vial was sealed with a rubber cap, removed from the glove-box and was irradiated under 390 nm LED (40 W × 2) for 1 h at 27 °C with vigorous stirring. After the reaction finished, the reaction mixture was analyzed by GC-MS. **3a** was not detected, and the TEMPO-trapped difluorobenzylic radical adduct **5** was obtained with 45% isolated yield.

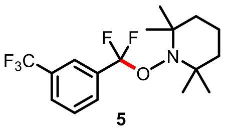

5

1-(difluoro(3-(trifluoromethyl)phenyl)methoxy)-2,2,6,6-tetramethylpiperidine (5)

The crude product was purified by flash chromatography on Al_2O_3 (PE/NEt$_3$ = 100:1). (0.2 mmol scale, 31.6 mg, 45%). Rf= 0.65 (PE/EA = 20:1). Colorless oil.

IR: ν = 2931, 1341, 1302, 1170, 1126, 1080, 1054, 906, 809, 701 cm^{-1}.
^{1}H NMR (500 MHz, Chloroform-*d*) δ 7.89 (s, 1H), 7.84 (d, *J* = 7.9 Hz, 1H), 7.74 (d, *J* = 7.8 Hz, 1H), 7.58 (t, *J* = 7.8 Hz, 1H), 1.71 – 1.62 (m, 3H), 1.60 – 1.54 (m, 2H), 1.44 – 1.37 (m, 1H), 1.26 (t, *J* = 3.3 Hz, 6H), 1.20 (s, 6H).
^{19}F NMR (471 MHz, Chloroform-*d*) δ -62.79 (s, 3F), -63.86 (s, 2F).
^{13}C NMR (126 MHz, Chloroform-*d*) δ 135.9 (t, *J* = 33.9 Hz), 131.0 (q, *J* = 32.7 Hz), 129.1, 128.9 – 128.8 (m), 127.2 (q, *J* = 3.8 Hz), 123.7 (q, *J* = 273.4 Hz), 122.6 – 122.3 (m), 121.3 (t, *J* = 263.3 Hz), 61.2, 40.3, 34.2 (t, *J* = 5.7 Hz), 21.0, 17.0.
HRMS (FI) calcd. for $C_{17}H_{22}ONF_5$ [M]$^+$: 351,1616, found: 351.1618.

Radical clock experiment

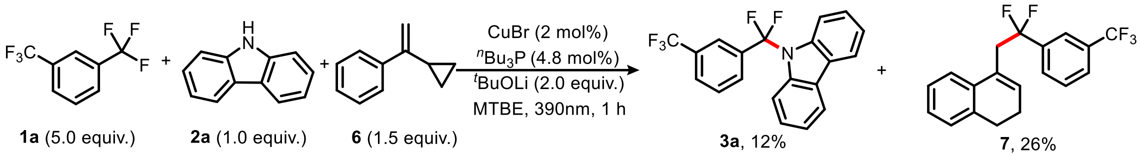

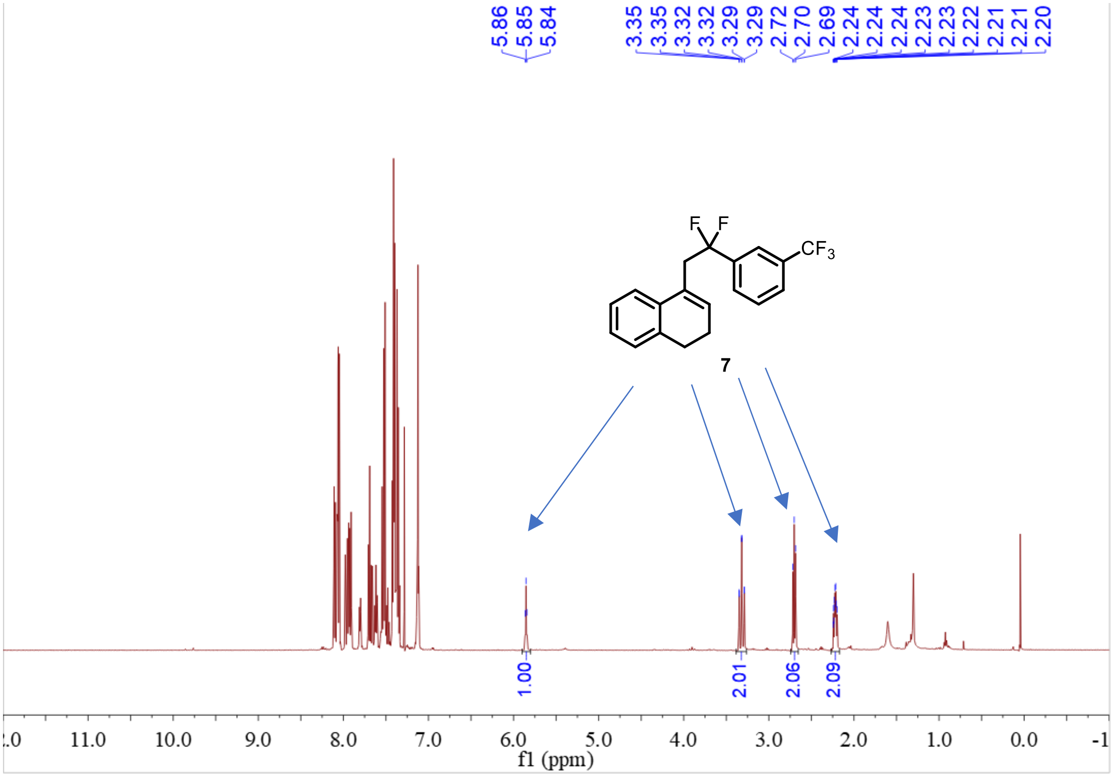

In a N_2-filled glove-box, an oven-dried vial (8 mL) equipped with a magnetic stir bar was charged with CuBr (0.29 mg, 0.002 mmol, 2.0 mol%), carbazole **2a** (16.7 mg, 0.1 mmol, 1.0 equiv.), ^{t}BuOLi (16.0 mg, 0.2 mmol, 2.0 equiv.), MTBE (4.0 mL) and ^{n}Bu$_3$P (1.2 µL, 0.0048 mmol, 4.8 mol%). Then the reaction mixture was allowed to stir at rt for 30 min. Subsequently, **1a** (85 µL, 0.5 mmol, 5.0 equiv.) and (1-cyclopropylvinyl)benzene **6** (21.6 mg, 0.15 mmol, 1.5 equiv.) were added sequentially. Then the vial was sealed with a rubber cap, removed from the glove-box and was irradiated under 390 nm LED (40 W × 2) for 1 h at 27 °C with vigorous stirring. After the reaction finished, the reaction mixture was analyzed by **^{1}H NMR**.

The yield of **3a** was reduced to 12%, and the radical ring-opening product **7** was obtained with 26% NMR yield.

7.2 The emission spectra for the light source

The UV-vis experiments were performed on F-7000 FL Spectrophotometer (Hitachi, Japan) with a quartz cuvette (10 mm path length).

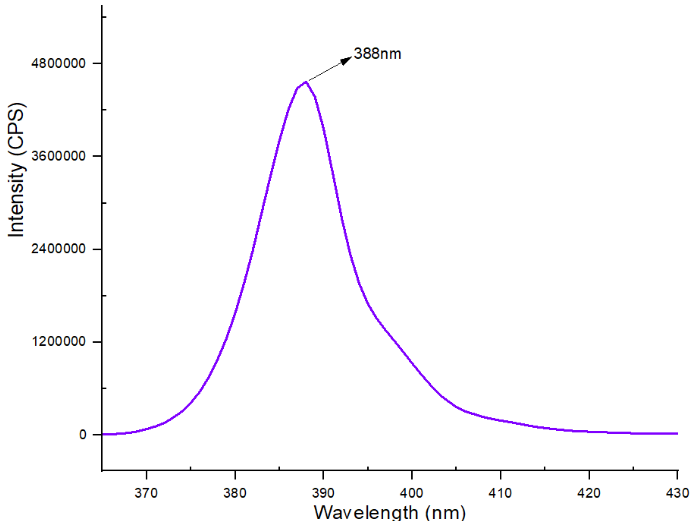

Supplementary Figure 4. Kessil PR160L-390nm LED emission spectrum

7.3 UV-vis absorption spectroscopic measurement

The UV-vis experiments were performed on F-7000 FL Spectrophotometer (Hitachi, Japan) with a quartz cuvette (10 mm path length). All of the samples are measured in MTBE with 250 µM concentration.

We found that the mixture of carbazole **2a**, CuBr, $^{n}Bu_3P$ and $^{t}BuOLi$ have obvious absorption in the emission wavelength range of the light source.

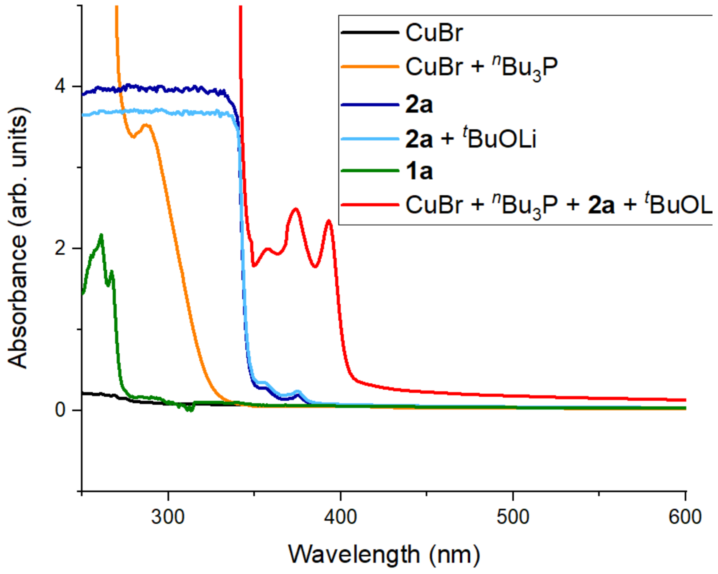

Supplementary Figure 5. UV-vis spectroscopic absorption

7.4 The total reaction profile

In a N_2-filled glove-box, an oven-dried vial (8 mL) equipped with a magnetic stir bar was charged with CuBr (0.29 mg, 0.002 mmol, 2.0 mol%), carbazole **2a** (16.7 mg, 0.1 mmol, 1.0 equiv.), ^{t}BuOLi (16.0 mg, 0.2 mmol, 2.0 equiv.), MTBE (4.0 mL) and ^{n}Bu$_3$P (1.2 µL, 0.0048 mmol, 4.8 mol%). Then the mixture was allowed to stir at rt for 30 min. **1a** (85µL, 0.5 mmol, 5.0 equiv.) and dodecane (23 µL, 0.1 mmol, 1.0 equiv.) were added sequentially. Then the vial was sealed with a rubber cap, removed from the glove-box and was irradiated under 390 nm LED (40 W × 2) for 1 h at 27 °C with vigorous stirring. At 1, 2, 3, 4, 5, 6, 7, 8, 9, 10, 11, 13, 14, 16, 18, 20, 25, 30, 35, 40, 50, 60, 120, 180, 240 min, 20 µL the reaction mixture was carefully taken out by micro-syringe into 2.0 mL vial. Then 1.0 ml EA was added into the vial. The reaction mixture was analyzed by GC after filtration.

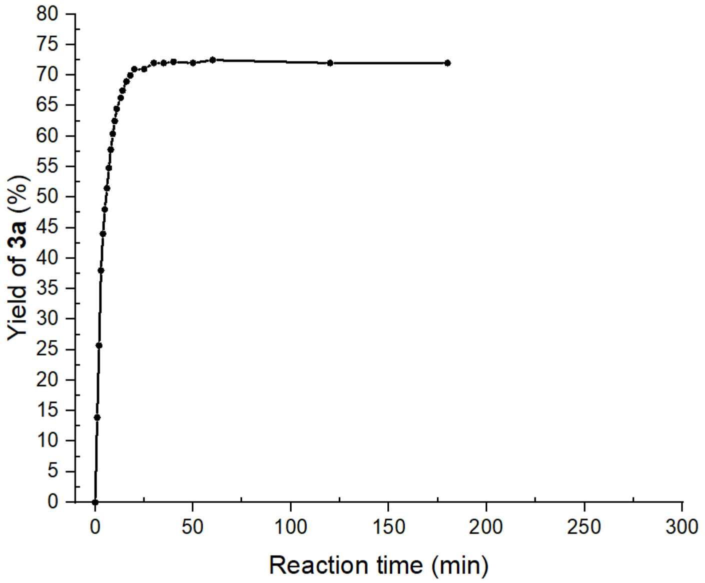

Supplementary Figure 6. The total reaction profile

7.5 Kinetic experiments

Dependence of the reaction rate on concentration of [CuBr + ^{n}Bu$_3$P]

In a N_2-filled glove-box, an oven-dried vial (8 mL) equipped with a magnetic stir bar was charged with CuBr (2.0/4.0/6.0/8.0 mol%), carbazole **2a** (16.7mg, 0.1 mmol, 1.0 equiv.), ^{t}BuOLi (16.0 mg, 0.2 mmol, 2.0 equiv.), MTBE (4.0 mL) and ^{n}Bu$_3$P (4.8/9.6/14.4/19.2 mol%). The mixture was allowed to stir at rt for 30 min. **1a** (85µL, 0.5 mmol, 5.0 equiv.) and dodecane (23 µL, 0.1 mmol, 1.0 equiv.) were added sequentially. Then the vial was sealed with a rubber cap, removed from the glove-box and was irradiated under 390 nm LED (40 W × 2) for 1 h at 27 °C with stirring. At 10, 20, 30, 40, 50, 60, 70, 80, 90s, 20 µL the reaction mixture was carefully taken out by micro-syringe into 2.0 mL vial. Then 1.0 ml EA was added into the vial. The reaction mixture was analyzed by GC after filtration.

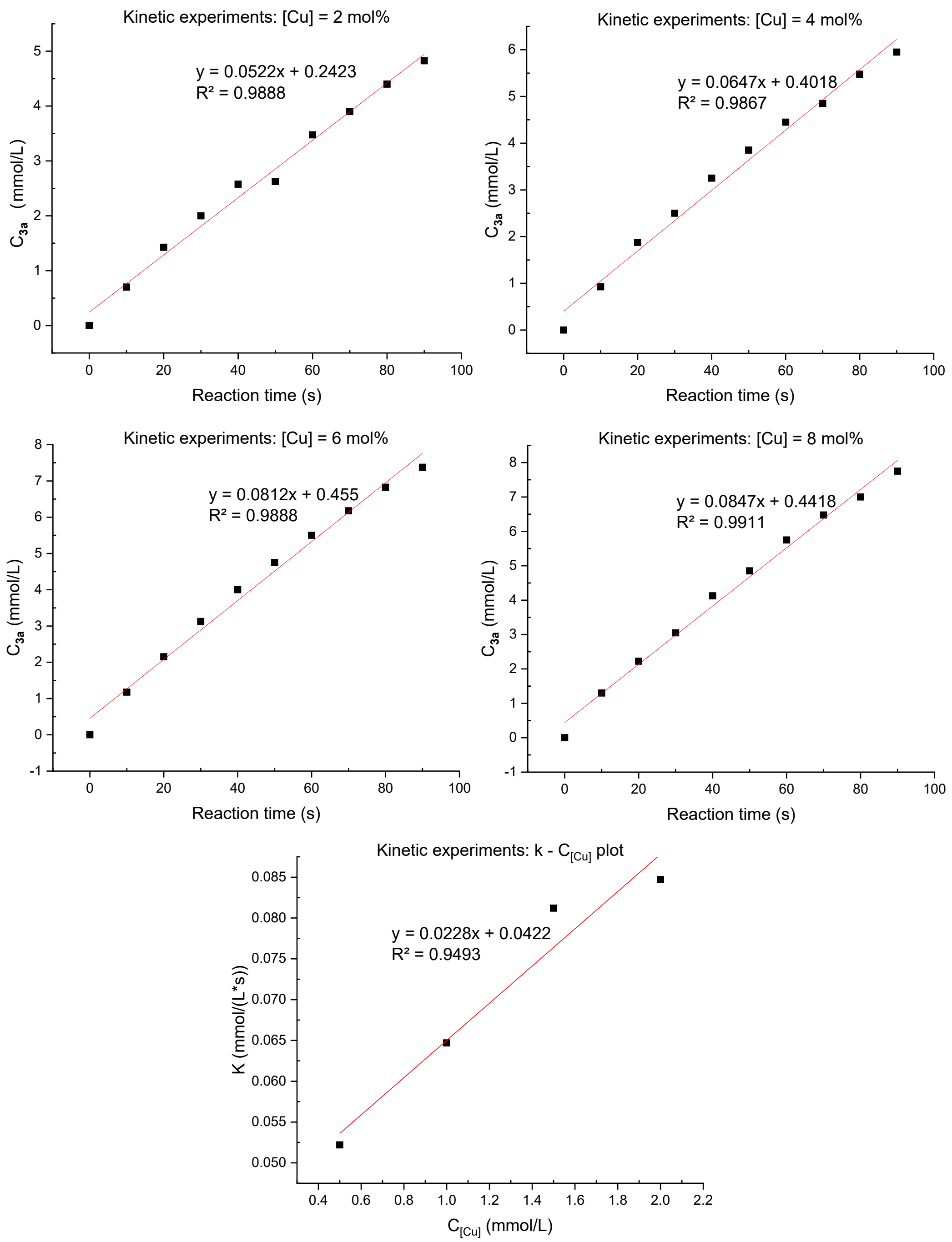

Supplementary Figure 7. Dependence of the reaction rate on concentration of [CuBr + ^{n}Bu$_3$P]

Dependence of the reaction rate on concentration of 1a

In a N_2-filled glove-box, an oven-dried vial (8 mL) equipped with a magnetic stir bar was charged with CuBr (0.29 mg, 0.002 mmol, 2.0 mol%), carbazole **2a** (16.7mg, 0.1 mmol, 1.0 equiv.), ^{t}BuOLi (16.0 mg, 0.2 mmol, 2.0 equiv.), MTBE (4.0 mL) and ^{n}Bu$_3$P (1.2 μL, 0.0048 mmol, 4.8 mol%). The mixture was allowed to stir at rt for 30 min. **1a** (0.25/0.50/0.75/1.00 mmol) and dodecane (23 μL, 0.1 mmol, 1.0 equiv.) were added sequentially. Then the vial was sealed with a rubber cap, removed from the glove-box and was irradiated under 390 nm LED (40 W × 2) for 1 h at 27 °C with stirring. At 10, 20, 30, 40, 50, 60, 70, 80, 90s, 20 μL the reaction mixture was carefully taken out by micro-

syringe into 2.0 mL vial. Then 1.0 ml EA was added into the vial. The reaction mixture was analyzed by GC after filtration.

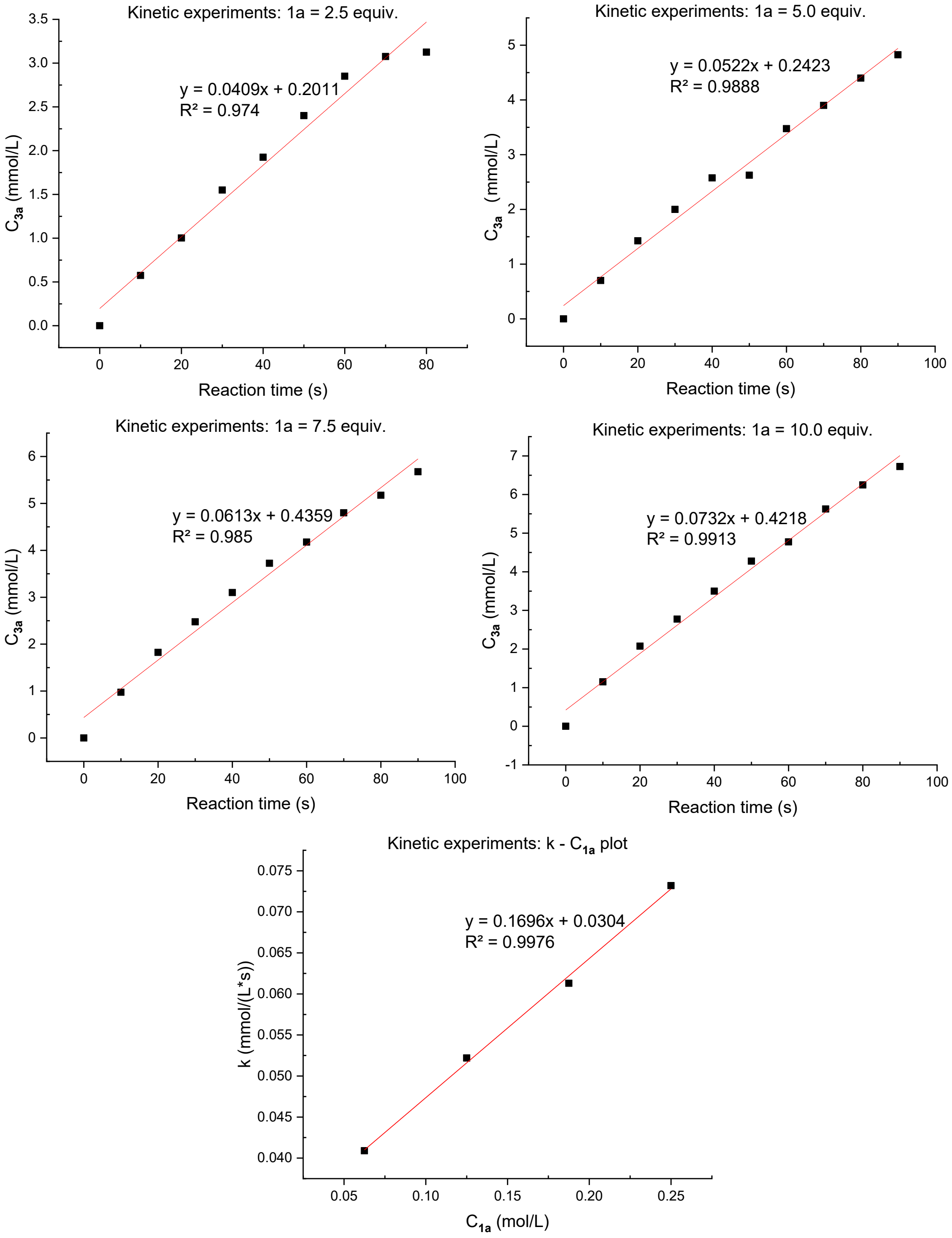

Supplementary Figure 8. Dependence of the reaction rate on concentration of **1a**

Dependence of the reaction rate on concentration of 2a

In a N_2-filled glove-box, an oven-dried vial (8 mL) equipped with a magnetic stir bar was charged with CuBr (0.29 mg, 0.002 mmol, 2.0 mol%), carbazole **2a** (0.05/0.10/0.15/0.20 mmol), ^{t}BuOLi (16.0 mg, 0.2 mmol, 2.0 equiv.), MTBE (4.0 mL) and ^{n}Bu$_3$P (1.2 µL, 0.0048 mmol, 4.8 mol%). The mixture was allowed to stir at rt for 30 min. **1a** (85µL, 0.5 mmol, 5.0 equiv.) and dodecane (23 µL, 0.1 mmol, 1.0 equiv.) were added sequentially. Then the vial was sealed with a rubber cap, removed

from the glove-box and was irradiated under 390 nm LED (40 W × 2) for 1 h at 27 °C with stirring. At 10, 20, 30, 40, 50, 60, 70, 80, 90s, 20 μL the reaction mixture was carefully taken out by micro-syringe into 2.0 mL vial. Then 1.0 ml EA was added into the vial. The reaction mixture was analyzed by GC after filtration.

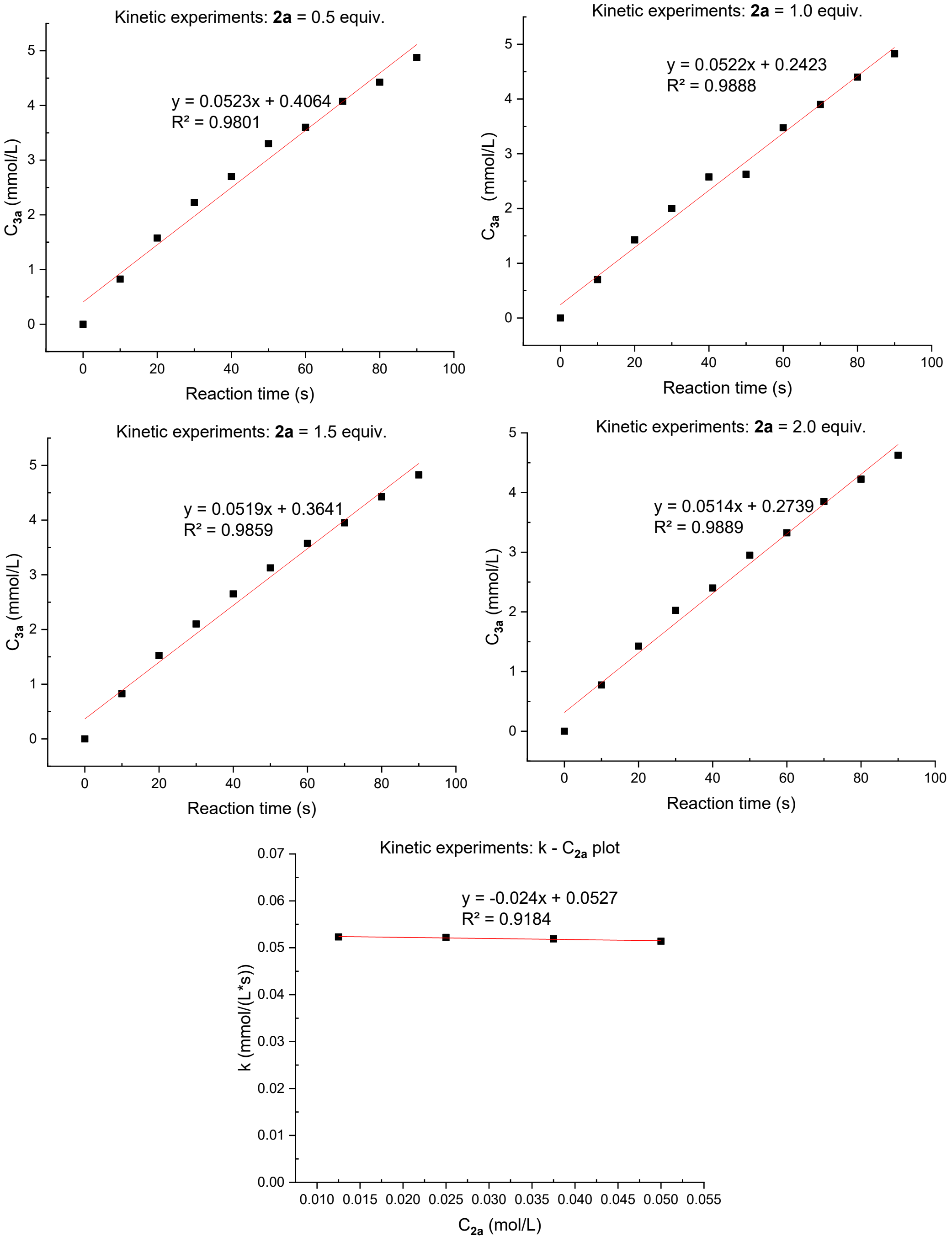

Supplementary Figure 9. Dependence of the reaction rate on concentration of **2a**

The plots of *K*obs suggest a zero-order kinetic dependence in **2a**, and a first-order kinetic dependence in [CuBr + ^{n}Bu$_3$P] and **1a**. The activation of C-F bond probably is the rate determining step of the reaction.

7.6 Turn on/off profile experiment

In a N_2-filled glove-box, an oven-dried vial (8 mL) equipped with a magnetic stir bar was charged with CuBr (0.29 mg, 0.002 mmol, 2.0 mol%), carbazole **2a** (16.7mg, 0.1 mmol, 1.0 equiv.), *ᵗ*BuOLi (16.0 mg, 0.2 mmol, 2.0 equiv.), MTBE (4.0 mL) and *ⁿ*Bu_3P (1.2 µL, 0.0048 mmol, 4.8 mol%). The mixture was allowed to stir at rt for 30 min. **1a** (85µL, 0.5 mmol, 5.0 equiv.) and dodecane (23 µL, 0.1 mmol, 1.0 equiv.) were added sequentially. Then the vial was sealed with a rubber cap, removed from the glove-box and was irradiated under 390 nm LED (40 W × 2) for 1 h at 27 °C with stirring. The process of photocatalytic reaction with and without light was monitored by GC. It was found that the formation of **3a** needed continuous irradiation of light.

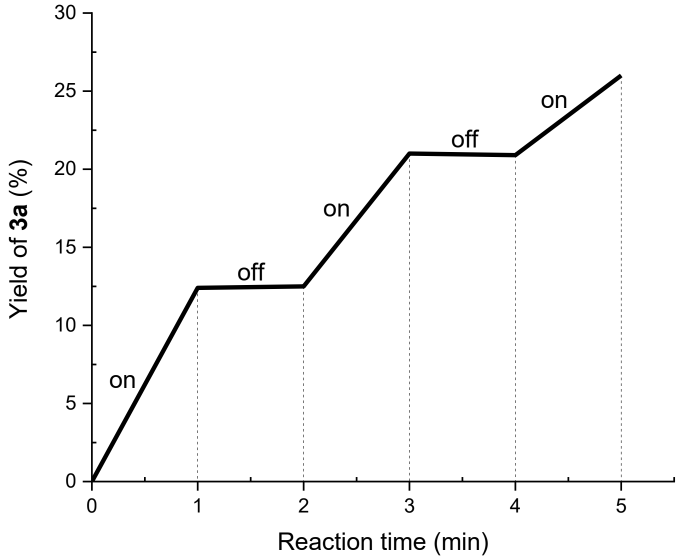

Supplementary Figure 10. Turn on/off profile experiment

7.7 Luminescence quenching experiment

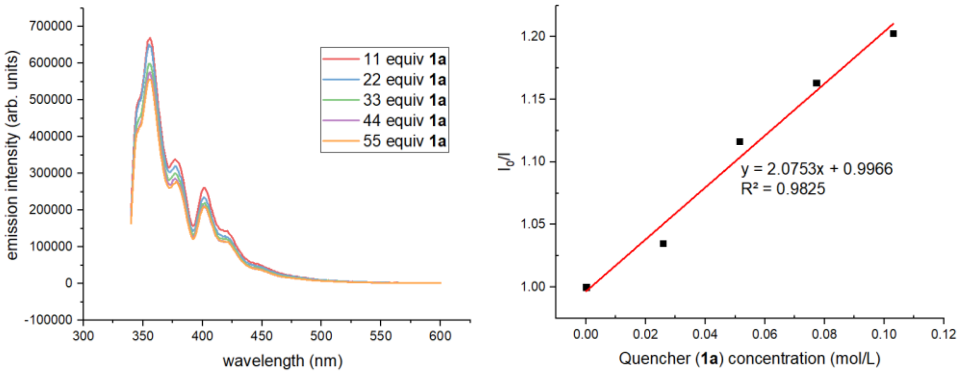

Supplementary Figure 11. Luminescence quenching experiment

The luminescence quenching experiment was taken using a F-7000 FL Spectrophotometer (Hitachi, Japan). The experiments were carried out in 2 x 10^{-3} mol/L of [CuBr+*ⁿ*Bu_3P+**2a**+*ᵗ*BuOLi] in MTBE at 25 °C. The excitation wavelength was 320 nm and the emission intensity was collected at 356

nm. The concentrations of quenchers (**1a**) in MTBE were 22, 44, 66, 88, 110 mM. Based on the above data, photoexcited [CuBr+^{n}Bu$_3$P+**2a**+^{t}BuOLi] can be quenched by **1a**.

7.8 Quantum yield measurement

The quantum yield (ϕ) was determined by the known ferrioxalate actinometry method. A ferrioxalate actinometry solution was prepared by following the Hammond variation of the Hatchard and Parker procedure outlined in Handbook of Photochemistry. The actinometry solutions (1mL) were irradiated with two 40 W 390nm LEDs for specified time intervals (15 s, 30 s, 45 s, 60 s, and 75 s). The UV-Vis spectra was shown in Fig.1a. Based on the data, we got the graph (Fig.1b) between the number of moles of products (y axis) and time (x axis). Then, the irradiated light intensity was estimated to 1.180 x 10^{-7} einstein S^{-1} by using $K_3[Fe(C_2O_4)_3]$ as an actinometer. For five clean tubes, according to the general procedure, the 0.025 mmol scale model reaction solution was irradiated with two 40 W 390nm LEDs for specified time intervals (0 s, 10 s, 20 s, 30 s, 40 s, and 50 s). The moles of products formed were determined by GC yield with dodecane as reference standard. The number of moles of products (y axis) per unit time is related to the number of photons (x axis, calculated from the light intensity) (Fig.1c). The slope gives the quantum yield (Φ) of the photoreaction, 0.377 (37.7%). Thus, the reaction is less likely to proceed in a radical chain pathway.

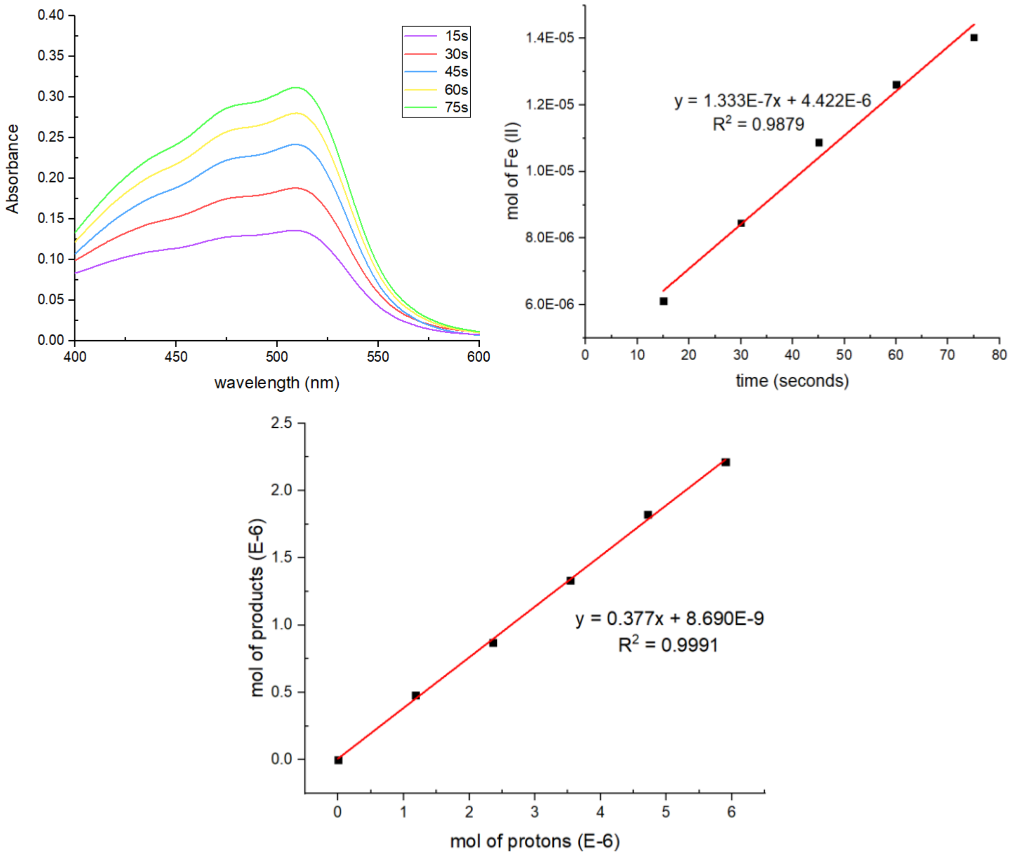

Supplementary Figure 12. Quantum yield measurement

7.9 ^{19}F NMR tracing of the model reaction mixture

In a N_2-filled glove-box, an oven-dried vial (8 mL) equipped with a magnetic stir bar was charged with CuBr (0.29 mg, 0.002 mmol, 2.0 mol%), carbazole **2a** (16.7mg, 0.1 mmol, 1.0 equiv.), ^{t}BuOLi (16.0 mg, 0.2 mmol, 2.0 equiv.), MTBE (4.0 mL) and ^{n}Bu$_3$P (1.2 µL, 0.0048 mmol, 4.8 mol%). The mixture was allowed to stir at rt for 30 min. **1a** (85 µL, 0.5 mmol, 5.0 equiv.) was added sequentially. Then the vial was sealed with a rubber cap, removed from the glove-box and was irradiated under 390 nm LED (40 W × 2) for 1 h at 27 °C with stirring. After 0 min, 5 min, 10 min, 15 min, the samples were taken from reaction mixture and subjected to **^{19}F NMR**. We found that only the formation of **3a** was observed in the reaction, and no other fluorine- containing intermediates or by-products were observed.

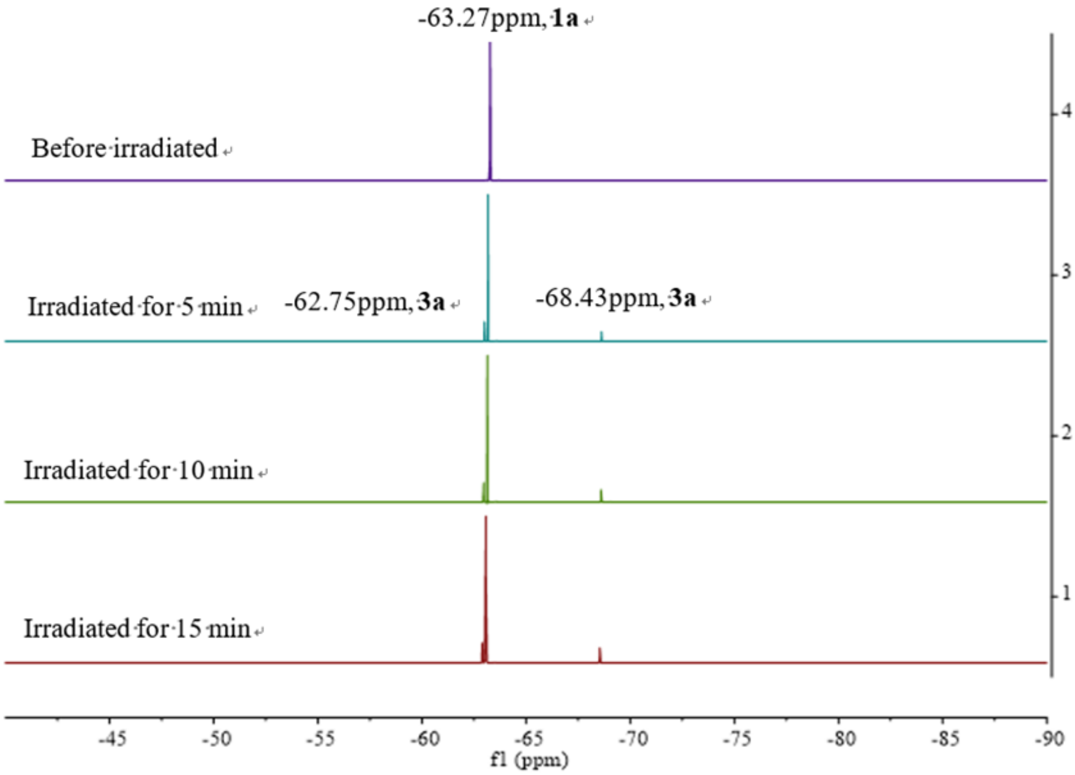

Supplementary Figure 13. ^{19}F NMR tracing of the model reaction mixture

7.10 UV-vis absorption spectroscopic measurement of L1Cu

The UV-vis experiments were performed on F-7000 FL Spectrophotometer (Hitachi, Japan) with a quartz cuvette (10 mm path length). **L1**Cu was measured in Et_2O with 150 µM concentration.
We found that the addition of **1a** didn't significantly affect the UV-visible absorption of **L1**Cu in the range of 300-600 nm. Therefore we speculated that the interaction between **L1**Cu and **1a** is very weak at the ground state, EDA complex pathway is thus less likely.

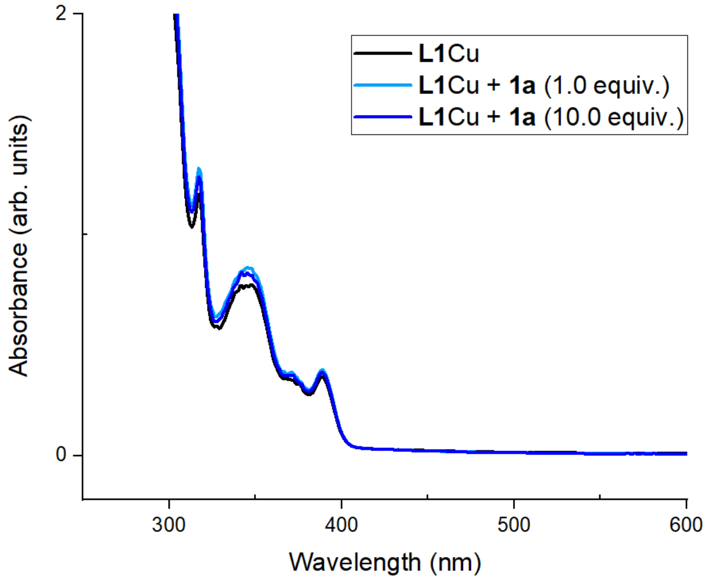

Supplementary Figure 14. UV-vis spectroscopic absorption of **L1**Cu

7.11 Attempt of other nucleophiles

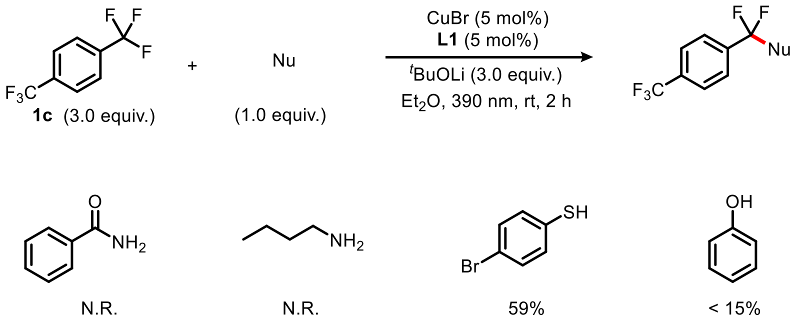

Considering that some strong nucleophilic reagents such as alkylamines, phenols, etc., can not achieve good yields, we think that the reaction is less likely to go through the α,α-difluorobenzyl cation intermediates.

8. DFT Studies

Gaussian 09B program[13] was carried out for all density functional theory (DFT) calculations. All geometry optimizations were conducted with PBE0 functional[14], utilizing the D3 version of Grimme's dispersion correction with Becke-Johnson damping[15]. The def2-TZVP[16] basis set was employed to describe the copper atom, and all other atoms were described by def2-SVP[16] basis set. In view of the solvation effects of ether, the Integral Equation Formalism PCM (IEFPCM)[17-19] was applied. Vibrational frequency analyses were performed at the same theory as geometry optimizations to ensure all optimized stationary points as local minima (zero imaginary frequencies) or transition states (one imaginary frequency), and then thermodynamic corrections for free energies at 300.15 K were derived in Shermo Program[20] accordingly. Cogent evidences of the transition states with the corresponding intermediates were presented by intrinsic reaction coordinate (IRC)[21,22] calculations. Vertical excited energies were computed using time-dependent density function theory (TDDFT)[23] in concert with TPSSh functional[24,25].

The single-point energy calculations were evaluated with PBE0 functional[14] with D3 version of Grimme's dispersion corrections with Becke-Johnson damping[15], and all atoms were treated with def2-TZVP basis set[16]. The solvent (ether) effects were calculated with the Solvent Model Density (SMD)[26] method. All reported Gibbs free energy values were concentration corrected to concentrations corresponding to compounds and intermediates in this reaction. All optimized geometric figures were plotted by CYLview[27]. Simulated UV-vis spectra were calculated with Multiwfn 3.8 dev[28]. All relevant iso-surfaces and the spin-density plot were given by VMD program[29].

The outer-sphere single electron transfer (SET) from the triply excited copper complexes ([**L1**Cu^I]*) to 1,3-bis(trifluoromethyl)benzene, and the C-F bond cleavage sequentially. The energy barrier of the outer-sphere SET ($\Delta G^{\ddagger}$)[30,31] can be estimated by:

$$\Delta G^{\ddagger} = \frac{(\Delta G_r + \lambda)^2}{4\lambda} \tag{S1}$$

$$\lambda = \lambda_i + \lambda_0 \tag{S2}$$

where ΔG_r is the Gibbs free energy change, λ is total reorganization energy, consisting the internal energy (λ_i) and solvent external energy (λ_0). And the internal reorganization energy λ_i was estimated by the 4-point rule, according to Marcus theory:

$$\lambda_{i1} = [E^{D^*}(Q_P) + E^{A}(Q_P)] - [E^{D^*}(Q_R) + E^{A}(Q_R)] \tag{S3}$$

$$\lambda_{i2} = [E^{D+}(Q_R) + E^{A-}(Q_R)] - [E^{D+}(Q_P) + E^{A-}(Q_P)] \tag{S4}$$

$$\lambda_i = \frac{(\lambda_{i1} + \lambda_{i2})}{2} \tag{S5}$$

where λ_{i1} corresponds to the difference between the energy of the products and the energy of the reactants in their geometries respectively. λ_{i2} represents the difference between the energy of the products which donating or accepting an electron and that in their equilibrium geometry. Q_P and Q_R were the equilibrium geometries of the products and reactants respectively.

The external reorganization energy can be approximated as:

$$\lambda_0=332(\frac{1}{2a_1}+\frac{1}{2a_2}-\frac{1}{R})(\frac{1}{\varepsilon_{op}}-\frac{1}{\varepsilon}) \quad \text{(S6)}$$

$$R=a_1+a_2 \quad \text{(S7)}$$

where a_1 and a_2 represent the radii of the donor and acceptor respectively. ε_{op} and ε correspond to the optical dielectric constant and static dielectric constant of the solvent used, for ether, ε_{op}=1.83, ε=4.24.

According to the equations above, λ_i and λ_0 were calculated to be 10.6 and 12.5 kcal·mol^{-1}, so the total reorganization of the outer-sphere SET was 23.1 kcal·mol^{-1}. The free energy change in the SET process was 8.3 kcal·mol^{-1}. Thus the energy barrier of the outer-sphere SET was 10.7 kcal·mol^{-1}.

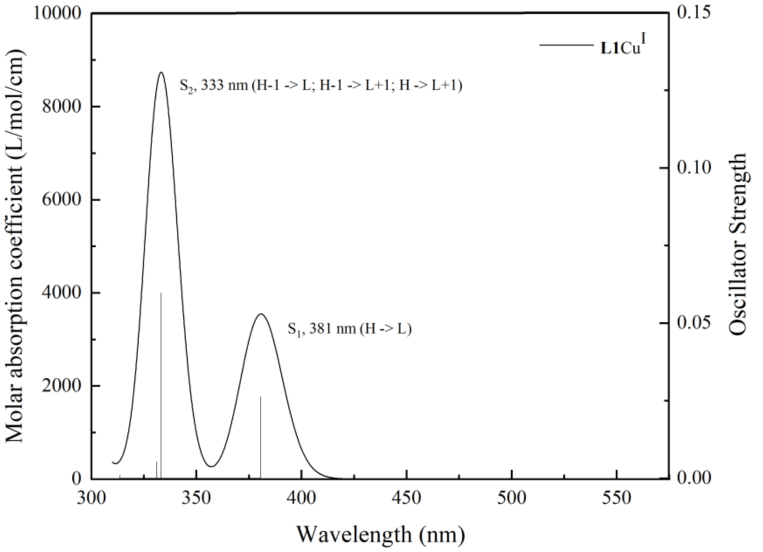

Supplementary Figure 15. Simulated UV-vis absorption spectra with state number and major character for proposed luminescent species using Gaussian broadening with a full-width at half-maximum of 0.2 eV. **L1**Cu^I at TDDFT/TPSSh/def2-SVP/def2-TZVP/IEFPEM(ether) level of theory.

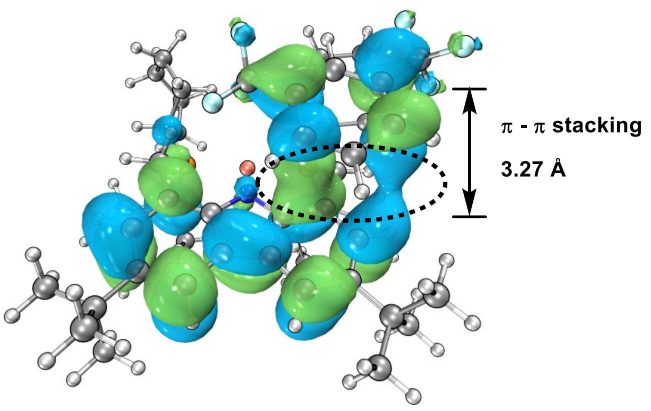

Supplementary Figure 16. π-π stacking interaction between 1,3-bis(trifluoromethyl)-benzene (1a) and carbazole in HOMO of INT1.

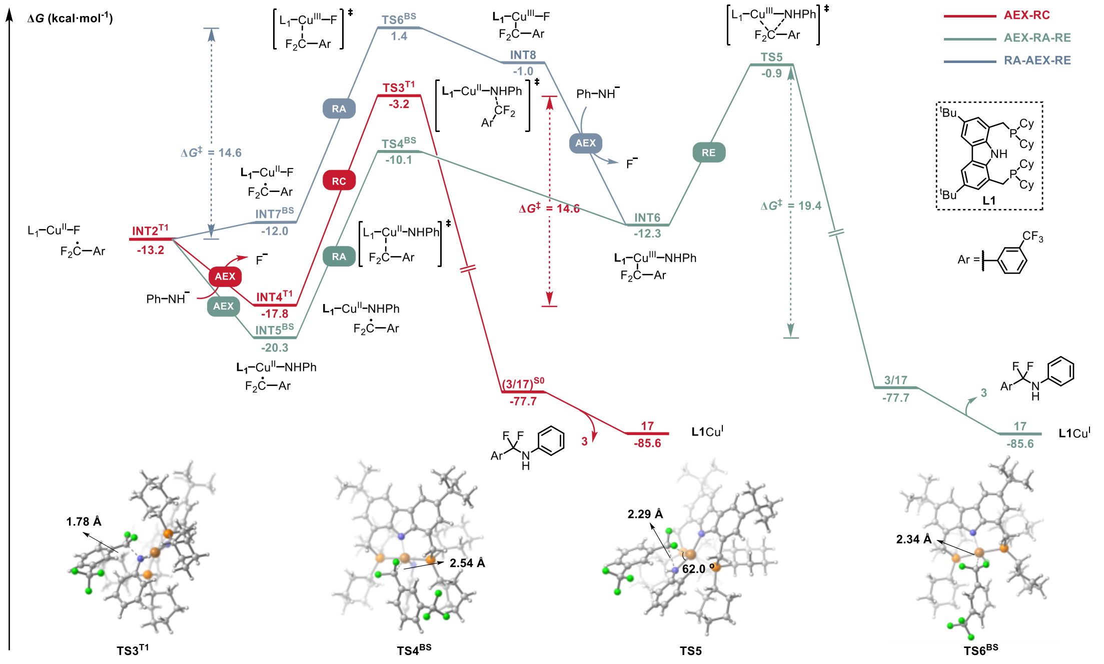

Supplementary Figure 17. Computed potential energy profiles for potential pathways of C-N cross-coupling at the PBE0(D3BJ)/def2-SVP/def2-TZVP/IEFPCM(ether)//PBE0(D3BJ)/def2-TZVP/SMD(ether) level theory. AEX = Anion Exchange, RC = Radical Capturing, RA = Radical Addition, RE = Reductive Elimination. T1= triplet state. BS = broken-symmetry state, S0 = singlet ground state.

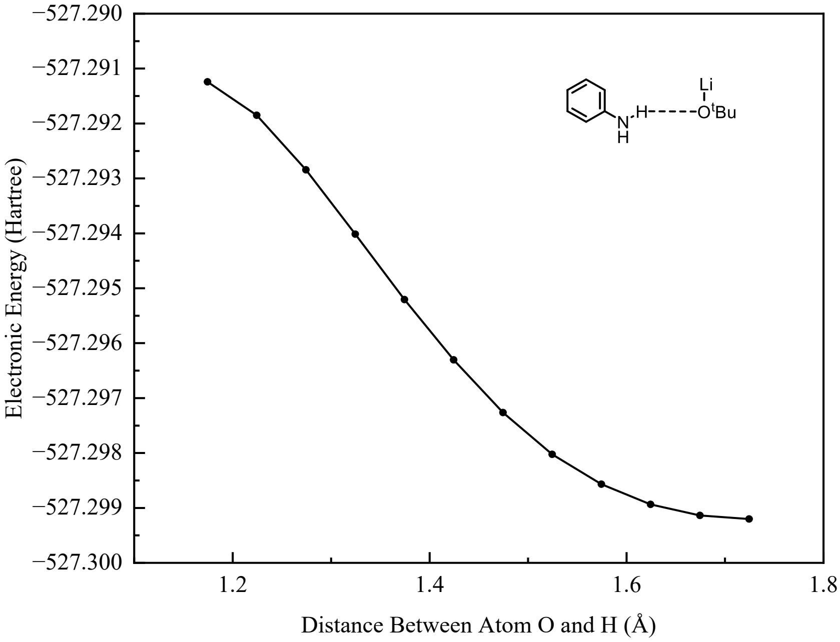

Supplementary Figure 18. Relaxed scan for the electronic potential energy surface along the distance between O atom of ^{t}BuOLi and H atom of anilines.

In the presence of ^{t}BuOLi, a barrierless formation of the aniline anion could occur (Supplemetary Figure 17), which readily engaged in anion exchange (AEX) with [**L1**Cu^{II}-F], yielding [**L1**Cu^{II}-NHPh] species. This Cu^{II}-species can not only serve as an intermediary for the subsequent C-N coupling process, but also thermodynamically stable, with a free energy release of 4.1 kcal·mol^{-1}.

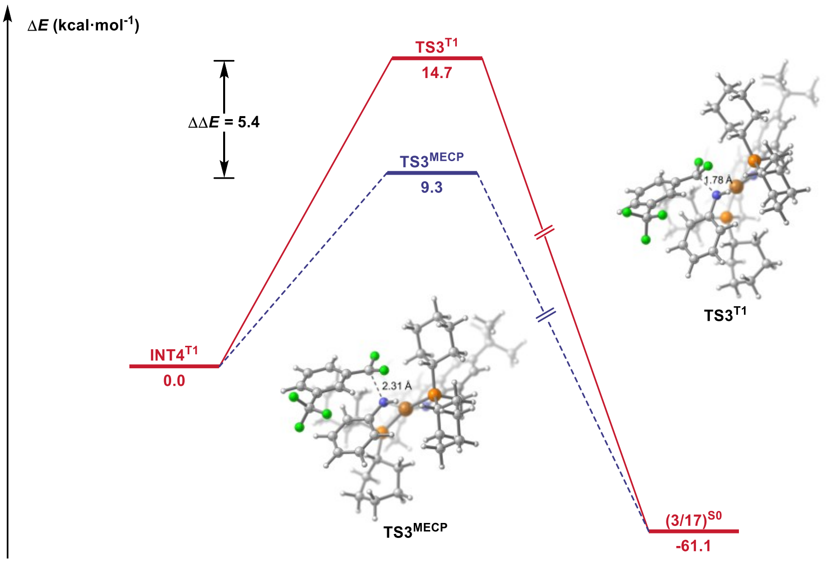

Supplementary Figure 19. Computed electronic potential energy profiles for pathways of radical capturing involving **TS3** in the triplet state or through MECP.

Drawing from earlier studies[32,33], we postulated potential pathways for C-N coupling, involving either direct capture of the α-*N*-benzylic radical by N atoms (radical capturing) or the addition of difluorobenzylic radical to the copper center, subsequently followed by reductive elimination (RE). In the radical capturing (RC) pathway, the C-N coupling process takes place when difluorobenzylic radical approaches the amine moiety, leading to a transition state (**TS3^{T1}**) with a free energy barrier of 14.6 kcal·mol^{-1}. Alternatively, critical Cu^{III}-species intermediates can form from the radical addition (RA) of difluorobenzylic radical to the Cu^{II}-metal center, resulting in **INT6** or **INT8**, depending on the order RA and AEX events. Regarding the radical addition process (RA) of difluorobenzylic radical to [**L1**Cu^{II}-F], the energy barrier for the rate-determination step (**TS6^{BS}**) is 14.6 kcal·mol^{-1}. While in the scenario that leads to **INT6**, although its key intermediate (**INT5^{BS}**) for RA reveals significant thermodynamics favorability, the overall free energy barrier of the entire pathway is 19.4 kcal·mol^{-1}, surpassing that of the RC pathway (14.6 kcal·mol^{-1}). It is noted that the transition states (**TS4^{BS}** and **TS6^{BS}**) for RA process could be only found in broken symmetry (BS) states, where both fragments in this step share the same spin doublet state. We also note that the addition of difluorobenzylic radical to the Cu^{II}-metal center can be viewed as the combination of two radicals, potentially undergoing a spin crossover process, since the reactant have a triplet spin state and the C-N coupling product possesses a singlet state. This energy barrier could be represented by the minimum energy crossing point (MECP), with the optimized structure and energy of the transition state determined by sobMECP[34]. The electronic energy of the transition state (**TS3^{MECP}**) through MECP was found to be 5.4 kcal·mol^{-1} lower than that of the triplet state (**TS3^{T1}**) (Supplementary Figure 19). Consequently, the C-N coupling preferentially progressed via the RC pathway. Taking all these insights into consideration, our computational results suggest that the reaction favored the ISET pathway, followed by MECP mediated RC process.

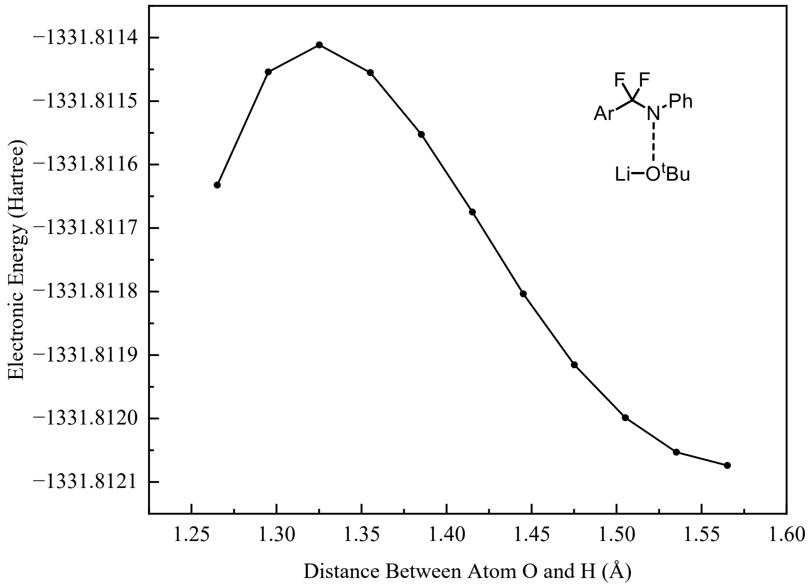

Supplementary Figure 20. Relaxed scan of potential surface for the distance between the O atom in ^{t}BuOLi and the acidic H atom in α,α-difluoromethylamines.

Furthermore, DFT studies revealed an absence of a barrier for the elimination of hydrogen fluoride. Although we located a transition state on the electronic potential energy surface (Supplementary Figure 20), its free energy was lower than the precursor by 0.33 kcal/mol, hence we regard it as a barrierless reaction. Thus, the Boltzmann distribution can be used for explaining the E/Z selectivity. The free energy difference between *E*- and *Z*- imidoyl fluoride (**9**) is 2.3 kcal·mol^{-1}, with the estimated distribution for the dominant configuration (**9**) is 97.9%, consistent with experimental results.

Images of key structures

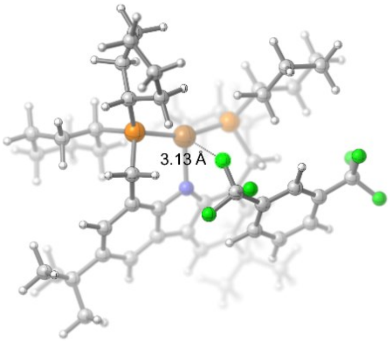

INT1

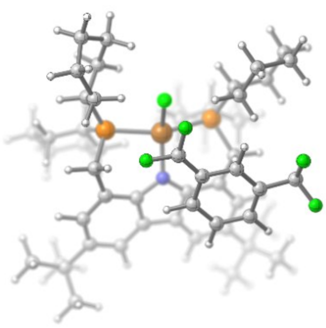

INT2^{T1}

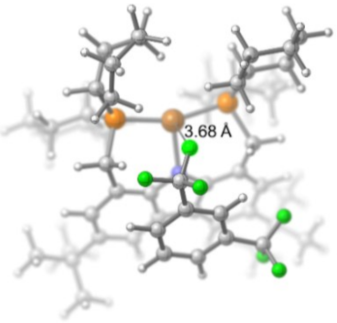

INT3

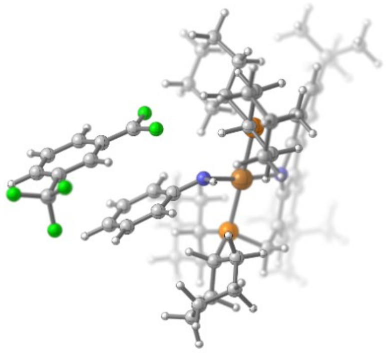

INT4^{T1}

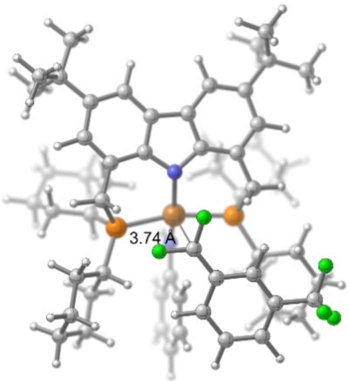

INT5^{BS}

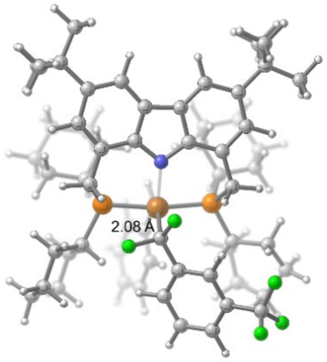

INT6

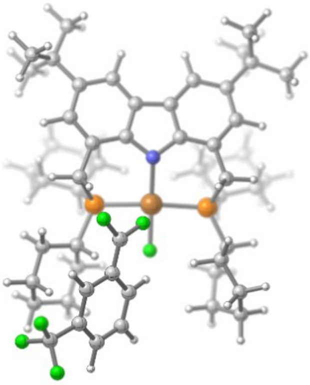

INT7^{BS}

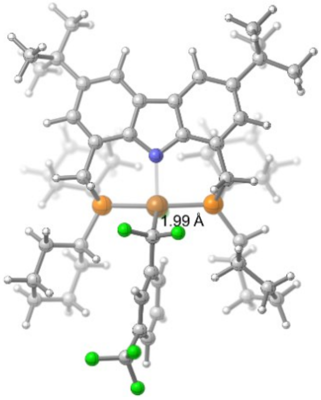

INT8

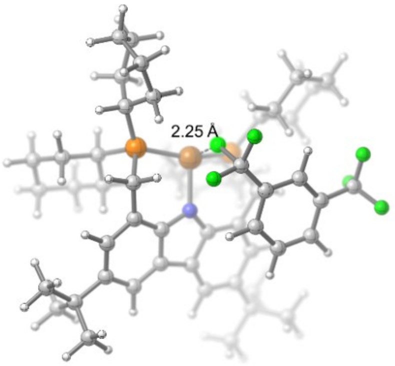

TS1

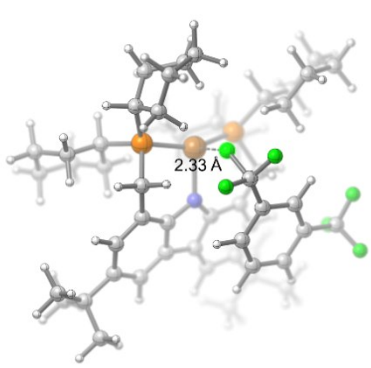

TS2

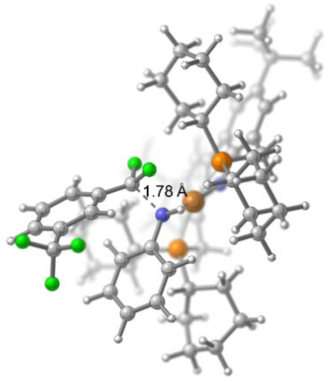

TS3^{T1}

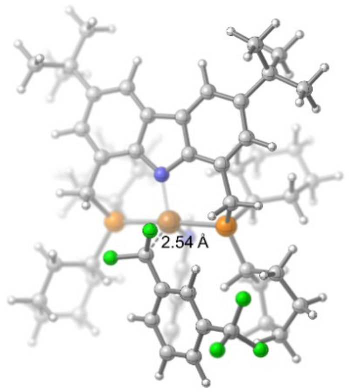

TS4^{BS}

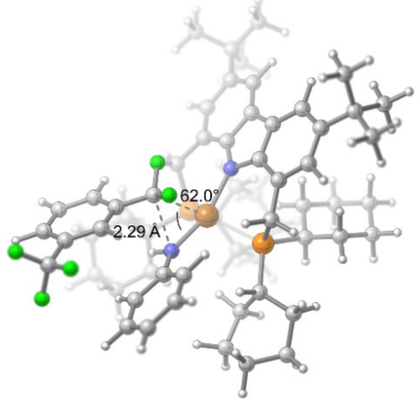

TS5

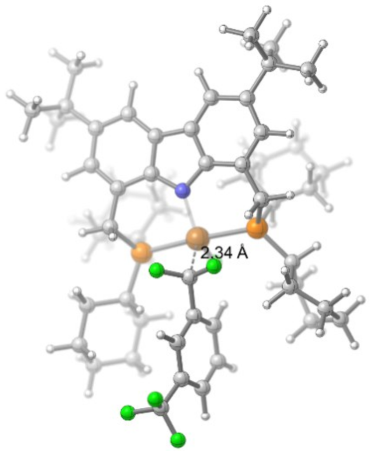

TS6^{BS}

9. X-Ray Crystal Data

Compound **3u** was crystallized as a colorless crystal via vaporization of a hexane/ethyl acetate solution, and its relative configuration is determined by x-ray structure analaysis. CCDC 2212746 contains the supplementary crystallographic data that can be obtained free of charge from The Cambridge Crystallographic Data Centre via www.ccdc.cam.ac.uk/data_request/cif.

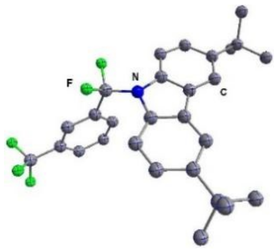

Supplementary Figure 21. X-Ray Crystal of **3u**

Identification code	CCDC 2212746
Empirical formula	$C_{28}H_{28}F_5N$
Formula weight	473.51
Temperature	296(2) K
Wavelength	0.71073 Å
Crystal system	Monoclinic
Space group	P 21/c
a/Å	12.606(5)
b/Å	15.731(6)
c/Å	12.931(6)
α/°	90
β/°	107.499(13)
γ/°	90
Volume	2445.9(17) Å3
Z	4
Density (calculated)	1.286 Mg/m3
Absorption coefficient	0.101 mm-1
F(000)	992
Theta range for data collection	2.098 to 27.517°
Index ranges	-16<=h<=16, -19<=k<=20, -16<=l<=14
Reflections collected	20918
Independent reflections	5474 [R(int) = 0.0708]
Completeness to theta = 25.242°	97.50%
Refinement method	Full-matrix least-squares on F2
Data / restraints / parameters	5474 / 24 / 313
Goodness-of-fit on F2	1.154
Final R indices [I>2sigma(I)]	R1 = 0.1003, wR2 = 0.2953
R indices (all data)	R1 = 0.1417, wR2 = 0.3496
Largest diff. peak and hole	0.785 and -0.393 e.Å-3

Compound **L1**Cu was crystallized as a colorless crystal via vaporization of a hexane/ethyl acetate solution, and its relative configuration is determined by x-ray structure anaalysis. CCDC 2270870 contains the supplementary crystallographic data that can be obtained free of charge from The Cambridge Crystallographic Data Centre via www.ccdc.cam.ac.uk/data_request/cif.

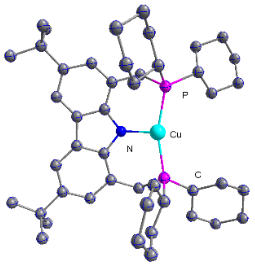

Supplementary Figure 22. X-Ray Crystal of **L1Cu**

Identification code	CCDC 2270870
Empirical formula	$C_{46}H_{70}CuNP_2$
Formula weight	762.51
Temperature/K	193.00
Crystal system	triclinic
Space group	P-1
a/Å	12.0219(7)
b/Å	12.9859(8)
c/Å	15.4197(7)
α/°	67.084(2)
β/°	71.423(2)
γ/°	76.487(2)
Volume/Å^3	2085.1(2)
Z	2
ρ_{calc}g/cm^3	1.217
μ/mm^{-1}	0.698
F(000)	826.0
Crystal size/mm^3	0.13 × 0.12 × 0.11
Radiation	MoKα (λ = 0.71073)
2Θ range for data collection/°	3.602 to 54.946
Index ranges	$-15 \leq h \leq 15$, $-16 \leq k \leq 14$, $-19 \leq l \leq 19$
Reflections collected	19220
Independent reflections	9482 [R_{int} = 0.0699, R_{sigma} = 0.1332]
Data/restraints/parameters	9482/234/626
Goodness-of-fit on F^2	1.037
Final R indexes [I>=2σ (I)]	R_1 = 0.0881, wR_2 = 0.1688
Final R indexes [all data]	R_1 = 0.1847, wR_2 = 0.2162
Largest diff. peak/hole / e Å^{-3}	0.46/-0.54

Compound **9b** was crystallized as a colorless crystal via vaporization of a hexane/ dichloromethane solution, and its relative configuration is determined by x-ray structure analaysis. CCDC 2281146 contains the supplementary crystallographic data that can be obtained free of charge from The Cambridge Crystallographic Data Centre via www.ccdc.cam.ac.uk/data_request/cif.

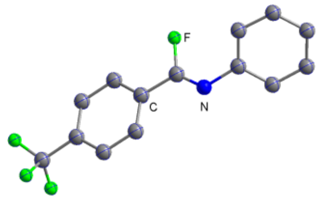

Supplementary Figure 23. X-Ray Crystal of **9b**

Identification code	CCDC 2281146
Empirical formula	$C_{14}H_9F_4N$
Formula weight	267.22
Temperature/K	193.00
Crystal system	monoclinic
Space group	P21/n
a/Å	5.6334(3)
b/Å	32.3772(18)
c/Å	6.9221(4)
α/°	90
β/°	107.414(2)
γ/°	90
Volume/?3	1204.68(12)
Z	4
ρcalcg/cm3	1.473
μ/mm?1	0.726
F(000)	544.0
Crystal size/mm3	0.2 × 0.18 × 0.16
Radiation	GaKα (λ = 1.34139)
2Θ range for data collection/°	4.748 to 108.096
Index ranges	-6 ≤ h ≤ 6, -39 ≤ k ≤ 38, -8 ≤ l ≤ 4
Reflections collected	7683
Independent reflections	2199 [Rint = 0.0342, Rsigma = 0.0316]
Data/restraints/parameters	2199/52/210
Goodness-of-fit on F2	1.039
Final R indexes [I>=2σ (I)]	R1 = 0.0454, wR2 = 0.1109
Final R indexes [all data]	R1 = 0.0652, wR2 = 0.1241
Largest diff. peak/hole / e Å -3	0.15/-0.25

10. Copies of NMR Spectra

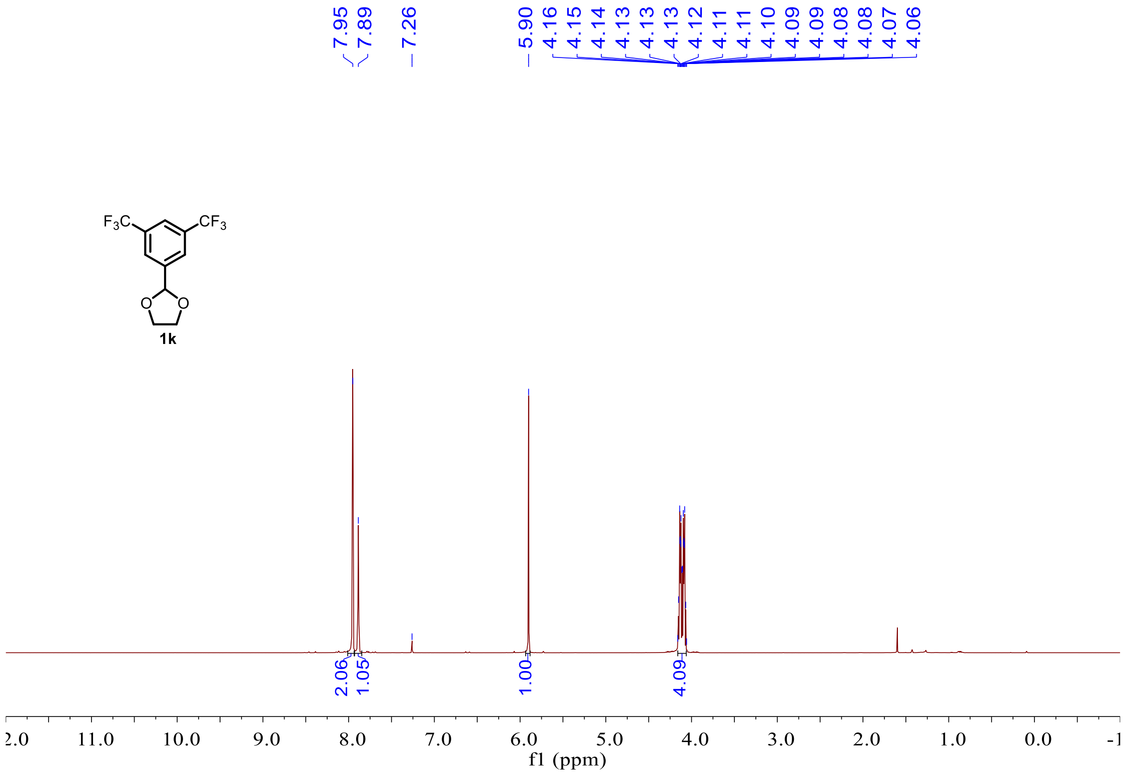

Supplementary Figure 24. **^{1}H NMR** (500 MHz, $CDCl_3$) spectrum for compound **1k**

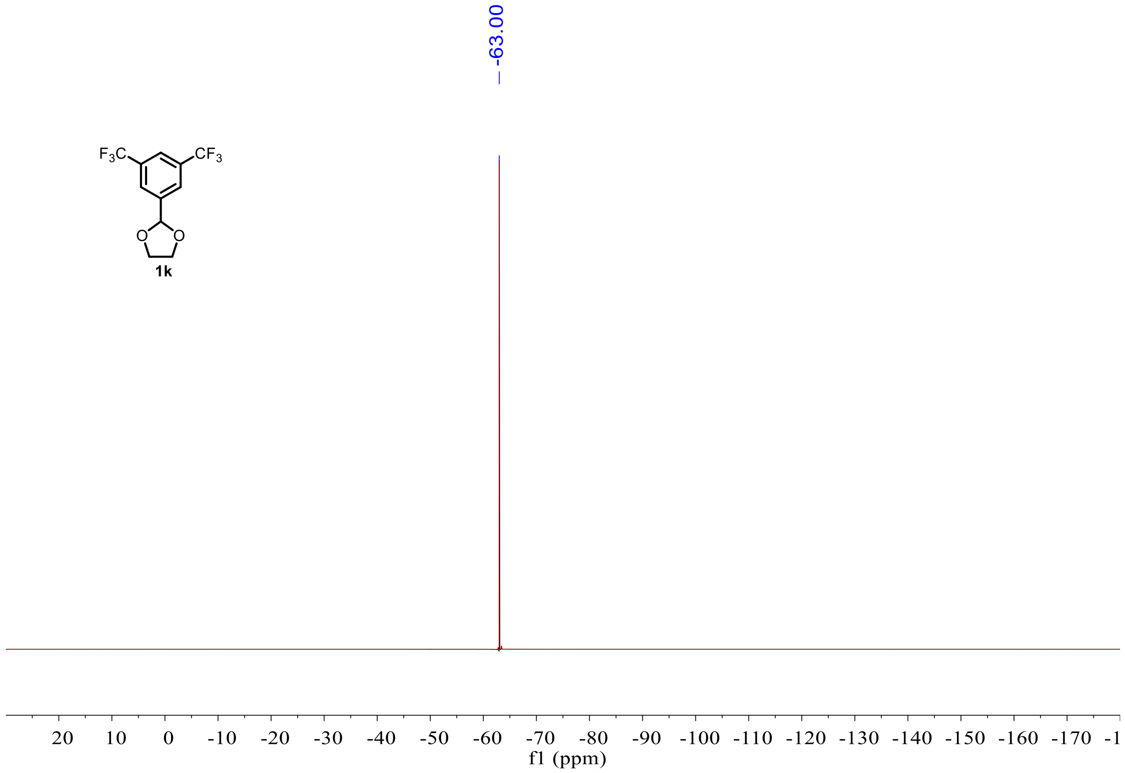

Supplementary Figure 25. **^{19}F NMR** (471 MHz, $CDCl_3$) spectrum for compound **1k**

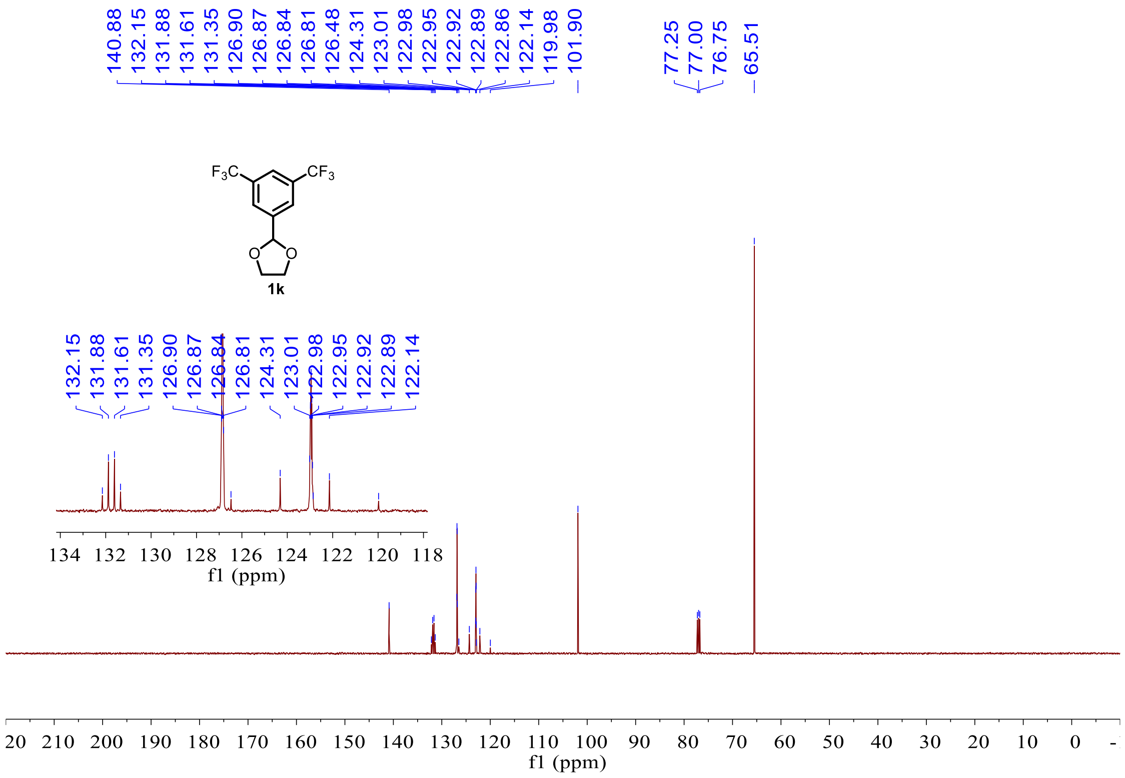

Supplementary Figure 26. ^{13}C NMR (126 MHz, $CDCl_3$) spectrum for compound **1k**

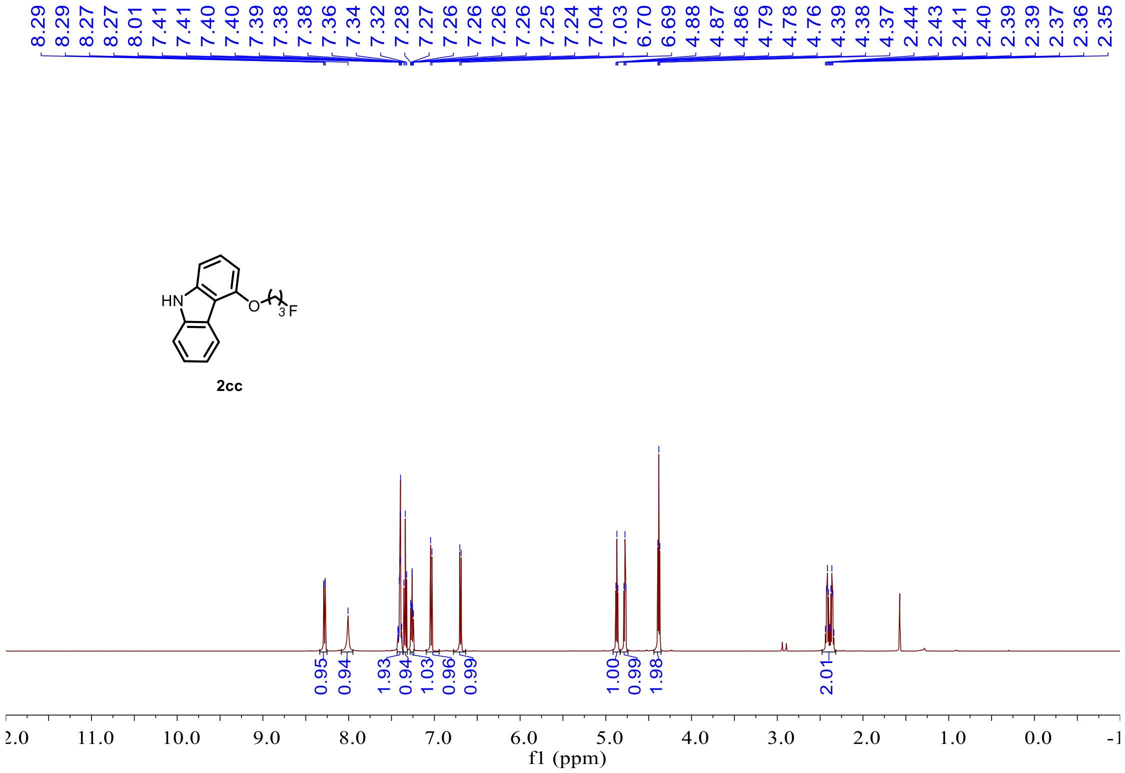

Supplementary Figure 27. ^{1}H NMR (500 MHz, $CDCl_3$) spectrum for compound **2cc**

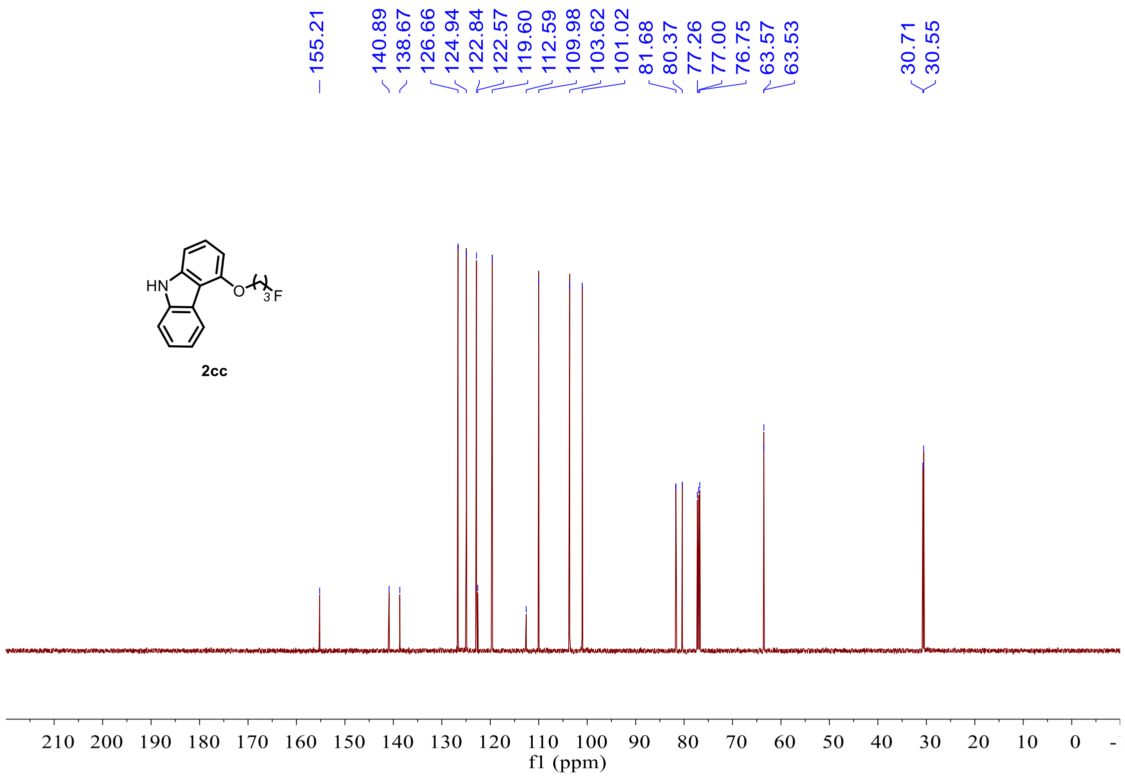

Supplementary Figure 28. ^{13}**C NMR** (126 MHz, $CDCl_3$) spectrum for compound **2cc**

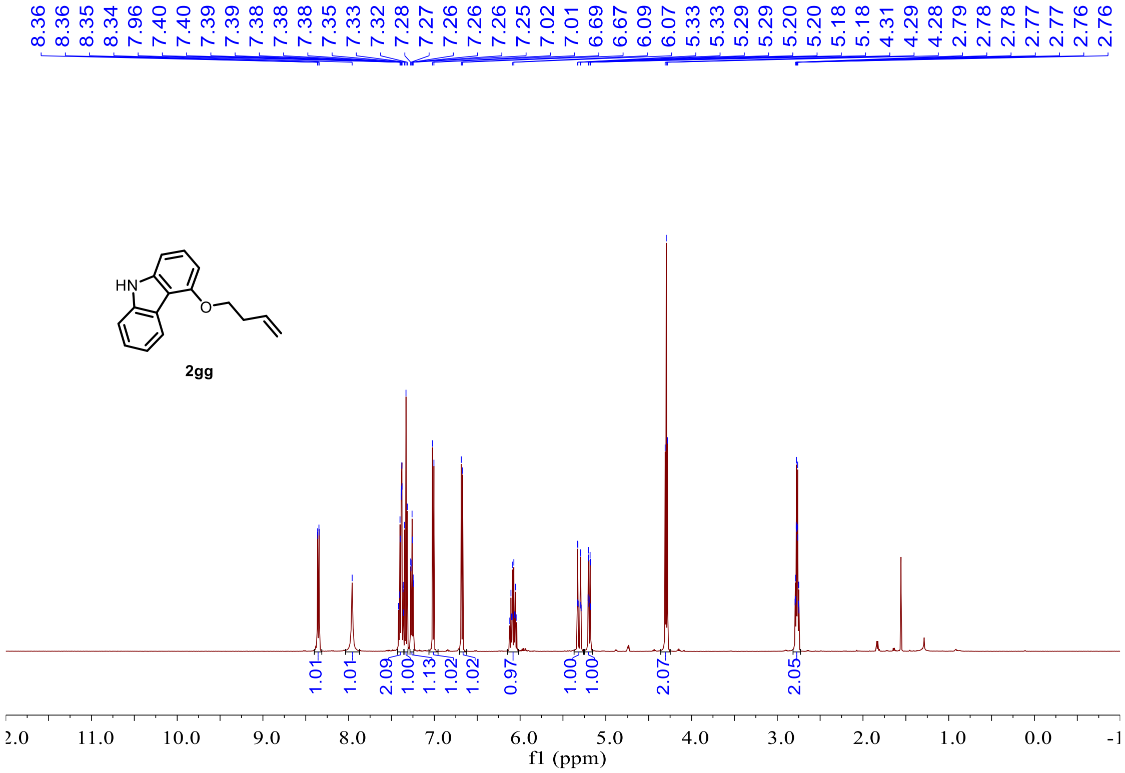

Supplementary Figure 29. ^{1}**H NMR** (500 MHz, $CDCl_3$) spectrum for compound **2gg**

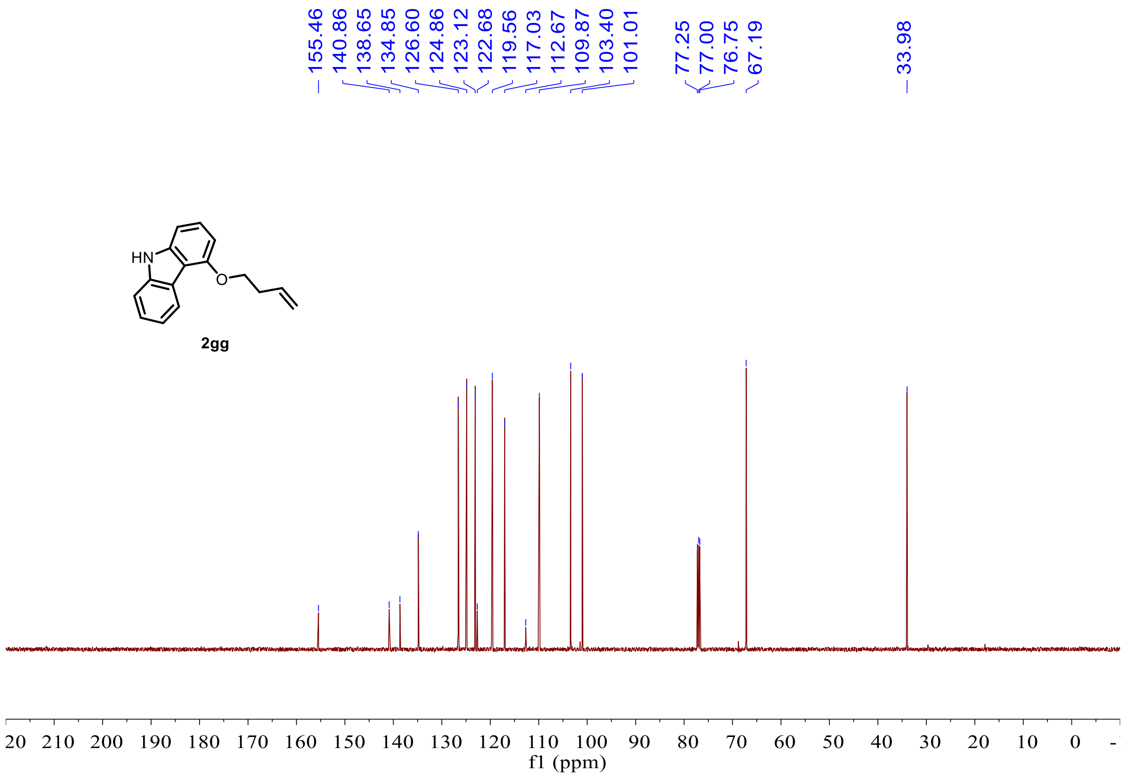

Supplementary Figure 30. ^{13}**C NMR** (126 MHz, $CDCl_3$) spectrum for compound **2gg**

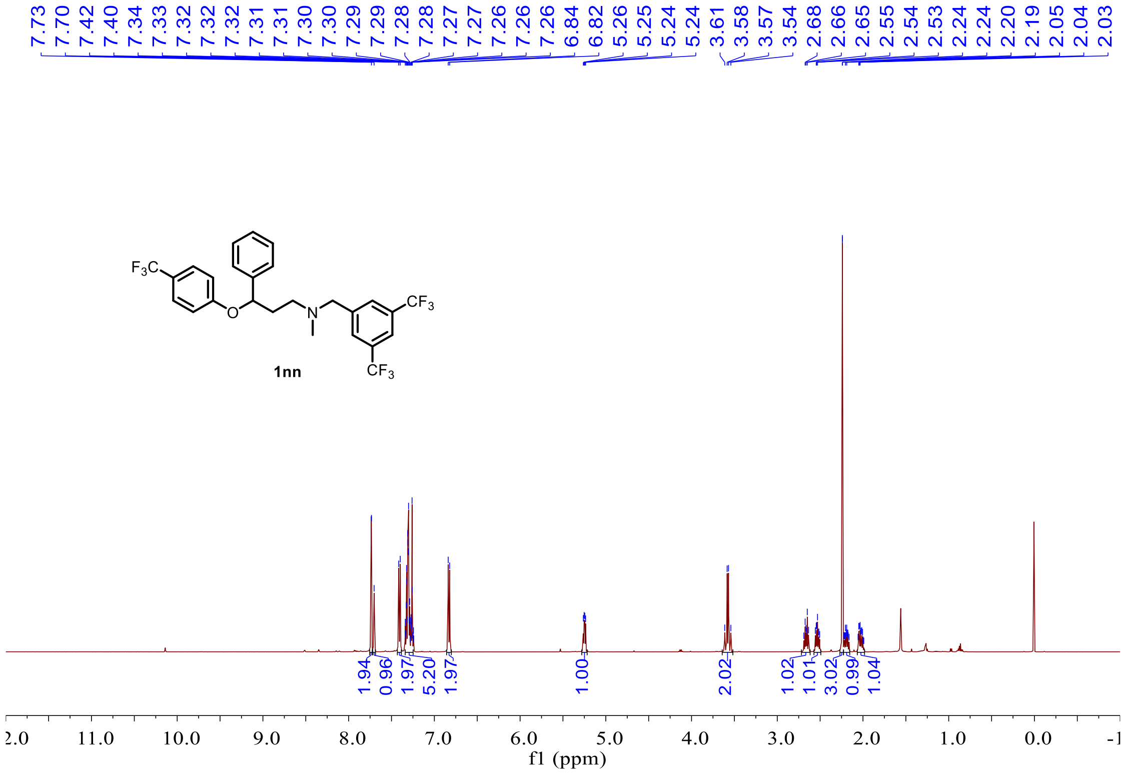

Supplementary Figure 31. ^{1}**H NMR** (500 MHz, $CDCl_3$) spectrum for compound **1nn**

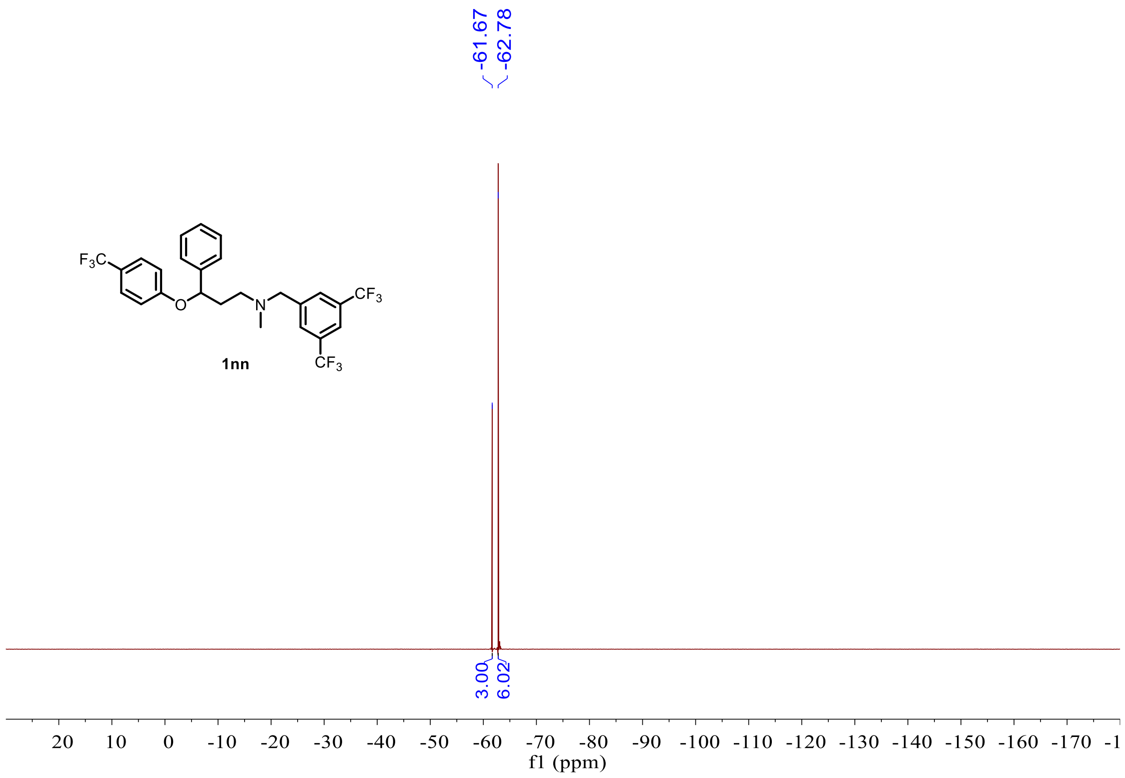

Supplementary Figure 32. ^{19}**F NMR** (471 MHz, $CDCl_3$) spectrum for compound **1nn**

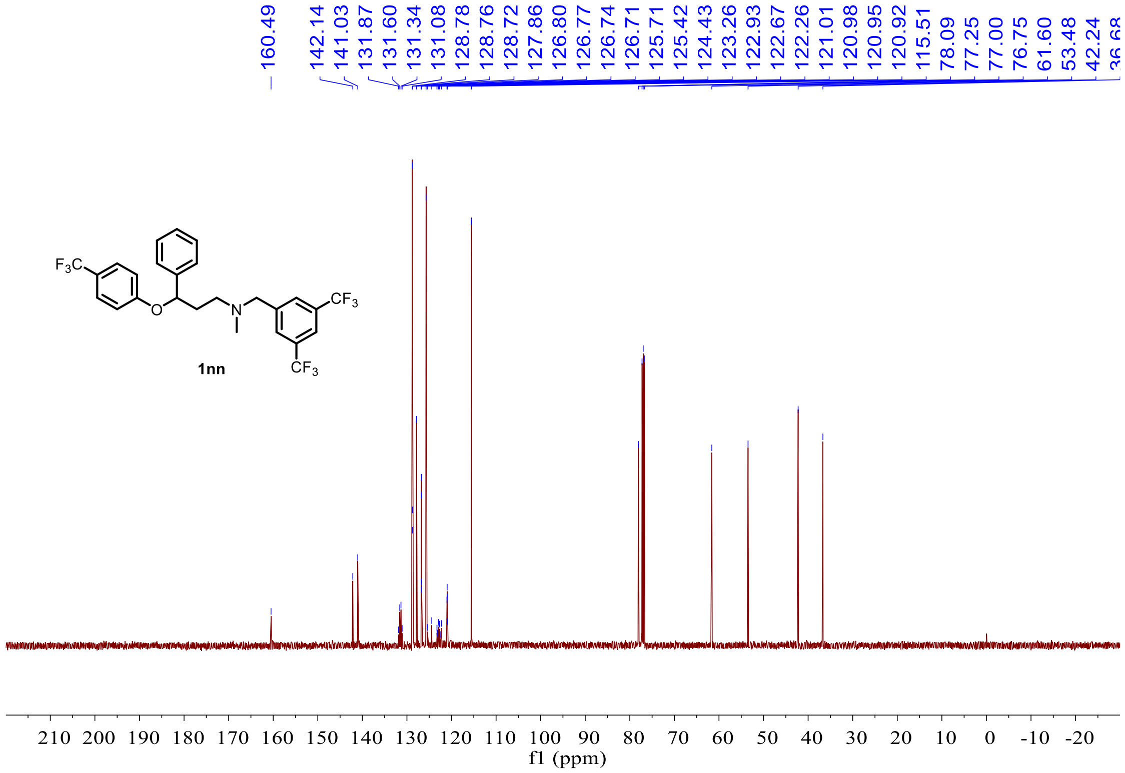

Supplementary Figure 33. ^{13}**C NMR** (126 MHz, $CDCl_3$) spectrum for compound **1nn**

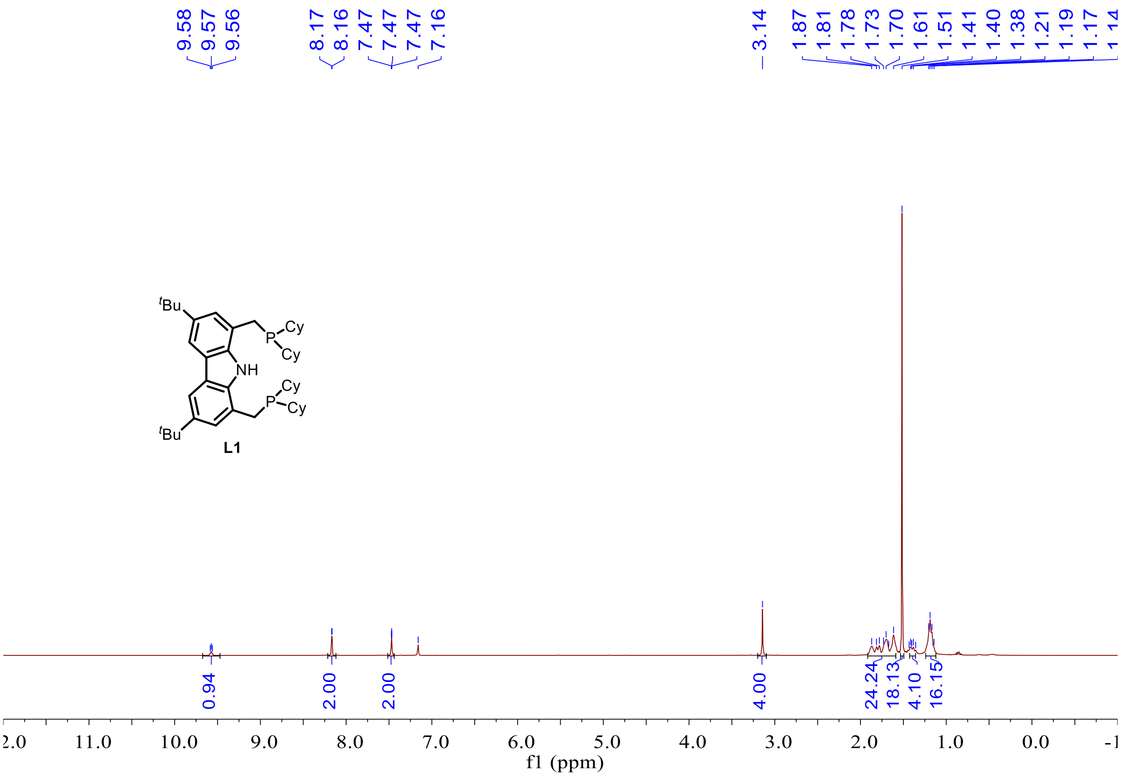

Supplementary Figure 34. ^{1}H NMR (400 MHz, Benzene-d_6) spectrum for compound **L1**

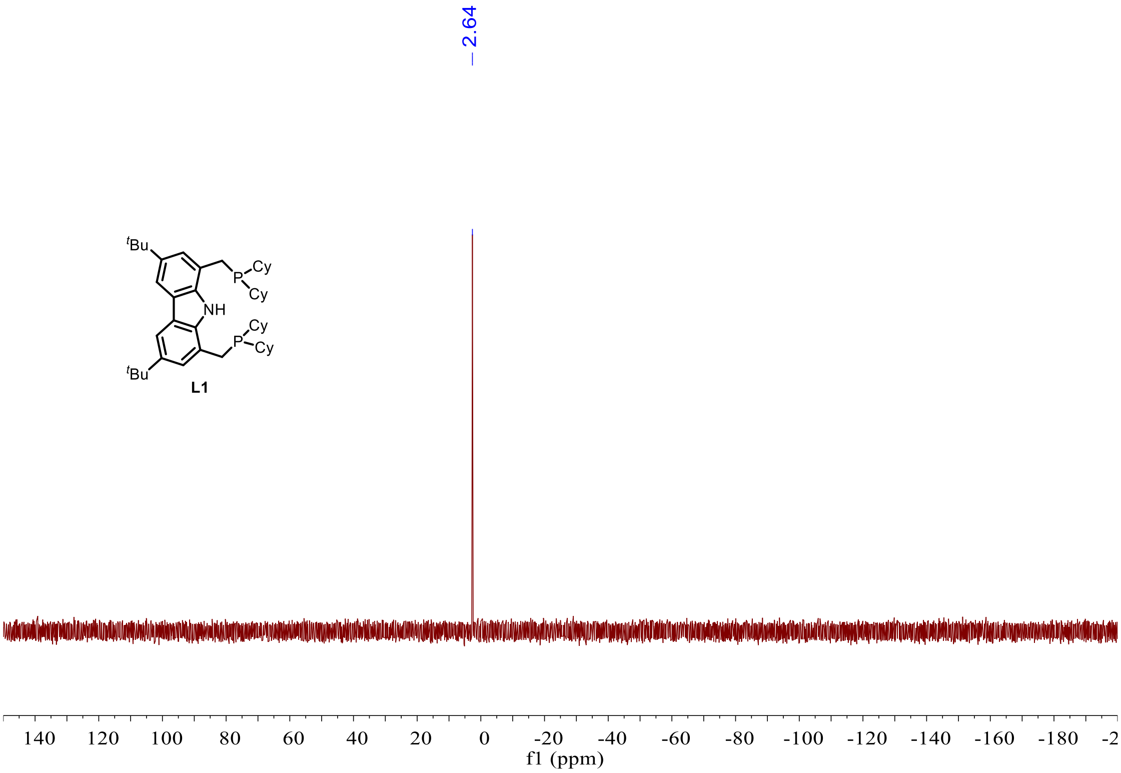

Supplementary Figure 35. ^{31}P NMR (162 MHz, Benzene-d_6) spectrum for compound **L1**

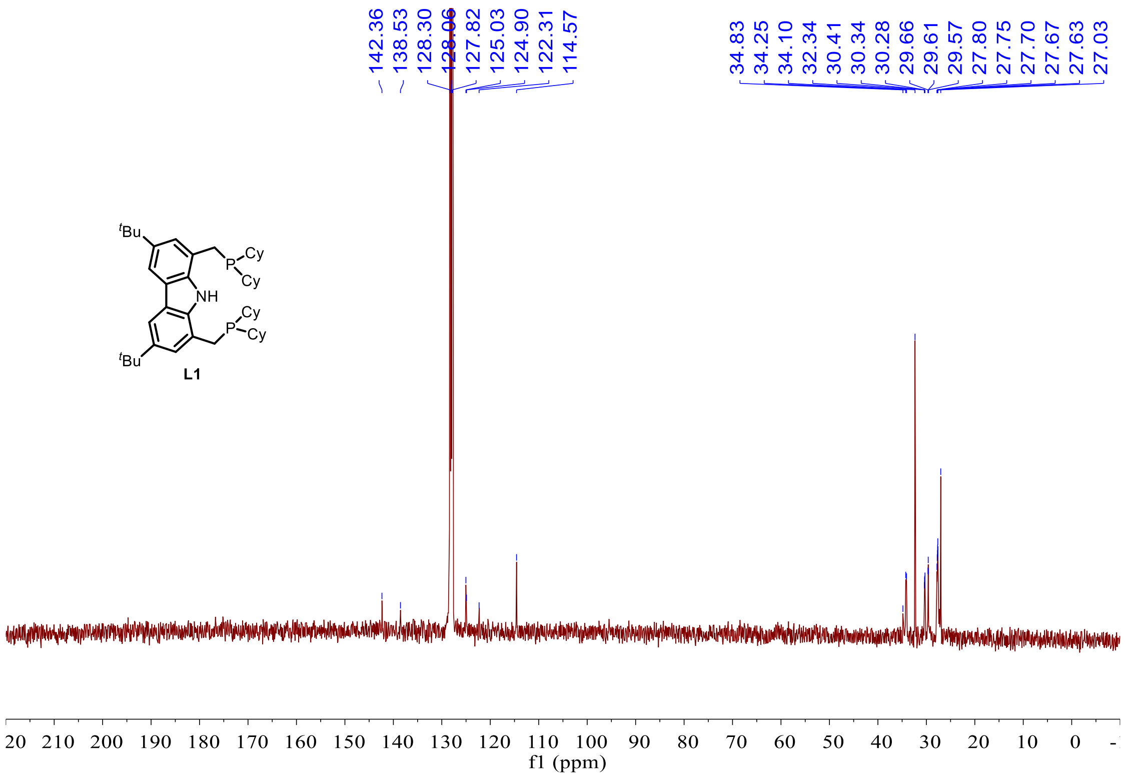

Supplementary Figure 36. ^{13}**C NMR** (101 MHz, Benzene-d_6) spectrum for compound **L1**

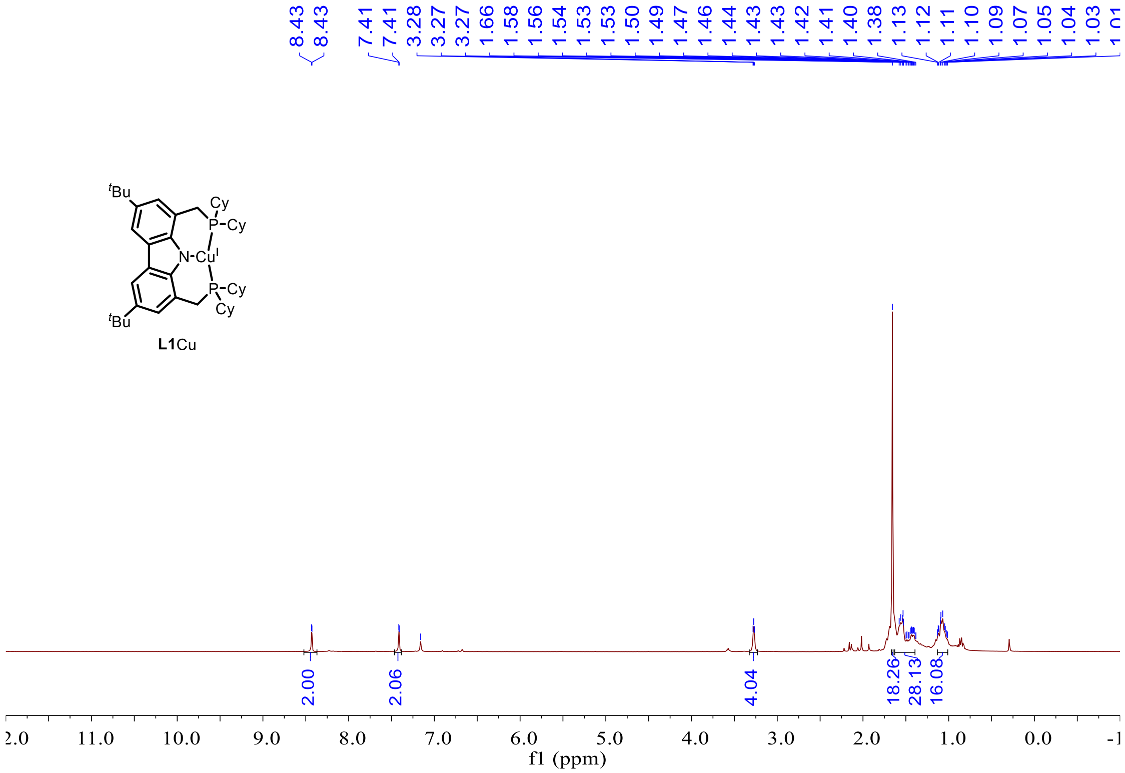

Supplementary Figure 37. ^{1}**H NMR** (400 MHz, Benzene-d_6) spectrum for compound **L1**Cu

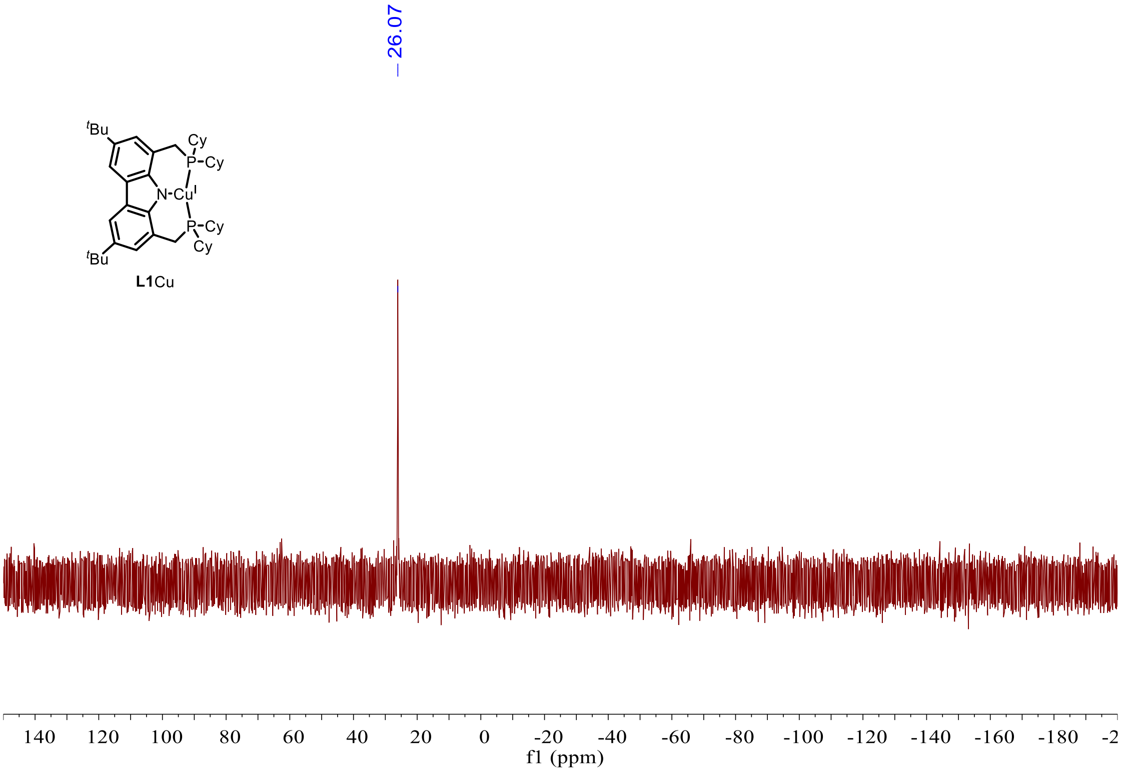

Supplementary Figure 38. ^{31}**P NMR** (162 MHz, Benzene-d_6) spectrum for compound **L1**Cu

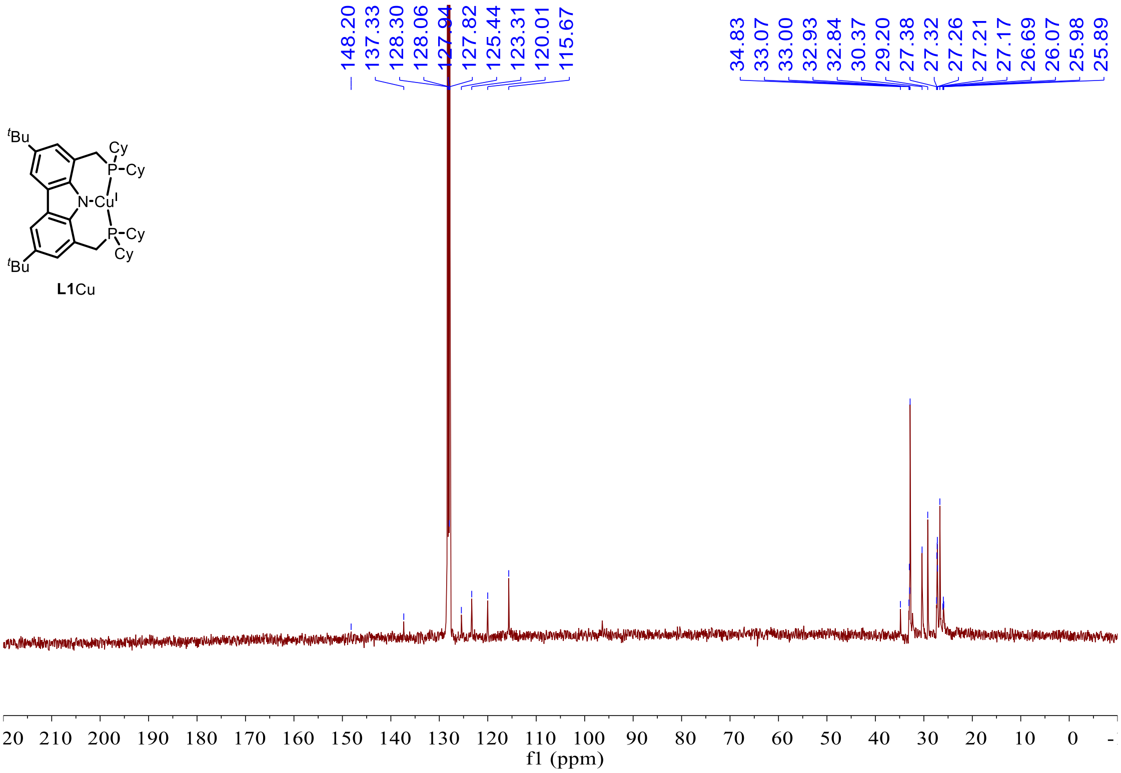

Supplementary Figure 39. ^{13}**C NMR** (101 MHz, Benzene-d_6) spectrum for compound **L1**Cu

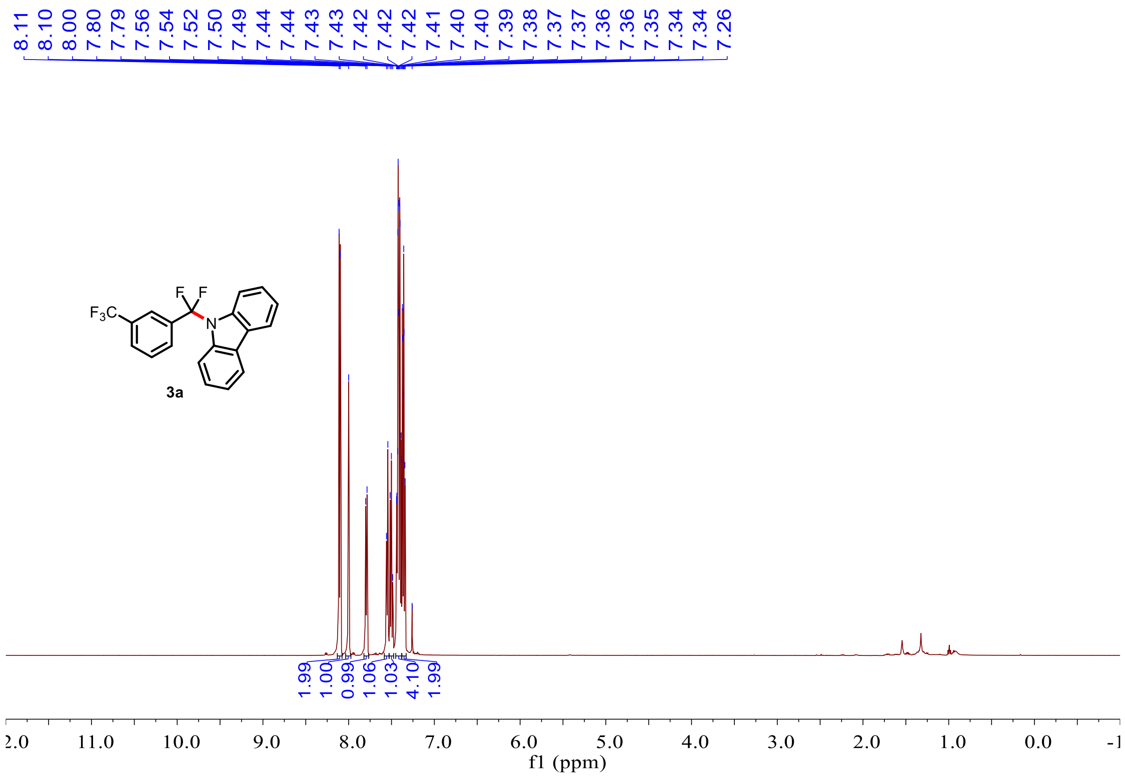

Supplementary Figure 40. ^{1}H NMR (500 MHz, $CDCl_3$) spectrum for compound **3a**

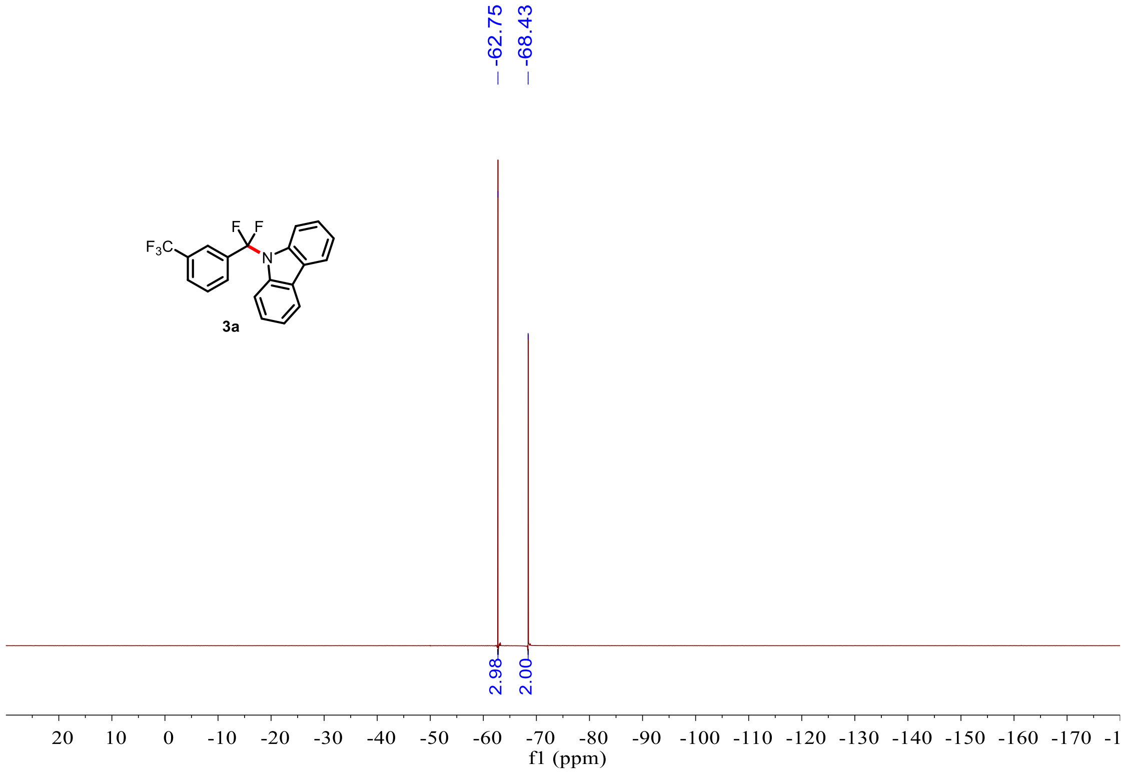

Supplementary Figure 41. ^{19}F NMR (471 MHz, $CDCl_3$) spectrum for compound **3a**

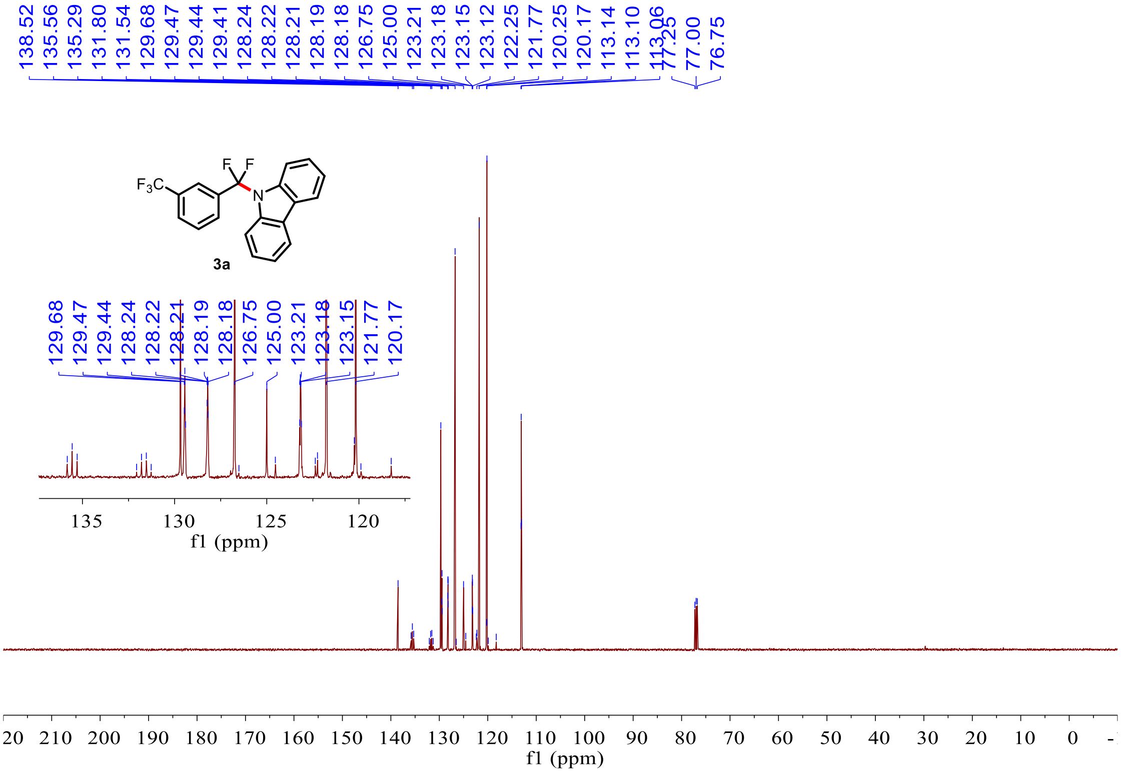

Supplementary Figure 42. ^{13}**C NMR** (126 MHz, $CDCl_3$) spectrum for compound **3a**

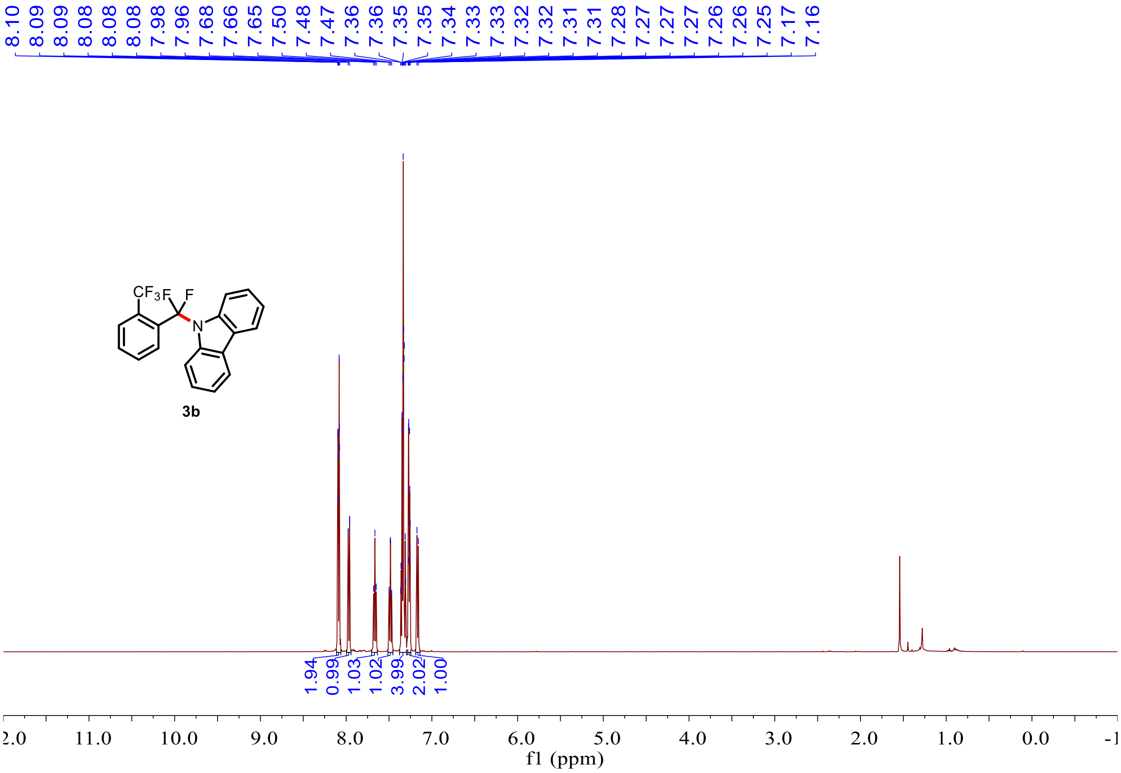

Supplementary Figure 43. ^{1}**H NMR** (500 MHz, $CDCl_3$) spectrum for compound **3b**

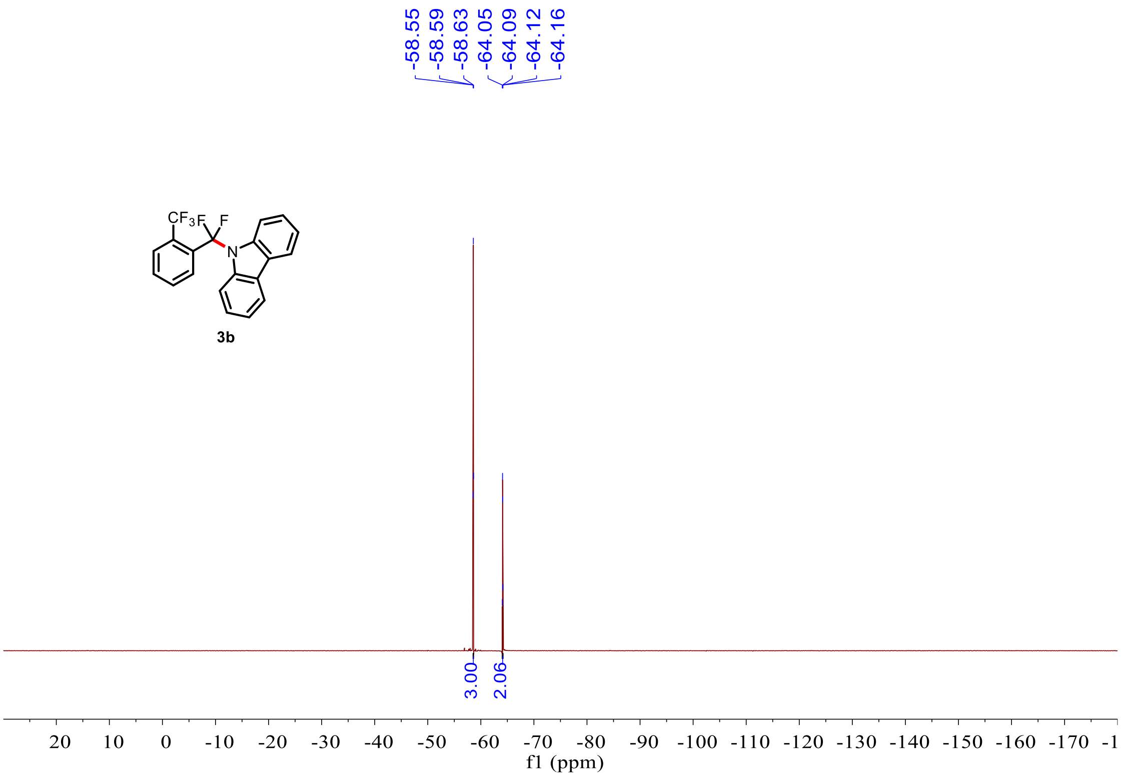

Supplementary Figure 44. ^{19}**F NMR** (471 MHz, $CDCl_3$) spectrum for compound **3b**

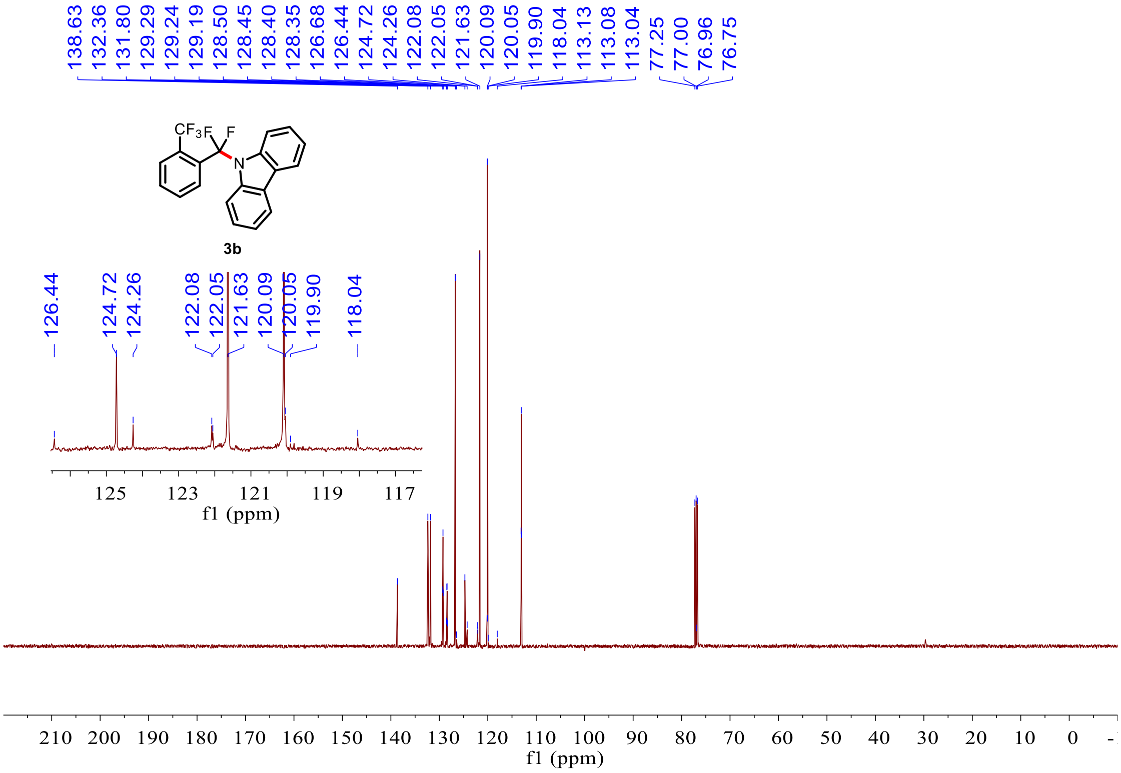

Supplementary Figure 45. ^{13}**C NMR** (126 MHz, $CDCl_3$) spectrum for compound **3b**

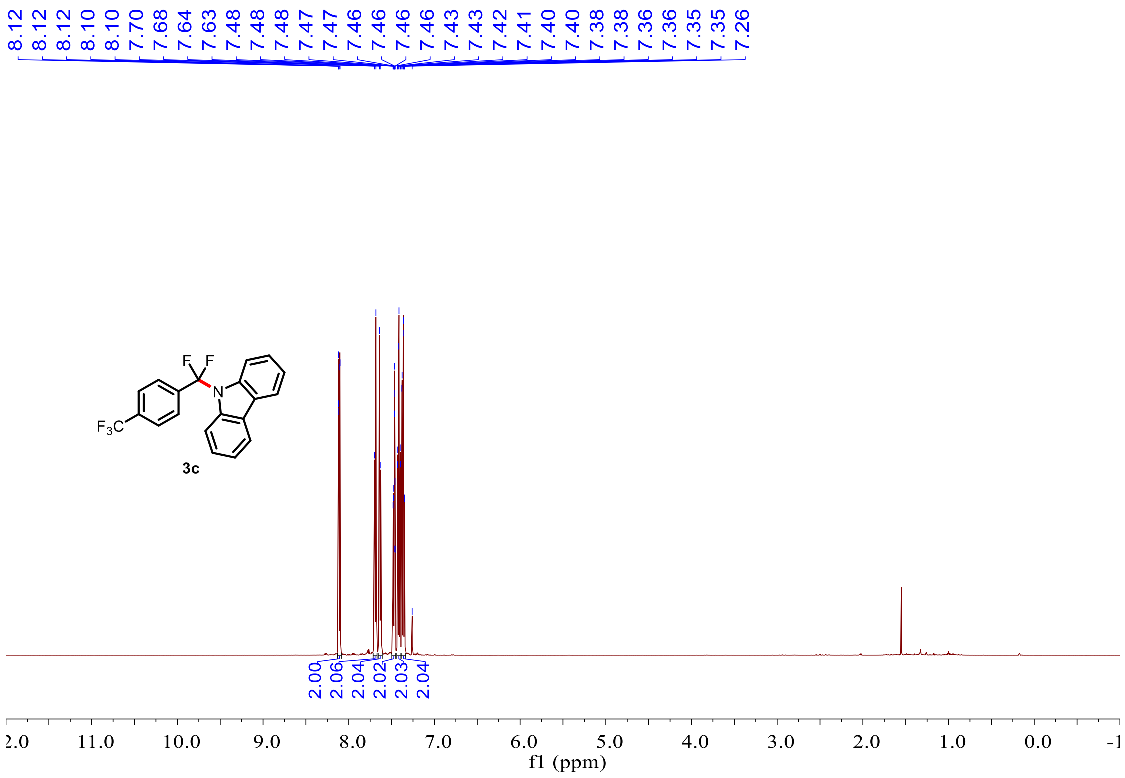

Supplementary Figure 46. ^{1}H NMR (500 MHz, $CDCl_3$) spectrum for compound **3c**

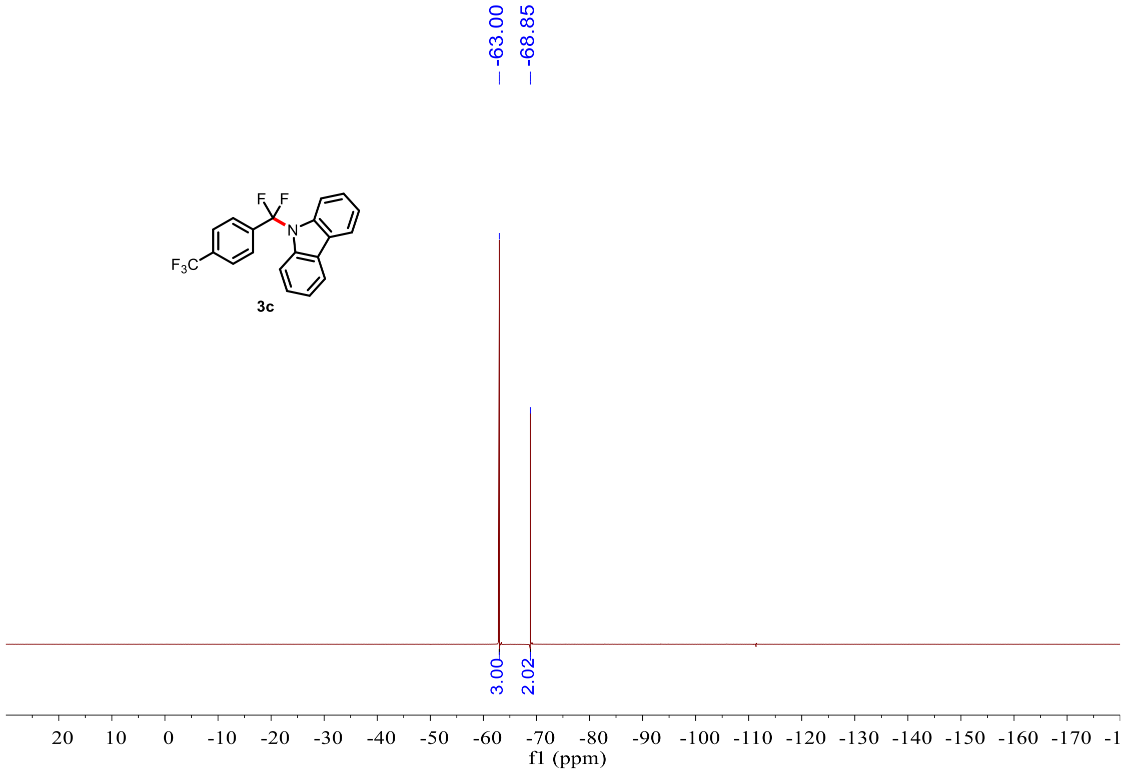

Supplementary Figure 47. ^{19}F NMR (471 MHz, $CDCl_3$) spectrum for compound **3c**

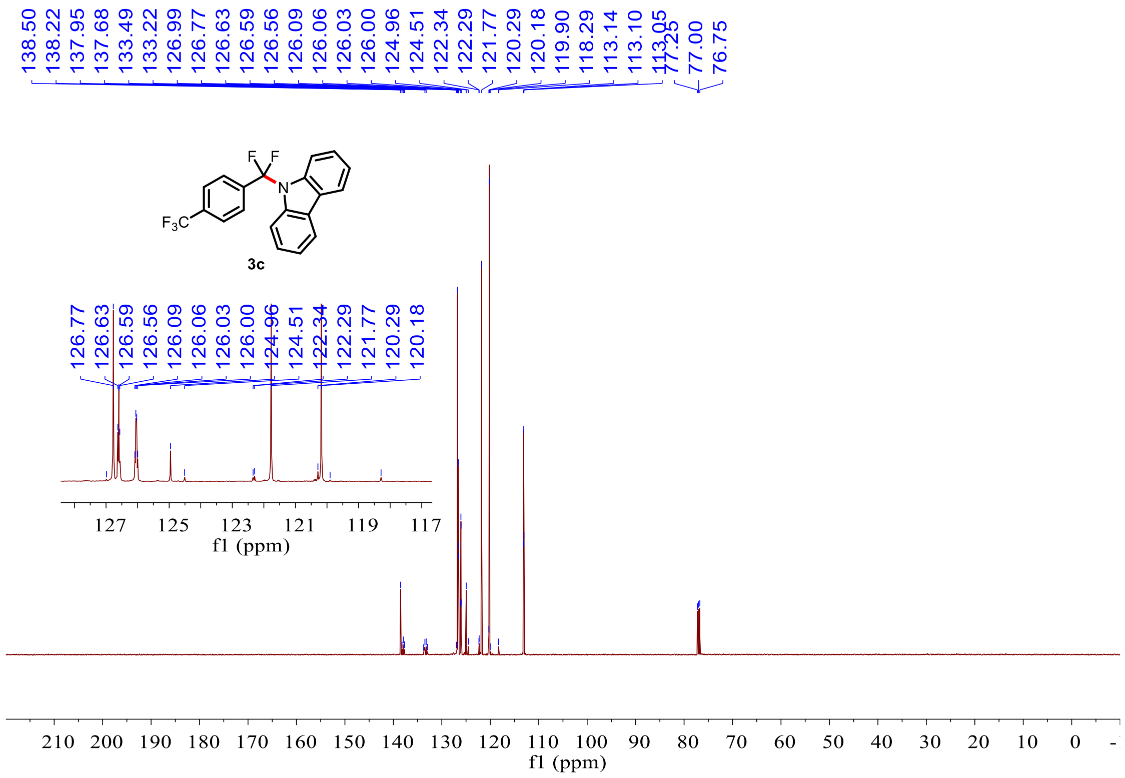

Supplementary Figure 48. ^{13}C NMR (126 MHz, $CDCl_3$) spectrum for compound **3c**

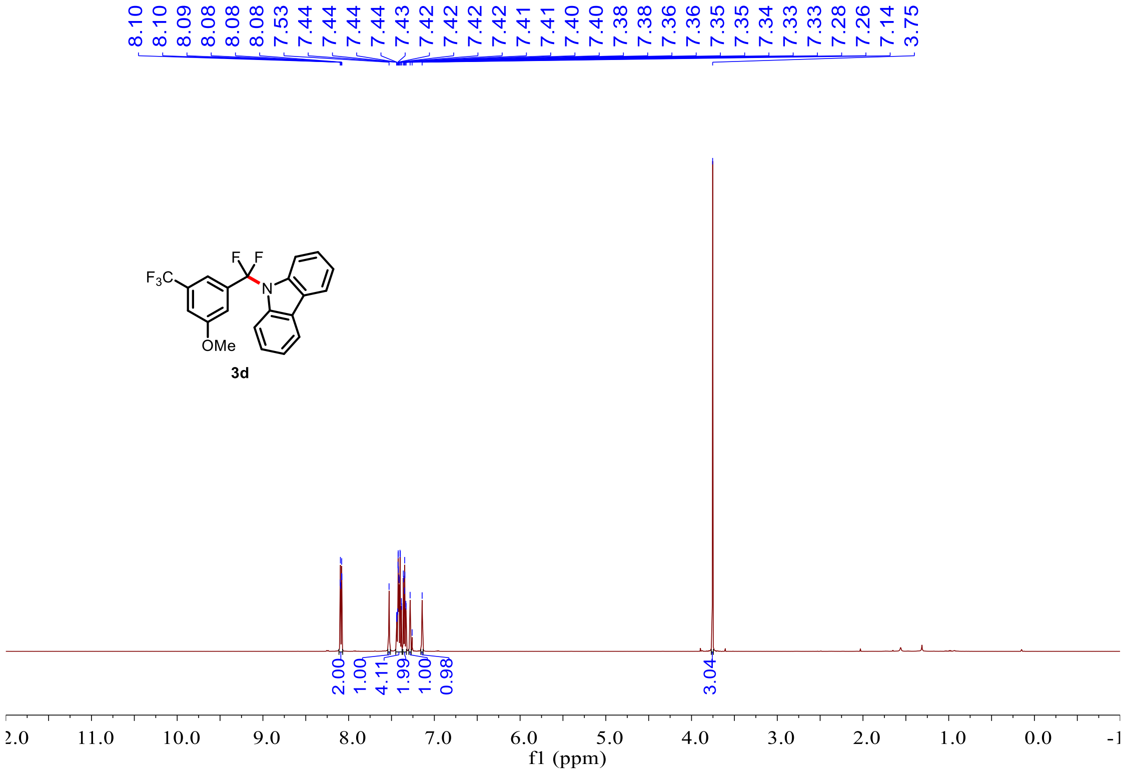

Supplementary Figure 49. ^{1}H NMR (500 MHz, $CDCl_3$) spectrum for compound **3d**

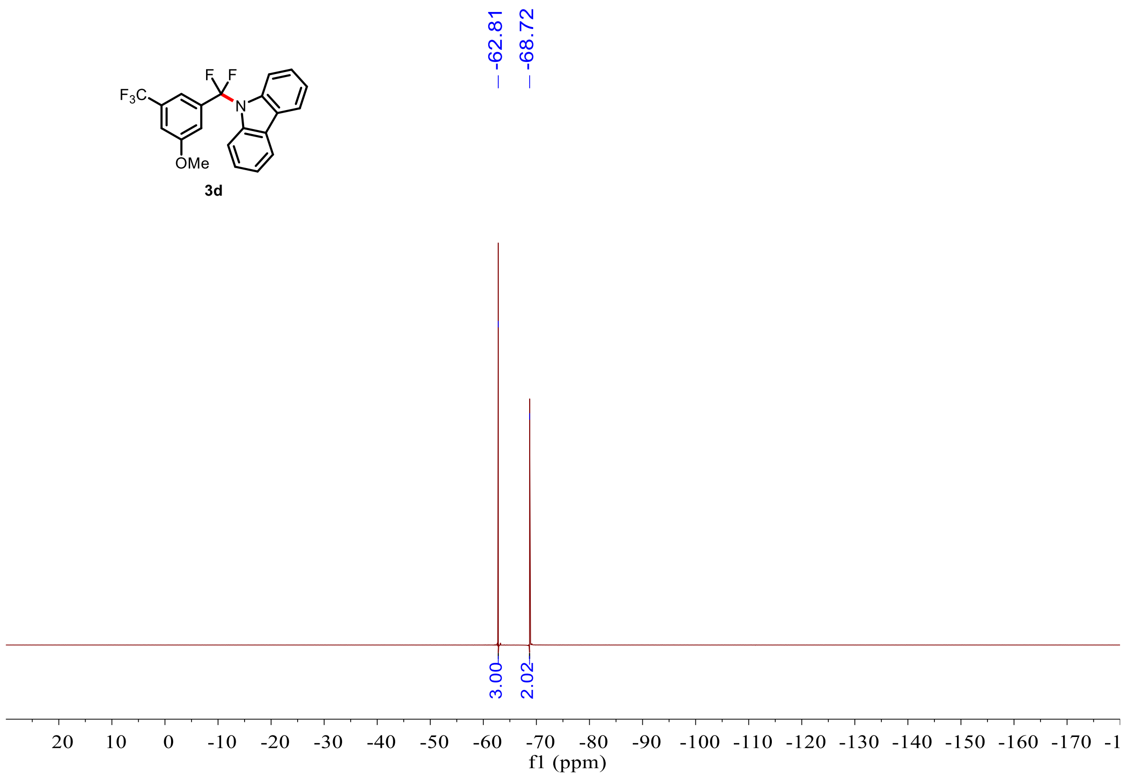

Supplementary Figure 50. **^{19}F NMR** (471 MHz, $CDCl_3$) spectrum for compound **3d**

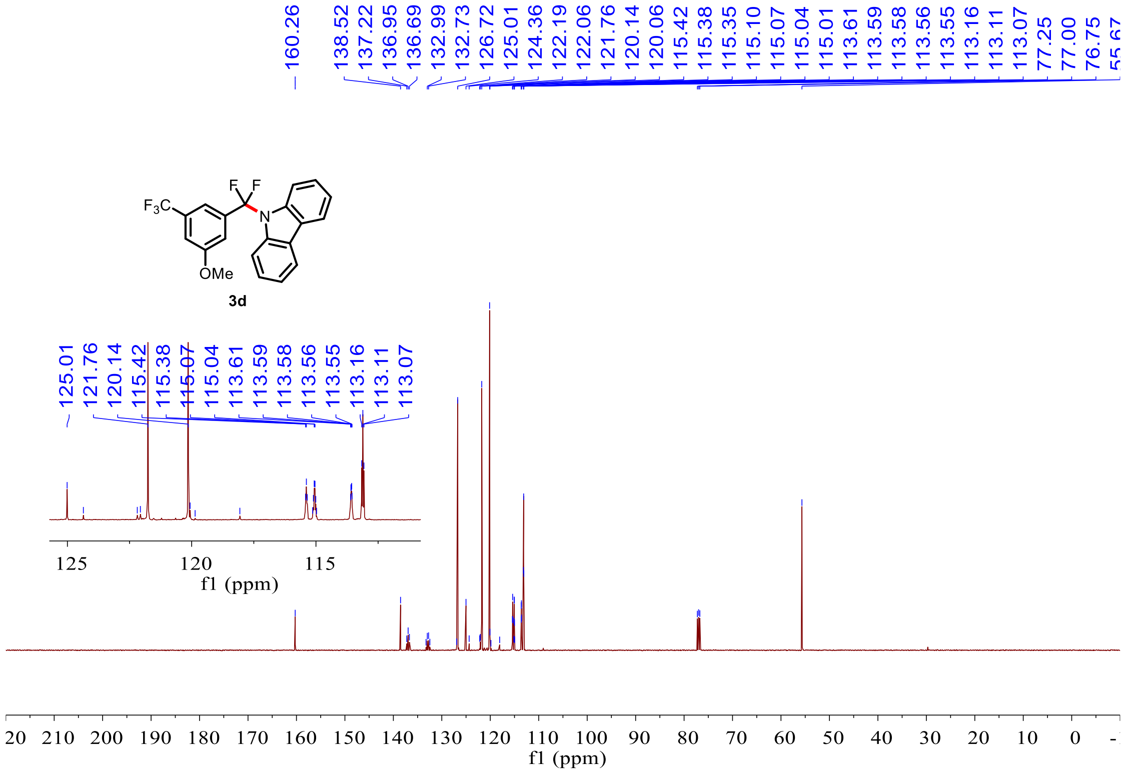

Supplementary Figure 51. **^{13}C NMR** (126 MHz, $CDCl_3$) spectrum for compound **3d**

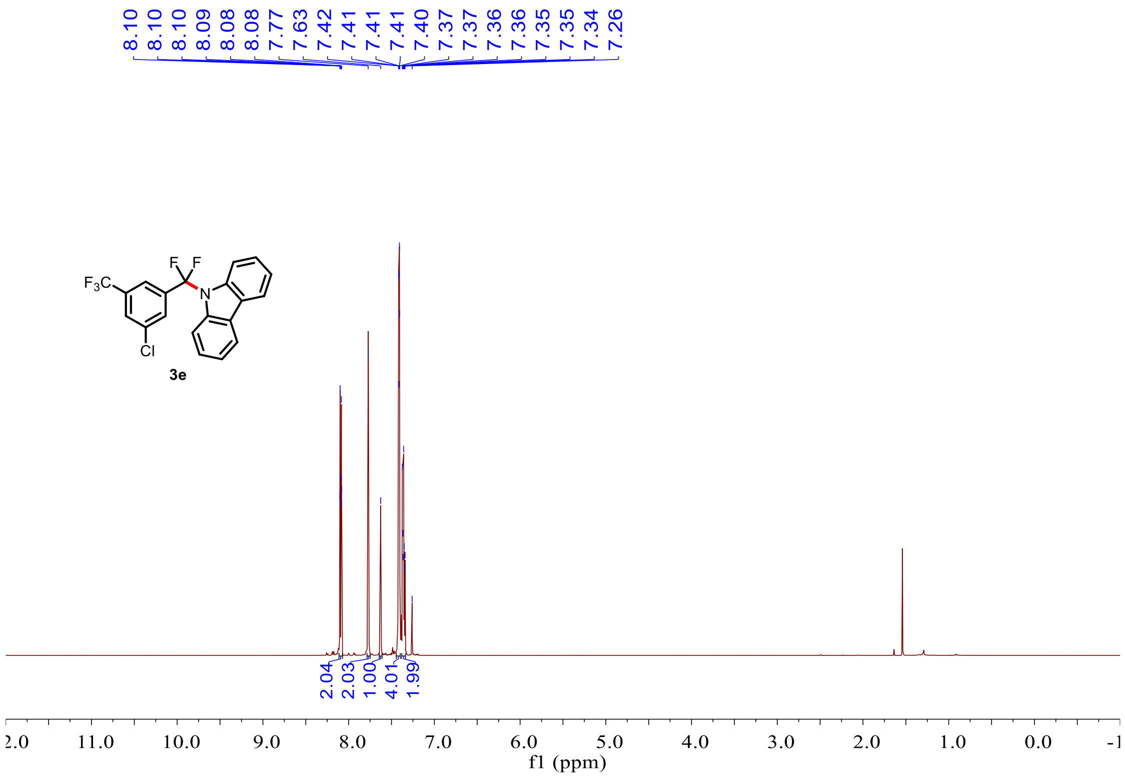

Supplementary Figure 52. **^{1}H NMR** (500 MHz, $CDCl_3$) spectrum for compound **3e**

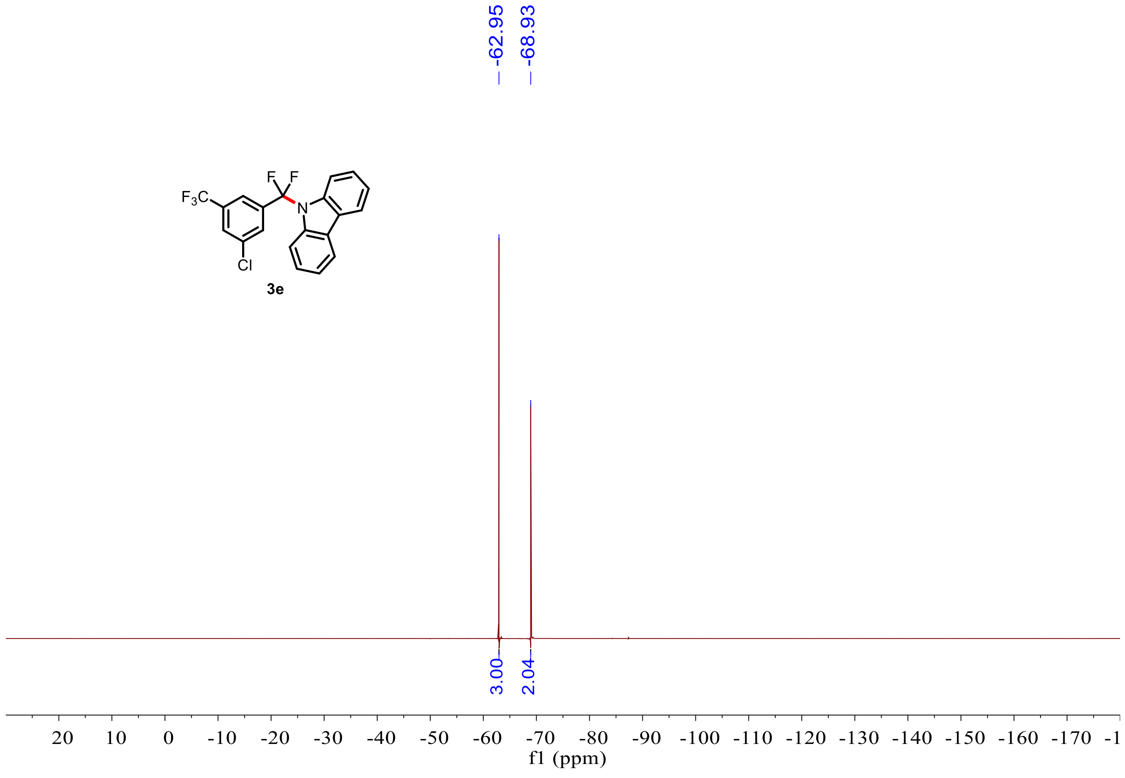

Supplementary Figure 53. **^{19}F NMR** (471 MHz, $CDCl_3$) spectrum for compound **3e**

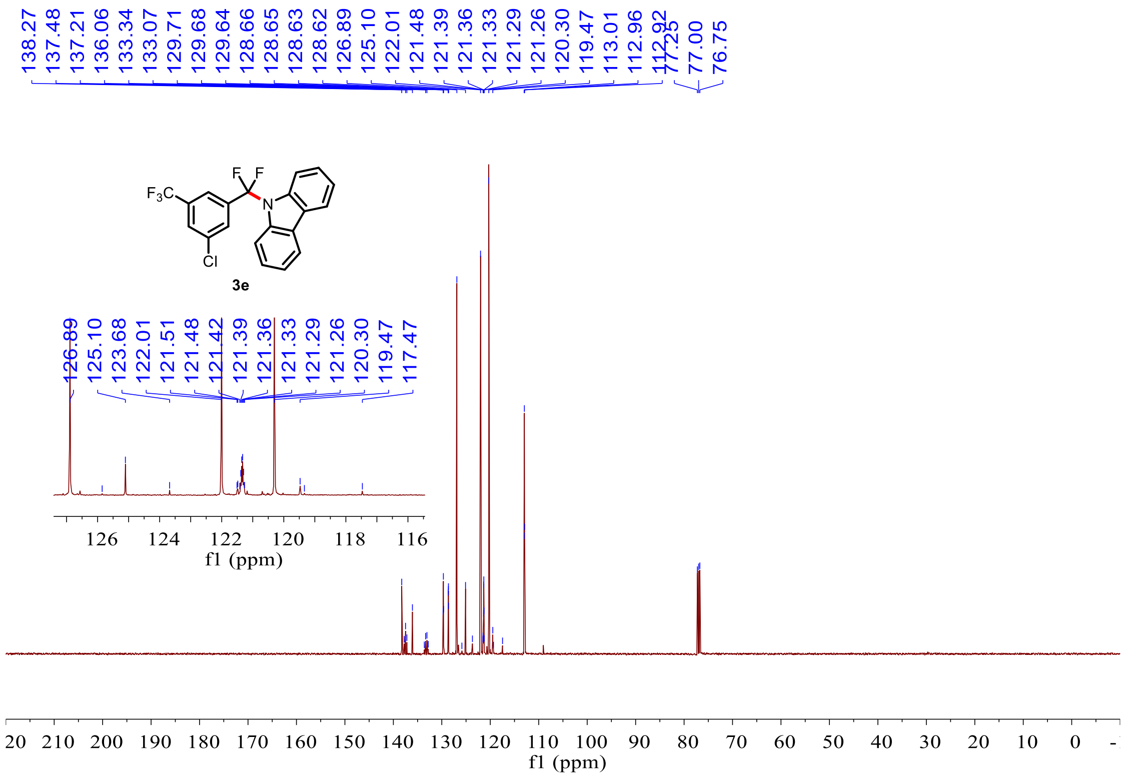

Supplementary Figure 54. ^{13}**C NMR** (126 MHz, $CDCl_3$) spectrum for compound **3e**

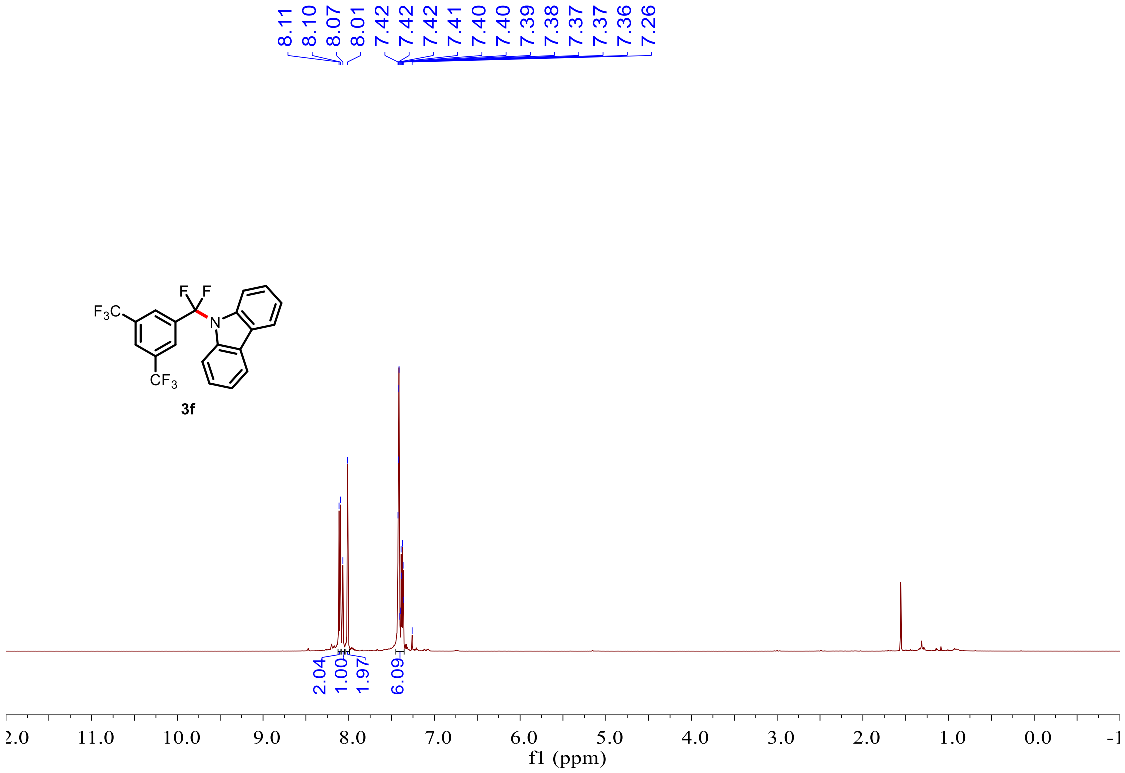

Supplementary Figure 55. ^{1}**H NMR** (500 MHz, $CDCl_3$) spectrum for compound **3f**

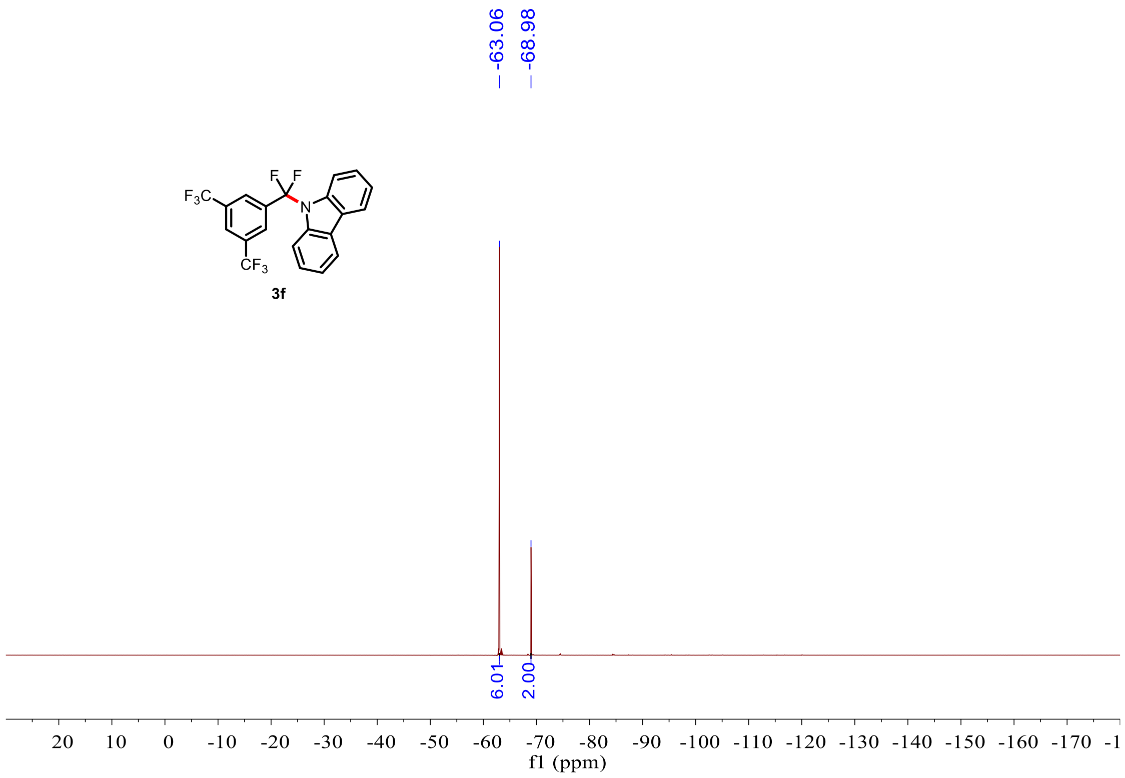

Supplementary Figure 56. ^{19}**F NMR** (471 MHz, $CDCl_3$) spectrum for compound **3f**

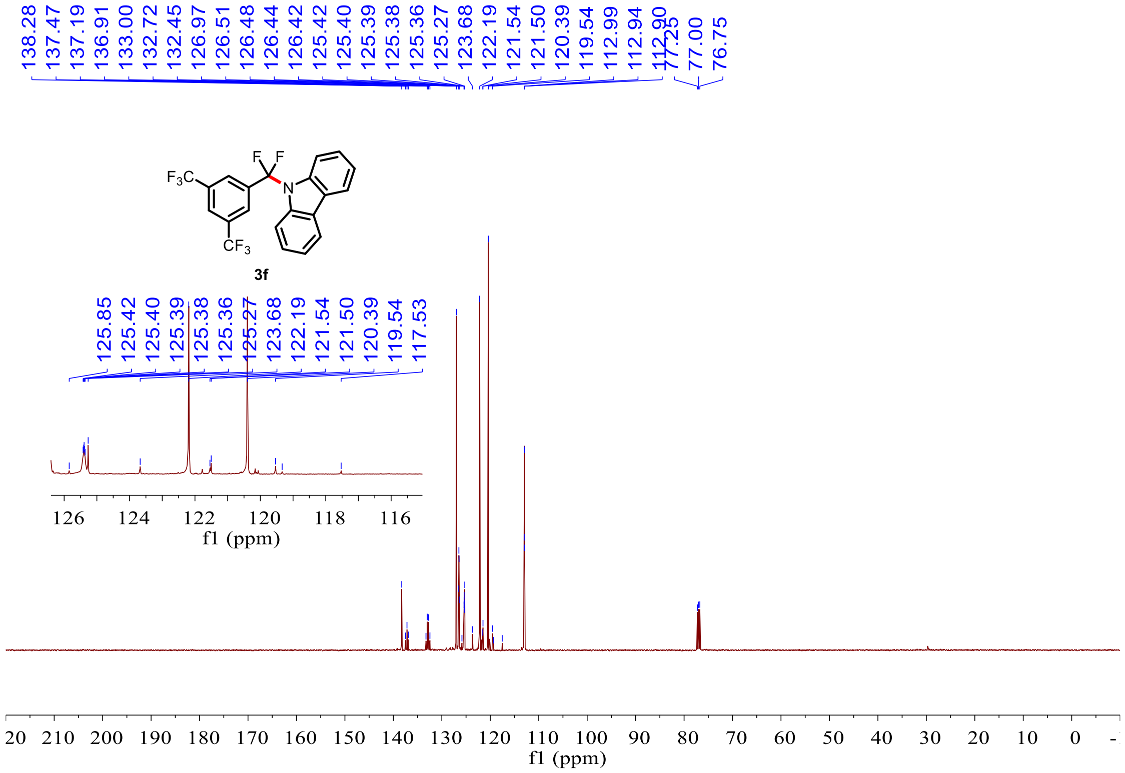

Supplementary Figure 57. ^{13}**C NMR** (126 MHz, $CDCl_3$) spectrum for compound **3f**

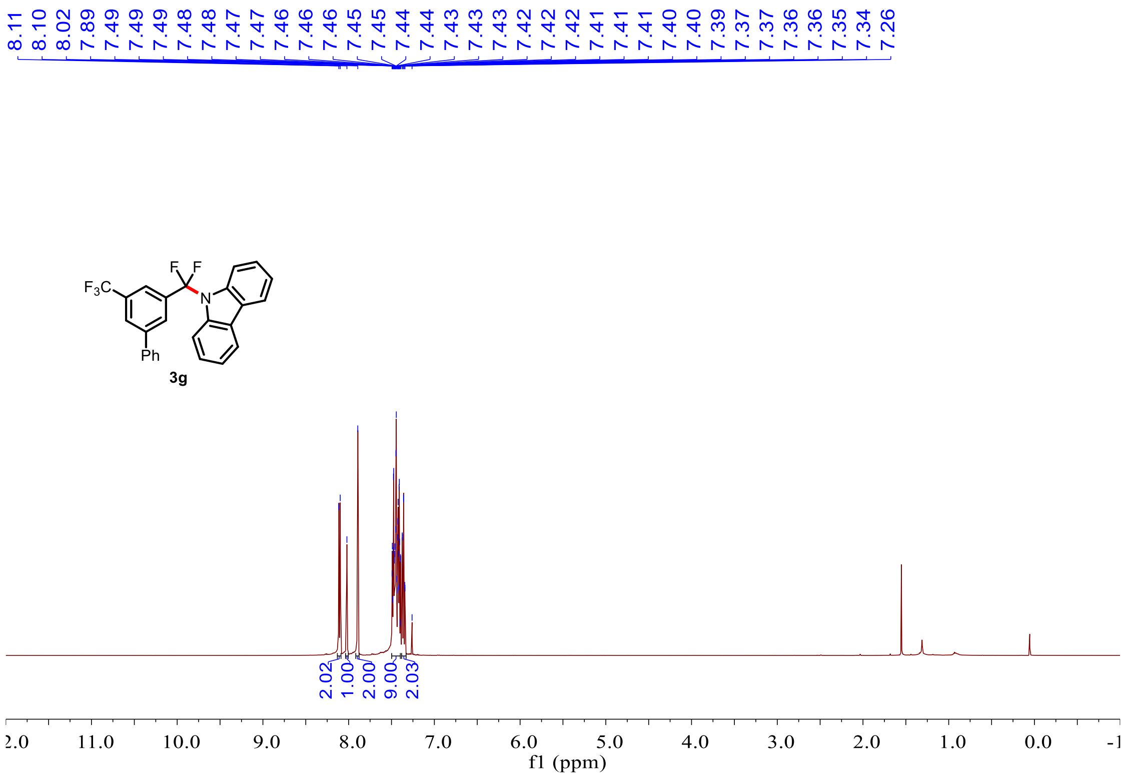

Supplementary Figure 58. ^{1}**H NMR** (500 MHz, $CDCl_3$) spectrum for compound **3g**

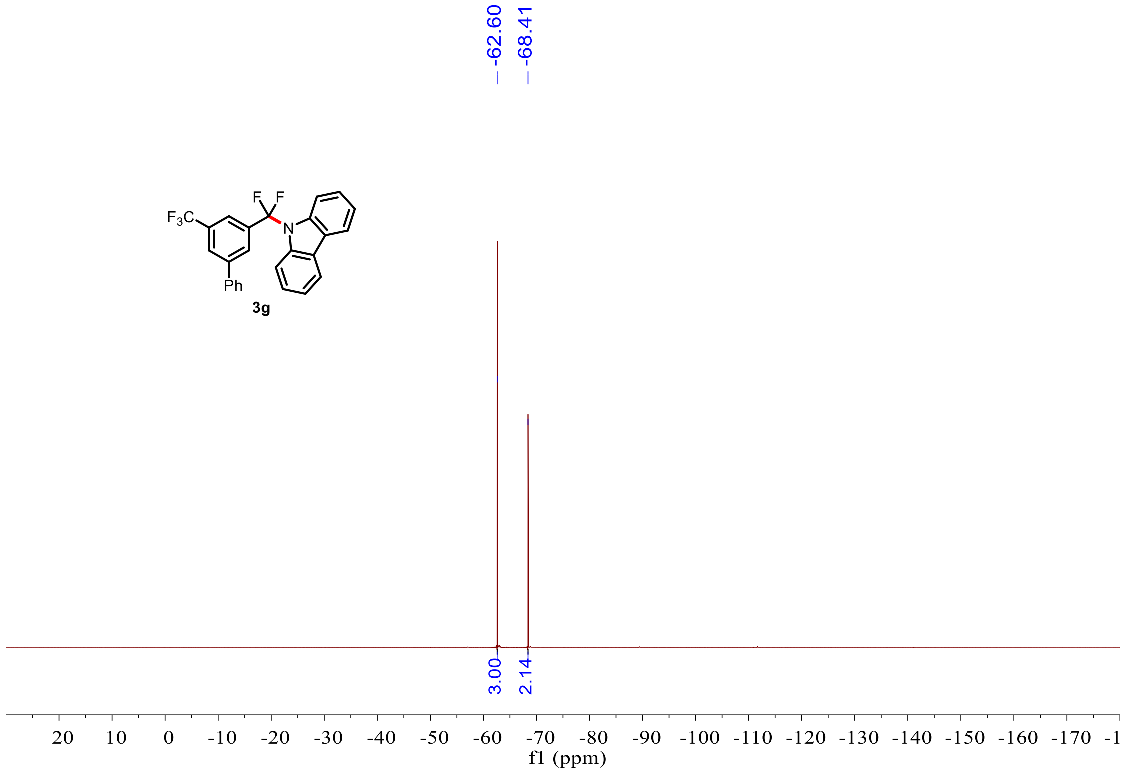

Supplementary Figure 59. ^{19}**F NMR** (471 MHz, $CDCl_3$) spectrum for compound **3g**

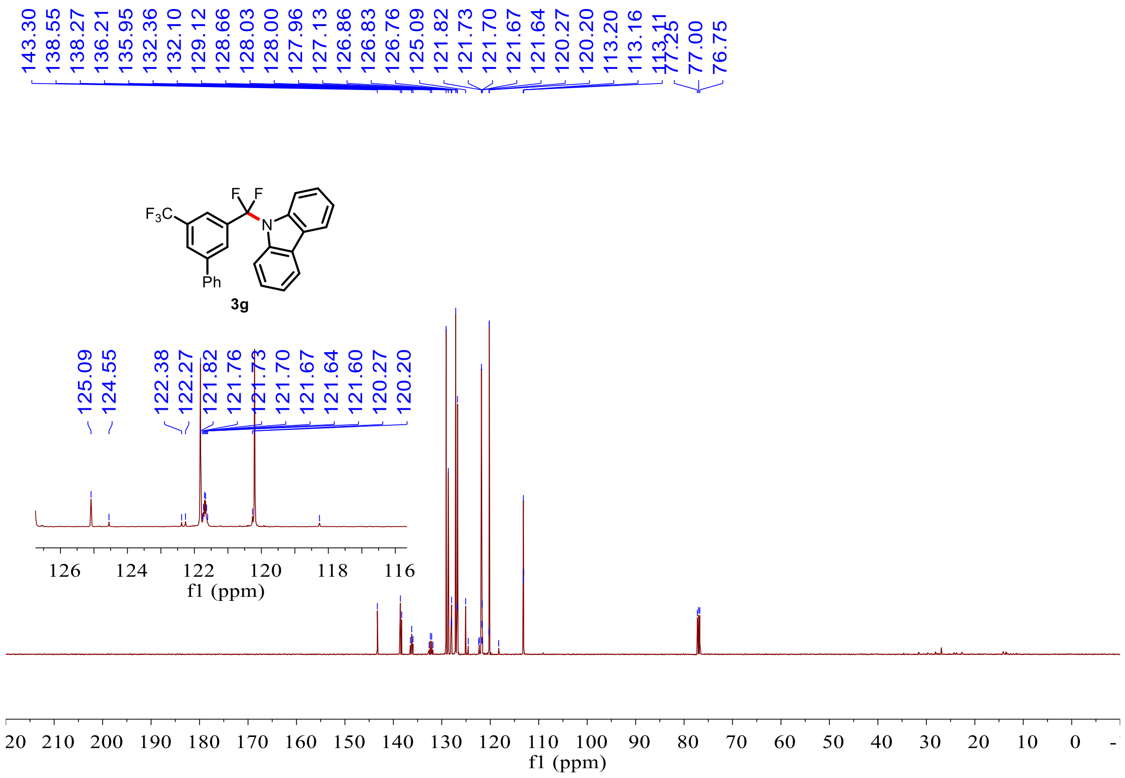

Supplementary Figure 60. ^{13}C NMR (126 MHz, $CDCl_3$) spectrum for compound **3g**

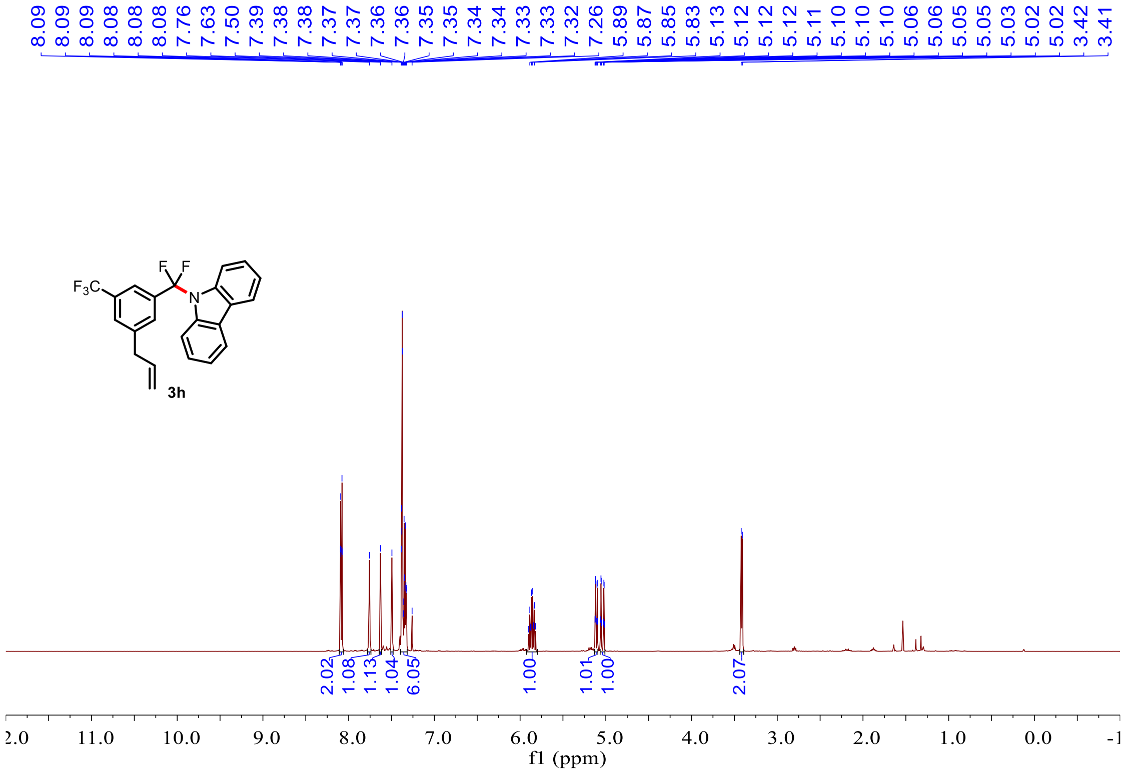

Supplementary Figure 61. ^{1}H NMR (500 MHz, $CDCl_3$) spectrum for compound **3h**

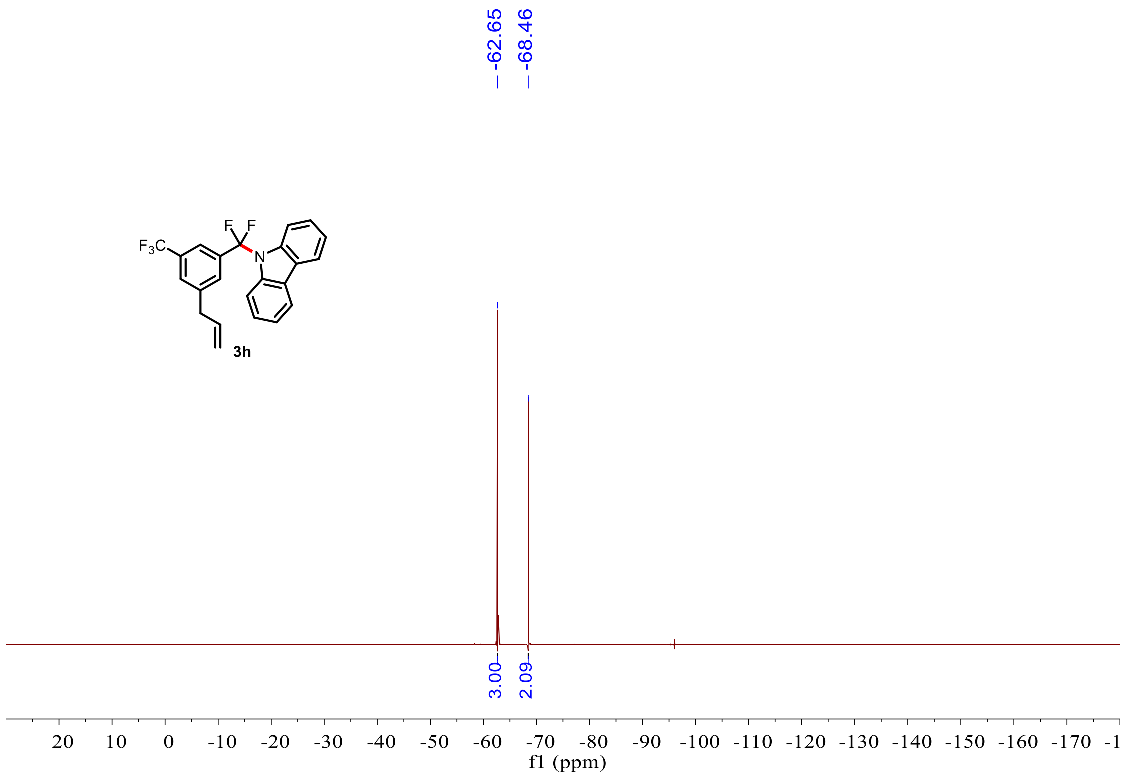

Supplementary Figure 62. ^{19}**F NMR** (471 MHz, $CDCl_3$) spectrum for compound **3h**

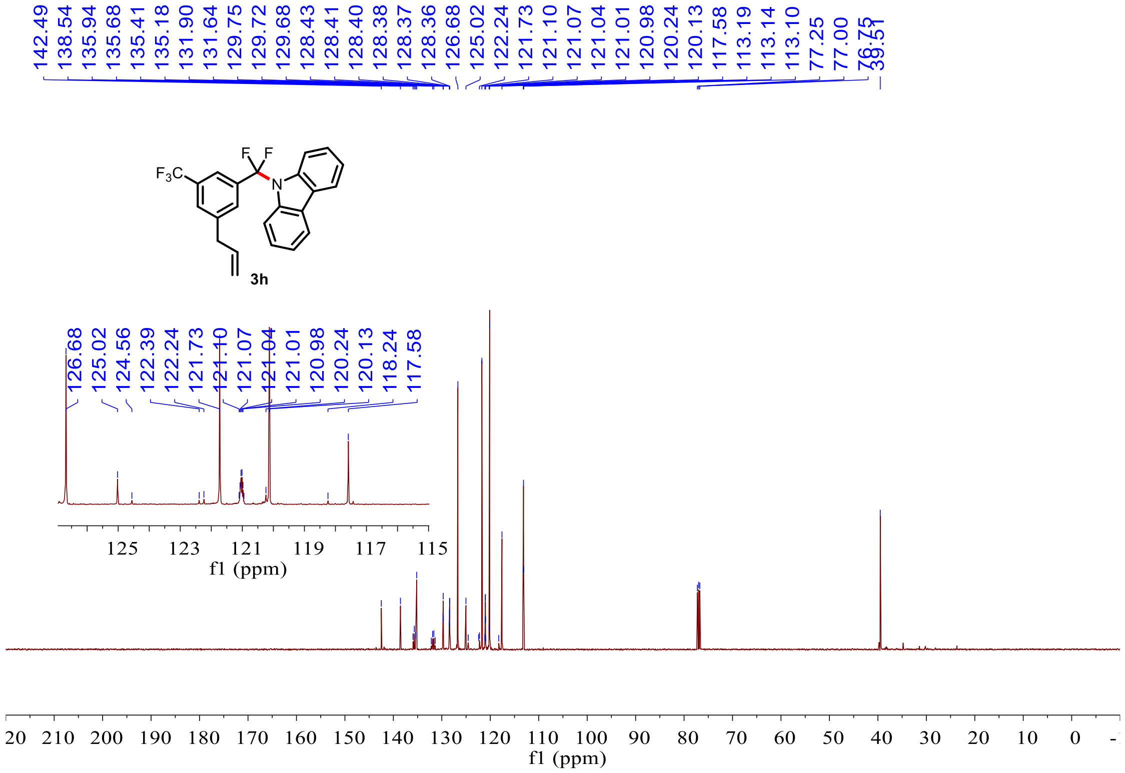

Supplementary Figure 63. ^{13}**C NMR** (126 MHz, $CDCl_3$) spectrum for compound **3h**

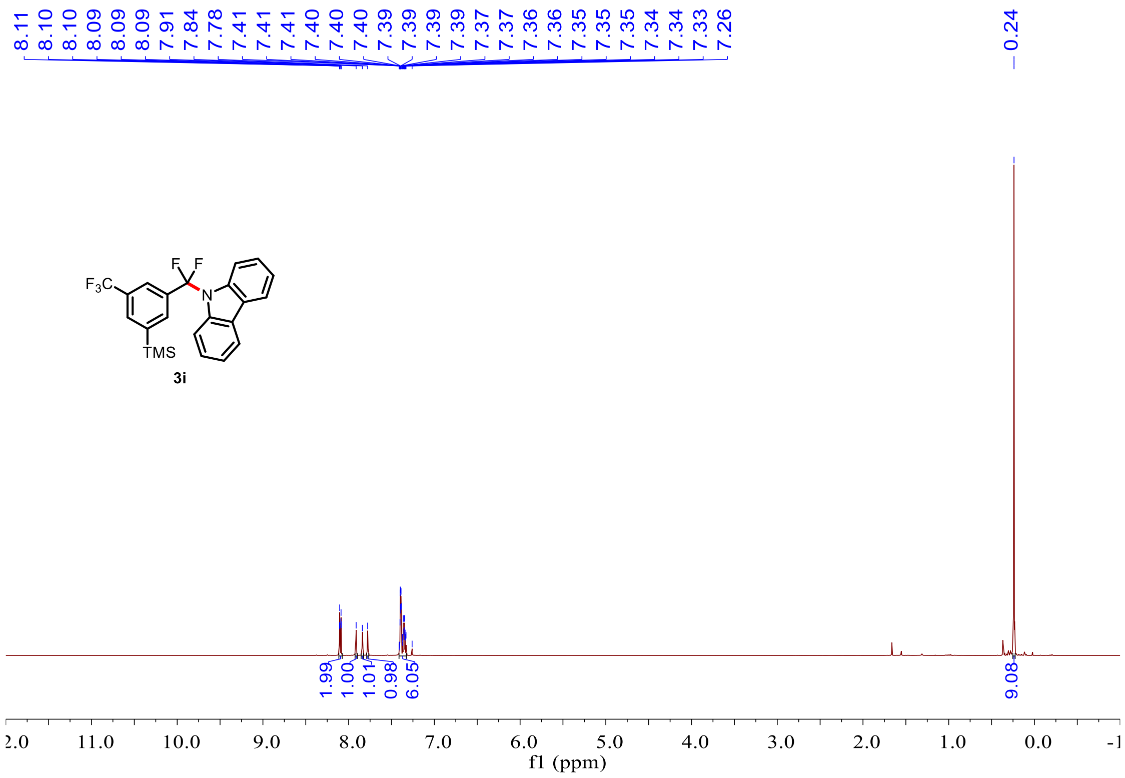

Supplementary Figure 64. ^{1}H NMR (500 MHz, $CDCl_3$) spectrum for compound **3i**

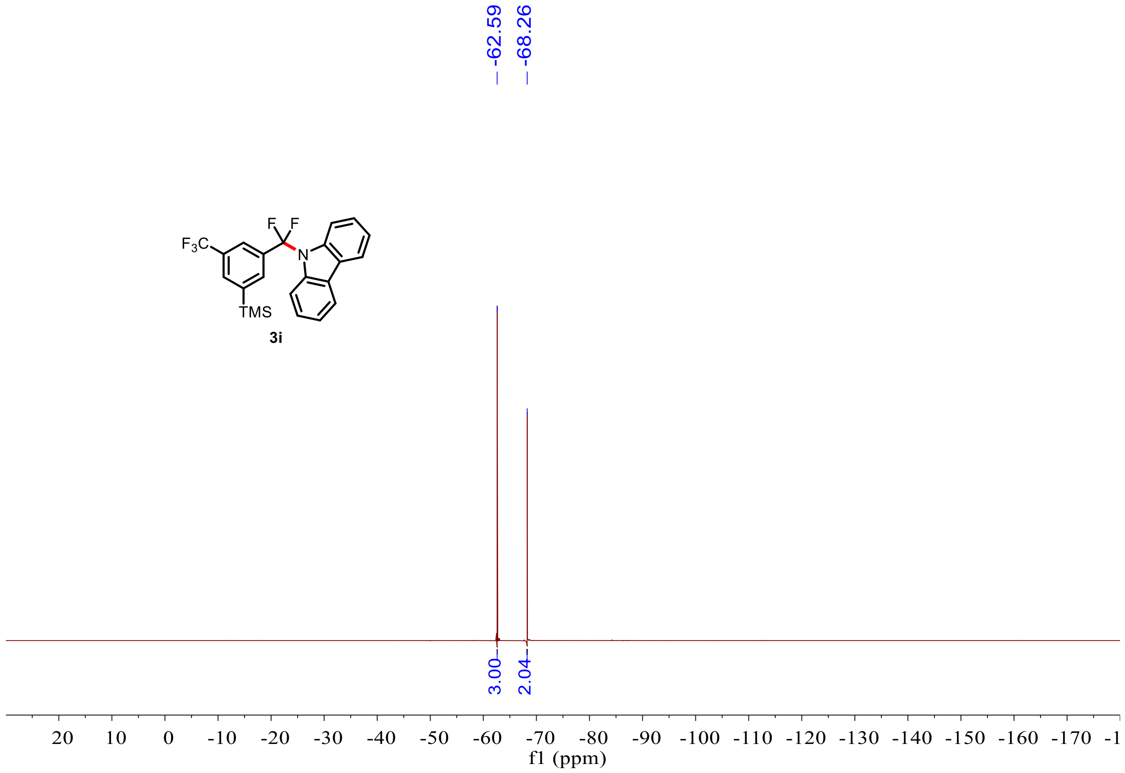

Supplementary Figure 65. ^{19}F NMR (471 MHz, $CDCl_3$) spectrum for compound **3i**

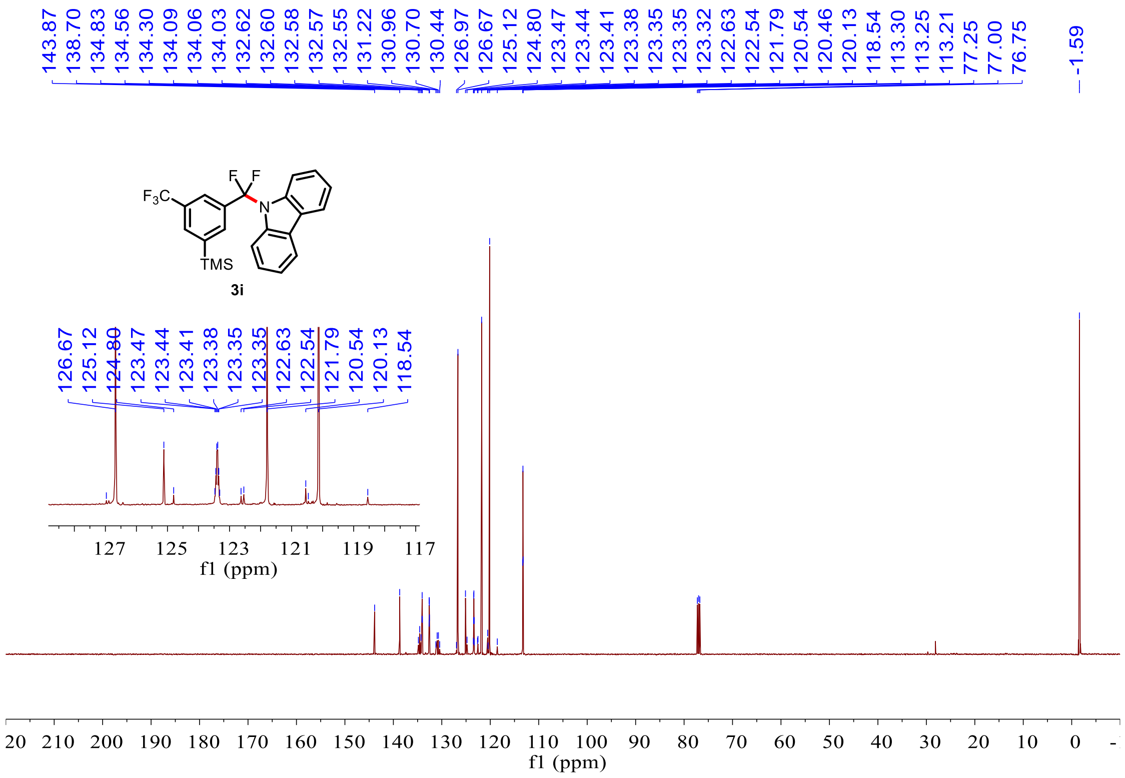

Supplementary Figure 66. ^{13}C NMR (126 MHz, $CDCl_3$) spectrum for compound **3i**

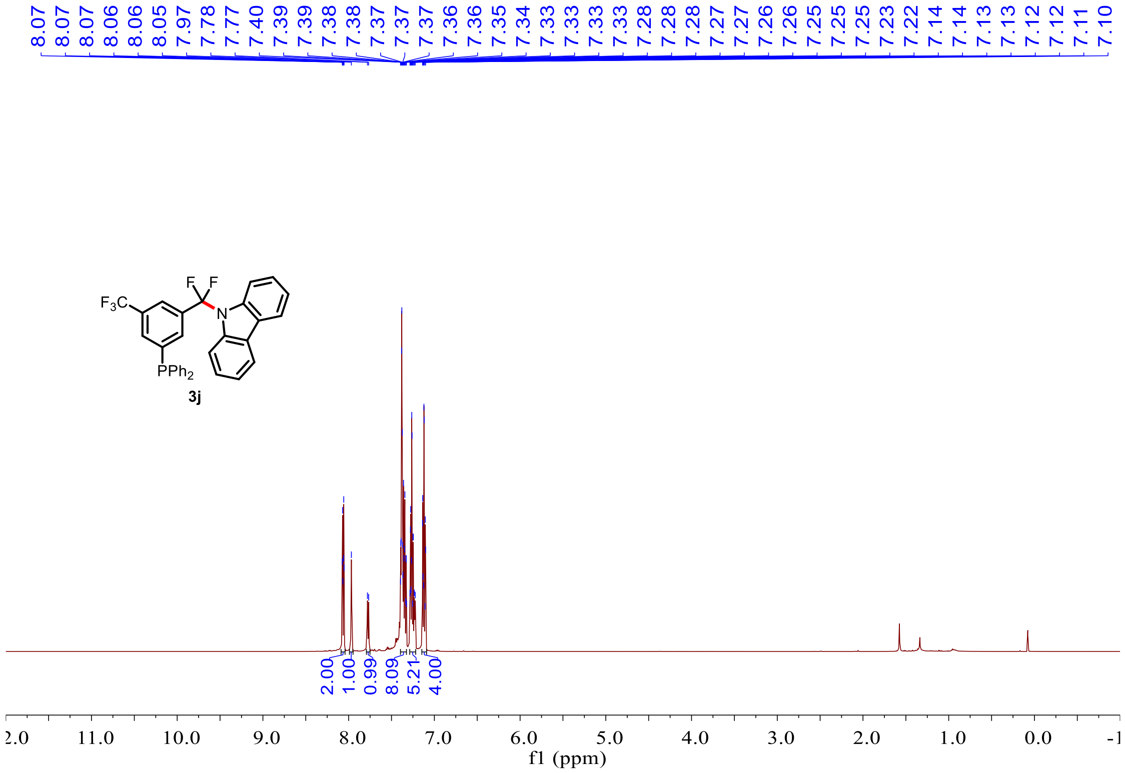

Supplementary Figure 67. ^{1}H NMR (500 MHz, $CDCl_3$) spectrum for compound **3j**

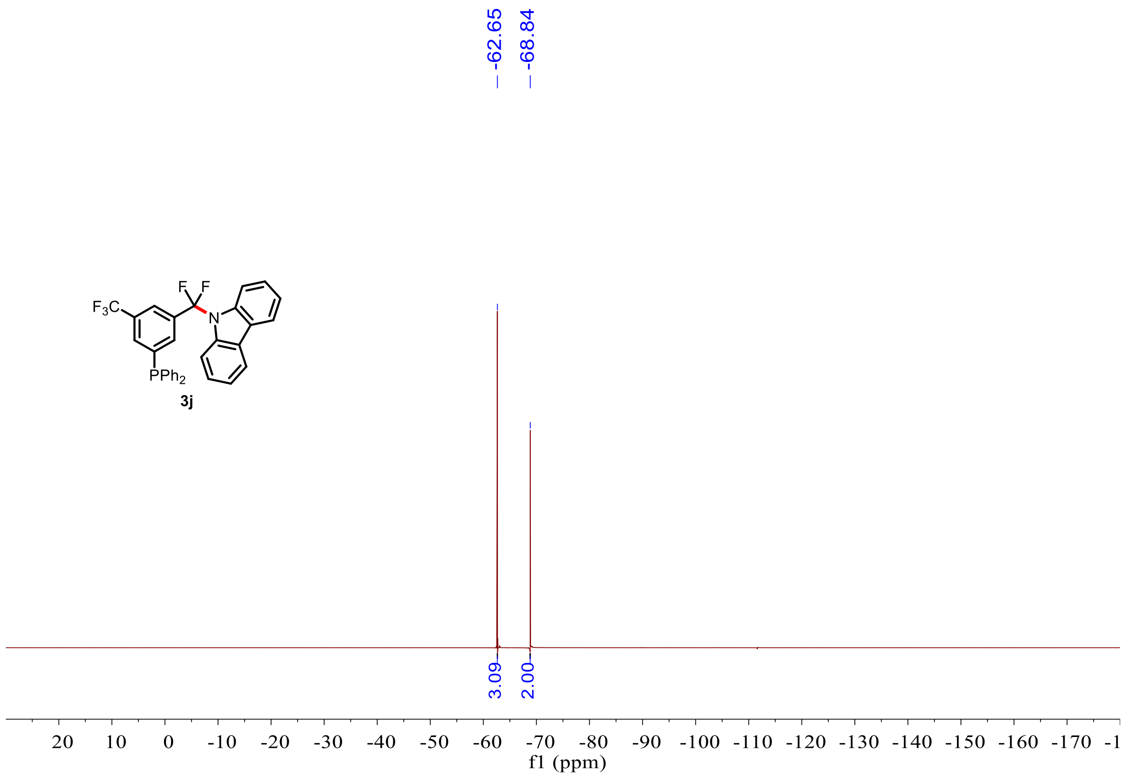

Supplementary Figure 68. ^{19}F NMR (471 MHz, $CDCl_3$) spectrum for compound **3j**

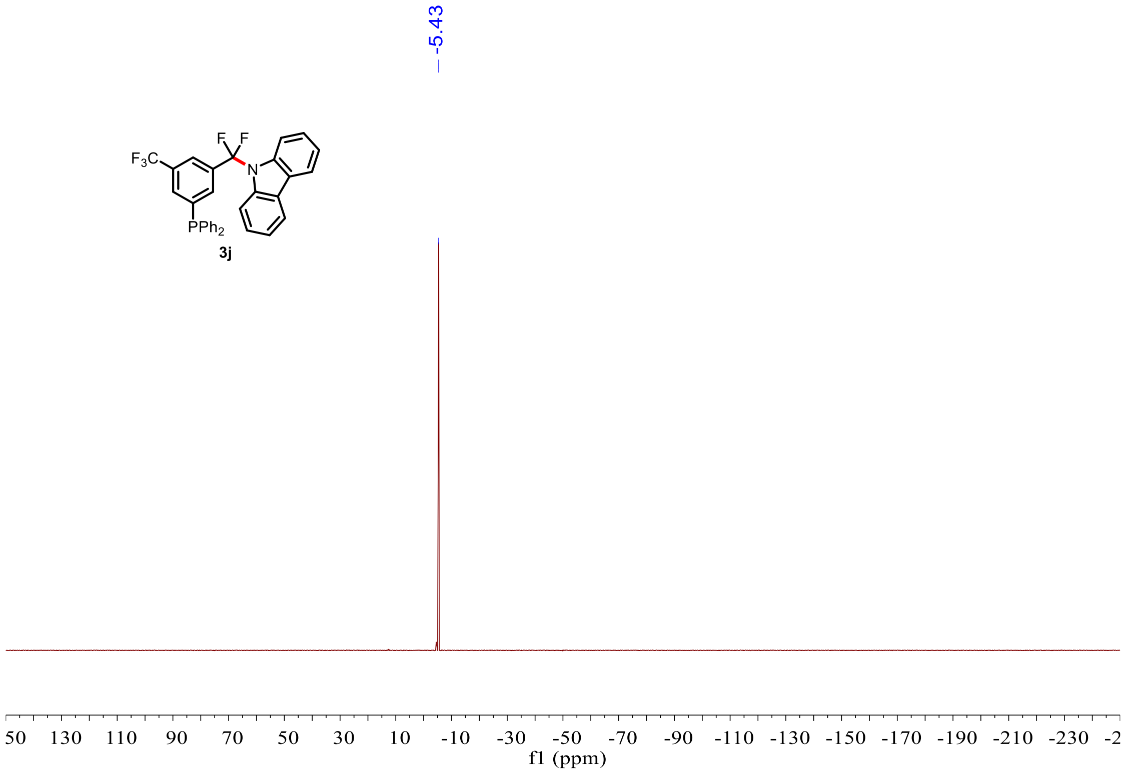

Supplementary Figure 69. ^{31}P NMR (202 MHz, $CDCl_3$) spectrum for compound **3j**

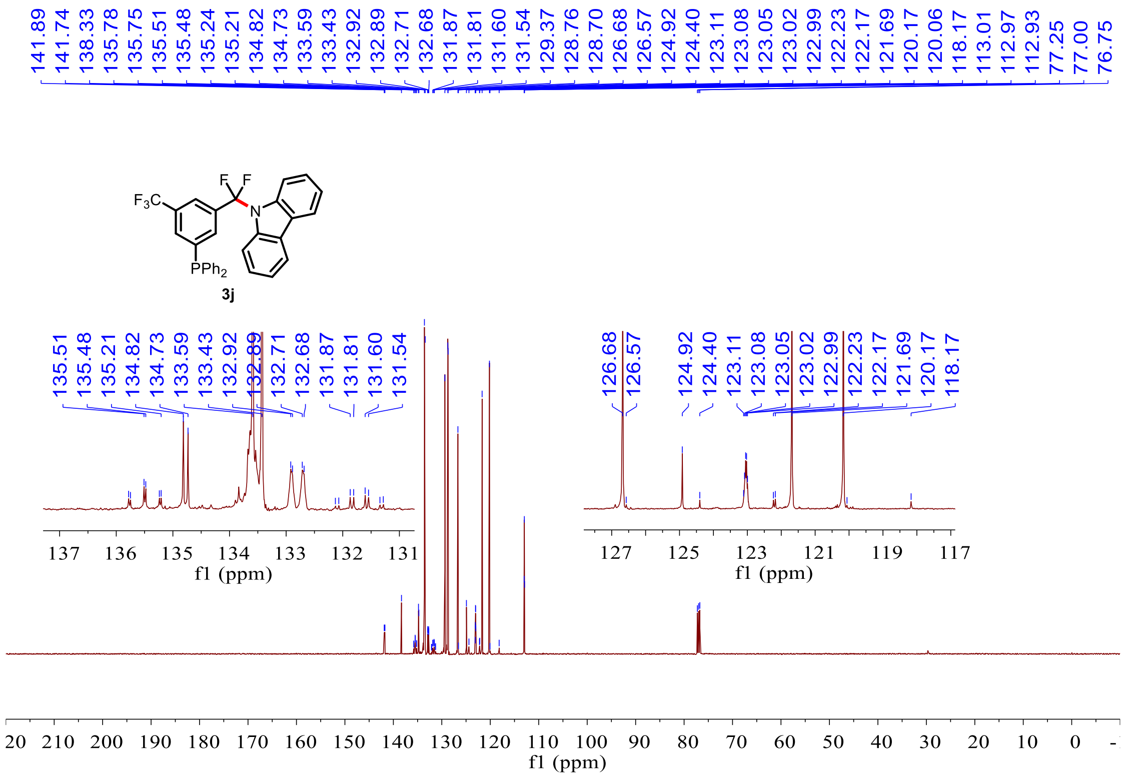

Supplementary Figure 70. ^{13}**C NMR** (126 MHz, $CDCl_3$) spectrum for compound **3j**

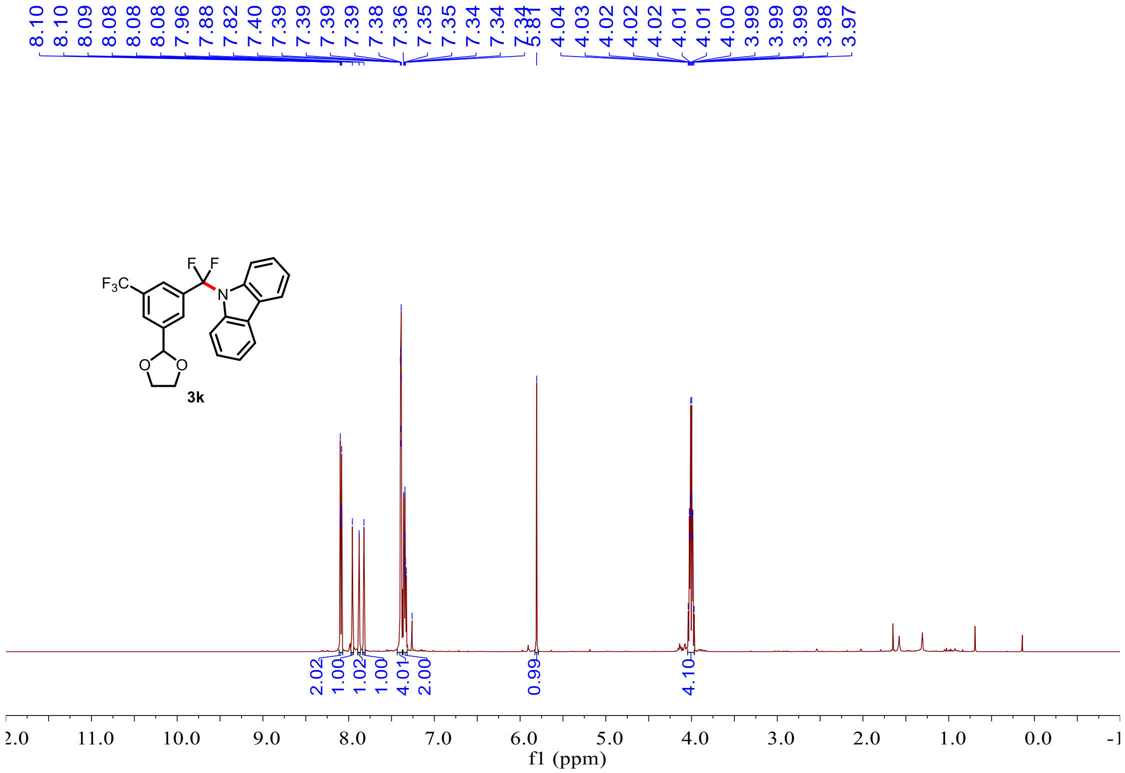

Supplementary Figure 71. ^{1}**H NMR** (500 MHz, $CDCl_3$) spectrum for compound **3k**

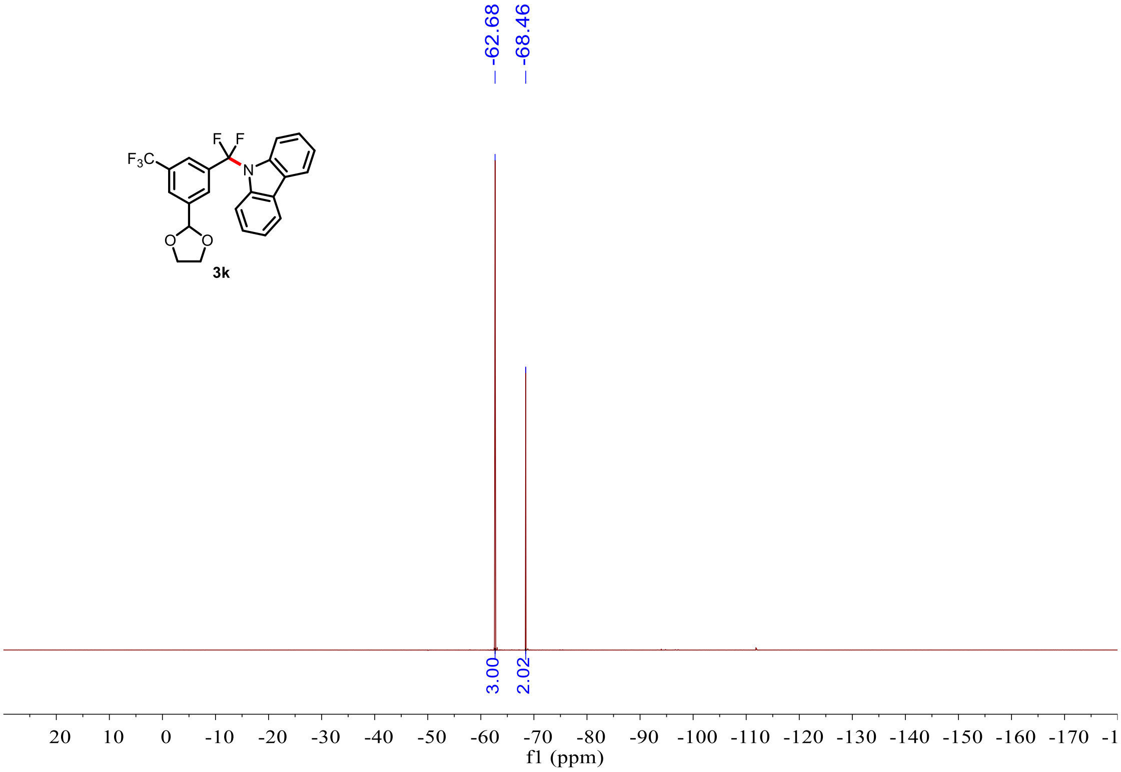

Supplementary Figure 72. ^{19}**F NMR** (471 MHz, $CDCl_3$) spectrum for compound **3k**

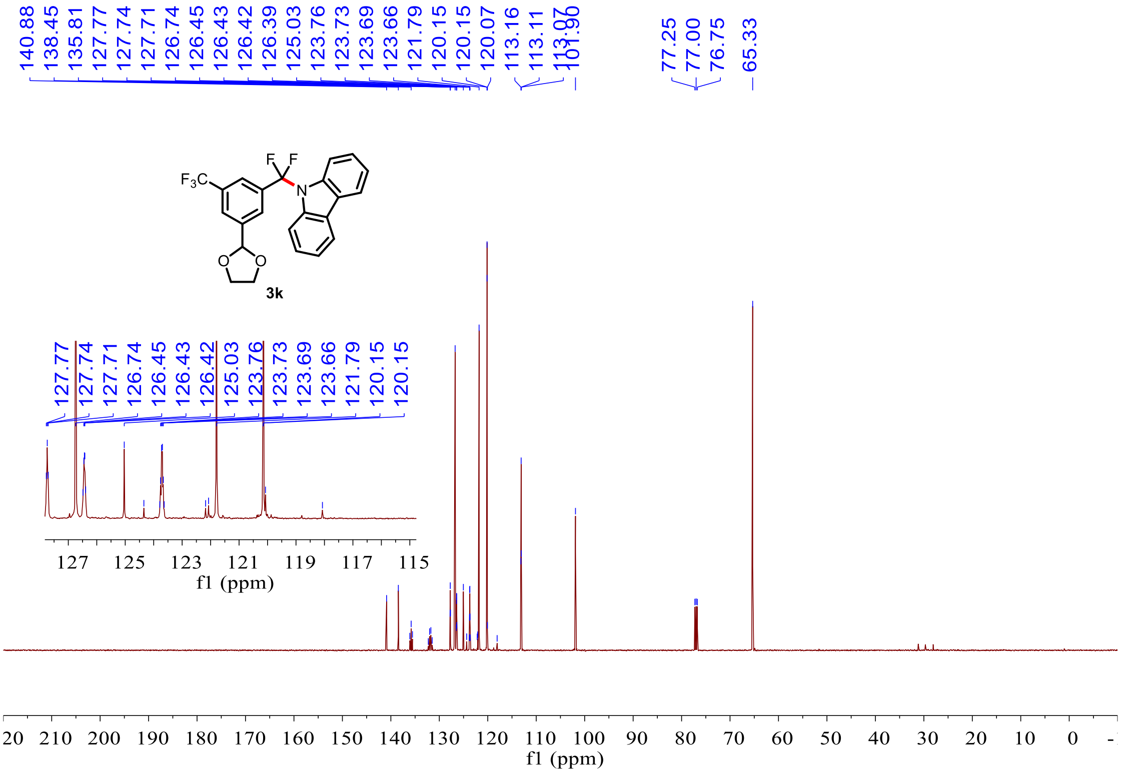

Supplementary Figure 73. ^{13}**C NMR** (126 MHz, $CDCl_3$) spectrum for compound **3k**

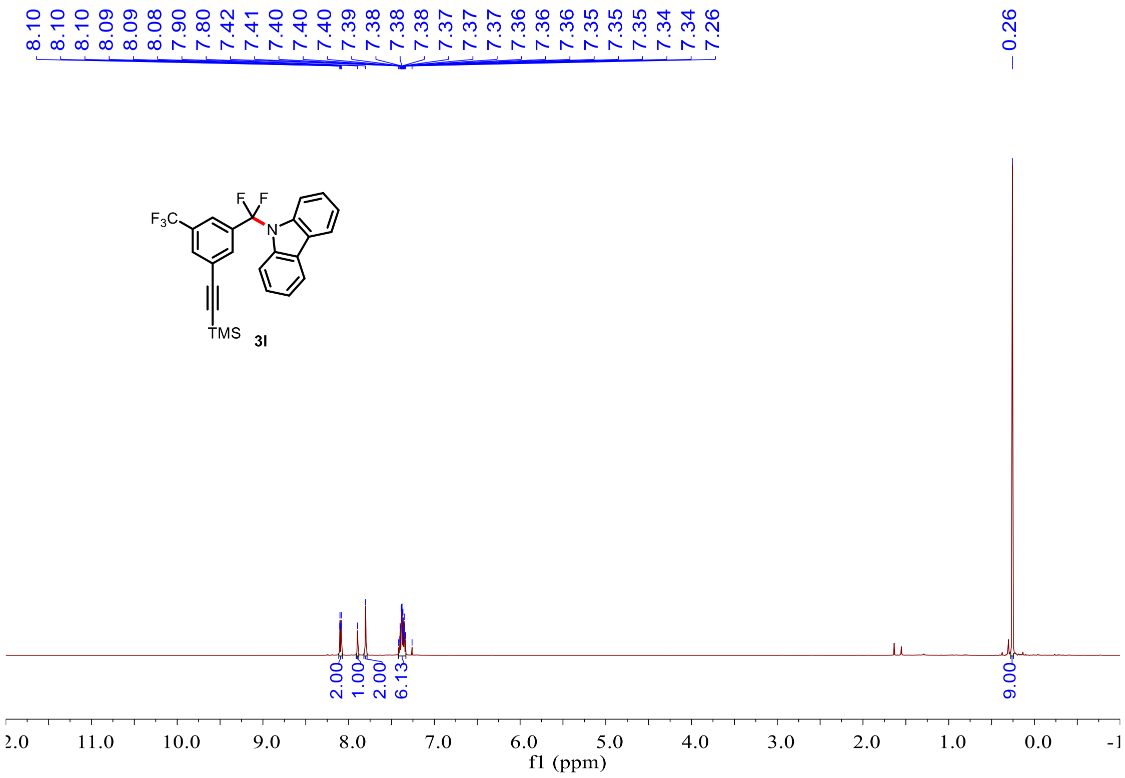

Supplementary Figure 74. ^{1}**H NMR** (500 MHz, $CDCl_3$) spectrum for compound **3l**

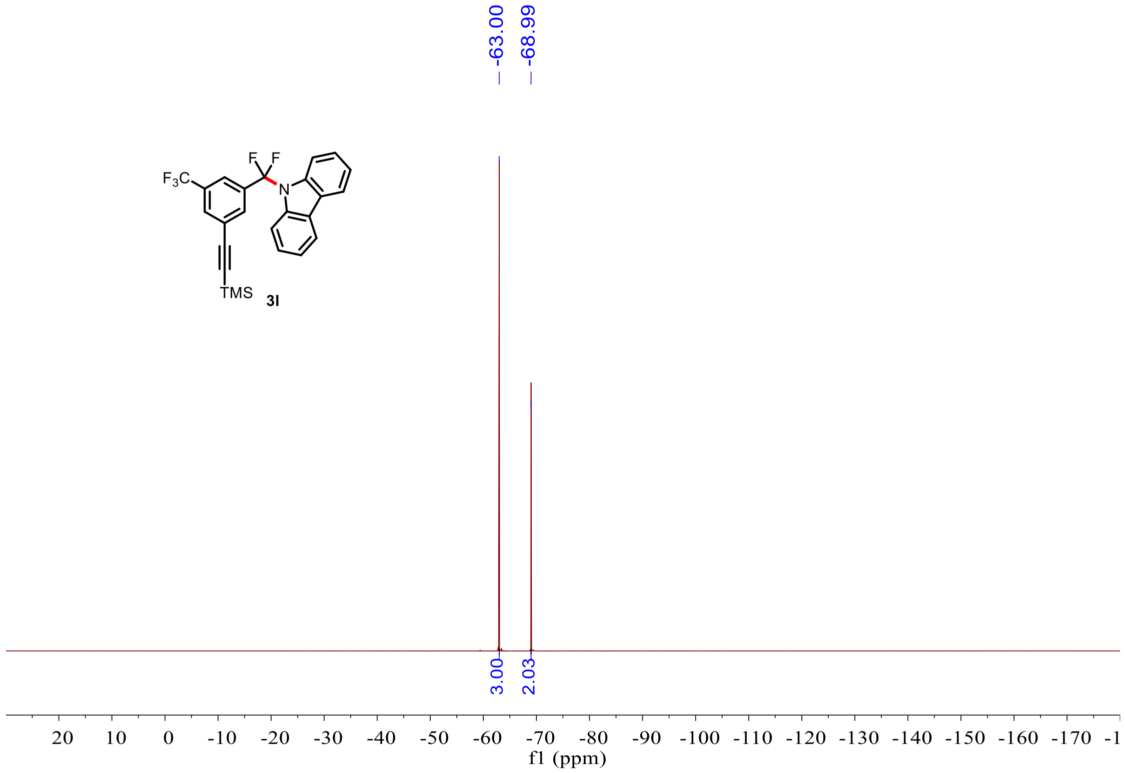

Supplementary Figure 75. ^{19}**F NMR** (471 MHz, $CDCl_3$) spectrum for compound **3l**

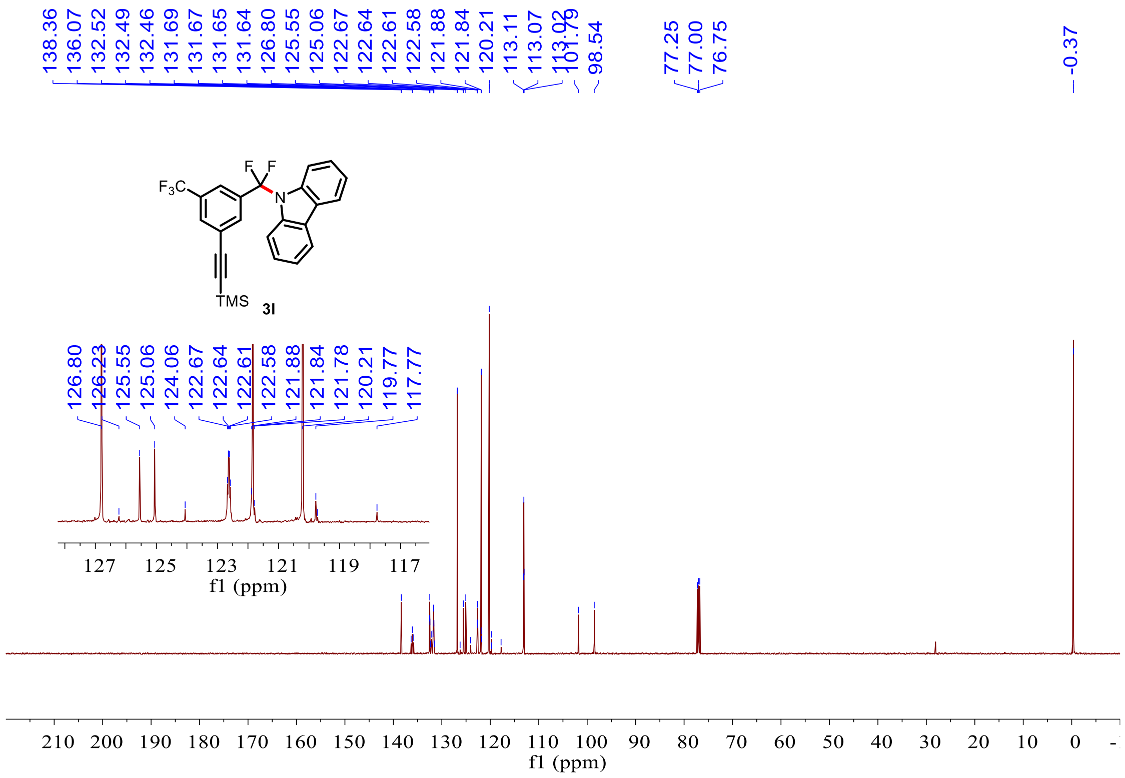

Supplementary Figure 76. ^{13}C NMR (126 MHz, $CDCl_3$) spectrum for compound **3l**

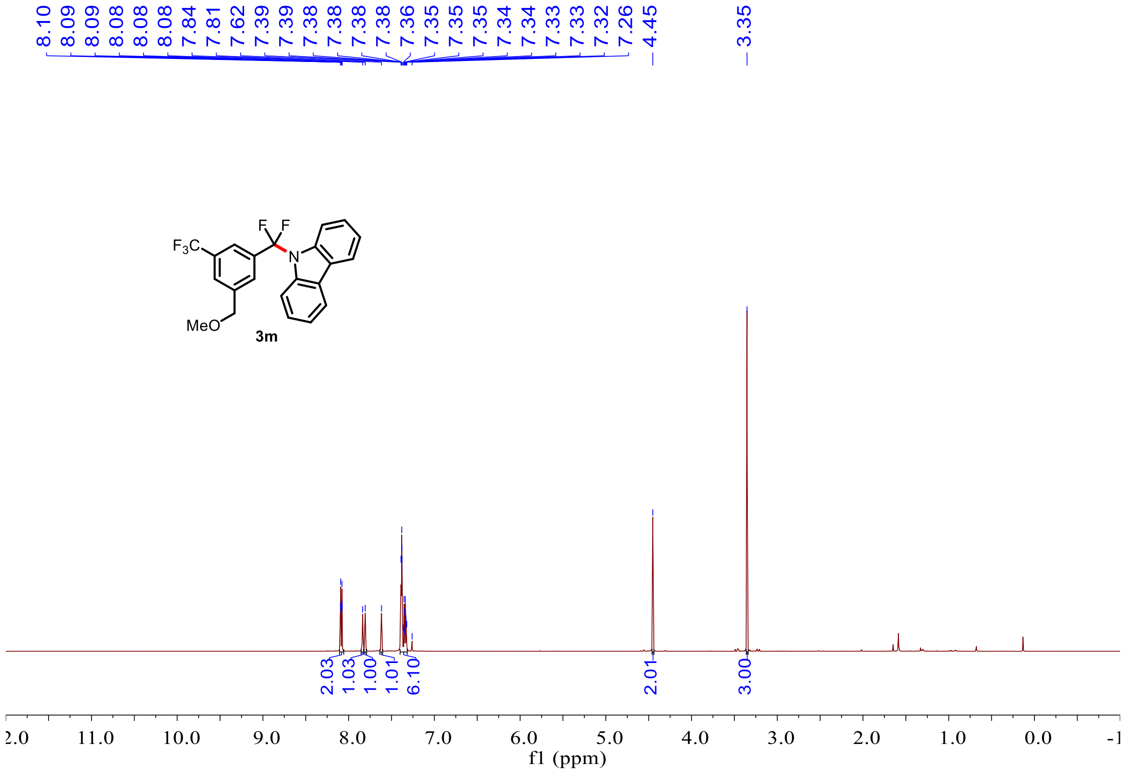

Supplementary Figure 77. ^{1}H NMR (500 MHz, $CDCl_3$) spectrum for compound **3m**

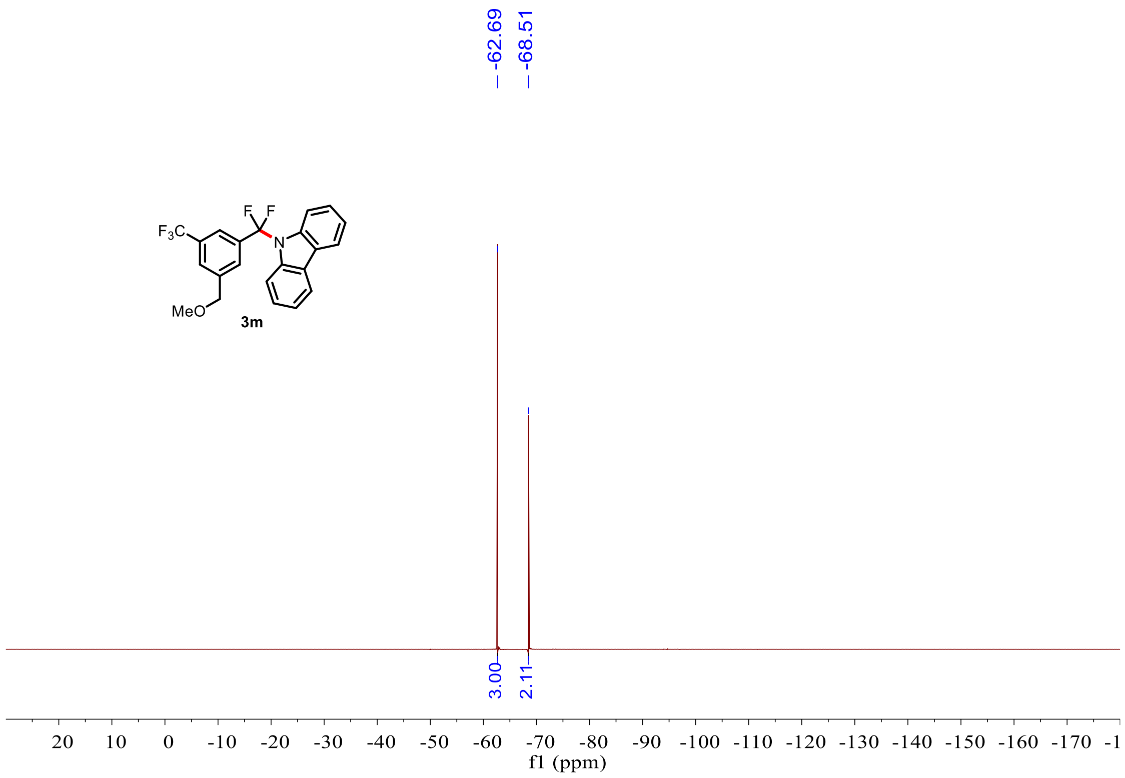

Supplementary Figure 78. ^{19}F NMR (471 MHz, $CDCl_3$) spectrum for compound **3m**

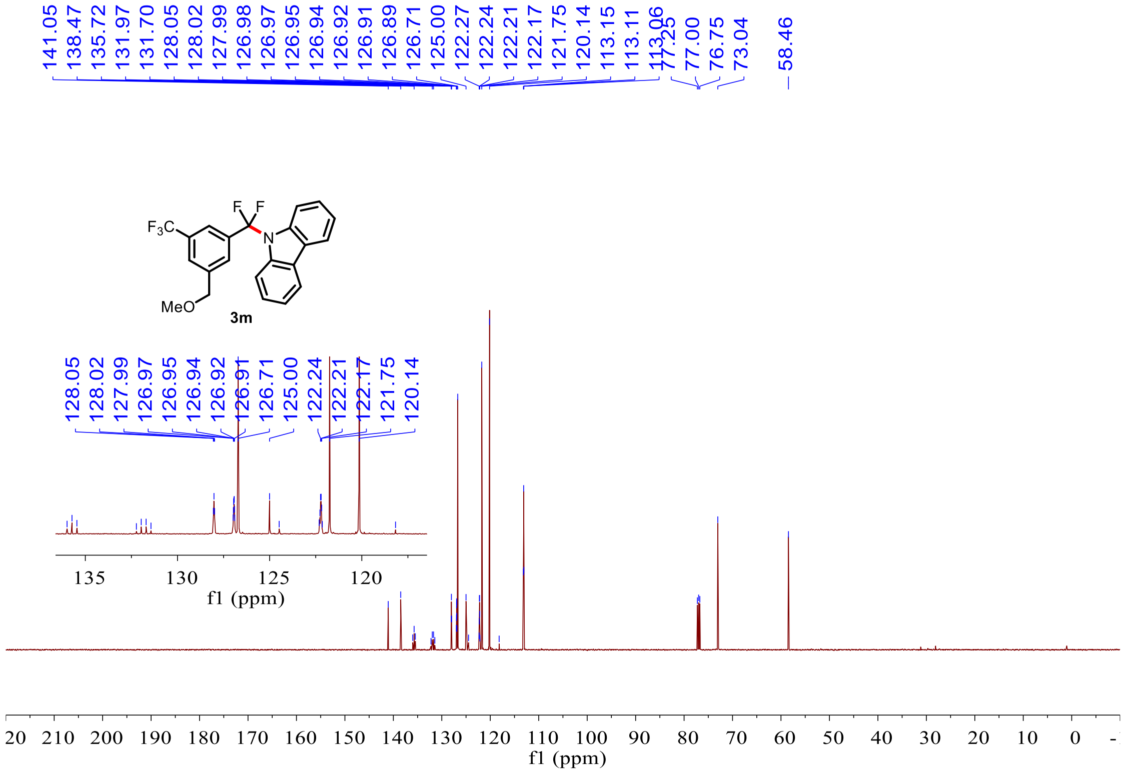

Supplementary Figure 79. ^{13}C NMR (126 MHz, $CDCl_3$) spectrum for compound **3m**

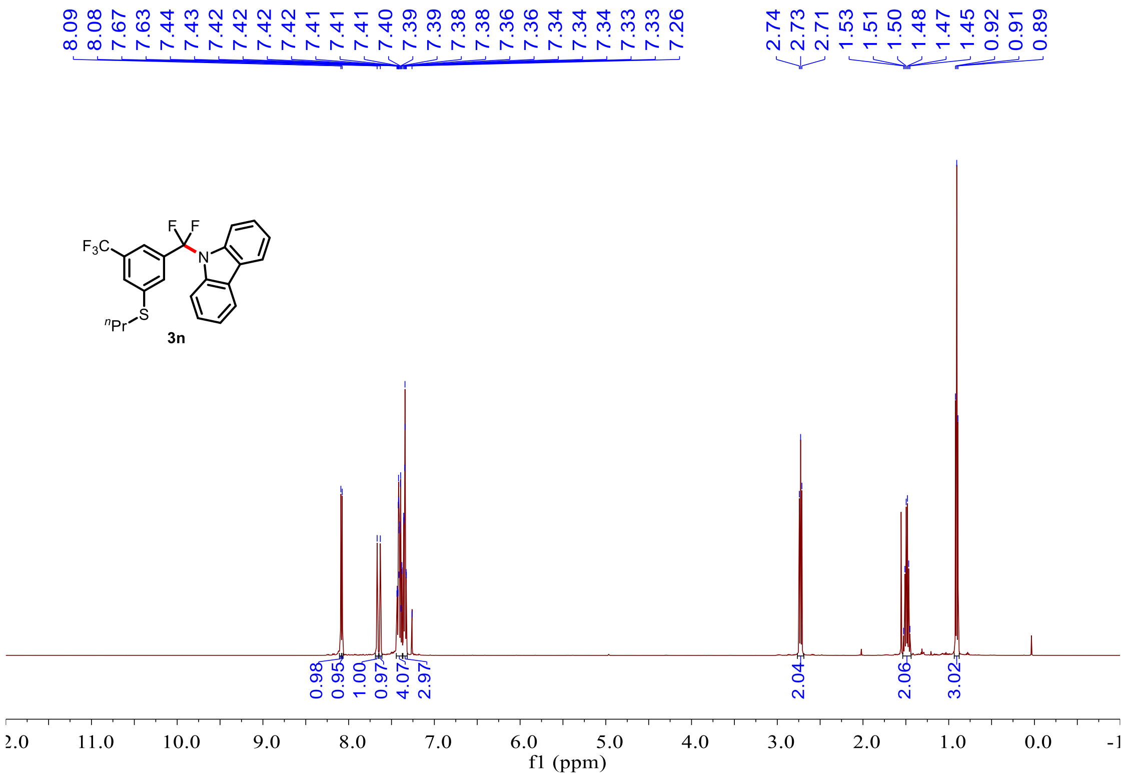

Supplementary Figure 80. **^{1}H NMR** (500 MHz, $CDCl_3$) spectrum for compound **3n**

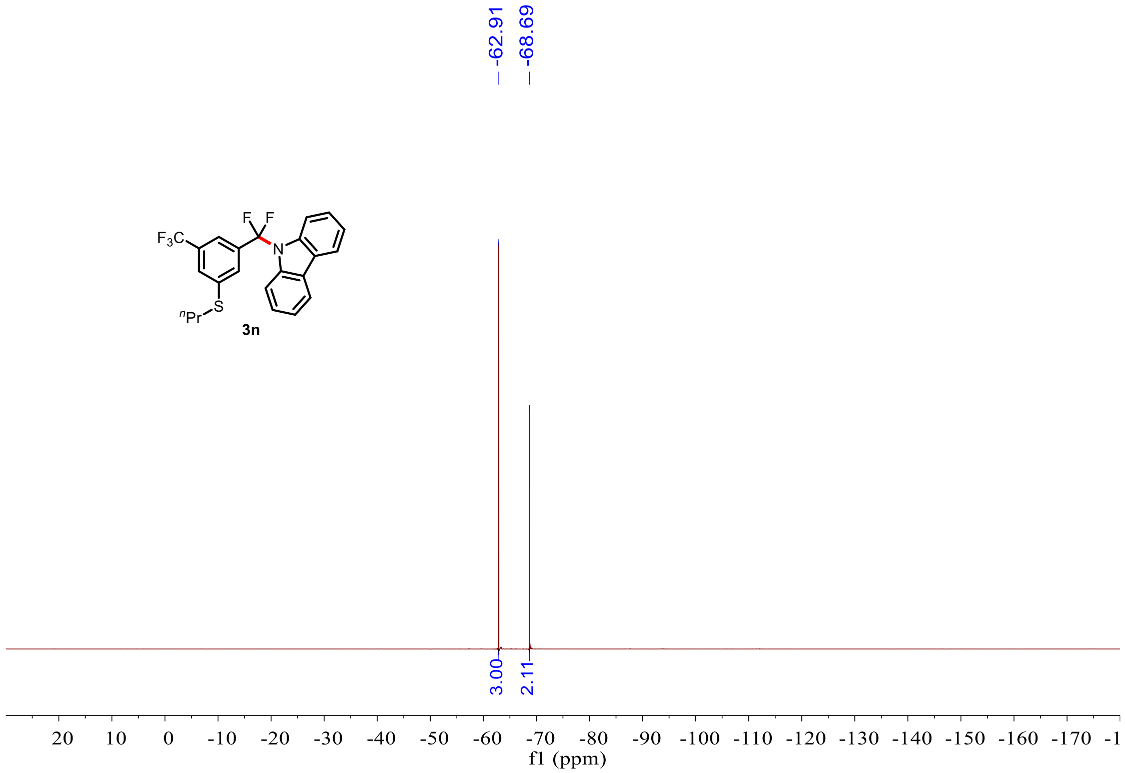

Supplementary Figure 81. **^{19}F NMR** (471 MHz, $CDCl_3$) spectrum for compound **3n**

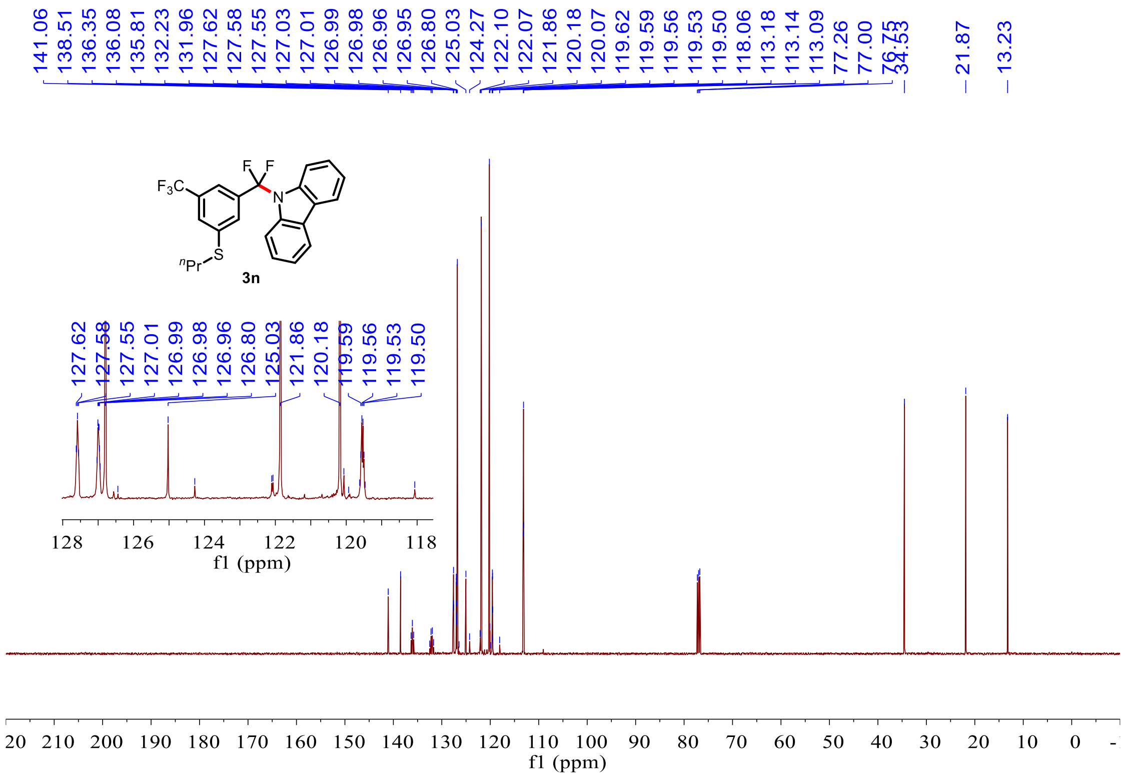

Supplementary Figure 82. ^{13}C NMR (126 MHz, $CDCl_3$) spectrum for compound **3n**

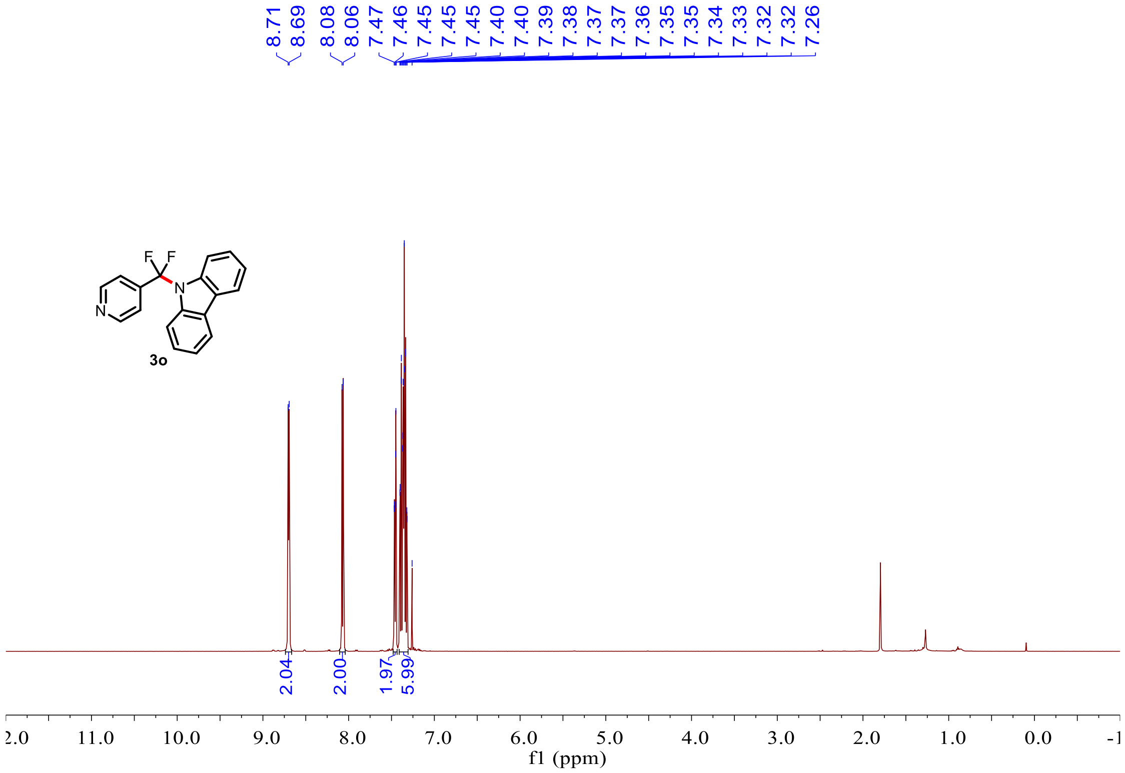

Supplementary Figure 83. ^{1}H NMR (500 MHz, $CDCl_3$) spectrum for compound **3o**

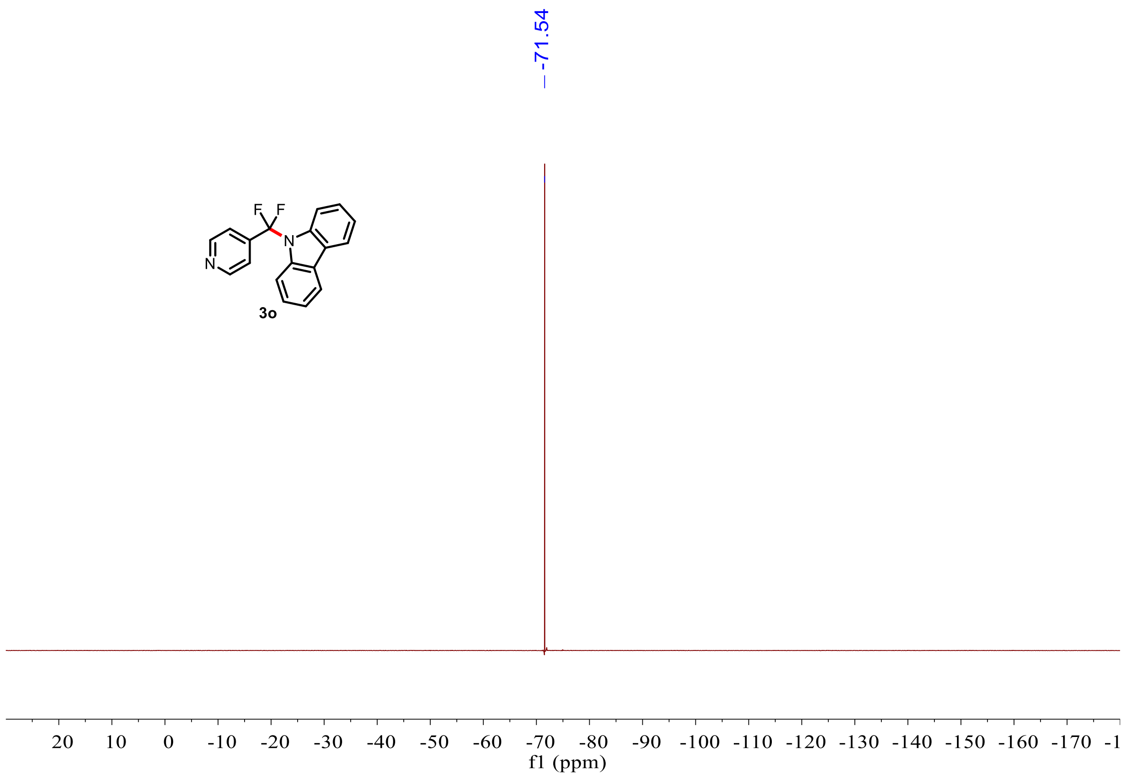

Supplementary Figure 84. ^{19}**F NMR** (471 MHz, $CDCl_3$) spectrum for compound **3o**

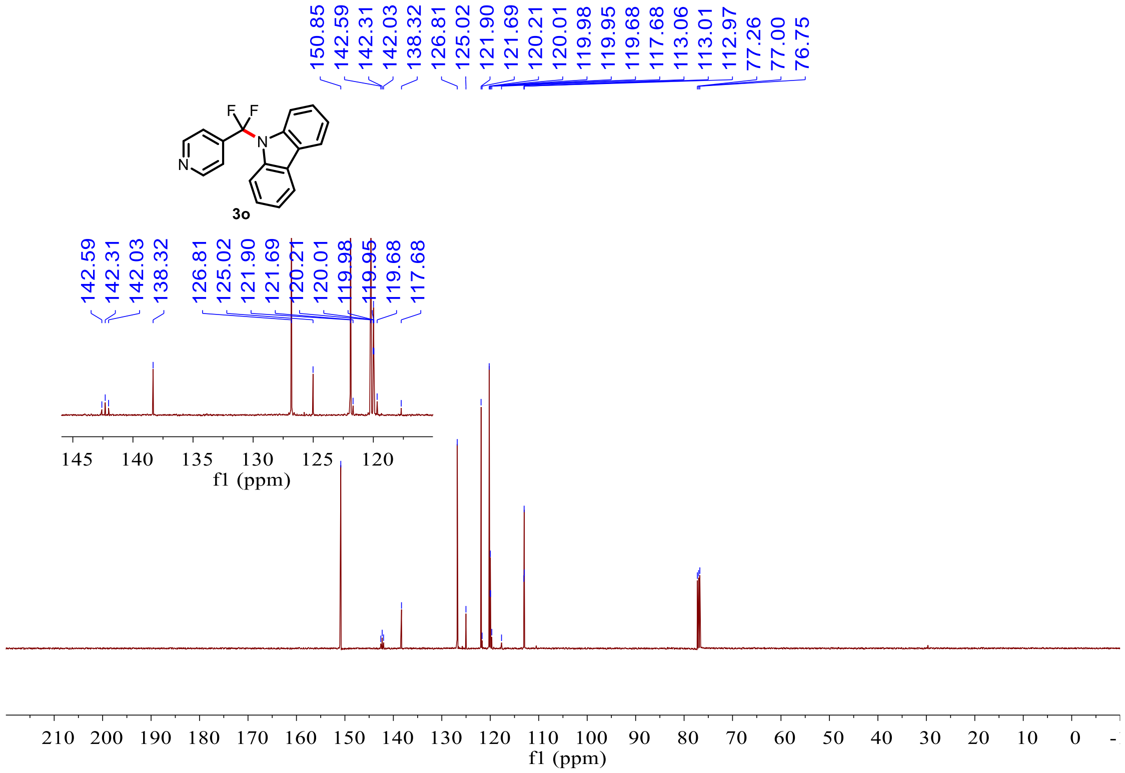

Supplementary Figure 85. ^{13}**C NMR** (126 MHz, $CDCl_3$) spectrum for compound **3o**

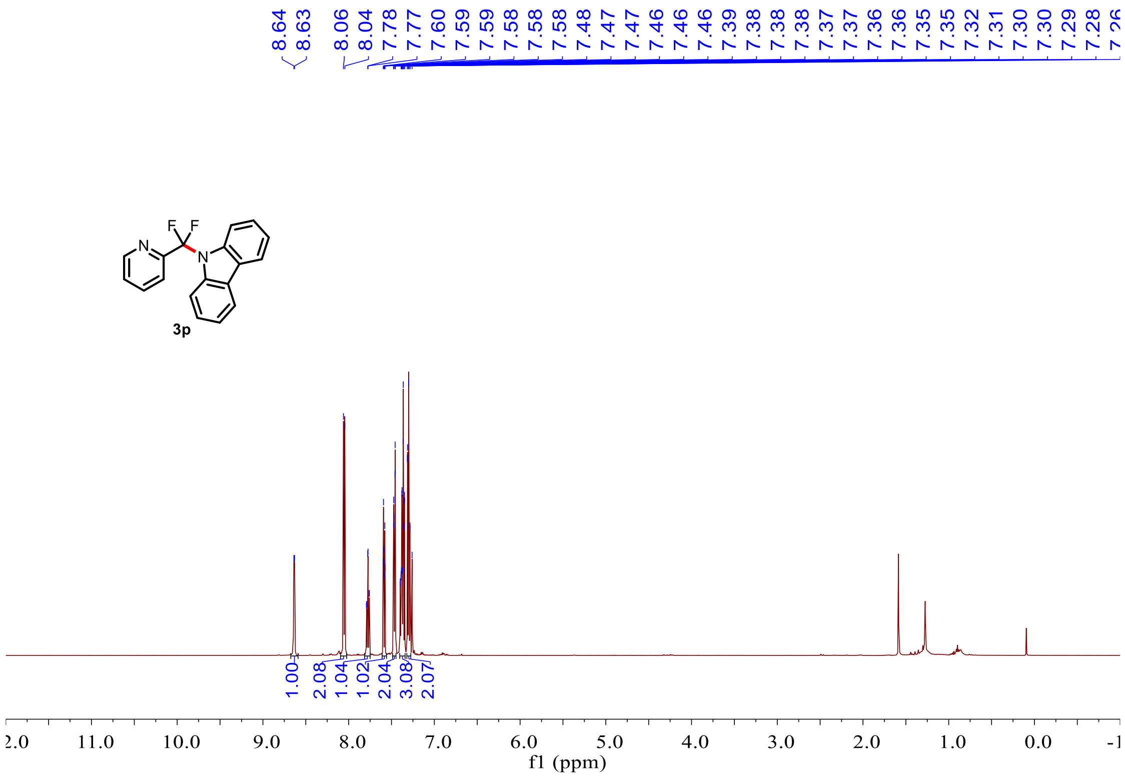

Supplementary Figure 86. ^{1}**H NMR** (500 MHz, $CDCl_3$) spectrum for compound **3p**

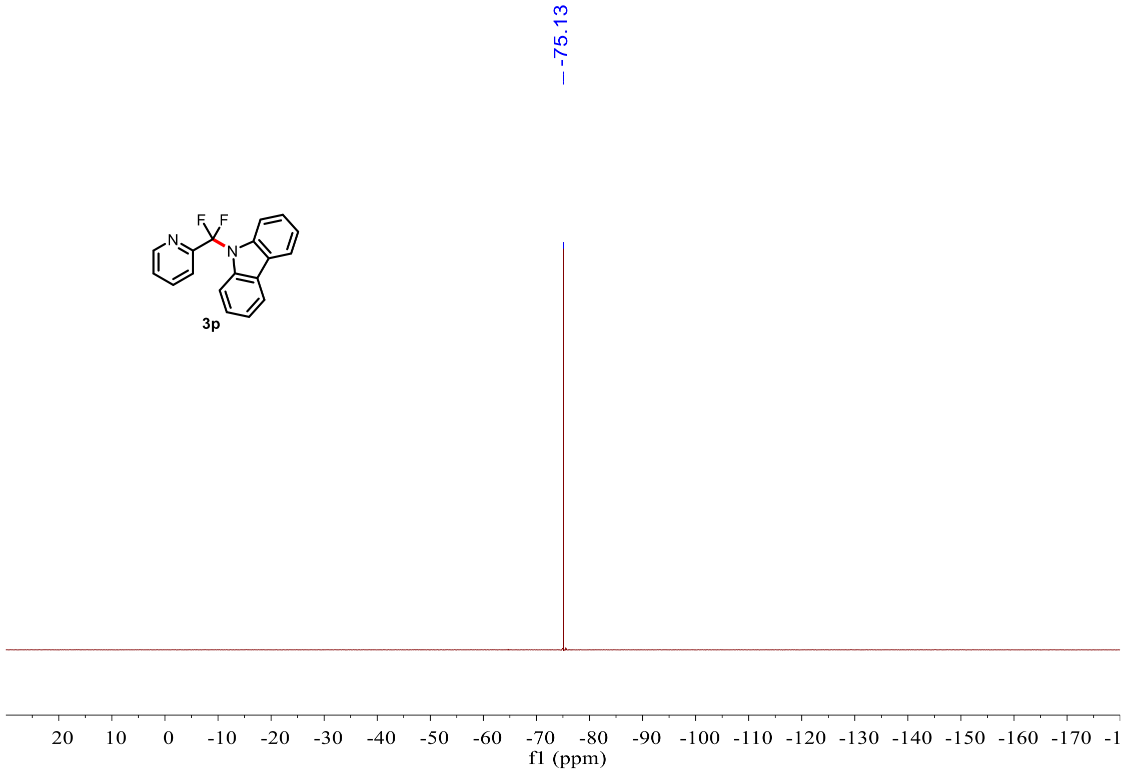

Supplementary Figure 87. ^{19}**F NMR** (471 MHz, $CDCl_3$) spectrum for compound **3p**

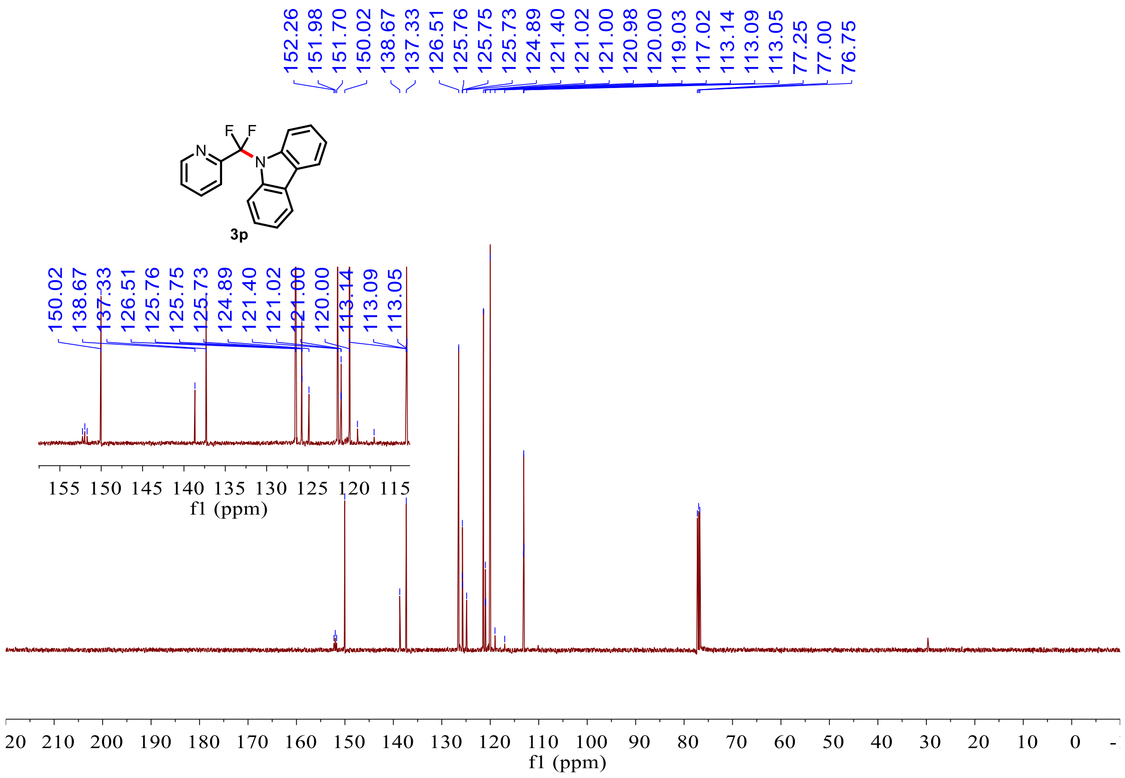

Supplementary Figure 88. ^{13}**C NMR** (126 MHz, $CDCl_3$) spectrum for compound **3p**

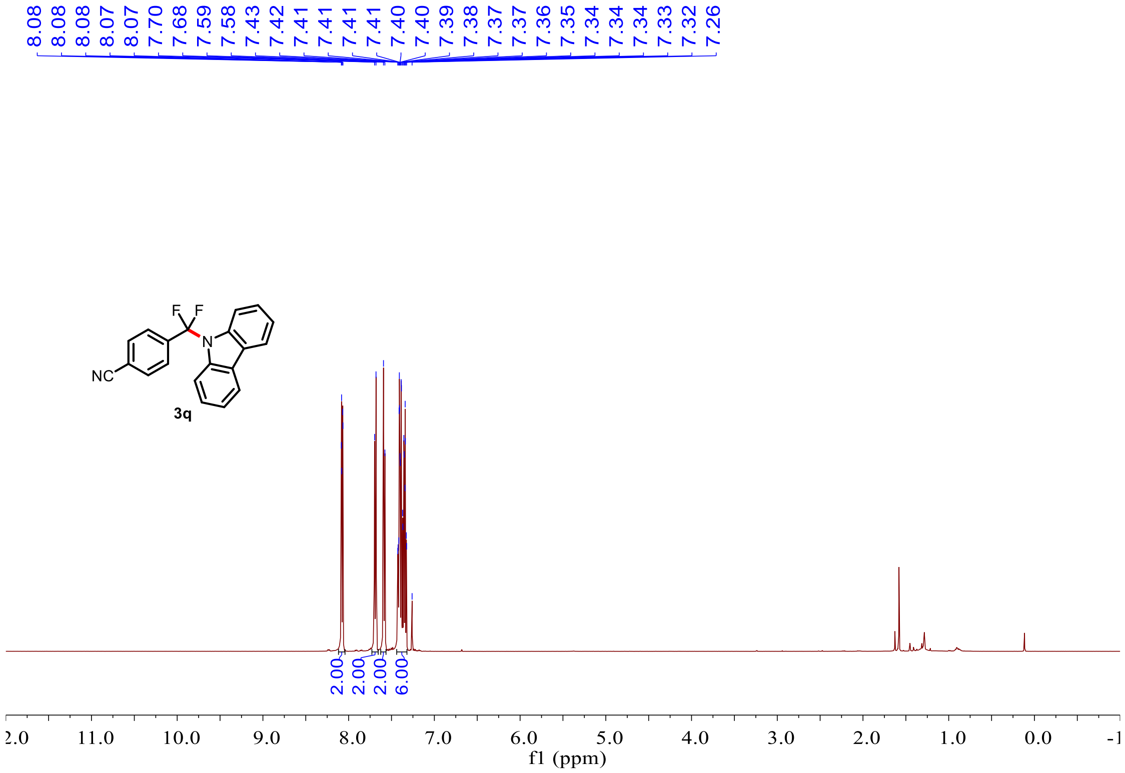

Supplementary Figure 89. ^{1}**H NMR** (500 MHz, $CDCl_3$) spectrum for compound **3q**

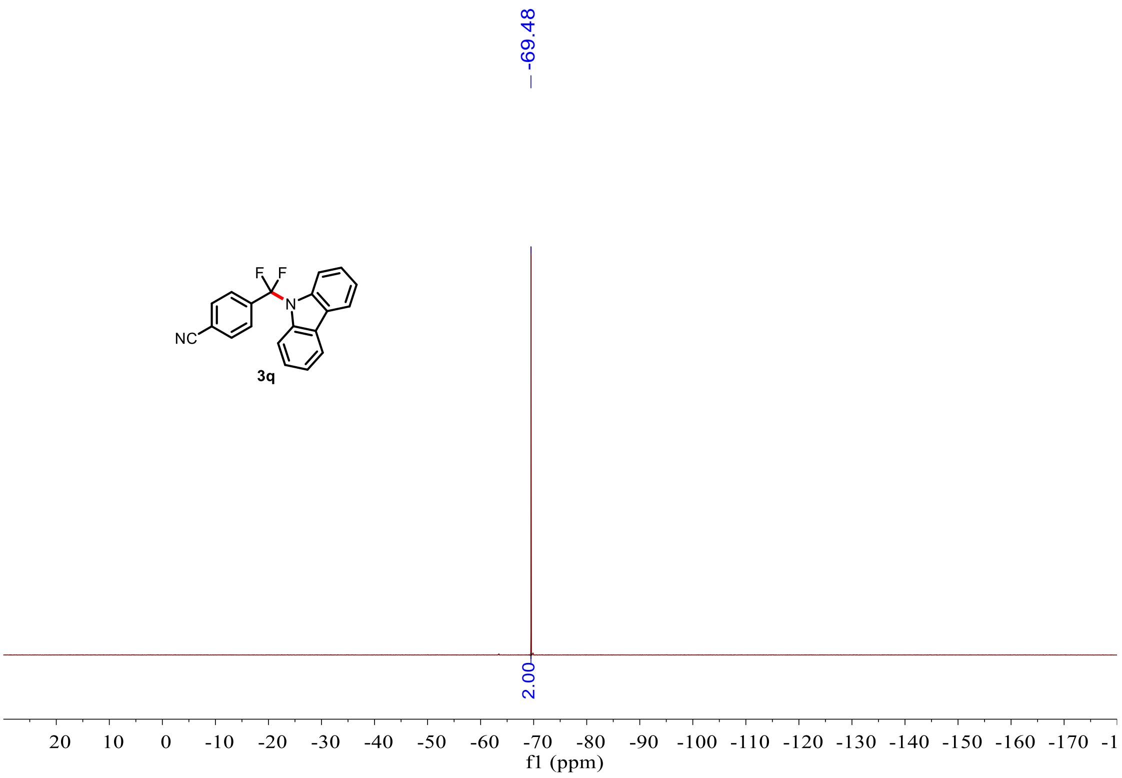

Supplementary Figure 90. **^{19}F NMR** (471 MHz, $CDCl_3$) spectrum for compound **3q**

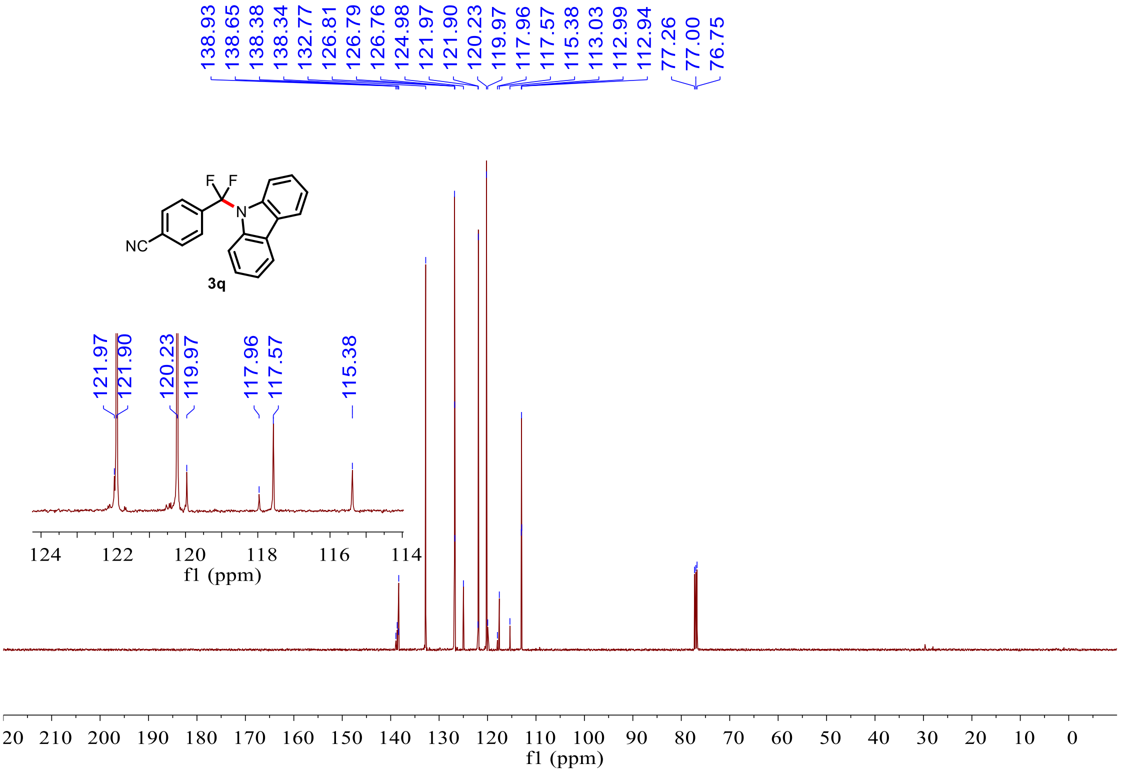

Supplementary Figure 91. **^{13}C NMR** (126 MHz, $CDCl_3$) spectrum for compound **3q**

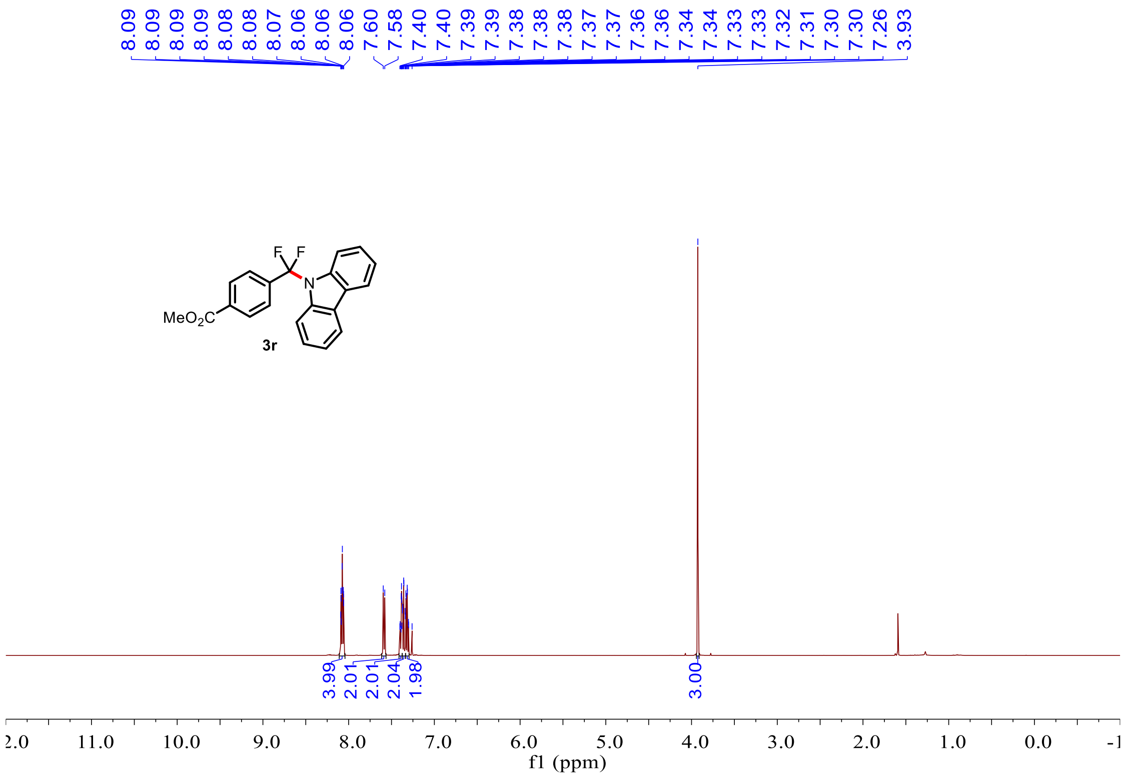

Supplementary Figure 92. ^{1}H NMR (500 MHz, $CDCl_3$) spectrum for compound **3r**

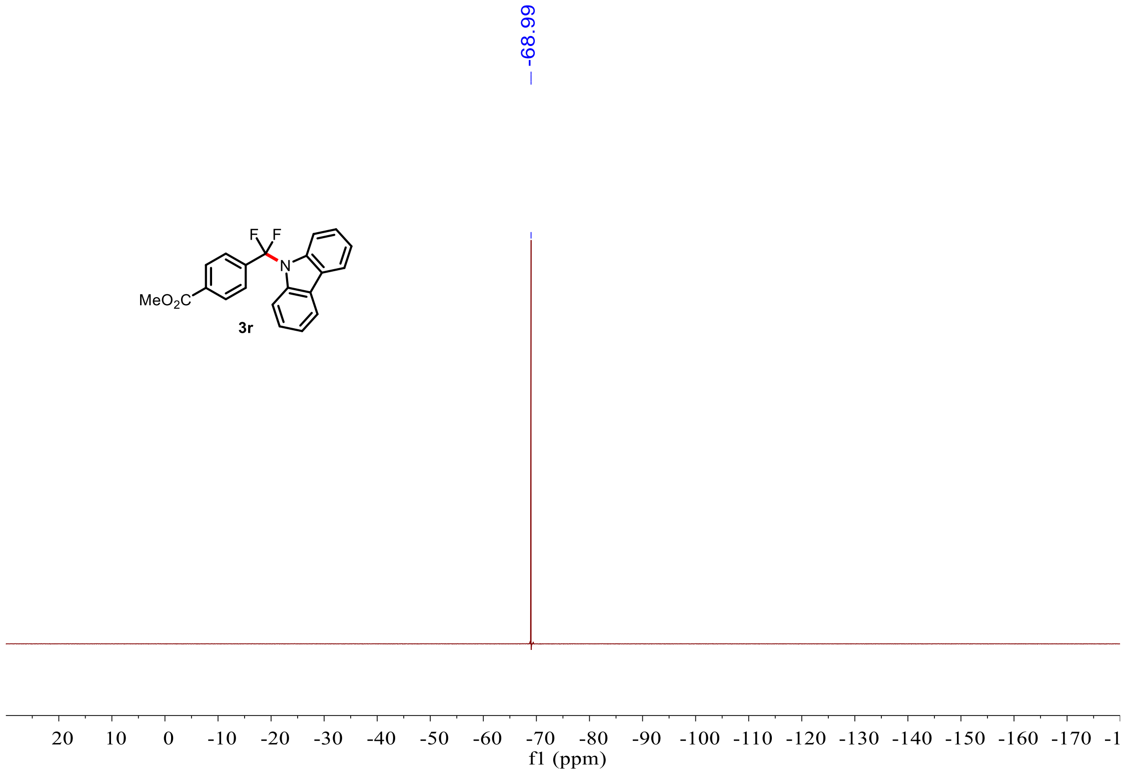

Supplementary Figure 93. ^{19}F NMR (471 MHz, $CDCl_3$) spectrum for compound **3r**

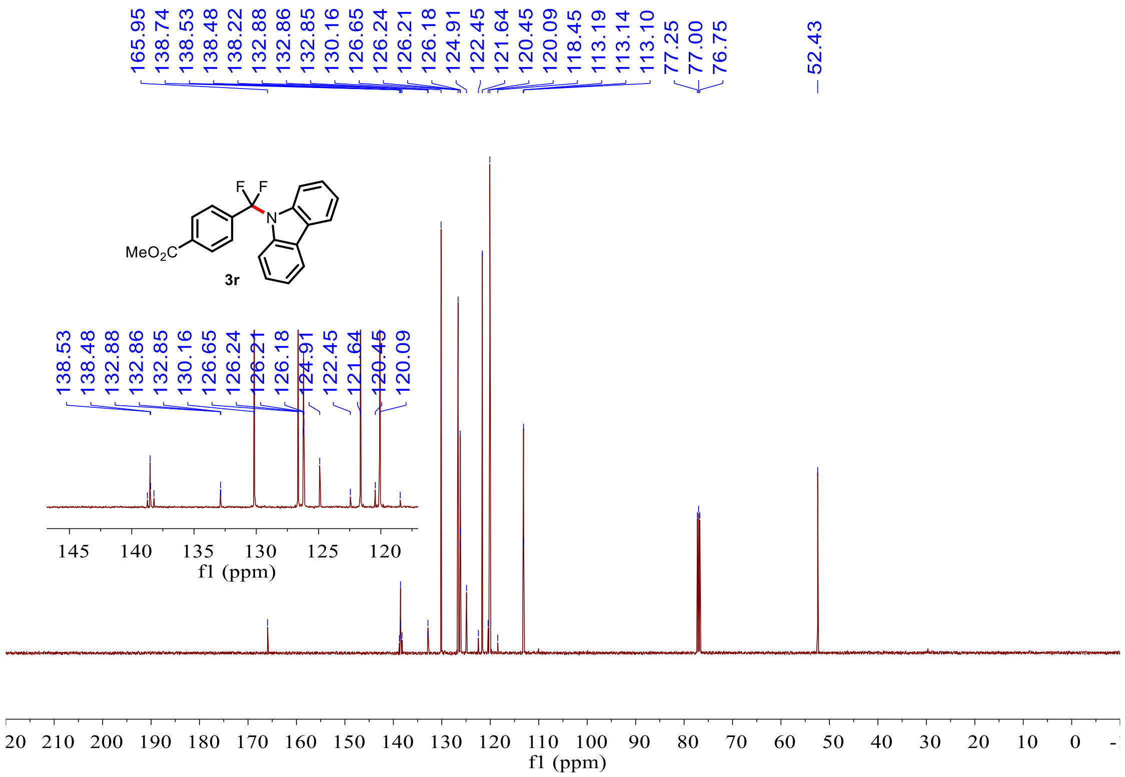

Supplementary Figure 94. ^{13}**C NMR** (126 MHz, $CDCl_3$) spectrum for compound **3r**

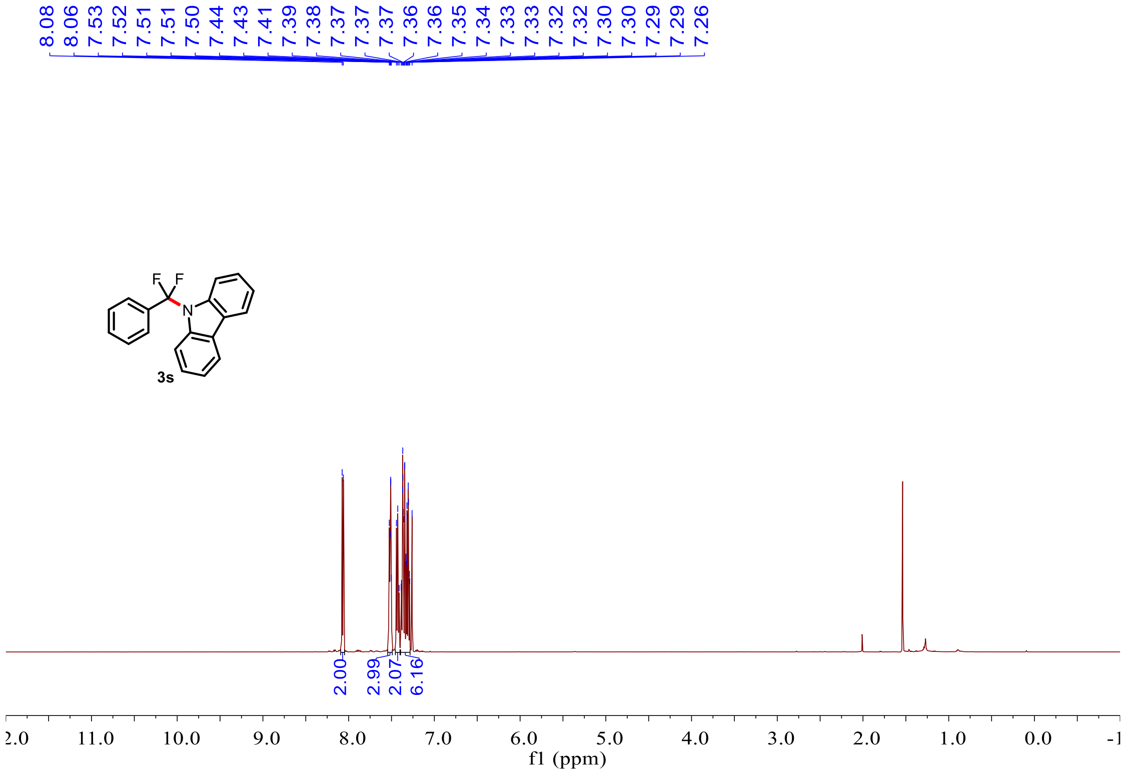

Supplementary Figure 95. ^{1}**H NMR** (500 MHz, $CDCl_3$) spectrum for compound **3s**

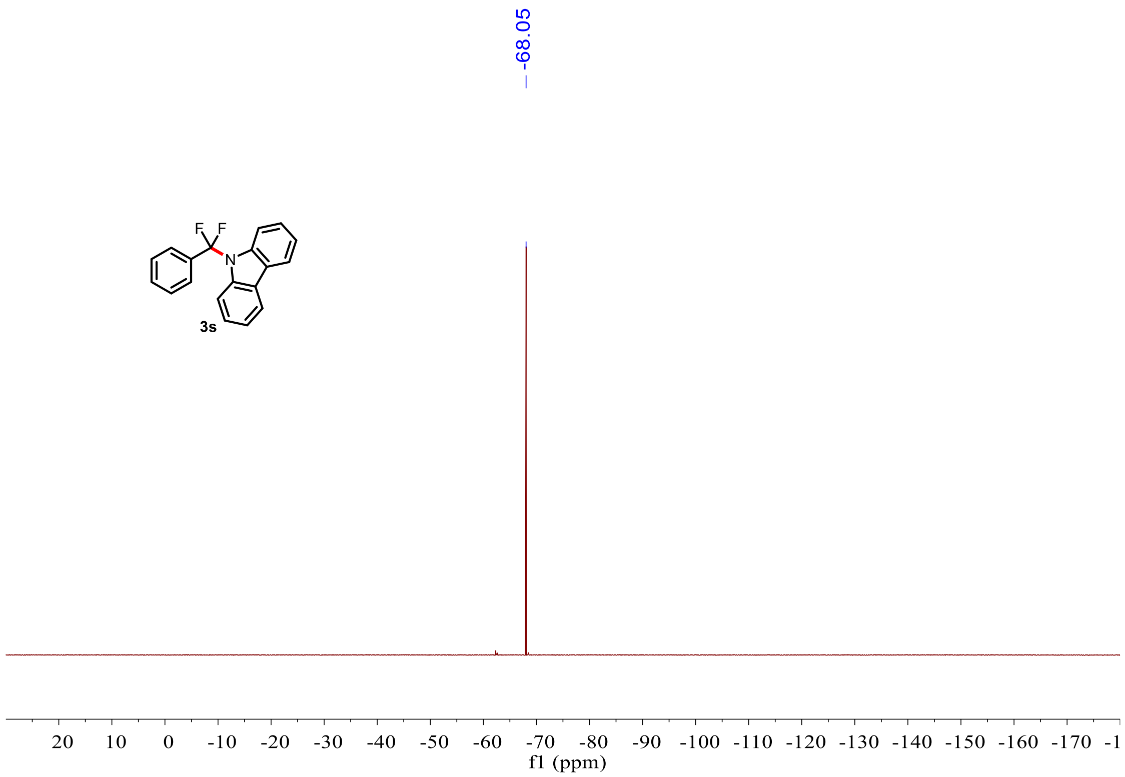

Supplementary Figure 96. ^{19}**F NMR** (471 MHz, $CDCl_3$) spectrum for compound **3s**

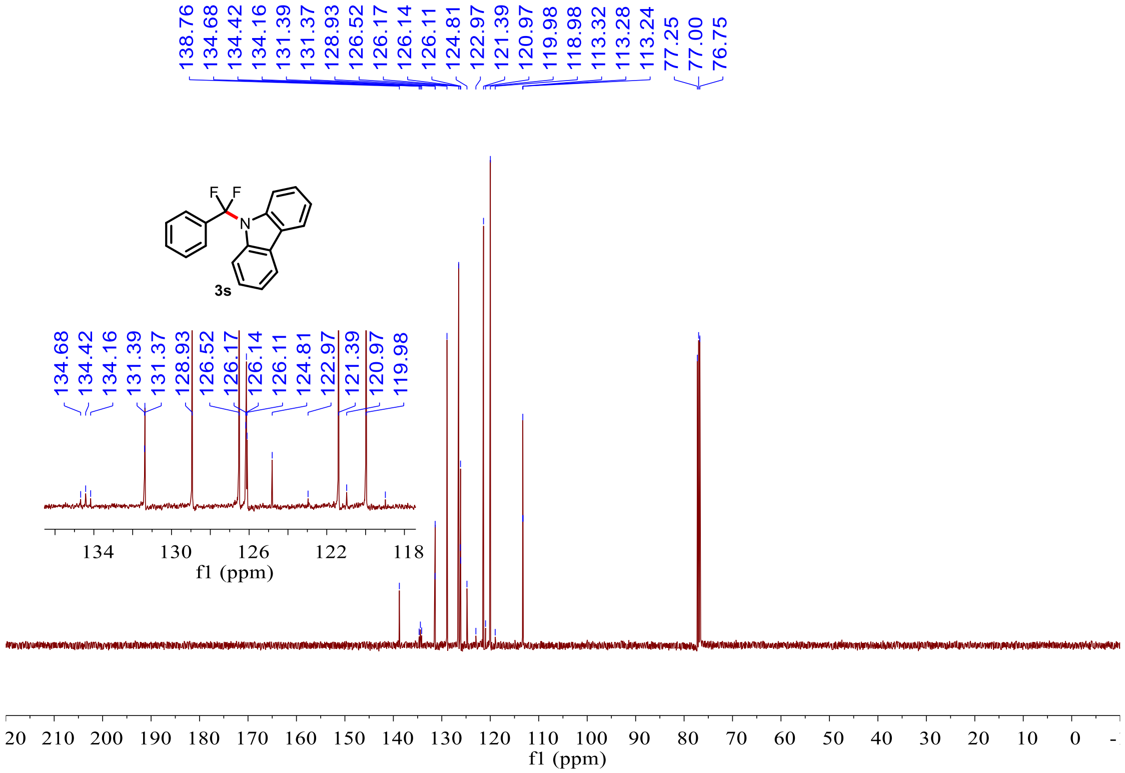

Supplementary Figure 97. ^{13}**C NMR** (126 MHz, $CDCl_3$) spectrum for compound **3s**

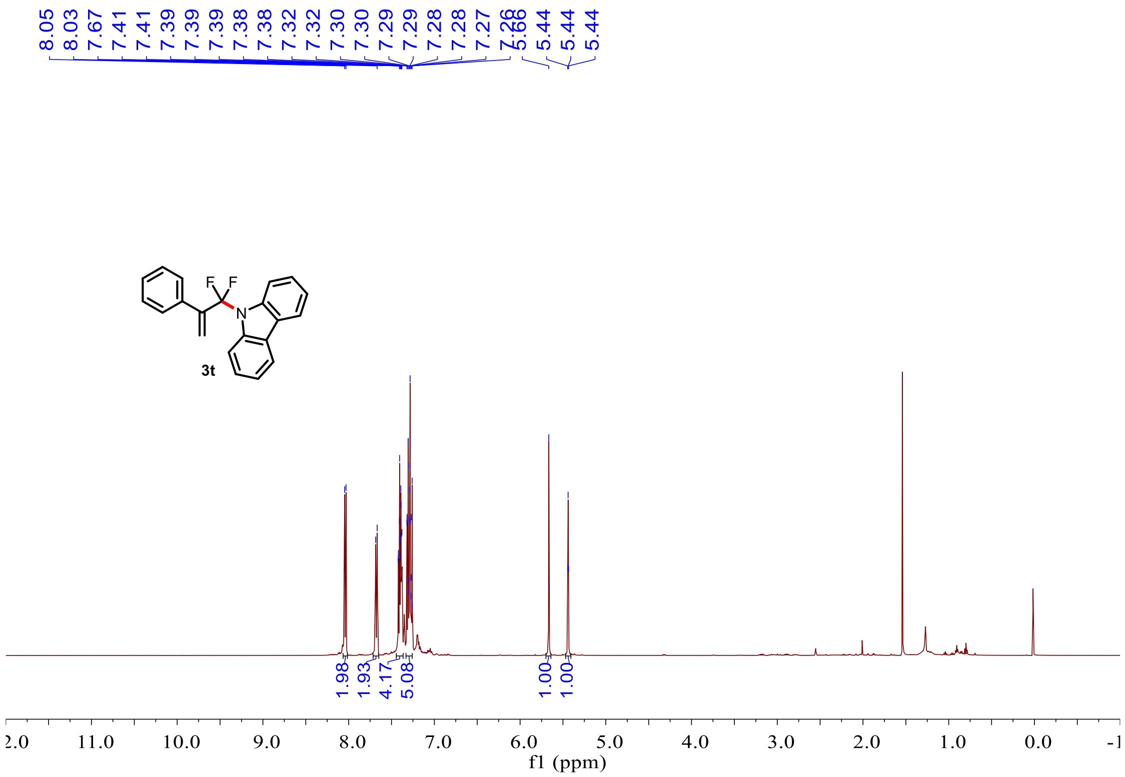

Supplementary Figure 98. **^{1}H NMR** (500 MHz, $CDCl_3$) spectrum for compound **3t**

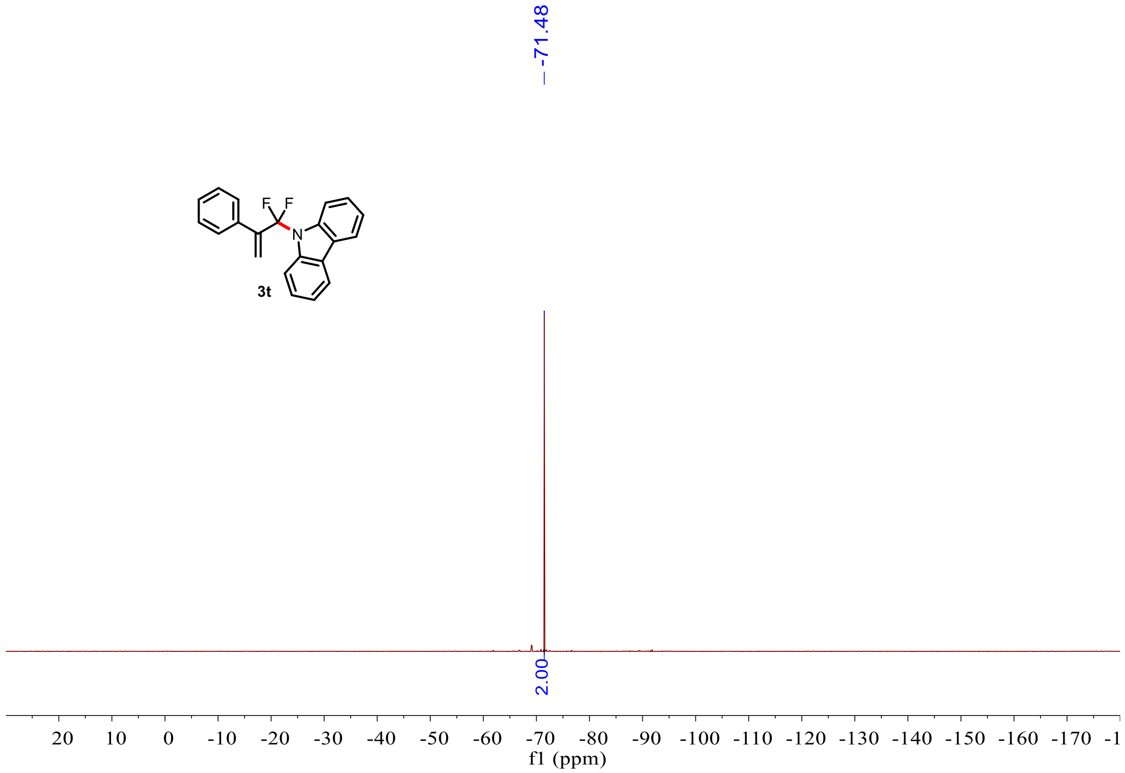

Supplementary Figure 99. **^{19}F NMR** (471 MHz, $CDCl_3$) spectrum for compound **3t**

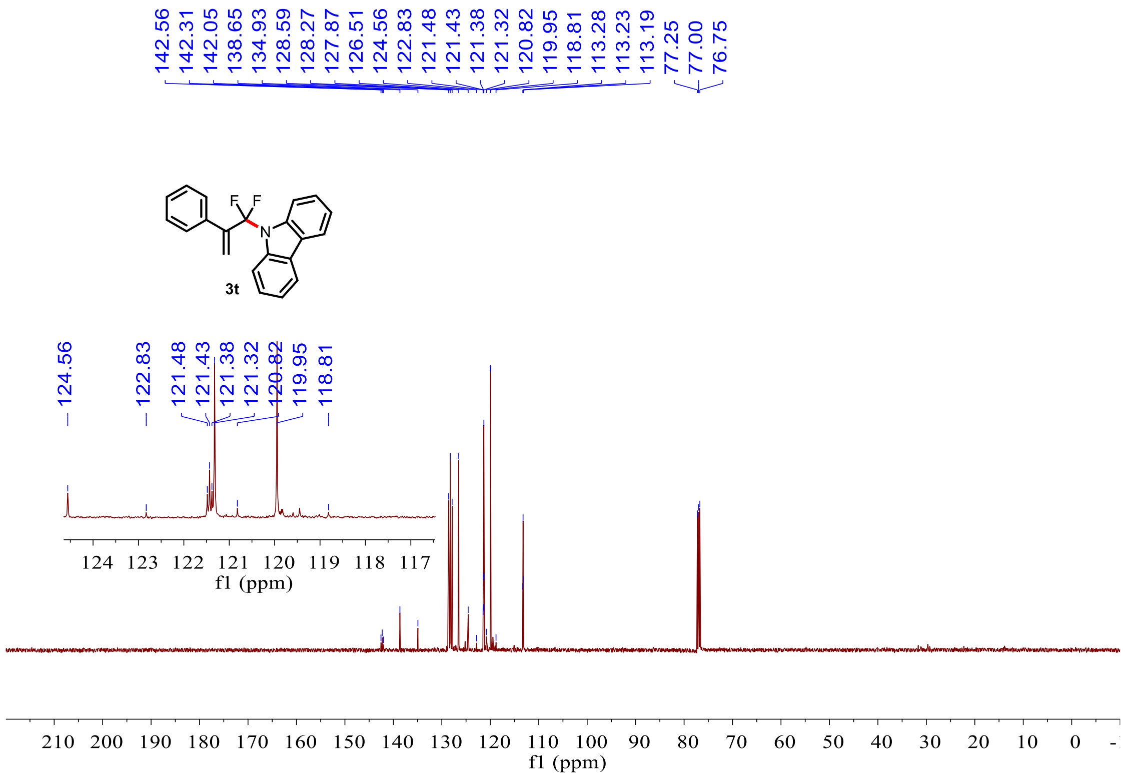

Supplementary Figure 100. ^{13}**C NMR** (126 MHz, $CDCl_3$) spectrum for compound **3t**

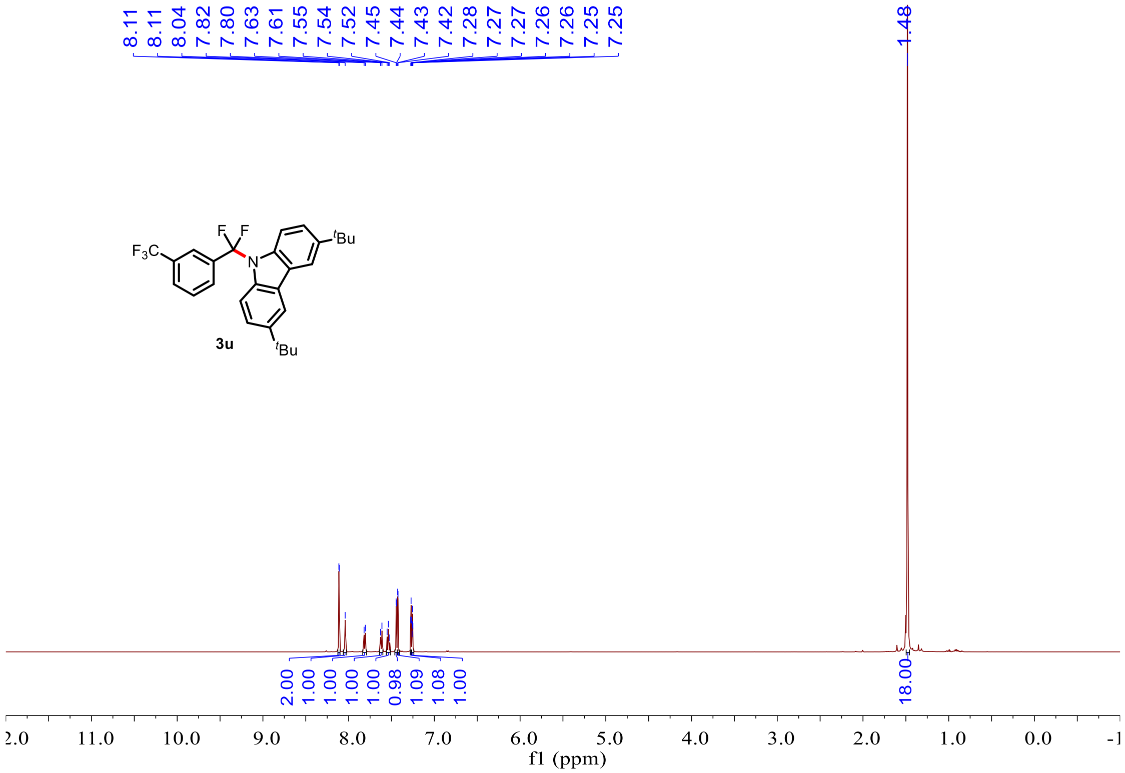

Supplementary Figure 101. ^{1}**H NMR** (500 MHz, $CDCl_3$) spectrum for compound **3u**

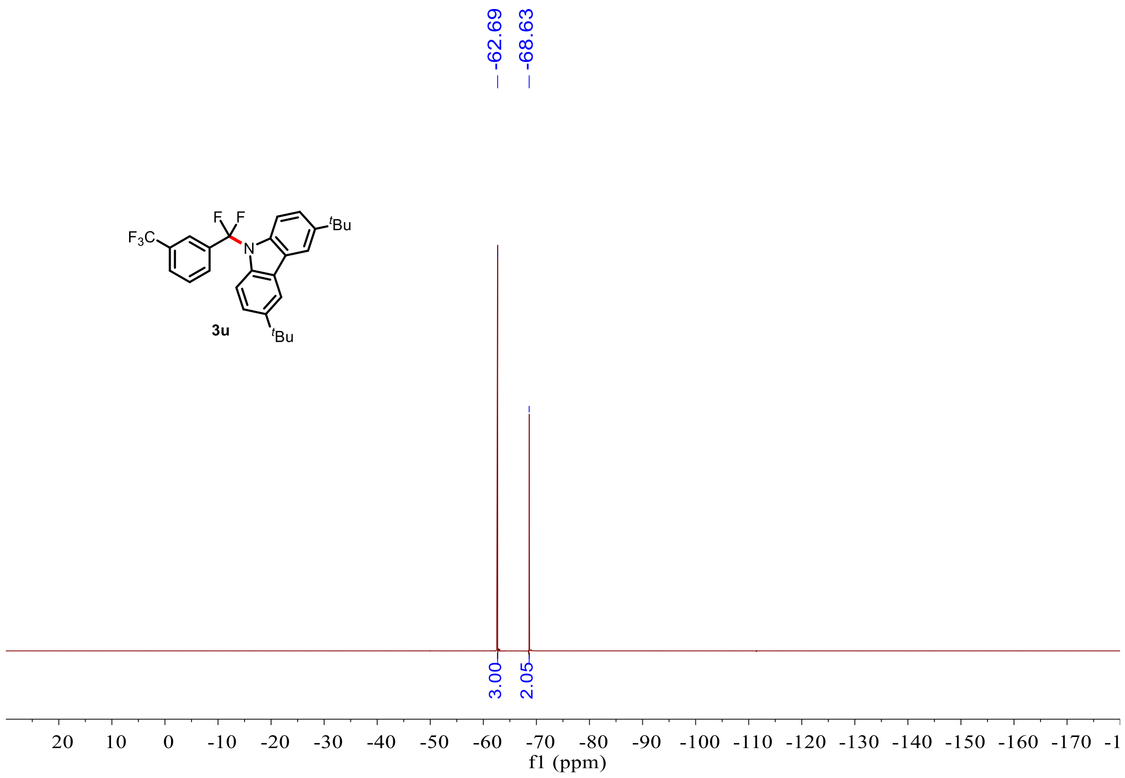

Supplementary Figure 102. ^{19}**F NMR** (471 MHz, $CDCl_3$) spectrum for compound **3u**

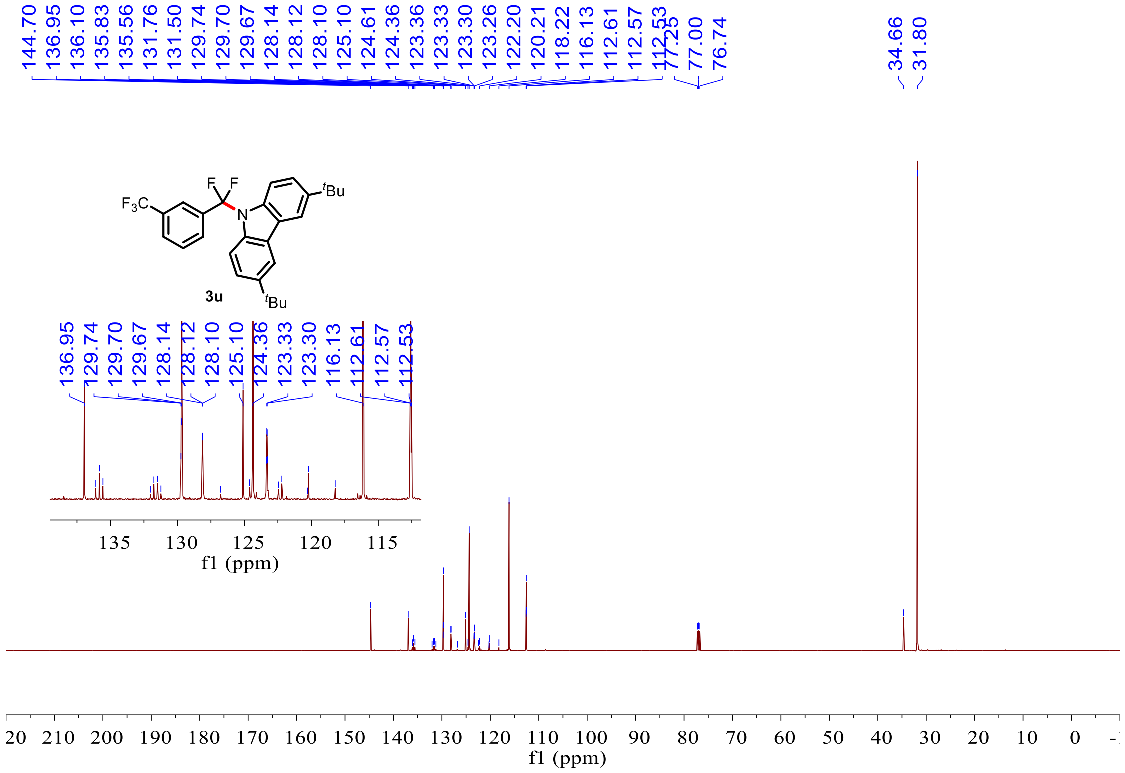

Supplementary Figure 103. ^{13}**C NMR** (126 MHz, $CDCl_3$) spectrum for compound **3u**

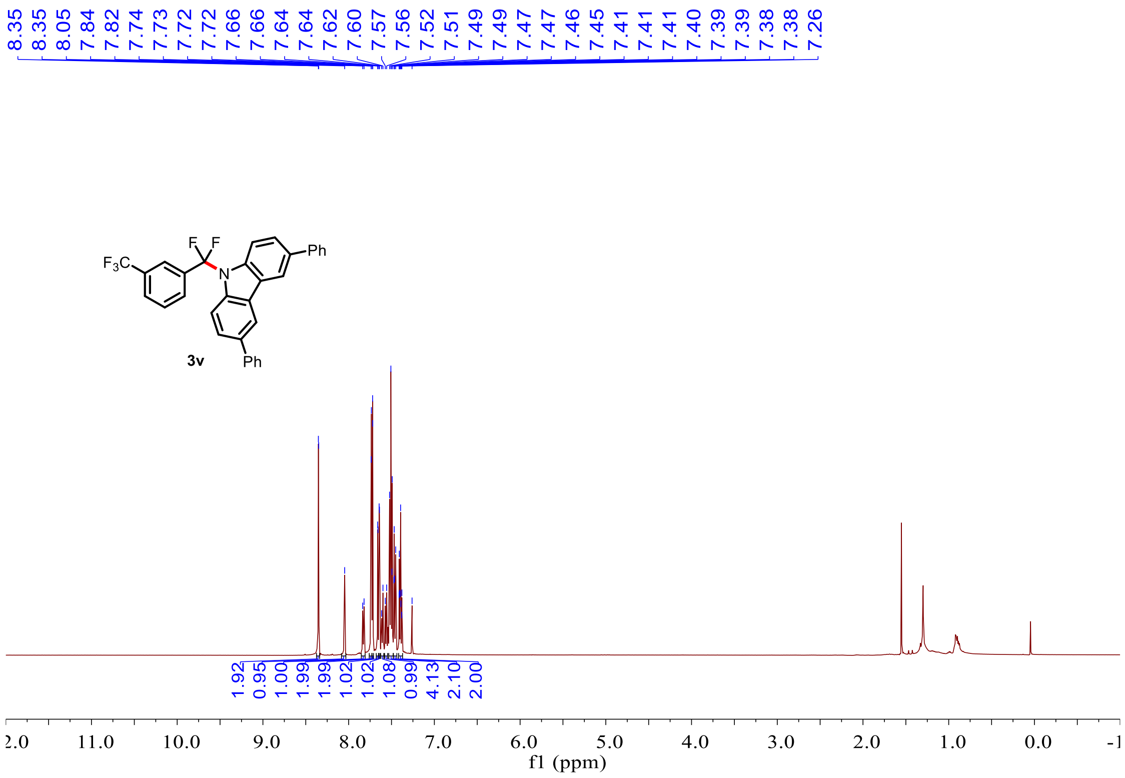

Supplementary Figure 104. ^{1}**H NMR** (500 MHz, $CDCl_3$) spectrum for compound **3v**

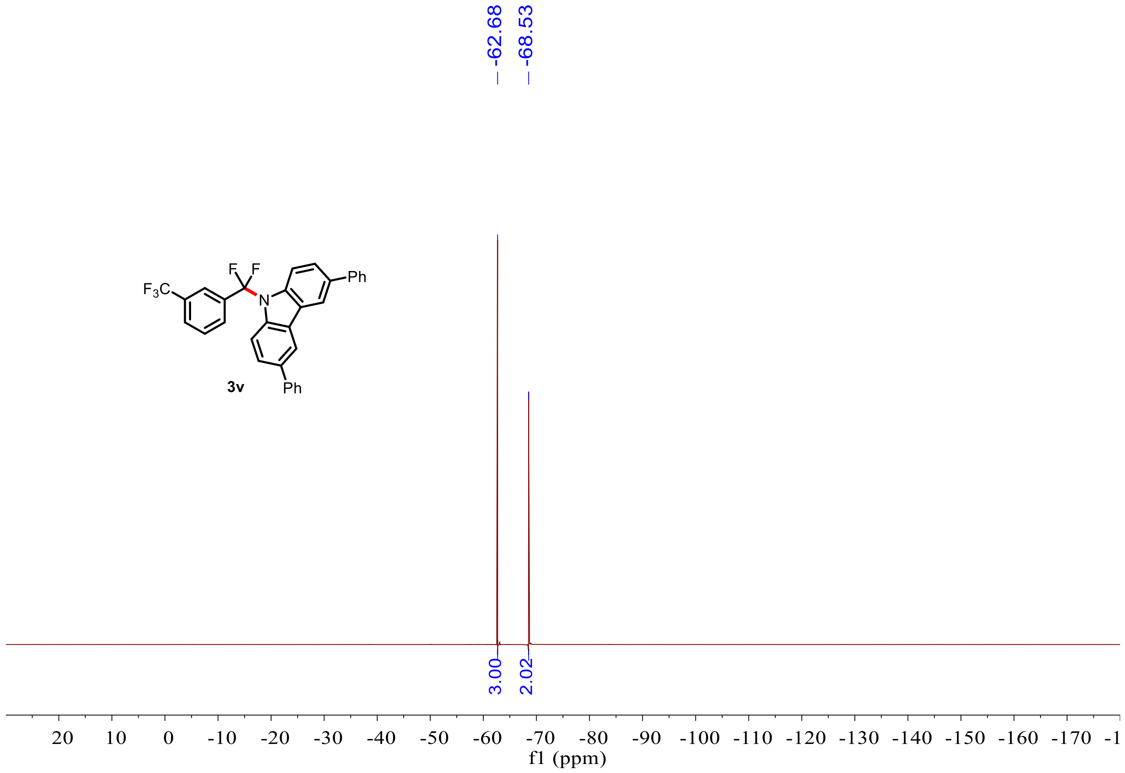

Supplementary Figure 105. ^{19}**F NMR** (471 MHz, $CDCl_3$) spectrum for compound **3v**

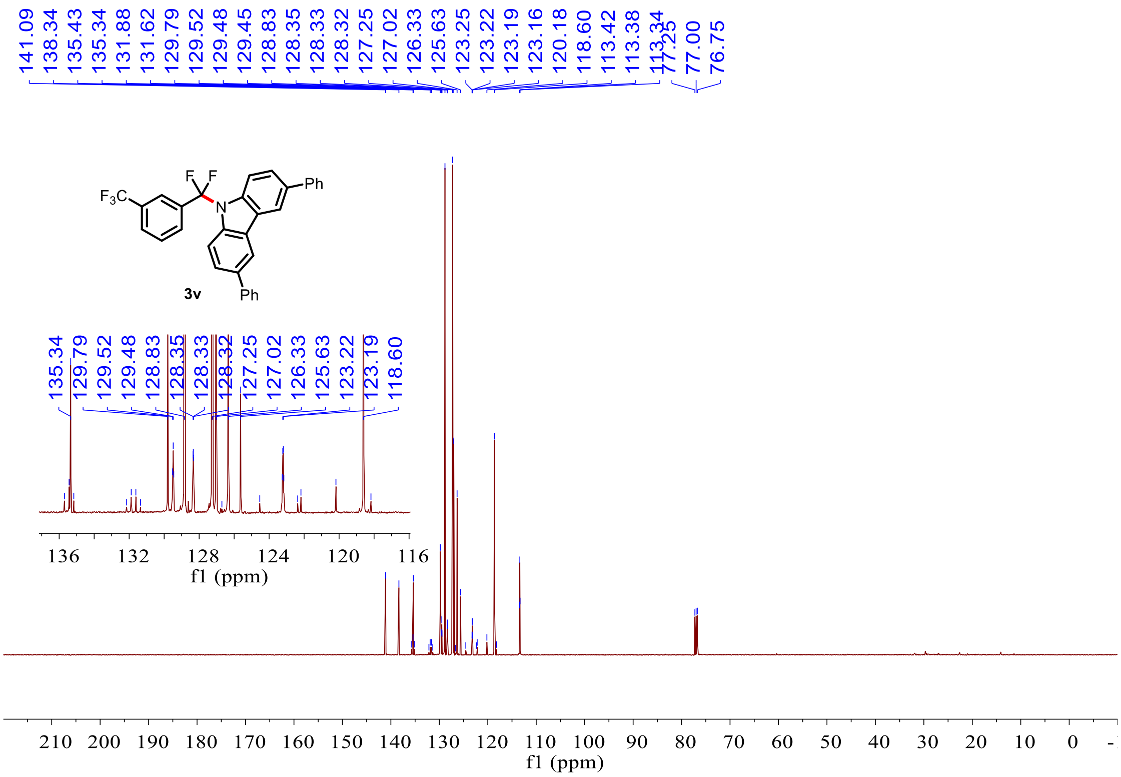

Supplementary Figure 106. ^{13}C NMR (126 MHz, $CDCl_3$) spectrum for compound **3v**

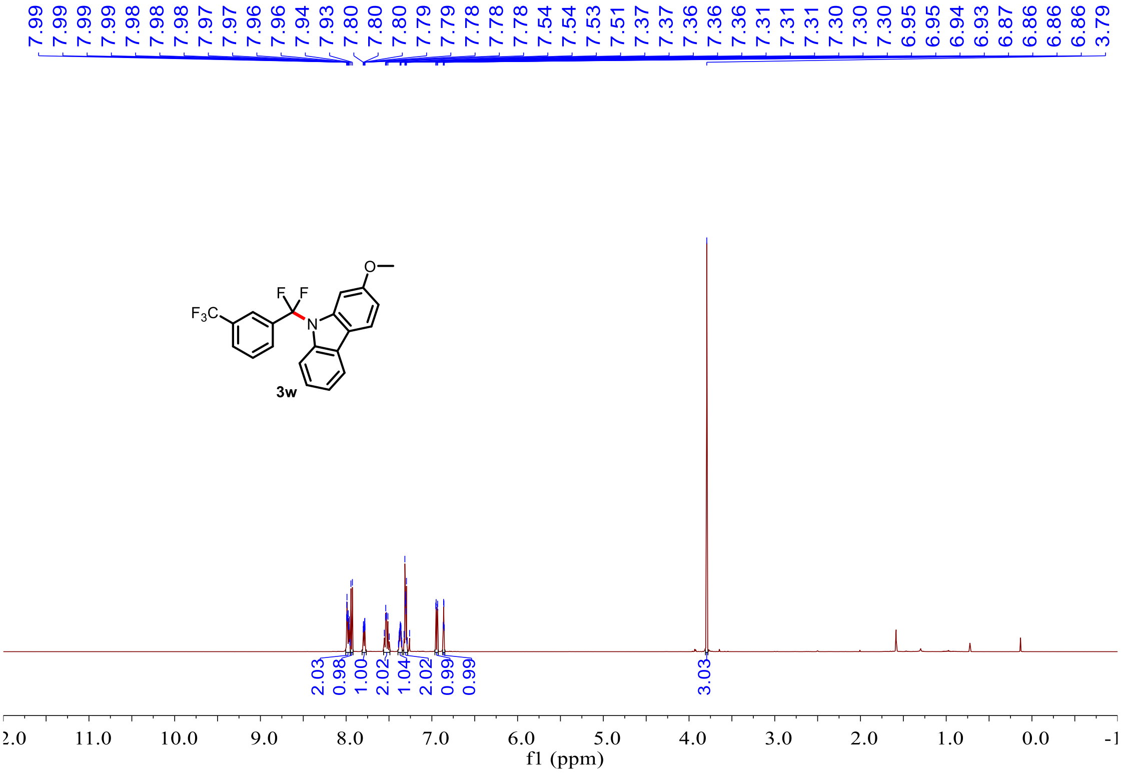

Supplementary Figure 107. ^{1}H NMR (500 MHz, $CDCl_3$) spectrum for compound **3w**

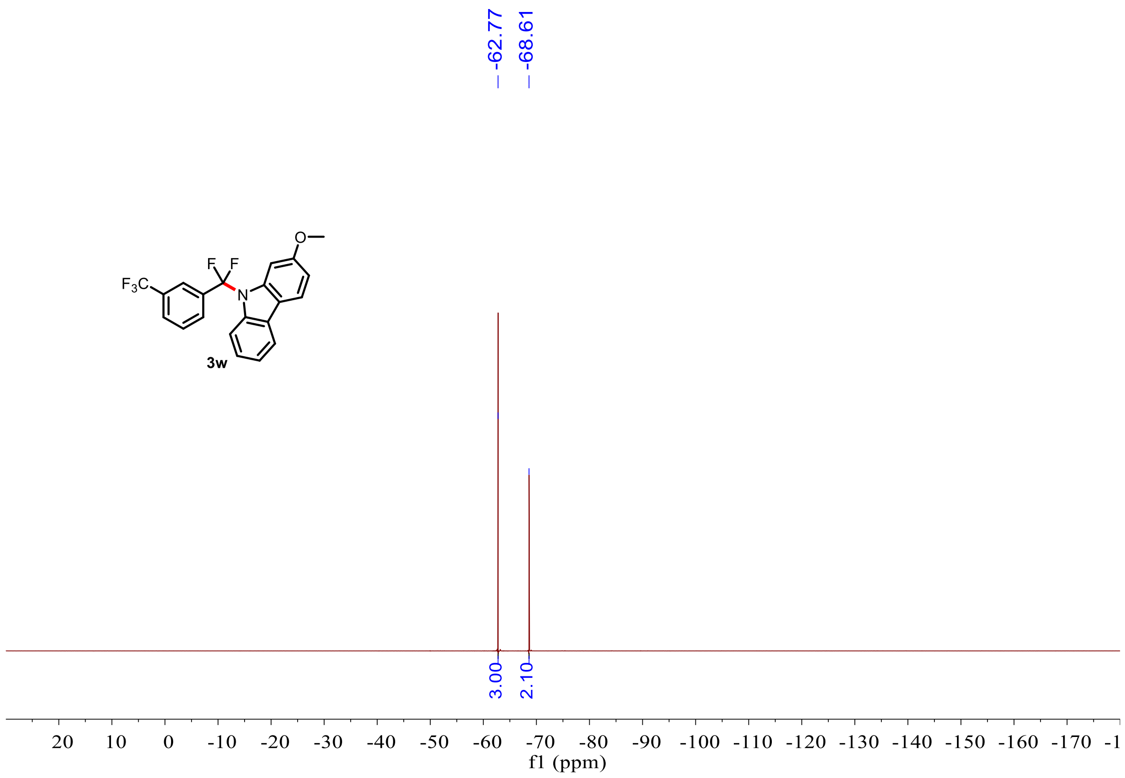

Supplementary Figure 108. ^{19}F NMR (471 MHz, $CDCl_3$) spectrum for compound **3w**

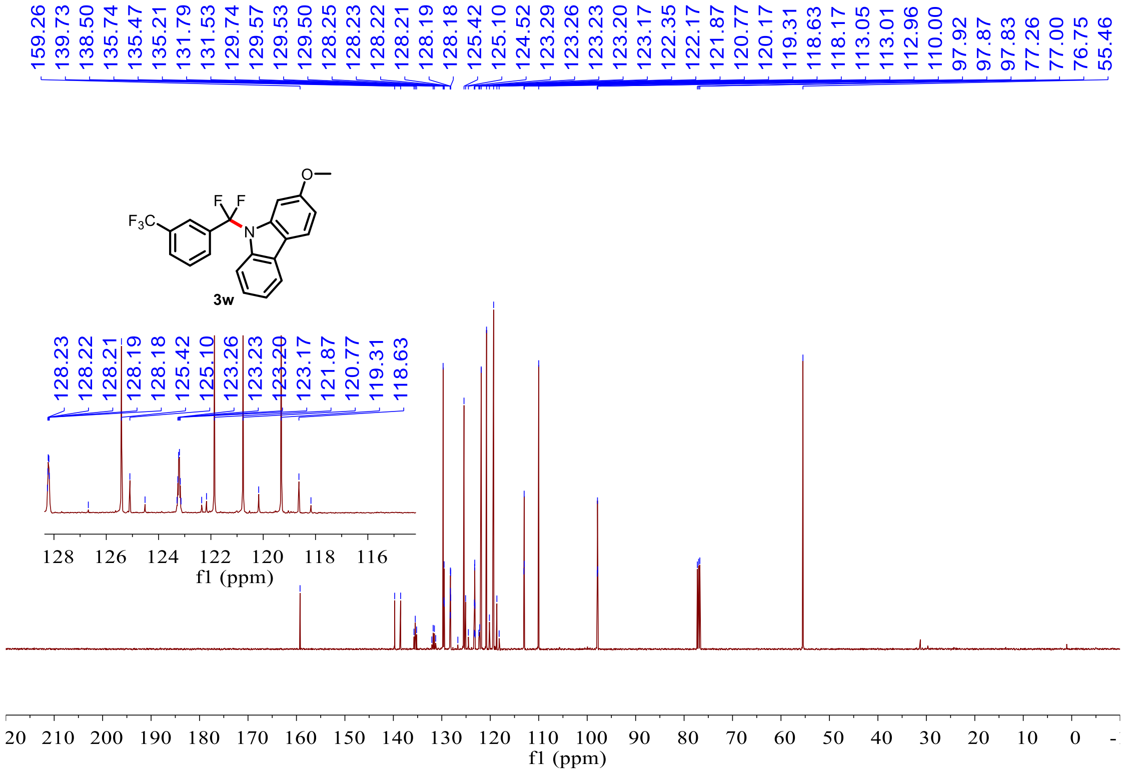

Supplementary Figure 109. ^{13}C NMR (126 MHz, $CDCl_3$) spectrum for compound **3w**

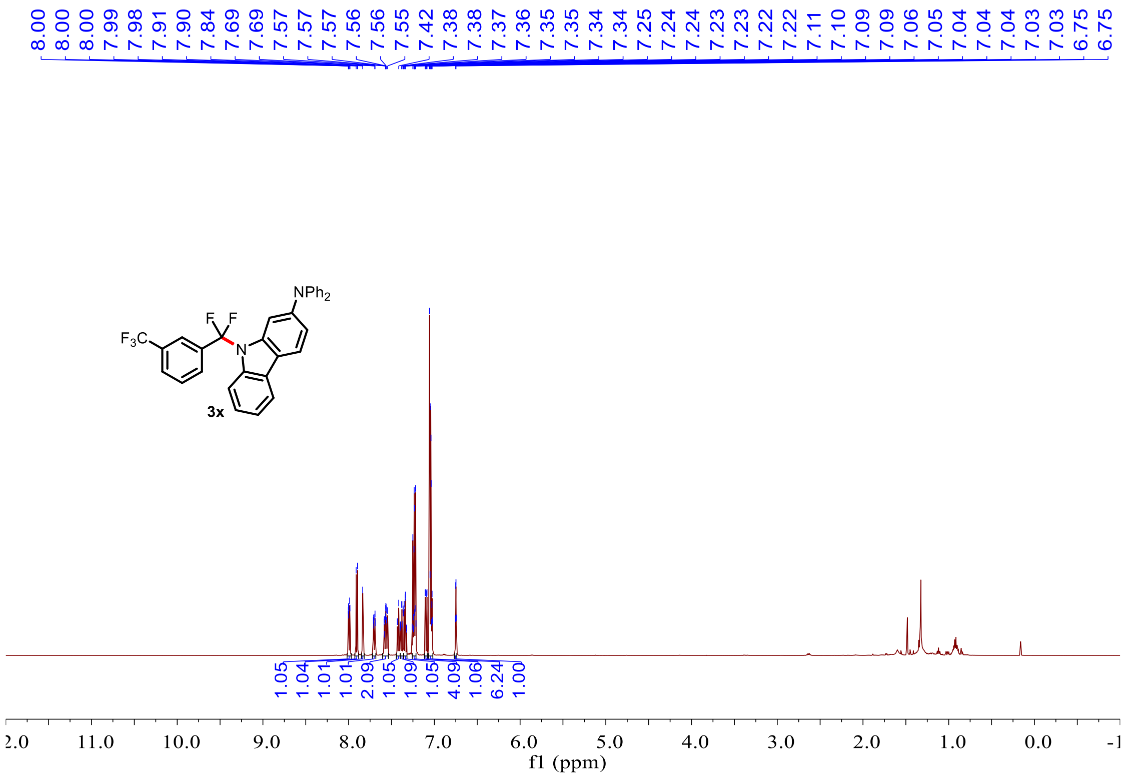

Supplementary Figure 110. ¹H NMR (500 MHz, $CDCl_3$) spectrum for compound **3x**

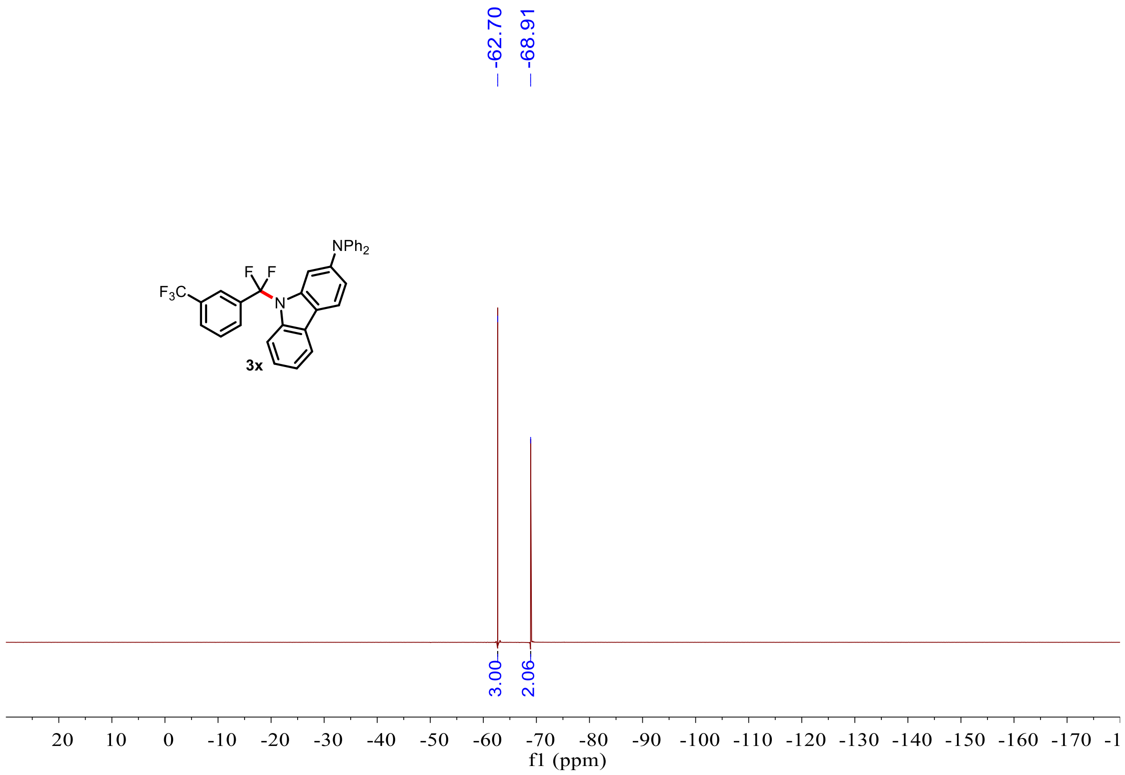

Supplementary Figure 111. ^{19}**F NMR** (471 MHz, $CDCl_3$) spectrum for compound **3x**

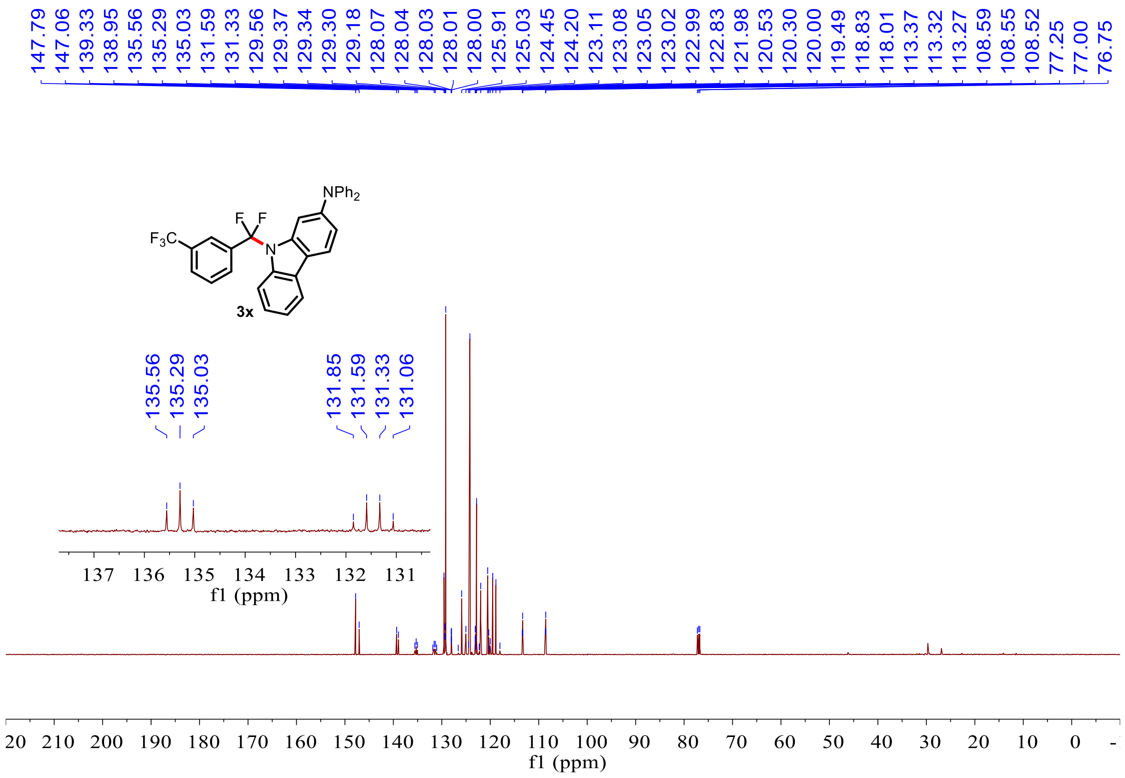

Supplementary Figure 112. ^{13}C NMR (126 MHz, $CDCl_3$) spectrum for compound **3x**

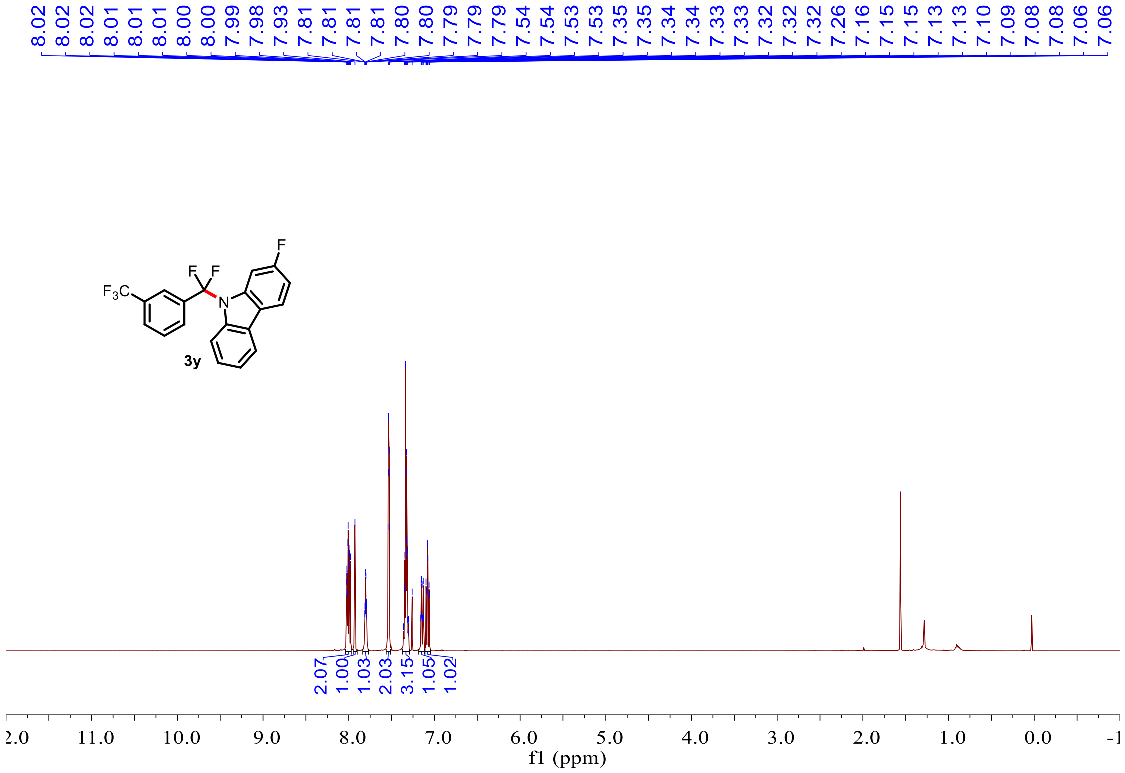

Supplementary Figure 113. ^{1}H NMR (500 MHz, $CDCl_3$) spectrum for compound **3y**

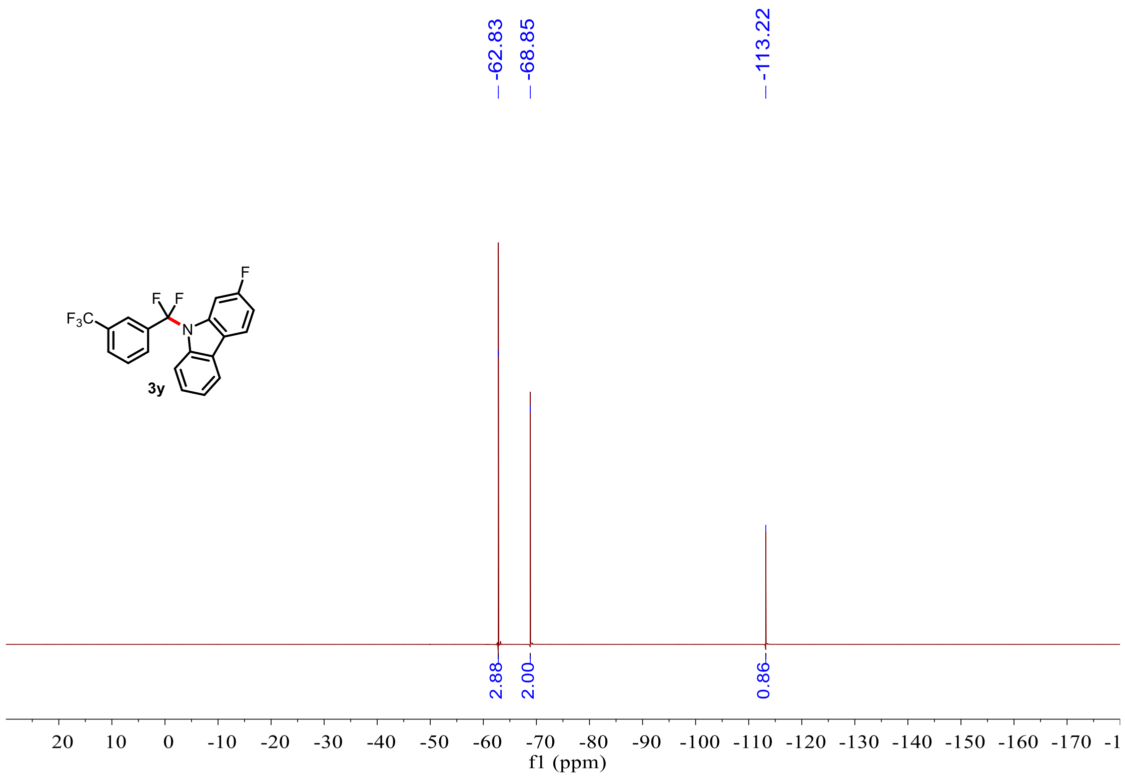

Supplementary Figure 114. ^{19}**F NMR** (471 MHz, $CDCl_3$) spectrum for compound **3y**

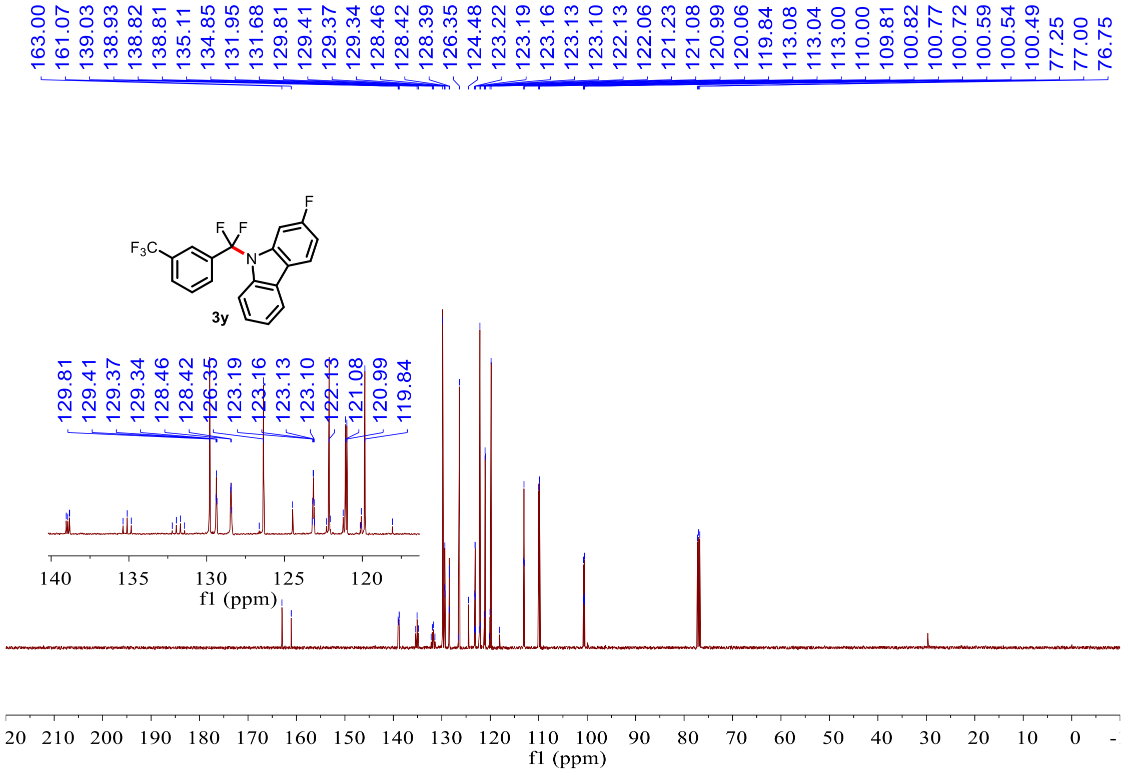

Supplementary Figure 115. ^{13}**C NMR** (126 MHz, $CDCl_3$) spectrum for compound **3y**

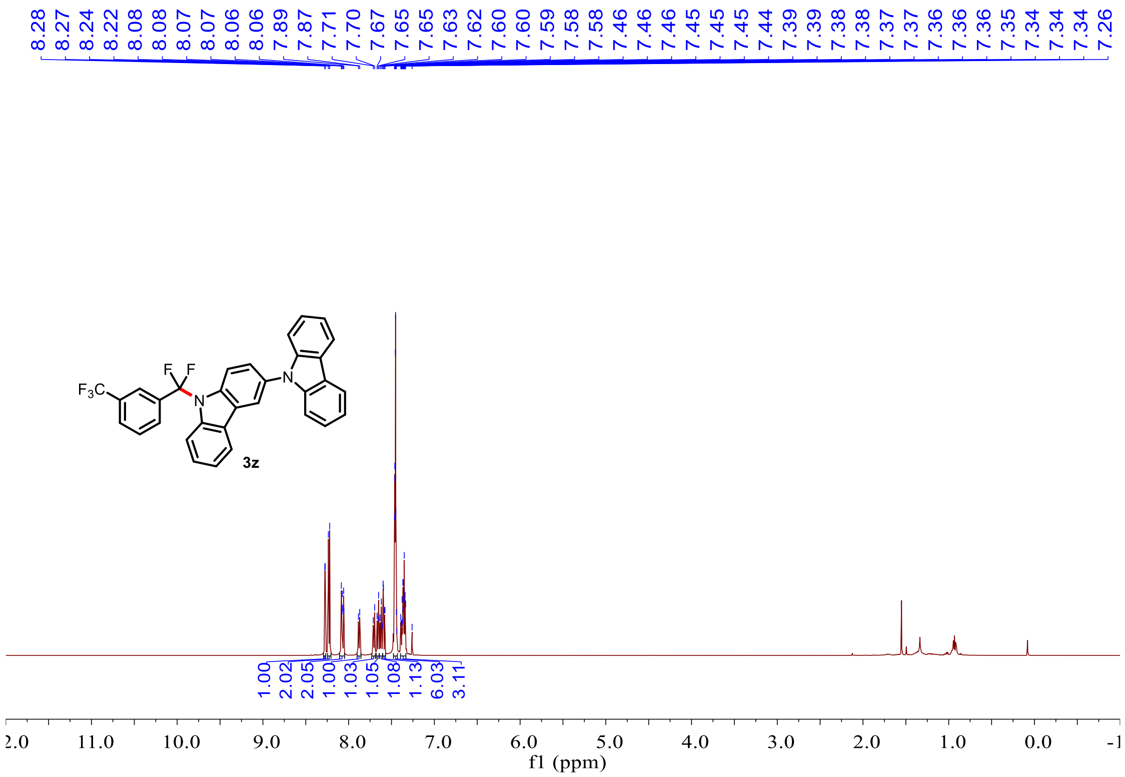

Supplementary Figure 116. **^{1}H NMR** (500 MHz, $CDCl_3$) spectrum for compound **3z**

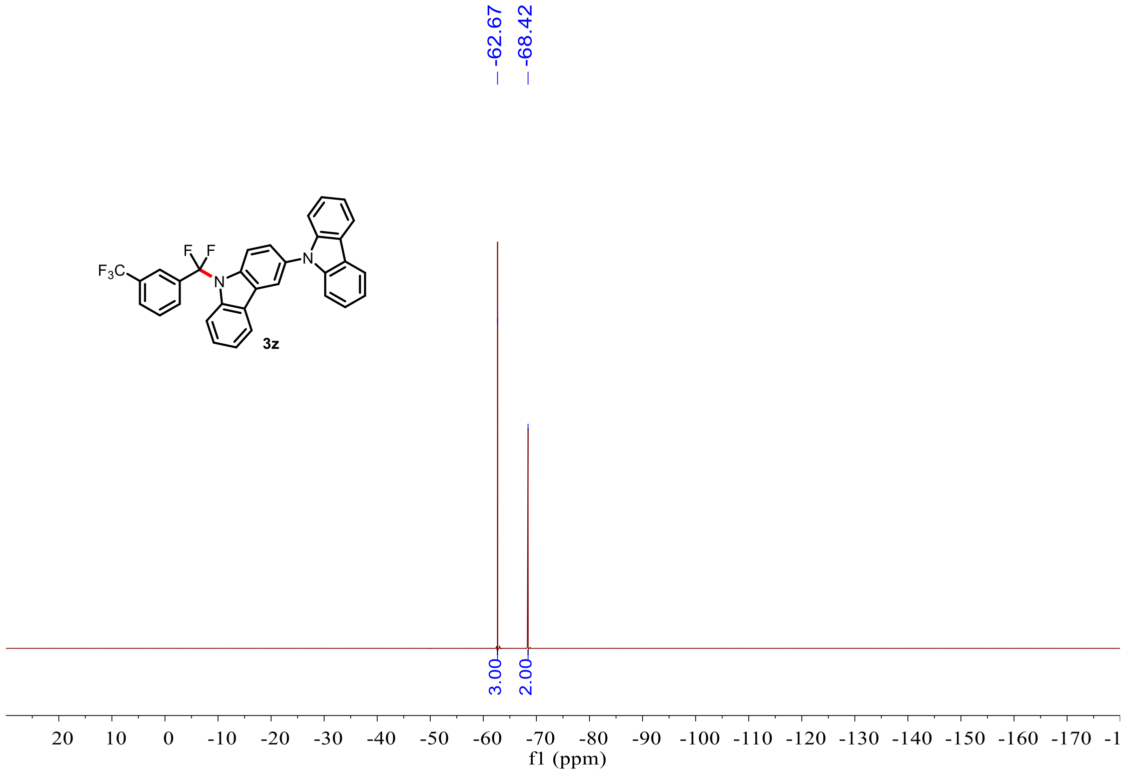

Supplementary Figure 117. **^{19}F NMR** (471 MHz, $CDCl_3$) spectrum for compound **3z**

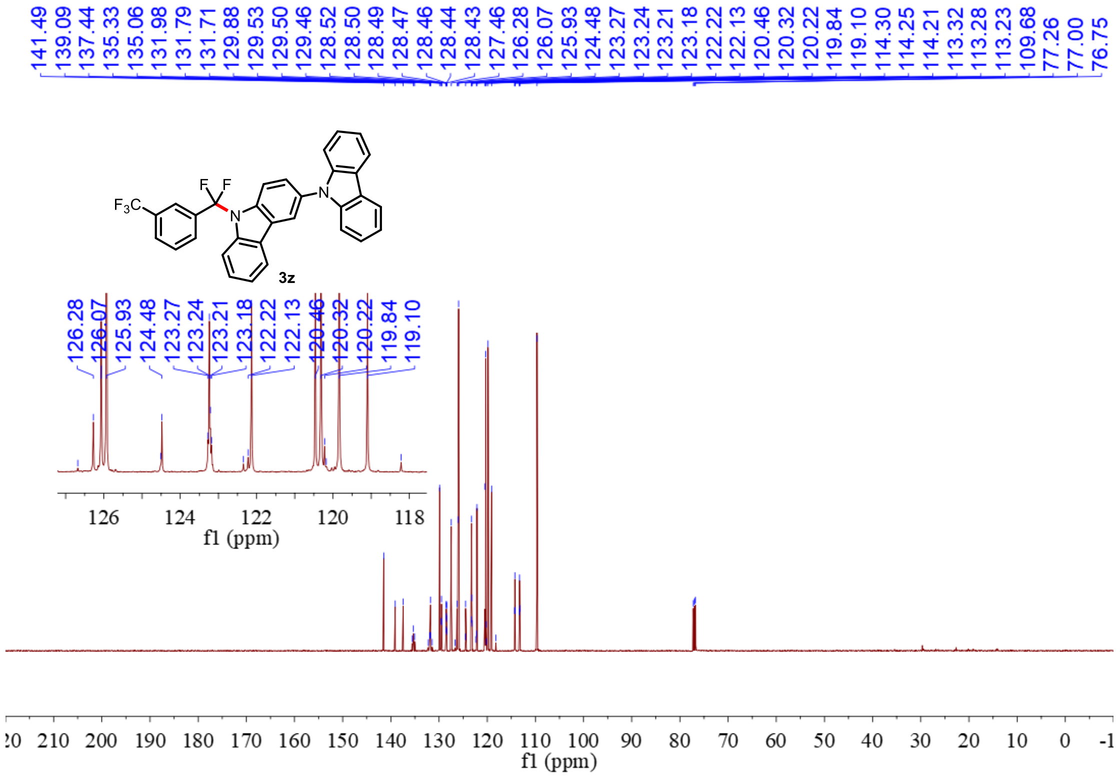

Supplementary Figure 118. ^{13}**C NMR** (126 MHz, $CDCl_3$) spectrum for compound **3z**

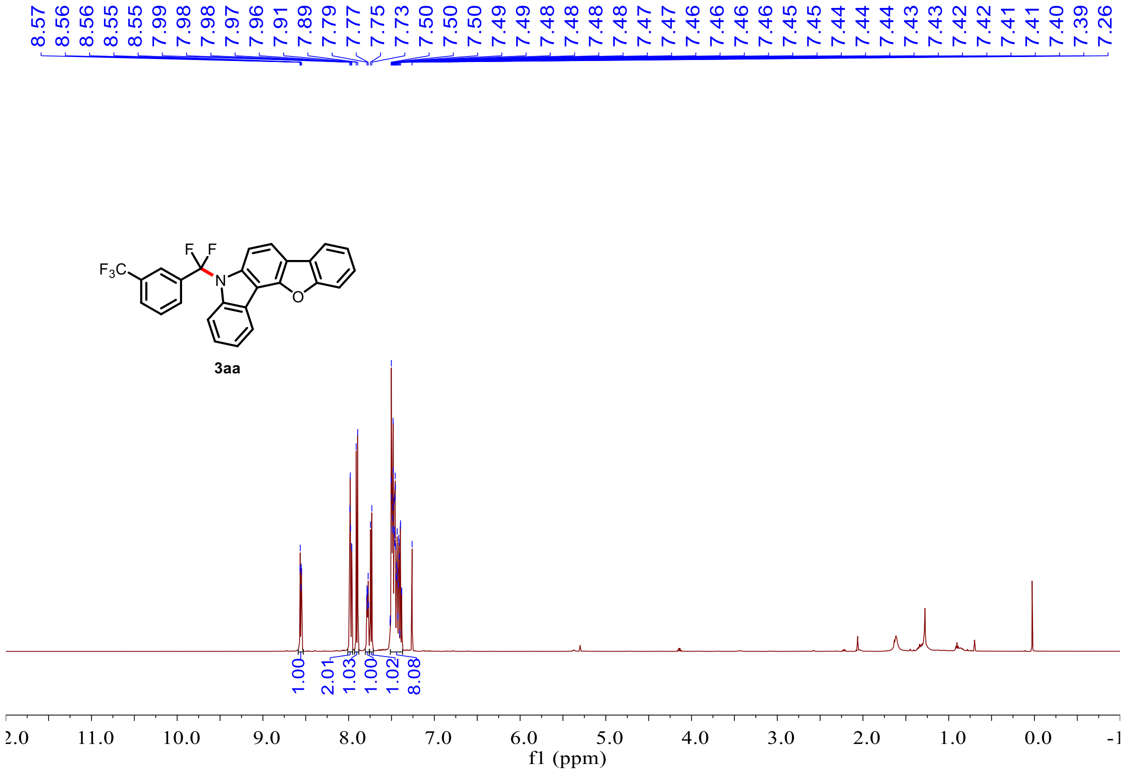

Supplementary Figure 119. ^{1}**H NMR** (500 MHz, $CDCl_3$) spectrum for compound **3aa**

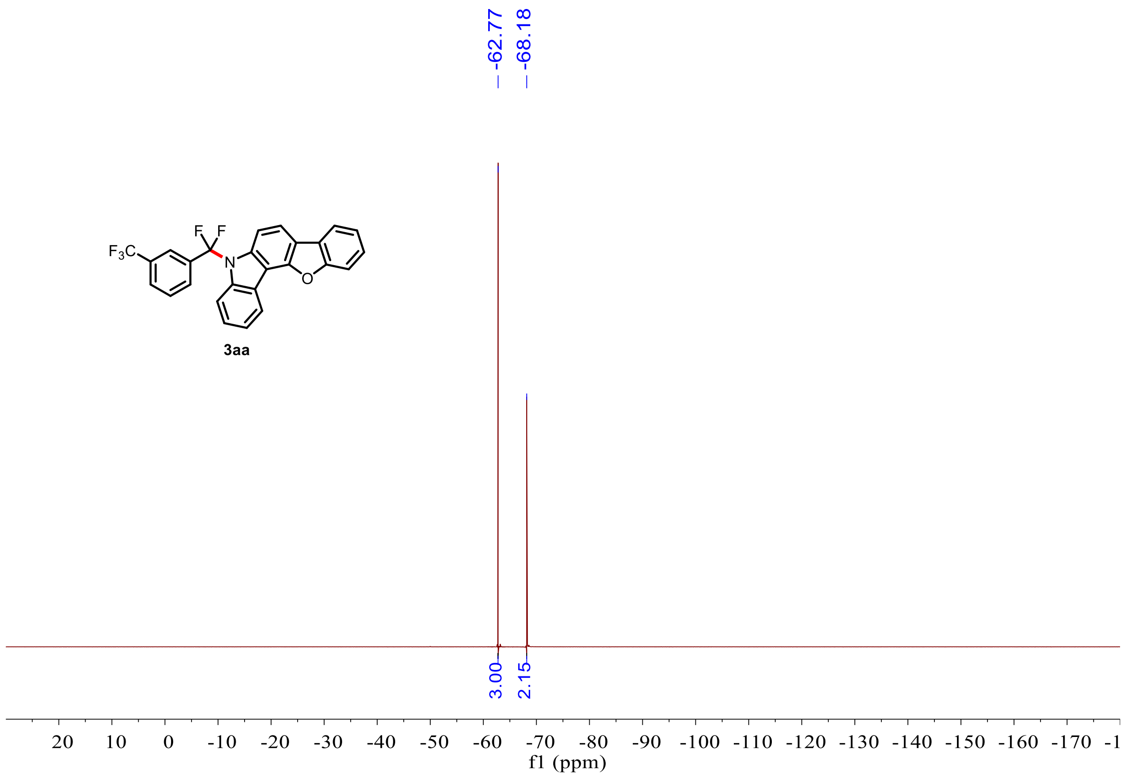

Supplementary Figure 120. ^{19}F NMR (471 MHz, $CDCl_3$) spectrum for compound **3aa**

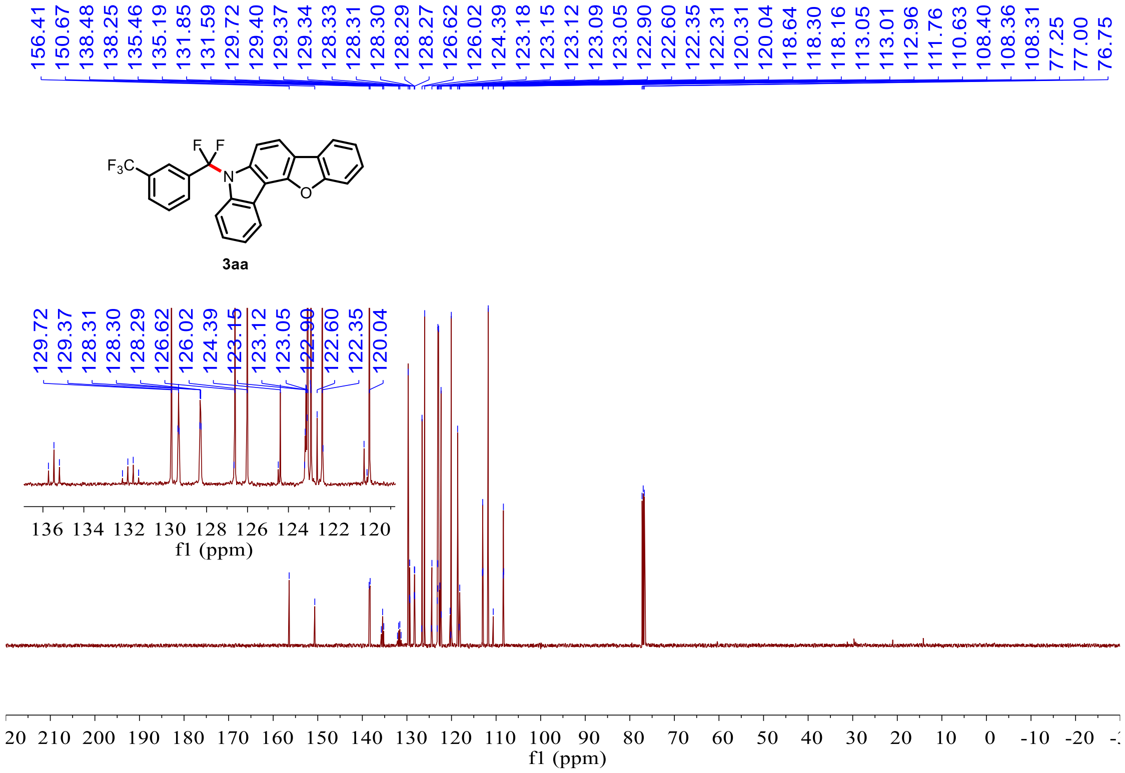

Supplementary Figure 121. ^{13}C NMR (126 MHz, $CDCl_3$) spectrum for compound **3aa**

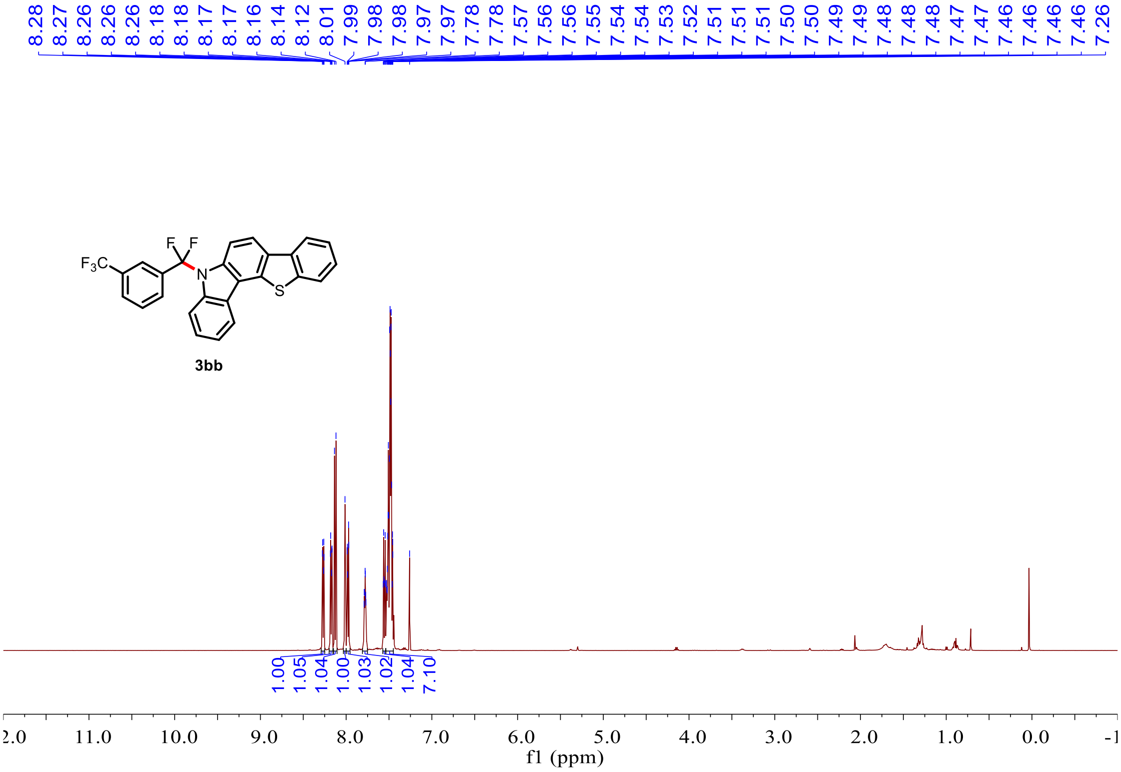

Supplementary Figure 122. ^{1}H NMR (500 MHz, $CDCl_3$) spectrum for compound **3bb**

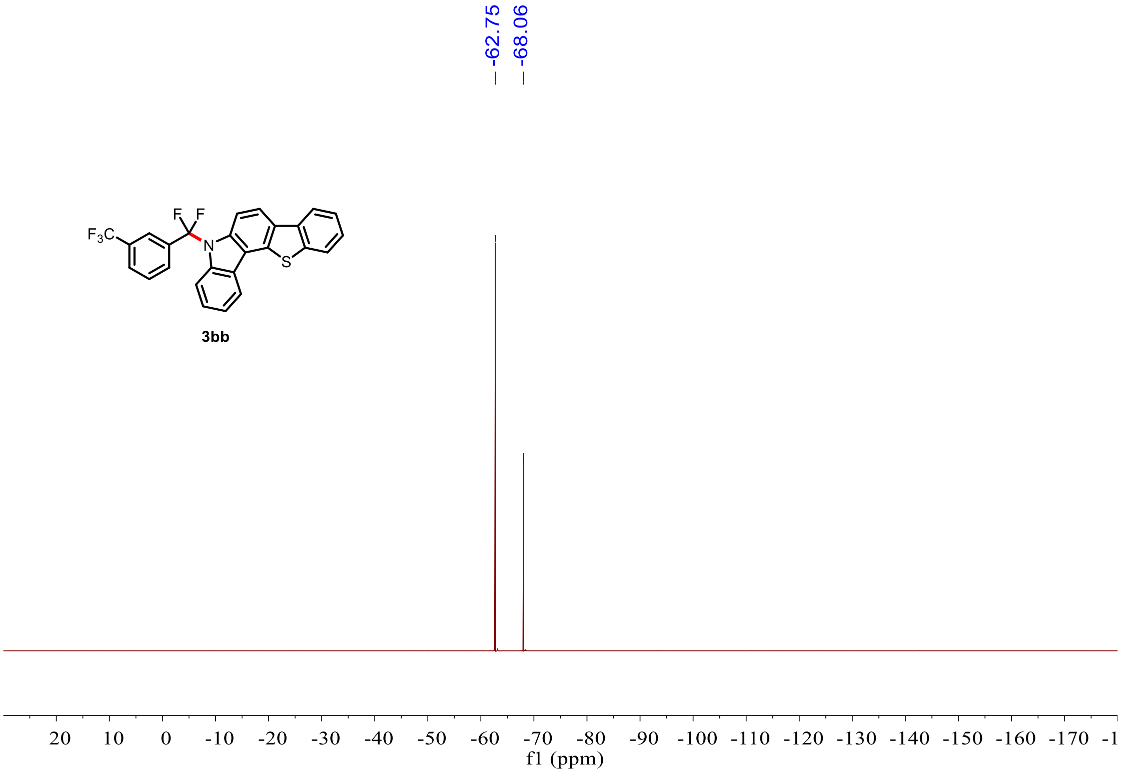

Supplementary Figure 123. ^{19}F NMR (471 MHz, $CDCl_3$) spectrum for compound **3bb**

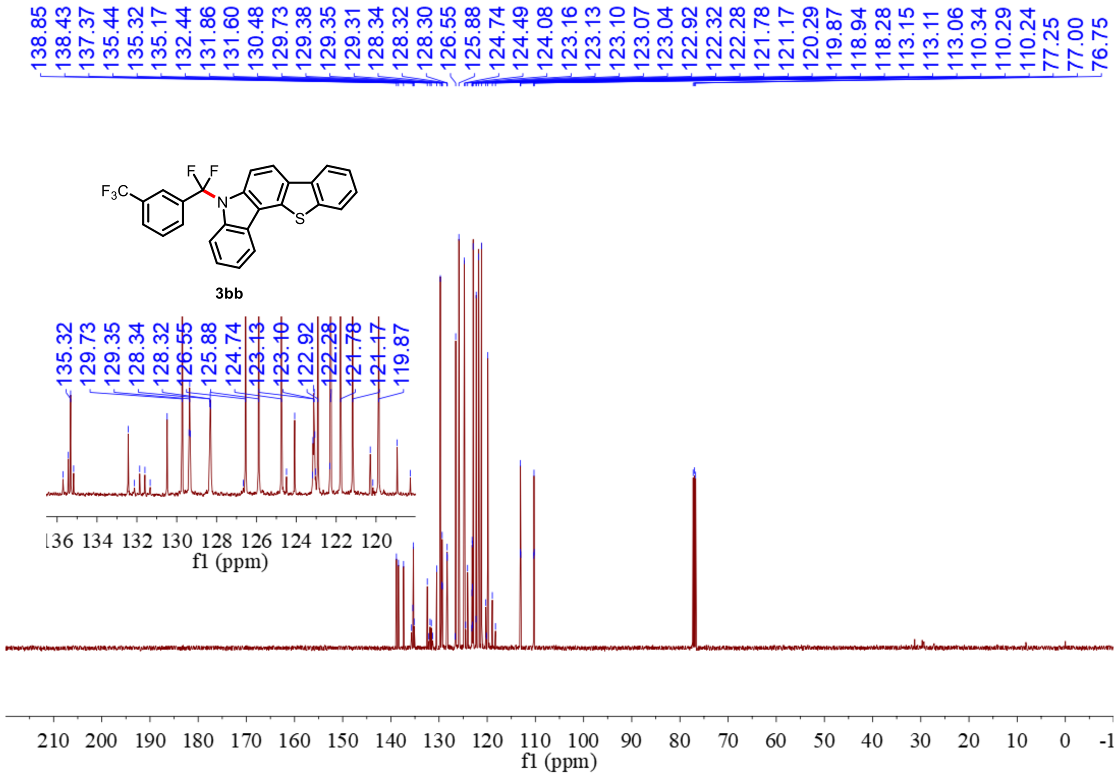

Supplementary Figure 124. ^{13}**C NMR** (126 MHz, $CDCl_3$) spectrum for compound **3bb**

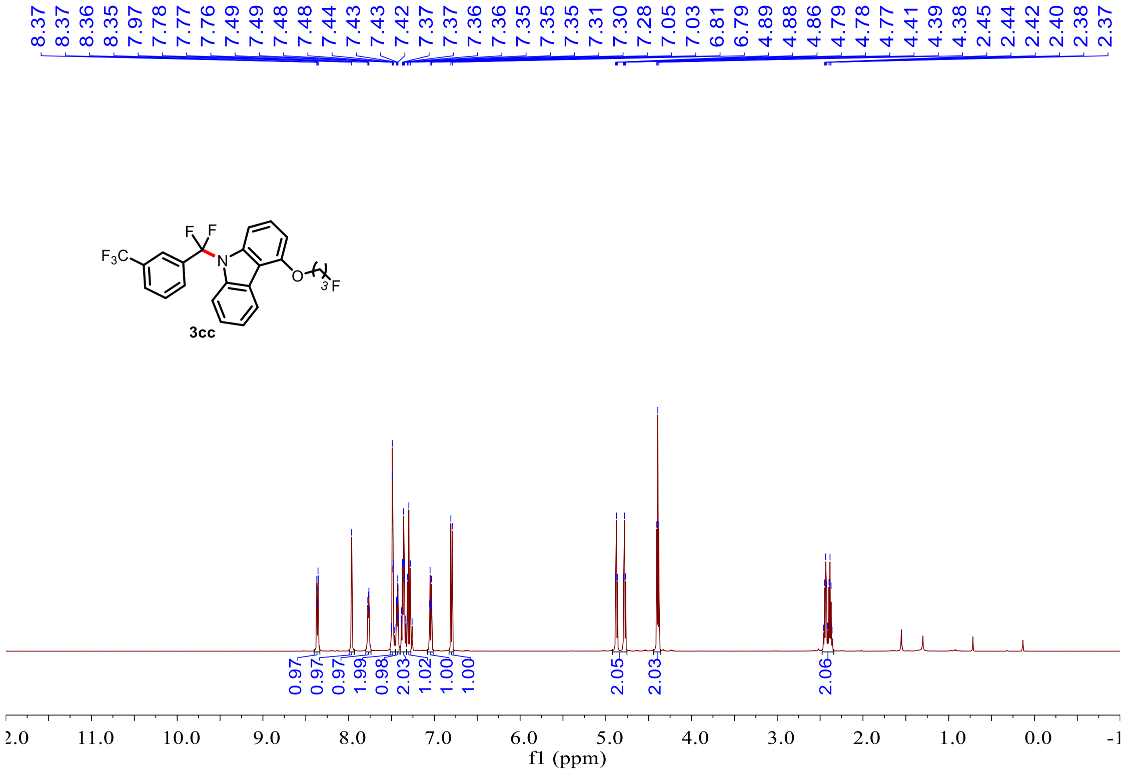

Supplementary Figure 125. ^{1}**H NMR** (500 MHz, $CDCl_3$) spectrum for compound **3cc**

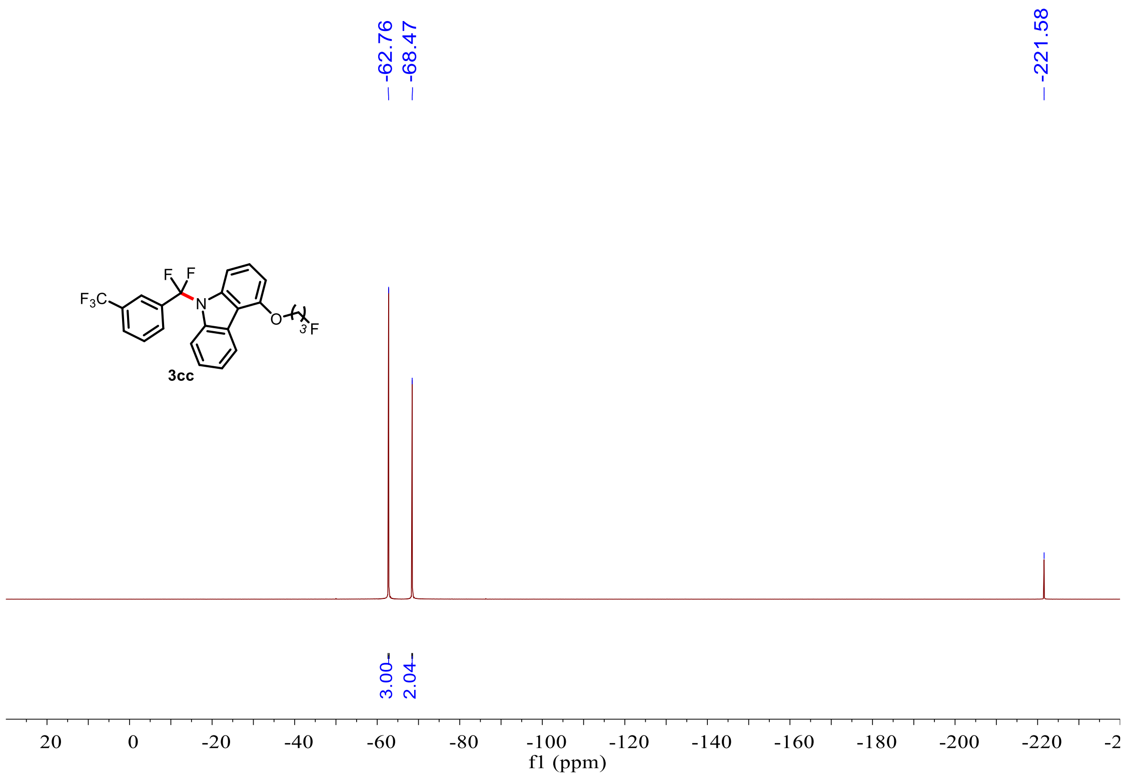

Supplementary Figure 126. ^{19}**F NMR** (471 MHz, CDCl$_3$) spectrum for compound **3cc**

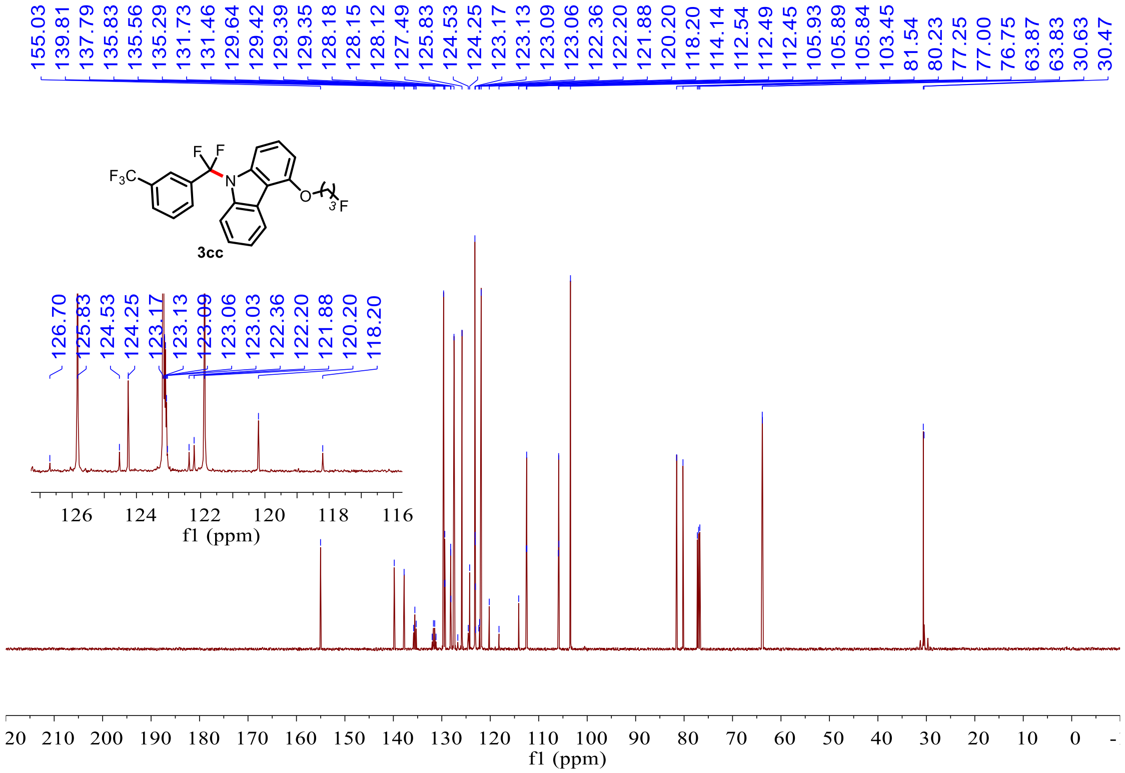

Supplementary Figure 127. ^{13}**C NMR** (126 MHz, CDCl$_3$) spectrum for compound **3cc**

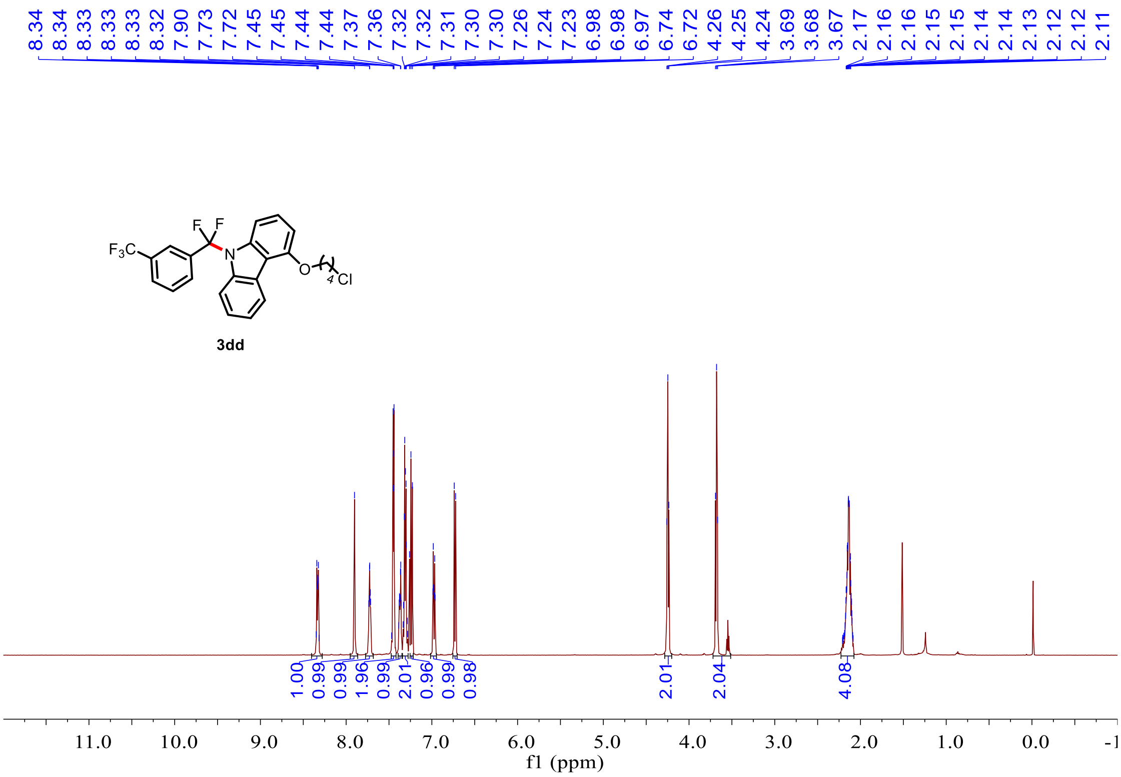

Supplementary Figure 128. ^{1}H NMR (500 MHz, $CDCl_3$) spectrum for compound **3dd**

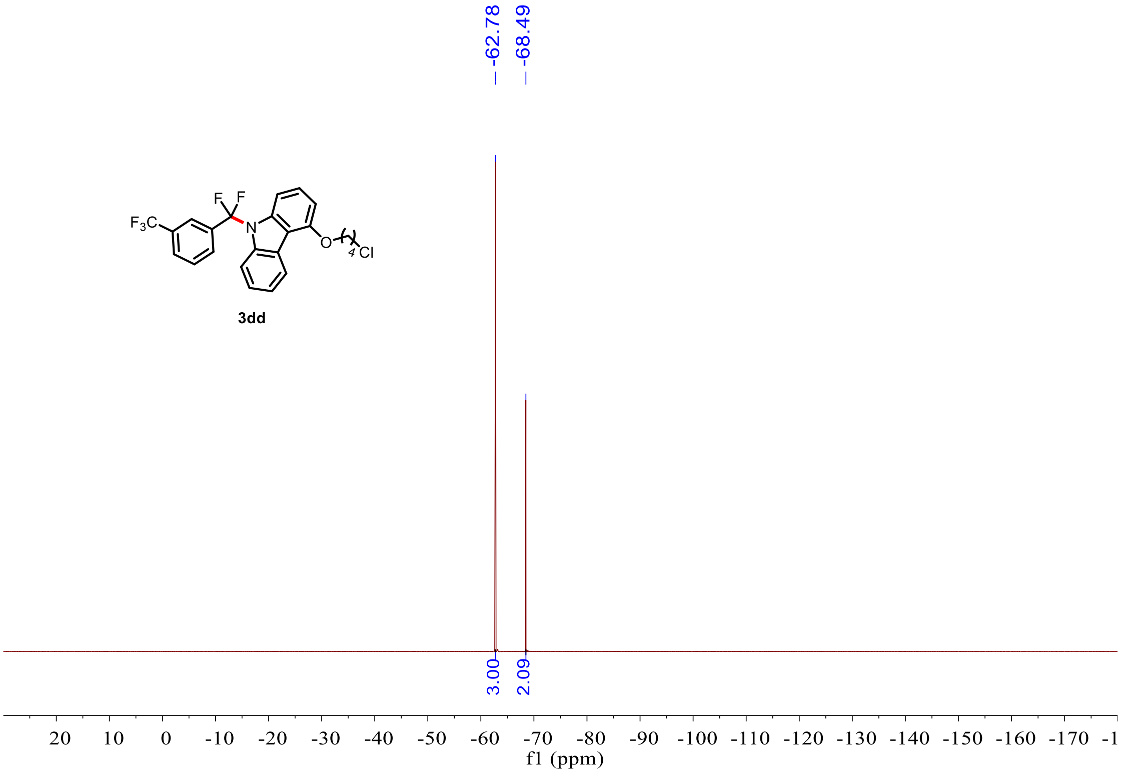

Supplementary Figure 129. ^{19}F NMR (471 MHz, $CDCl_3$) spectrum for compound **3dd**

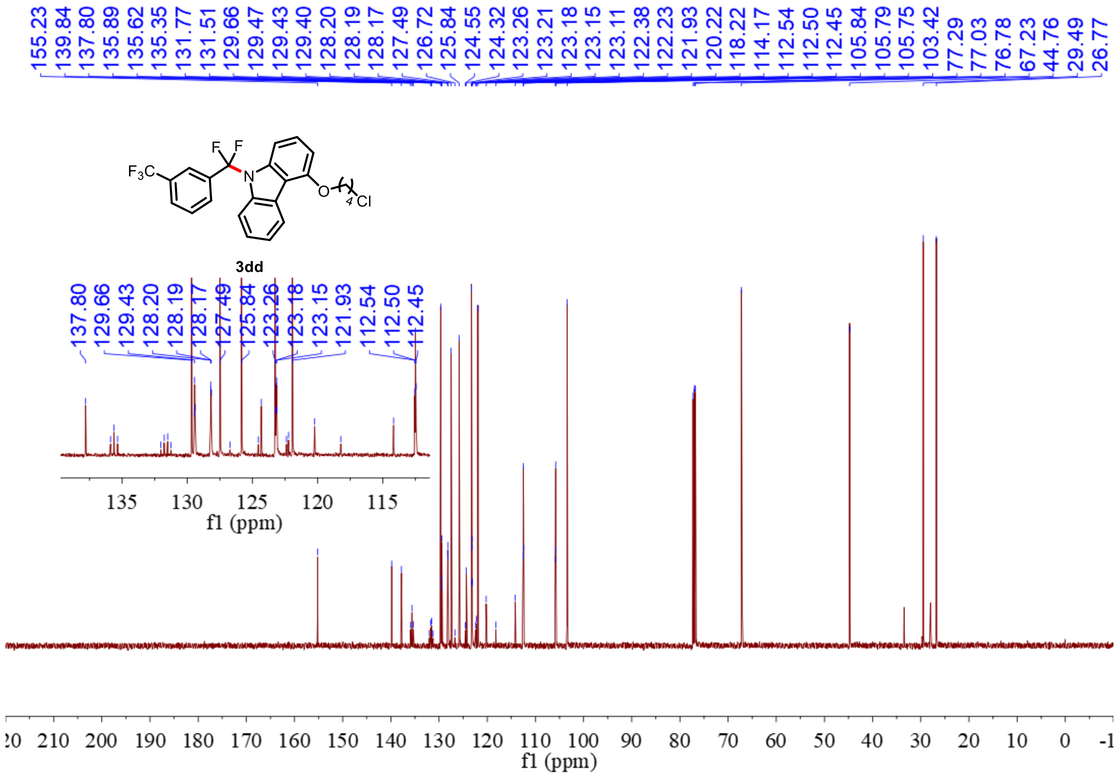

Supplementary Figure 130. ^{13}**C NMR** (126 MHz, $CDCl_3$) spectrum for compound **3dd**

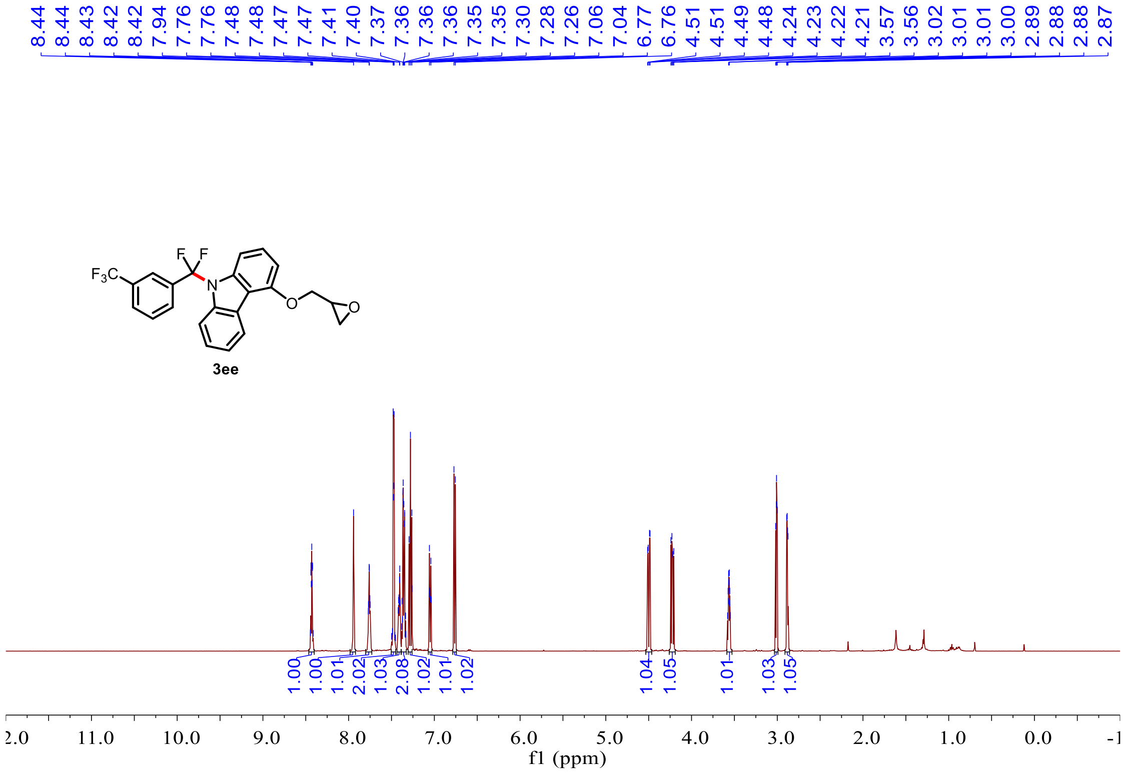

Supplementary Figure 131. ^{1}**H NMR** (500 MHz, $CDCl_3$) spectrum for compound **3ee**

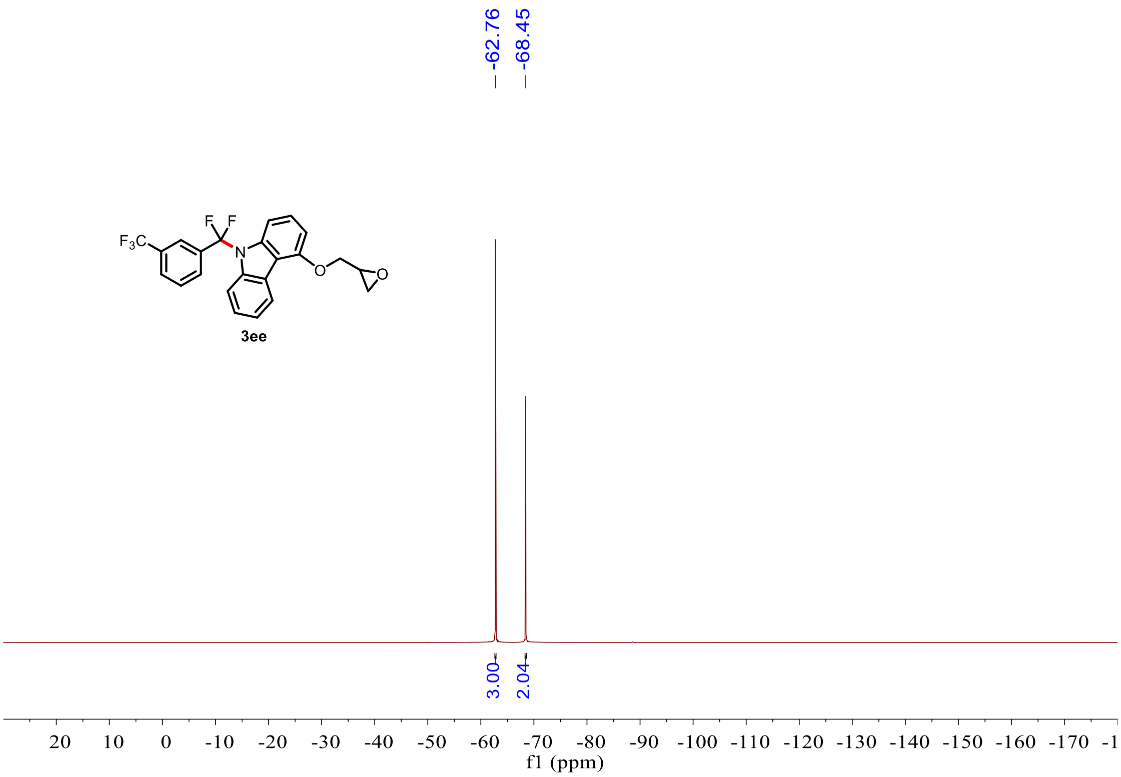

Supplementary Figure 132. ^{19}F NMR (471 MHz, $CDCl_3$) spectrum for compound **3ee**

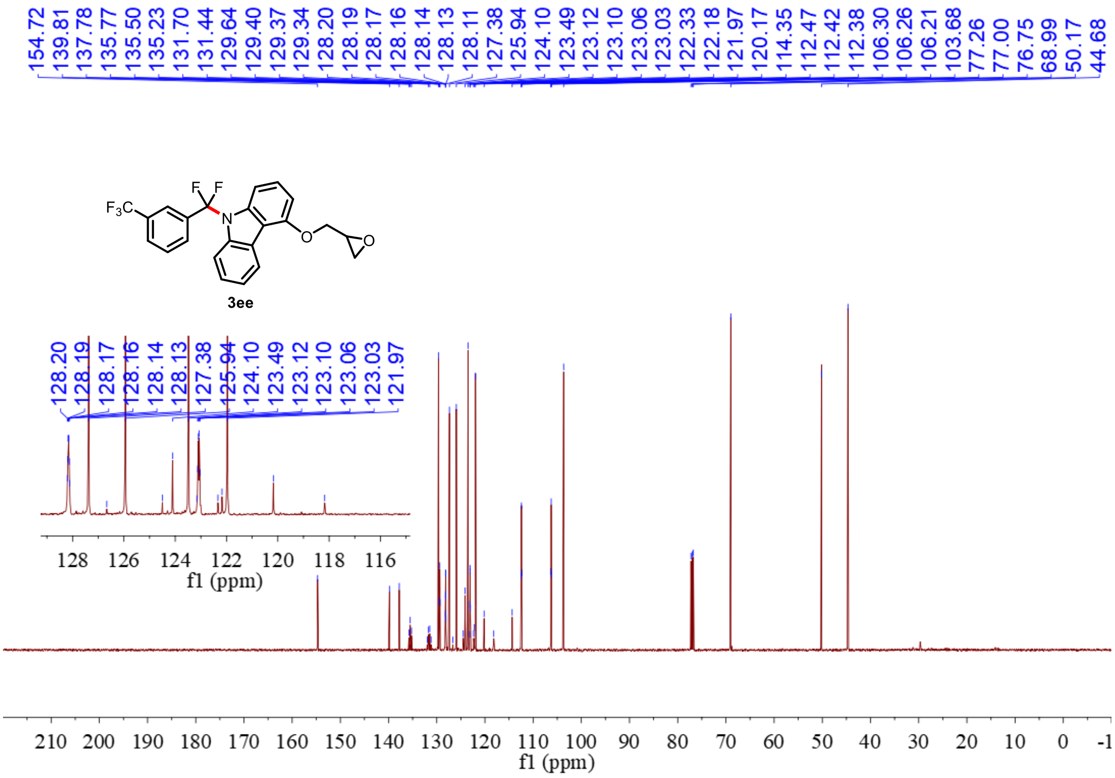

Supplementary Figure 133. ^{13}C NMR (126 MHz, $CDCl_3$) spectrum for compound **3ee**

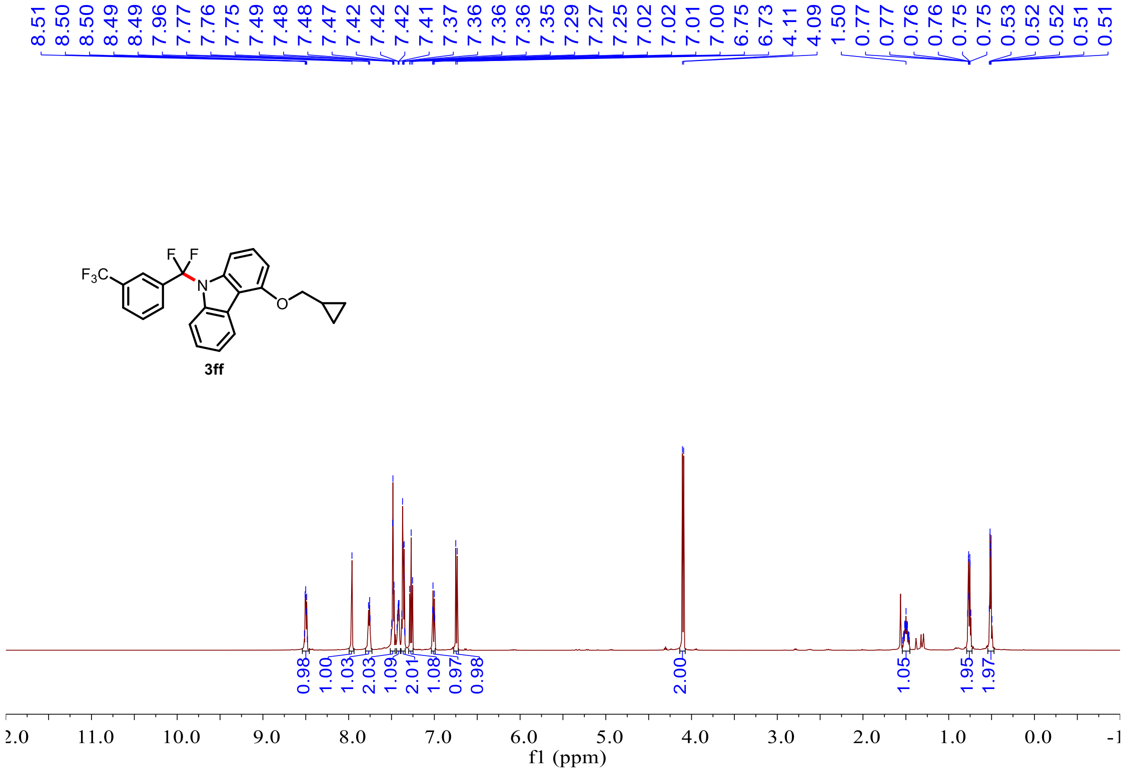

Supplementary Figure 134. ^{1}**H NMR** (500 MHz, $CDCl_3$) spectrum for compound **3ff**

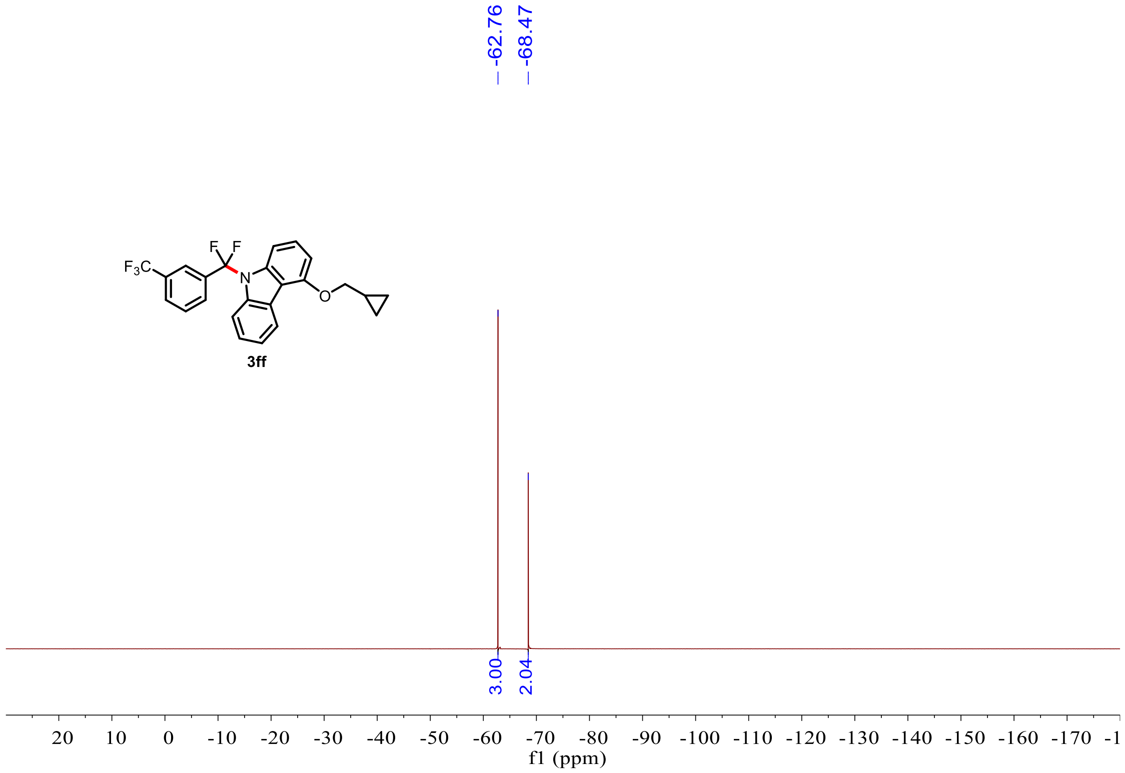

Supplementary Figure 135. ^{19}**F NMR** (471 MHz, $CDCl_3$) spectrum for compound **3ff**

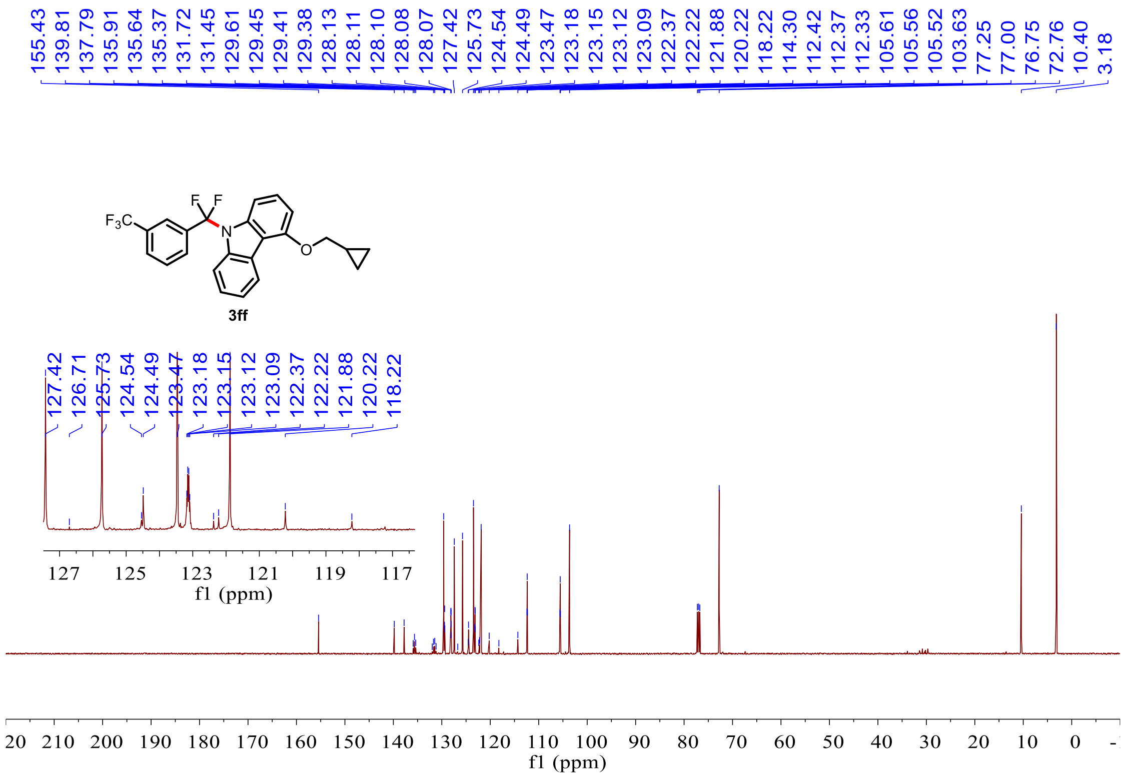

Supplementary Figure 136. ^{13}C NMR (126 MHz, $CDCl_3$) spectrum for compound **3ff**

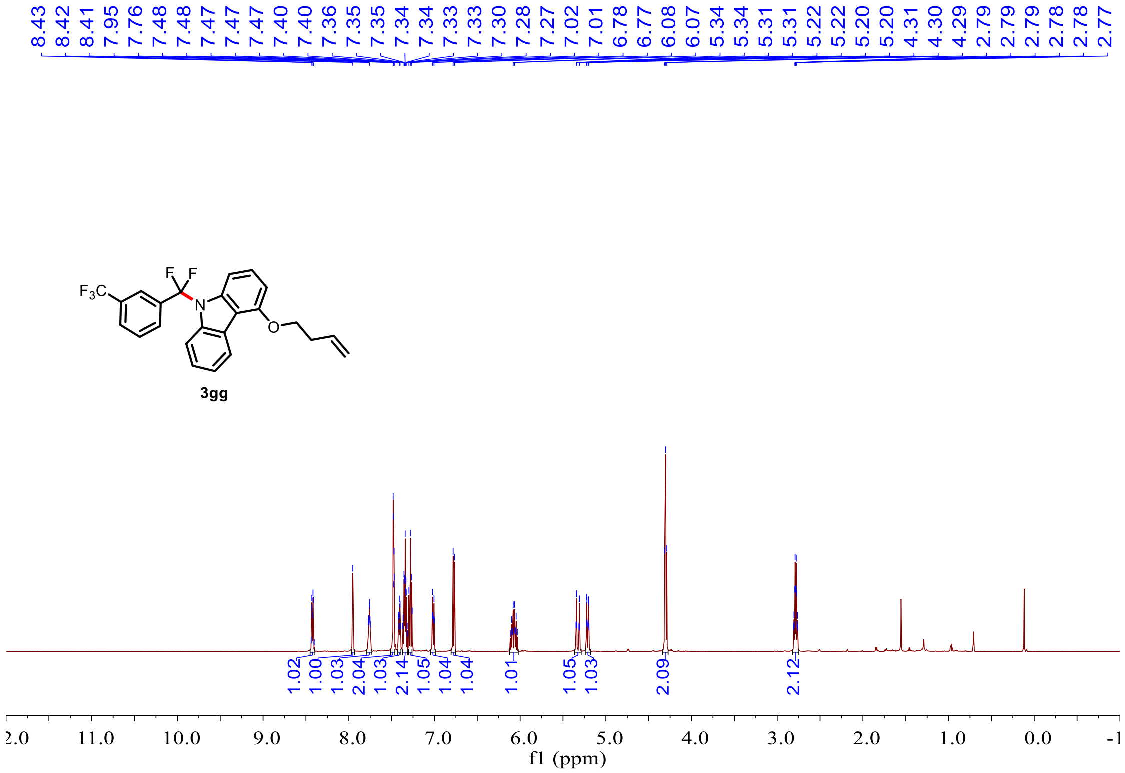

Supplementary Figure 137. ^{1}H NMR (500 MHz, $CDCl_3$) spectrum for compound **3gg**

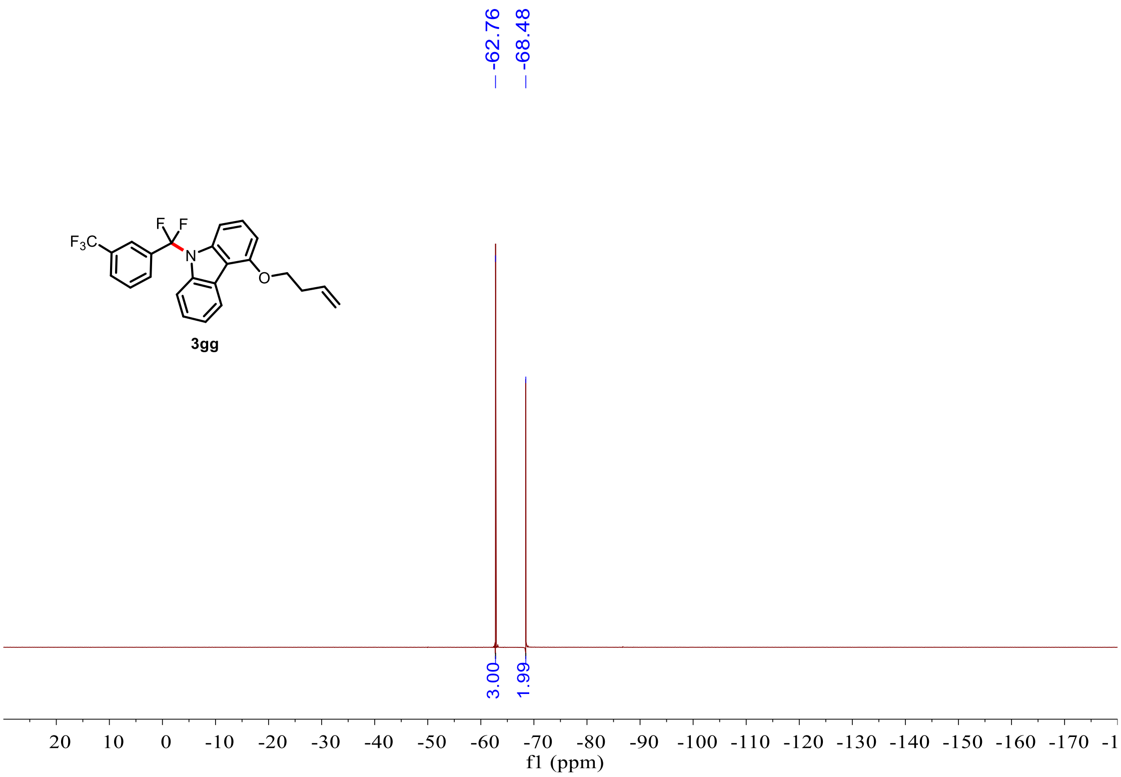

Supplementary Figure 138. ^{19}**F NMR** (471 MHz, $CDCl_3$) spectrum for compound **3gg**

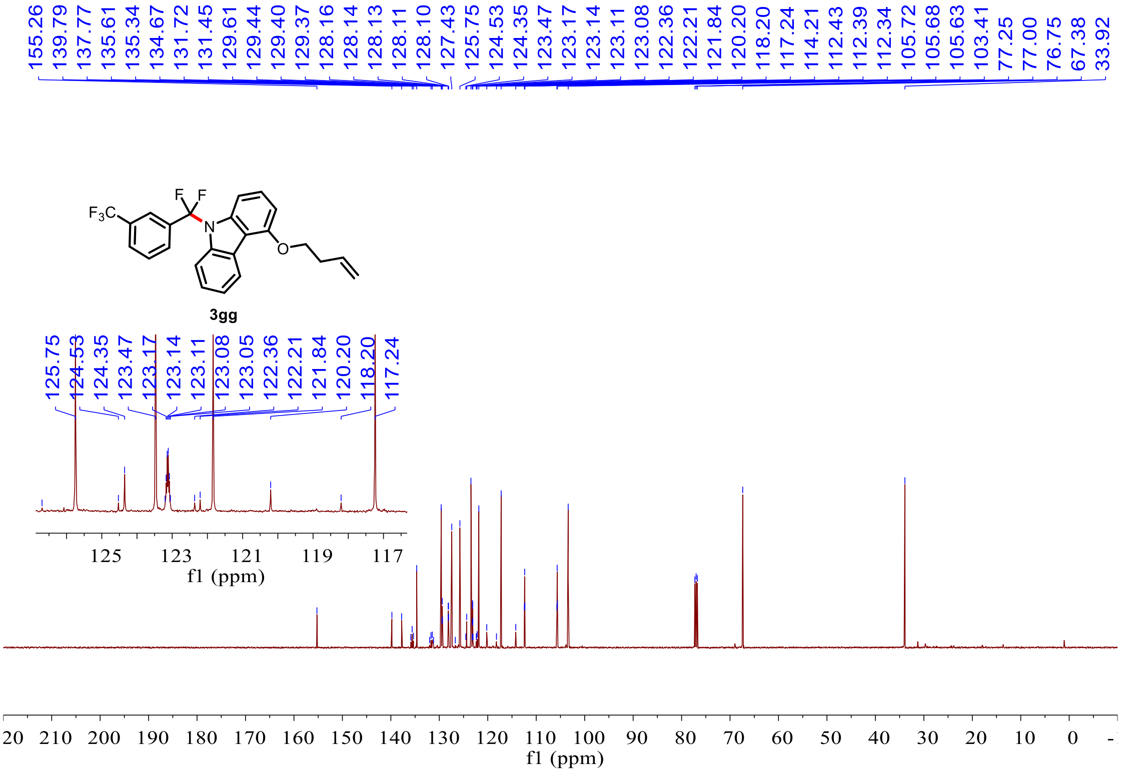

Supplementary Figure 139. ^{13}**C NMR** (126 MHz, $CDCl_3$) spectrum for compound **3gg**

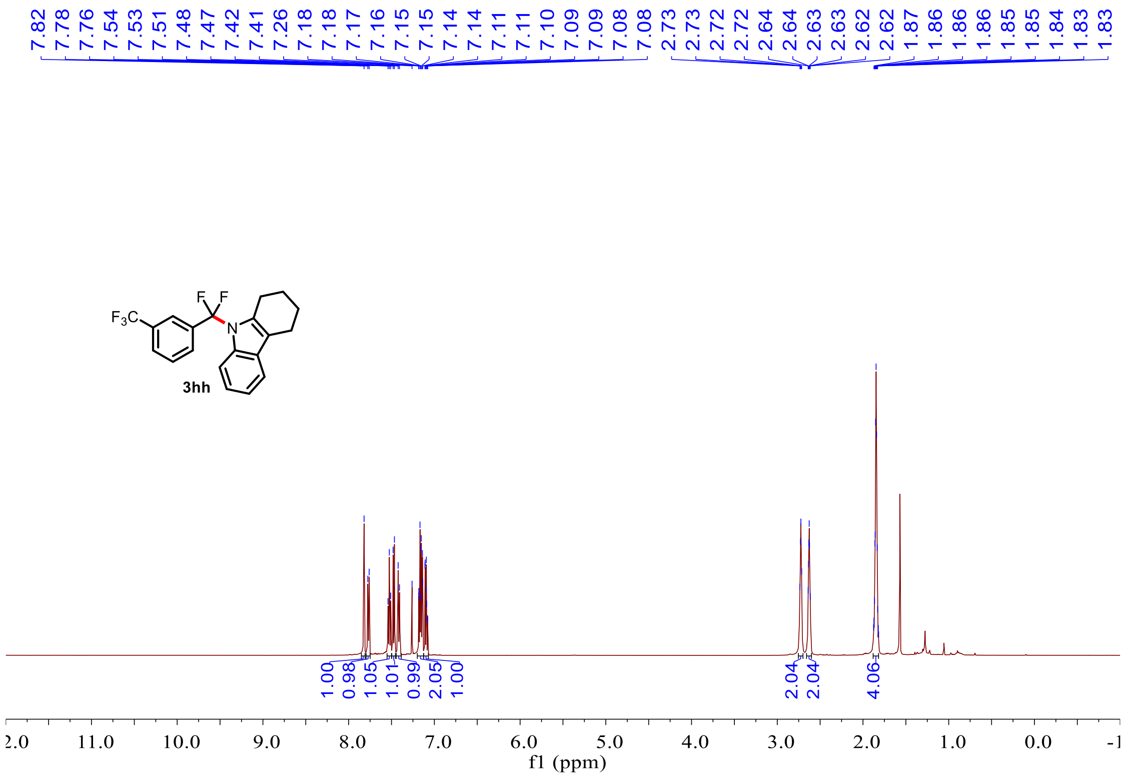

Supplementary Figure 140. ^{1}H NMR (500 MHz, $CDCl_3$) spectrum for compound **3hh**

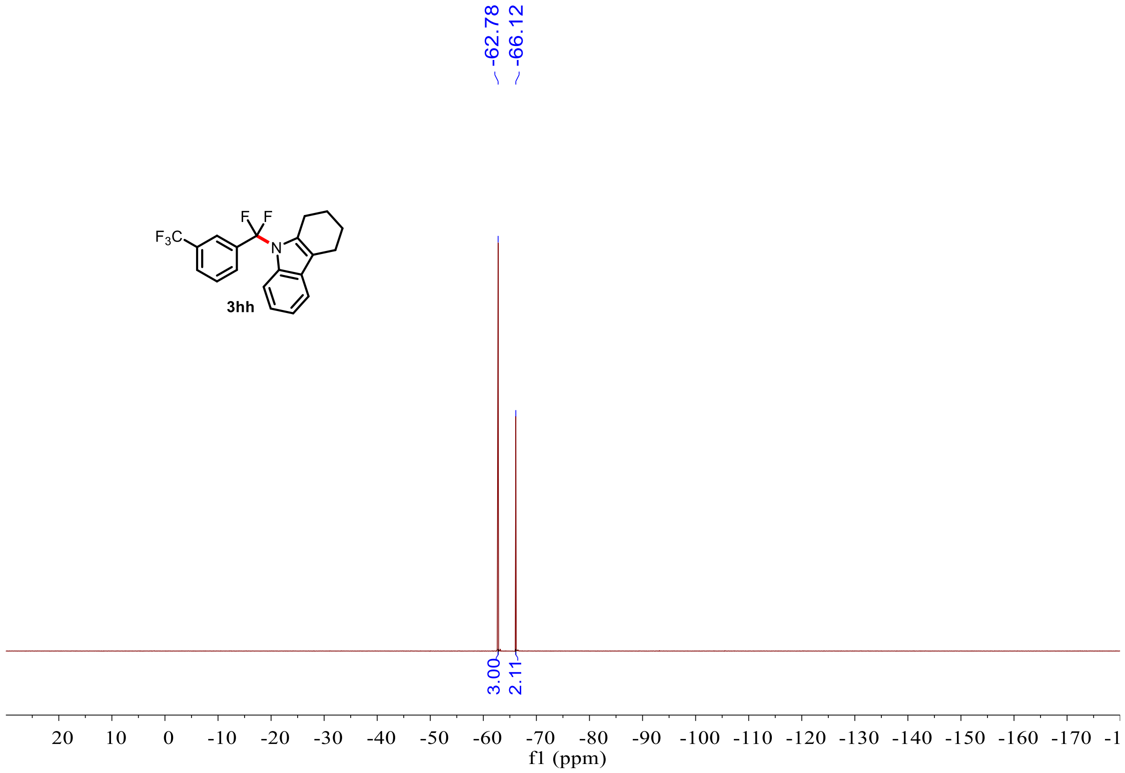

Supplementary Figure 141. ^{19}F NMR (471 MHz, $CDCl_3$) spectrum for compound **3hh**

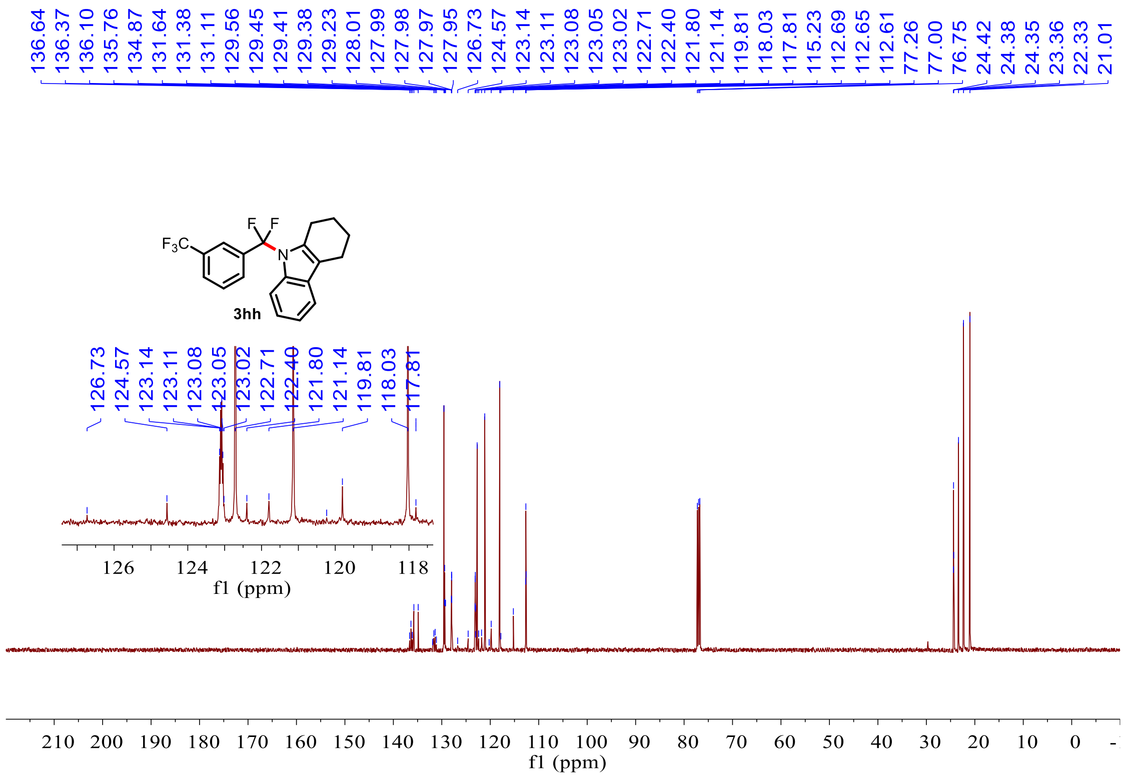

Supplementary Figure 142. ^{13}**C NMR** (126 MHz, CDCl$_3$) spectrum for compound **3hh**

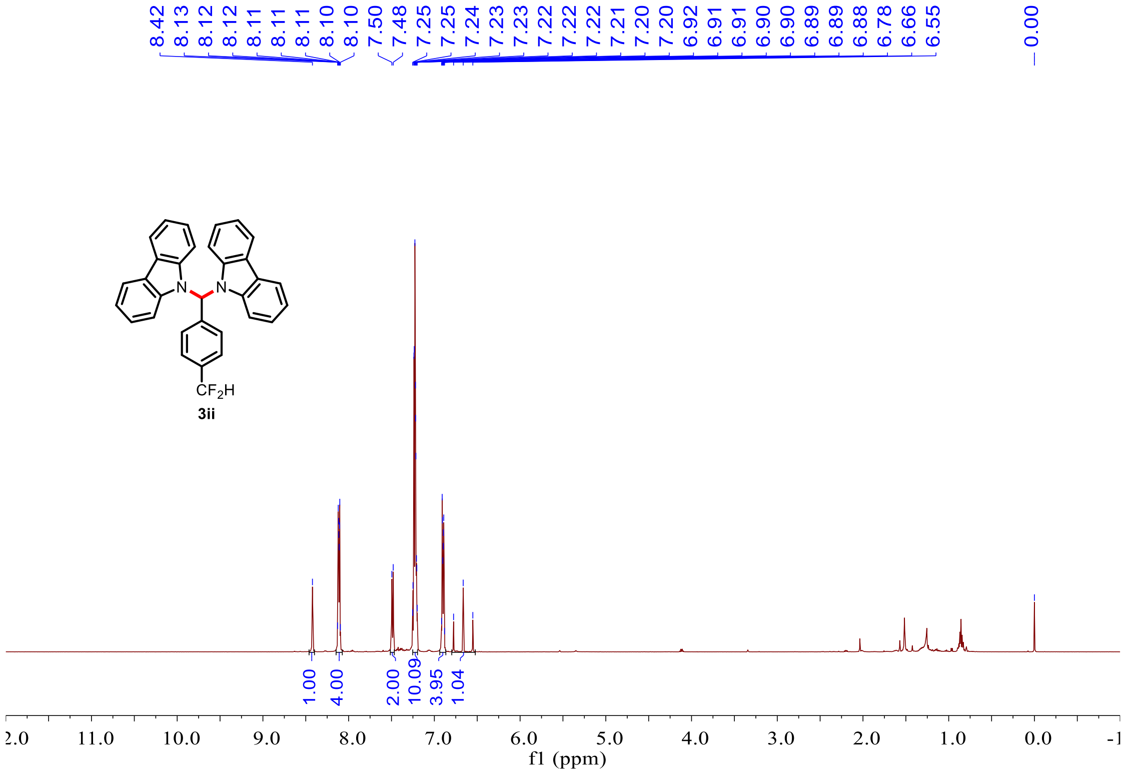

Supplementary Figure 143. ^{1}**H NMR** (500 MHz, CDCl$_3$) spectrum for compound **3ii**

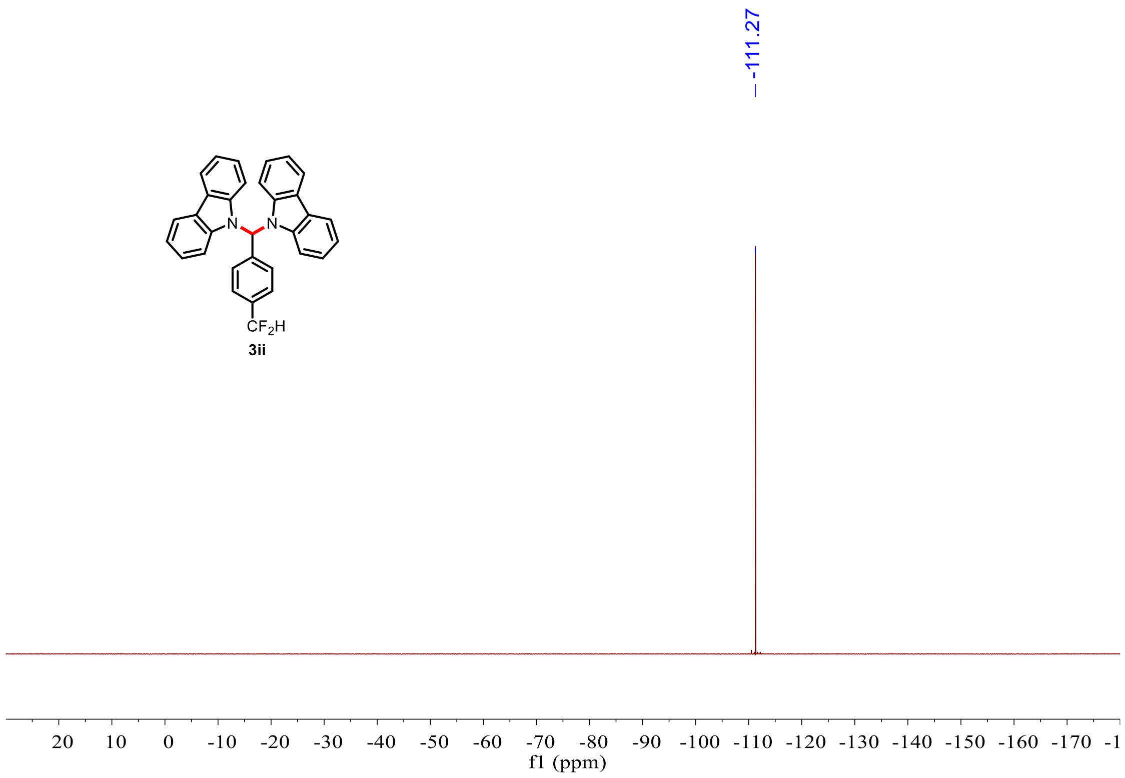

Supplementary Figure 144. ^{19}**F NMR** (471 MHz, $CDCl_3$) spectrum for compound **3ii**

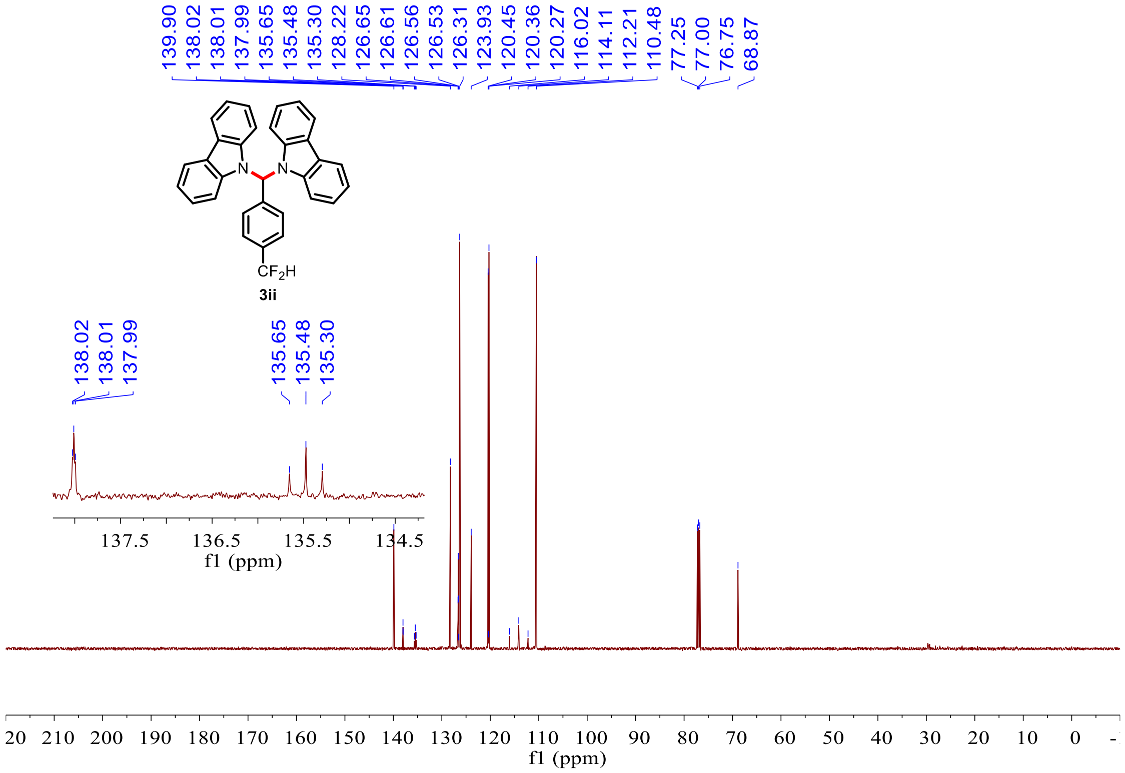

Supplementary Figure 145. ^{13}**C NMR** (126 MHz, $CDCl_3$) spectrum for compound **3ii**

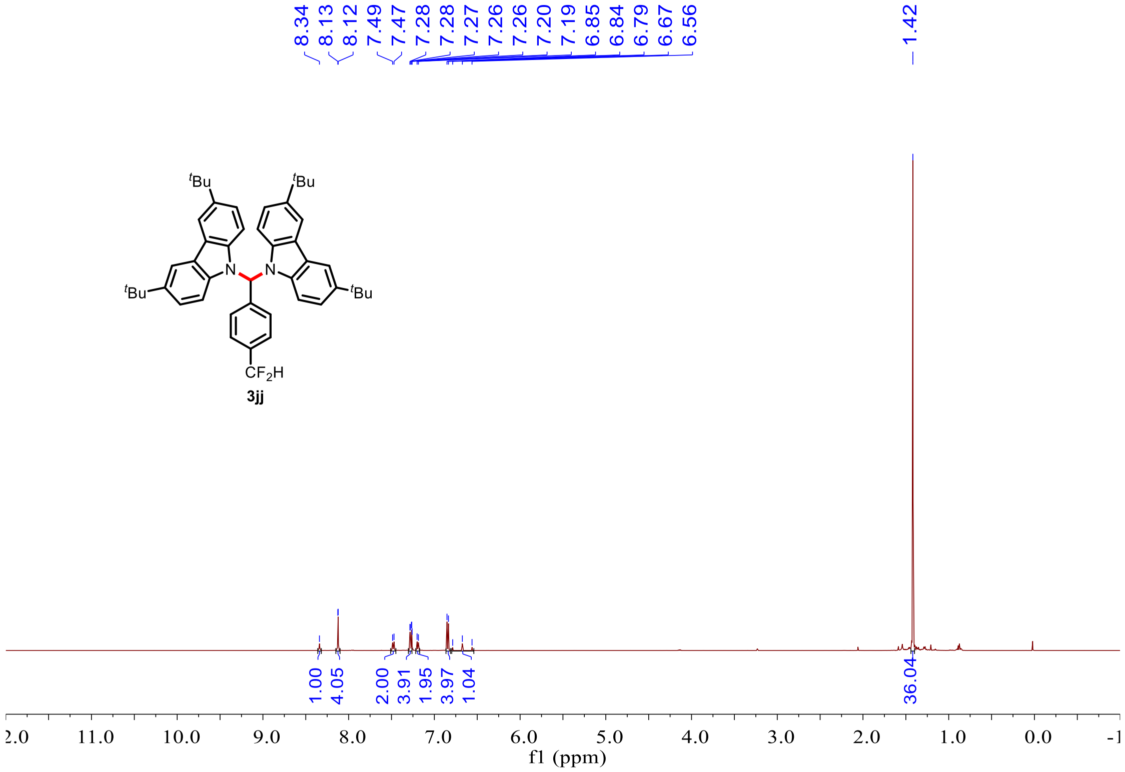

Supplementary Figure 146. ^{1}H NMR (500 MHz, $CDCl_3$) spectrum for compound **3jj**

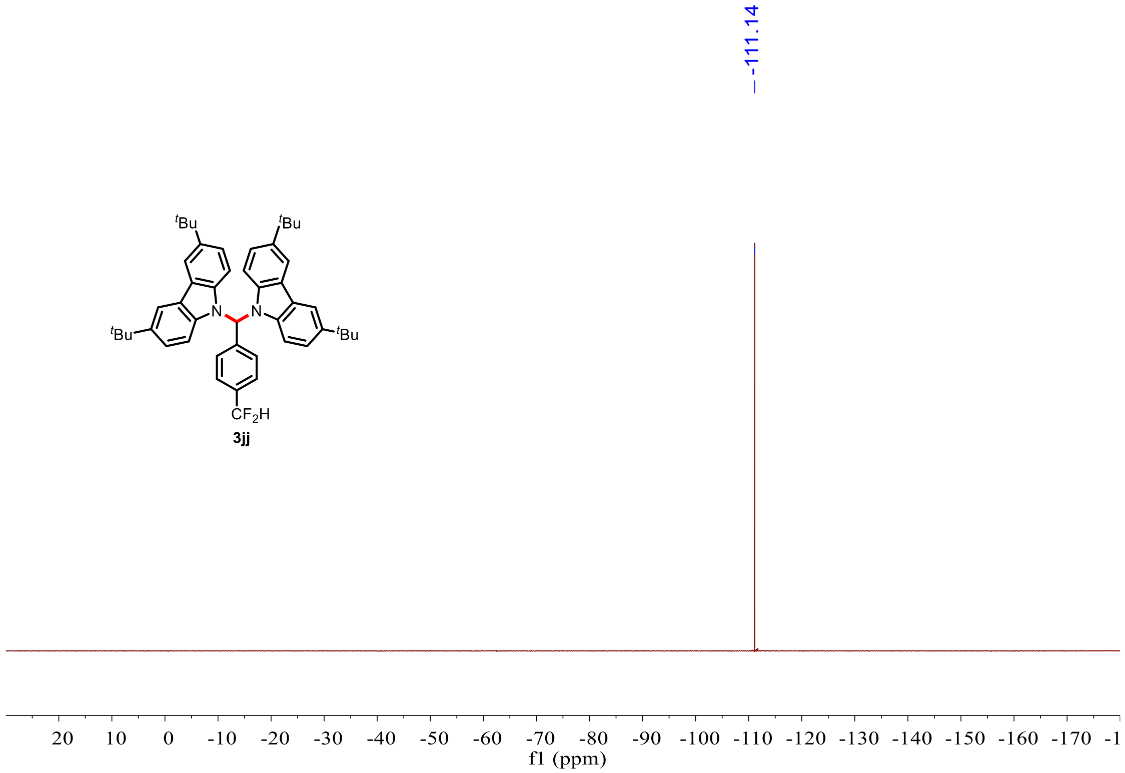

Supplementary Figure 147. ^{19}F NMR (471 MHz, $CDCl_3$) spectrum for compound **3jj**

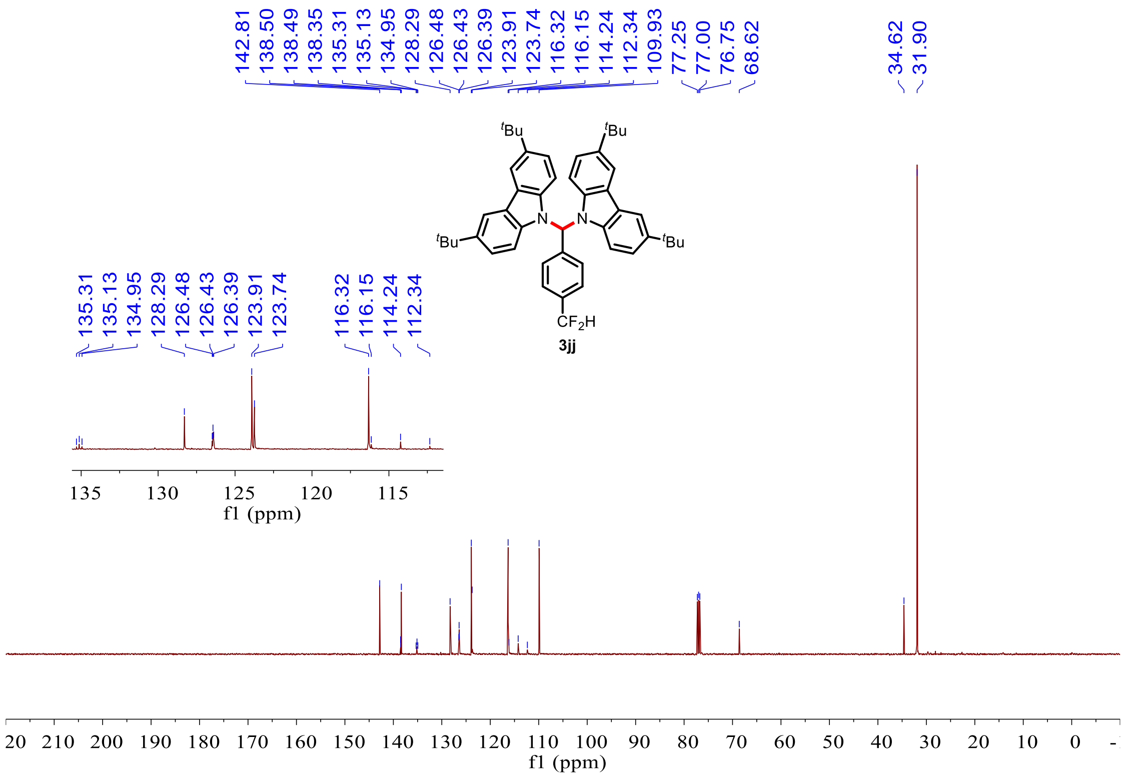

Supplementary Figure 148. ^{13}**C NMR** (126 MHz, $CDCl_3$) spectrum for compound **3jj**

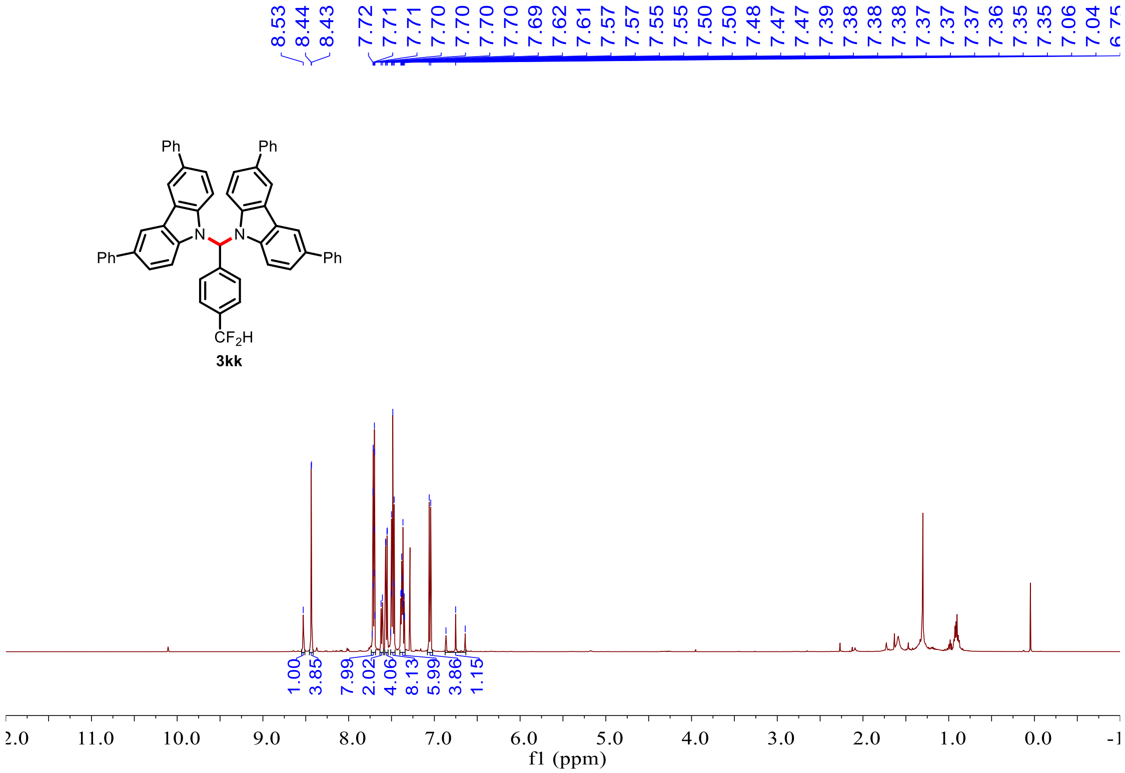

Supplementary Figure 149. ^{1}**H NMR** (500 MHz, $CDCl_3$) spectrum for compound **3kk**

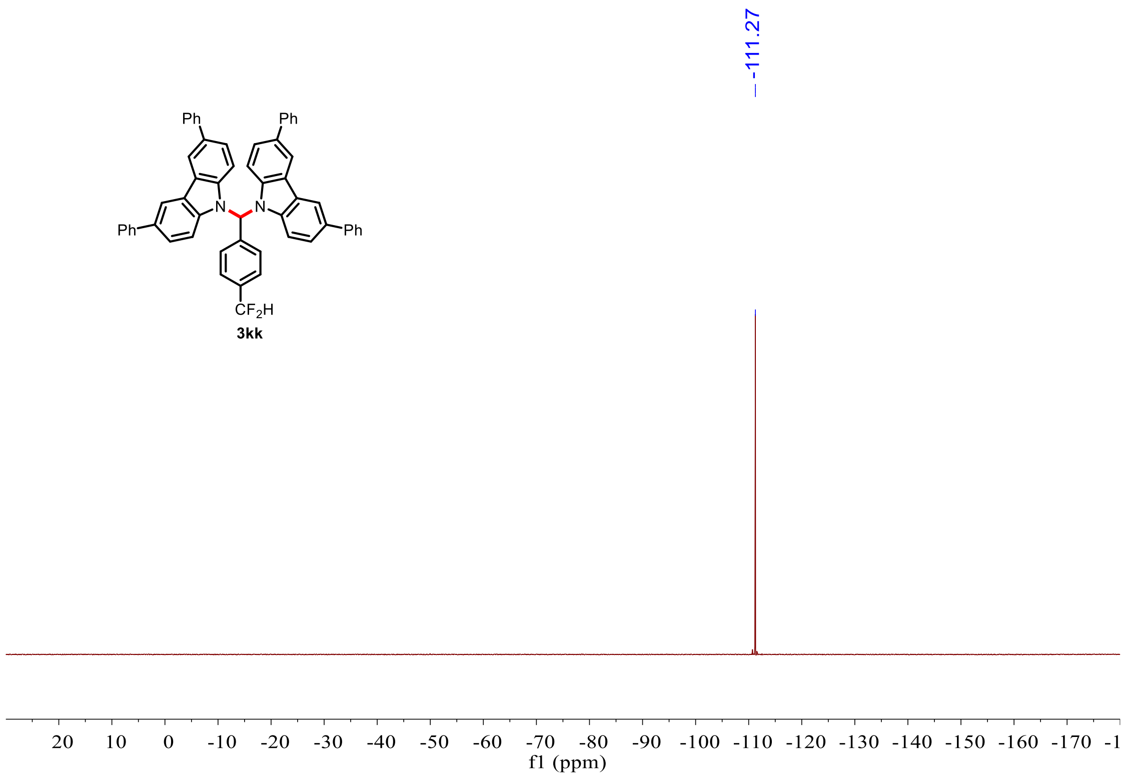

Supplementary Figure 150. ^{19}**F NMR** (471 MHz, $CDCl_3$) spectrum for compound **3kk**

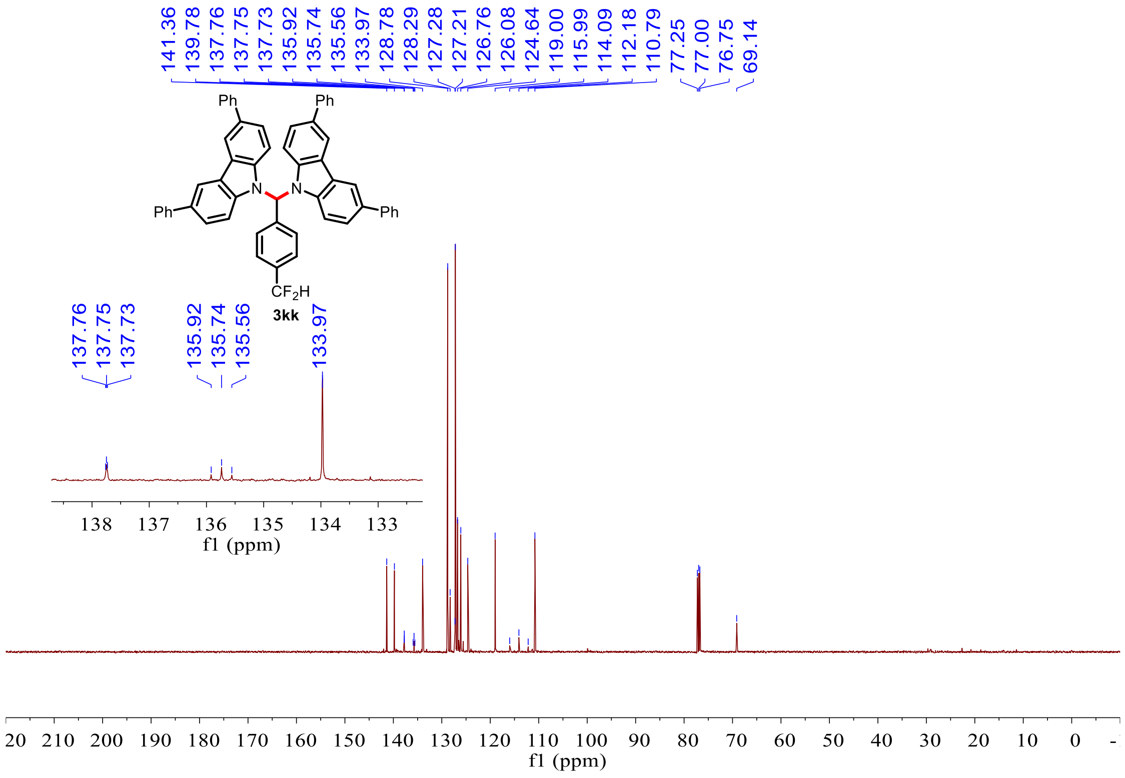

Supplementary Figure 151. ^{13}**C NMR** (126 MHz, $CDCl_3$) spectrum for compound **3kk**

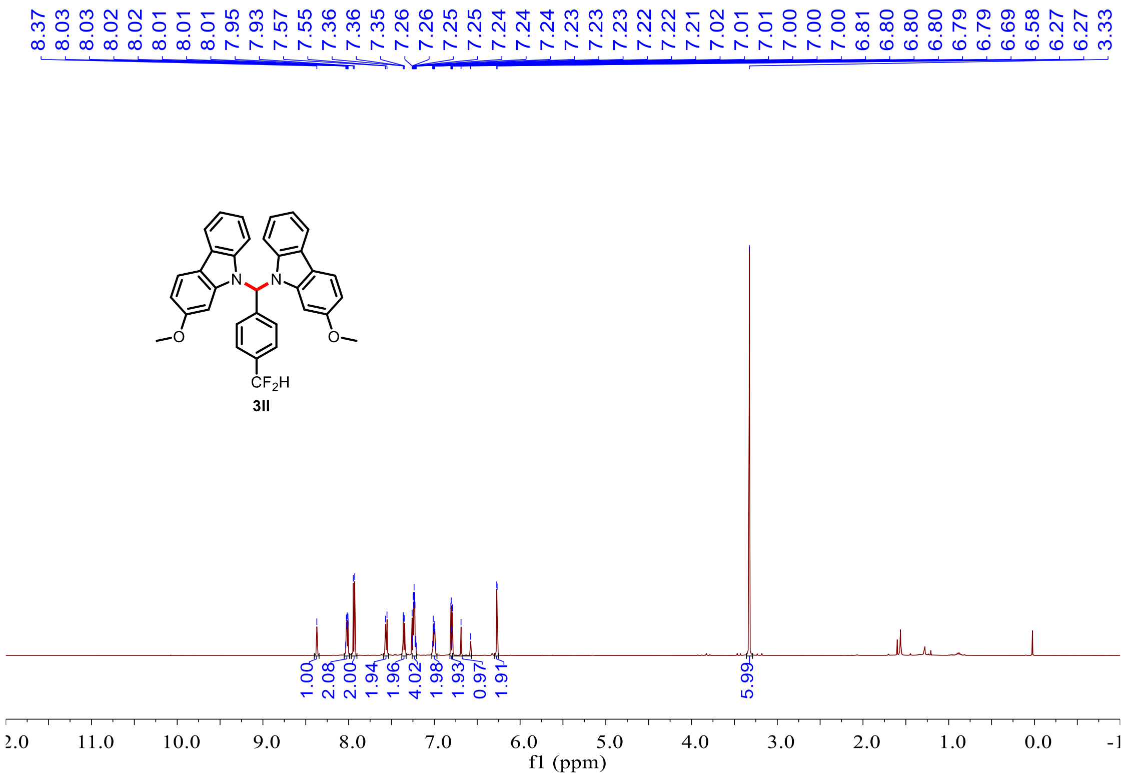

Supplementary Figure 152. ^{1}H NMR (500 MHz, $CDCl_3$) spectrum for compound **3ll**

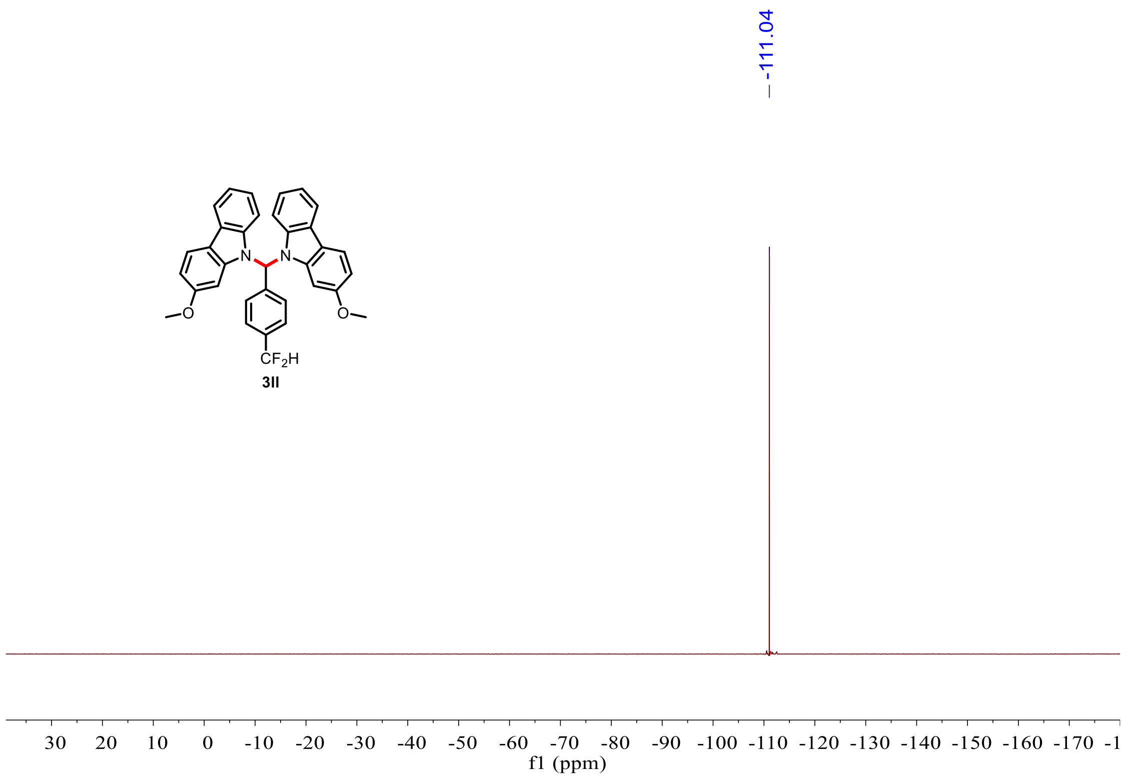

Supplementary Figure 153. ^{19}F NMR (471 MHz, $CDCl_3$) spectrum for compound **3ll**

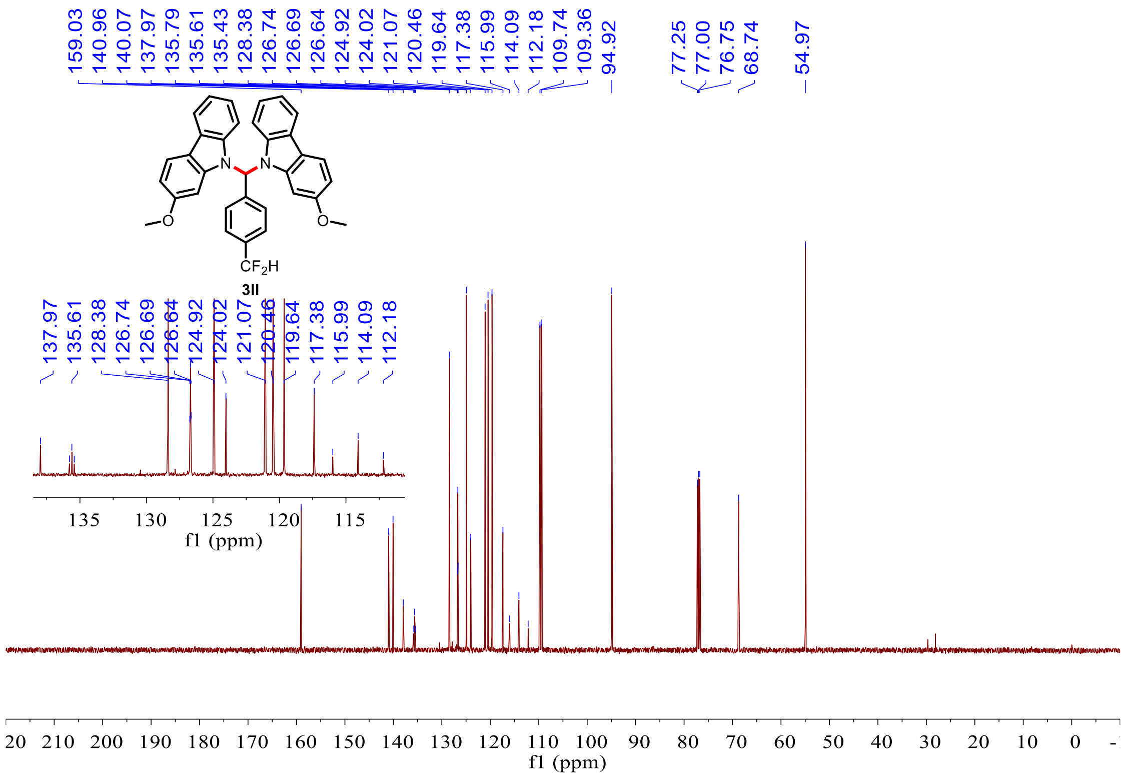

Supplementary Figure 154. ^{13}C NMR (126 MHz, $CDCl_3$) spectrum for compound **3ll**

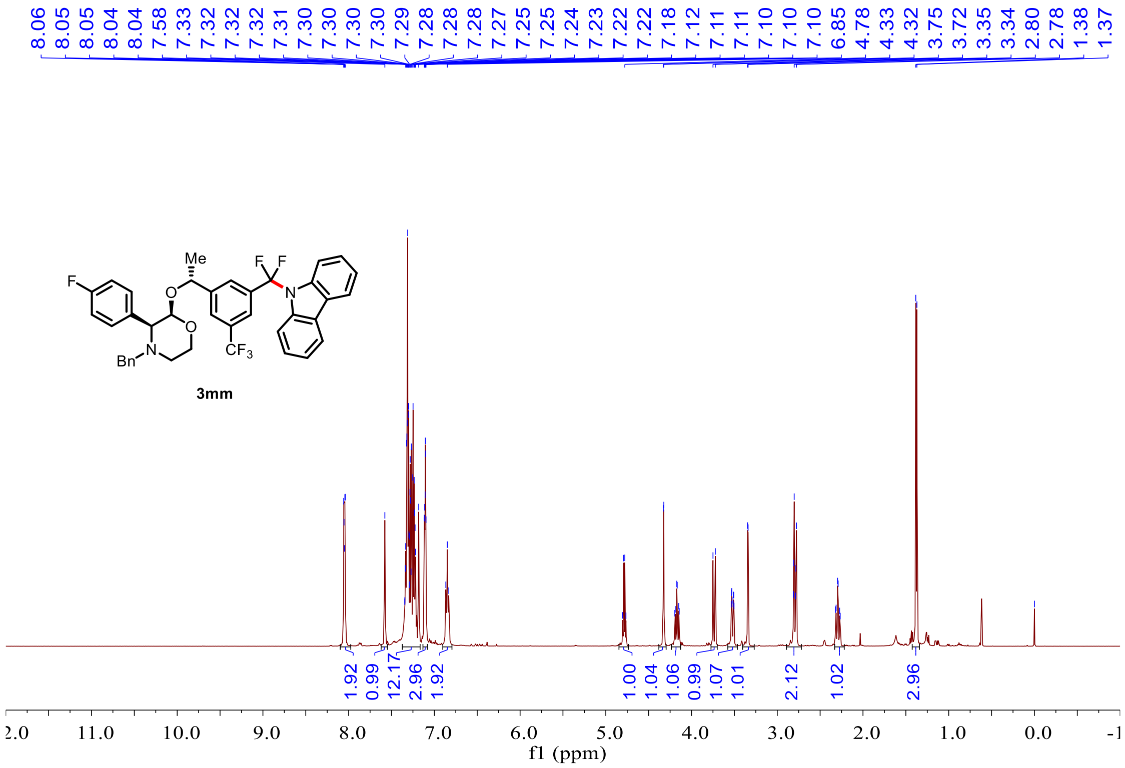

Supplementary Figure 155. ^{1}H NMR (500 MHz, $CDCl_3$) spectrum for compound **3mm**

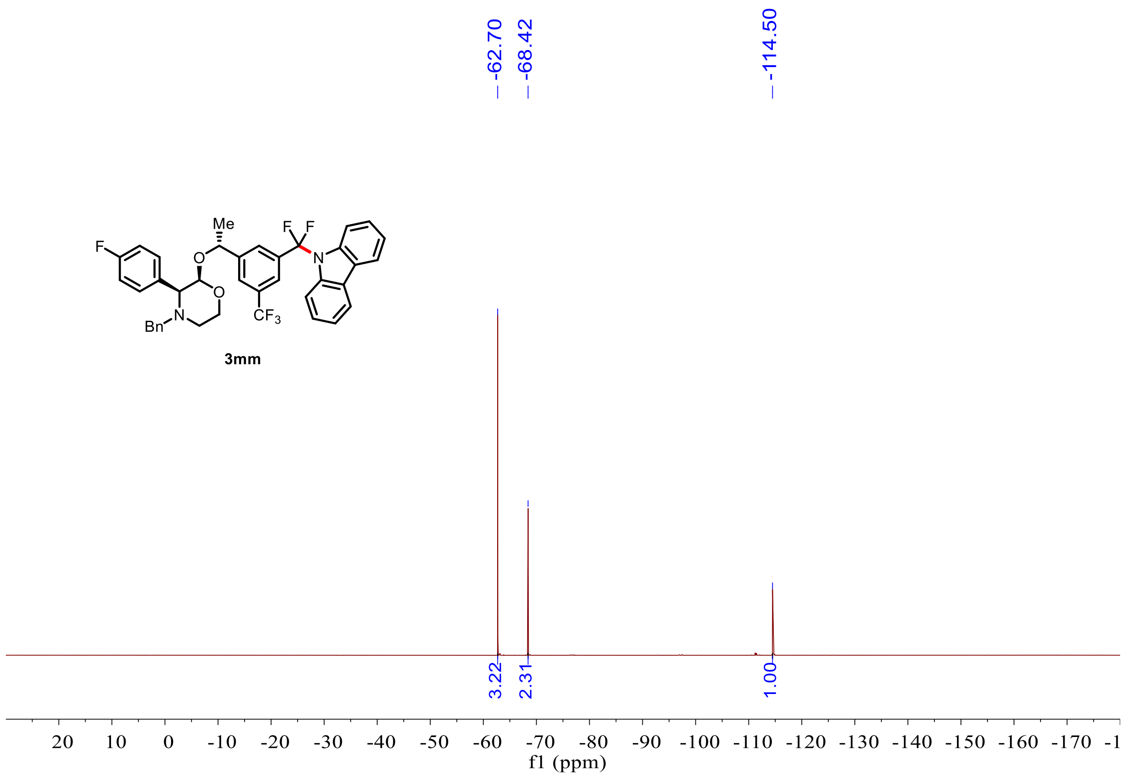

Supplementary Figure 156. **^{19}F NMR** (471 MHz, $CDCl_3$) spectrum for compound **3mm**

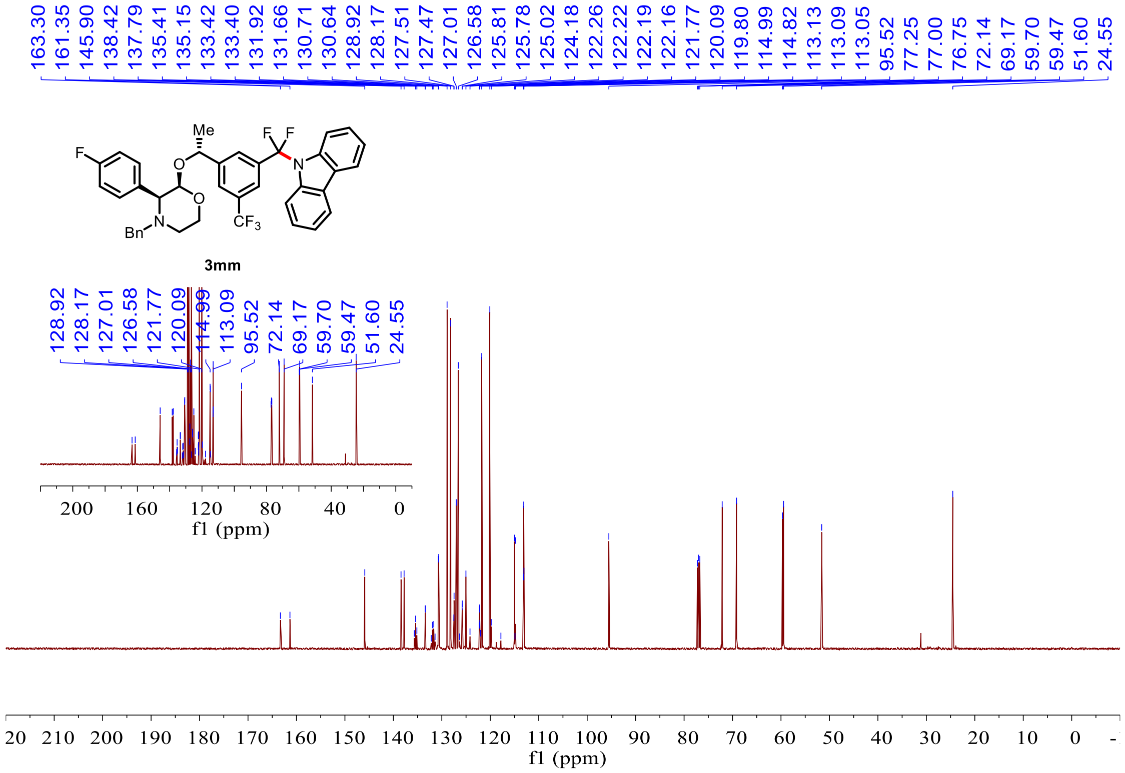

Supplementary Figure 157. **^{13}C NMR** (126 MHz, $CDCl_3$) spectrum for compound **3mm**

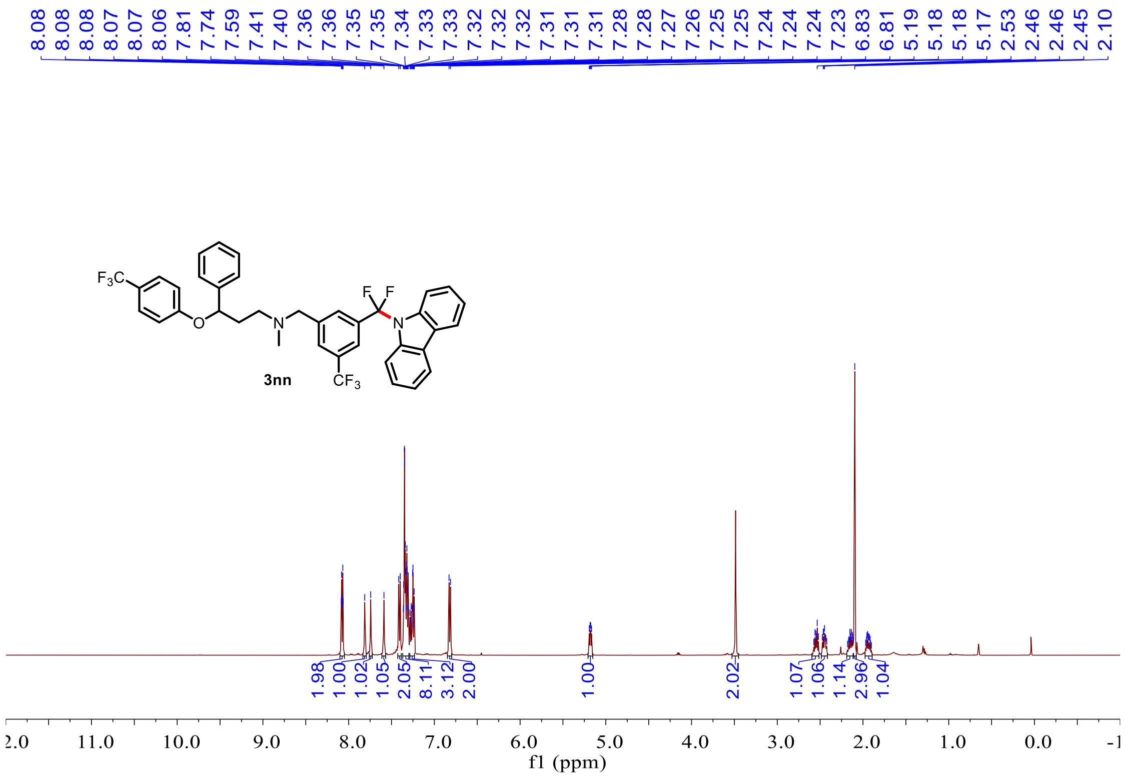

Supplementary Figure 158. ^{1}**H NMR** (500 MHz, $CDCl_3$) spectrum for compound **3nn**

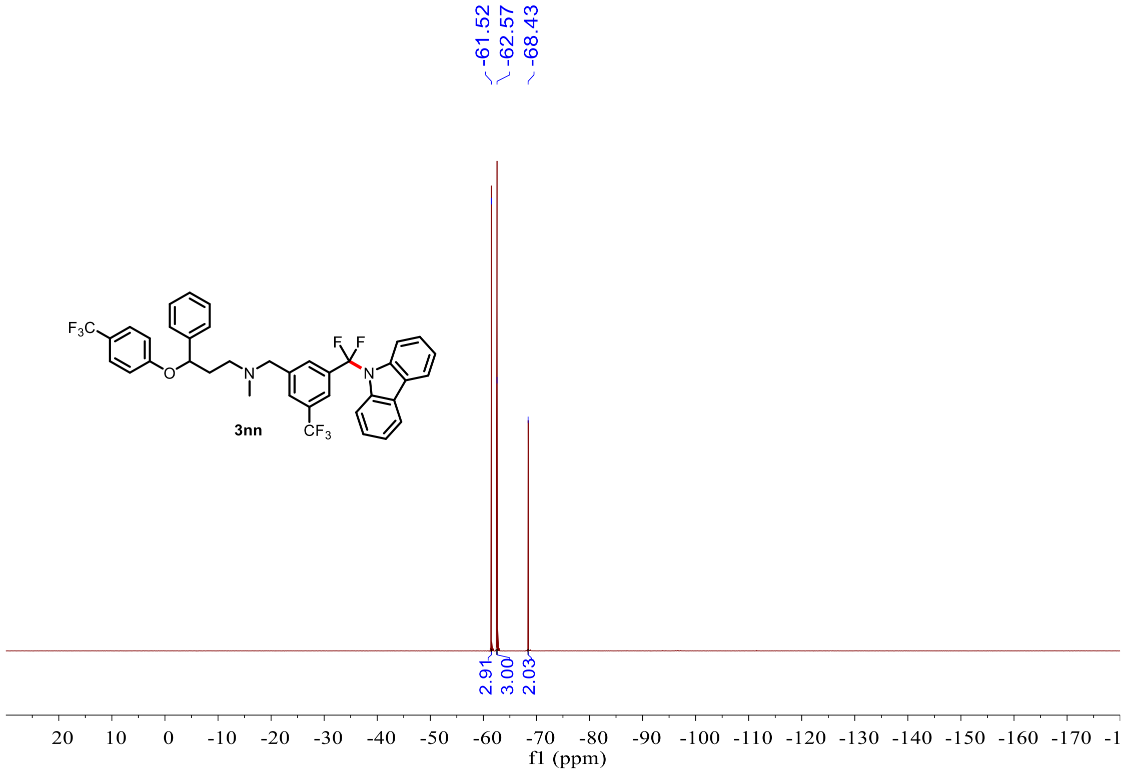

Supplementary Figure 159. ^{19}**F NMR** (471 MHz, $CDCl_3$) spectrum for compound **3nn**

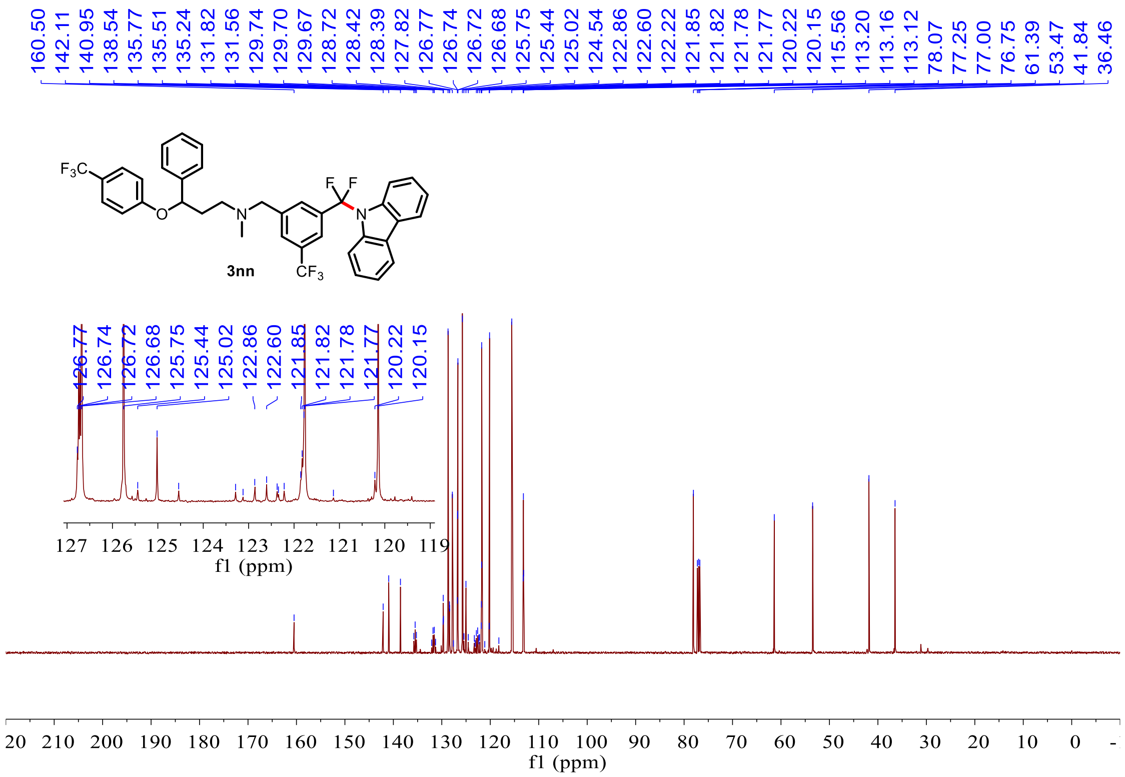

Supplementary Figure 160. ^{13}C NMR (126 MHz, $CDCl_3$) spectrum for compound **3nn**

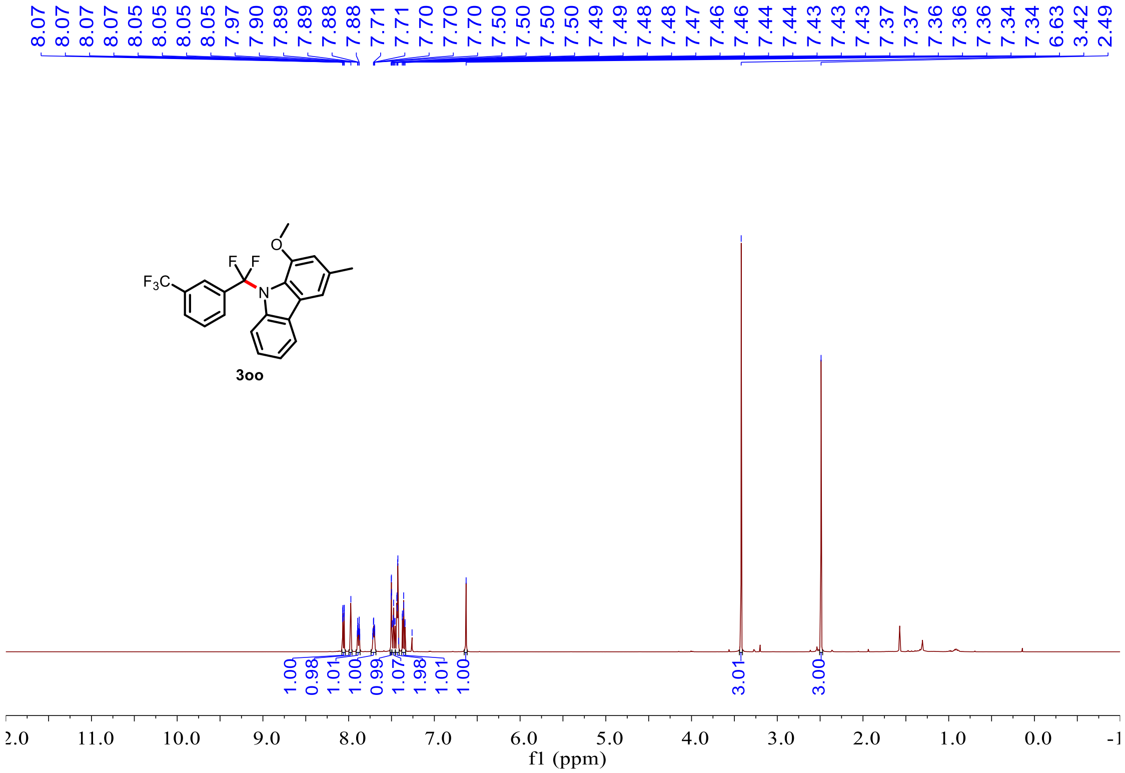

Supplementary Figure 161. ^{1}H NMR (500 MHz, $CDCl_3$) spectrum for compound **3oo**

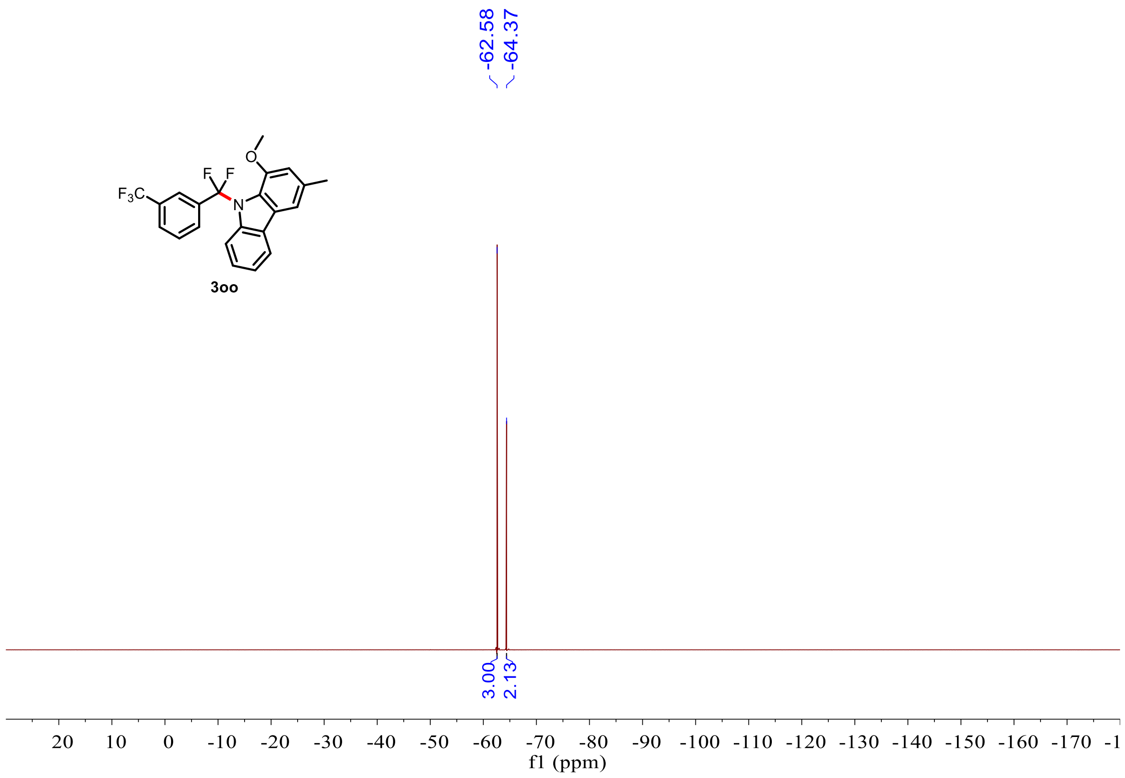

Supplementary Figure 162. ^{19}**F NMR** (471 MHz, $CDCl_3$) spectrum for compound **3oo**

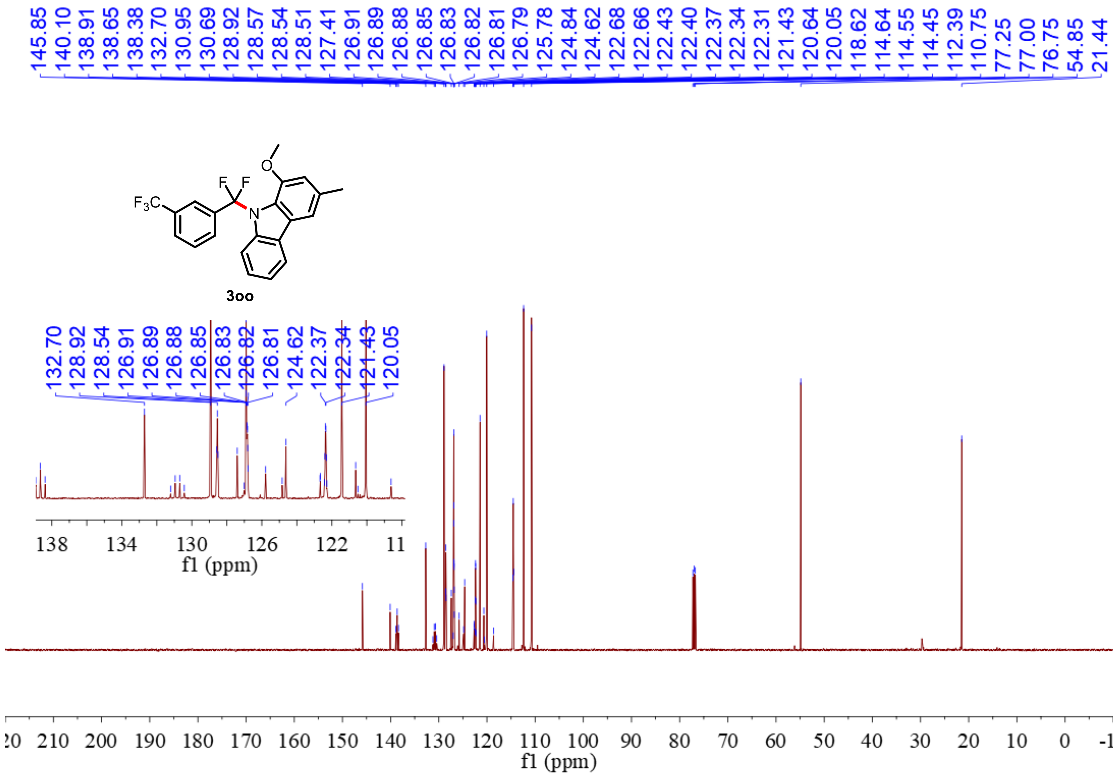

Supplementary Figure 163. ^{13}**C NMR** (126 MHz, $CDCl_3$) spectrum for compound **3oo**

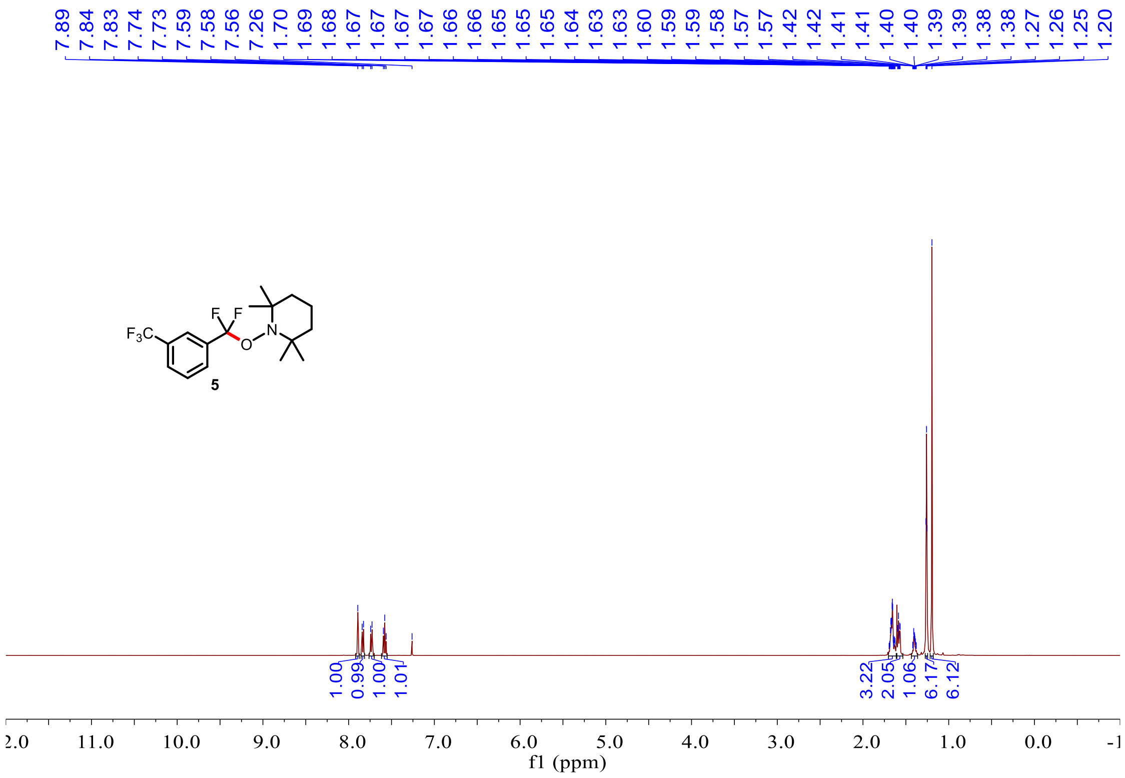

Supplementary Figure 164. **^{1}H NMR** (500 MHz, $CDCl_3$) spectrum for compound **5**

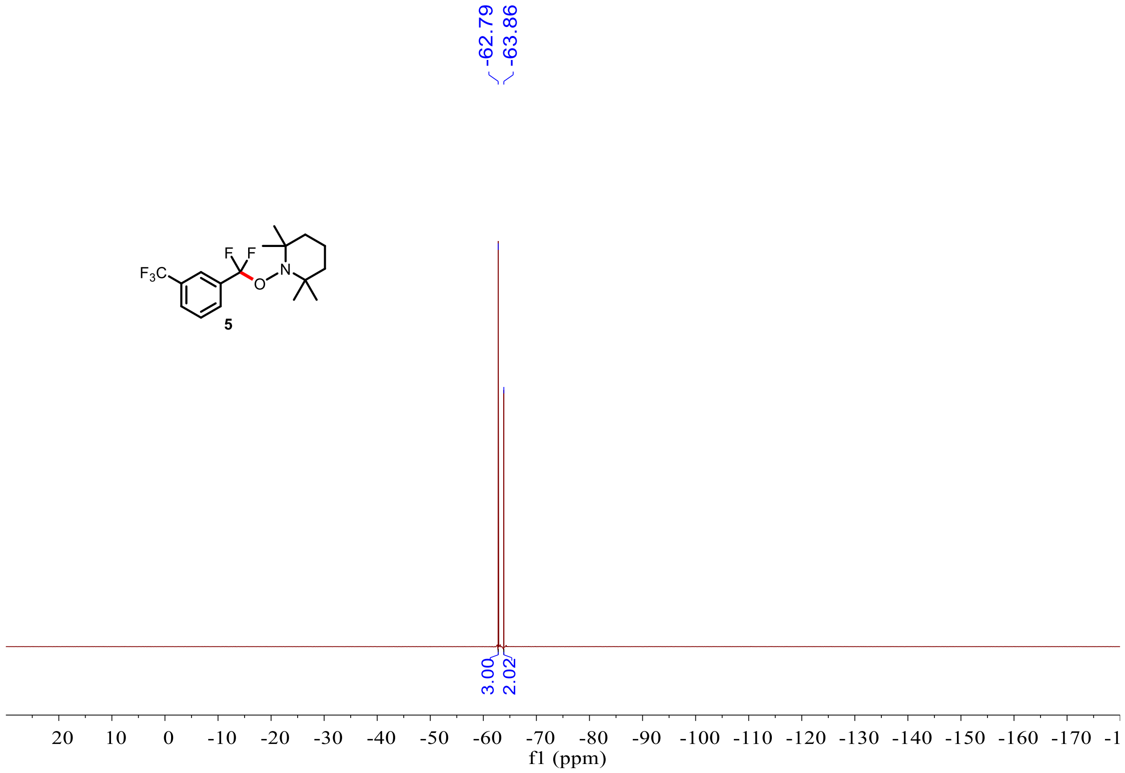

Supplementary Figure 165. **^{19}F NMR** (471 MHz, $CDCl_3$) spectrum for compound **5**

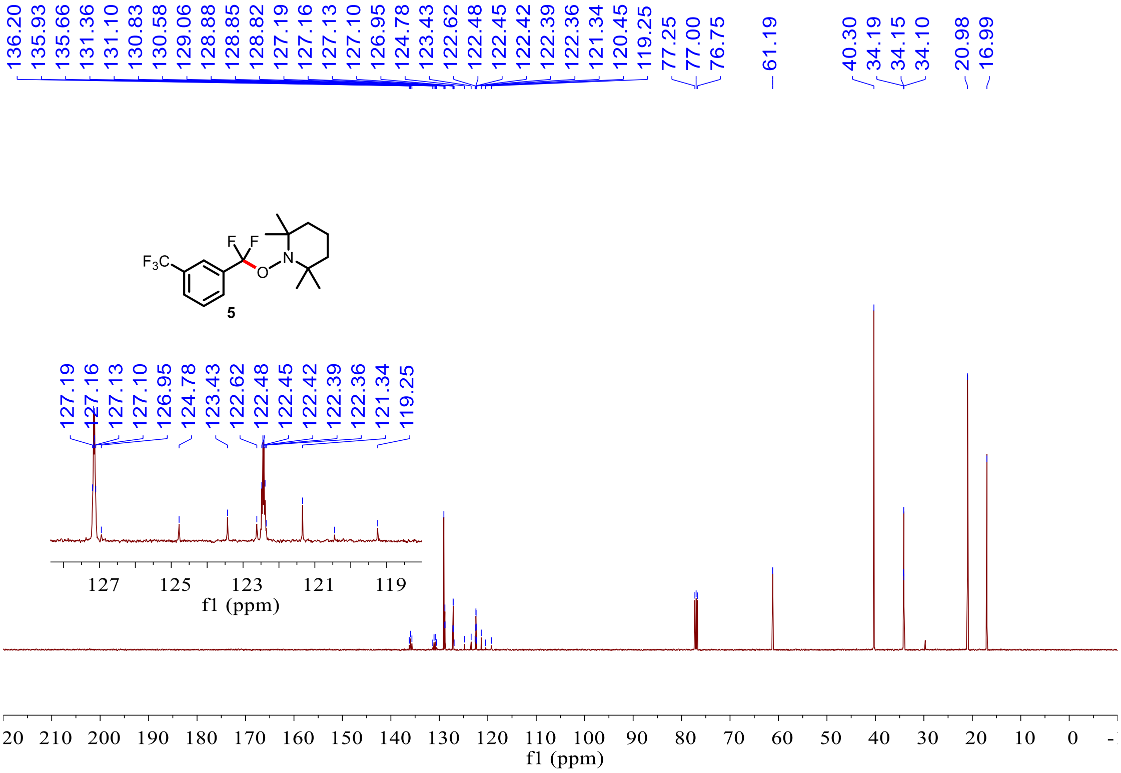

Supplementary Figure 166. ^{13}**C NMR** (126 MHz, $CDCl_3$) spectrum for compound **5**

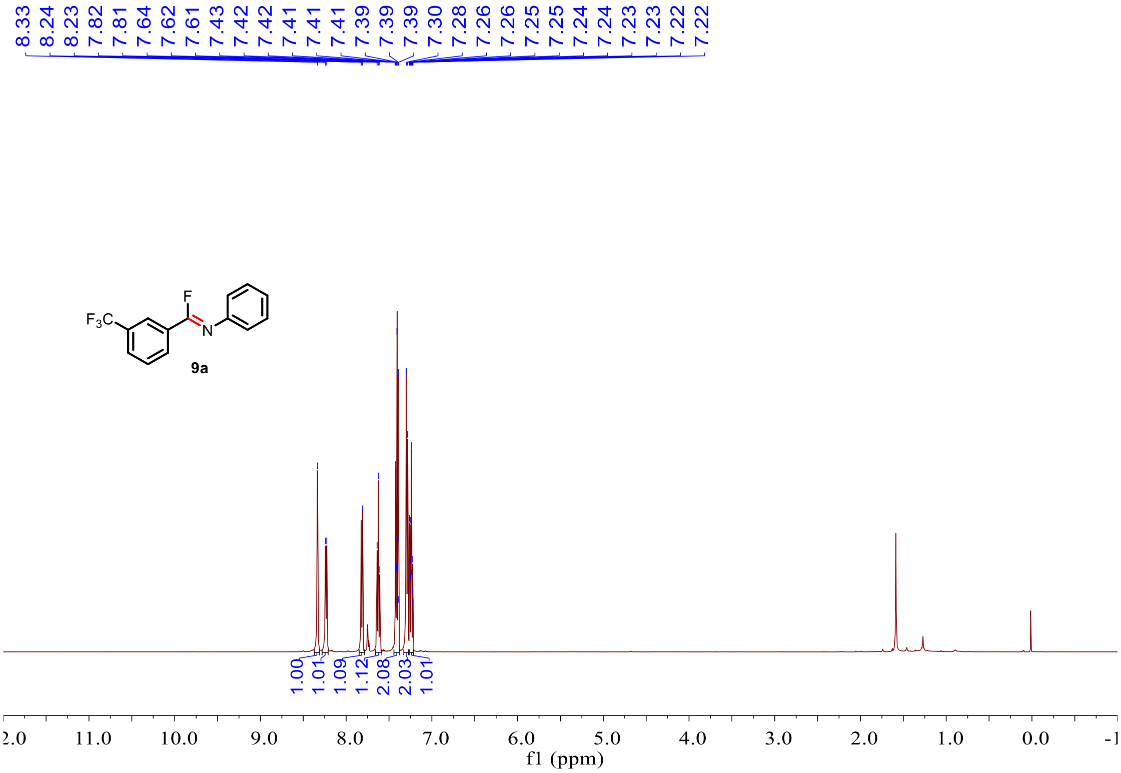

Supplementary Figure 167. ^{1}**H NMR** (500 MHz, $CDCl_3$) spectrum for compound **9a**

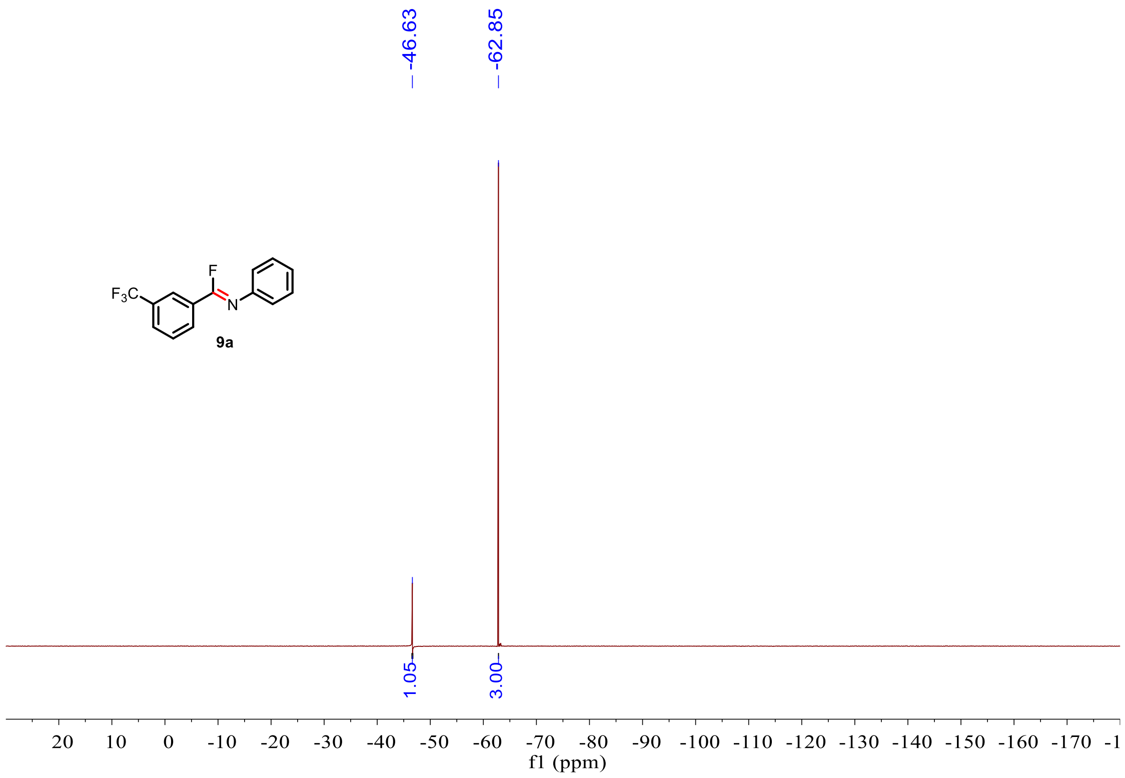

Supplementary Figure 168. ^{19}**F NMR** (471 MHz, $CDCl_3$) spectrum for compound **9a**

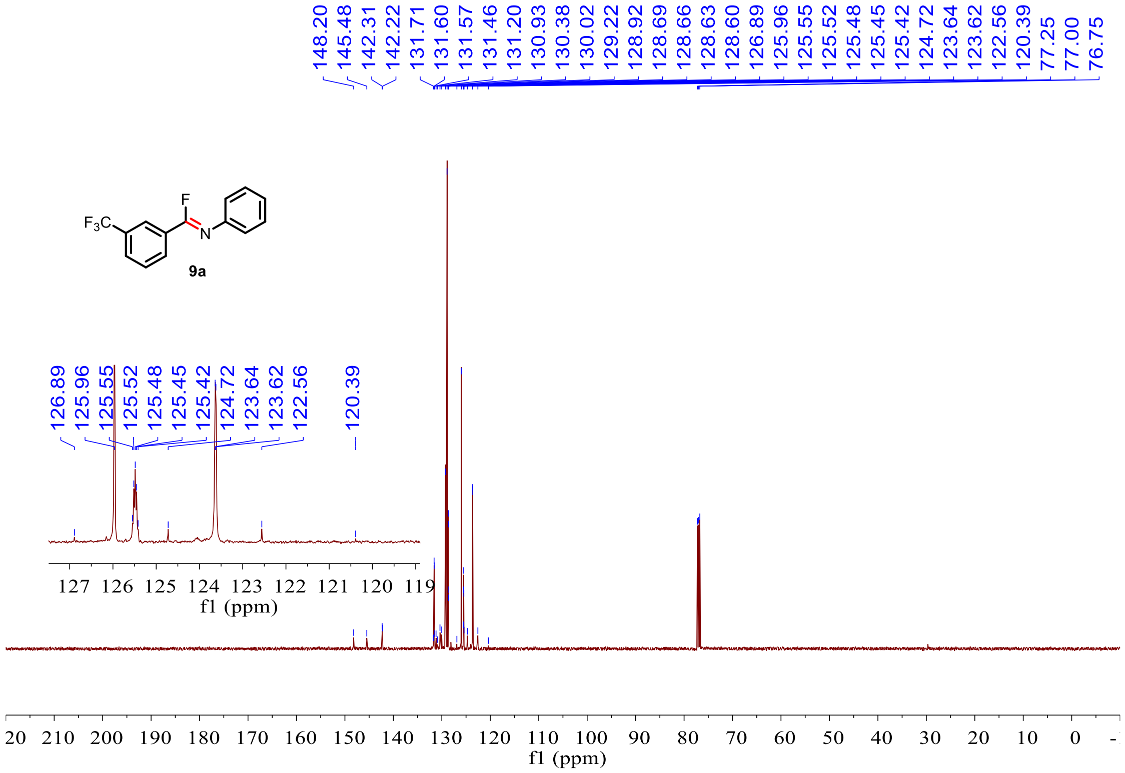

Supplementary Figure 169. ^{13}**C NMR** (126 MHz, $CDCl_3$) spectrum for compound **9a**

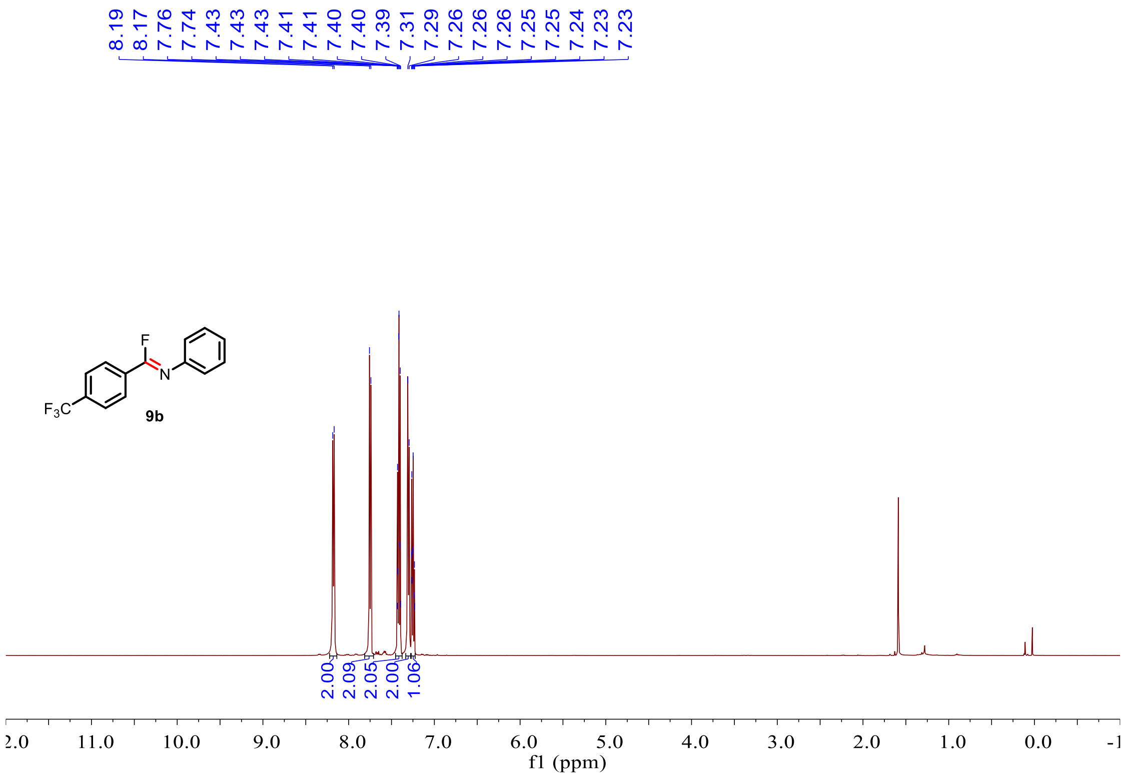

Supplementary Figure 170. ^{1}**H NMR** (500 MHz, $CDCl_3$) spectrum for compound **9b**

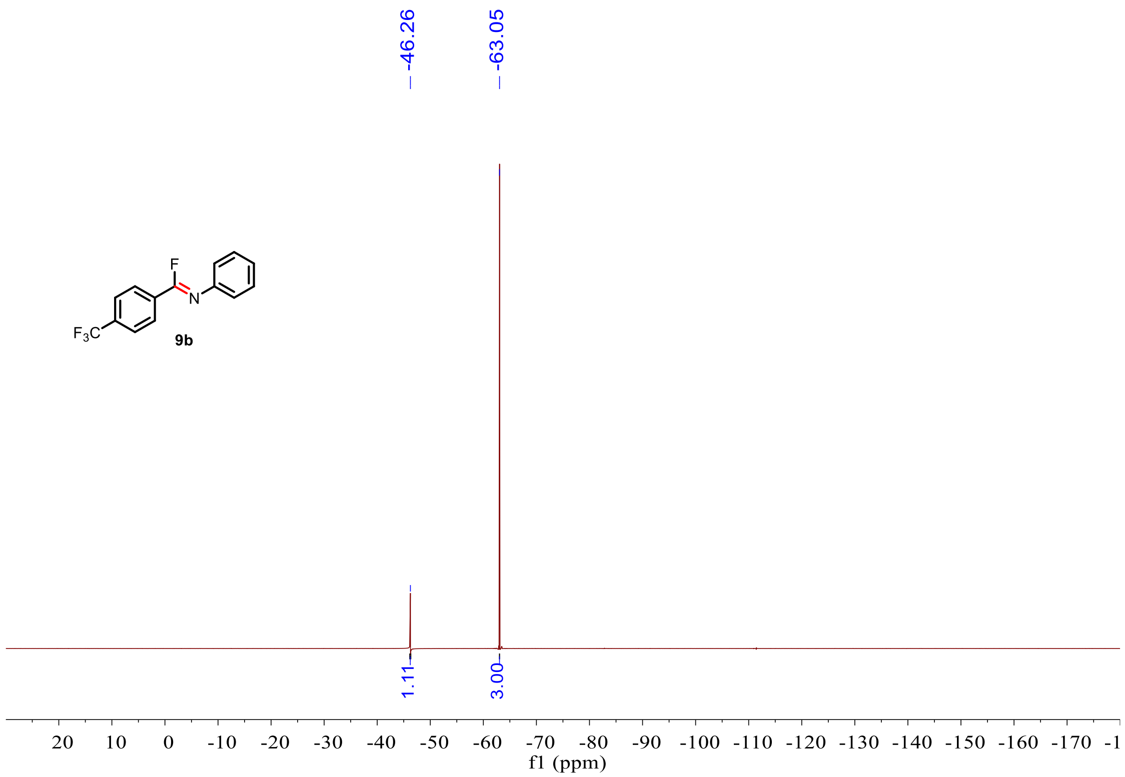

Supplementary Figure 171. ^{19}**F NMR** (471 MHz, $CDCl_3$) spectrum for compound **9b**

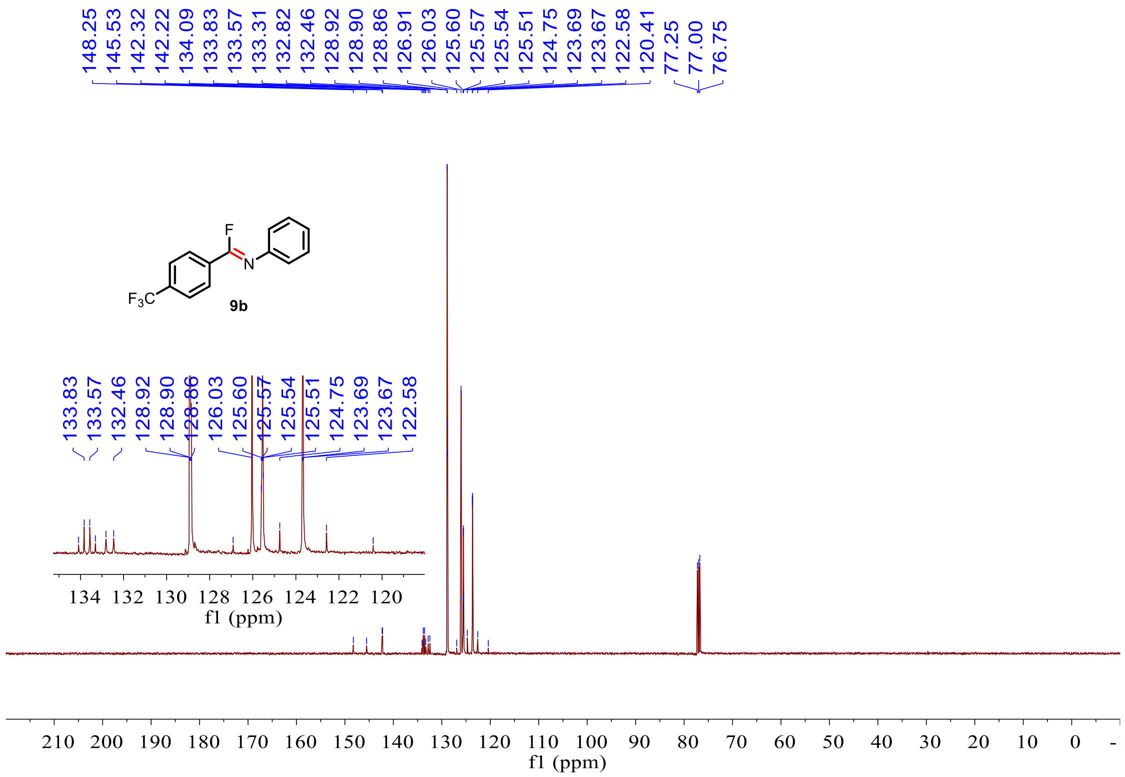

Supplementary Figure 172. ^{13}C NMR (126 MHz, $CDCl_3$) spectrum for compound **9b**

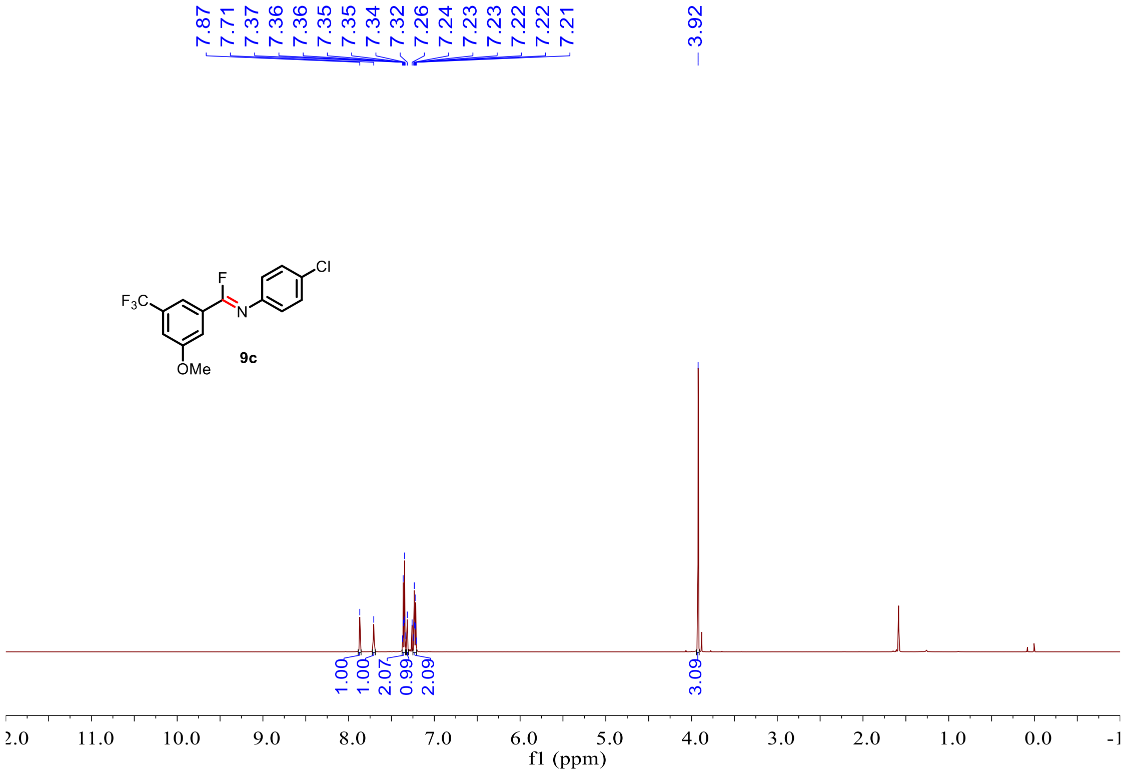

Supplementary Figure 173. ^{1}H NMR (500 MHz, $CDCl_3$) spectrum for compound **9c**

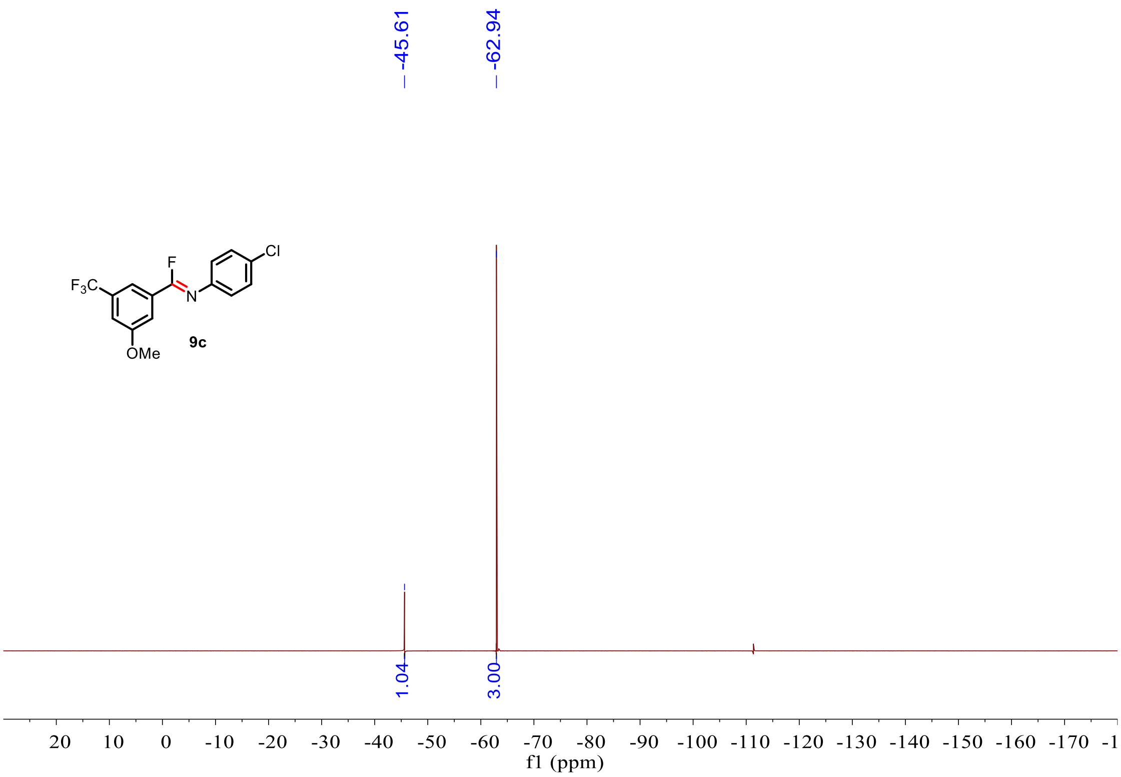

Supplementary Figure 174. ^{19}**F NMR** (471 MHz, $CDCl_3$) spectrum for compound **9c**

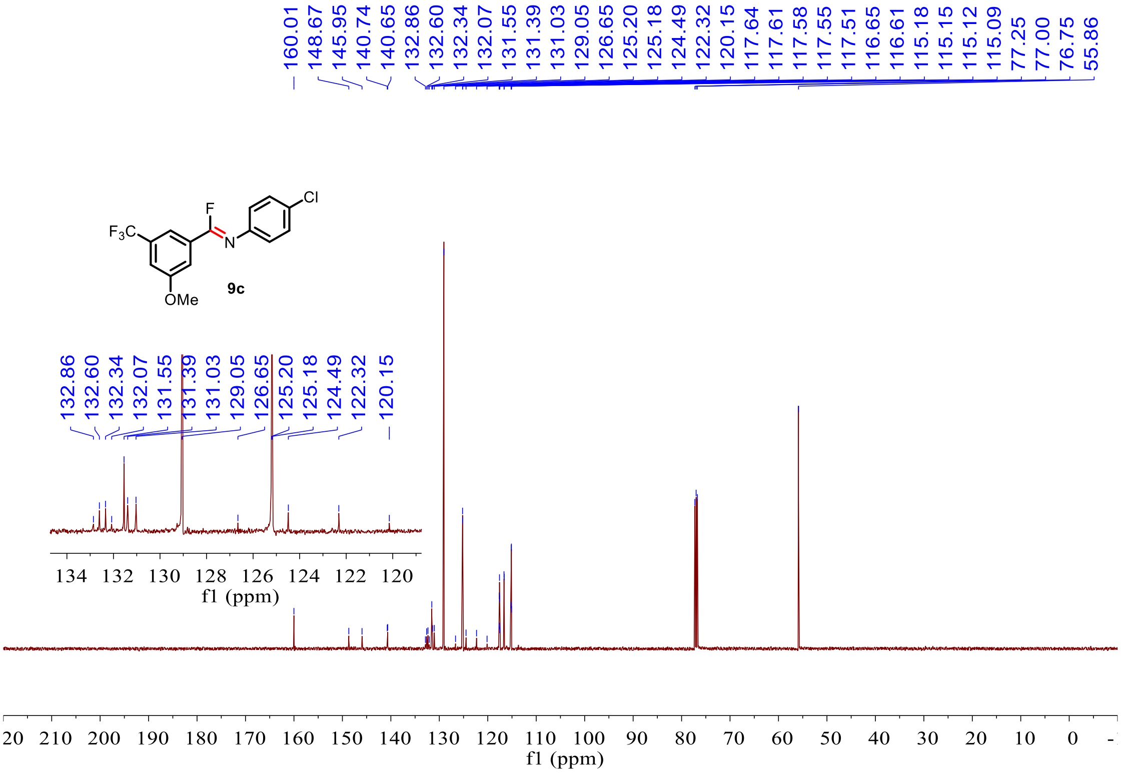

Supplementary Figure 175. ^{13}**C NMR** (126 MHz, $CDCl_3$) spectrum for compound **9c**

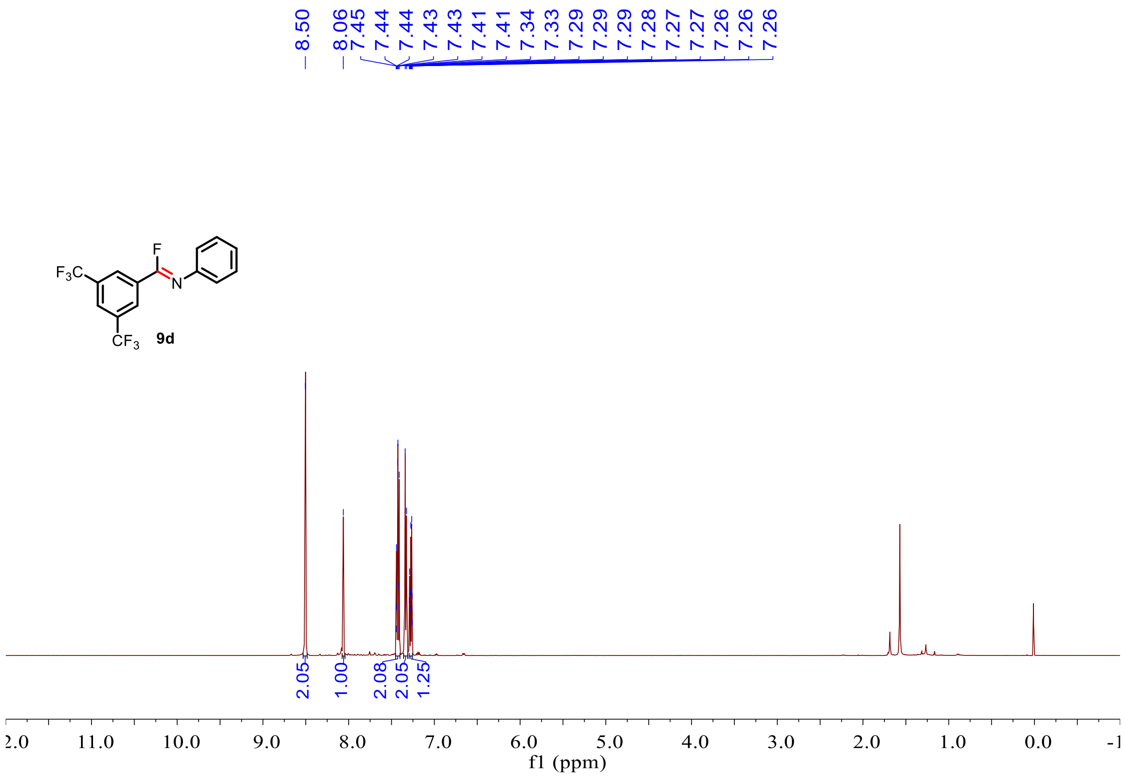

Supplementary Figure 176. ^{1}H NMR (500 MHz, $CDCl_3$) spectrum for compound **9d**

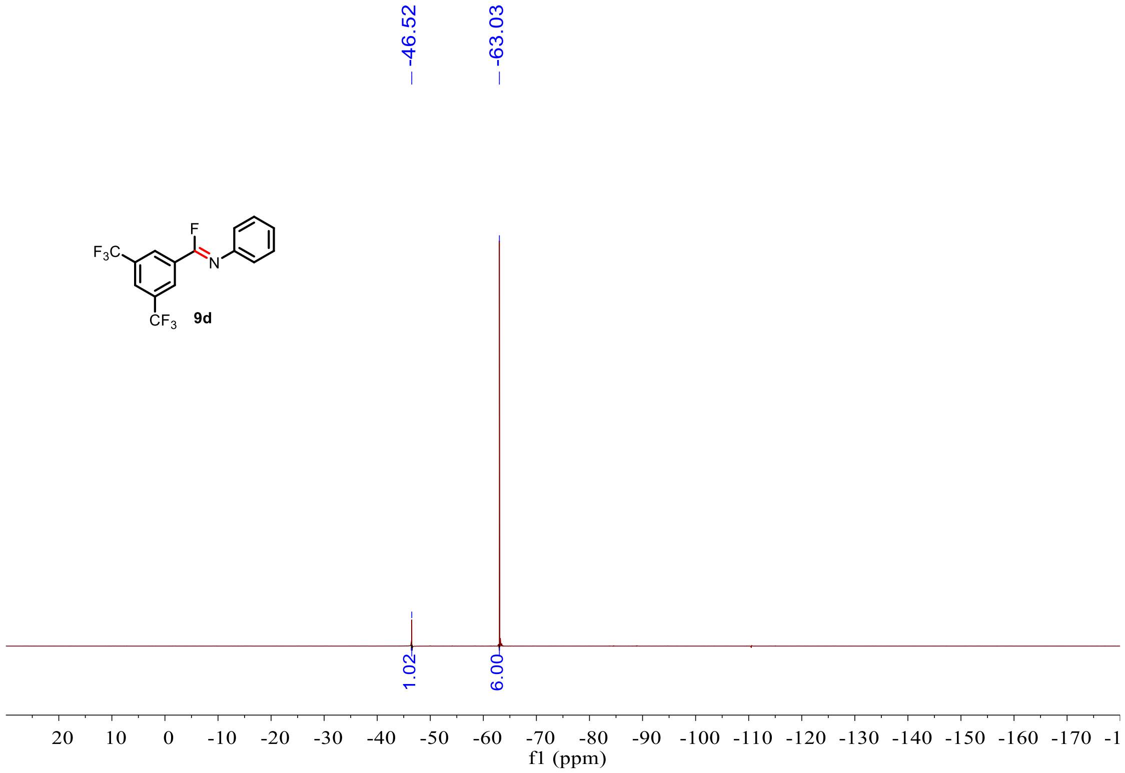

Supplementary Figure 177. ^{19}F NMR (471 MHz, $CDCl_3$) spectrum for compound **9d**

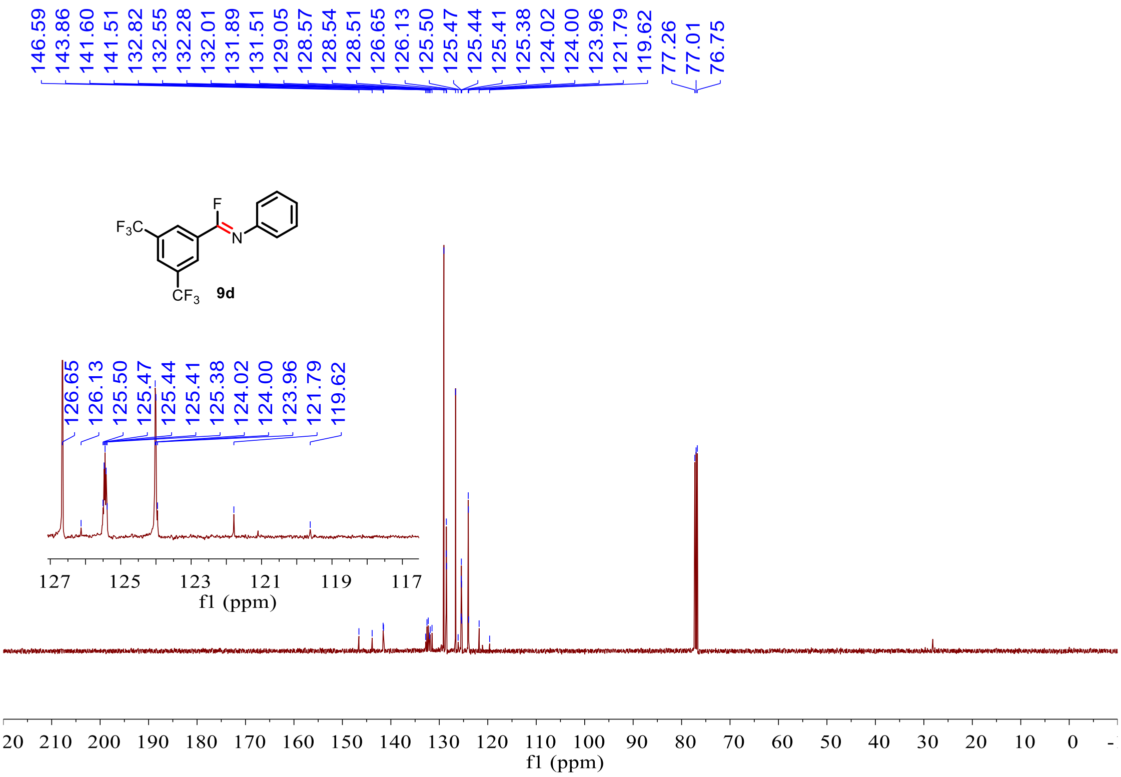

Supplementary Figure 178. ^{13}C NMR (126 MHz, $CDCl_3$) spectrum for compound **9d**

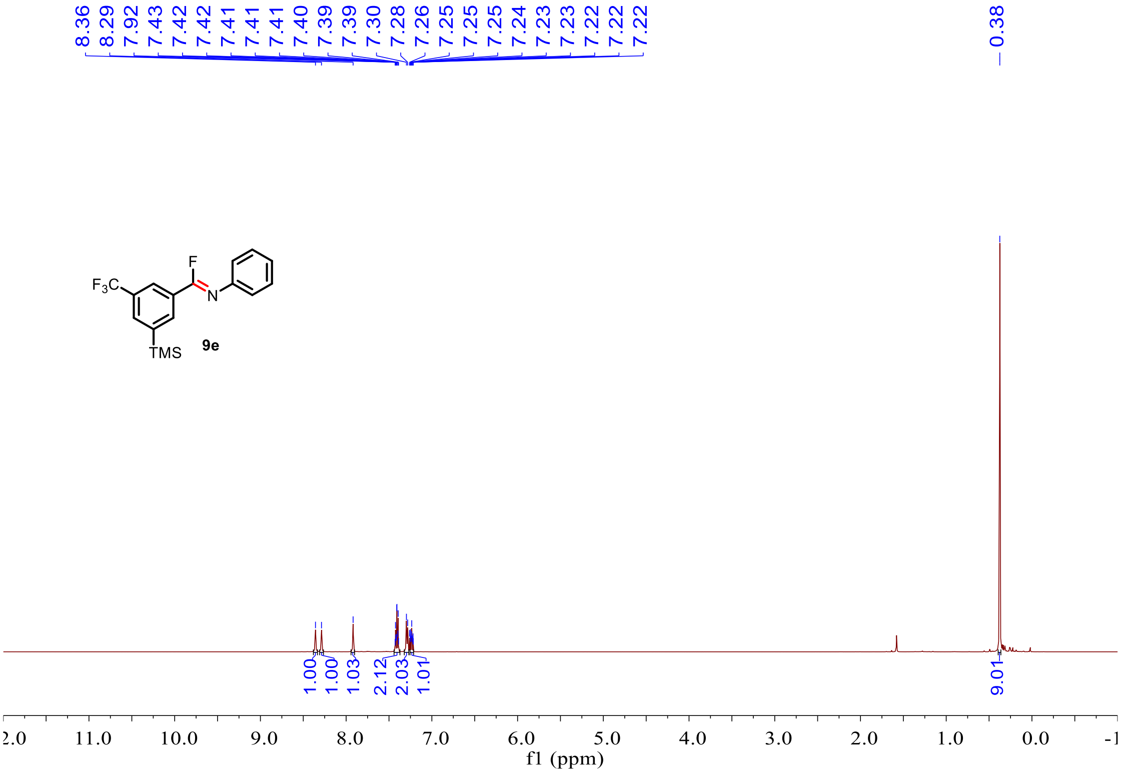

Supplementary Figure 179. ^{1}H NMR (500 MHz, $CDCl_3$) spectrum for compound **9e**

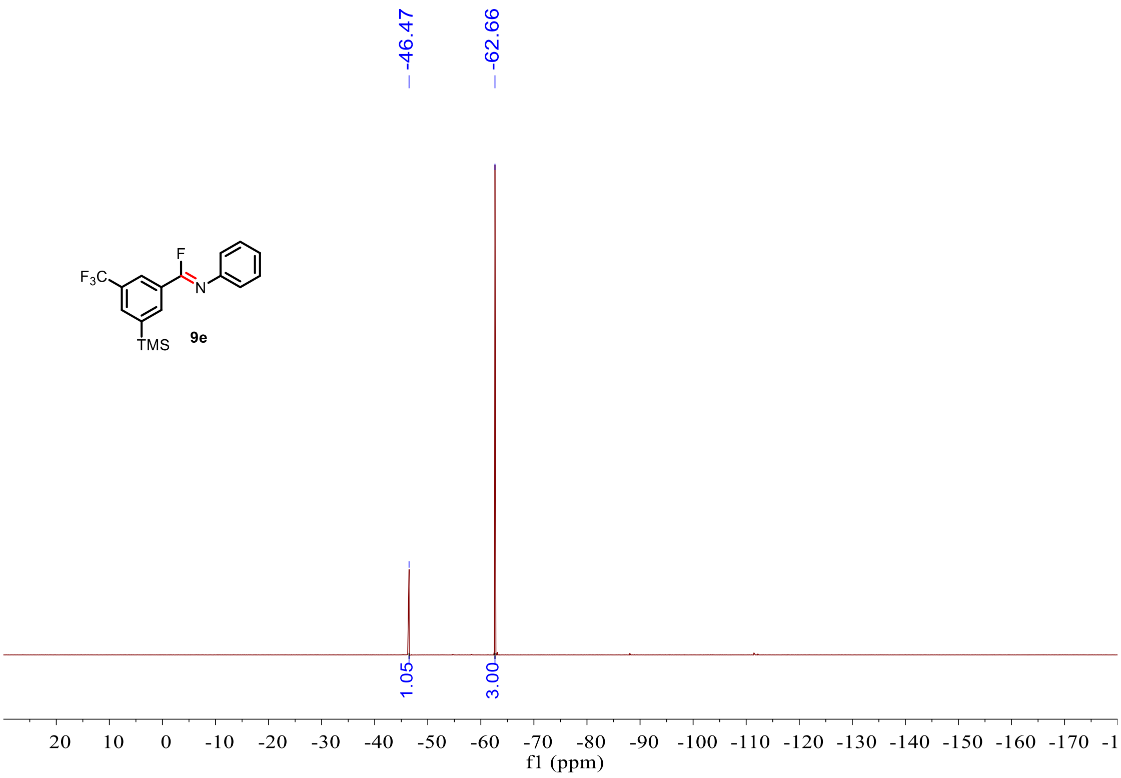

Supplementary Figure 180. ^{19}**F NMR** (471 MHz, $CDCl_3$) spectrum for compound **9e**

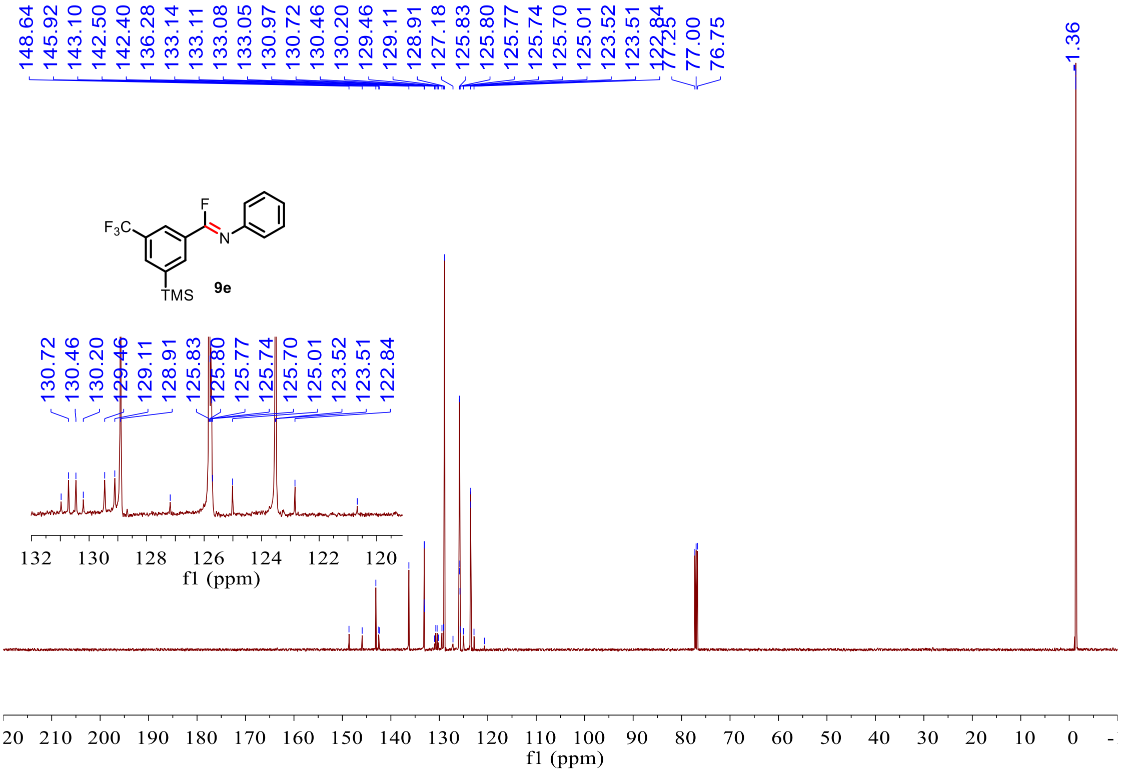

Supplementary Figure 181. ^{13}**C NMR** (126 MHz, $CDCl_3$) spectrum for compound **9e**

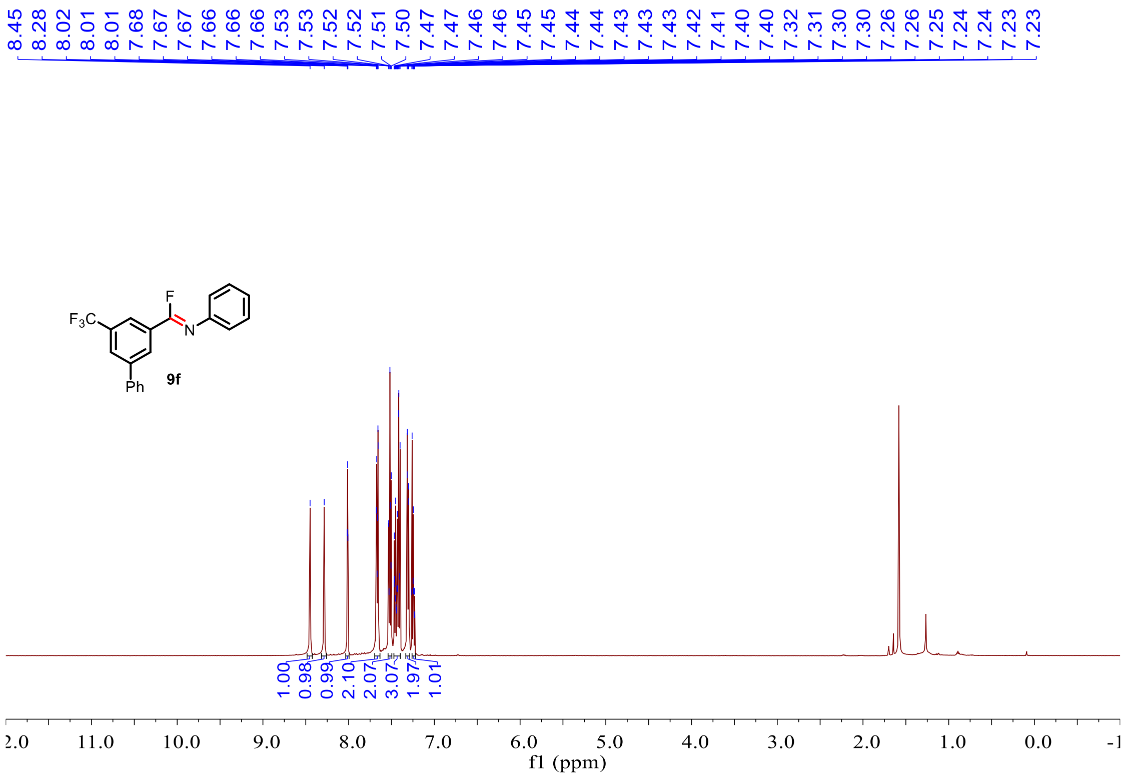

Supplementary Figure 182. ^{1}**H NMR** (500 MHz, $CDCl_3$) spectrum for compound **9f**

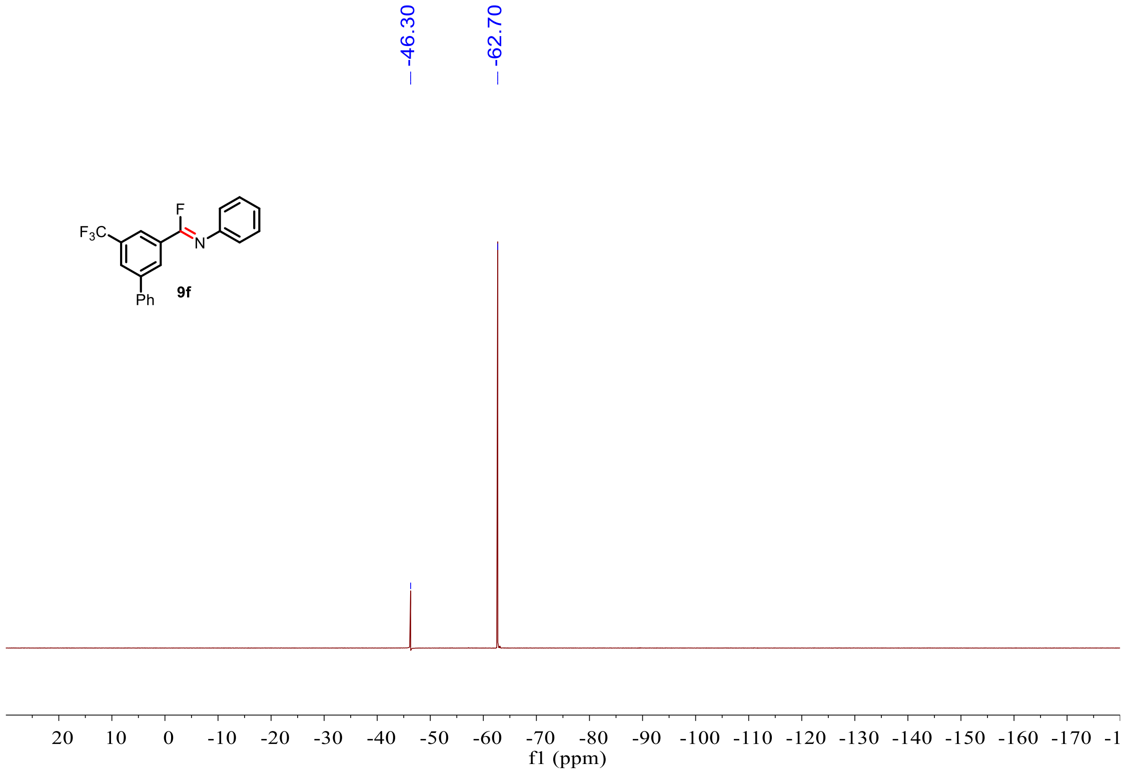

Supplementary Figure 183. ^{19}**F NMR** (471 MHz, $CDCl_3$) spectrum for compound **9f**

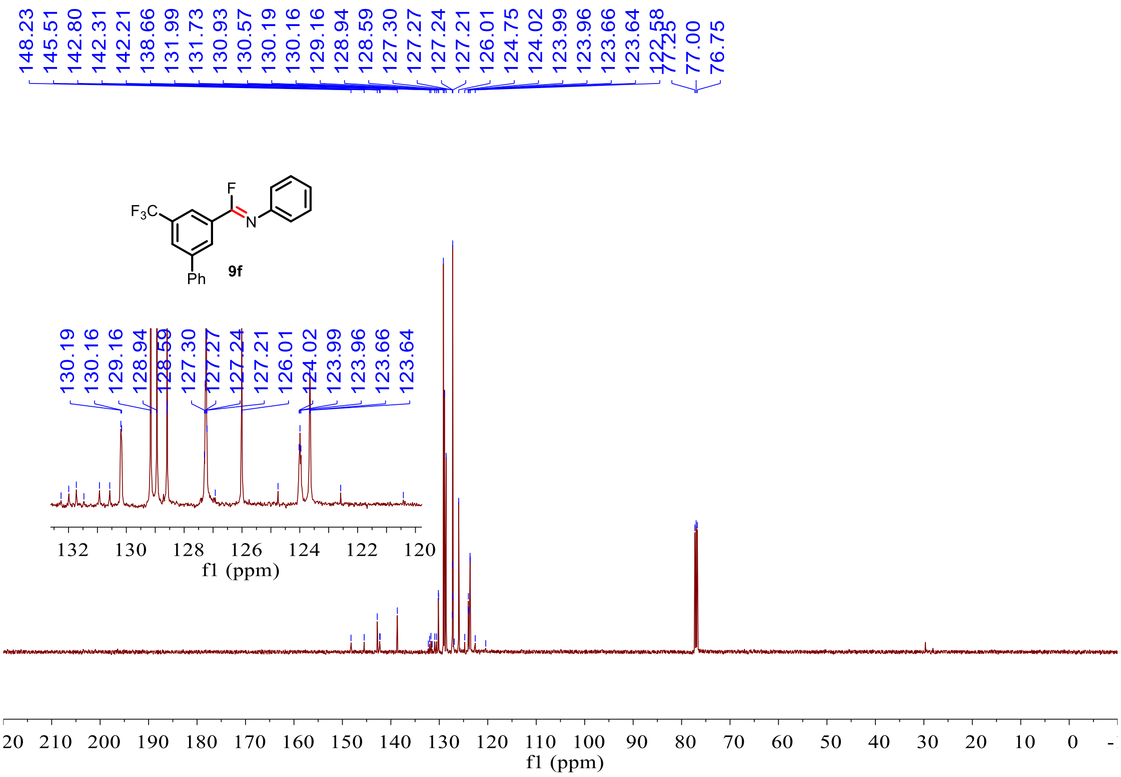

Supplementary Figure 184. ^{13}C NMR (126 MHz, CDCl$_3$) spectrum for compound **9f**

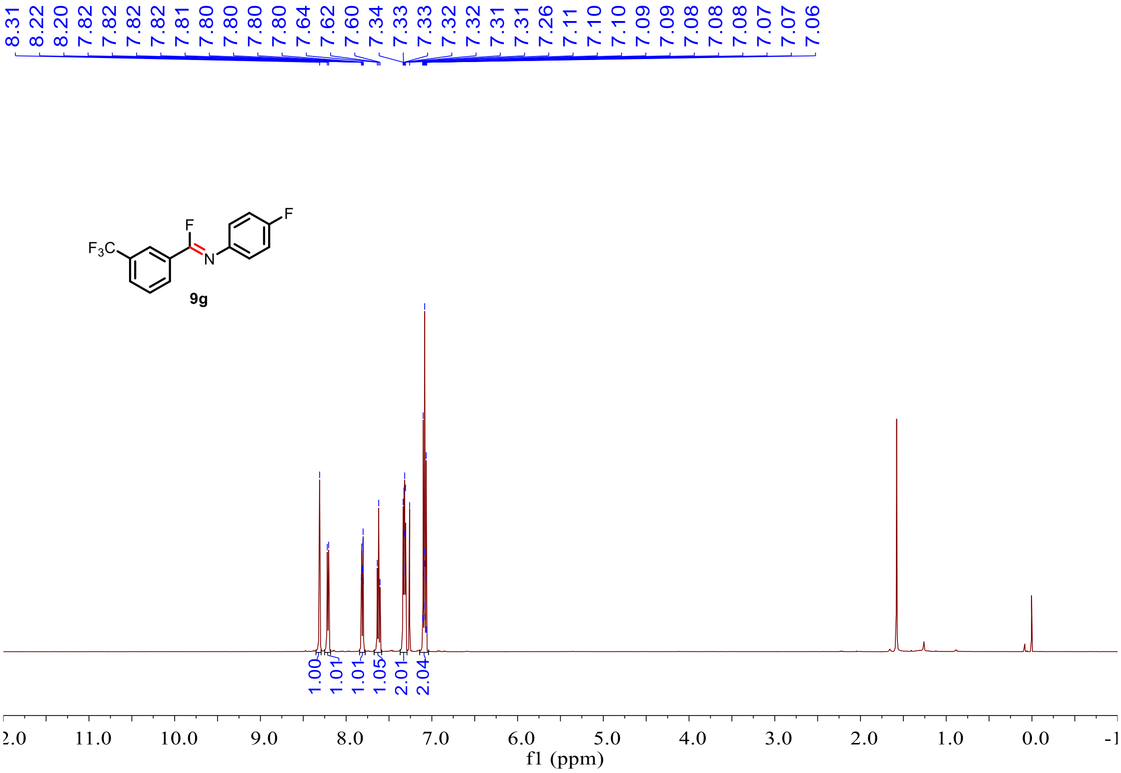

Supplementary Figure 185. ^{1}H NMR (500 MHz, CDCl$_3$) spectrum for compound **9g**

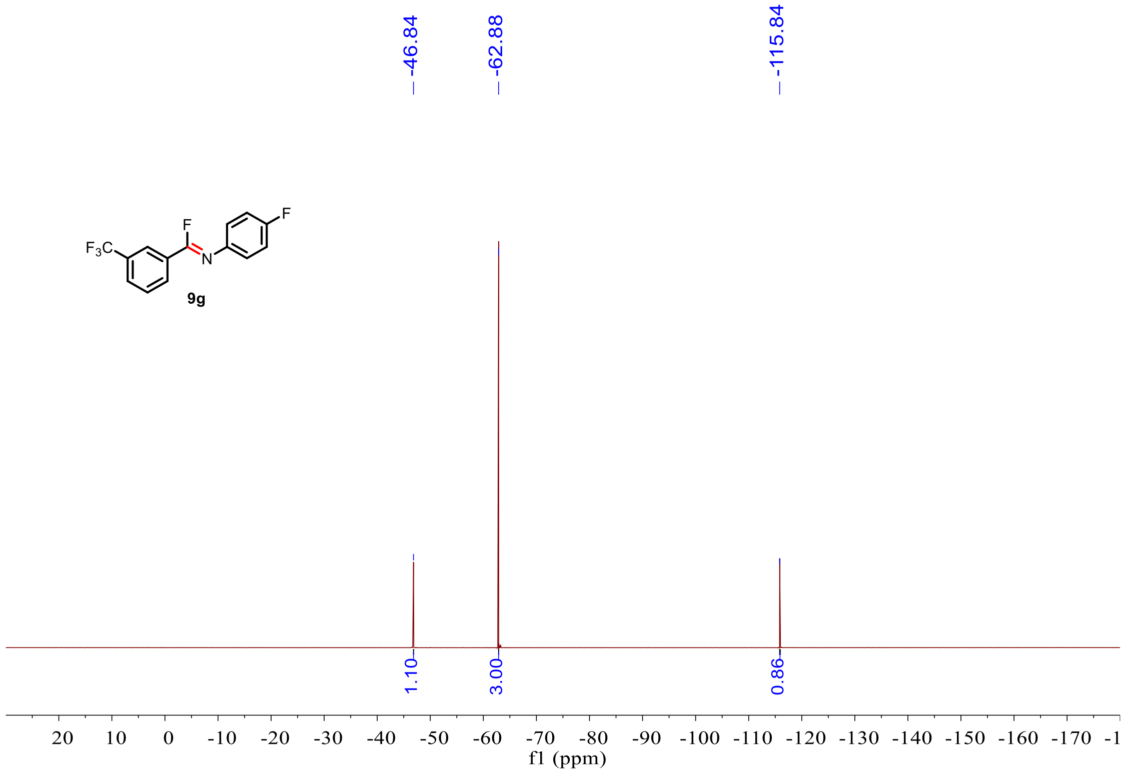

Supplementary Figure 186. ^{19}**F NMR** (471 MHz, $CDCl_3$) spectrum for compound **9g**

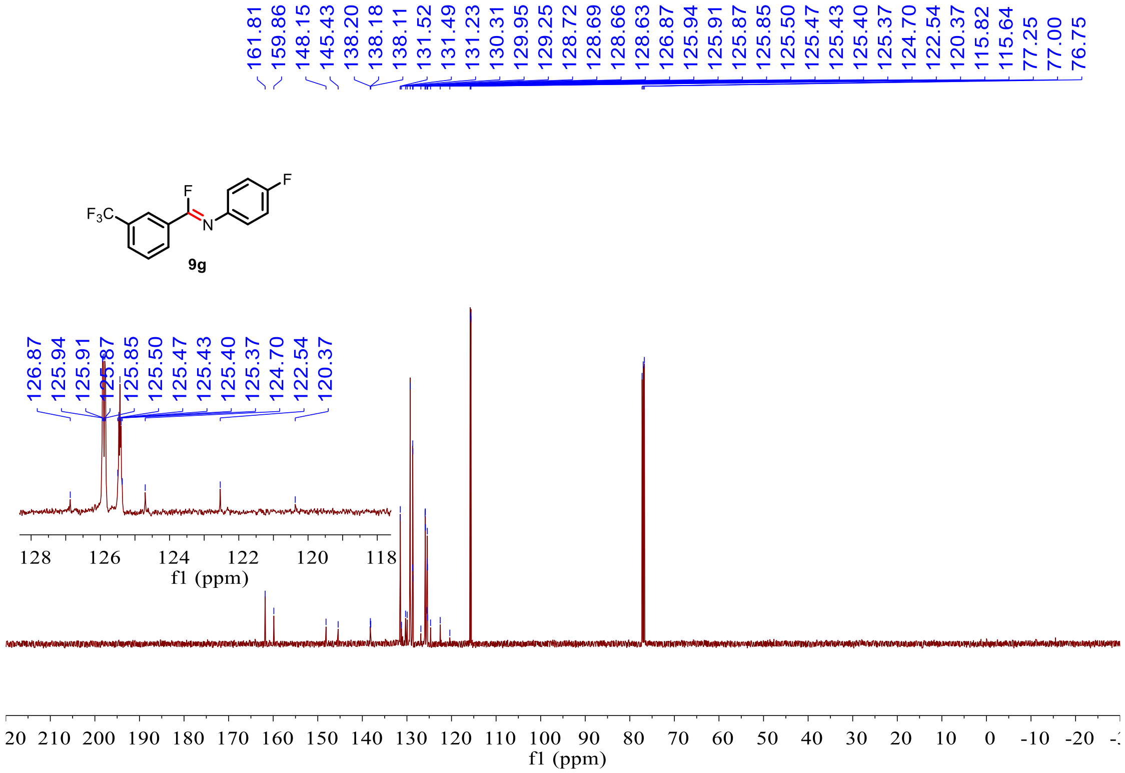

Supplementary Figure 187. ^{13}**C NMR** (126 MHz, $CDCl_3$) spectrum for compound **9g**

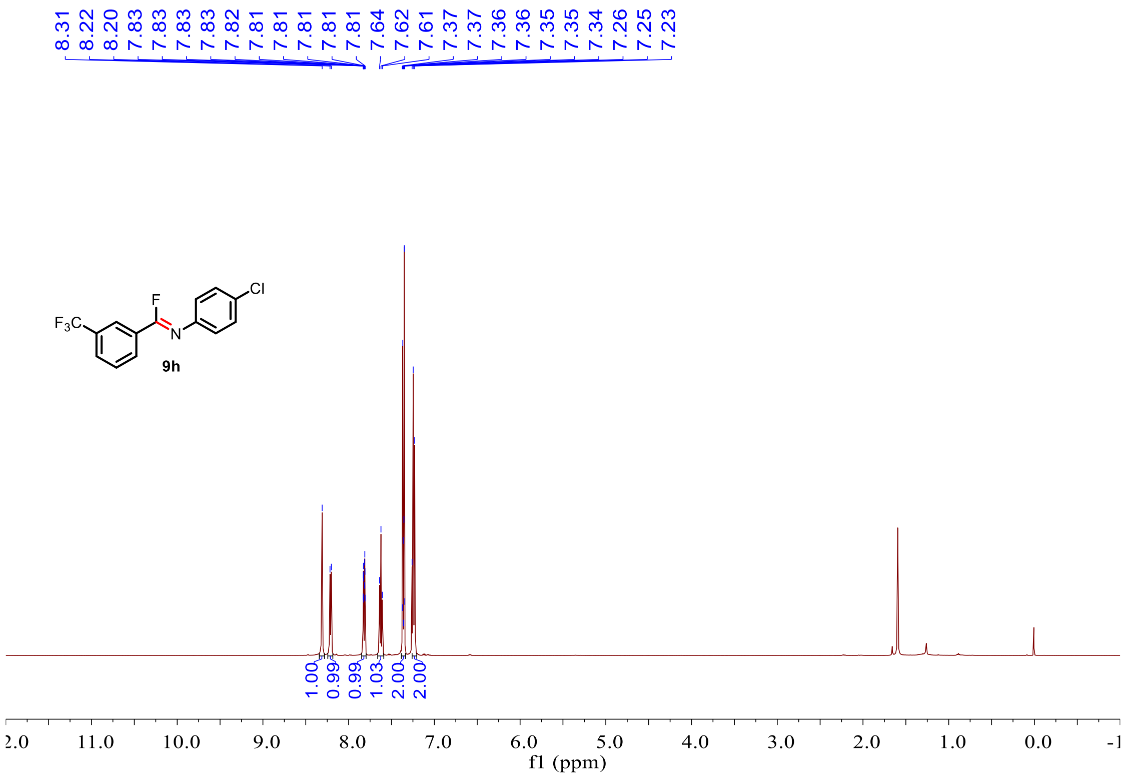

Supplementary Figure 188. ^{1}**H NMR** (500 MHz, $CDCl_3$) spectrum for compound **9h**

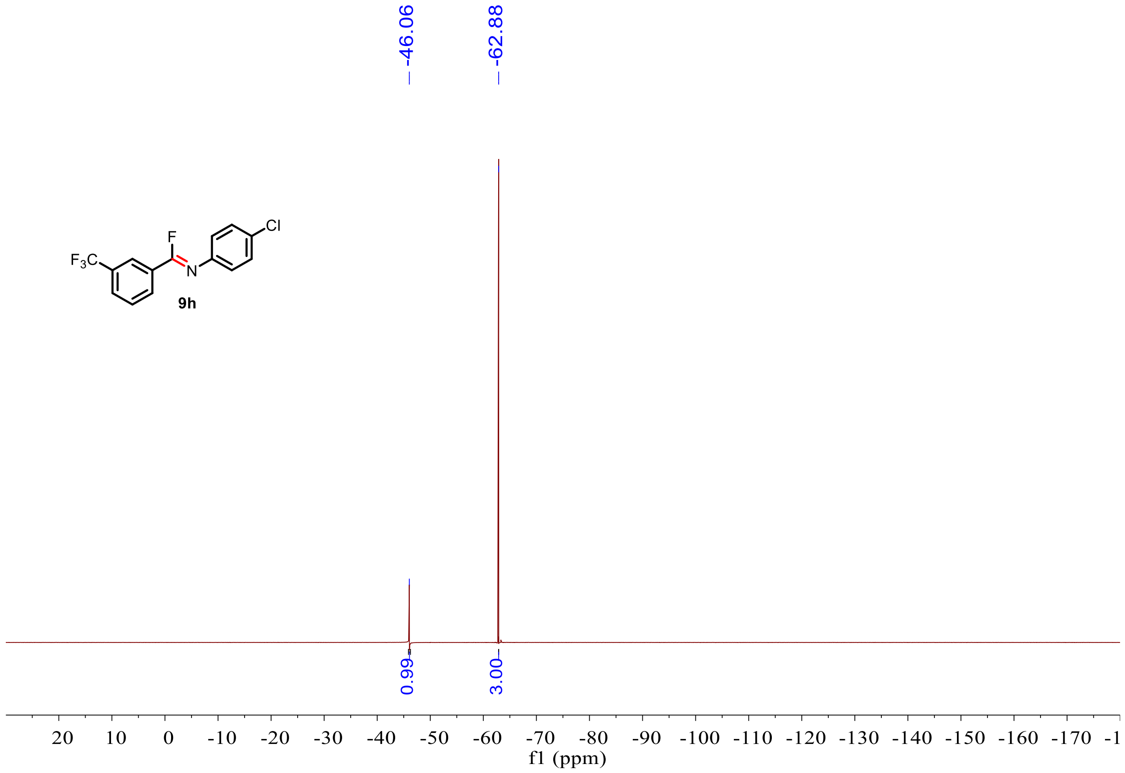

Supplementary Figure 189. ^{19}**F NMR** (471 MHz, $CDCl_3$) spectrum for compound **9h**

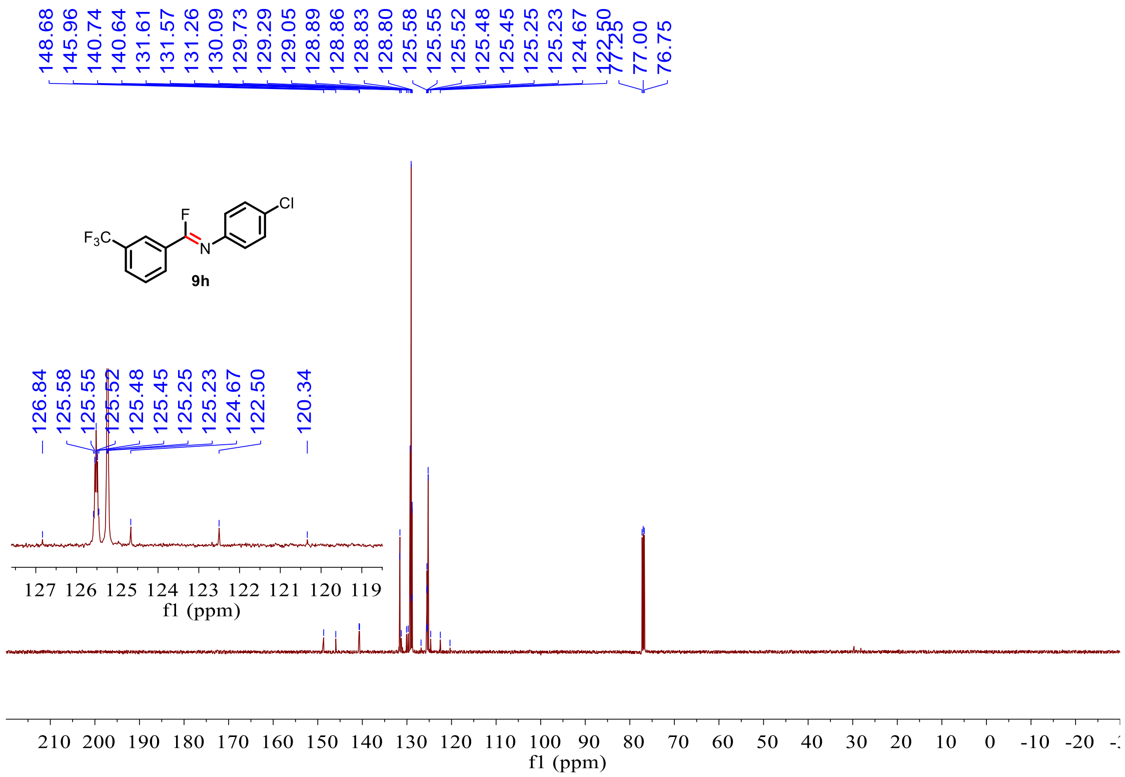

Supplementary Figure 190. ^{13}C NMR (126 MHz, $CDCl_3$) spectrum for compound **9h**

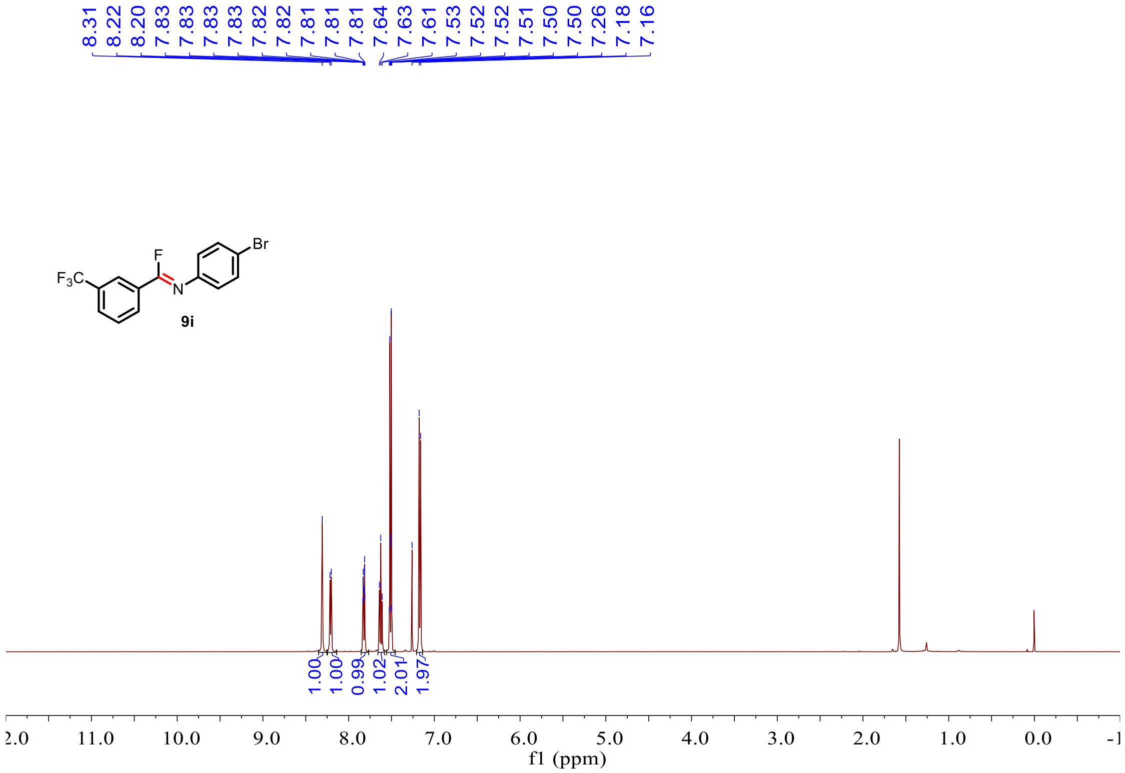

Supplementary Figure 191. ^{1}H NMR (500 MHz, $CDCl_3$) spectrum for compound **9i**

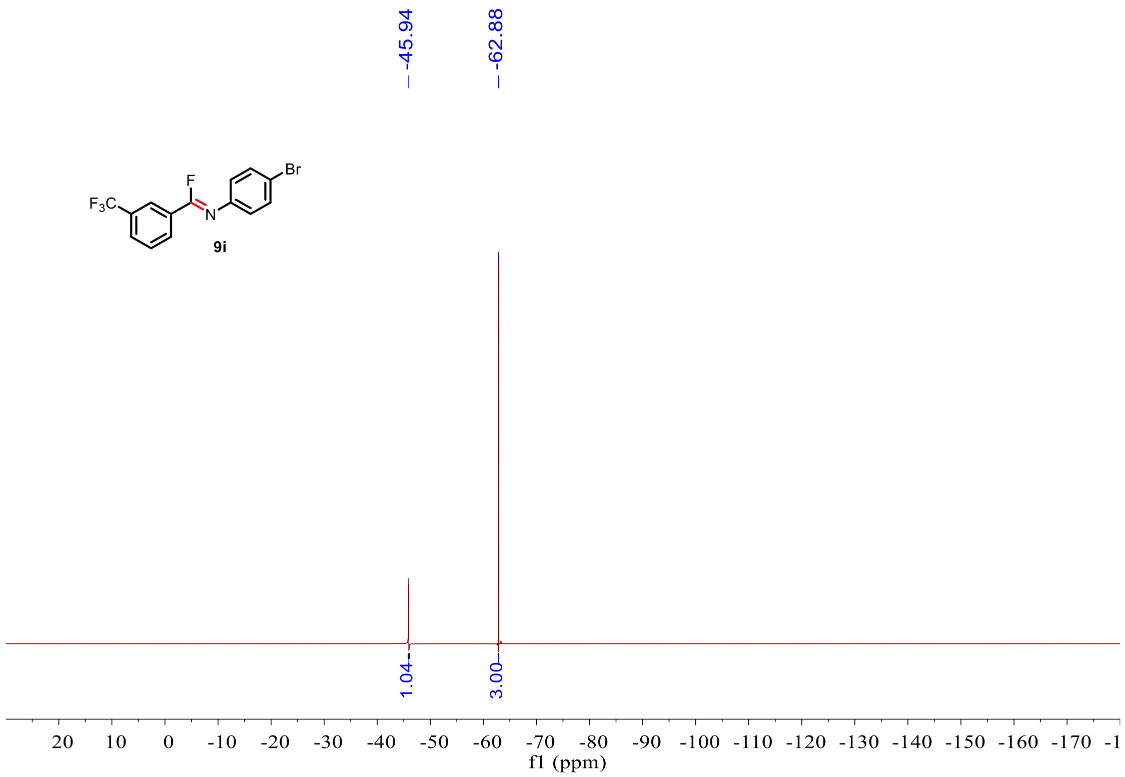

Supplementary Figure 192. ^{19}**F NMR** (471 MHz, $CDCl_3$) spectrum for compound **9i**

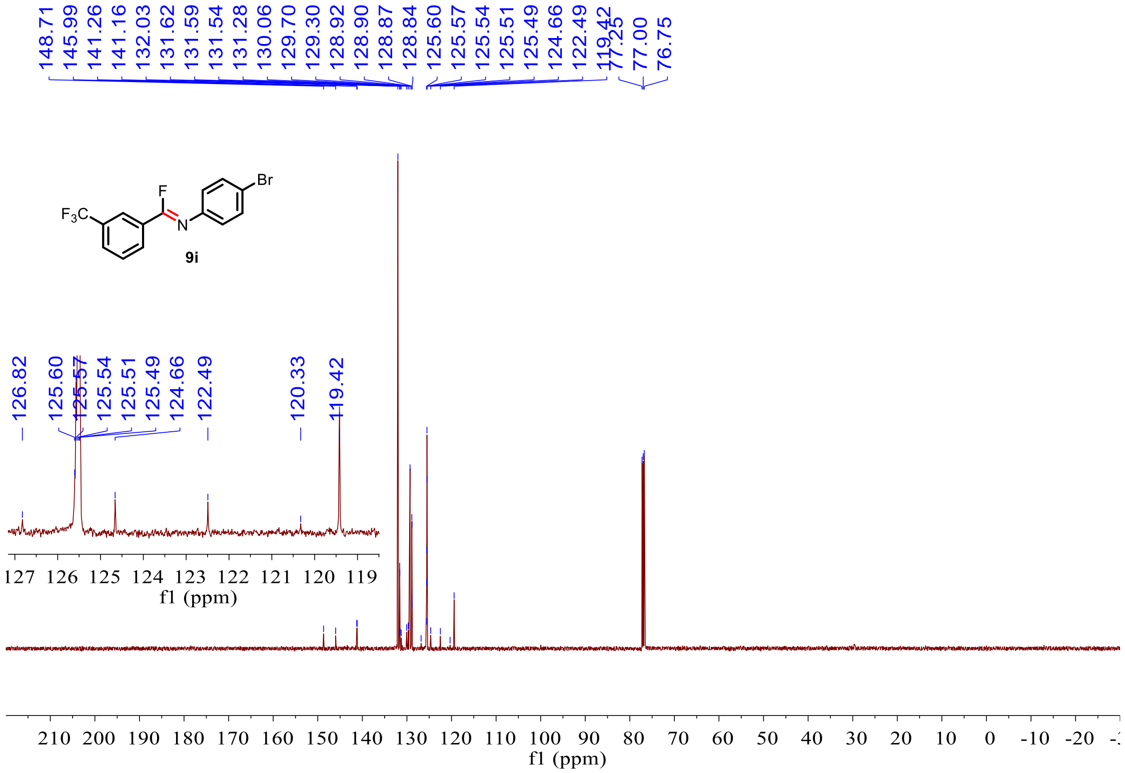

Supplementary Figure 193. ^{13}**C NMR** (126 MHz, $CDCl_3$) spectrum for compound **9i**

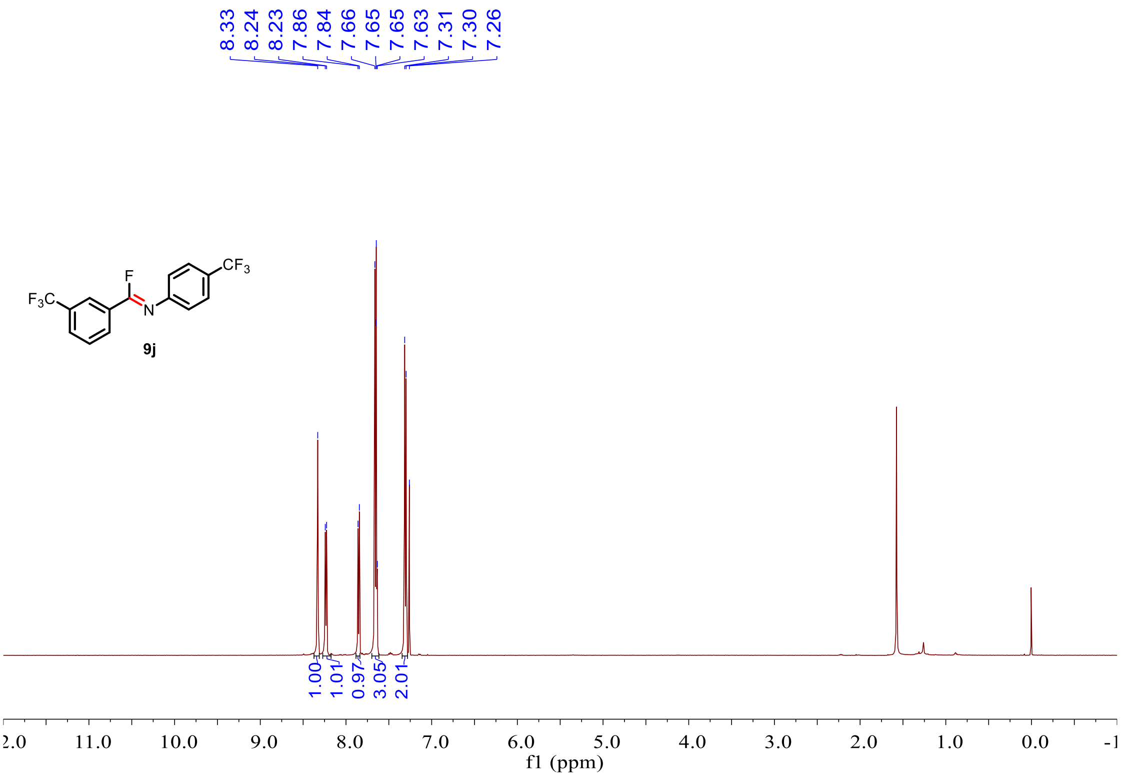

Supplementary Figure 194. ^{1}**H NMR** (500 MHz, $CDCl_3$) spectrum for compound **9j**

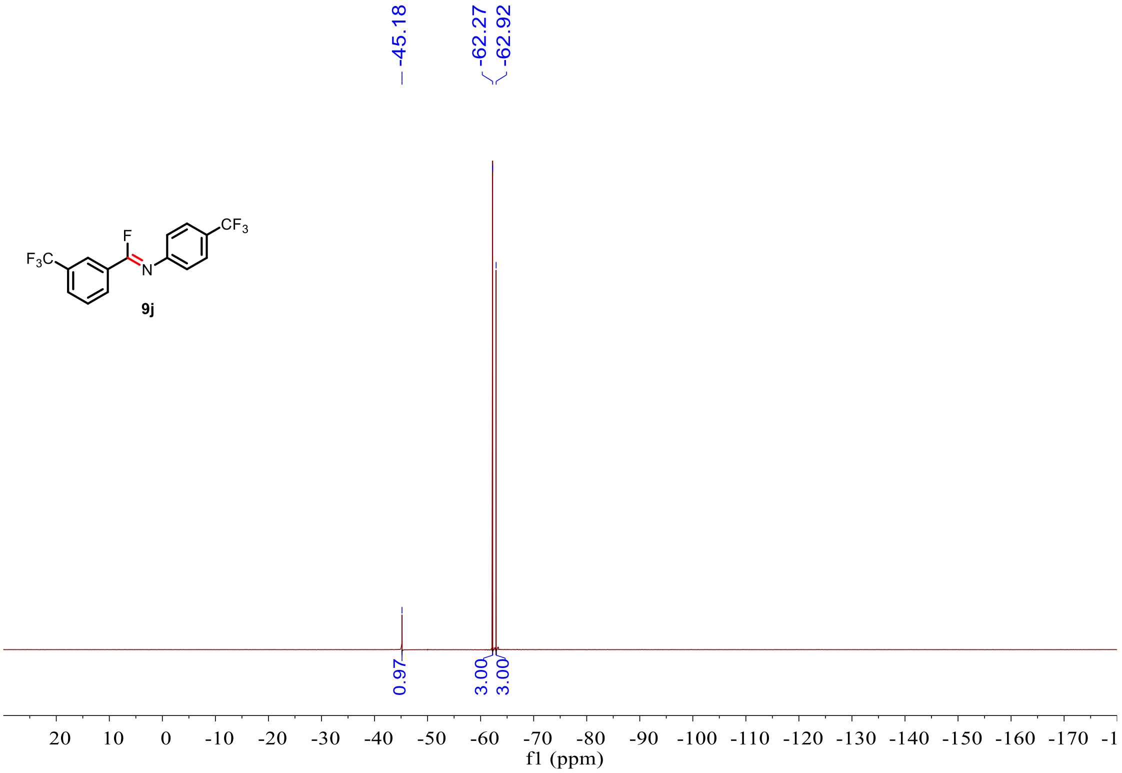

Supplementary Figure 195. ^{19}**F NMR** (471 MHz, $CDCl_3$) spectrum for compound **9j**

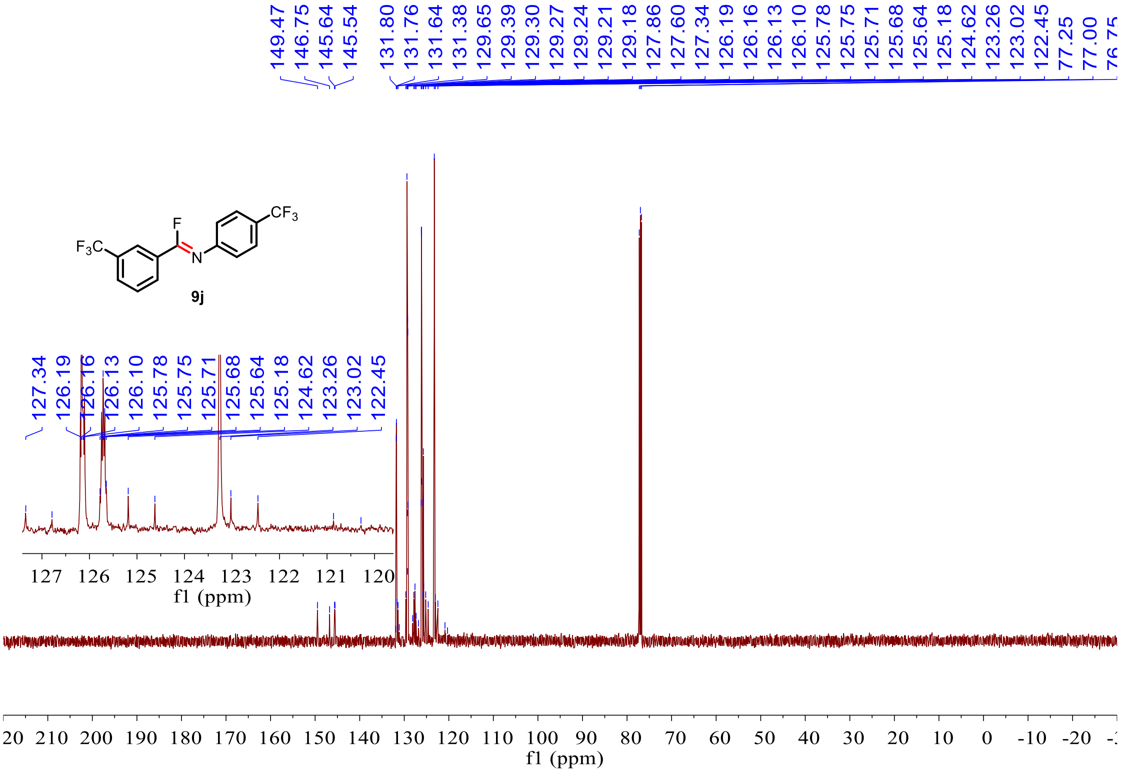

Supplementary Figure 196. ^{13}**C NMR** (126 MHz, $CDCl_3$) spectrum for compound **9j**

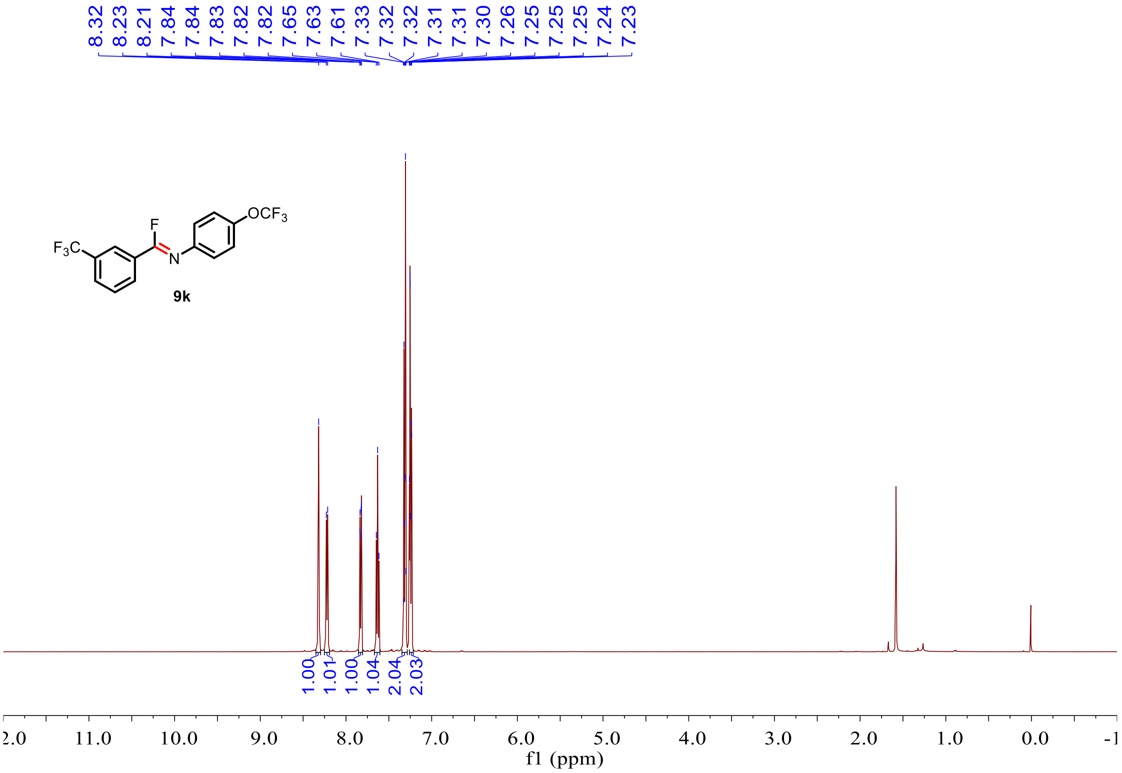

Supplementary Figure 197. ^{1}**H NMR** (500 MHz, $CDCl_3$) spectrum for compound **9k**

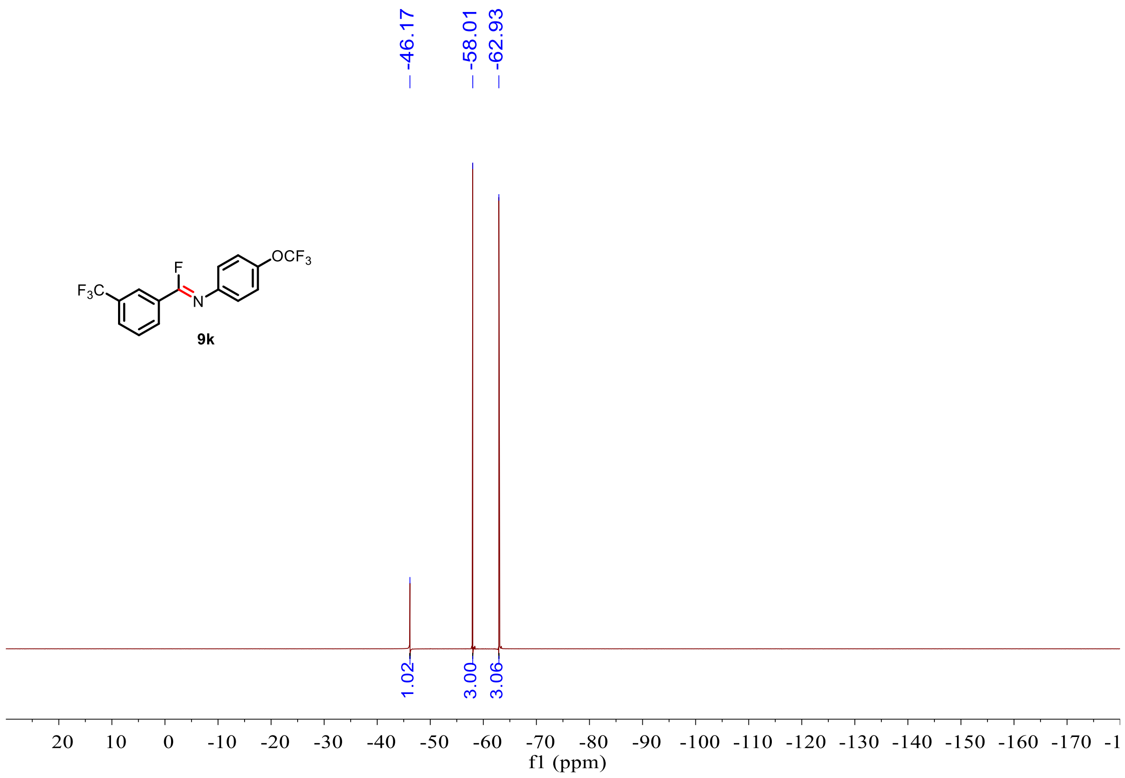

Supplementary Figure 198. **^{19}F NMR** (471 MHz, $CDCl_3$) spectrum for compound **9k**

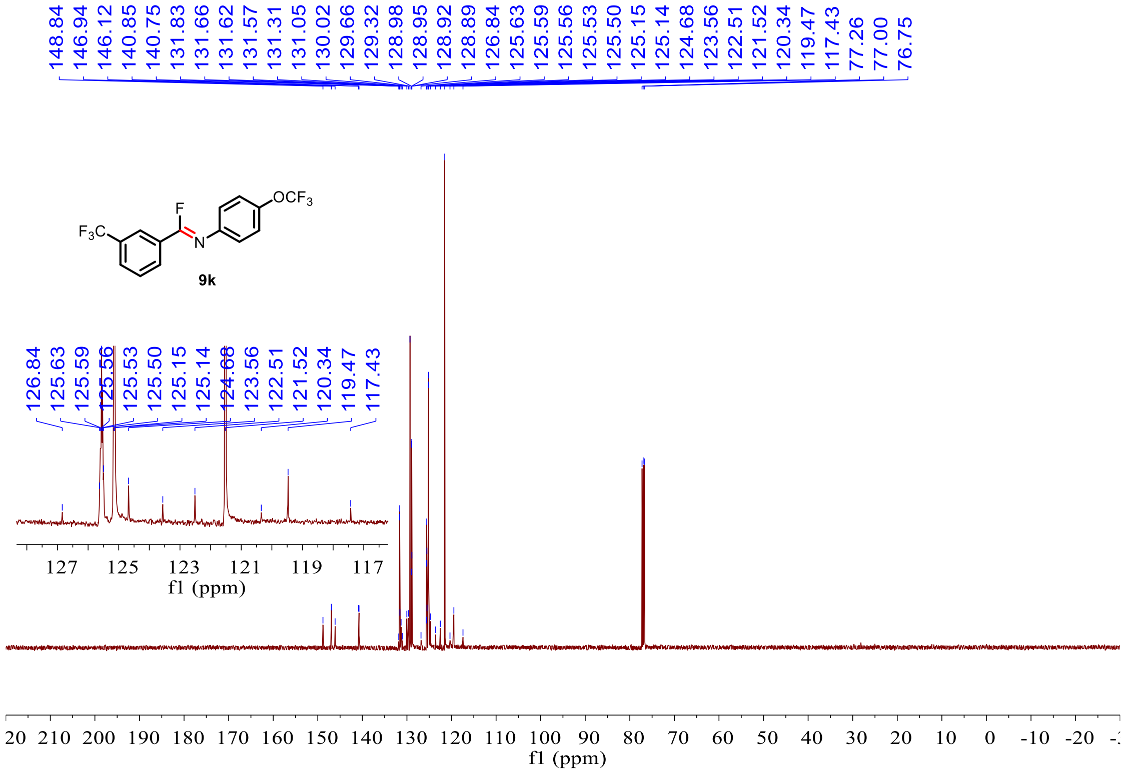

Supplementary Figure 199. **^{13}C NMR** (126 MHz, $CDCl_3$) spectrum for compound **9k**

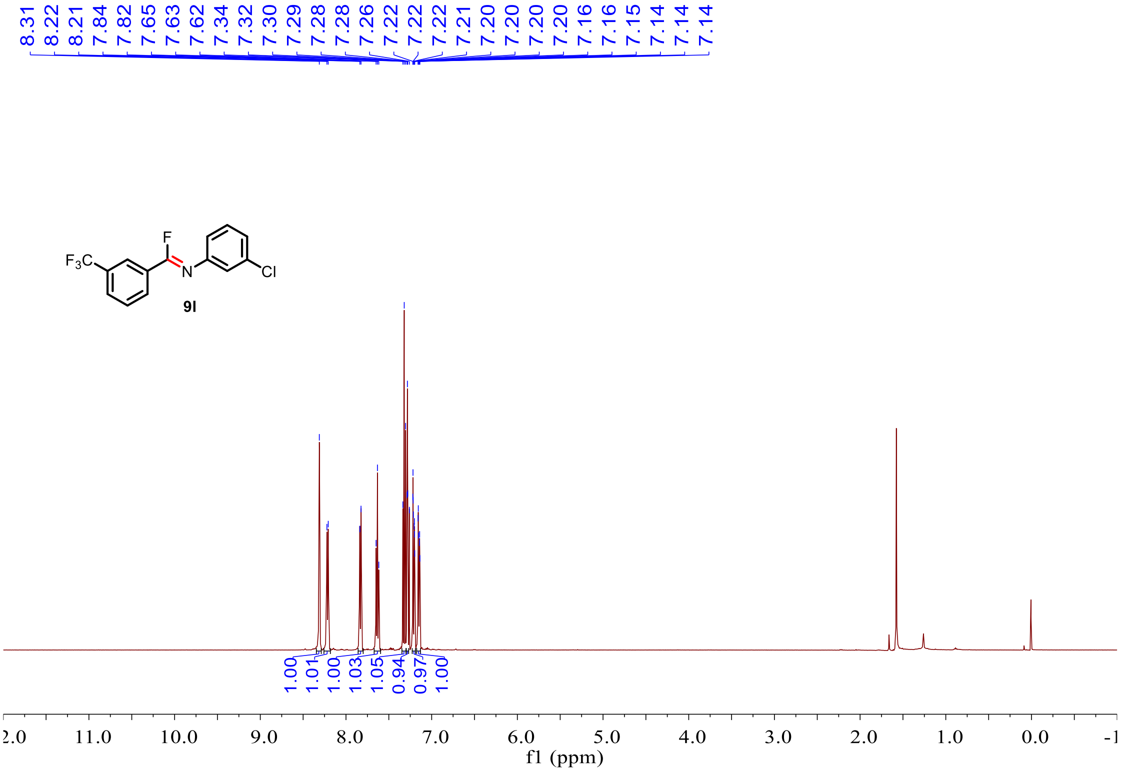

Supplementary Figure 200. **^{1}H NMR** (500 MHz, $CDCl_3$) spectrum for compound **9l**

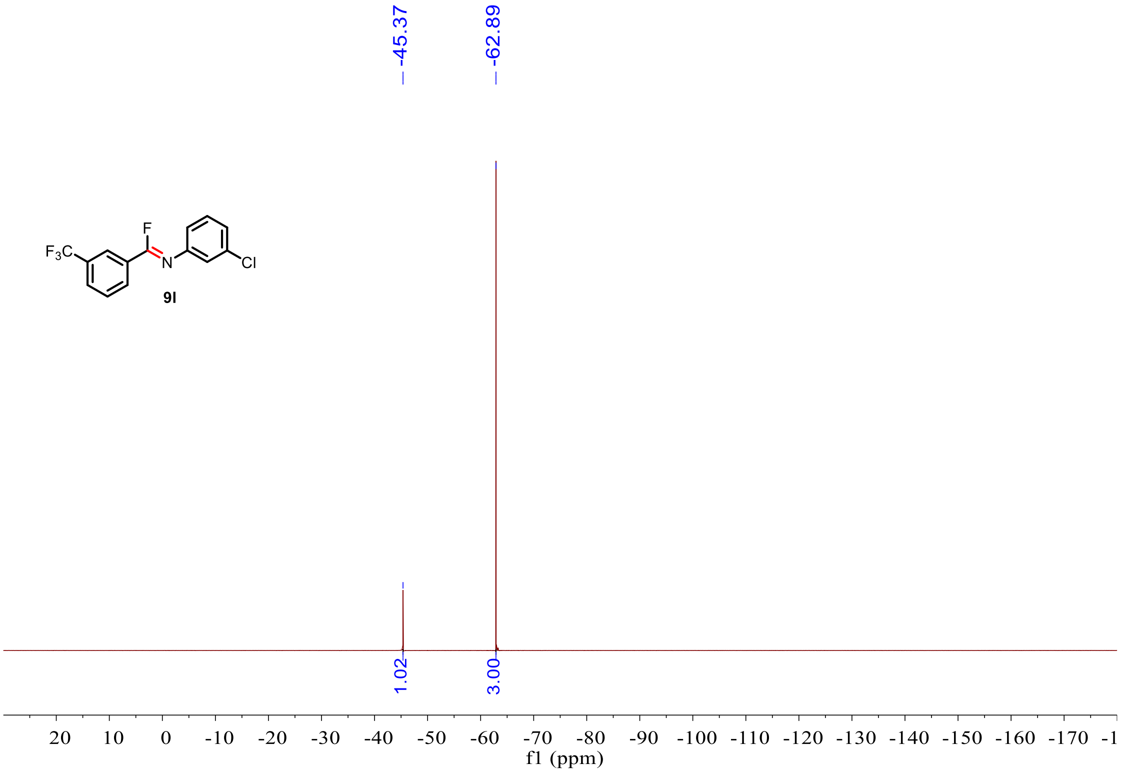

Supplementary Figure 201. **^{19}F NMR** (471 MHz, $CDCl_3$) spectrum for compound **9l**

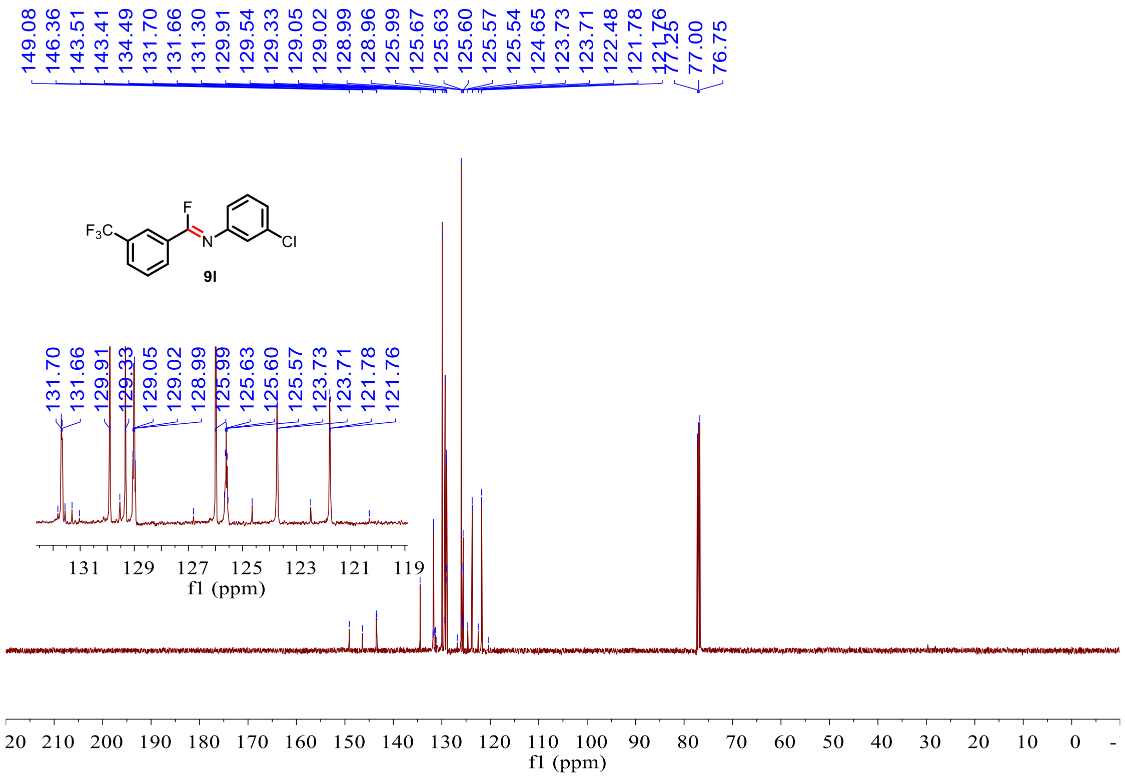

Supplementary Figure 202. ^{13}C NMR (126 MHz, $CDCl_3$) spectrum for compound **9l**

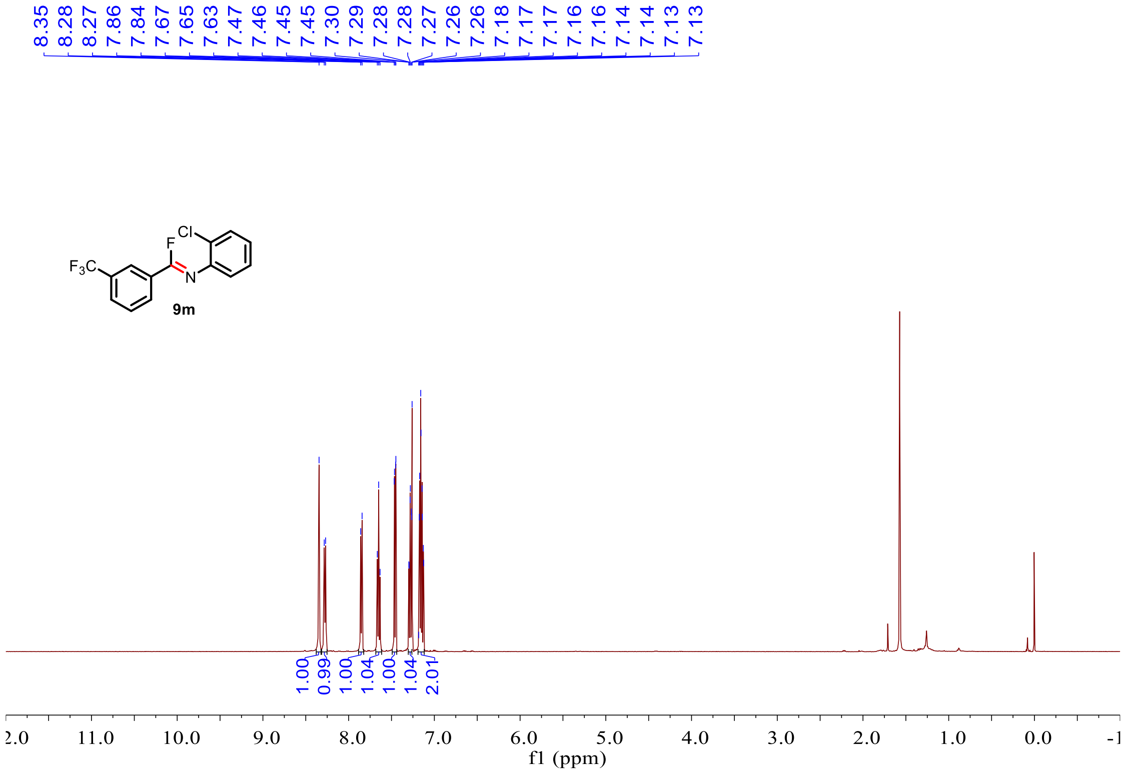

Supplementary Figure 203. ^{1}H NMR (500 MHz, $CDCl_3$) spectrum for compound **9m**

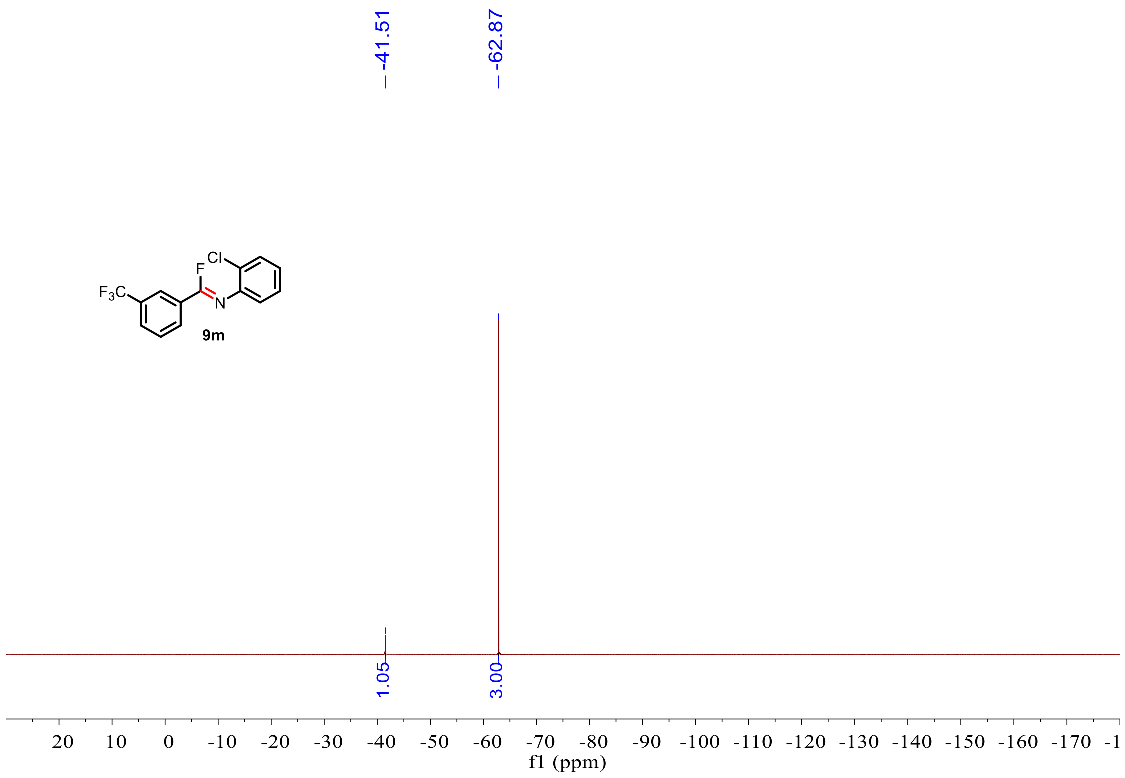

Supplementary Figure 204. ^{19}**F NMR** (471 MHz, $CDCl_3$) spectrum for compound **9m**

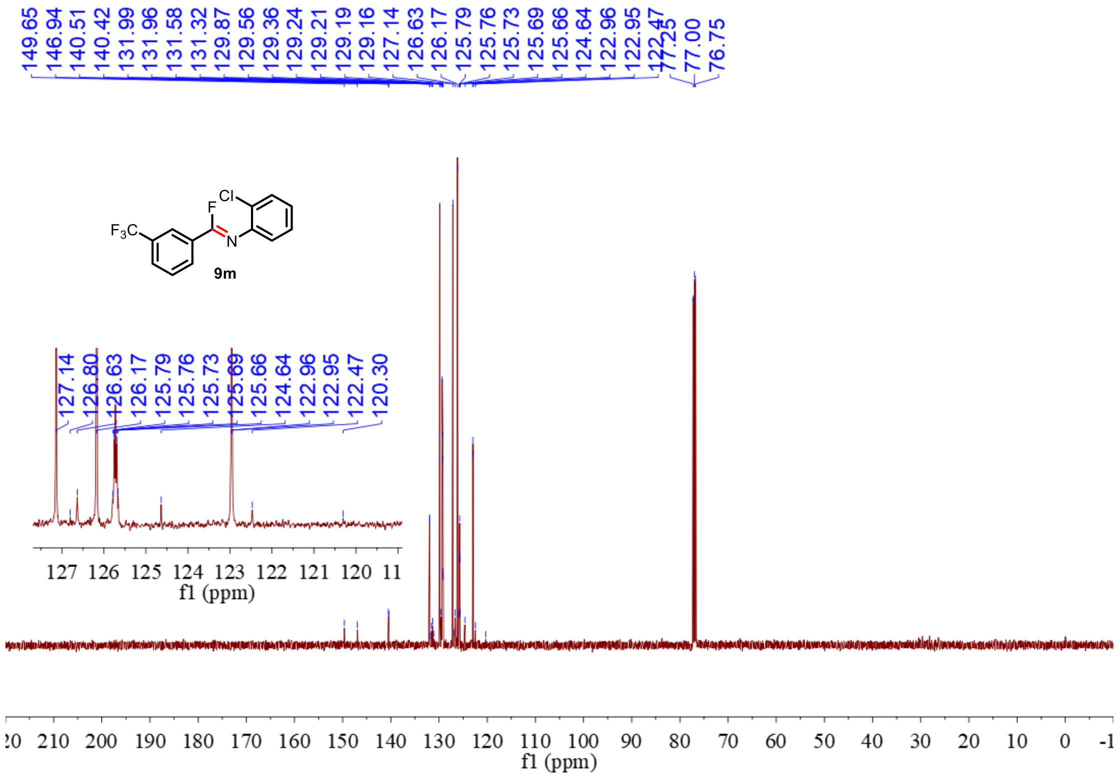

Supplementary Figure 205. ^{13}**C NMR** (126 MHz, $CDCl_3$) spectrum for compound **9m**

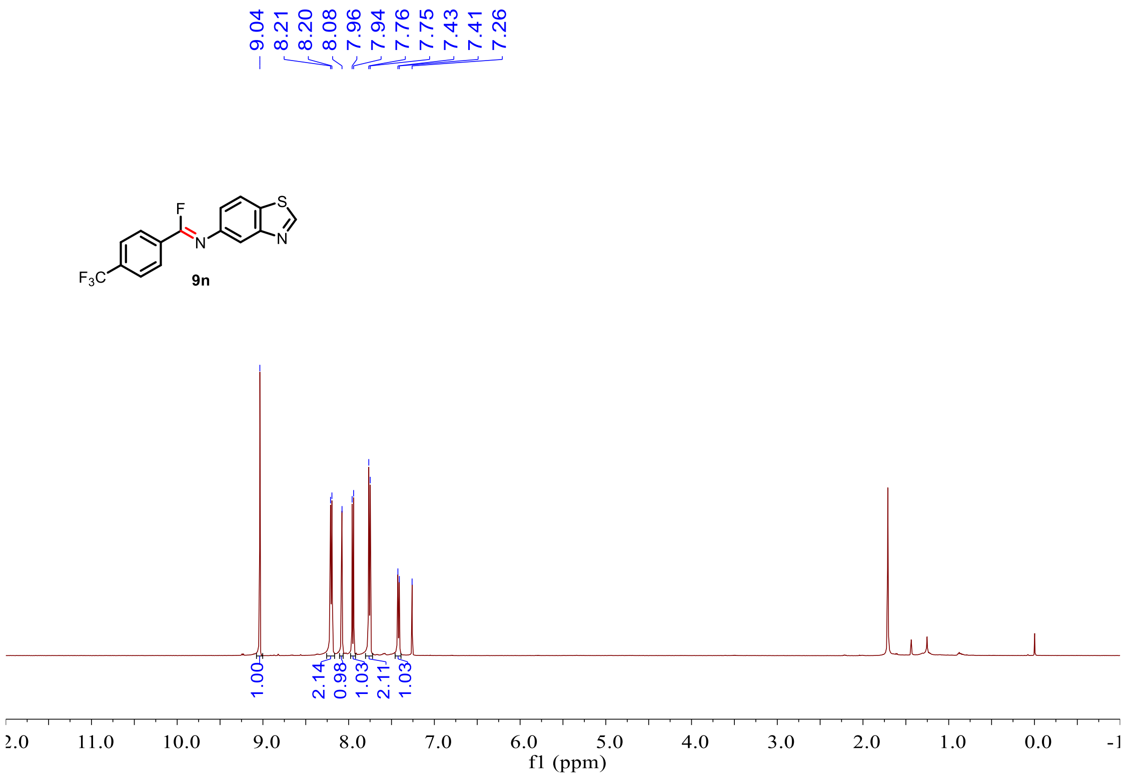

Supplementary Figure 206. **^{1}H NMR** (500 MHz, $CDCl_3$) spectrum for compound **9n**

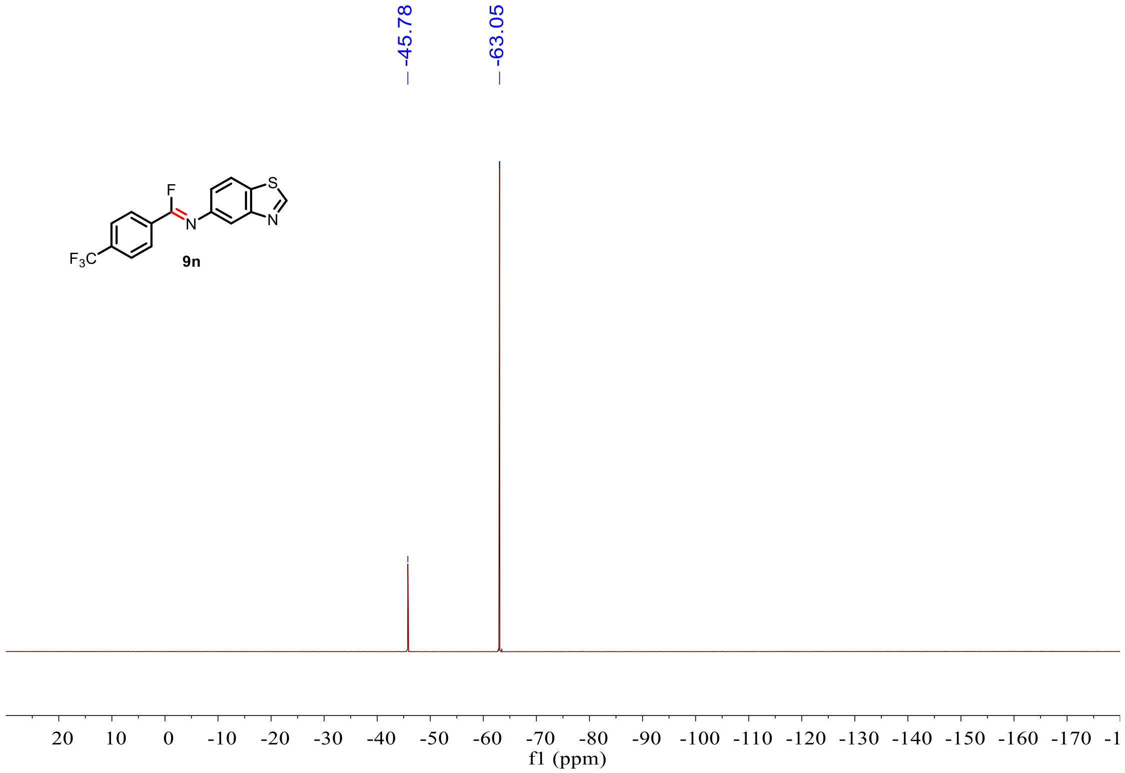

Supplementary Figure 207. **^{19}F NMR** (471 MHz, $CDCl_3$) spectrum for compound **9n**

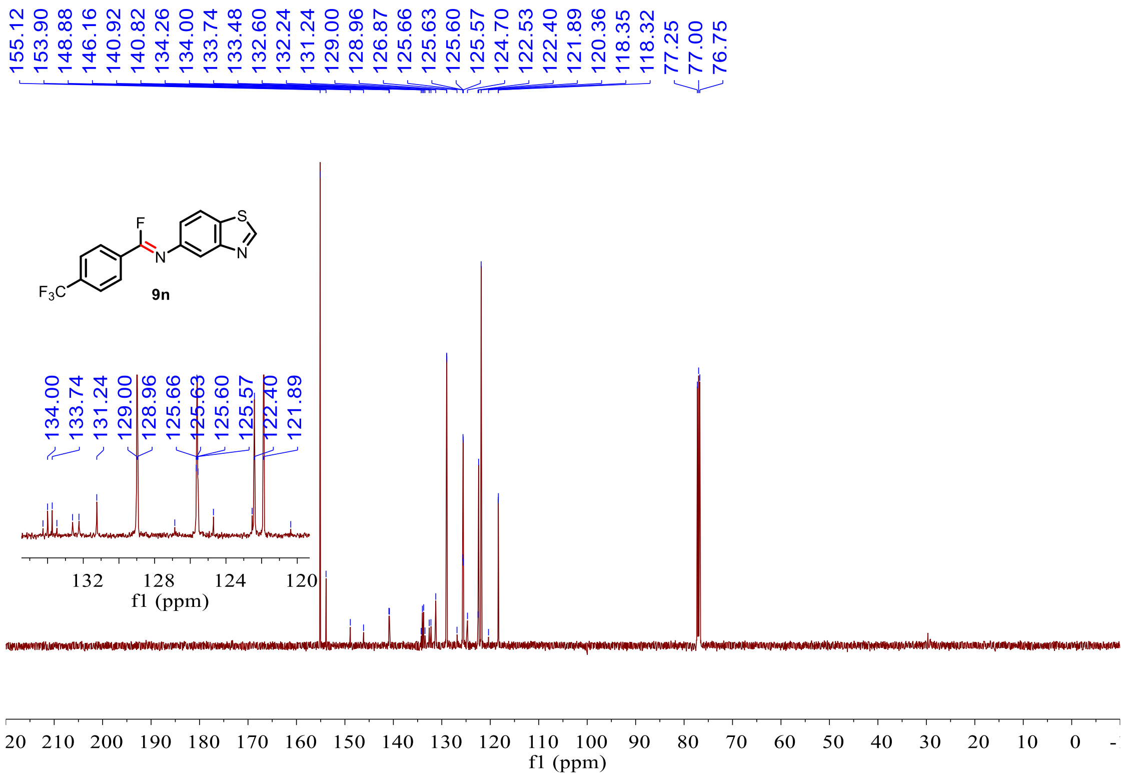

Supplementary Figure 208. ^{13}**C NMR** (126 MHz, $CDCl_3$) spectrum for compound **9n**

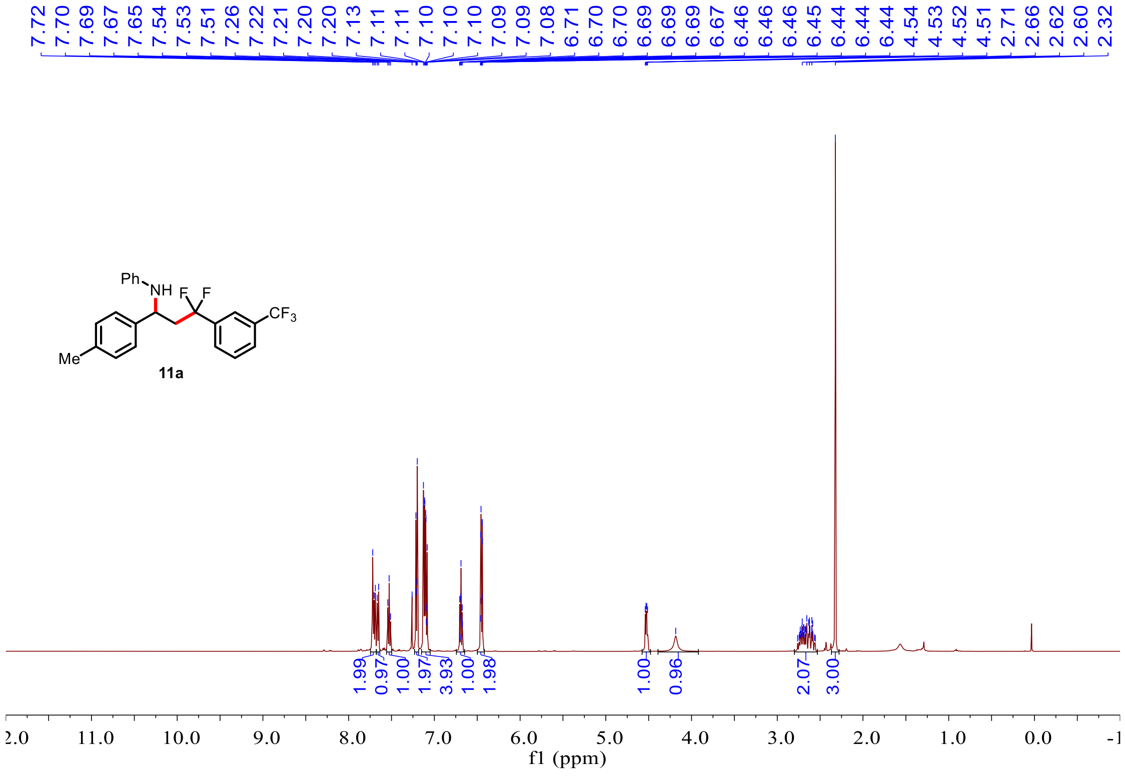

Supplementary Figure 209. ^{1}**H NMR** (500 MHz, $CDCl_3$) spectrum for compound **11a**

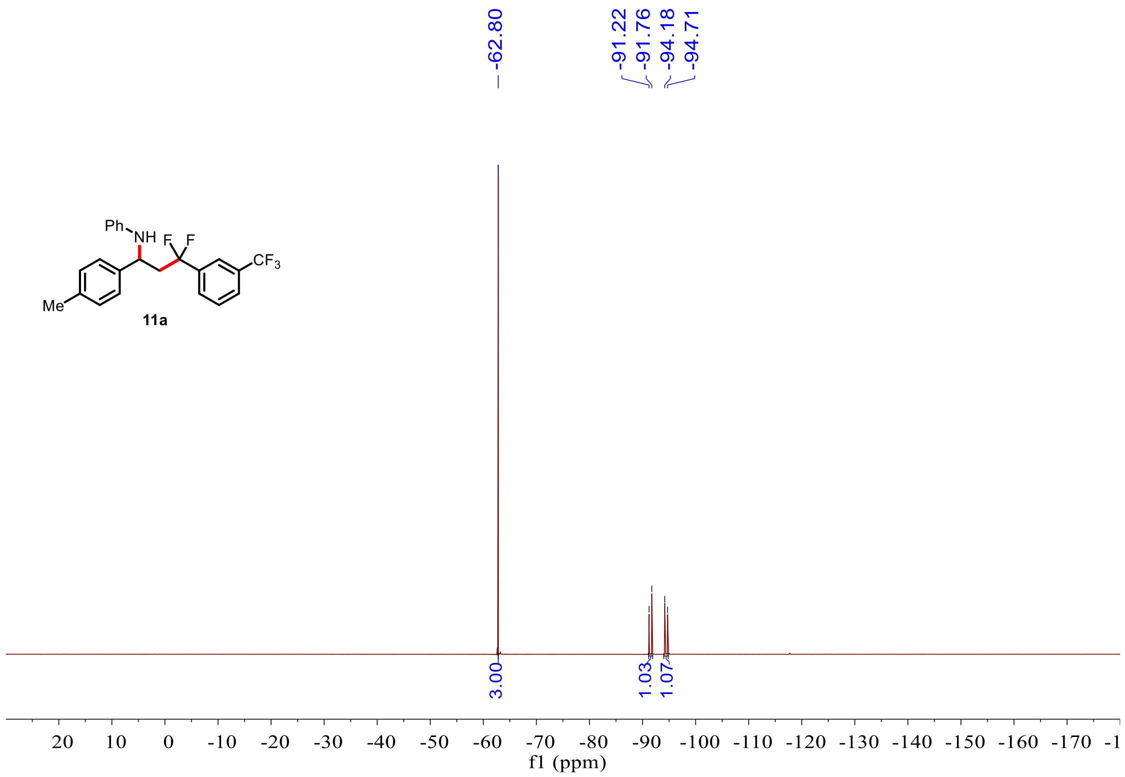

Supplementary Figure 210. ^{19}**F NMR** (471 MHz, $CDCl_3$) spectrum for compound **11a**

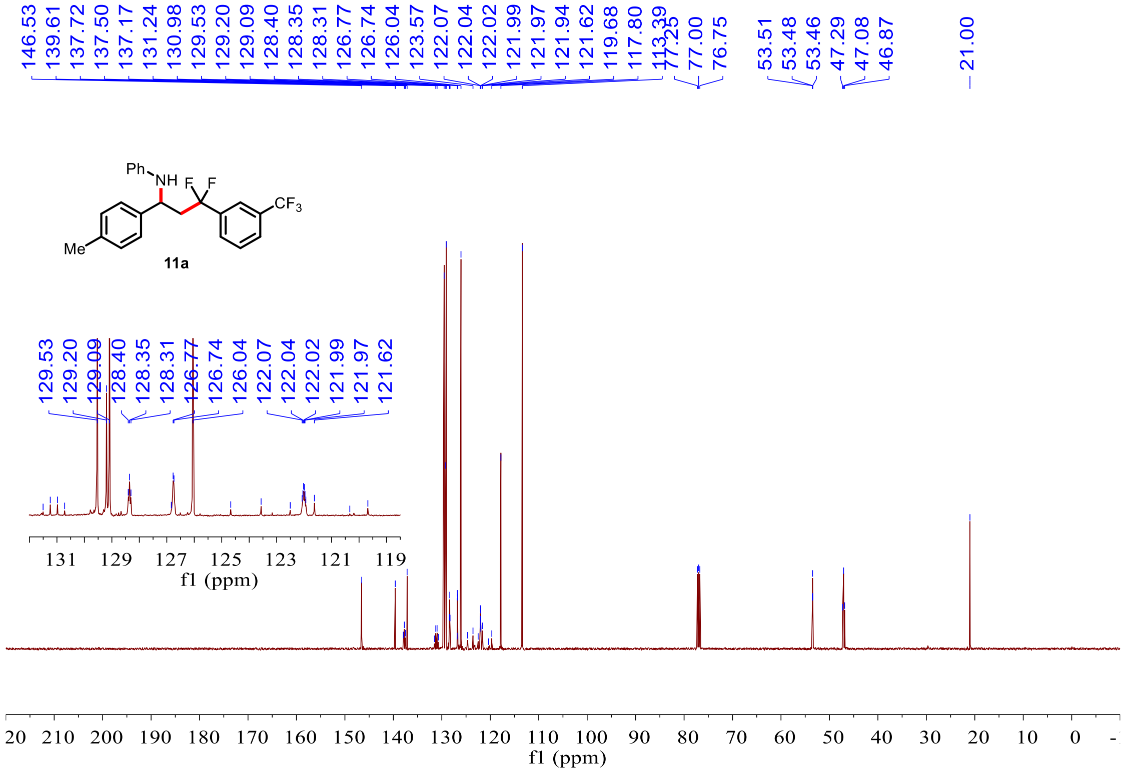

Supplementary Figure 211. ^{13}**C NMR** (126 MHz, $CDCl_3$) spectrum for compound **11a**

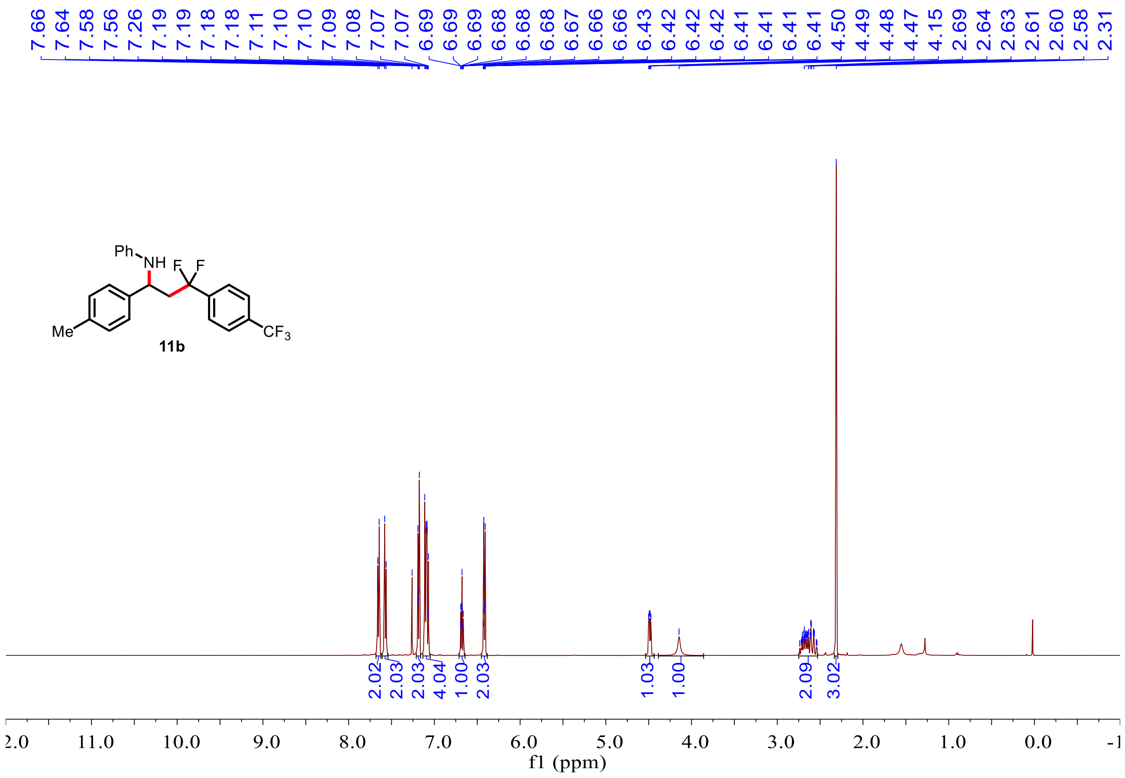

Supplementary Figure 212. ^{1}**H NMR** (500 MHz, $CDCl_3$) spectrum for compound **11b**

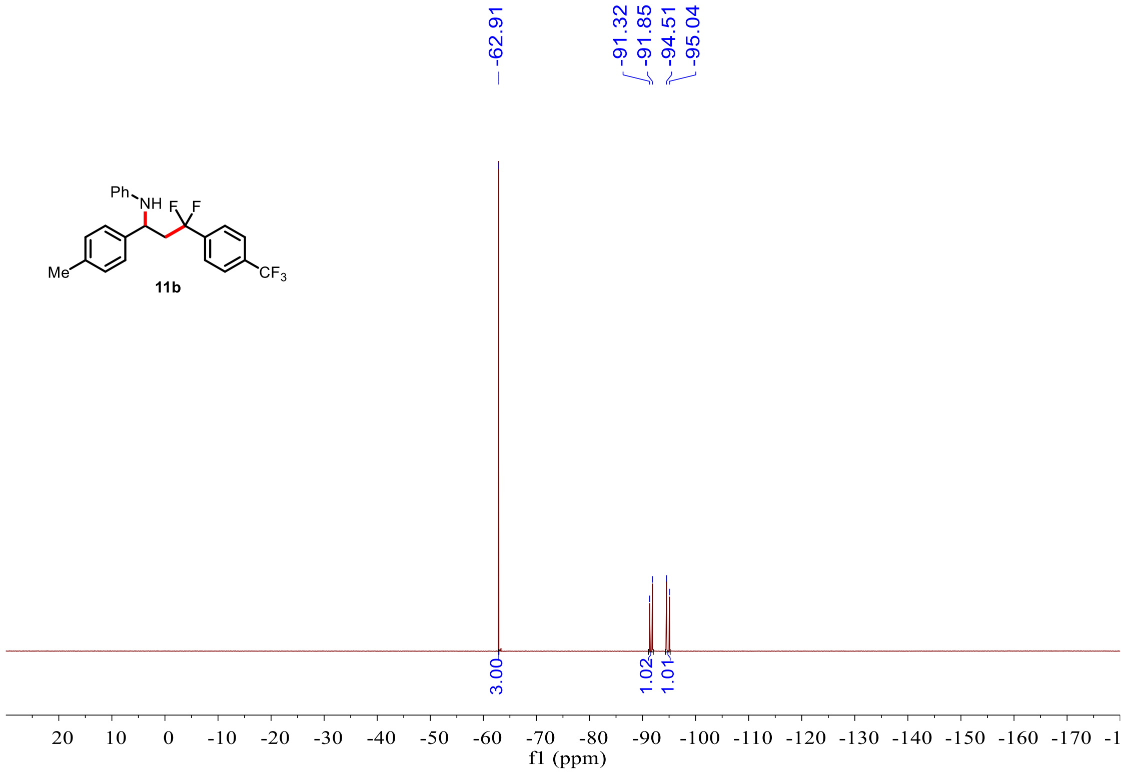

Supplementary Figure 213. ^{19}**F NMR** (471 MHz, $CDCl_3$) spectrum for compound **11b**

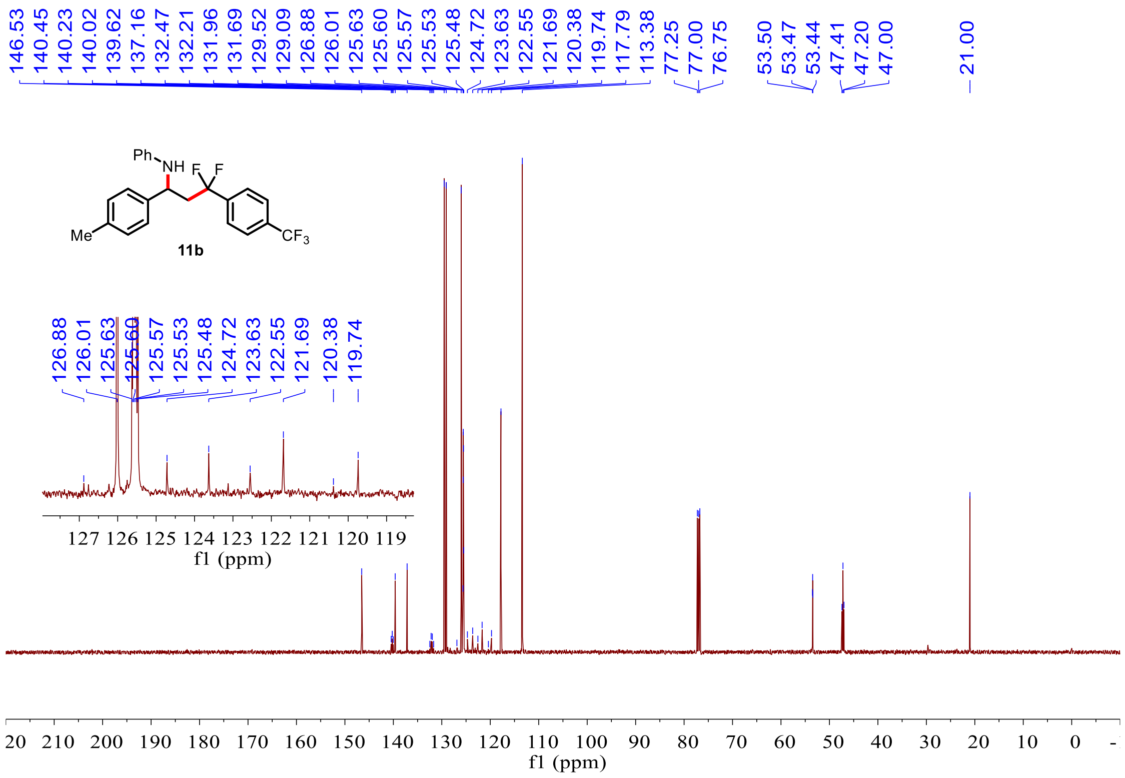

Supplementary Figure 214. **^{13}C NMR** (126 MHz, $CDCl_3$) spectrum for compound **11b**

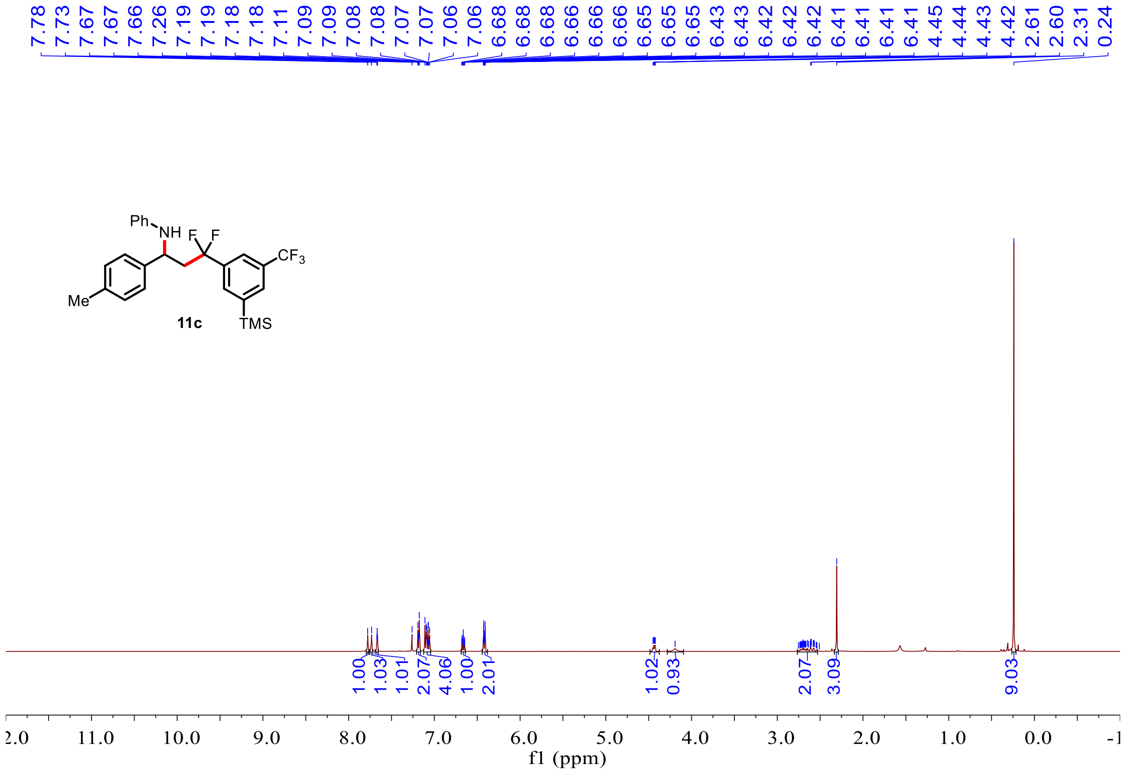

Supplementary Figure 215. **^{1}H NMR** (500 MHz, $CDCl_3$) spectrum for compound **11c**

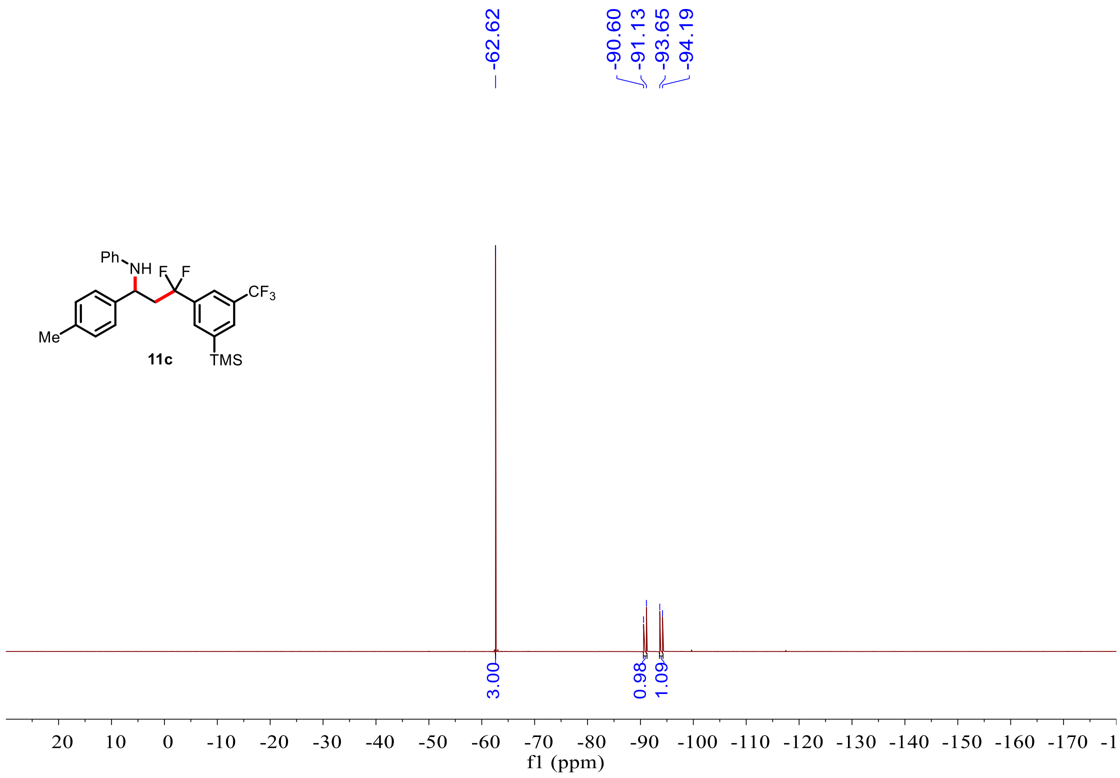

Supplementary Figure 216. ^{19}F NMR (471 MHz, $CDCl_3$) spectrum for compound **11c**

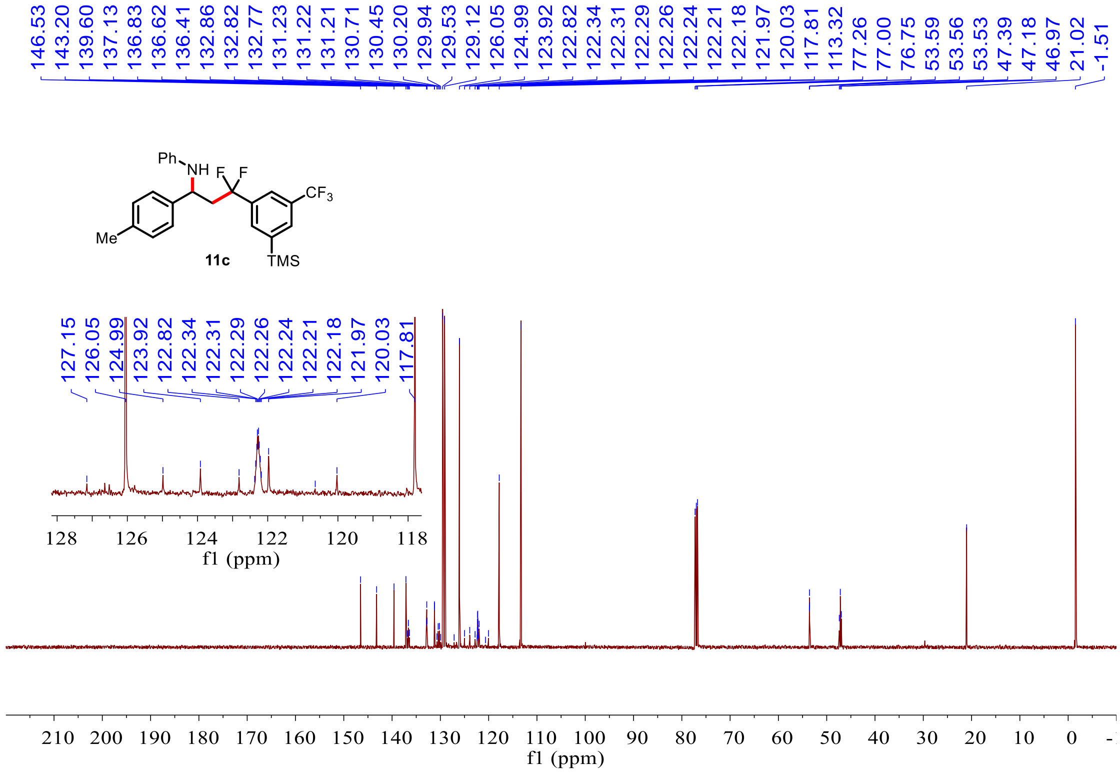

Supplementary Figure 217. ^{13}C NMR (126 MHz, $CDCl_3$) spectrum for compound **11c**

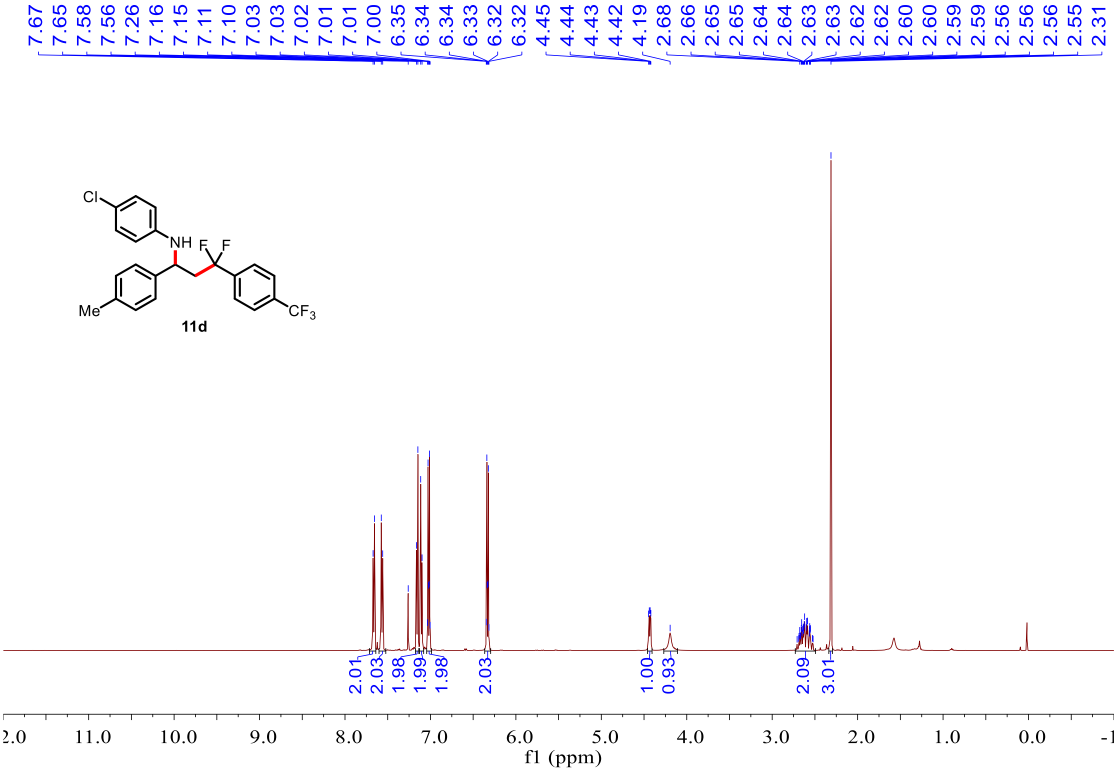

Supplementary Figure 218. ^{1}**H NMR** (500 MHz, $CDCl_3$) spectrum for compound **11d**

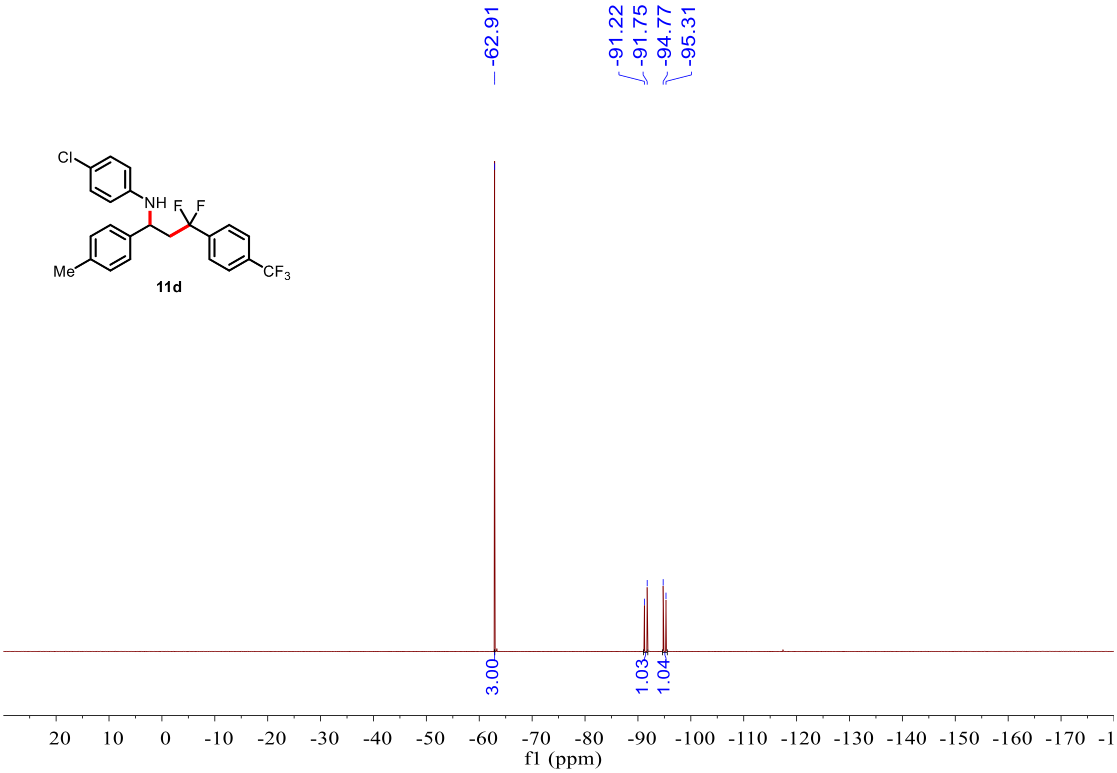

Supplementary Figure 219. ^{19}**F NMR** (471 MHz, $CDCl_3$) spectrum for compound **11d**

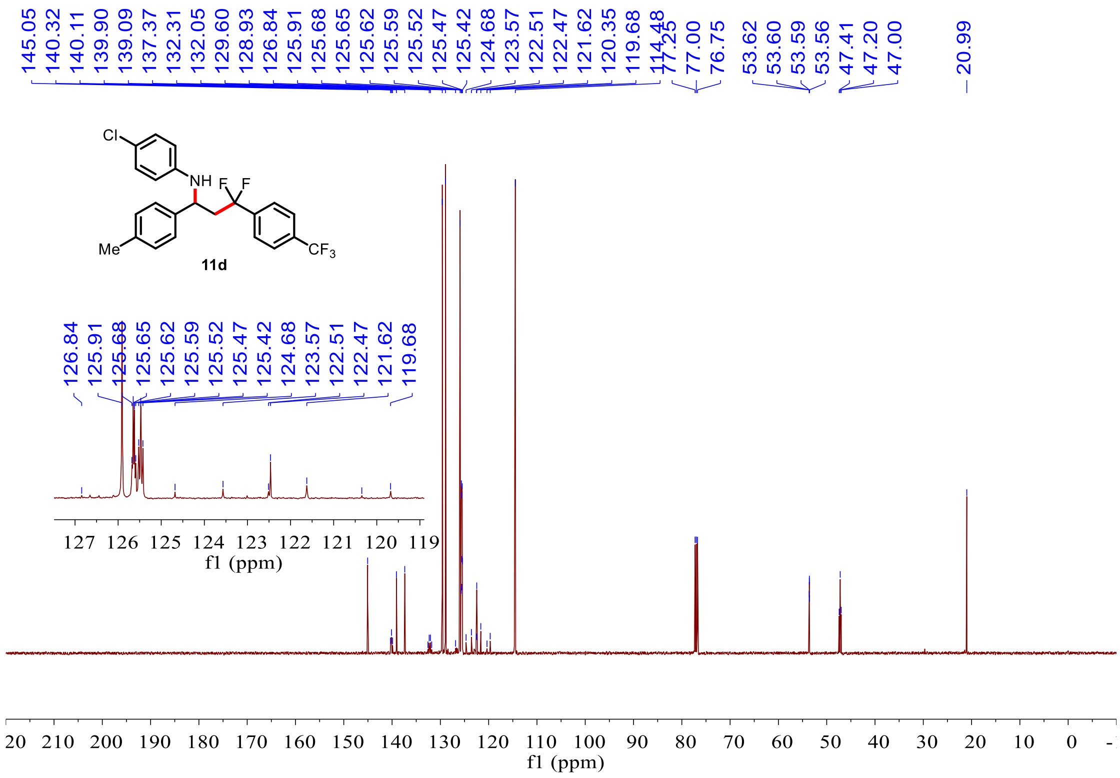

Supplementary Figure 220. ^{13}**C NMR** (126 MHz, $CDCl_3$) spectrum for compound **11d**

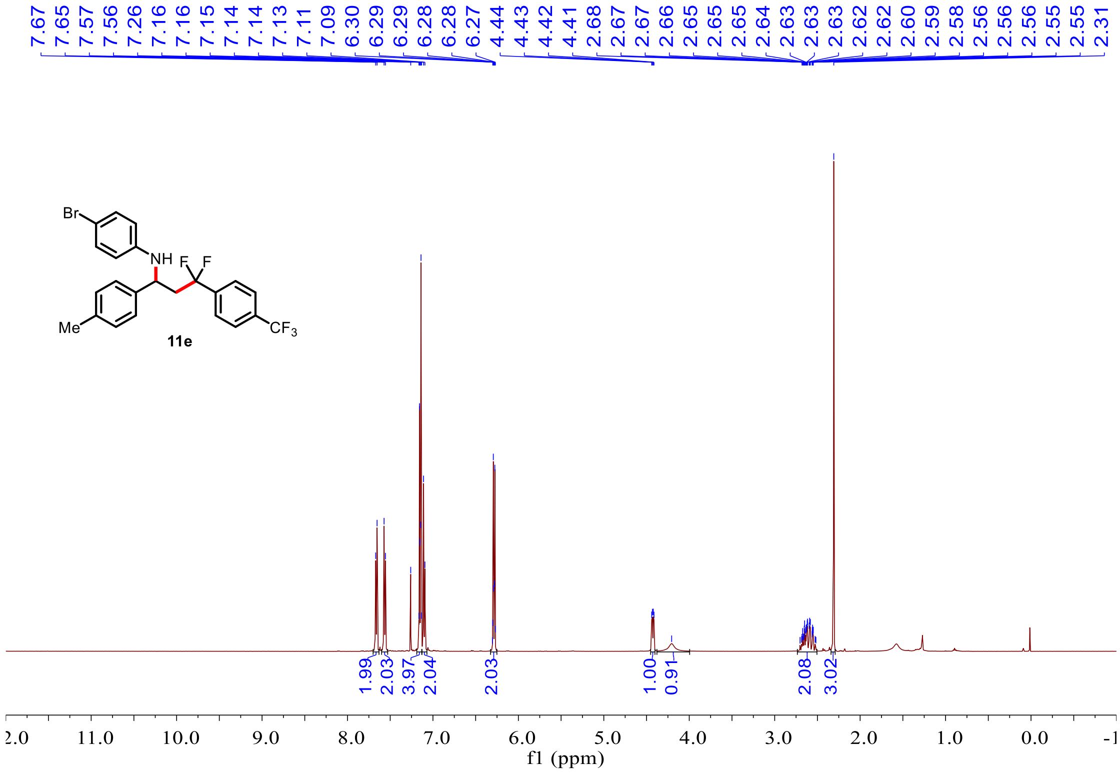

Supplementary Figure 221. ^{1}**H NMR** (500 MHz, $CDCl_3$) spectrum for compound **11e**

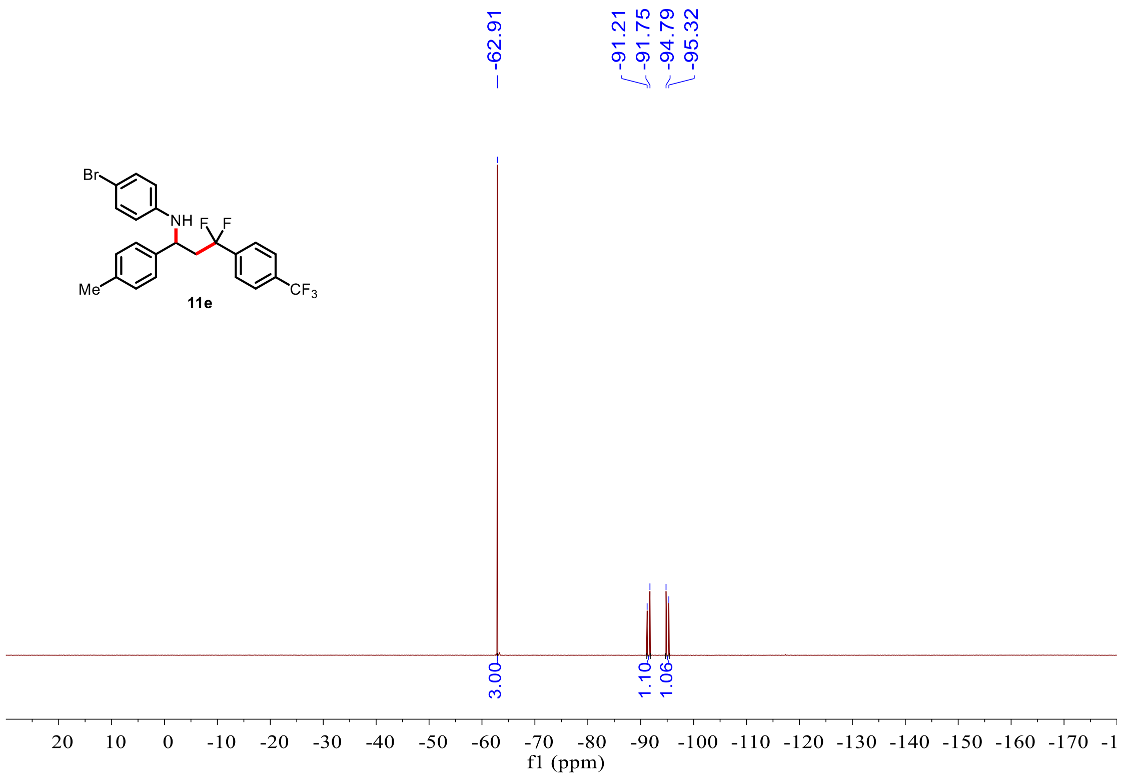

Supplementary Figure 222. ^{19}**F NMR** (471 MHz, $CDCl_3$) spectrum for compound **11e**

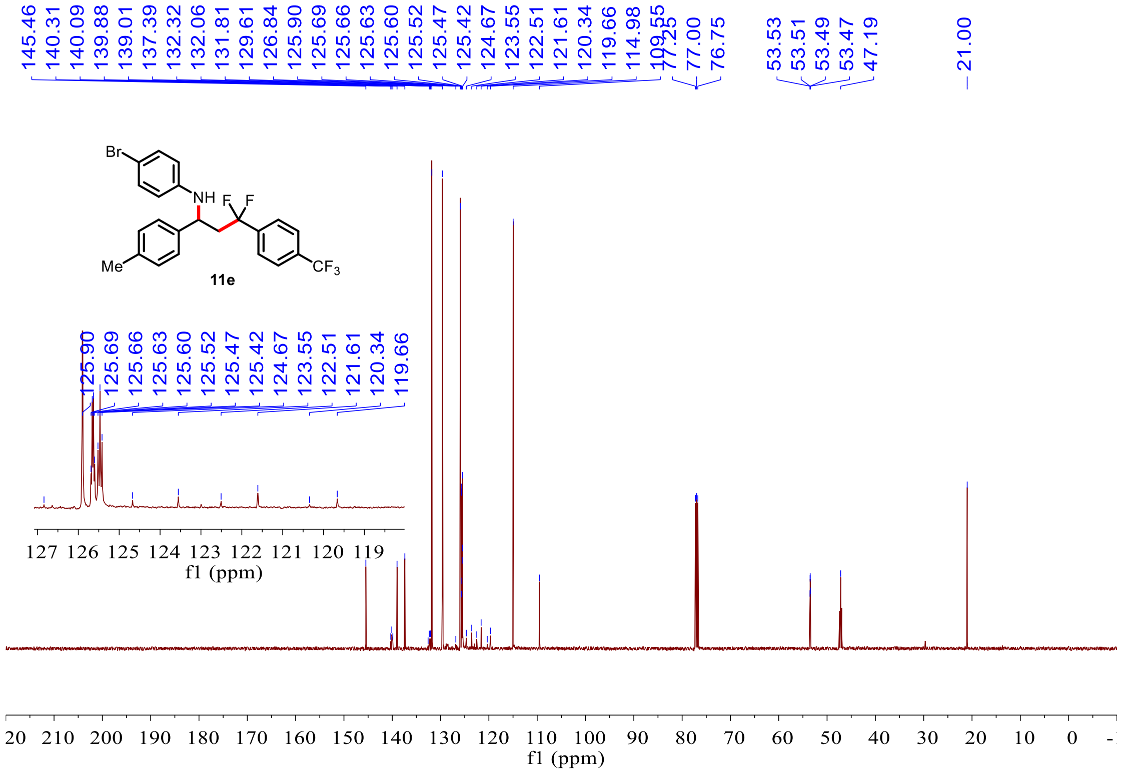

Supplementary Figure 223. ^{13}**C NMR** (126 MHz, $CDCl_3$) spectrum for compound **11e**

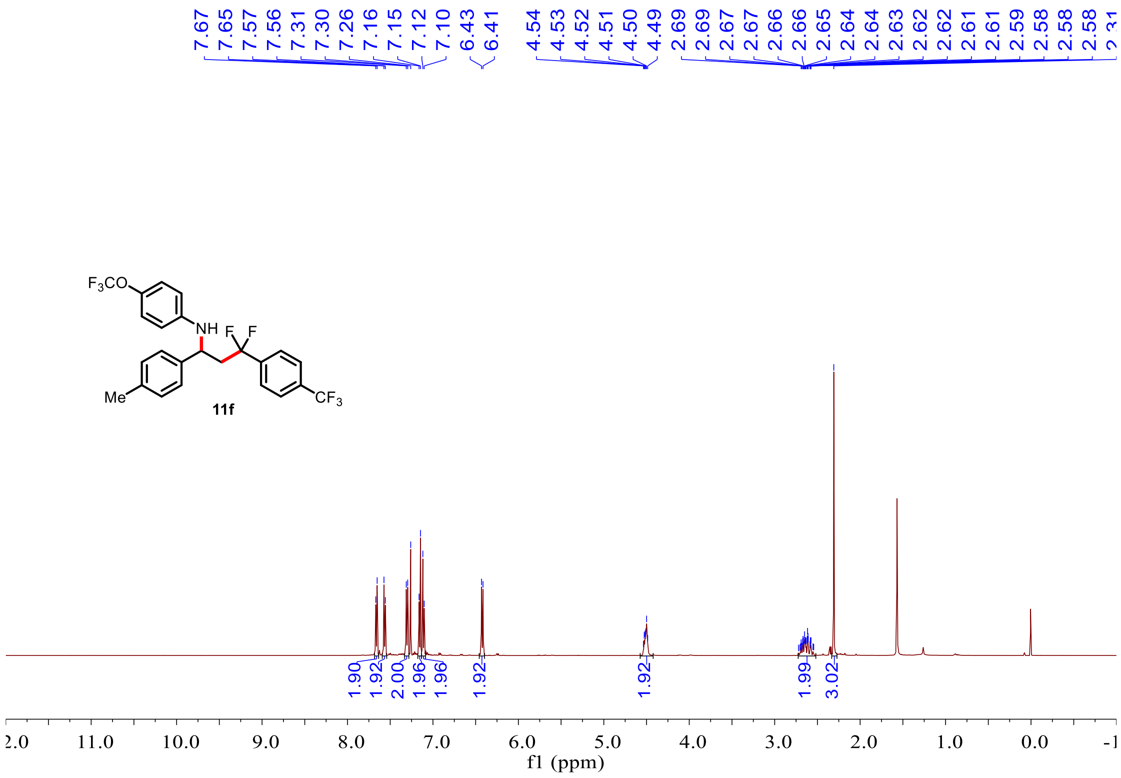

Supplementary Figure 224. ^{1}H NMR (500 MHz, $CDCl_3$) spectrum for compound **11f**

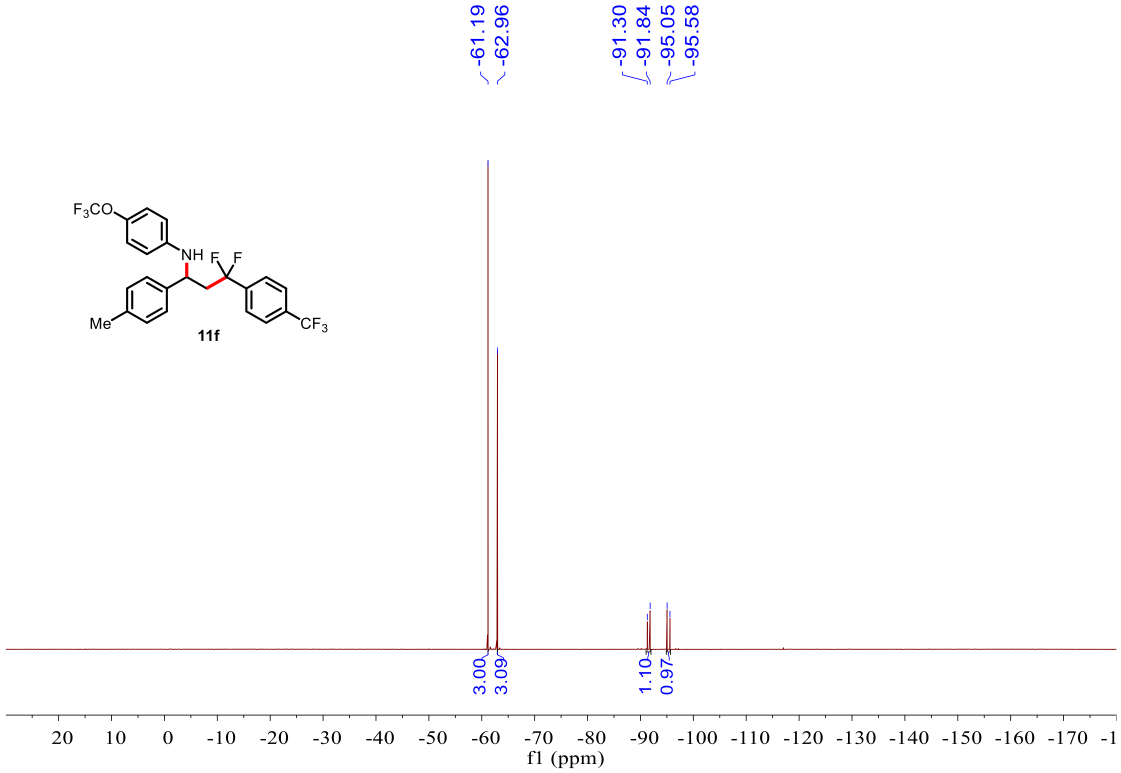

Supplementary Figure 225. ^{19}F NMR (471 MHz, $CDCl_3$) spectrum for compound **11f**

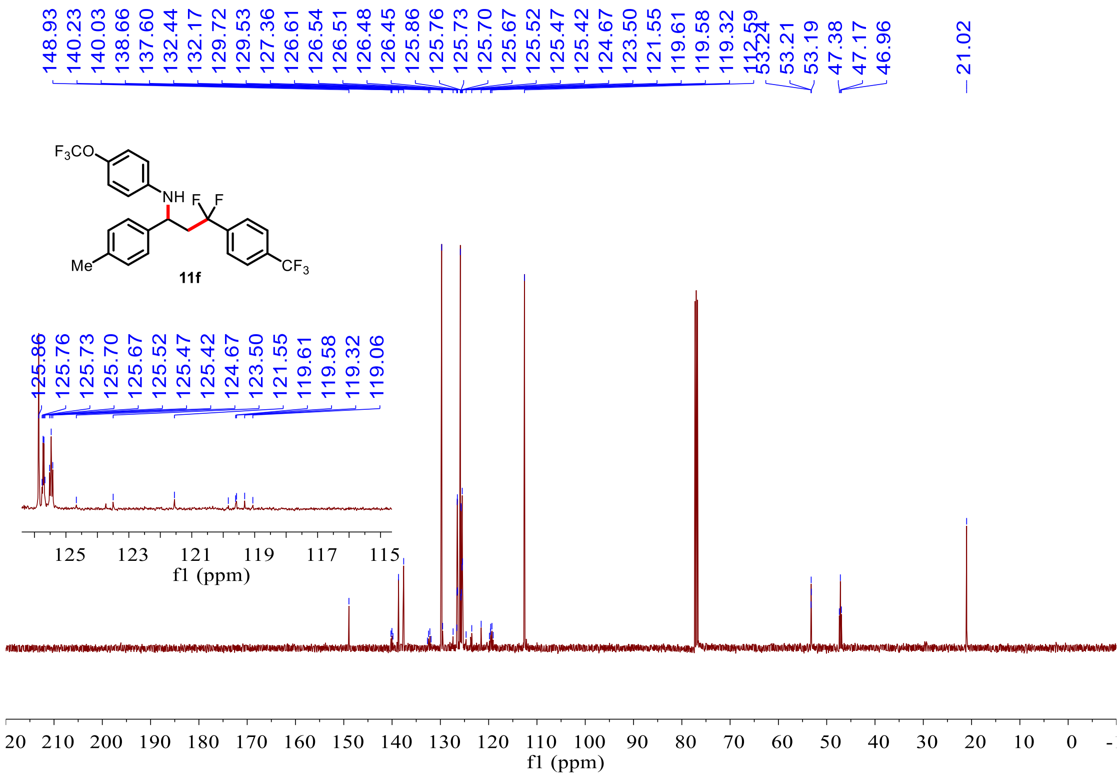

Supplementary Figure 226. ^{13}**C NMR** (126 MHz, $CDCl_3$) spectrum for compound **11f**

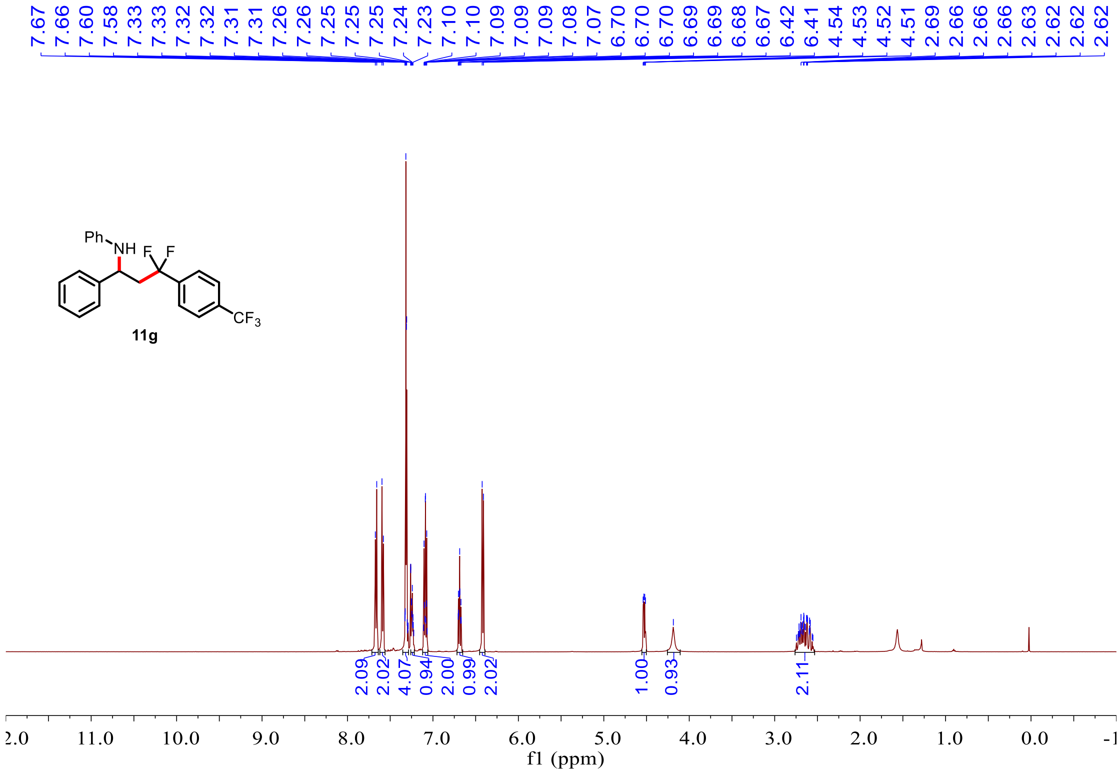

Supplementary Figure 227. ^{1}**H NMR** (500 MHz, $CDCl_3$) spectrum for compound **11g**

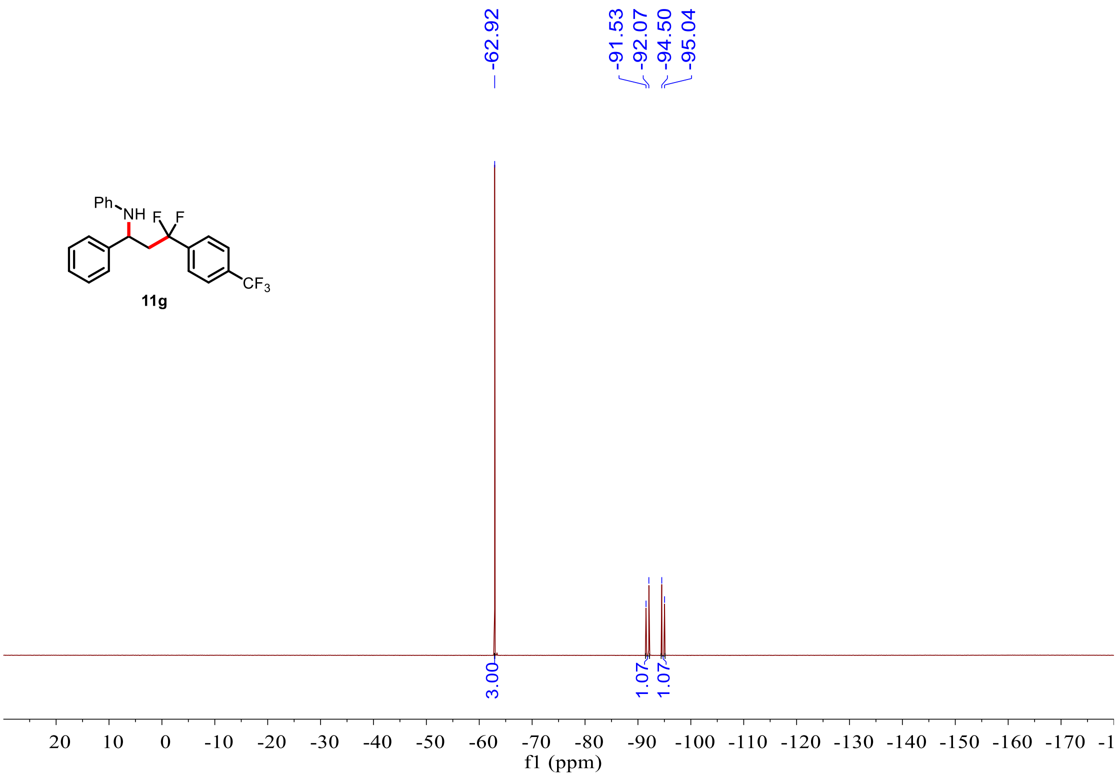

Supplementary Figure 228. ^{19}**F NMR** (471 MHz, $CDCl_3$) spectrum for compound **11g**

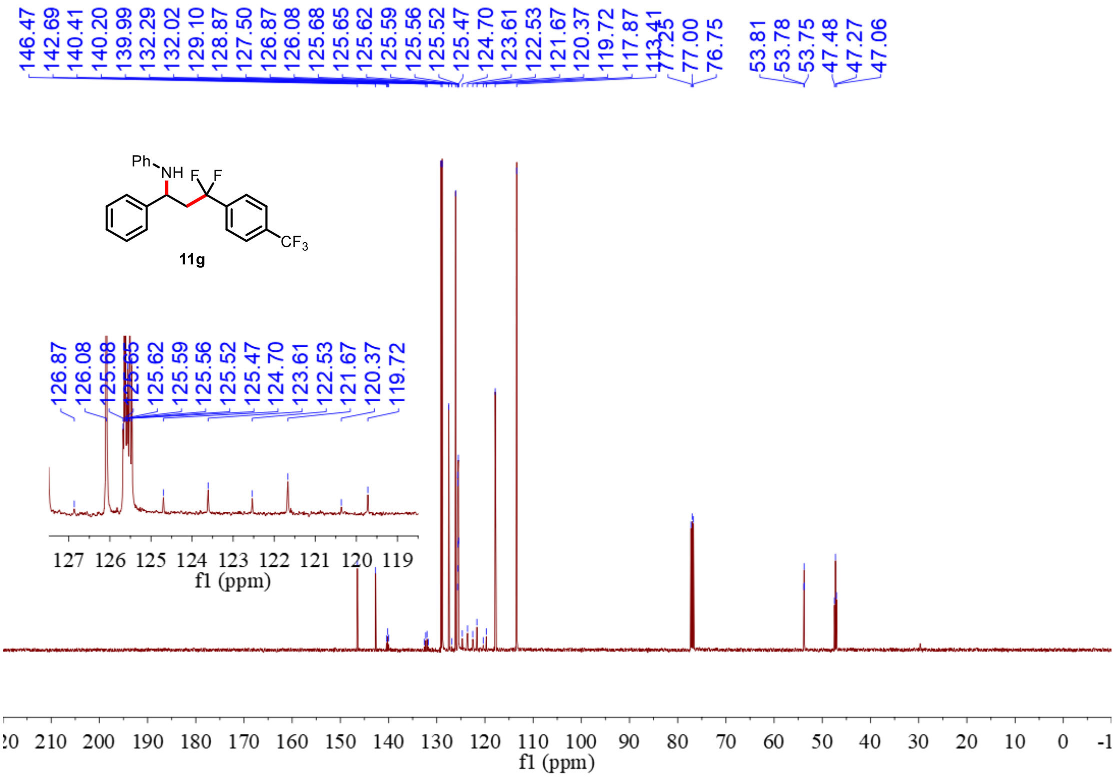

Supplementary Figure 229. ^{13}**C NMR** (126 MHz, $CDCl_3$) spectrum for compound **11g**

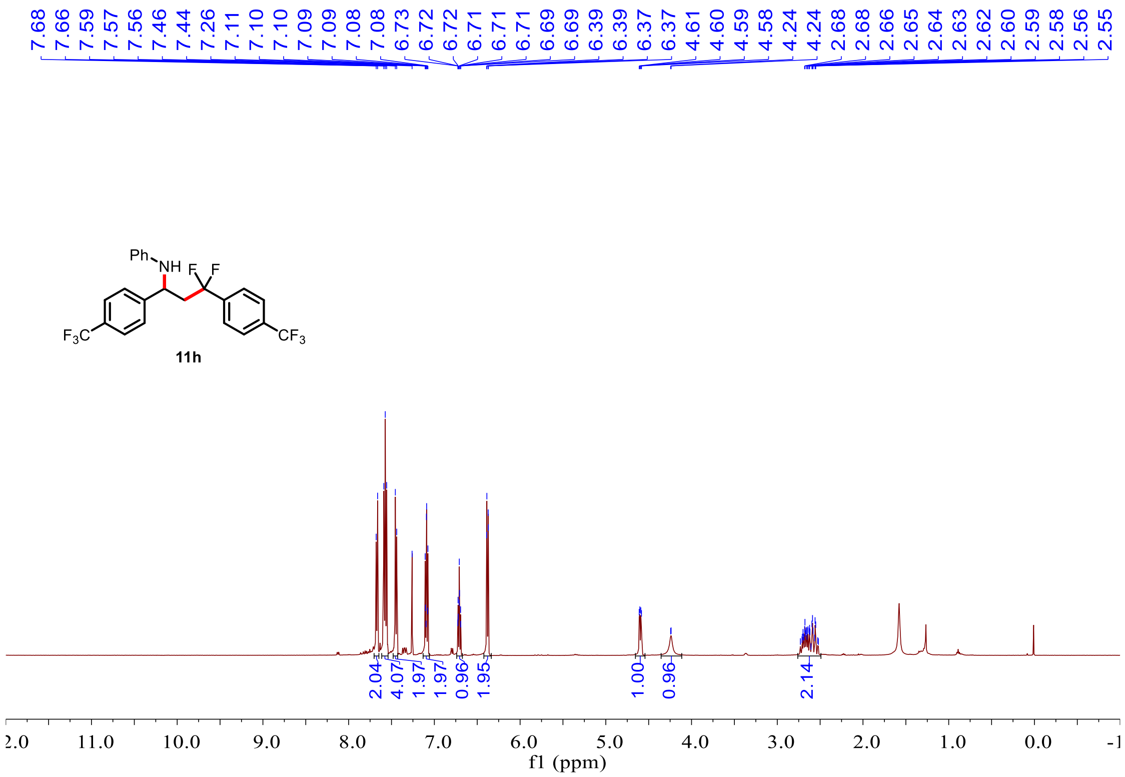

Supplementary Figure 230. ^{1}**H NMR** (500 MHz, $CDCl_3$) spectrum for compound **11h**

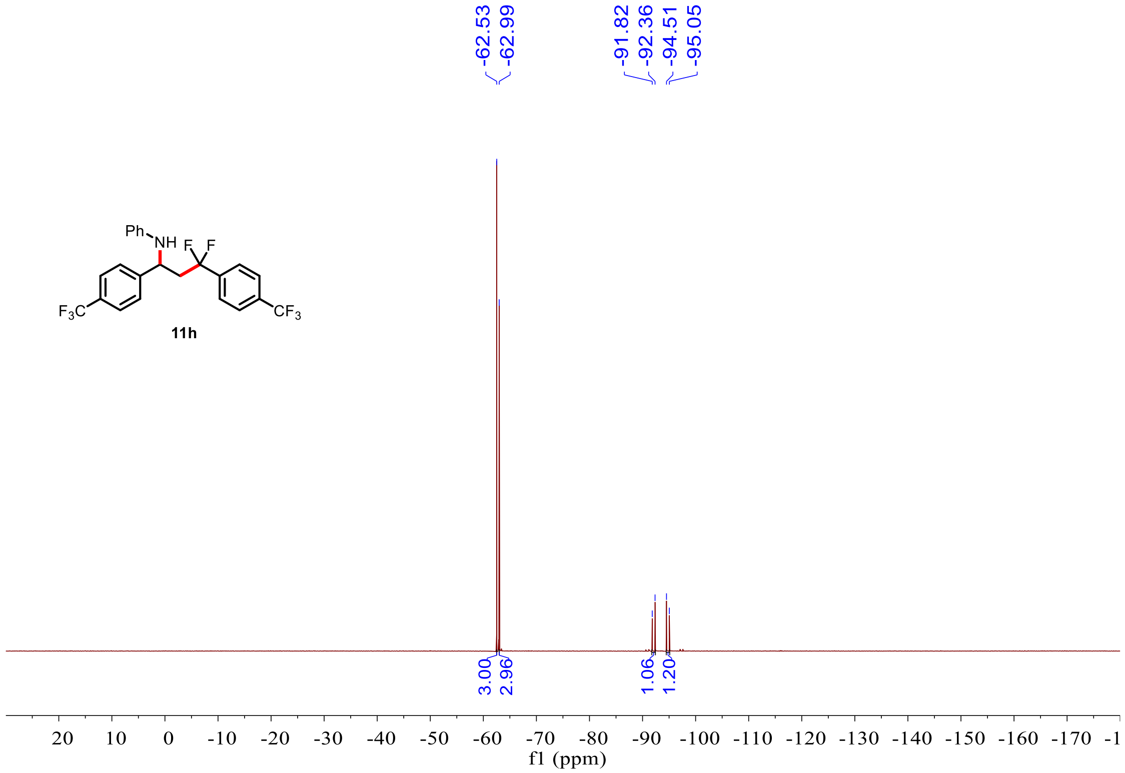

Supplementary Figure 231. ^{19}**F NMR** (471 MHz, $CDCl_3$) spectrum for compound **11h**

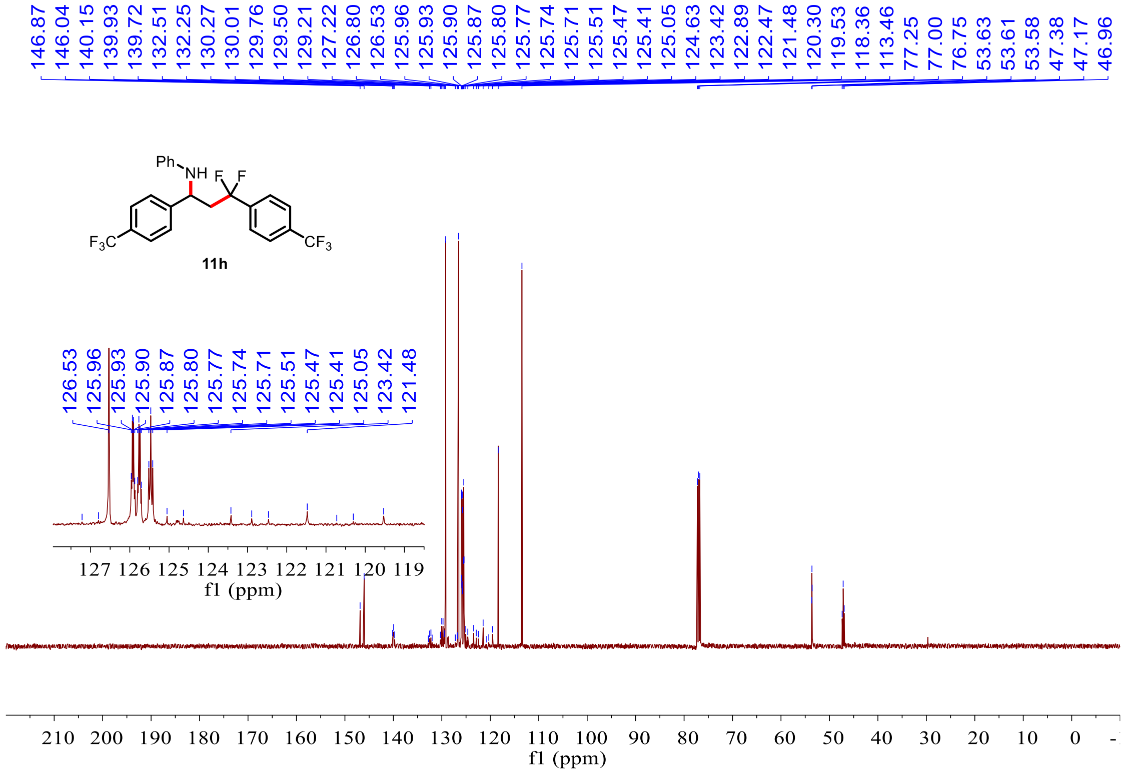

Supplementary Figure 232. ^{13}**C NMR** (126 MHz, $CDCl_3$) spectrum for compound **11h**

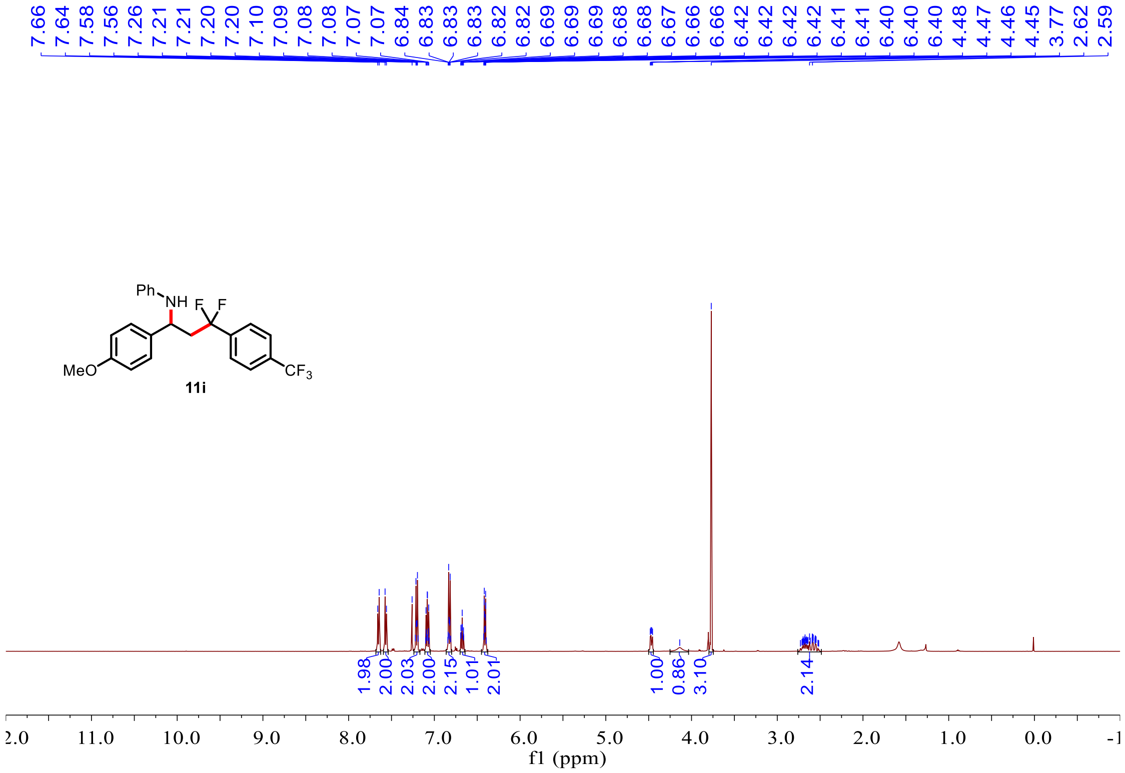

Supplementary Figure 233. ^{1}**H NMR** (500 MHz, $CDCl_3$) spectrum for compound **11i**

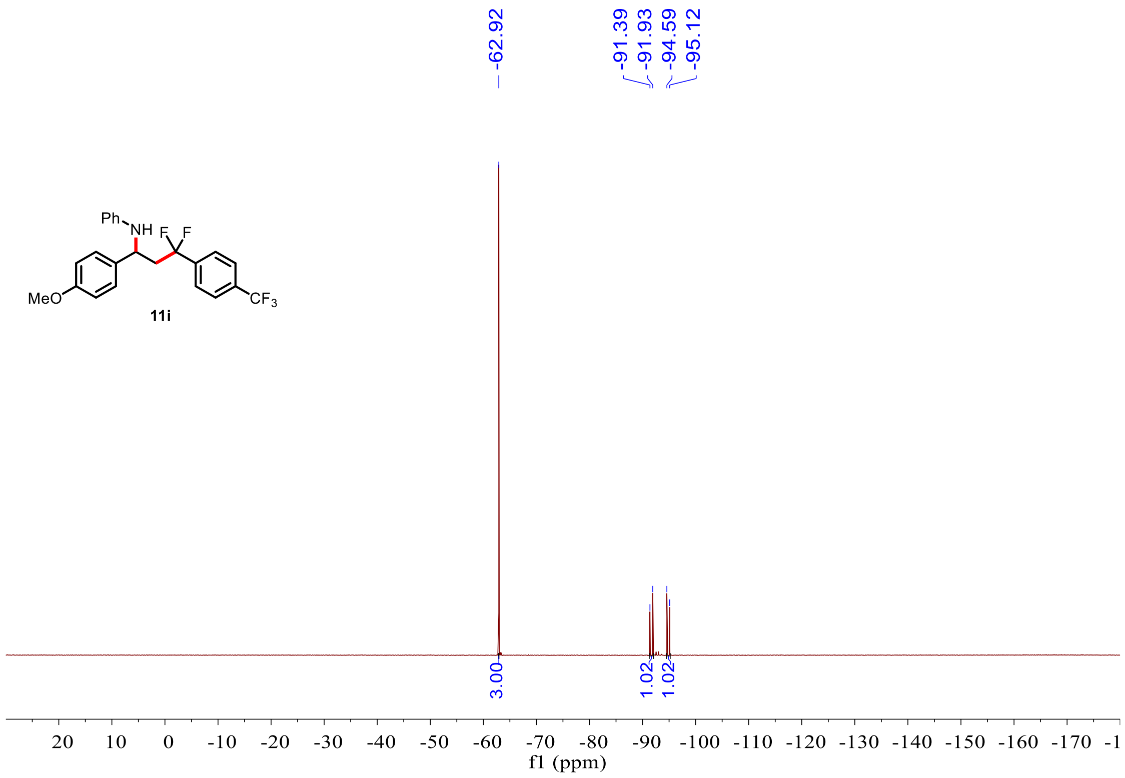

Supplementary Figure 234. ^{19}**F NMR** (471 MHz, $CDCl_3$) spectrum for compound **11i**

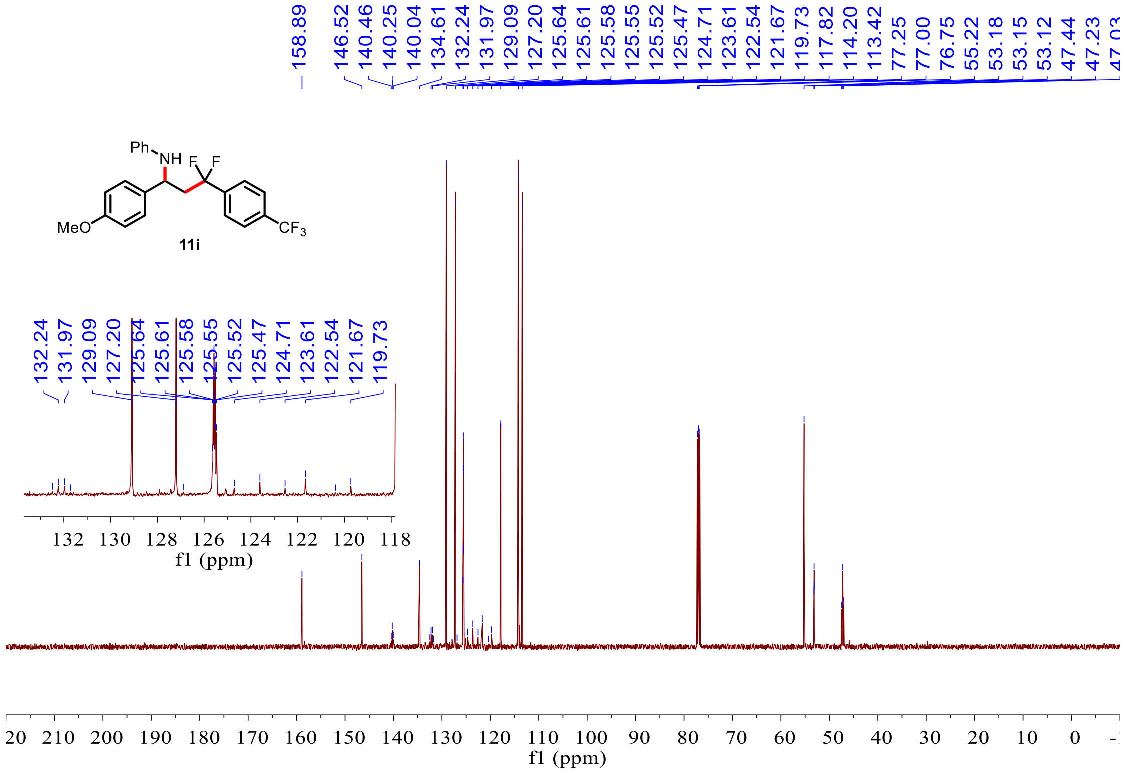

Supplementary Figure 235. ^{13}**C NMR** (126 MHz, $CDCl_3$) spectrum for compound **11i**

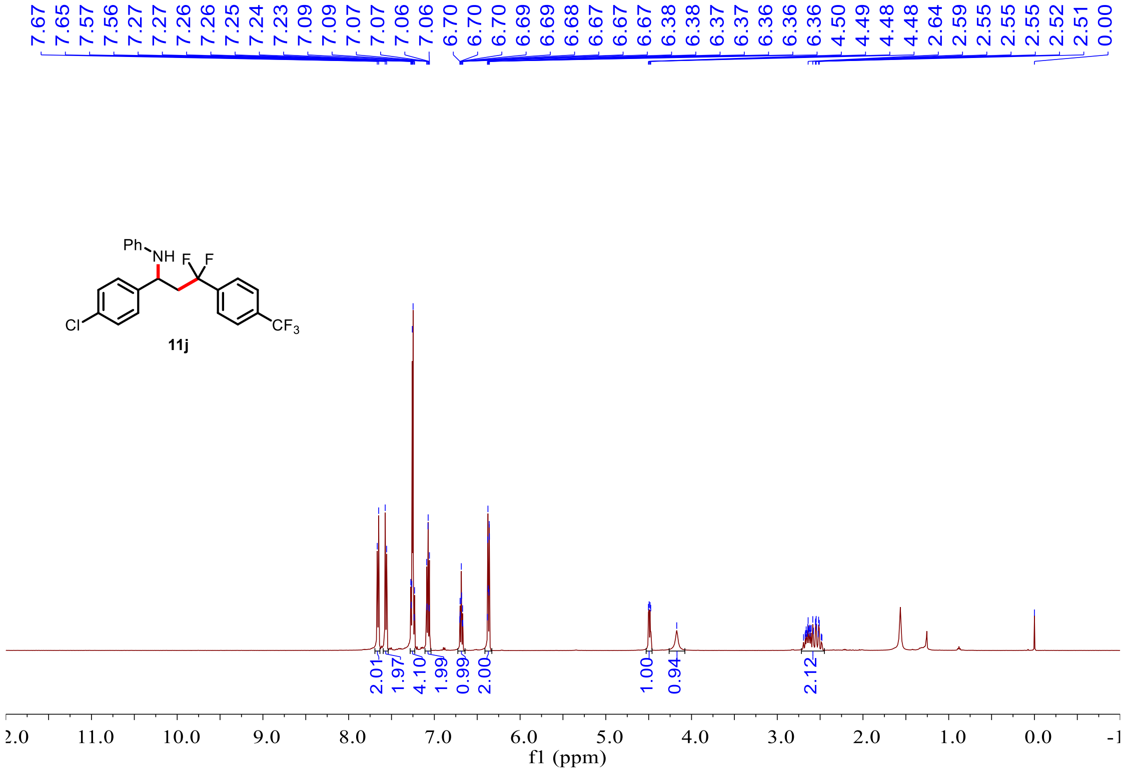

Supplementary Figure 236. ^{1}**H NMR** (500 MHz, $CDCl_3$) spectrum for compound **11j**

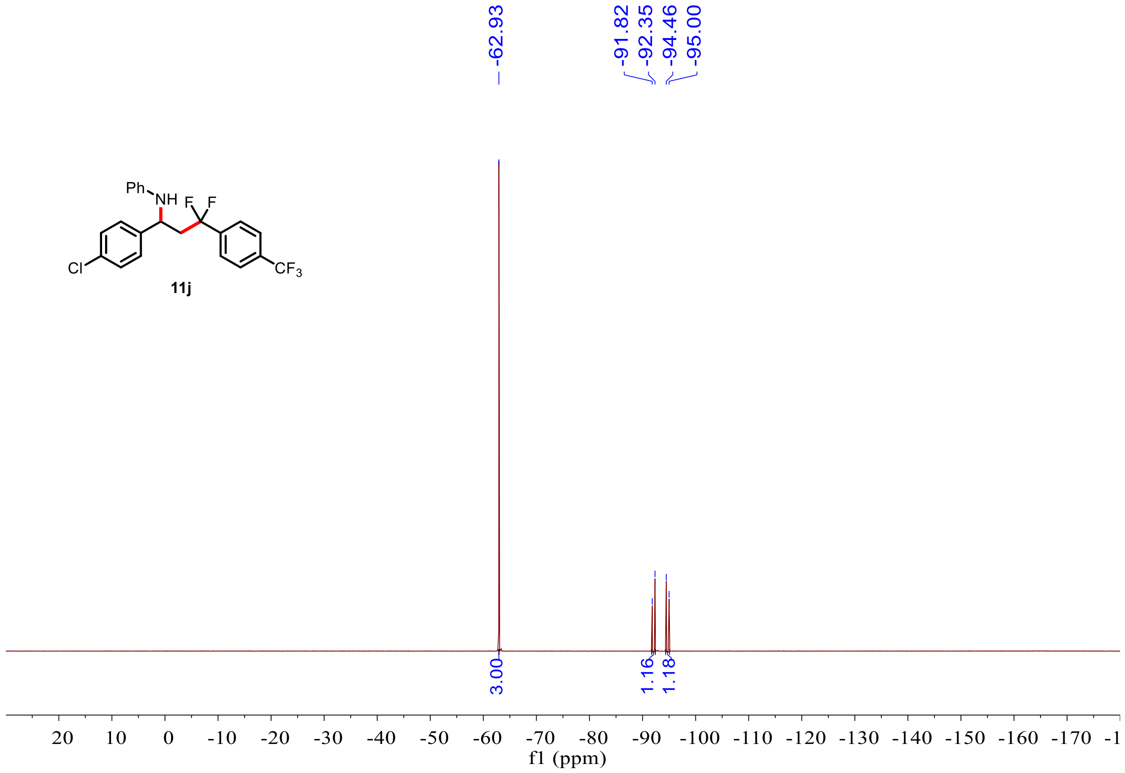

Supplementary Figure 237. ^{19}**F NMR** (471 MHz, $CDCl_3$) spectrum for compound **11j**

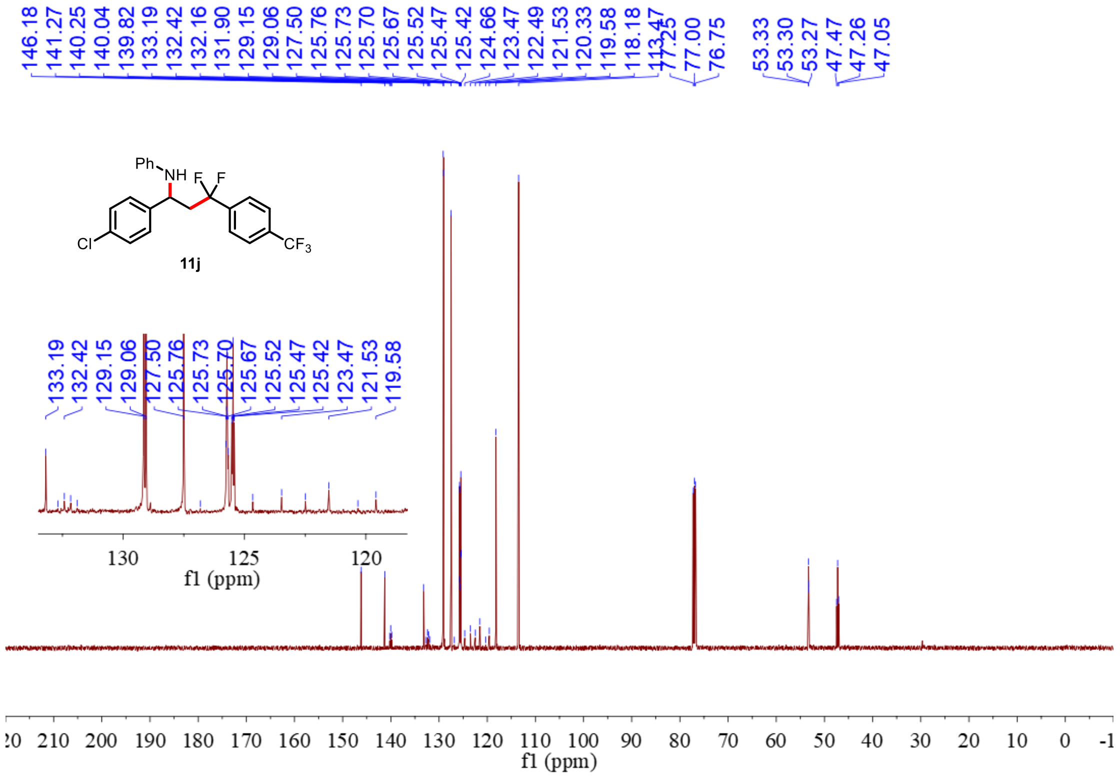

Supplementary Figure 238. ^{13}**C NMR** (126 MHz, $CDCl_3$) spectrum for compound **11j**

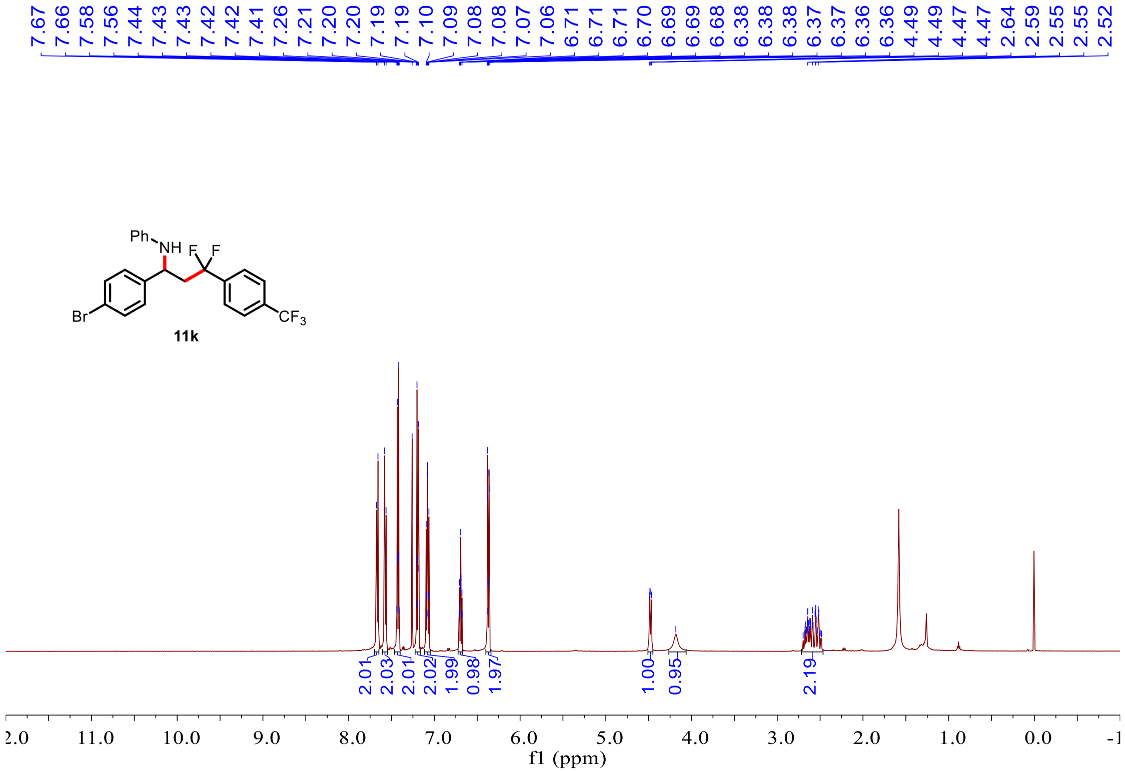

Supplementary Figure 239. ^{1}**H NMR** (500 MHz, $CDCl_3$) spectrum for compound **11k**

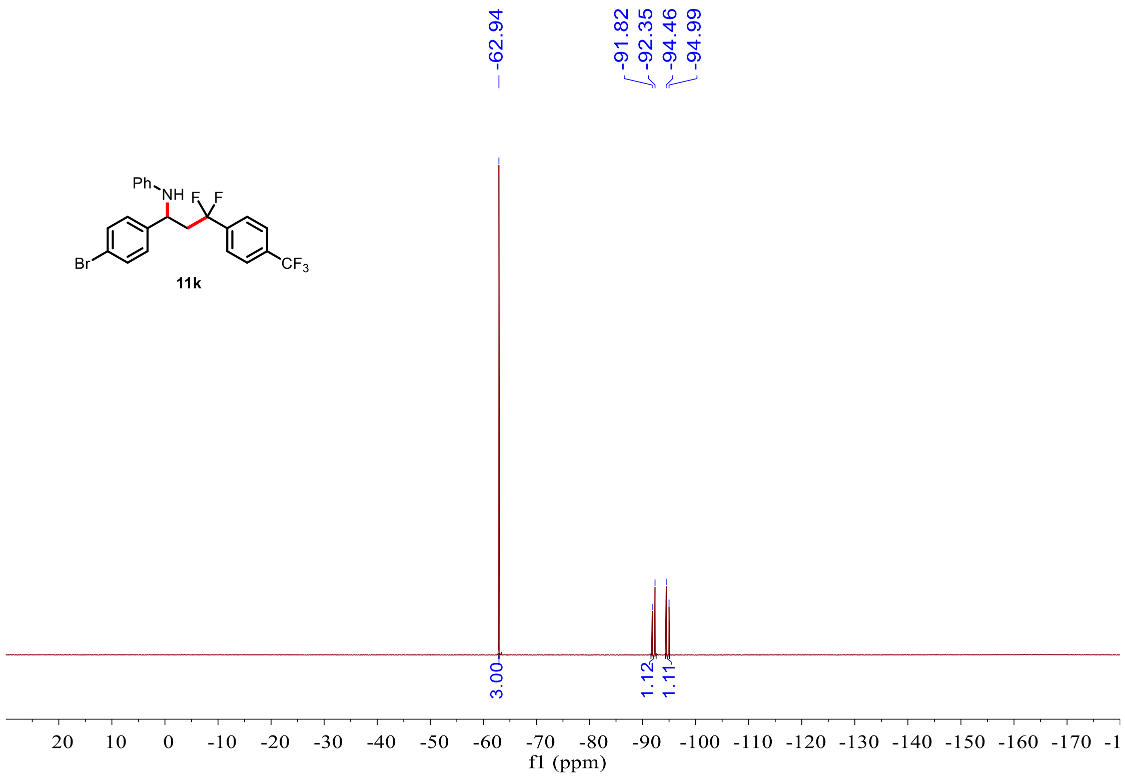

Supplementary Figure 240. ^{19}F NMR (471 MHz, $CDCl_3$) spectrum for compound **11k**

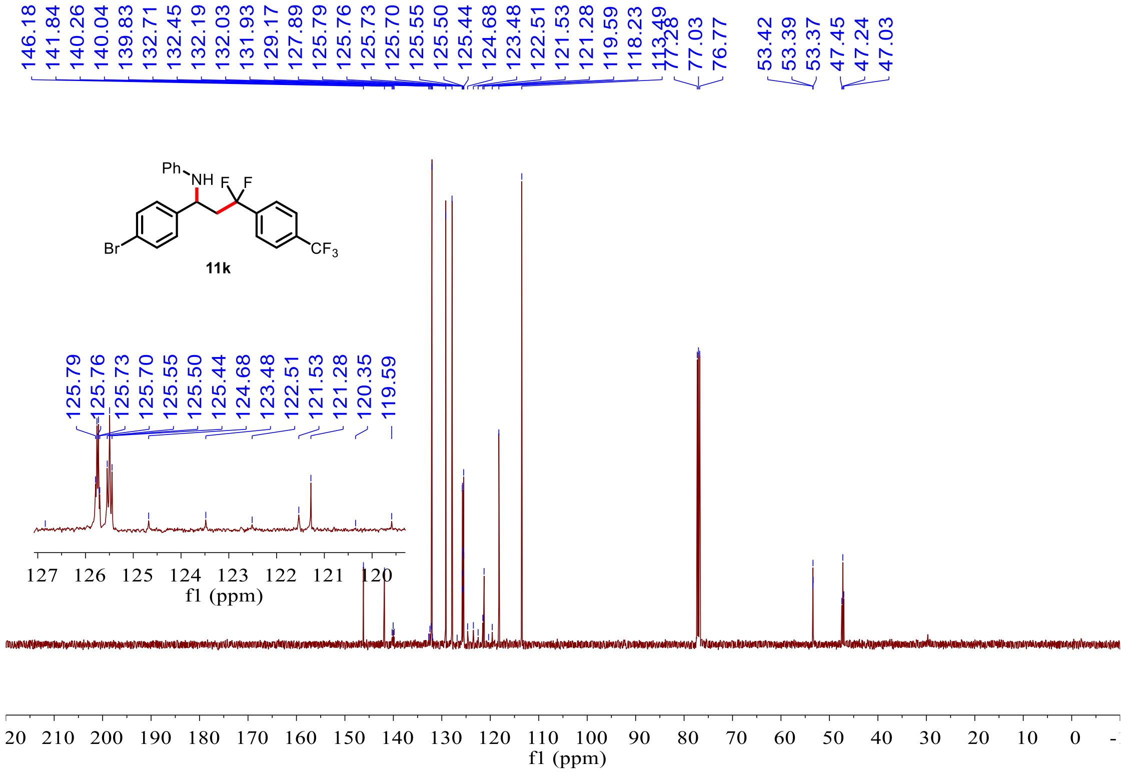

Supplementary Figure 241. ^{13}C NMR (126 MHz, $CDCl_3$) spectrum for compound **11k**

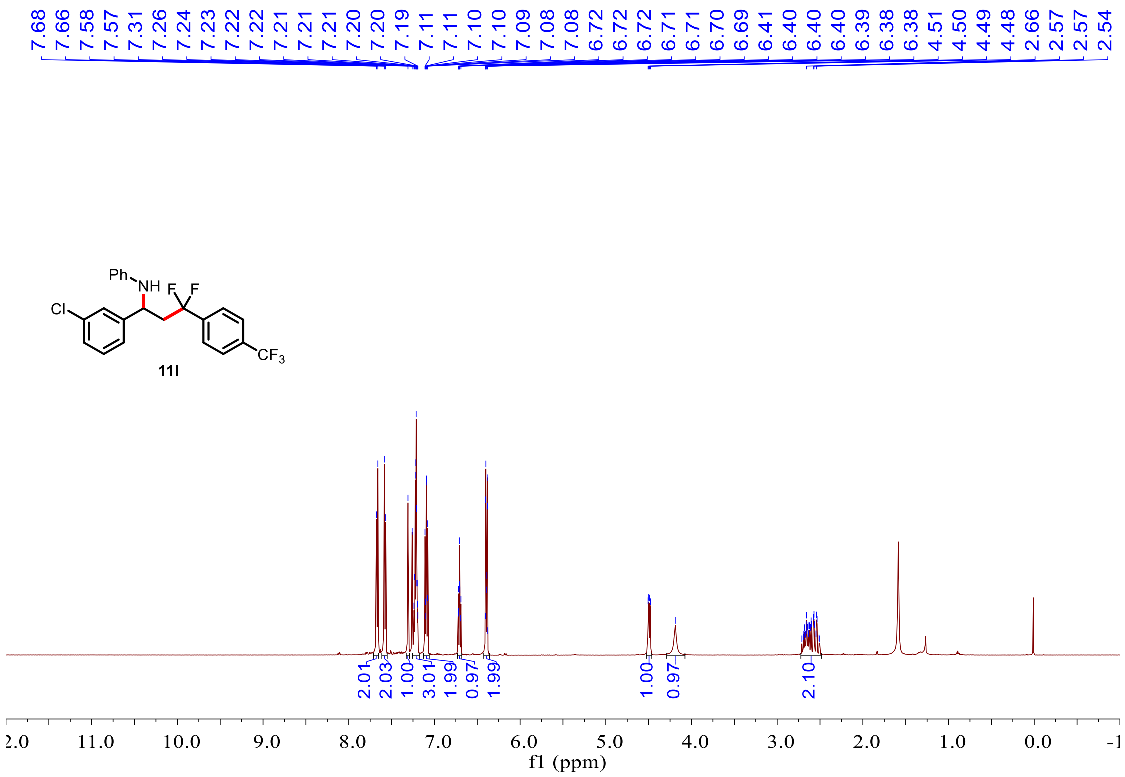

Supplementary Figure 242. ^{1}**H NMR** (500 MHz, $CDCl_3$) spectrum for compound **11l**

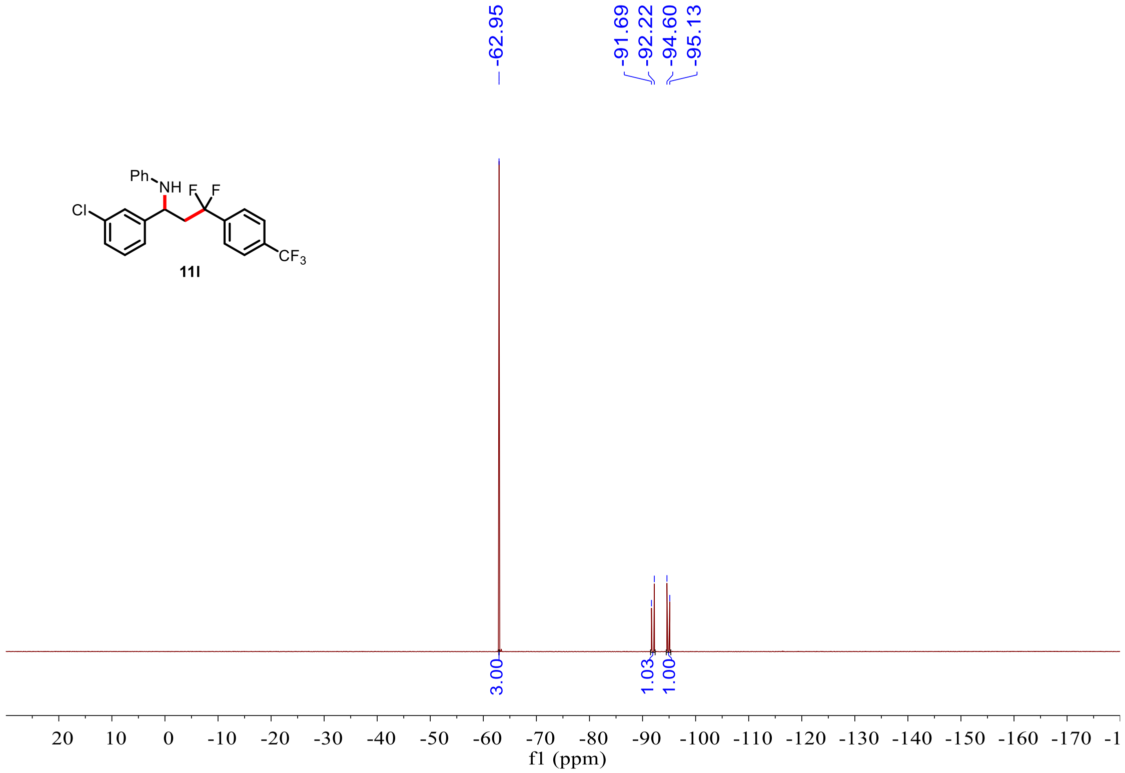

Supplementary Figure 243. ^{19}**F NMR** (471 MHz, $CDCl_3$) spectrum for compound **11l**

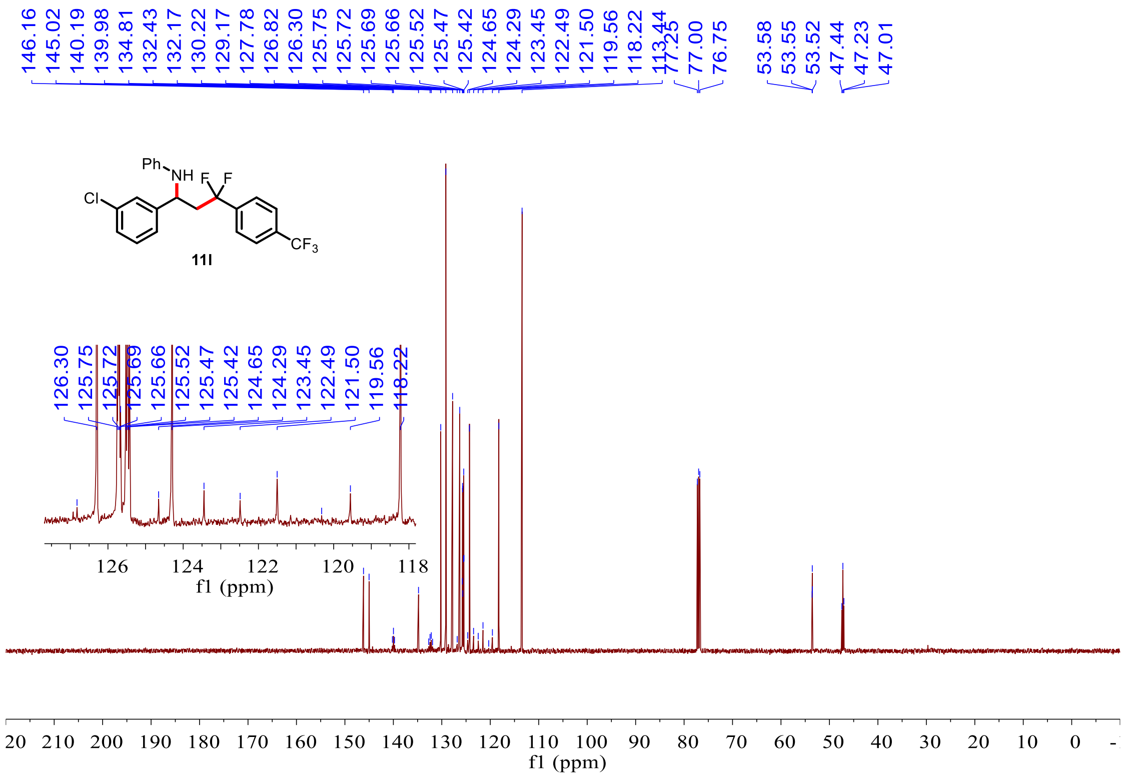

Supplementary Figure 244. ^{13}C NMR (126 MHz, $CDCl_3$) spectrum for compound **11l**

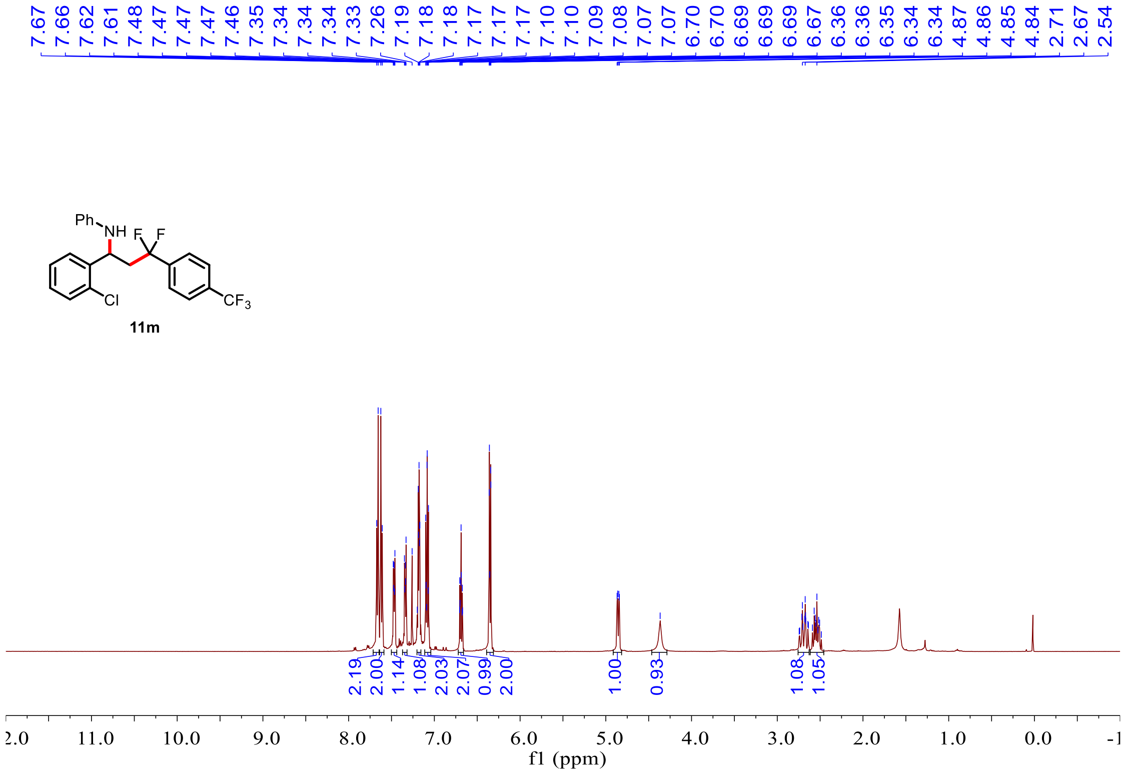

Supplementary Figure 245. ^{1}H NMR (500 MHz, $CDCl_3$) spectrum for compound **11m**

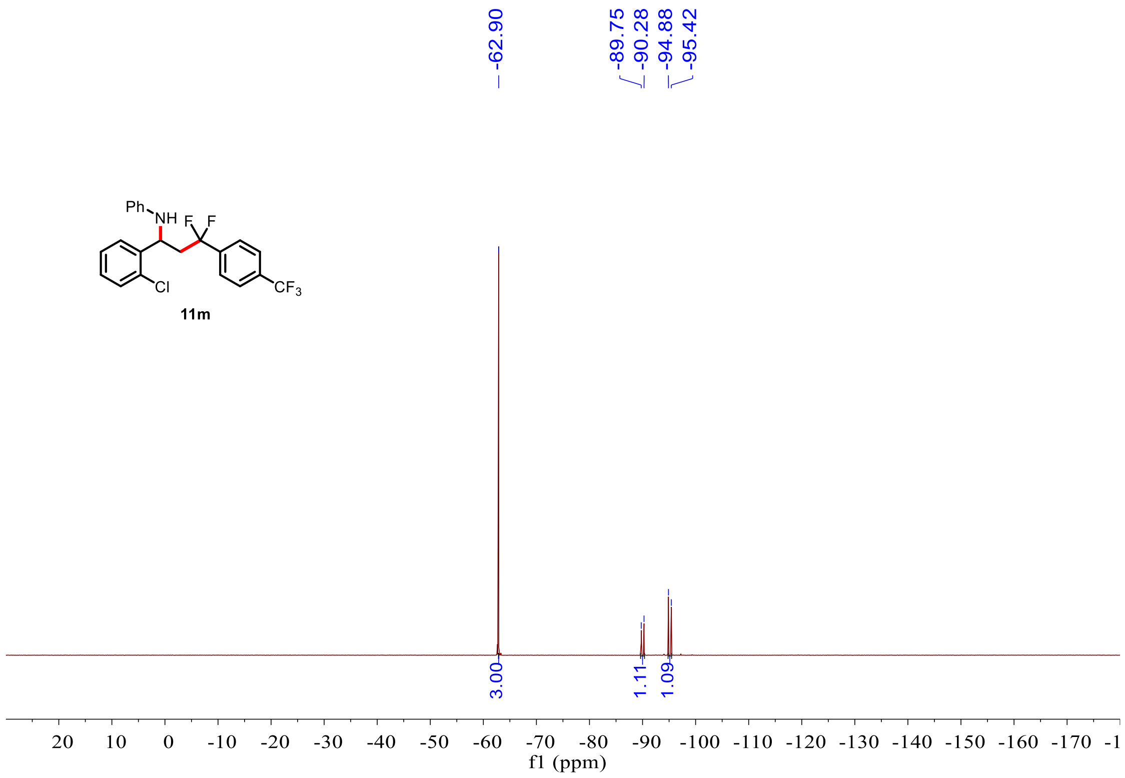

Supplementary Figure 246. ^{19}**F NMR** (471 MHz, $CDCl_3$) spectrum for compound **11m**

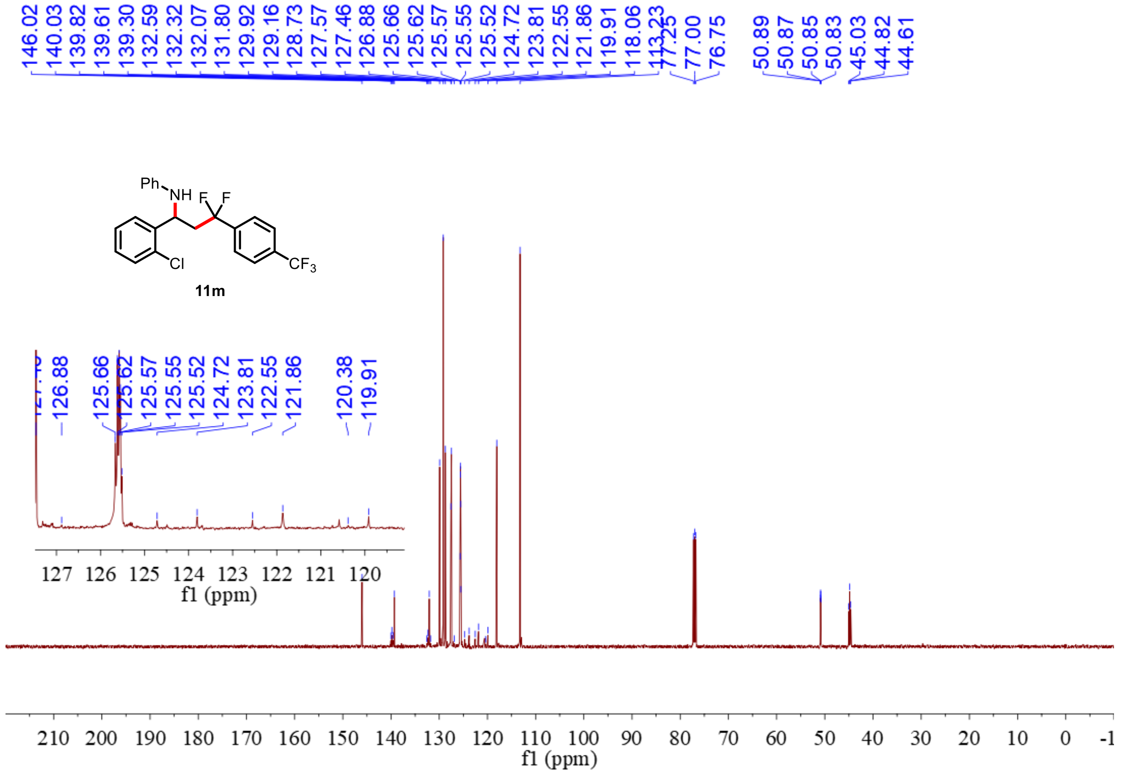

Supplementary Figure 247. ^{13}**C NMR** (126 MHz, $CDCl_3$) spectrum for compound **11m**

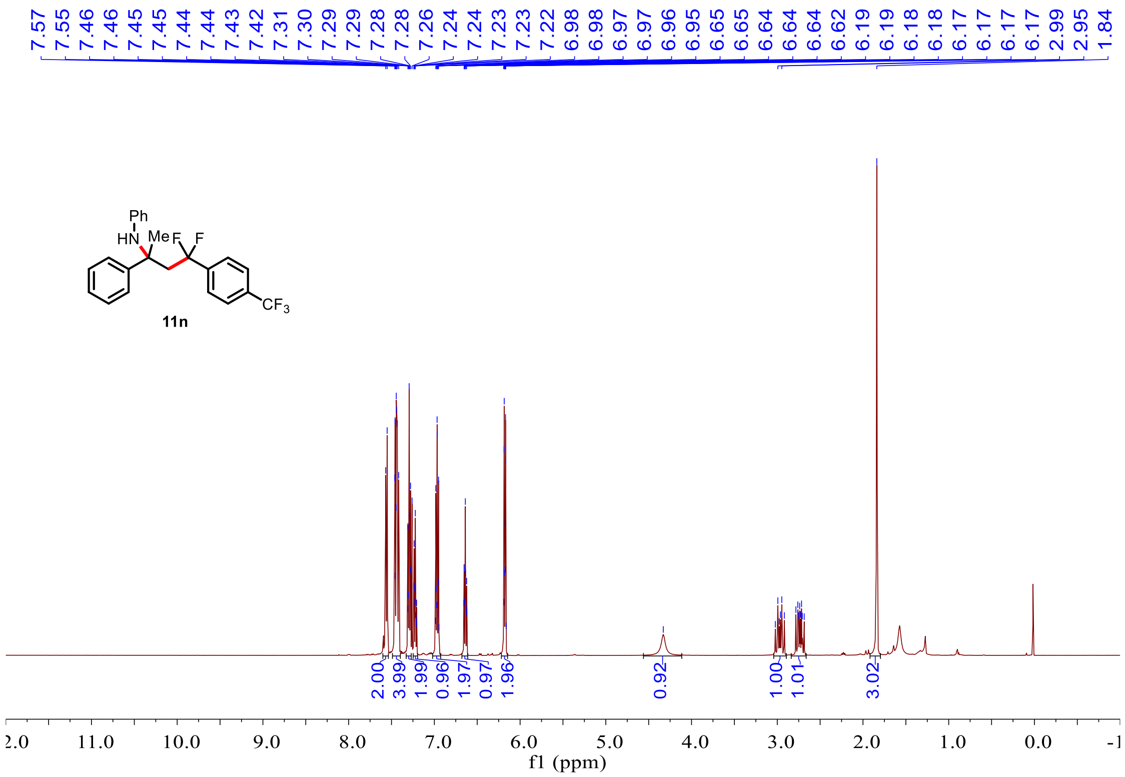

Supplementary Figure 248. ^{1}**H NMR** (500 MHz, $CDCl_3$) spectrum for compound **11n**

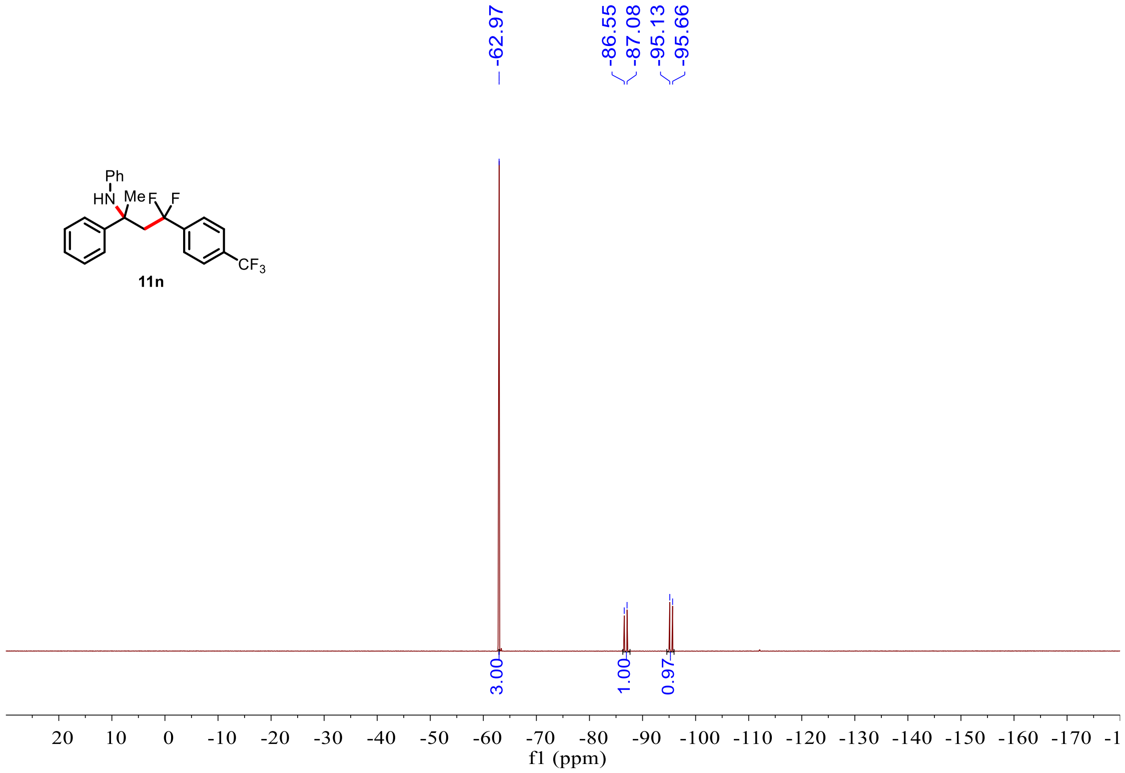

Supplementary Figure 249. ^{19}**F NMR** (471 MHz, $CDCl_3$) spectrum for compound **11n**

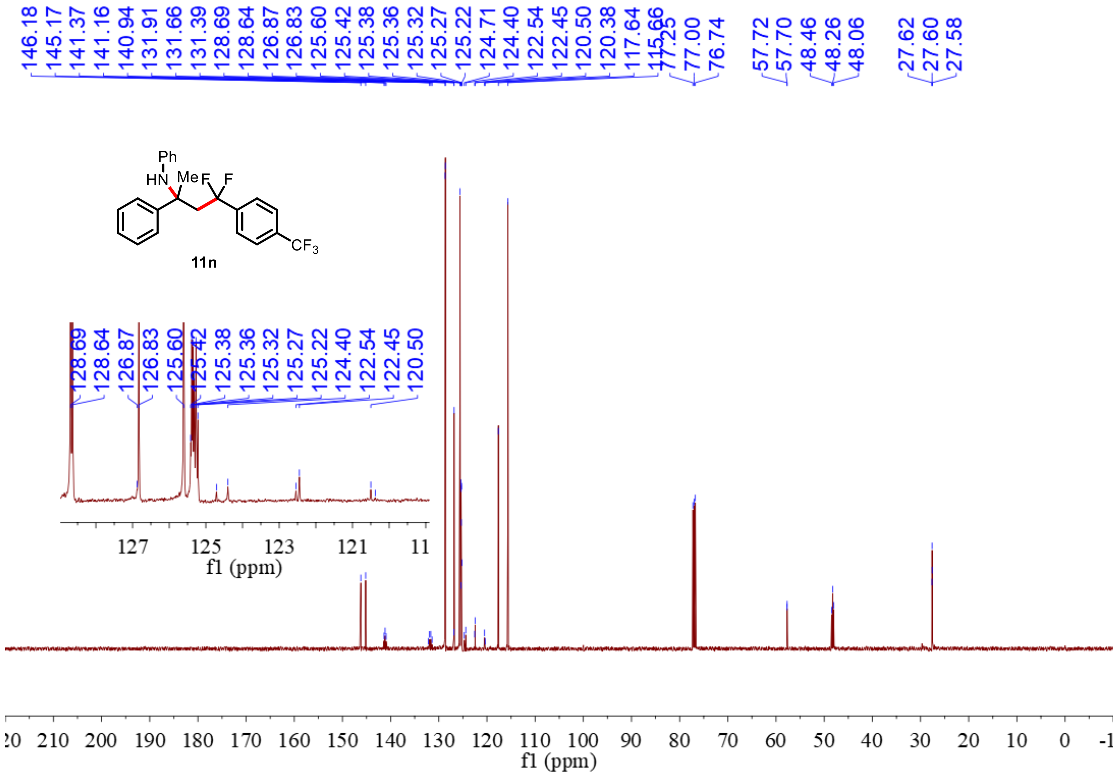

Supplementary Figure 250. ^{13}C NMR (126 MHz, $CDCl_3$) spectrum for compound **11n**

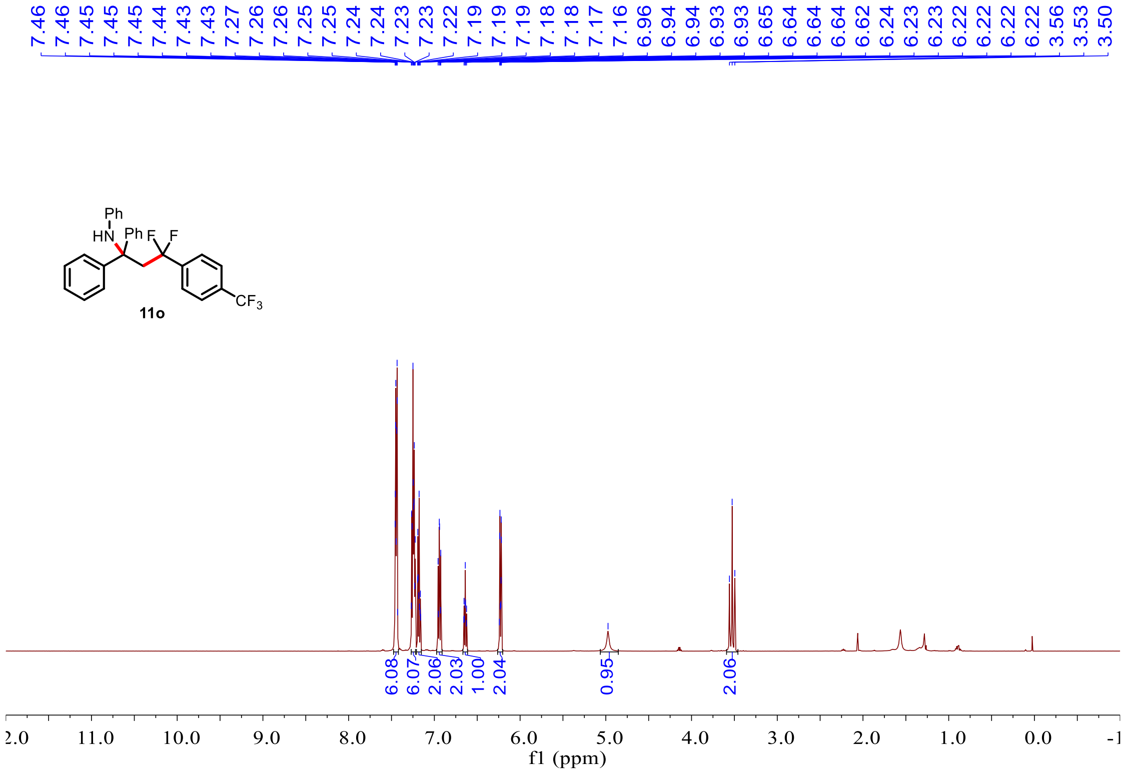

Supplementary Figure 251. ^{1}H NMR (500 MHz, $CDCl_3$) spectrum for compound **11o**

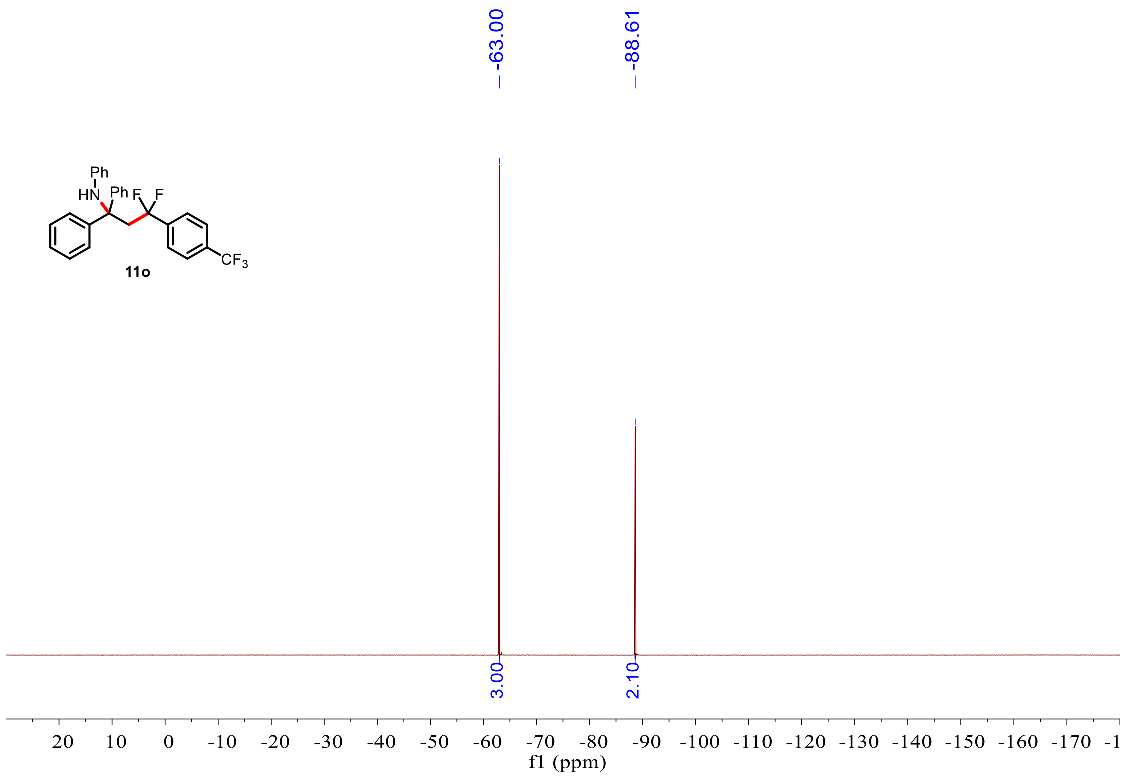

Supplementary Figure 252. ^{19}**F NMR** (471 MHz, $CDCl_3$) spectrum for compound **11o**

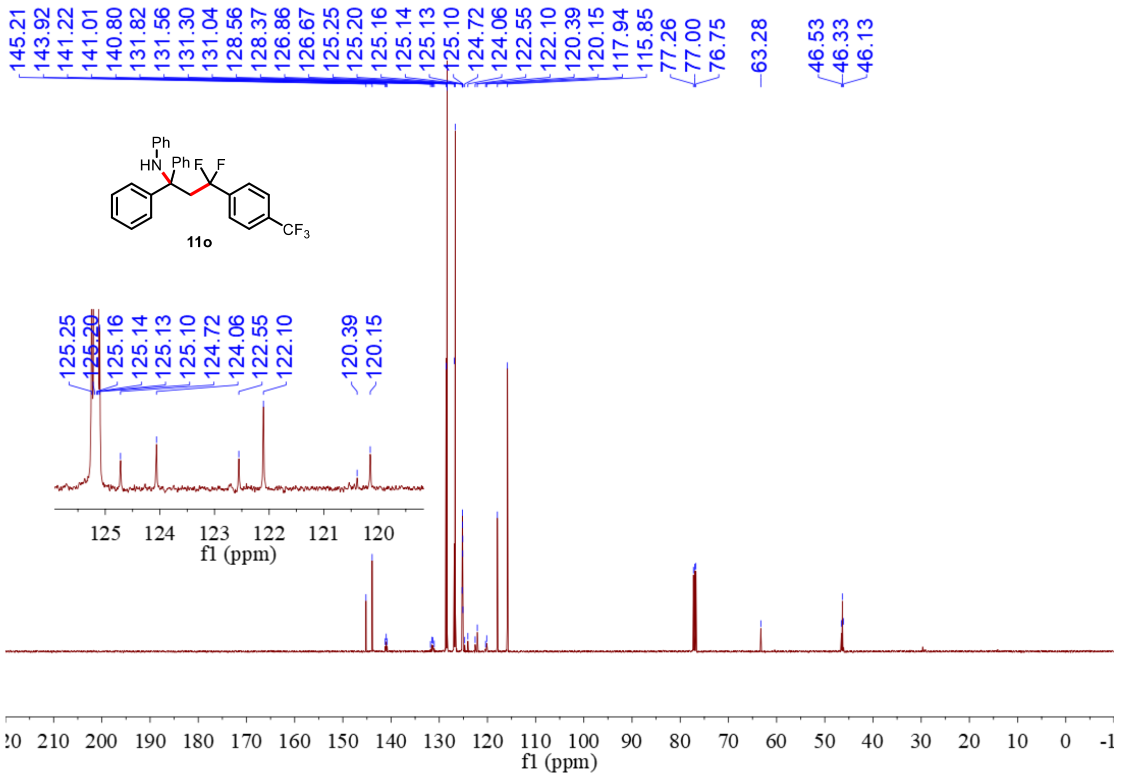

Supplementary Figure 253. ^{13}**C NMR** (126 MHz, $CDCl_3$) spectrum for compound **11o**

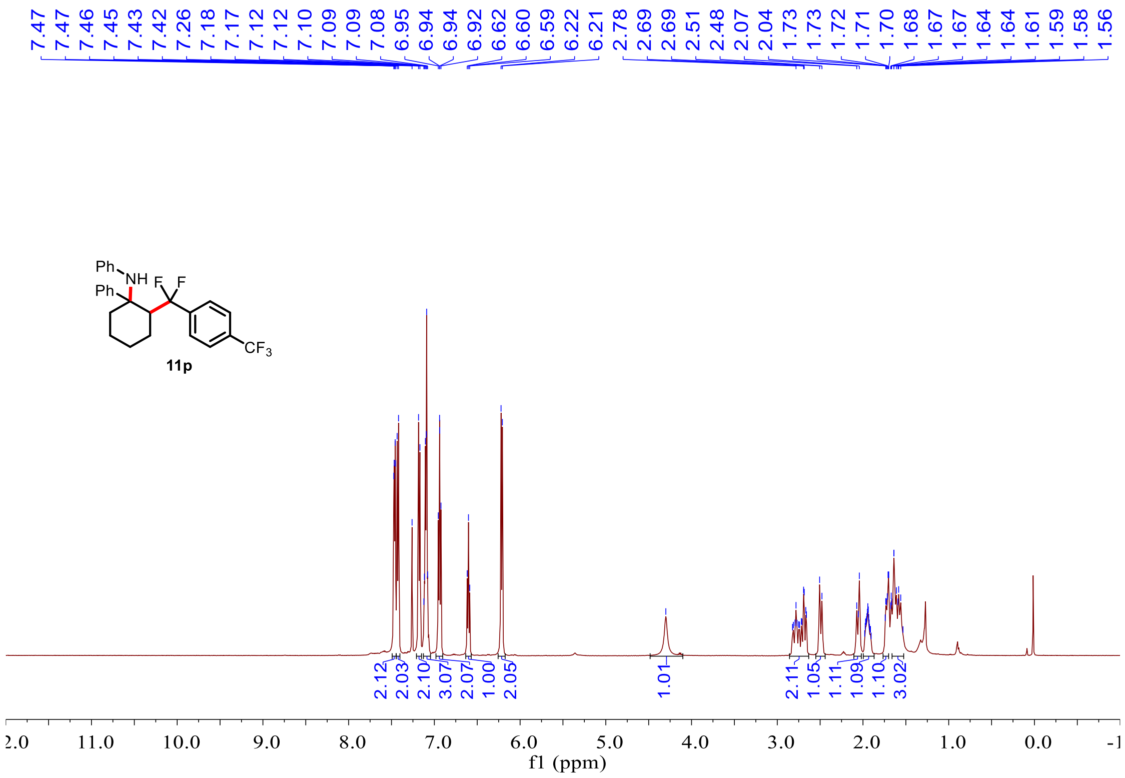

Supplementary Figure 254. ^{1}**H NMR** (500 MHz, $CDCl_3$) spectrum for compound **11p**

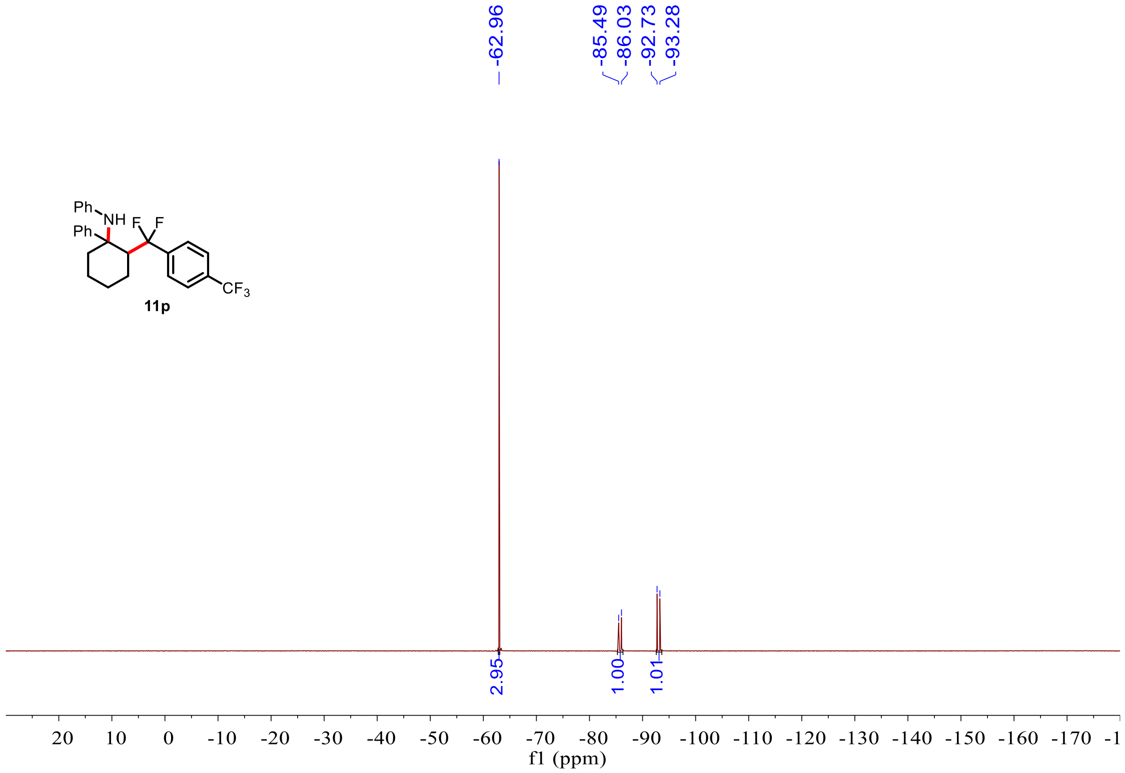

Supplementary Figure 255. ^{19}**F NMR** (471 MHz, $CDCl_3$) spectrum for compound **11p**

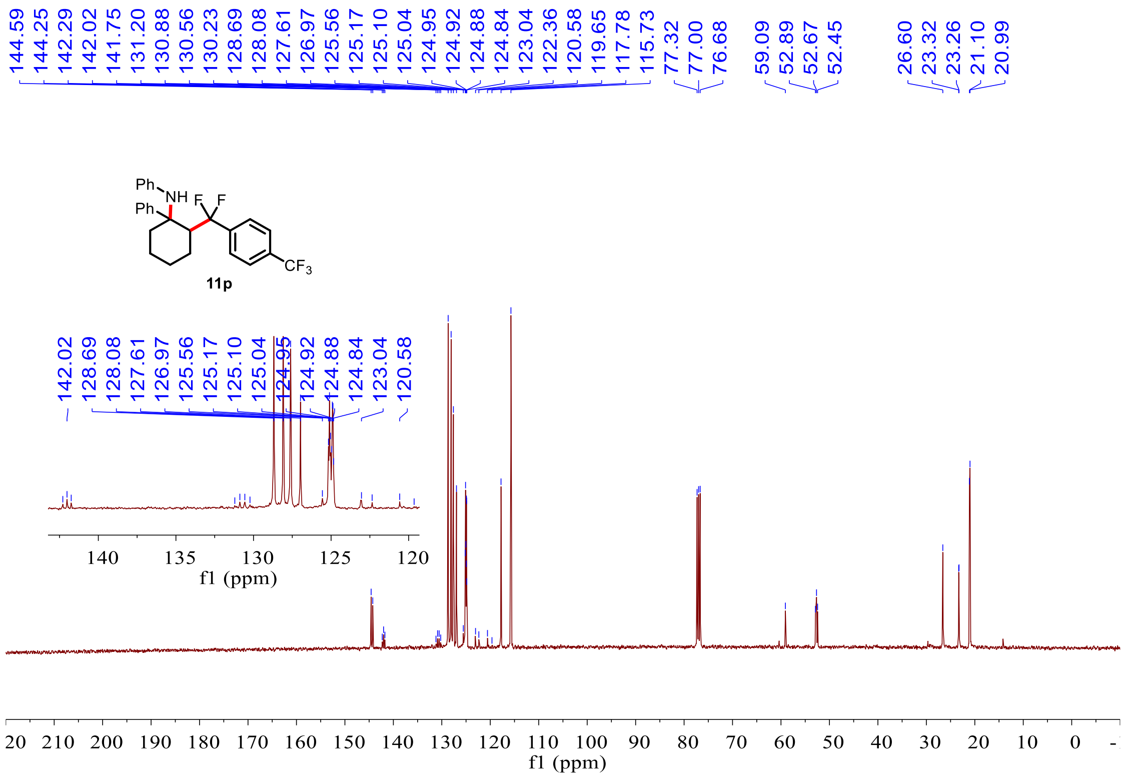

Supplementary Figure 256. ^{13}**C NMR** (101 MHz, $CDCl_3$) spectrum for compound **11p**

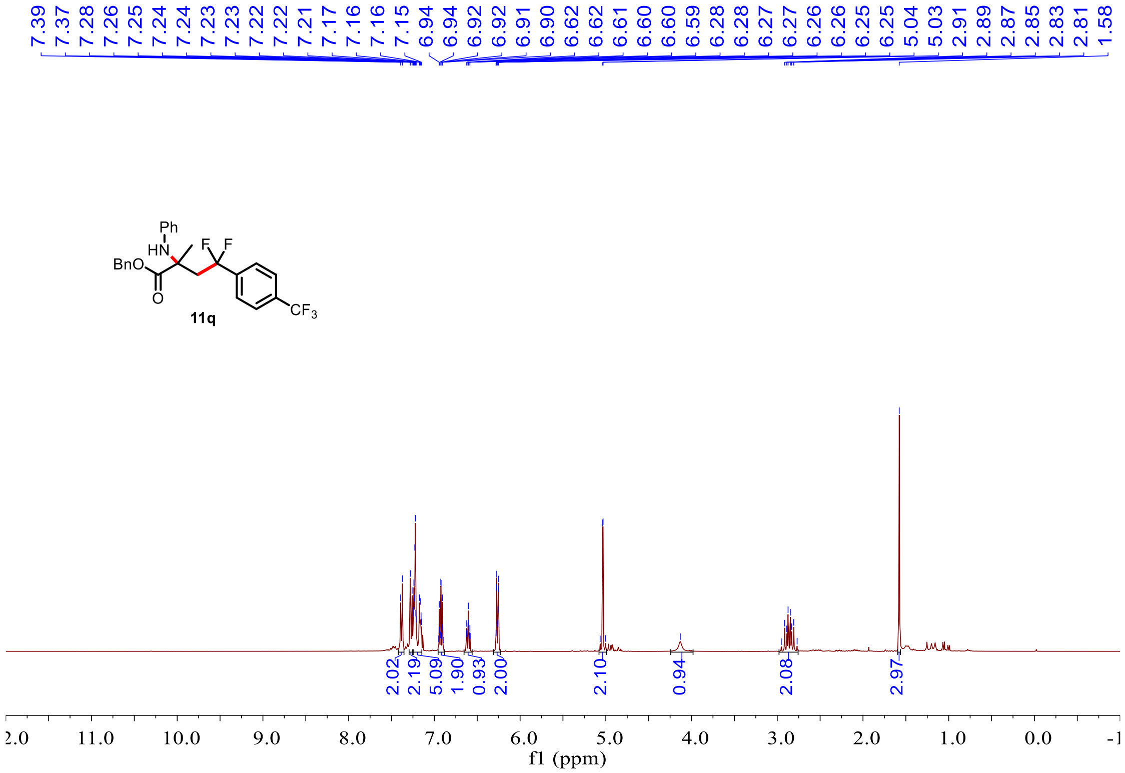

Supplementary Figure 257. ^{1}**H NMR** (400 MHz, $CDCl_3$) spectrum for compound **11q**

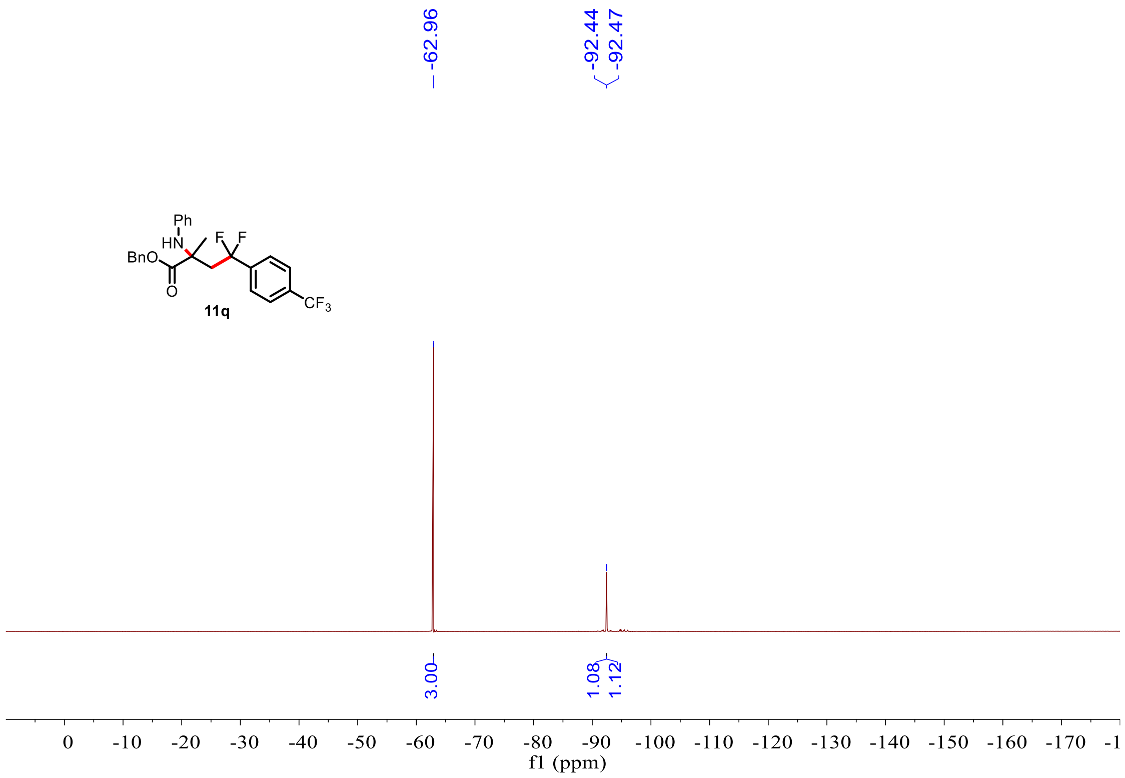

Supplementary Figure 258. **^{19}F NMR** (376 MHz, $CDCl_3$) spectrum for compound **11q**

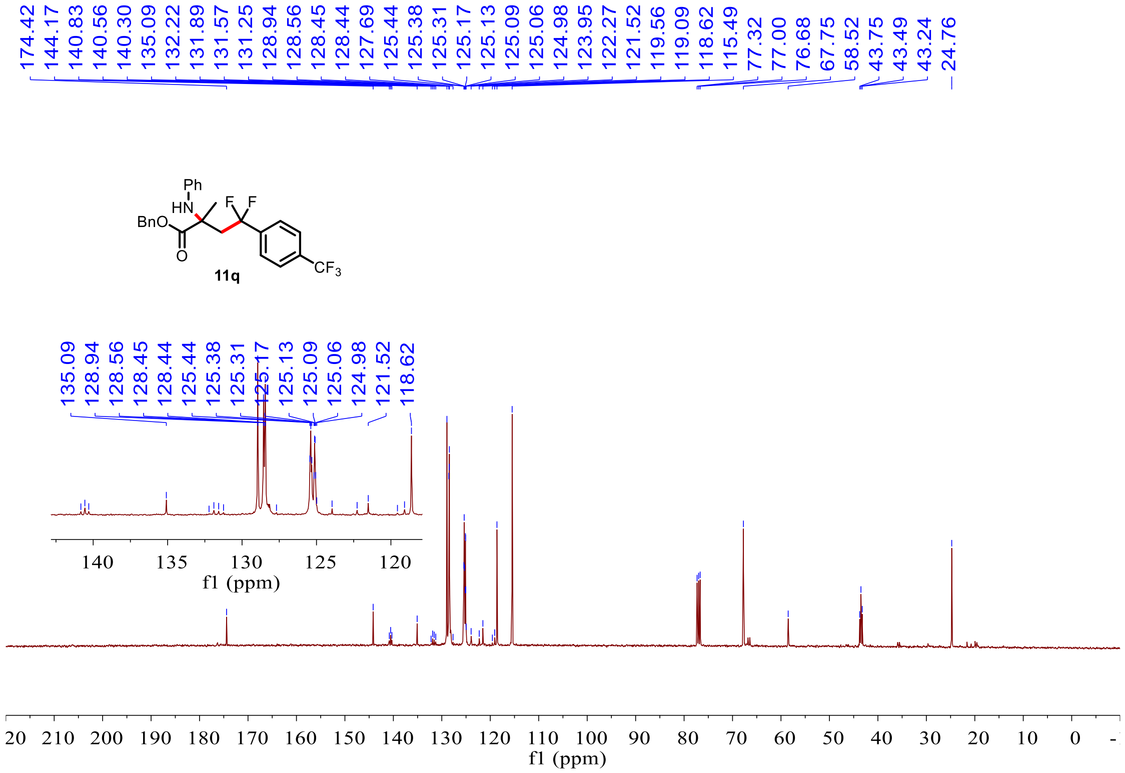

Supplementary Figure 259. **^{13}C NMR** (101 MHz, $CDCl_3$) spectrum for compound **11q**

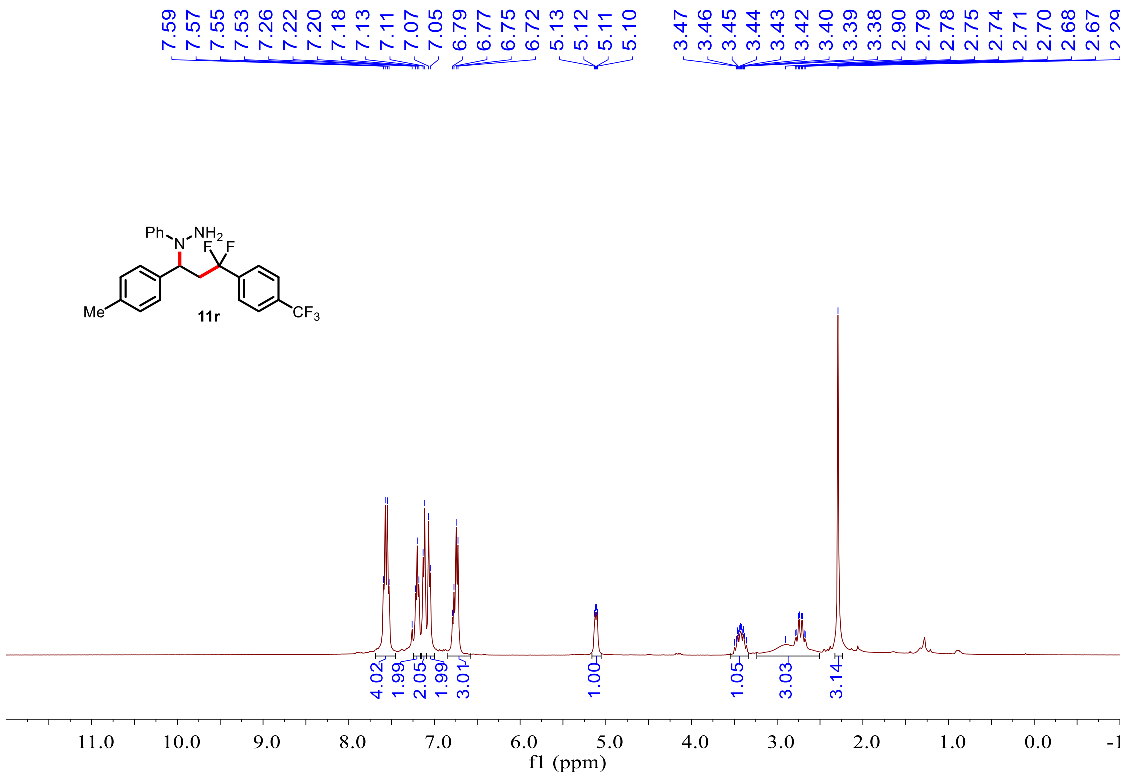

Supplementary Figure 260. ^{1}**H NMR** (400 MHz, $CDCl_3$) spectrum for compound **11r**

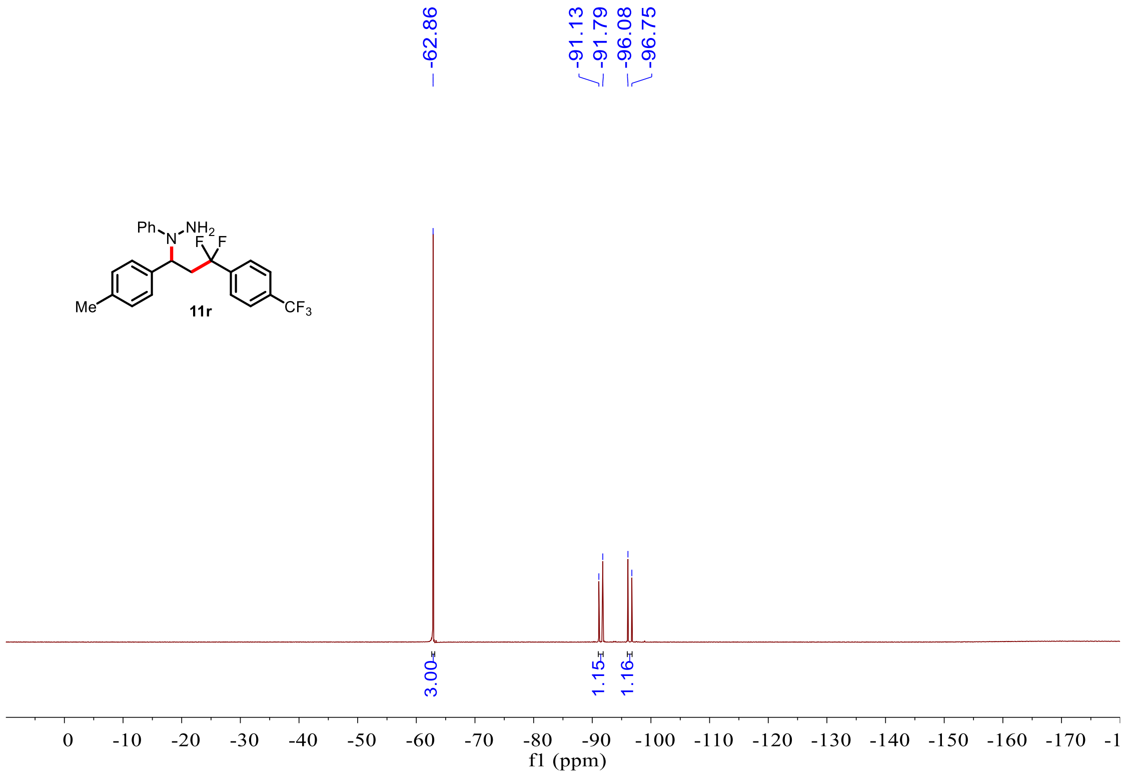

Supplementary Figure 261. ^{19}**F NMR** (376 MHz, $CDCl_3$) spectrum for compound **11r**

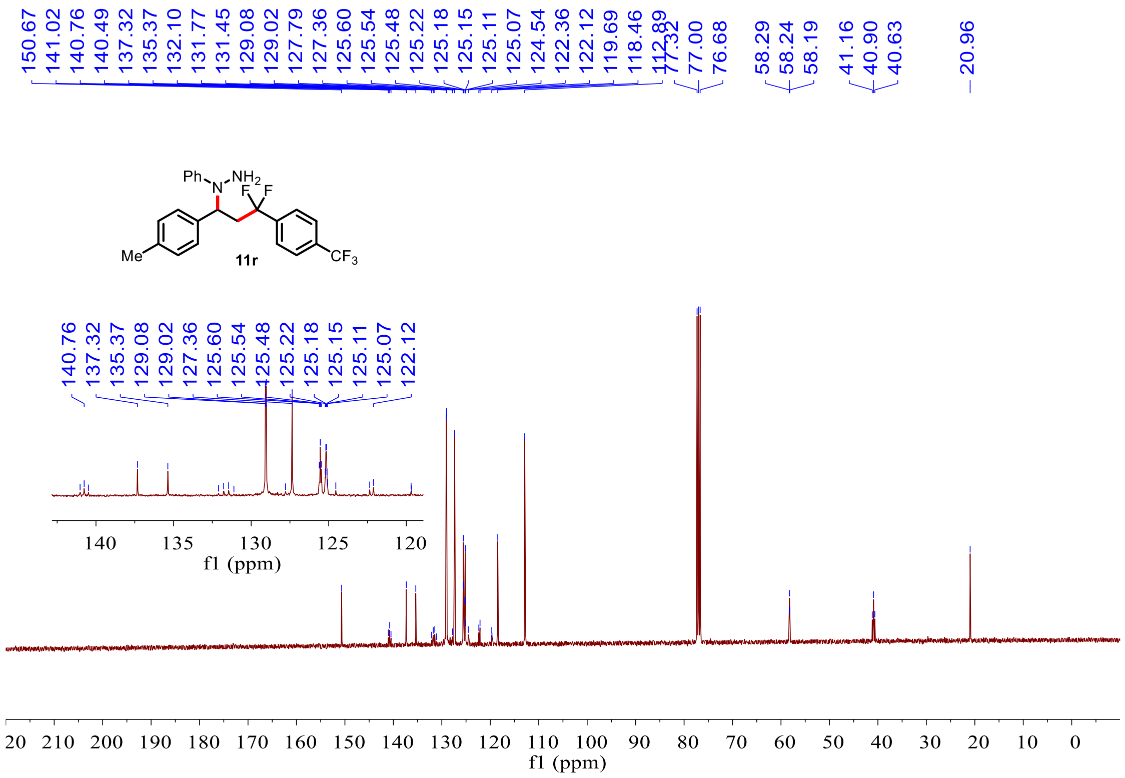

Supplementary Figure 262. ^{13}**C NMR** (101 MHz, $CDCl_3$) spectrum for compound **11r**

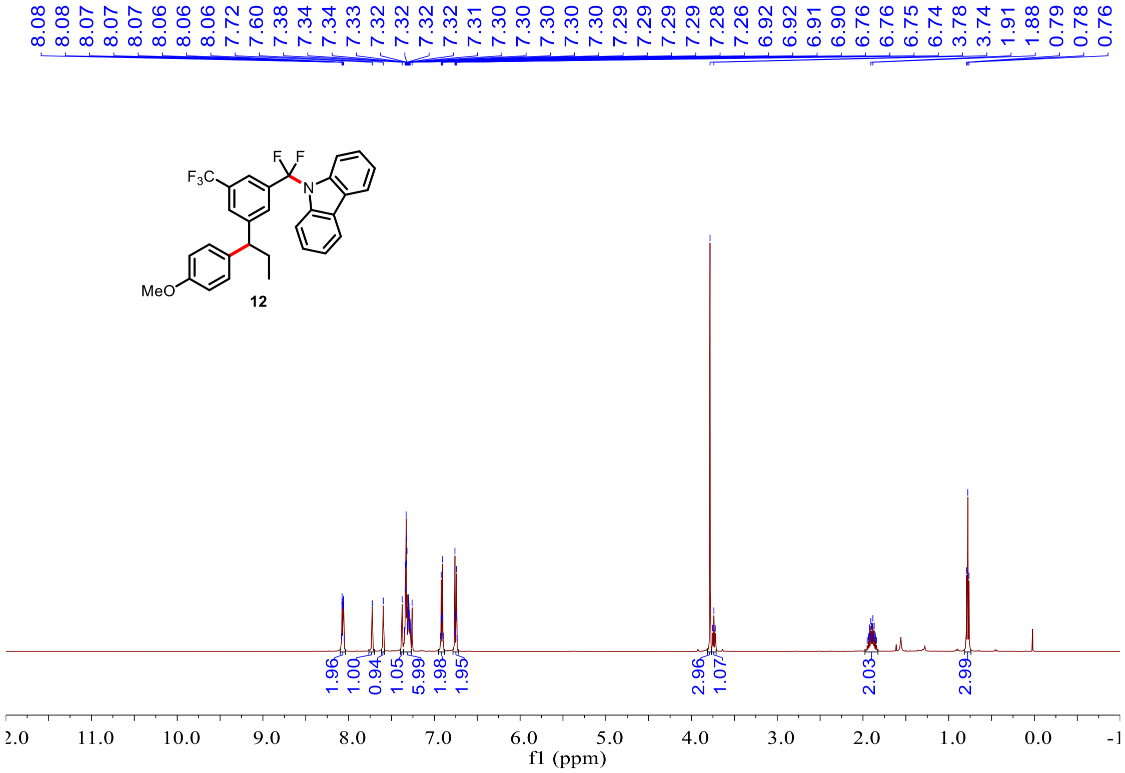

Supplementary Figure 263. ^{1}**H NMR** (500 MHz, $CDCl_3$) spectrum for compound **12**

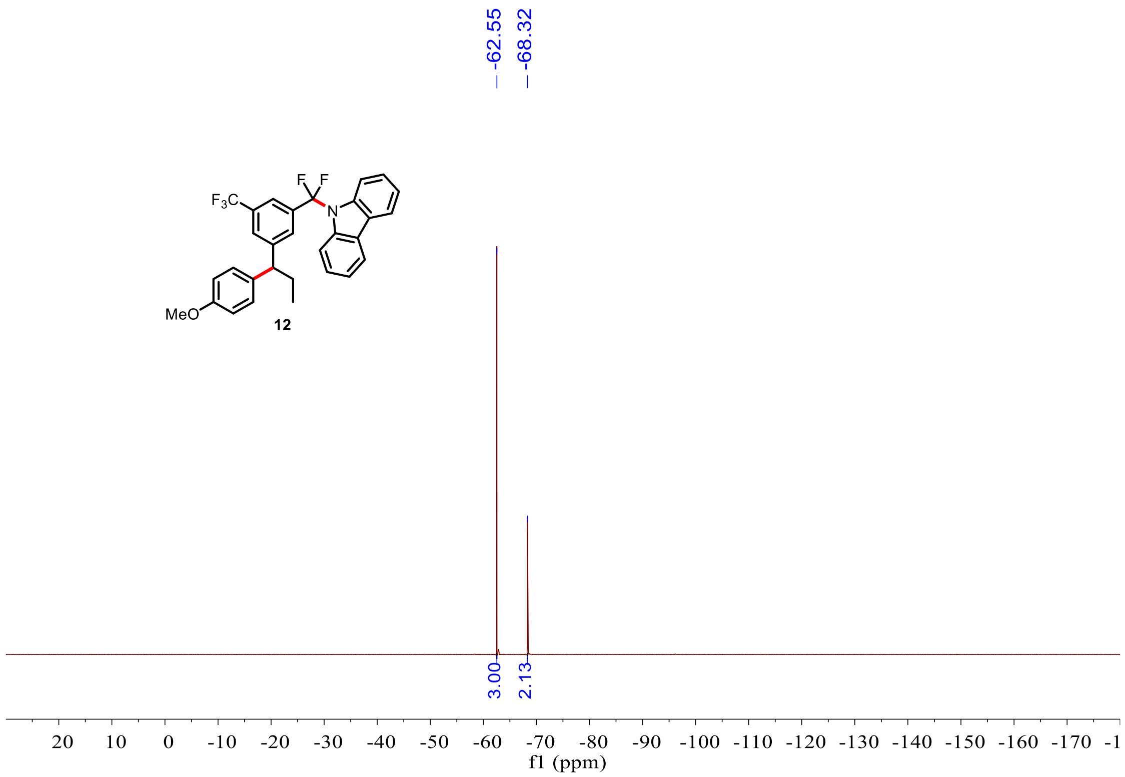

Supplementary Figure 264. ^{19}**F NMR** (471 MHz, $CDCl_3$) spectrum for compound **12**

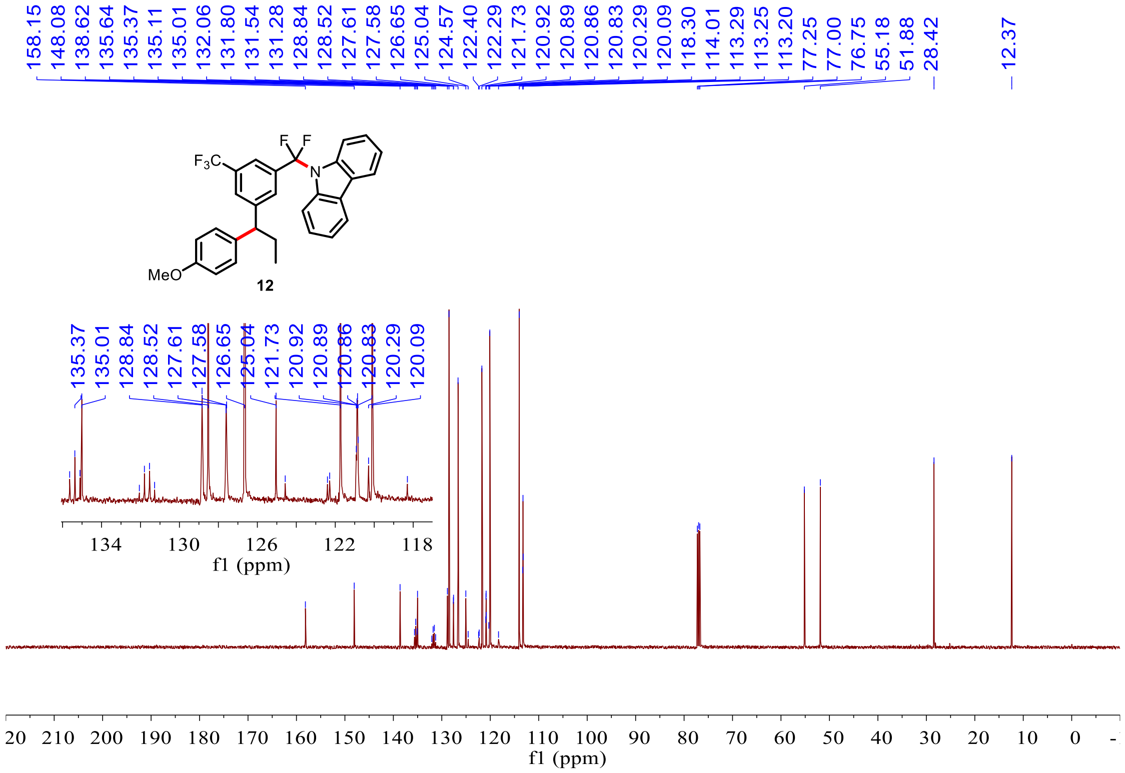

Supplementary Figure 265. ^{13}**C NMR** (126 MHz, $CDCl_3$) spectrum for compound **12**

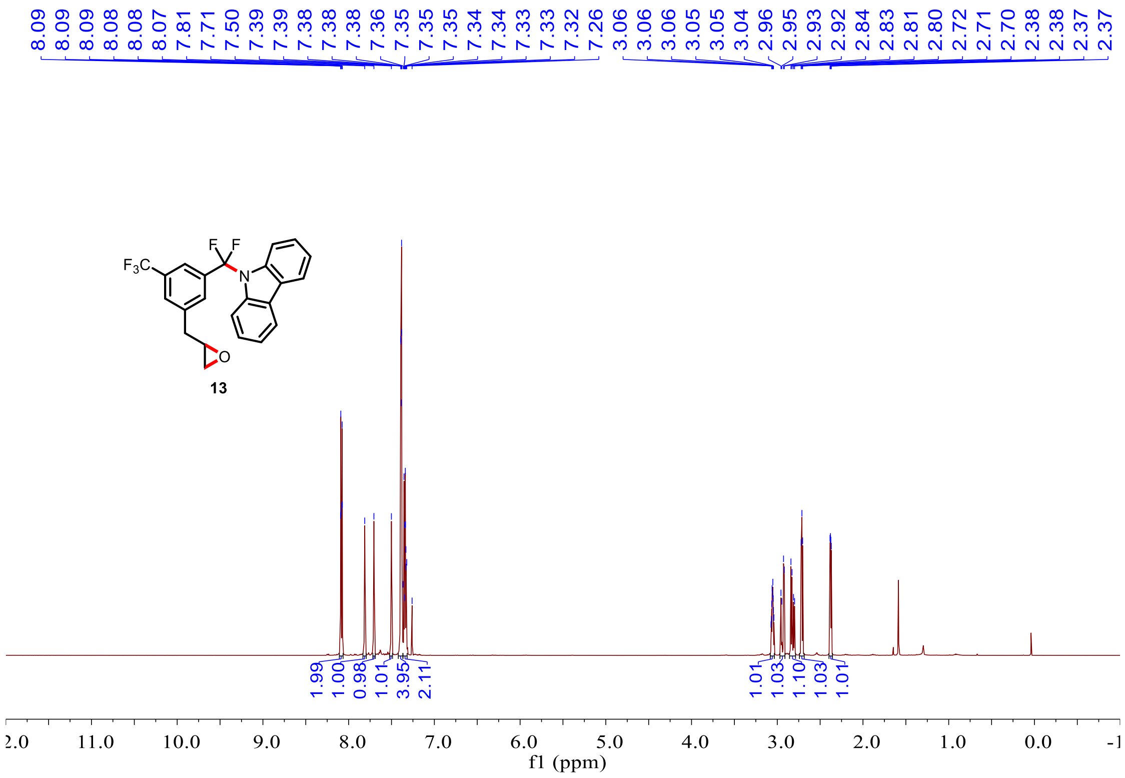

Supplementary Figure 266. **^{1}H NMR** (500 MHz, $CDCl_3$) spectrum for compound **13**

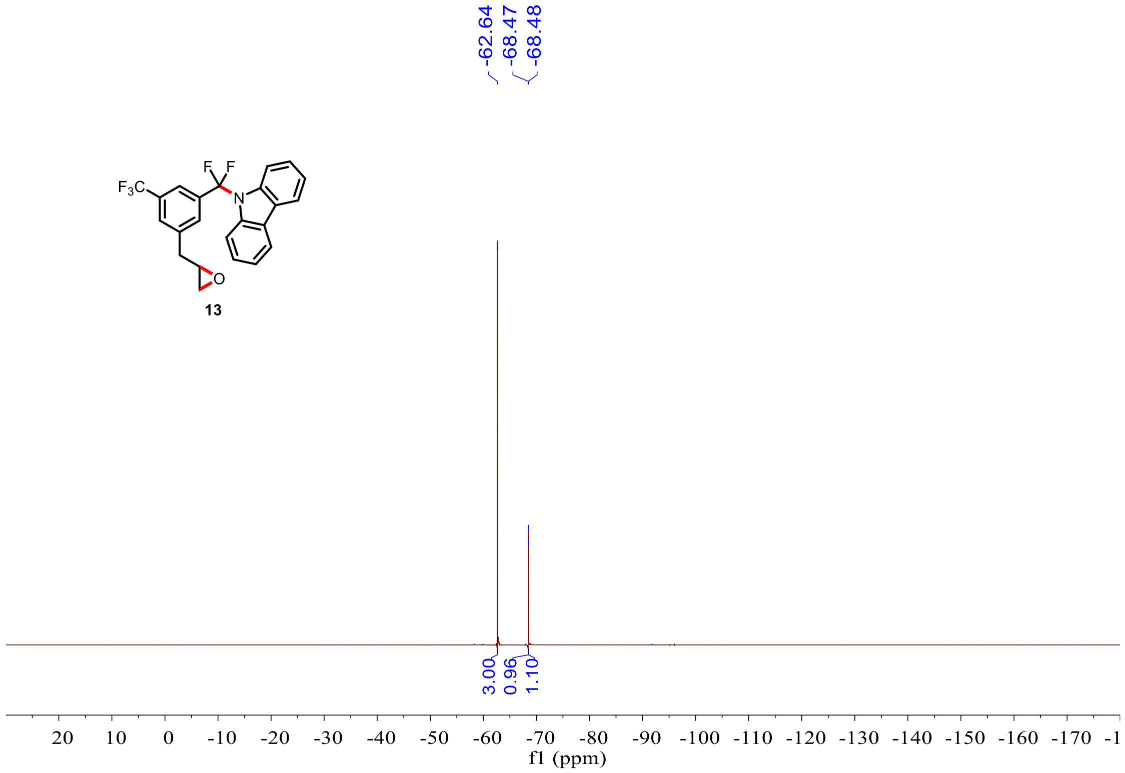

Supplementary Figure 267. **^{19}F NMR** (471 MHz, $CDCl_3$) spectrum for compound **13**

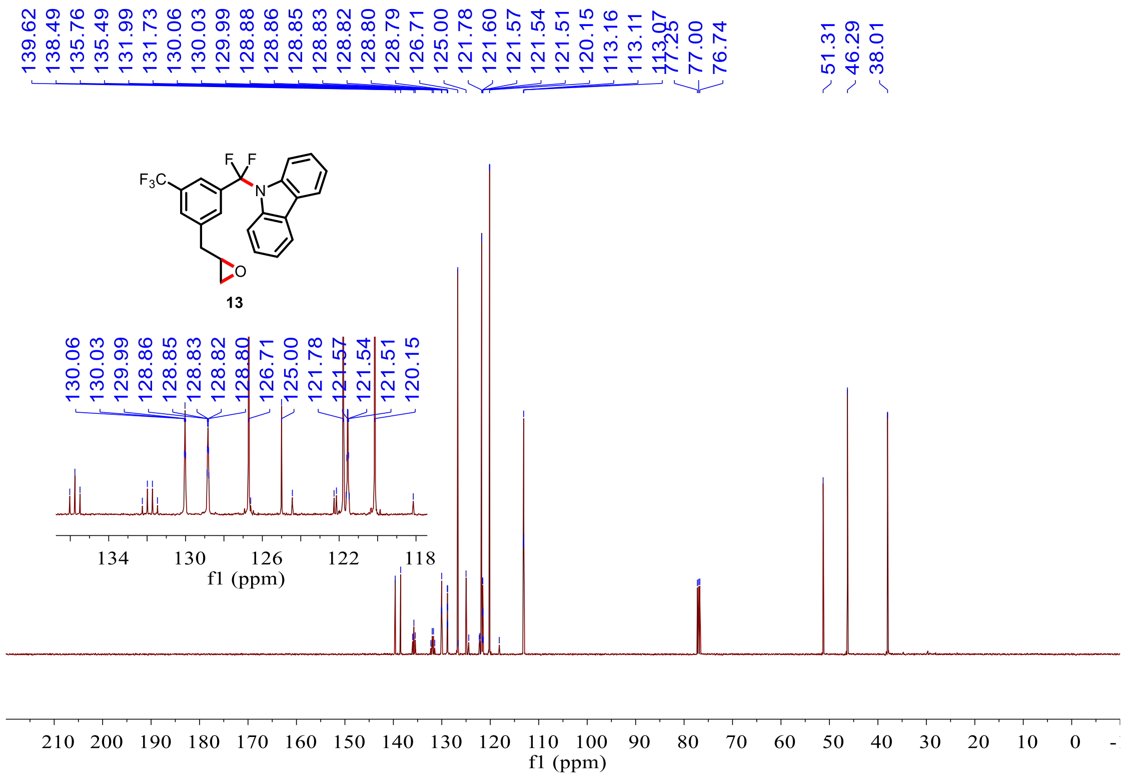

Supplementary Figure 268. ^{13}**C NMR** (126 MHz, $CDCl_3$) spectrum for compound **13**

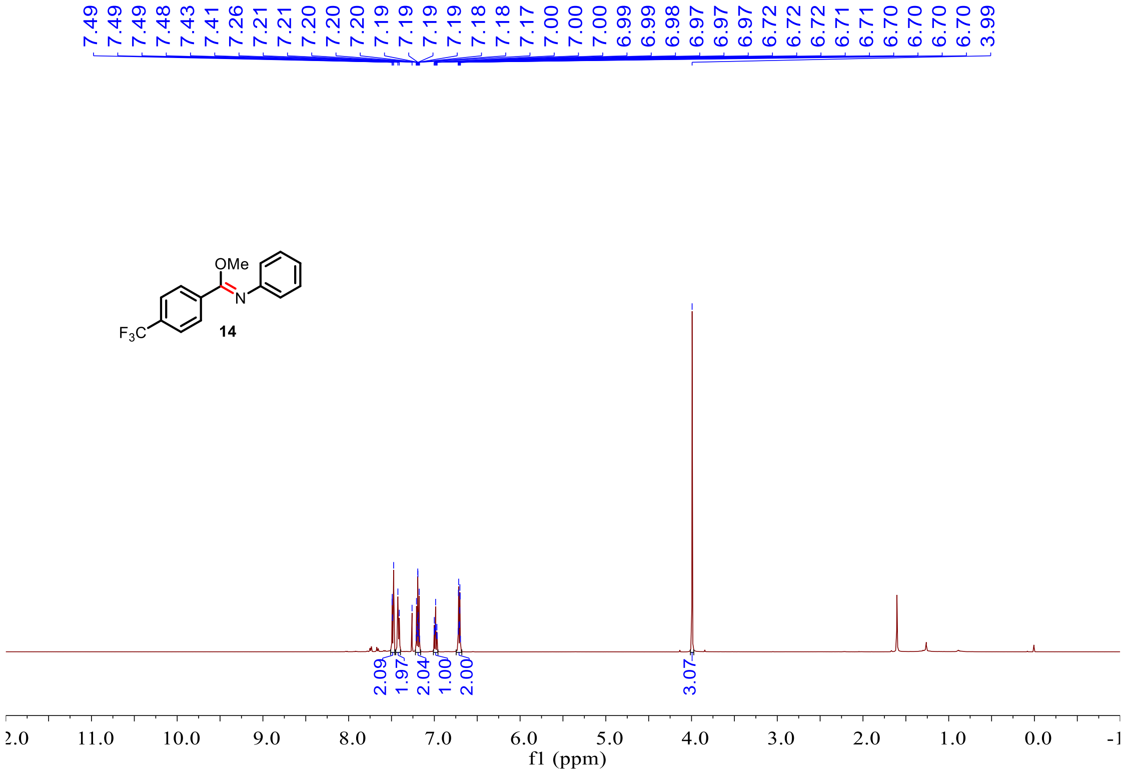

Supplementary Figure 269. ^{1}**H NMR** (500 MHz, $CDCl_3$) spectrum for compound **14**

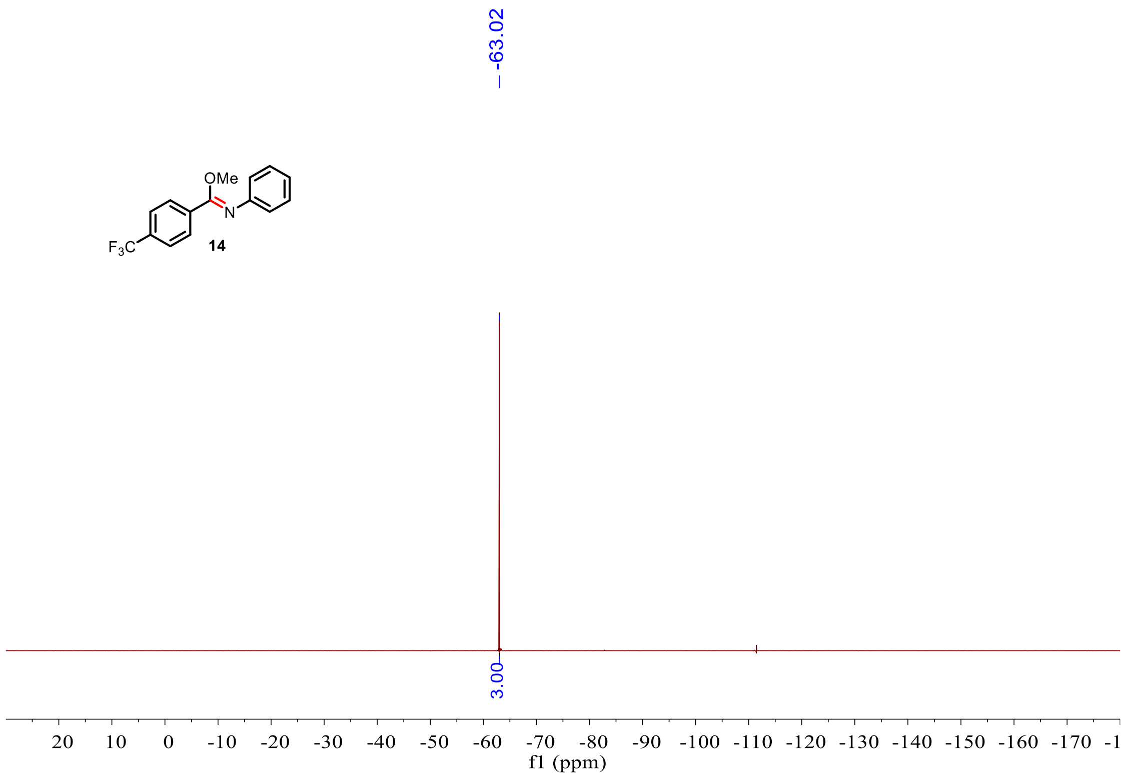

Supplementary Figure 270. ^{19}**F NMR** (471 MHz, $CDCl_3$) spectrum for compound **14**

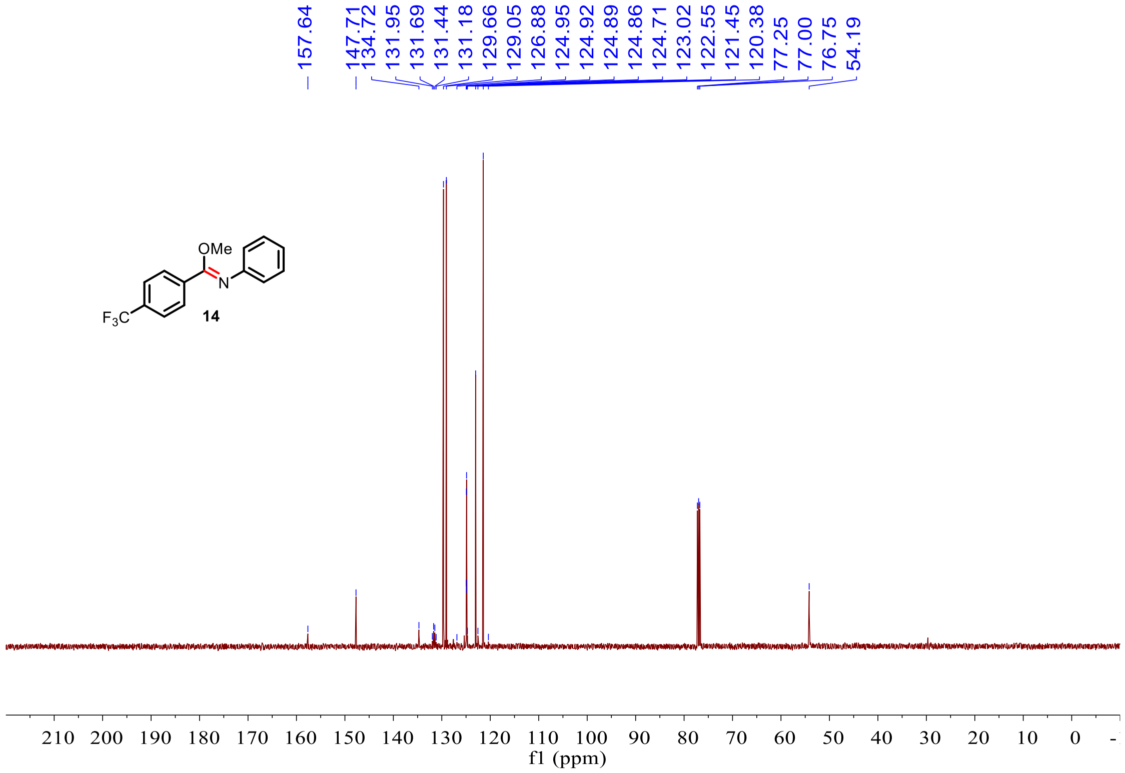

Supplementary Figure 271. ^{13}**C NMR** (126 MHz, $CDCl_3$) spectrum for compound **14**

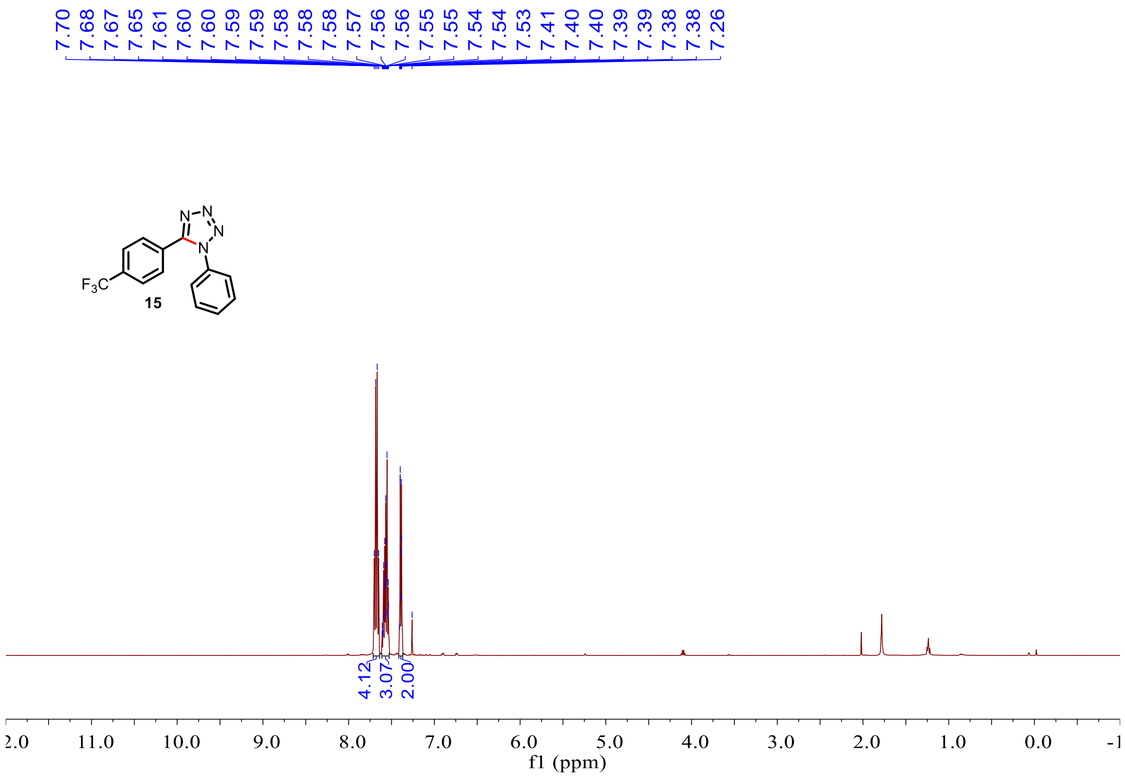

Supplementary Figure 272. ^{1}**H NMR** (500 MHz, $CDCl_3$) spectrum for compound **15**

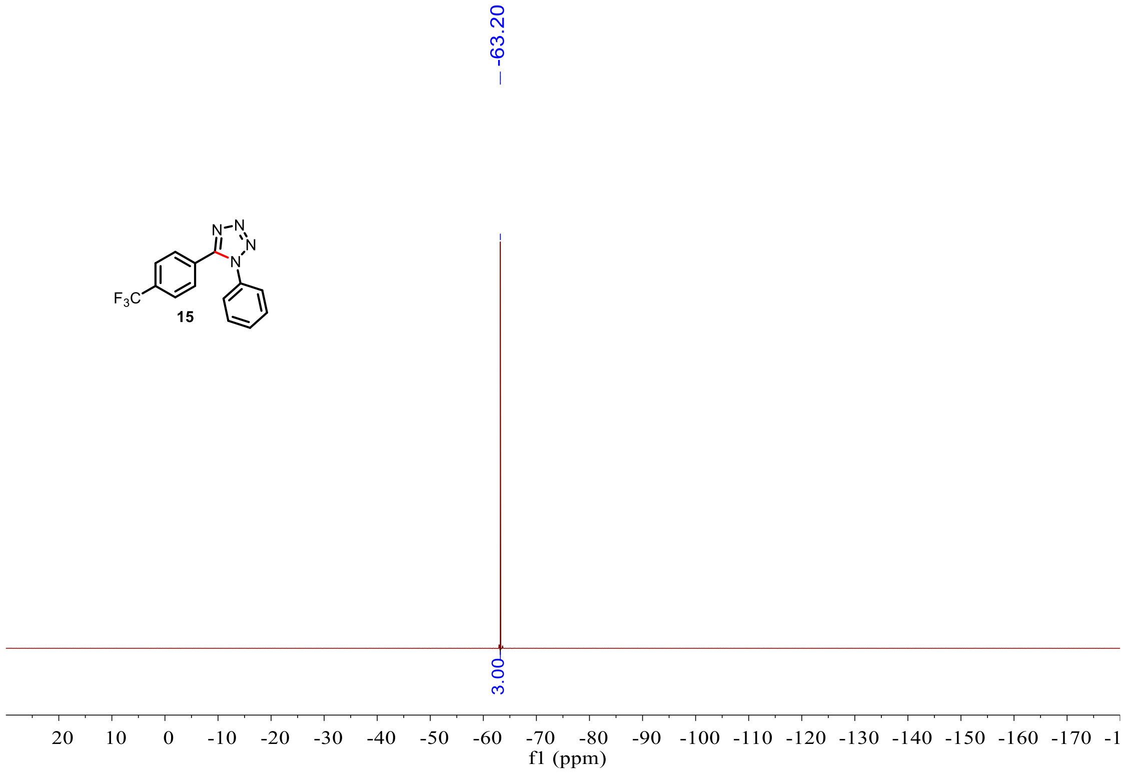

Supplementary Figure 273. ^{19}**F NMR** (471 MHz, $CDCl_3$) spectrum for compound **15**

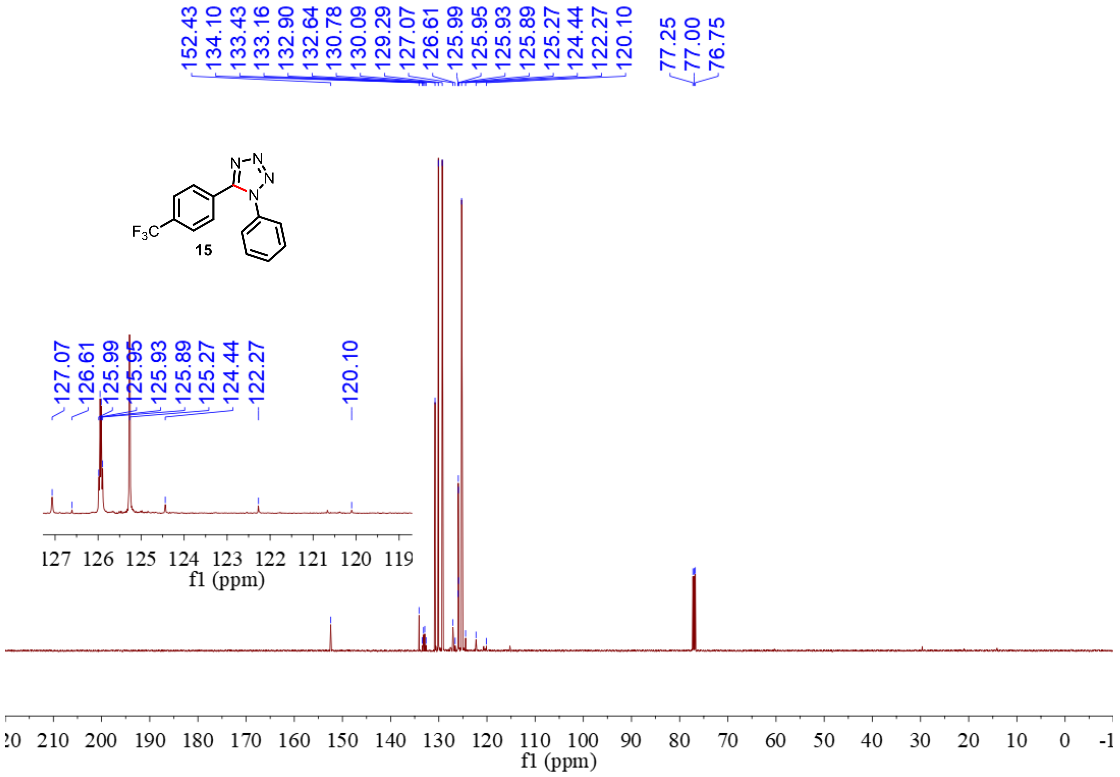

Supplementary Figure 274. ^{13}**C NMR** (126 MHz, $CDCl_3$) spectrum for compound **15**

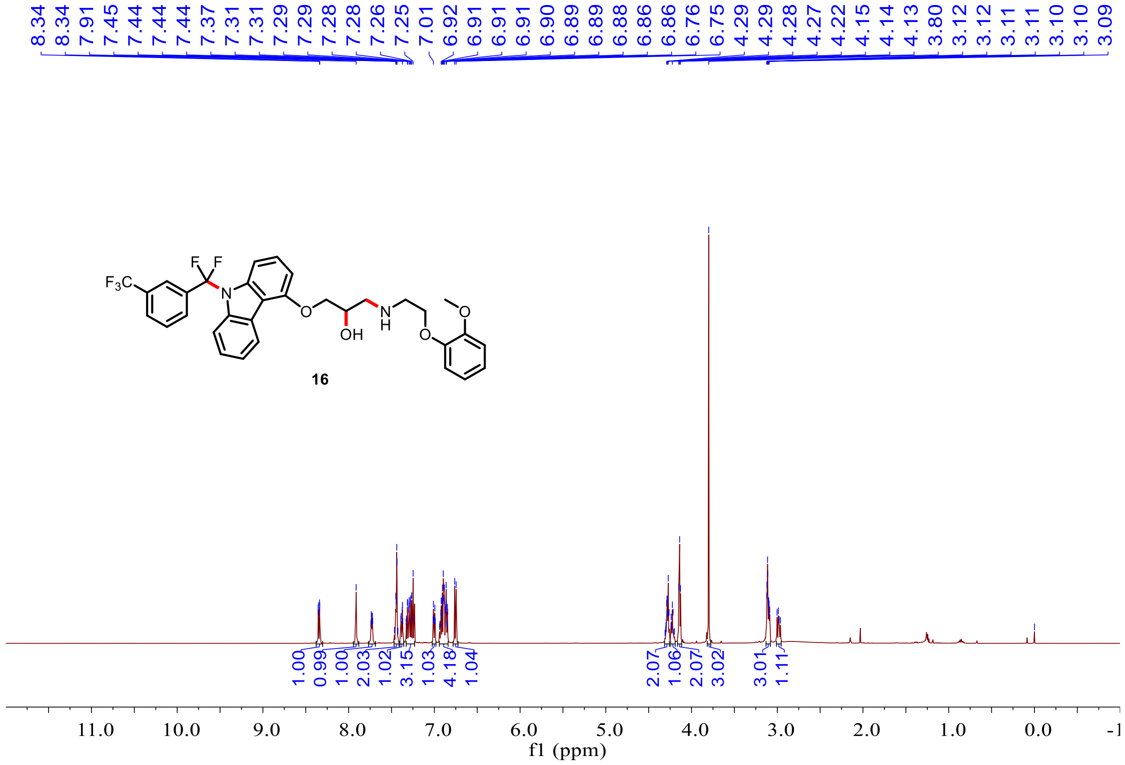

Supplementary Figure 275. ^{1}**H NMR** (500 MHz, $CDCl_3$) spectrum for compound **16**

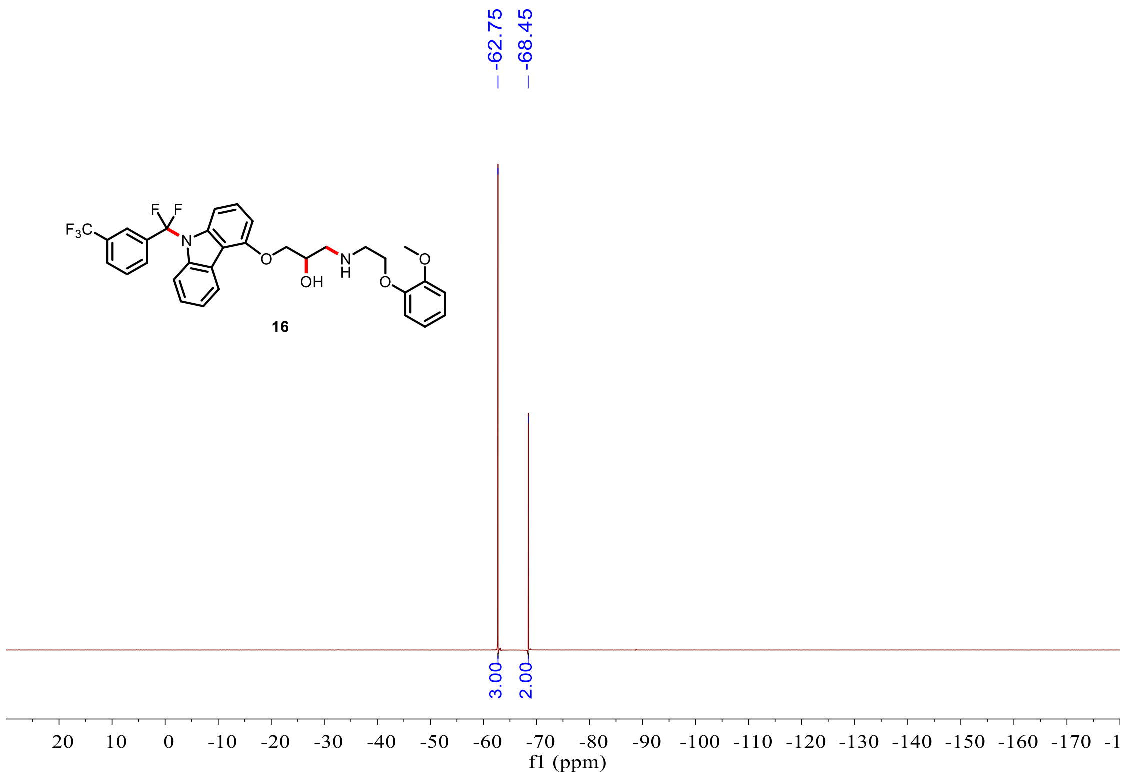

Supplementary Figure 276. ^{19}**F NMR** (471 MHz, $CDCl_3$) spectrum for compound **16**

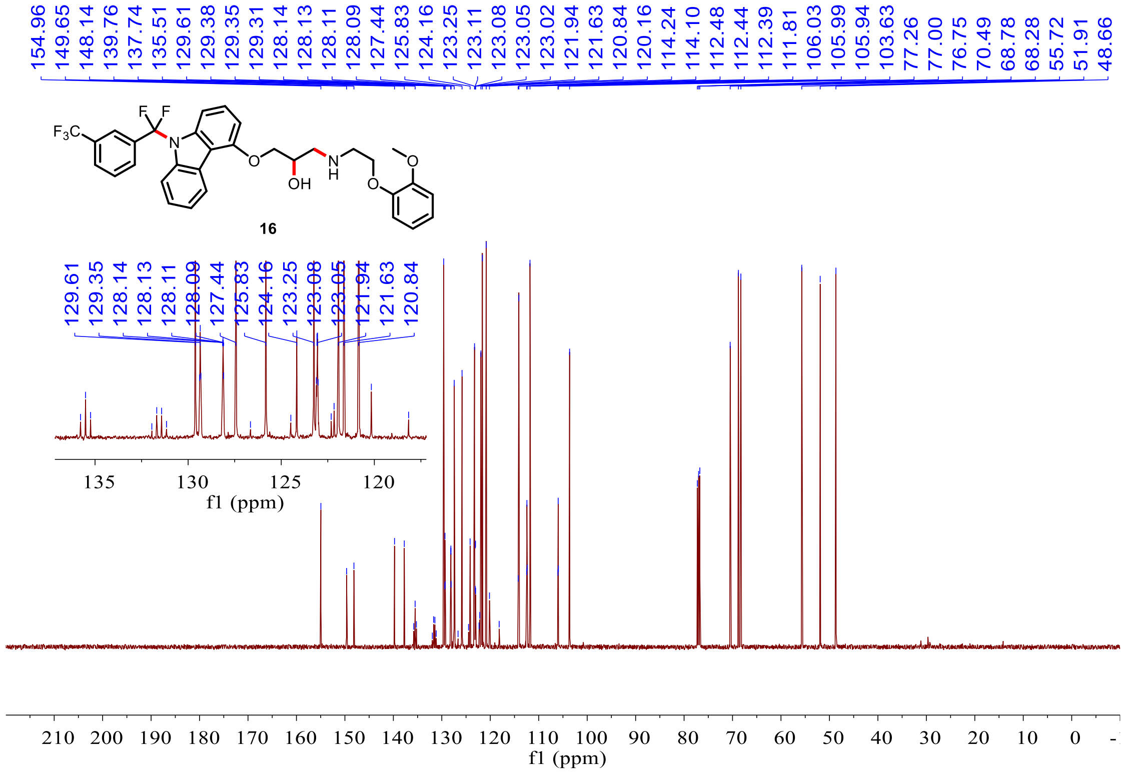

Supplementary Figure 277. ^{13}**C NMR** (126 MHz, $CDCl_3$) spectrum for compound **16**

10. References

1. Luo, Y. et al. Visible-Light-Induced Palladium-Catalyzed Selective Defluoroarylation of Trifluoromethylarenes with Arylboronic Acids. *J. Am. Chem. Soc.* **143**, 13971-13979 (2021).

2. Kenichi, M., Tsuyoshi, M. & Yoshihiro, S. Cobalt-Catalyzed Allylic C(sp^3)–H Carboxylation with CO_2. *J. Am. Chem. Soc.* **139**, 6094-6097 (2017).

3. Das, M. & O'Shea, D. F. Highly Selective Addition of a Broad Spectrum of Trimethylsilane Pro-nucleophiles to *N-tert*-Butanesulfinyl Imines. *Chem. Eur. J.* **21**, 18717-18723 (2015).

4. Peral, D. et al. Strong π-acceptor sulfonated phosphines in biphasic rhodium-catalyzed hydroformylation of polar alkenes. *Catal. Sci. Technol.* **6**, 800-808 (2016).

5. Wang, S. et al. A site isolation-enabled organocatalytic approach to enantiopure γ-amino alcohol drugs. *Tetrahedron*, **74**, 3943-3946 (2018).

6. Enno, L. et al. Aggregation induced emission – emissive stannoles in the solid state. *Chem. Commun.* **56**, 9775-9778 (2020).

7. Khurana, J. M. & Sahoo, P. K. Chemoselective Alkylation of Thiols: A Detailed Investigation of Reactions of Thiols with Halides. *Synth. Commun.* **22**, 1691-1702 (1992).

8. Qiu, J. et al. Ni-Catalyzed Radical-Promoted Defluoroalkylborylation of Trifluoromethyl Alkenes To Access gem-Difluorohomoallylic Boronates. *Org. Lett.* **24**, 2446-2451 (2022).

9. Wrigh, S. E. & Bandar, J. S. A Base-Promoted Reductive Coupling Platform for the Divergent Defluorofunctionalization of Trifluoromethylarenes. *J. Am. Chem. Soc.* **144**, 13032-13038 (2022).

10. Grüger, N. et al. Achiral and Chiral PNP-Pincer Ligands with a Carbazole Backbone: Coordination Chemistry with d^8 Transition Metals. *Inorg. Chem.* **52**, 2050-2059 (2013).

11. Chen, F. et al. Remote Migratory Cross-Electrophile Coupling and Olefin Hydroarylation Reactions Enabled by in Situ Generation of NiH. *J. Am. Chem. Soc.* **139**, 13929-13935 (2017).

12. Kim, H. E., Choi, J.-H. & Chung, W.-J. Fluorine-Assisted Rearrangement of Geminal Azidofluorides to Imidoyl Fluorides. *J. Org. Chem.* **88**, 6878-6889 (2023).

13. Gaussian 09, Revision B.01, Frisch, M. J. et al. Gaussian, Inc., Wallingford CT (2013).

14. Carlo, A. & Vincenzo, B. Toward reliable density functional methods without adjustable parameters: The PBE0 model. *J. Chem. Phys.* **110**, 6158-6170 (1999).

15. Grimme, S., Ehrlich, S. & Goerigk, L. Effect of the damping function in dispersion corrected density functional theory. *J. Comput. Chem.* **32**, 1456-1465 (2011).

16. Florian, W. & Reinhart, A. Balanced basis sets of split valence, triple zeta valence and quadruple zeta valence quality for H to Rn: Design and assessment of accuracy. *Phys. Chem. Chem. Phys.* **7**, 3297-3305 (2005).

17. Pascual-Ahuir, J. L., Silla, E. & Tunon, I. GEPOL: An improved description of molecular surfaces. III. A new algorithm for the computation of a solvent-excluding surface. *J. Comput. Chem.* **15**, 1127-1138 (1994).

18. Miertus, S., Scrocco, E. & Tomasi, J. Electrostatic interaction of a solute with a continuum. A direct utilizaion of AB initio molecular potentials for the prevision of solvent effects. *Chem. Phys.* **55**, 117-129 (1981).

19. Miertus, S. & Tomasi, J. Approximate evaluations of the electrostatic free energy and internal energy changes in solution processes. *Chem. Phys.* **65**, 239-245 (1982).

20. Lu, T. & Chen, Q. Shermo: A general code for calculating molecular thermochemistry properties. *Comput. Theor. Chem.* **1200**, 113249 (2021).

21. Kenichi, F. Formulation of the reaction coordinate. *J. Phys. Chem.* **74**, 4161-4163 (1970).

22. Kenichi, F. The path of chemical reactions - the IRC approach. *Acc. Chem. Res.* **14**, 363-368 (1981).

23. Andreas, D. & Martin, H.-G. Single-Reference ab Initio Methods for the Calculation of Excited States of Large Molecules. *Chem. Rev.* **105**, 4009-4037 (2005).

24. Tao, J. et al. Climbing the Density Functional Ladder: Nonempirical Meta–Generalized Gradient Approximation Designed for Molecules and Solids. *Phys. Rev. Lett.* **91**, 146401(2003).

25. Staroverov, V. N. et al. Comparative assessment of a new nonempirical density functional: Molecules and hydrogen-bonded complexes. *J. Chem. Phys.* **119**, 12129-12137 (2003).

26. Marenich, A. V., Cramer, C. J. & Truhlar, D. G. Universal Solvation Model Based on Solute Electron Density and on a Continuum Model of the Solvent Defined by the Bulk Dielectric Constant and Atomic Surface Tensions. *J. Phys. Chem. B* **113**, 6378-6396 (2009).

27. CYLview 1.0b & Legault, C. Y. Université de Sherbrooke, (2009). (http://www.cylview.org)

28. Lu, T. & Chen, F. Multiwfn: a multifunctional wavefunction analyzer. *J. Comput. Chem.* **33**,

580–592 (2012).

29. Humphrey, W., Dalke, A. & Schulten, K. VMD: Visual molecular dynamics. *J. Mol. Graph.* **14**, 33-38 (1996).

30. Savéant, J. M. Electron transfer, bond breaking, and bond formation. *Acc. Chem. Res.* **26**, 455-461 (1993).

31. Savéant, J. M. A simple model for the kinetics of dissociative electron transfer in polar solvents. Application to the homogeneous and heterogeneous reduction of alkyl halides. *J. Am. Chem. Soc.* **109**, 6788-6795 (1987).

32. Fang, C. et al. Mechanistically Guided Predictive Models for Ligand and Initiator Effects in Copper-Catalyzed Atom Transfer Radical Polymerization (Cu-ATRP). *J. Am. Chem. Soc.* **141**, 7486-7497 (2019).

33. Lee, H. et al. Investigation of the C–N Bond-Forming Step in a Photoinduced, Copper-Catalyzed Enantioconvergent N–Alkylation: Characterization and Application of a Stabilized Organic Radical as a Mechanistic Probe. *J. Am. Chem. Soc.* **144**, 4114-4123 (2022).

34. Lu, T. sobMECP program, (accessed 9.29, 2023). (http://sobereva.com/286).
